# Supplementary material for: Modular synthesis of PAHs from aryl halides and terminal alkynes via photoinduced palladium catalysis
Source: Nat Commun. 2025 Sep 24;16:8349. doi: 10.1038/s41467-025-62466-7 (PMC12460797; doi:10.1038/s41467-025-62466-7)
Supplement: Supplementary file 1 — Supplementary Information [file 41467_2025_62466_MOESM1_ESM.pdf]

Supplementary Information

for

Modular Synthesis of PAHs from Aryl Halides and Terminal Alkynes *via* Photoinduced Palladium Catalysis

Authors: Chen Zhou[1+], Peishang Li[1+], and Ming Chen[1]*

Affiliations: [1]Jiangsu Key Laboratory of Advanced Catalytic Materials & Technology, School of Petrochemical Engineering, Changzhou University, Changzhou 213164, China

*Corresponding author. Email: chenming0228@cczu.edu.cn

[+]These authors contributed equally to this work.

Table of Contents

General Information 3

Supplementary Fig 1. The Parameters of the Blue LEDs 5

Supplementary Fig 2. Preparation of 2-aryl iodinated arenes. 6

Supplementary Fig 3. Preparation of terminal alkynes. 7

Experimental Section 8

Procedure for the Preparation of 2-aryl iodoaryl compound 1l. 8

Procedure for the Preparation of 2-aryl iodoaryl compound 1aa. 9

Supplementary Table 1. Reaction Optimization[a,b,c,d] 10

General Procedure 12

Typical procedure for the synthesis of product 3 12

Supplementary Fig 4. Reaction setup 12

Unsuccessful substrate 44

Synthetic applications for the synthesis of PAHs 44

Synthesis of the PAH 6 44

Synthesis of the PAH 9 45

Synthesis of the PAH 12 46

Synthesis of 9-iodophenanthrene 16 49

Synthesis of unbranched PAHs 17-23. 50

Reaction of aryl diiodobenzene 24 and subsequent transformation of its product. 54

Experimental Procedures for the Mechanistic Studies 56

Control experiments: 56

The Radical Trapping Experiment with TEMPO 57

Radical clock experiments 58

Light On/Off Experiment 59

EPR Experiment 60

Competitive KIE Experiment 63

Synthesis of 3a-D[4] 63

Typical procedure for the synthesis of 1a-D 66

Competitive KIE Experiment of intermolecular products 67

NMR Spectra 68

Supplementary Reference 162

General Information

Unless noted otherwise, all the solvents and commercially available reagents were purchased and used directly. Benzene, 1,4-dioxaneand tetrahydrofuran were distilled freshly over sodium, benzotrifluoride was distilled freshly over P_2O_5, DCM was distilled freshly over CaH_2 and carefully freeze-pump-thawed. Sensitive reagents and solvents were transferred under nitrogen into a nitrogen-filled glovebox with standard techniques. Reactions were monitored with thin layer chromatography (TLC) using silica gel 60 F-254 plates. TLC plates were normally visualized by UV irradiation (254 nm or 365 nm), stained with basic $KMnO_4$. Flash chromatography was performed using silica gel 60 (200−300 mesh). Vials (15 x 45 mm 1 dram (4 mL) / 17 x 60 mm 3 dram (7.5 mL) with PTFE lined cap attached) were purchased from Qorpak and flame-dried or put in an oven overnight and cooled in a desiccator. Mass (HRMS) analysis was obtained using Agilent 6200 Accurate-Mass TOF LC/MS system with Electrospray Ionization (ESI). Nuclear magnetic resonance spectra (^{1}H NMR and ^{13}C NMR) were recorded with Bruker AVANCE Ⅲ–300 (300 MHz, ^{1}H at 300 MHz, ^{13}C at 75 MHz), 400 (400 MHz, ^{1}H at 400 MHz, ^{13}C at 101 MHz) or 600(600 MHz, ^{1}H at 600 MHz, ^{13}C at 151 MHz).^{19}F NMR spectra were recorded on Bruker AVANCE Ⅲ–300.Unless otherwise noted, all spectra were acquired in $CDCl_3$. Chemical shifts are reported in parts per million (ppm, δ), downfield from tetramethylsilane (TMS, δ = 0.00 ppm) and are referenced to residual solvent ($CDCl_3$, δ = 7.26 ppm (^{1}H) and 77.00 ppm (^{13}C). Coupling constants were reported in Hertz (Hz). Data for ^{1}H NMR spectra were reported as follows: chemical shift (ppm, referenced to protium, s = singlet, d = doublet, t = triplet, q = quartet, quin = quintet, dd = doublet of doublets, td = triplet of doublets, ddd = doublet of doublet of doublets, m = multiplet, coupling constant (Hz), and integration). The electron paramagnetic resonance (EPR) spectra were recorded on a Bruker E500 spectrometer. All other materials were obtained from Energy Chemical and were used as received.

Safety Note on Benzene Use and Handling

Benzene is a known carcinogenic and toxic solvent and must be handled with strict care. In this study, all reactions using benzene were conducted on a small scale (typically 2 mL per 0.2 mmol reaction) in a nitrogen-filled glovebox and/or a well-ventilated fume hood to minimize exposure. After reaction completion, benzene was removed under reduced pressure using a rotary evaporator, with the exhaust connected directly to an active fume hood to ensure safe venting of any residual vapors.

Researchers are strongly advised to:

- Wear appropriate personal protective equipment (PPE), including gloves, lab coat, and safety goggles.
- Perform all operations involving benzene within a certified fume hood.
- Collect all benzene-containing waste in designated sealed waste containers and dispose of it according to local institutional and environmental regulations.
- Avoid direct inhalation and skin contact at all stages of handling.

All experimental procedures involving benzene should comply with institutional chemical safety protocols and relevant legal requirements.

Supplementary Fig 1. The Parameters of the Blue LEDs

Test Report of LED Photoelectric Test System

Test project:	LED spectral analysis	
Test equipment:	Photochromic-electric integrated test system	
The test identification	Product model: 3 W Blue LED	
	Ambient temperature: 27 ℃	Ambient humidity: 65%
	Test organization: spectrotest department	
Spectral relative energy distribution curve		

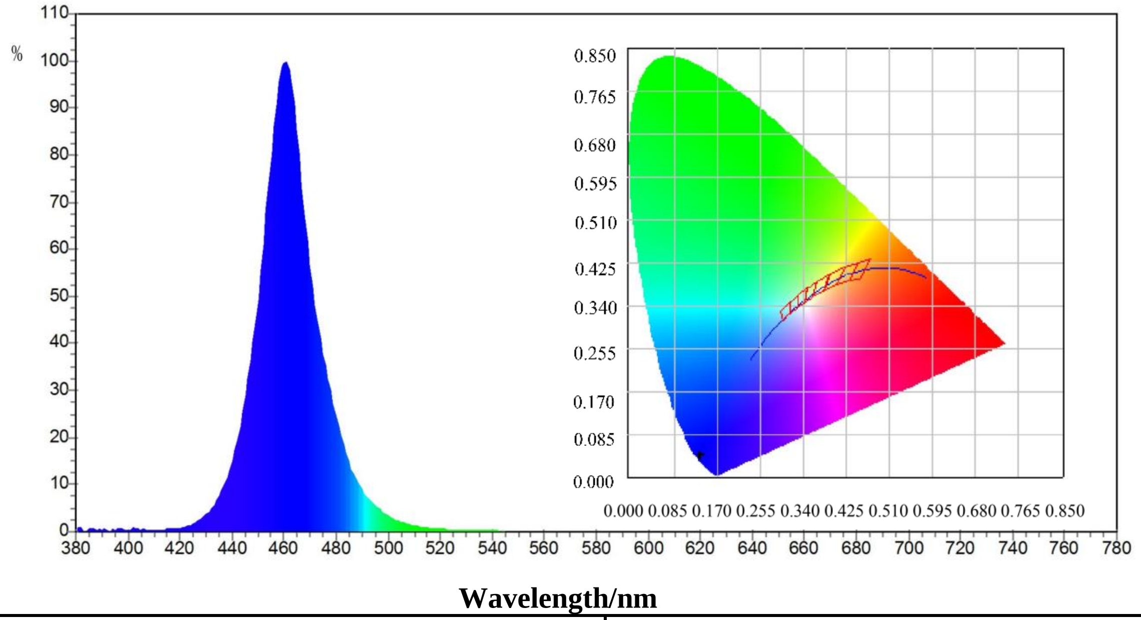

Spectrum parameter		Photoelectric parameter	
peak wavelength:	453.6 nm	lighting current:	3.0 mA
main wavelength:	460.2 nm	preheating time:	500 ms
centroid wavelength:	445.7 nm	test current:	700.0 mA
central wavelength:	446.0 nm	direct voltage:	3.52 V
half-wave width:	22.0 nm	light flow:	40547.6 mlm
colour temperature:	K	light efficiency:	16.456 lm/w
chromaticity coordinate (x, y):	0.1467, 0.0349	optical power:	896.0946 mv
chromaticity coordinate (u, v):	0.1877, 0.0670	backward voltage:	5.00 V
CRI (color rendering index):	0	leakage current:	0.0 µA
colour purity:	0.984		

Note:Guanghong 45, 460
-462

Supplementary Fig 2. Preparation of 2-aryl iodinated arenes.

The 2-iodo biaryl were prepared according to the previously reported literature. The **1b~c**[1],**1f~g**[1], **1r**[1], **1d**[2], **1k**[2],**1p**[2], **1ad**[2,] **24**[2], **1e**[3], **1h~i**[4], **1t**[4], **1j**[5], **1m**[6], **1n~o**[7], **1q**[8], **1s**[9], **1u**[10], **1v**[11], **1w**[12], **1x~y**[13], **1z**[14] are known compounds. **1l** and **1aa** are unknown compounds.

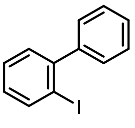

1a

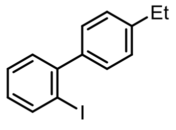

1b

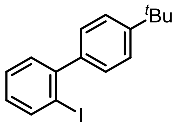

1c

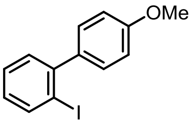

1d

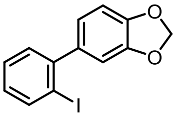

1e

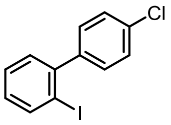

1f

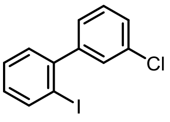

1g

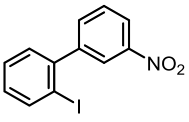

1h

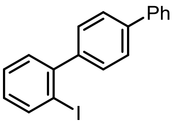

1i

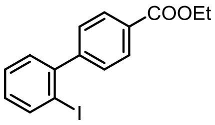

1j

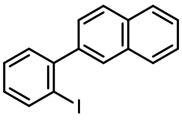

1k

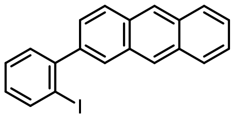

1l

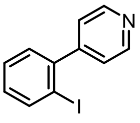

1m

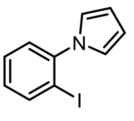

1n

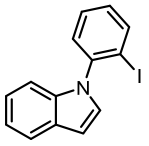

1o

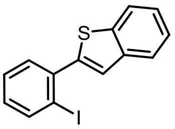

1p

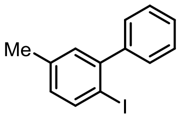

1q

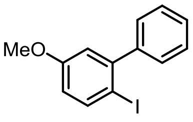

1r

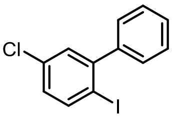

1s

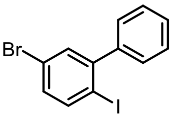

1t

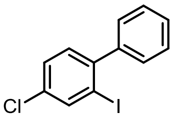

1u

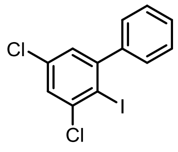

1v

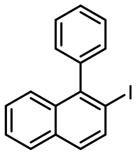

1w

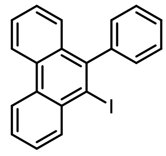

1x

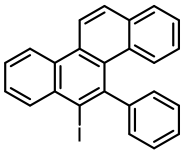

1y

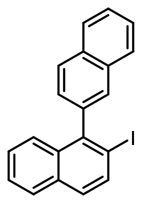

1z

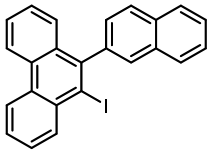

1aa

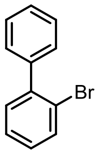

1a-Br

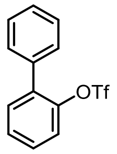

1a-OTf

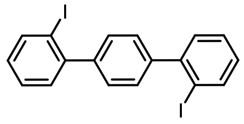

24

Supplementary Fig 3. Preparation of terminal alkynes.

The terminal alkynes **2ac~2ae**[15] **2af**[16], **2ag**[17] were prepared according to the previously reported literature. The others are commercially available and were used as received.

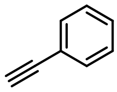

2a

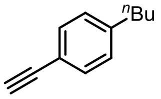

2b

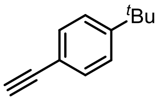

2c

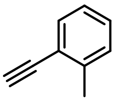

2d

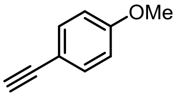

2e

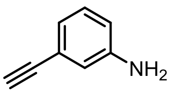

2f

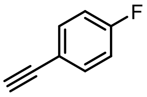

2g

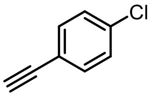

2h

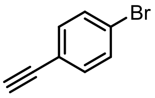

2i

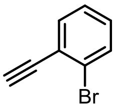

2j

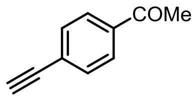

2k

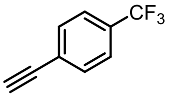

2l

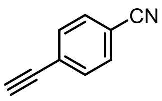

2m

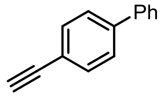

2n

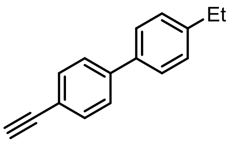

2o

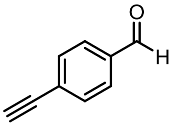

2p

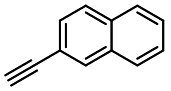

2q

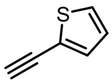

2r

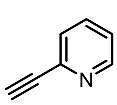

2s

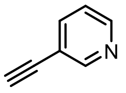

2t

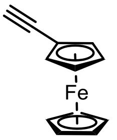

2u

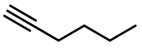

2v

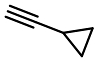

2w

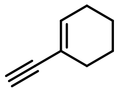

2x

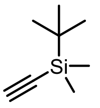

2y

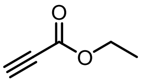

2z

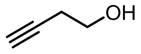

2aa

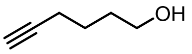

2ab

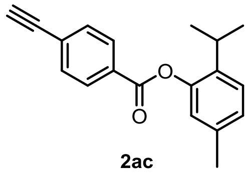

2ac

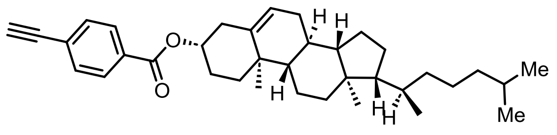

2ad

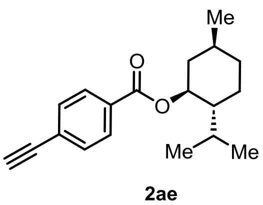

2ae

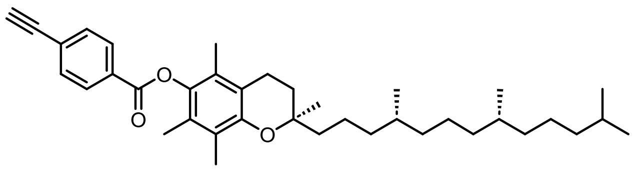

2af

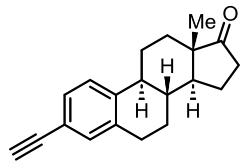

2ag

Experimental Section

Procedure for the Preparation of 2-aryl iodoaryl compound 1l.

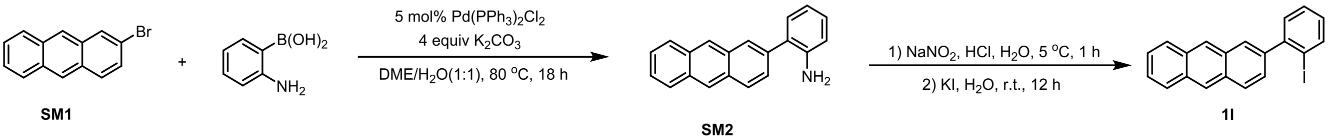

Step 1: To a solution of the **SM-1** (514 mg, 2.0 mmol, 1.0 equiv.) in dimethoxyethane/water (1:1, 0.25 M) under nitrogen atmosphere was added $Pd(PPh_3)_2Cl_2$ (70.2 mg, 5 mol%), K_2CO_3 (1.1 g, 8.0 mmol, 4 equiv), (2-aminophenyl)boronic acid (328 mg, 2.4 mmol, 1.2 equiv). After the reaction mixture was stirred for 12 hours at 80 ℃ in a sealed tube, it was allowed to cool to room temperature. The reaction mixture was extracted with EtOAc (3×10 mL), and the combined organic phases were washed with 50 mL of water. The organic phases were dried over anhydrous Na_2SO_4, and concentrated in *vacuo*. Purification by column chromatography on silica geleluting with petroleum ether: ethyl acetate = 10:1 gave **SM-2** in 87% isolated yield (470 mg).

Step 2: A solution of concentration HCl (36-38%; 0.88 mL, 2.5 mmol) in water (3.5 mL) was added slowly to the prepared **SM-2** (1.75 mmol) with stirring. An aqueous solution of $NaNO_2$ (20%; 1.04 g, 3 mmol) was added to the reaction mixture below 5 ℃ within 10 min and stirred for 1 hour at the same temperature. An aqueous solution of KI (0.51 g, 3.5 mmol) in water (3.5 mL) was added and the reaction mixture was stirred at room temperature overnight. EtOAc (20 mL) was added to the reaction mixture and the organic phase was treated with saturated $NaHSO_3$ to decolorize. The aqueous phase was extracted with EtOAc (3×10 mL). The combined organic phases were dried over anhydrous Na_2SO_4 and concentrated in *vacuo*. Purification by column chromatography on silica geleluting with petroleum ether gave **1l** in 62% isolated yield (411 mg). M.p. 145.9 - 146.9 °C. **2-(2-Iodophenyl)anthracene (1l).** **^{1}H NMR (400 MHz, $CDCl_3$)** δ 8.47 (s, 2H), 8.07 – 8.01 (m, 4H), 7.95 (s, 1H), 7.51 – 7.45 (m, 5H), 7.10 (dt, *J* = 8.0, 4.4 Hz, 1H). **^{13}C NMR (101 MHz, $CDCl_3$)** δ 146.5, 141.0, 139.6, 132.0, 131.9, 131.2, 130.8, 130.4, 128.9, 128.21, 128.19, 128.13, 128.08, 127.6, 127.4, 126.6, 126.1, 125.51, 125.49, 98.6. **HRMS (EI)** calcd for $C_{20}H_{13}I$ $[M]^+$: 380.0056, found 380.0061.

Procedure for the Preparation of 2-aryl iodoaryl compound 1aa.

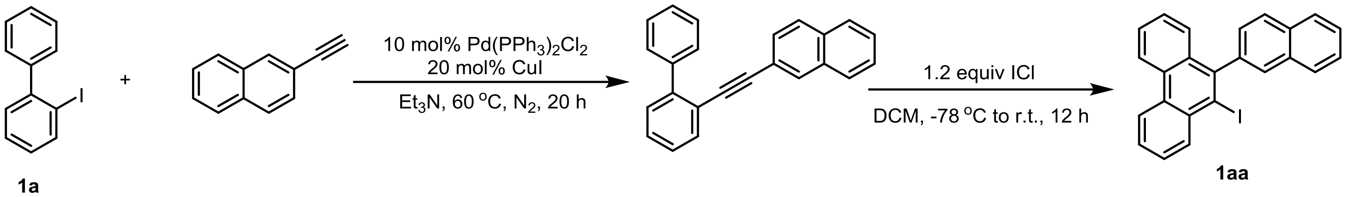

An oven-dried 20 mL reaction flask was charged with 2-iodo-1,1'-biphenyl **1a** (1.4 g, 5.0 mmol, 1.0 equiv.), 2-ethynylnaphthalene (913.2 mg, 6 mmol, 1.2 equiv.), $Pd(PPh_3)_2Cl_2$ (175.5 mg, 0.25 mmol, 5 mol%) and CuI (95.1 mg, 0.5 mmol, 10 mol%). It was directly transferred in a nitrogen-filled glovebox with caps. In the glovebox, 12 mL of degassed Et_3N were added to the vial. The vial was tightly sealed, transferred out of glovebox and stirred at 60 ^oC for 20 hours. After completion of the reaction, the resulting mixture was diluted with acetone (30 mL), filtered (Celite), and concentrated under a reduced pressure. The residue was purified by column chromatography on silica gel (eluent: Petroleum ether/ Ethyl acetate = 70:1) to afford 2-([1,1'-biphenyl]-2-ylethynyl)naphthalene in 89% isolated yield (1.35 g).

To a stirred solution of 2-([1,1'-biphenyl]-2-ylethynyl)naphthalene (1.2 g, 4 mmol) in DCM (15 mL) at -78 ^oC was added ICl (844.5 mg, 5.2 mmol, 1.3 equiv, ICl dissolved in 8 ml DCM), the mixture was stirred at room temperature for 12 hours. After filtration and concentration, the crude was purified by column chromatography on silica gel (eluent: Petroleum ether/ Ethyl acetate = 40:1), yielding the desired compound **1aa** as a yellow solid (1.4 g, 82% yield). **9-Iodo-10-(naphthalen-2-yl)phenanthrene (1aa).** **^{1}H NMR (400 MHz, $CDCl_3$)** δ 8.75 (dd, J = 16.0, 8.0 Hz, 2H), 8.55 – 8.48 (m, 1H), 8.06 (d, J = 8.4 Hz, 1H), 8.00 (d, J = 7.2 Hz, 1H), 7.95 – 7.89 (m, 1H), 7.80 (s, 1H), 7.77 – 7.65 (m, 3H), 7.64 – 7.55 (m, 2H), 7.45 - 7.39 (m, 3H). **^{13}C NMR (101 MHz, $CDCl_3$)** δ 145.1, 142.7, 134.7, 133.3, 132.8, 132.4, 132.4, 130.5, 130.2, 128.9, 128.7, 128.2, 128.1, 128.1, 127.9, 127.6, 127.1, 127.0, 126.3, 126.3, 122.6, 122.6, 106.6. **HRMS (EI)** calcd for $C_{24}H_{15}$ $[M-I]^+$: 303.1168, found 303.1165.

Supplementary Table 1. Reaction Optimization[a,b,c,d]

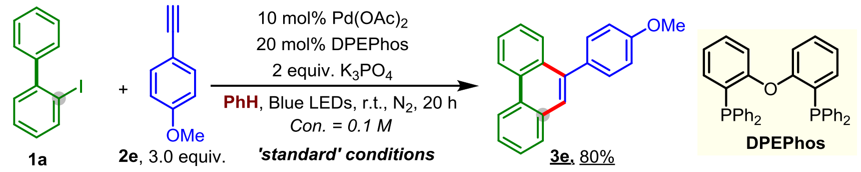

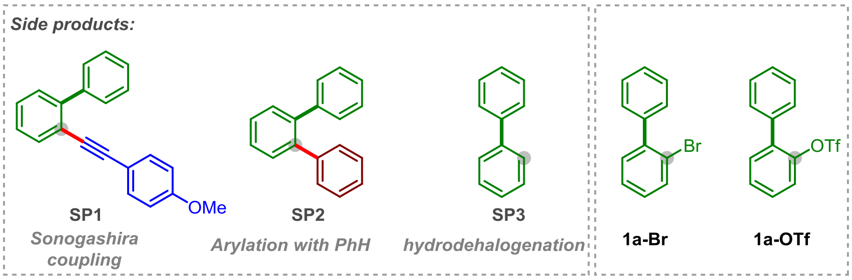

Entry	Variations from the 'standard' conditions	Yield (%)[a] of **3e/SP1/SP2**
1	None	80/0/0
2	Without **$Pd(OAc)_2$ (C1)**	trace/0/0
3	Without **DPEPhos (L1)**	0/19/0
4	Without **K_3PO_4 (B1)**	18/0/0
5	Without **Blue LEDs**	0/14/0
6	**C2-9** instead of **$Pd(OAc)_2$**	Listed below
7	**L2-9** instead of **DPEPhos**	Listed below
8	**B2-9** instead of **K_3PO_4**	Listed below
9	carried out in air	trace/52/0
10	Without **Blue LEDs**, carried out in 80 °C	0/49/2
11	solvent = THF	4/0/0
12	solvent = dioxane	14/3/0
13	solvent = PhF	38/0/0
14	solvent = DMSO	22/8/0
15	1.2 equiv. **2a**	36/0/22
16	1.5 equiv. **2a**	44/0/16
17	2.0 equiv. **2a**	54/0/9
18	2.5 equiv. **2a**	67/0/8
19[c]	**1a-Br** instead of **1a**	74/0/6
20	**1a-OTf** instead of **1a**	9/18/7

$Pd(PPh_3)_4$	$Pd_2(dba)_3$	$Pd(TFA)_2$	$(PPh_3)_2PdCl_2$
**C2**, 36/0/2	**C3**, 76/0/5	**C4**, 38/2/3	**C5**, 48/0/0
$(XantPhos)PdCl_2$	$(dppf)PdCl_2$	$(CH_3CN)_2PdCl_2$	$PdCl_2$
**C6**, 45/0/0	**C7**, 66/0/0	**C8**, 43/0/0	**C9**, 29/0/4

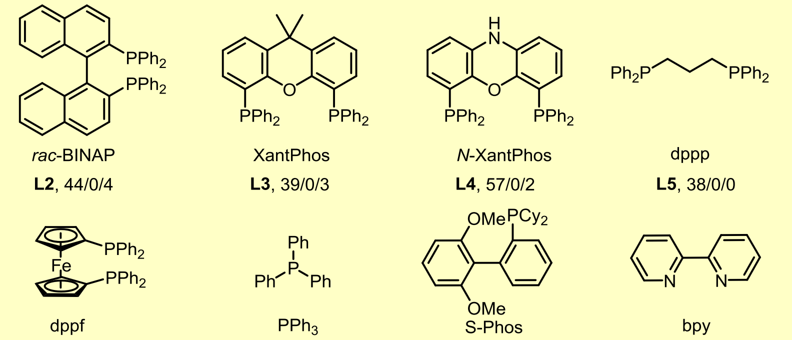

rac-BINAP	XantPhos	*N*-XantPhos	dppp
**L2**, 44/0/4	**L3**, 39/0/3	**L4**, 57/0/2	**L5**, 38/0/0
dppf	PPh_3	S-Phos	bpy
**L6**, 65/0/8	**L7**, 45/0/0	**L8**, 43/0/3	**L9**, 0/9/0

K_2CO_3	$NaHCO_3$	Cs_2CO_3	CH_3ONa
**B2**, 41/0/2	**B3**, 30/0/0	**B4**, 44/38/2	**B5**, 5/0/0
t-BuOLi	*t*-BuONa	*t*-BuOK	Cy_2NMe
**B6**, 54/0/10	**B7**,41/19/0	**B8**, 49/39/3	**B9**, 23/0/8

[a]Each reaction was run on a 0.1 mmol scale in a sealed 4 mL vial for 20. [b]Yields of **3e** were determined by ^1H NMR using CH_2Br_2 as the internal standard. [c]$(dppf)PdCl_2$ was used as the catalyst. [d]TFA = trifluoroacetate, dba = dibenzylideneacetone, dppf = 1,1'-bis(diphenylphosphino)ferrocene.

Our investigation commenced with the reaction between 2-iodobiphenyl (**1a**) and *para*-methoxyphenylacetylene (**2e**) as model substrates (Supplementary Table 1). Through extensive optimization, the desired product **3e** was obtained in an excellent yield of 80% under the optimized conditions. The reaction was conducted using $Pd(OAc)_2$ as the catalyst, DPEPhos as the ligand, and K_3PO_4 as the base in benzene under blue LED irradiation and a nitrogen atmosphere. Under these standard conditions, no detectable formation of the Sonogashira coupling byproduct (**SP1**) or the solvent-derived arylation byproduct (**SP2**) was observed. Additionally, hydrodehalogenation byproduct (**SP3**) was not detected, which can be attributed to the presence of excess alkyne and the solvent environment that likely suppresses this side reaction. To evaluate the necessity of each reaction component, a series of control experiments were conducted. The results clearly indicated that the palladium catalyst, the phosphine ligand, the base, and blue light irradiation are all essential for this transformation (entries 2–5). Excluding any of these components led to either significantly reduced efficiency or complete inhibition of the desired product formation. Subsequently, various Pd(0) and Pd(II) complexes were screened (entry 6). Among them, $Pd_2(dba)_3$, (dppf)$PdCl_2$, $(PPh_3)_2PdCl_2$, $(CH_3CN)_2PdCl_2$, and (XantPhos)$PdCl_2$ provided moderate to good yields, whereas $Pd(PPh_3)_4$, $Pd(TFA)_2$, and $PdCl_2$ exhibited significantly lower efficiencies. A range of ligands were also examined (entry 7). Bidentate phosphine ligands such as dppf (L2) and N-XantPhos (L3) demonstrated good catalytic efficiency. Other mono- and bidentate phosphine ligands (L4–L8) yielded moderate amounts of the desired product, while the bidentate nitrogen ligand bpy (L9) failed to deliver any target product. These results highlight the superior performance of bidentate phosphine ligands, particularly DPEPhos and dppf, in this reaction. Further optimization focused on the base (entry 8). Both inorganic bases (e.g., K_2CO_3, Cs_2CO_3) and strong organic bases (*e.g.*, *t*-BuOLi, *t*-BuONa, *t*-BuOK) promoted the reaction to varying degrees. Notably, K_3PO_4 delivered high efficiency, while K_2CO_3, *t*-BuOLi, and *t*-BuONa provided moderate yields. Interestingly, Cs_2CO_3 and *t*-BuOK significantly increased the formation of the Sonogashira side product **SP1**, indicating a shift in reaction selectivity under these conditions. Performing the reaction in ambient air resulted in only trace amounts of the desired product, suggesting that oxygen inhibits the reaction (entry 9). Heating the reaction mixture in the absence of blue light led predominantly to the formation of **SP1** (entry 10), emphasizing the crucial role of photoactivation. Solvent screening revealed benzene as the optimal solvent, with other solvents such as THF, dioxane, PhF, and DMSO leading to lower yields (entries 11–14). The effect of alkyne loading was also investigated (entries 15–18), showing that increasing the amount of **2e** to 3.0 equivalents significantly improved the yield of **3e** while reducing the formation of **SP2**. This suggests that excess alkyne suppresses competing side reactions. In addition to 2-iodobiphenyl (**1a**), its analog 2-bromobiphenyl (**1a-Br**) also afforded good yields under optimized conditions with (dppf)$PdCl_2$ as the catalyst (entry 19). However, [1,1'-biphenyl]-2-yl trifluoromethanesulfonate (**1a**-OTf) exhibited sluggish reactivity, and attempts to enhance its activity by adding NaI or KI were unsuccessful (entry 20).

General Procedure

Typical procedure for the synthesis of product 3

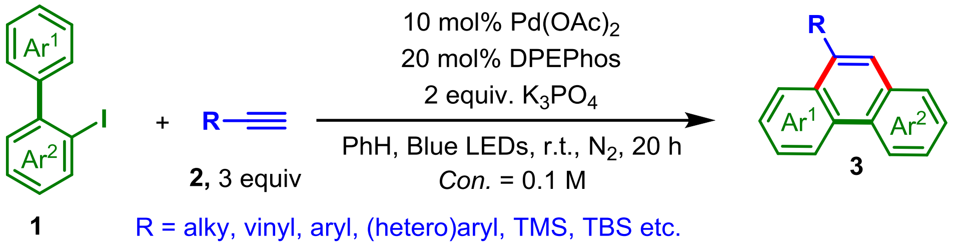

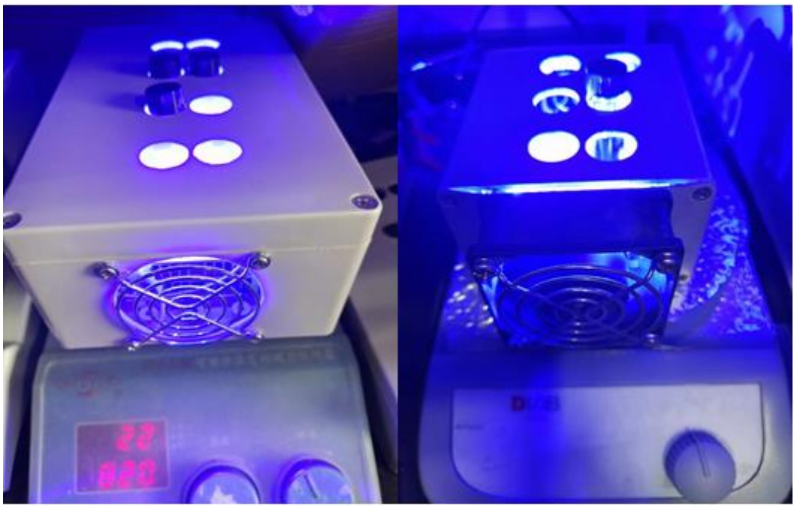

Supplementary Fig 4. Reaction setup

An oven-dried 4.0 mL vial was charged with 2-aryl iodinated arenes **1** (0.2 mmol, 1.0 equiv.), terminal alkyne **2** (0.6 mmol, 3.0 equiv.), $Pd(OAc)_2$ (4.5 mg, 0.02 mmol, 10 mol%), DPEPhos (21.5 mg, 0.04 mmol, 20 mol%) and K_3PO_4 (84.8 mg, 0.4 mmol, 2.0 equiv.). It was directly transferred in a nitrogen-filled glovebox with caps. In the glovebox, 2 mL of degassed benzene (PhH) were added to the vial. The vial was tightly sealed, transferred out of glovebox and stirred at room temperature under the irradiation of blue LEDs lamps for 20 hours. After completion of the reaction, the resulting mixture was diluted with acetone (5 mL), filtered (Celite), and concentrated under a reduced pressure. The residue was purified by column chromatography on silica gel (Petroleum ether to Petroleum ether/ Ethyl acetate = 5:1) to afford **3**.

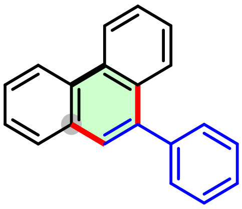

3a

9-Phenylphenanthrene (3a). Following the typical procedure described above, the reaction was carried out by the mixture of **1a** (56.0 mg, 0.2 mmol, 1.0 equiv.), ethynylbenzene (61.2 mg, 0.6 mmol, 3.0 equiv.), $Pd(OAc)_2$ (4.5mg, 0.02 mmol, 10 mol%), DPEPhos (21.5 mg, 0.04 mmol, 20 mol%) and K_3PO_4 (84.8

mg, 0.4 mmol, 2.0 equiv.) in PhH (2.0 mL) at room temperature in nitrogen atmosphere under the irradiation of blue LED lamps for 20 hours. Column chromatography on silica gel (eluent: Petroleum ether) afforded the title product in 82% isolated yield (41.7 mg) as a white solid; R_f = 0.7 (Petroleum ether). **^{1}H NMR (400 MHz, $CDCl_3$)** δ 8.80 (d, *J* = 8.4 Hz, 1H), 8.75 (d, *J* = 8.0 Hz, 1H), 7.93 (dd, *J* = 12.8, 8.0 Hz, 2H), 7.71 – 7.67 (m, 3H), 7.64 (t, *J* = 7.6 Hz, 1H), 7.59 – 7.52 (m, 5H), 7.50 – 7.46 (m, 1H). **^{13}C NMR (101 MHz, $CDCl_3$)** δ 140.8, 138.7, 131.5, 131.1, 130.6, 130.0, 129.9, 128.6, 128.3, 127.5, 127.3, 126.9, 126.8, 126.6, 126.5, 126.4, 122.9, 122.5. The spectroscopic data match the reported literature[18].

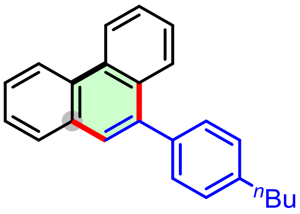

3b

9-(4-Butylphenyl)phenanthrene (3b). Following the typical procedure described above, the reaction was carried out by the mixture of **1a** (56.0 mg, 0.2 mmol, 1.0 equiv), 1-butyl-4-ethynylbenzene (94.9 mg, 0.6 mmol, 3.0 equiv), $Pd(OAc)_2$ (4.5mg, 0.02 mmol, 10 mol%), DPEPhos (21.5 mg, 0.04 mmol, 20 mol%) and *t*-BuOLi (32.0 mg, 0.4 mmol, 2.0 equiv) in PhH (2.0 mL) at room temperature in nitrogen atmosphere under the irradiation of blue LED lamps for 20 hours. Column chromatography on silica gel (eluent: Petroleum ether) afforded the title product in 64% isolated yield (39.9 mg) as a white solid; R_f = 0.6 (Petroleum ether). **^{1}H NMR (400 MHz, $CDCl_3$)** δ 8.79 (d, *J* = 8.0 Hz, 1H), 8.73 (d, *J* = 8.0 Hz, 1H), 7.97 (dd, *J* = 8.0, 1.2 Hz, 1H), 7.90 (dd, *J* = 7.8, 1.6 Hz, 1H), 7.70 – 7.60 (m, 4H), 7.55 (ddd, *J* = 8.4, 6.8, 1.2 Hz, 1H), 7.49 – 7.46 (m, 2H), 7.35 – 7.33 (m, 2H), 2.74 (t, *J* = 8.0 Hz, 2H), 1.76 – 1.68 (m, 2H), 1.51 – 1.42 (m, 2H), 1.00 (t, *J* = 7.2 Hz, 3H). **^{13}C NMR (101 MHz, $CDCl_3$)** δ 142.1, 138.8, 138.0, 131.7, 131.3, 130.6, 129.93, 129.89, 128.6, 128.4, 127.5, 127.0, 126.8, 126.5, 126.44, 126.39, 122.9, 122.5, 35.5, 33.7, 22.5, 14.1. The spectroscopic data match the reported literature[18].

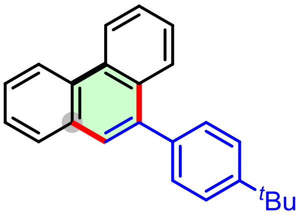

3c

9-(4-(*tert*-Butyl)phenyl)phenanthrene (3c). Following the typical procedure described above, the reaction was carried out by the mixture of **1a** (56.0 mg, 0.2 mmol, 1.0 equiv), 1-(*tert*-butyl)-4-ethynylbenzene (94.9 mg, 0.6 mmol, 3.0 equiv), $Pd(OAc)_2$ (4.5 mg, 0.02 mmol, 10 mol%), DPEPhos

(21.5 mg, 0.04 mmol, 20 mol%) and K_3PO_4 (84.8 mg, 0.4 mmol, 2.0 equiv) in PhH (2.0 mL) at room temperature in nitrogen atmosphere under the irradiation of blue LED lamps for 20 hours. Column chromatography on silica gel (eluent: Petroleum ether) afforded the title product in 72% isolated yield (44.5 mg) as a white solid; R_f = 0.6 (Petroleum ether). **^{1}H NMR (400 MHz, $CDCl_3$)** δ 8.81 (d, J = 8.4 Hz, 1H), 8.75 (d, J = 8.0 Hz, 1H), 8.03 (d, J = 8.0 Hz, 1H), 7.92 (d, J = 7.6 Hz, 1H), 7.75 – 7.66 (m, 3H), 7.64 (t, J = 7.6 Hz, 1H), 7.59 – 7.52 (m, 5H), 1.47 (s, 9H). **^{13}C NMR (101 MHz, $CDCl_3$)** δ 150.2, 138.7, 137.7, 131.6, 131.2, 130.6, 129.9, 129.8, 129.7, 128.6, 127.8, 127.5, 127.0, 126.8, 126.44, 126.41, 126.36, 125.2, 122.8, 122.5, 34.6, 31.5. The spectroscopic data match the reported literature[19].

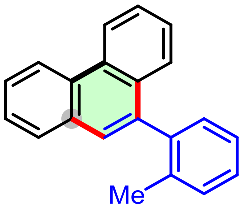

3d

9-(*o*-Tolyl)phenanthrene (3d). Following the typical procedure described above, the reaction was carried out by the mixture of **1a** (56.0 mg, 0.2 mmol, 1.0 equiv), 1-ethynyl-2-methylbenzene (69.7 mg, 0.6 mmol, 3.0 equiv), $Pd(OAc)_2$ (4.5 mg, 0.02 mmol, 10 mol%), DPEPhos (21.5 mg, 0.04 mmol, 20 mol%) and K_3PO_4 (84.8 mg, 0.4 mmol, 2.0 equiv) in PhH (2.0 mL) at room temperature in nitrogen atmosphere under the irradiation of blue LED lamps for 20 hours. Column chromatography on silica gel (Petroleum ether) afforded the title product in 71% isolated yield (38.2 mg) as a white solid; R_f = 0.7 (Petroleum ether). **^{1}H NMR (400 MHz, $CDCl_3$)** δ 8.80 (d, J = 8.4 Hz, 1H), 8.77 (d, J = 8.0 Hz, 1H), 7.91 (d, J = 8.8 Hz, 1H), 7.73 – 7.63 (m, 4H), 7.55 – 7.50 (m, 2H), 7.44 – 7.35 (m, 4H), 2.10 (s, 3H). **^{13}C NMR (101 MHz, $CDCl_3$)** δ 140.2, 138.4, 137.0, 131.6, 131.3, 130.33, 130.29, 130.0, 129.9, 128.6, 127.7, 127.1, 126.82, 126.76, 126.6, 126.5, 126.4, 125.7, 122.8, 122.5, 20.1. The spectroscopic data match the reported literature[18].

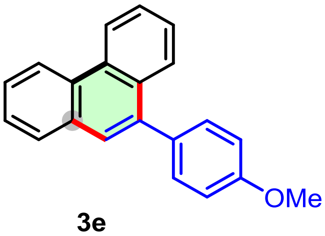

3e

9-(4-Methoxyphenyl)phenanthrene (3e). Following the typical procedure described above, the reaction was carried out by the mixture of **1a** (56.0 mg, 0.2 mmol, 1.0 equiv), 1-ethynyl-4-methoxybenzene (79.3 mg, 0.6 mmol, 3.0 equiv), $Pd(OAc)_2$ (4.5 mg, 0.02 mmol, 10 mol%), DPEPhos (21.5 mg, 0.04 mmol, 20 mol%) and K_3PO_4 (84.4 mg, 0.4 mmol, 2.0 equiv) in PhH (2.0 mL) at room temperature in nitrogen atmosphere under the irradiation of blue LED lamps for 20 hours. Column chromatography on silica gel

(eluent: Petroleum ether) afforded the title product in 80% isolated yield (45.3 mg) as a white solid. For the reaction starting from substrate **1a-Br**, following the typical procedure described above, the reaction was carried out by the mixture of **1a-Br** (46.6 mg, 0.2 mmol, 1.0 equiv), 1-ethynyl-4-methoxybenzene (79.3 mg, 0.6 mmol, 3.0 equiv), (dppf)$PdCl_2$ (14.6 mg, 0.02 mmol, 10 mol%), DPEPhos (21.5 mg, 0.04 mmol, 20 mol%) and K_3PO_4 (84.4 mg, 0.4 mmol, 2.0 equiv) in PhH (2.0 mL) at room temperature in nitrogen atmosphere under the irradiation of blue LED lamps for 20 hours. Column chromatography on silica gel (eluent: Petroleum ether) afforded the title product in 74% isolated yield (42.1 mg) as a white solid; R_f = 0.5 (Petroleum ether). **^{1}H NMR (400 MHz, $CDCl_3$)** δ 8.80 (d, J = 8.4 Hz, 1H), 8.75 (d, J = 8.0 Hz, 1H), 8.00 (d, J = 8.4 Hz, 1H), 7.92 (d, J = 7.6 Hz, 1H), 7.71 – 7.62 (m, 4H), 7.58 (t, J = 7.6 Hz, 1H), 7.51 (d, J = 8.0 Hz, 2H), 7.09 (d, J = 8.0 Hz, 2H), 3.93 (s, 3H). **^{13}C NMR (101 MHz, $CDCl_3$)** δ 159.0, 138.4, 133.1, 131.6, 131.4, 131.1, 130.6, 129.8, 128.5, 127.4, 126.9, 126.8, 126.42, 126.36, 122.9, 122.5, 113.7, 55.4. The spectroscopic data match the reported literature[18].

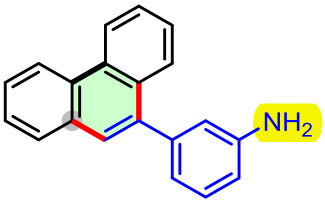

3f

3-(Phenanthren-9-yl)aniline (3f). Following the typical procedure described above, the reaction was carried out by the mixture of **1a** (56mg, 0.2 mmol, 1.0 equiv), 3-ethynylaniline (70.3 mg, 0.6 mmol, 3.0 equiv), $Pd(OAc)_2$ (4.5 mg, 0.02 mmol, 10 mol%), DPEPhos (21.5 mg, 0.04 mmol, 20 mol%) and *t*-BuOLi (32.0 mg, 0.4 mmol, 2.0 equiv) in PhH (2.0 mL) at room temperature in nitrogen atmosphere under the irradiation of blue LED lamps for 20 hours. Column chromatography on silica gel (eluent: Petroleum ether/ Ethyl acetate = 10:1) afforded the title product in 48% isolated yield (25.9 mg) as a yellow oil; R_f = 0.5 (Petroleum ether/ Ethyl acetate = 10:1). **^{1}H NMR (400 MHz, $CDCl_3$)** δ 8.78 (d, J = 8.4 Hz, 1H), 8.73 (d, J = 8.0 Hz, 1H), 8.02 (d, J = 8.4 Hz, 1H), 7.90 (d, J = 7.6 Hz, 1H), 7.70 – 7.61 (m, 4H), 7.55 (t, J = 7.6 Hz, 1H), 7.31 (t, J = 7.6 Hz, 1H), 6.96 (d, J = 7.6 Hz, 1H), 6.88 (s, 1H), 6.79 (d, J = 8.0 Hz, 1H), 3.65 (s, 2H). **^{13}C NMR (101 MHz, $CDCl_3$)** δ 146.3, 141.9, 138.9, 131.5, 131.1, 130.5, 129.9, 129.2, 128.6, 127.09, 127.08, 126.8, 126.5, 126.40, 126.36, 122.8, 122.5, 120.5, 116.8, 114.1. **HRMS (ESI)** calcd for $C_{20}H_{16}N$ $[M+H]^+$: 270.1277, found 270.1272.

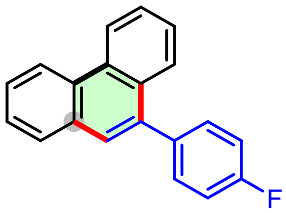

3g

9-(4-Fluorophenyl)phenanthrene (3g). Following the typical procedure described above, the reaction was carried out by the mixture of **1a** (56.0 mg, 0.2 mmol, 1.0 equiv), 1-ethynyl-4-fluorobenzene (72.1 mg, 0.6 mmol, 3.0 equiv), $Pd(OAc)_2$ (4.5 mg, 0.02 mmol, 10 mol%), DPEPhos (21.5 mg, 0.04 mmol, 20 mol%) and *t*-BuOLi (32.0 mg, 0.4 mmol, 2.0 equiv) in PhH (2.0 mL) at room temperature in nitrogen atmosphere under the irradiation of blue LED lamps for 20 hours. Column chromatography on silica gel (eluent: Petroleum ether) afforded the title product in 64% isolated yield (34.6 mg) as a white solid; R_f = 0.6 (Petroleum ether). **^{1}H NMR (400 MHz, $CDCl_3$)** δ 8.80 (d, *J* = 8.4 Hz, 1H), 8.74 (d, *J* = 8.0 Hz, 1H), 7.90 (td, *J* = 7.6, 1.6 Hz, 2H), 7.72 – 7.62 (m, 4H), 7.59 – 7.50 (m, 3H), 7.25 – 7.21 (m, 2H). **^{13}C NMR (101 MHz, $CDCl_3$)** δ 162.3 (d, $^1J_{C-F}$ = 247.1 Hz), 136.6 (d, $^4J_{C-F}$ = 3.4 Hz), 131.6, 131.5, 131.4, 131.0, 130.6, 129.9, 128.6, 127.6, 126.9, 126.7, 126.6, 126.6, 126.5, 122.7 (d, $^3J_{C-F}$ = 42.5 Hz), 115.2 (d, $^2J_{C-F}$ = 21.4 Hz). **^{19}F NMR (282 MHz, $CDCl_3$)** δ -115.2. The spectroscopic data match the reported literature[18].

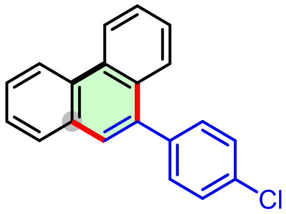

3h

9-(4-Chlorophenyl)phenanthrene (3h). Following the typical procedure described above, the reaction was carried out by the mixture of **1a** (56.0 mg, 0.2 mmol, 1.0 equiv), 1-chloro-4-ethynylbenzene (81.9 mg, 0.6 mmol, 3.0 equiv), $Pd(OAc)_2$ (4.5 mg, 0.02 mmol, 10 mol%), DPEPhos (21.5 mg, 0.04 mmol, 20 mol%) and *t*-BuOLi (32.0 mg, 0.4 mmol, 2.0 equiv) in PhH (2.0 mL) at room temperature in nitrogen atmosphere under the irradiation of blue LED lamps for 20 hours. Column chromatography on silica gel (eluent: Petroleum ether) afforded the title product in 57% isolated yield (33.0 mg) as a white solid; R_f = 0.6 (Petroleum ether). **^{1}H NMR (400 MHz, $CDCl_3$)** δ 8.79 (d, *J* = 8.4 Hz, 1H), 8.73 (d, *J* = 8.0 Hz, 1H), 7.88 (dd, *J* = 13.2, 8.0Z Hz, 2H), 7.71 – 7.61 (m, 4H), 7.58 – 7.54 (m, 1H), 7.52 – 7.47 (m, 4H). **^{13}C NMR (101 MHz, $CDCl_3$)** δ 139.2, 137.4, 133.4, 131.3, 130.8, 130.6, 130.0, 128.7, 128.5, 127.6, 126.9, 126.8, 126.62, 126.58, 123.0, 122.5. The spectroscopic data match the reported literature[18].

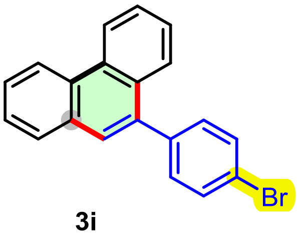

3i

9-(4-Bromophenyl)phenanthrene (3i). Following the typical procedure described above, the reaction was carried out by the mixture of **1a** (56.0 mg, 0.2 mmol, 1.0 equiv), 1-bromo-4-ethynylbenzene (108.6 mg, 0.6 mmol, 3.0 equiv), (dppf)$PdCl_2$ (14.6 mg, 0.02 mmol, 10 mol%), DPEPhos (21.5 mg, 0.04 mmol, 20 mol%) and K_3PO_4 (84.8 mg, 0.4 mmol, 2.0 equiv) in PhH (2.0 mL) at room temperature in nitrogen atmosphere under the irradiation of blue LED lamps for 20 hours. Column chromatography on silica gel (eluent: Petroleum ether) afforded the title product in 49% isolated yield (32.6 mg) as a white solid; R_f = 0.6 (Petroleum ether). **^{1}H NMR (300 MHz, $CDCl_3$)** δ 8.79 (d, *J* = 8.4 Hz, 1H), 8.73 (d, *J* = 8.1 Hz, 1H), 7.88 (t, *J* = 9.3 Hz, 2H), 7.72 – 7.60 (m, 6H), 7.56 (dt, *J* = 8.1, 4.2 Hz, 1H), 7.43 (d, *J* = 8.4zz Hz, 2H). **^{13}C NMR (101 MHz, $CDCl_3$)** δ 139.7, 137.4, 131.7, 131.5, 131.3, 130.7, 130.6, 130.0, 128.7, 127.6, 126.9, 126.8, 126.63, 126.60, 126.56, 123.0, 122.5, 121.5. The spectroscopic data match the reported literature[18].

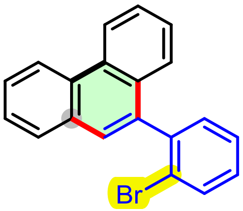

3j

9-(2-Bromophenyl)phenanthrene (3j). Following the typical procedure described above, the reaction was carried out by the mixture of **1a** (56.0 mg, 0.2 mmol, 1.0 equiv), 1-bromo-2-ethynylbenzene (108.6 mg, 0.6 mmol, 3.0 equiv), $Pd(OAc)_2$ (4.5mg, 0.02 mmol, 10 mol%), DPEPhos (21.5 mg, 0.04 mmol, 20 mol%) and K_3PO_4 (84.8 mg, 0.4 mmol, 2.0 equiv) in PhH (2.0 mL) at room temperature in nitrogen atmosphere under the irradiation of blue LED lamps for 20 hours. Column chromatography on silica gel (eluent: Petroleum ether) afforded the title product in 47% isolated yield (31.3 mg) as a white solid; R_f = 0.5 (Petroleum ether). **^{1}H NMR (400 MHz, $CDCl_3$)** δ 8.80 (d, *J* = 8.4 Hz, 1H), 8.77 (d, *J* = 8.0 Hz, 1H), 7.93 (d, *J* = 7.6 Hz, 1H), 7.79 (d, *J* = 8.0 Hz, 1H), 7.74 – 7.64 (m, 4H), 7.58 – 7.53 (m, 2H), 7.50 – 7.44 (m, 2H), 7.36 (ddd, *J* = 9.2, 6.4, 2.8 Hz, 1H). **^{13}C NMR (101 MHz, $CDCl_3$)** δ 141.3, 137.8, 132.7, 132.0, 131.3, 130.7, 130.3, 130.2, 129.2, 128.8, 127.7, 127.3, 126.84, 126.82, 126.7, 126.6, 126.5, 124.5, 122.9, 122.6. The spectroscopic data match the reported literature[20].

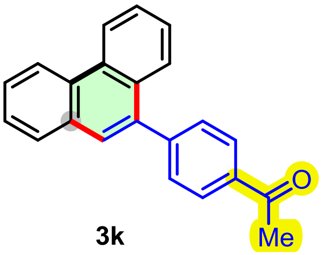

3k

1-(4-(Phenanthren-9-yl)phenyl)ethan-1-one (3k). Following the typical procedure described above, the reaction was carried out by the mixture of **1a** (56.0 mg, 0.2 mmol, 1.0 equiv), 1-(4-ethynylphenyl)ethan-1-one (86.8 mg, 0.6 mmol, 3.0 equiv), $Pd(OAc)_2$ (4.5mg, 0.02 mmol, 10 mol%), DPEPhos (21.5 mg, 0.04 mmol, 20 mol%) and K_3PO_4 (84.8 mg, 0.4 mmol, 2.0 equiv) in PhH (2.0 mL) at room temperature in nitrogen atmosphere under the irradiation of blue LED lamps for 20 hours. Column chromatography on silica gel (eluent: Petroleum ether/ Ethyl acetate = 100:1) afforded the title product in 62% isolated yield (36.8 mg) as a white solid; R_f = 0.5 (Petroleum ether/ Ethyl acetate = 50:1). **^{1}H NMR (600 MHz, $CDCl_3$)** δ 8.80 (d, J = 8.4 Hz, 1H), 8.74 (d, J = 8.4 Hz, 1H), 8.15 – 8.09 (m, 2H), 7.91 (dd, J = 7.8, 1.2 Hz, 1H), 7.85 (dd, J = 8.4, 1.2 Hz, 1H), 7.72 – 7.68 (m, 3H), 7.68 – 7.62 (m, 3H), 7.55 (ddd, J = 8.4, 6.9, 1.2 Hz, 1H), 2.71 (s, 3H). **^{13}C NMR (151 MHz, $CDCl_3$)** δ 197.9, 145.9, 137.6, 136.1, 131.3, 130.7, 130.5, 130.3, 130.1, 128.8, 128.4, 127.7, 127.01, 126.98, 126.7, 126.5, 123.0, 122.6, 26.7. The spectroscopic data match the reported literature[18].

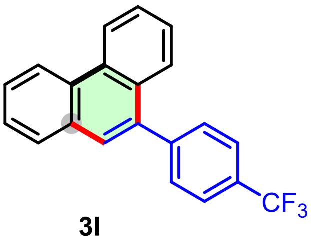

3l

9-(4-(Trifluoromethyl)phenyl)phenanthrene (3l). Following the typical procedure described above, the reaction was carried out by the mixture of **1a** (56.0 mg, 0.2 mmol, 1.0 equiv), 1-ethynyl-4-(trifluoromethyl)benzene (102.1 mg, 0.6 mmol, 3.0 equiv), $Pd(OAc)_2$ (4.5mg, 0.02 mmol, 10 mol%), DPEPhos (21.5. mg, 0.04 mmol, 20 mol%) and K_3PO_4 (84.8 mg, 0.4 mmol, 2.0 equiv) in PhH (2.0 mL) at room temperature in nitrogen atmosphere under the irradiation of blue LED lamps for 20 hours. Column chromatography on silica gel (eluent: Petroleum ether) afforded the title product in 65% isolated yield (41.7 mg) as a white solid; R_f = 0.5 (Petroleum ether). **^{1}H NMR (400 MHz, $CDCl_3$)** δ 8.81 (d, J = 8.4 Hz, 1H), 8.75 (d, J = 8.0 Hz, 1H), 7.92 (dd, J = 7.6, 1.6 Hz, 1H), 7.84 (dd, J = 8.0, 1.6 Hz, 1H), 7.80 (d, J = 8.0 Hz, 2H), 7.74 – 7.64 (m, 6H), 7.59 – 7.55 (m, 1H). **^{13}C NMR (101 MHz, $CDCl_3$)** δ144.5, 137.3, 131.2, 130.6, 130.5, 130.4, 130.1, 129.6(q, $^2J_{C-F}$ = 32.6 Hz), 128.8, 127.8, 127.6, 127.02, 127.01, 126.73, 126.72, 126.4, 125.2(q, $^3J_{C-F}$ = 3.8 Hz), 124.4 (q, $^1J_{C-F}$ = 273.1 Hz), 123.0, 122.6. **^{19}F NMR (282 MHz, CDCl3)** δ -62.3. The spectroscopic data match the reported literature[18].

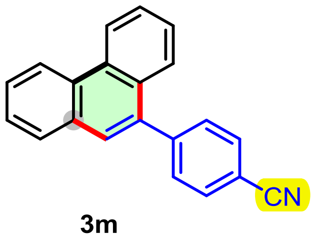

3m

4-(Phenanthren-9-yl)benzonitrile (3m). Following the typical procedure described above, the reaction was carried out by the mixture of **1a** (56.0 mg, 0.2 mmol, 1.0 equiv), 4-ethynylbenzonitrile (76.3 mg, 0.6 mmol, 3.0 equiv), $Pd(OAc)_2$ (4.5mg, 0.02 mmol, 10 mol%), DPEPhos (21.5 mg, 0.04 mmol, 20 mol%) and K_3PO_4 (84.8 mg, 0.4 mmol, 2.0 equiv) in PhH (2.0 mL) at room temperature in nitrogen atmosphere under the irradiation of blue LED lamps for 20 hours. Column chromatography on silica gel (eluent: Petroleum ether/Ethyl acetate = 50:1) afforded the title product in 72% isolated yield (40.5 mg) as a white solid; R_f = 0.5 (Petroleum ether/ Ethyl acetate = 20:1). **^{1}H NMR (400 MHz, $CDCl_3$)** δ 8.80 (d, J = 8.4 Hz, 1H), 8.74 (d, J = 8.4 Hz, 1H), 7.91 (dd, J = 7.6, 1.6 Hz, 1H), 7.83 – 7.77 (m, 3H), 7.74 – 7.63 (m, 6H), 7.57 (ddd, J = 8.4, 7.2, 1.2 Hz, 1H). **^{13}C NMR (101 MHz, $CDCl_3$)** δ 145.7, 136.8, 132.2, 131.1, 130.8, 130.7, 130.23, 130.16, 128.8, 127.9, 127.2, 127.1, 126.87, 126.86, 126.2, 123.1, 122.6, 118.9, 111.3. The spectroscopic data match the reported literature[16].

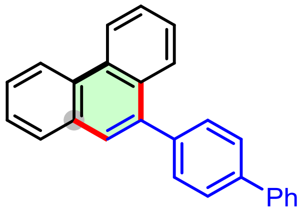

3n

9-([1,1'-Biphenyl]-4-yl)phenanthrene (3n). Following the typical procedure described above, the reaction was carried out by the mixture of **1a** (56mg, 0.2 mmol, 1.0 equiv), 4-ethynyl-1,1'-biphenyl (106.9 mg, 0.6 mmol, 3.0 equiv), $Pd(OAc)_2$ (4.5 mg, 0.02 mmol, 10 mol%), DPEPhos (21.5 mg, 0.04 mmol, 20 mol%) and K_3PO_4 (84.8 mg, 0.4 mmol, 2.0 equiv) in PhH (2.0 mL) at room temperature in nitrogen atmosphere under the irradiation of blue LED lamps for 20 hours. Column chromatography on silica gel (eluent: Petroleum ether) afforded the title product in 63% isolated yield (41.6 mg) as a white solid; R_f = 0.5 (Petroleum ether). **^{1}H NMR (400 MHz, $CDCl_3$)** δ 8.81 (d, J = 8.4 Hz, 1H), 8.75 (d, J = 8.0 Hz, 1H), 8.02 (d, J = 8.0 Hz, 1H), 7.92 (d, J = 7.6 Hz, 1H), 7.77 – 7.67 (m, 7H), 7.65 – 7.62 (m, 3H), 7.60 – 7.56 (m, 1H), 7.51 (t, J = 7.6 Hz, 2H), 7.43 – 7.38 (m, 1H). **^{13}C NMR (101 MHz, $CDCl_3$)** δ140.8, 140.2, 139.7, 138.3, 131.5, 131.0, 130.6, 130.5, 129.9, 128.9, 128.7, 127.5, 127.4, 127.1, 127.0, 126.89, 126.86, 126.6, 126.54, 126.49, 122.9, 122.5. The spectroscopic data match the reported literature[21].

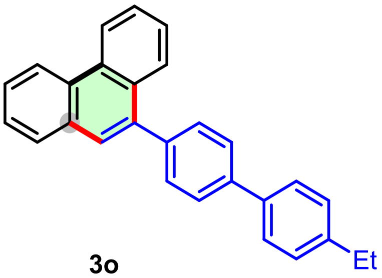

3o

9-(4'-Ethyl-[1,1'-biphenyl]-4-yl)phenanthrene (3o). Following the typical procedure described above, the reaction was carried out by the mixture of **1a** (56.0 mg, 0.2 mmol, 1.0 equiv), 4-ethyl-4'-ethynyl-1,1'-biphenyl (123.8 mg, 0.6 mmol, 3.0 equiv), $Pd(OAc)_2$ (4.5 mg, 0.02 mmol, 10 mol%), DPEPhos (21.5 mg, 0.04 mmol, 20 mol%) and K_3PO_4 (32.0 mg, 0.4 mmol, 2.0 equiv) in PhH (2.0 mL) at room temperature in nitrogen atmosphere under the irradiation of blue LED lamps for 20 hours. Column chromatography on silica gel (eluent: Petroleum ether) afforded the title product in 72% isolated yield (51.6 mg) as a white solid; M.p. 145.9 - 146.9 ^{o}C. R_f = 0.6 (Petroleum ether). **^{1}H NMR (400 MHz, CDCl$_3$)** δ 8.83 (d, J = 8.4 Hz, 1H), 8.78 (d, J = 8.0 Hz, 1H), 8.07 (d, J = 7.6 Hz, 1H), 7.95 (d, J = 8.4 Hz, 1H), 7.79 – 7.77 (m, 3H), 7.75 – 7.65 (m, 7H), 7.63 – 7.59 (m, 1H), 7.38 (d, J = 8.0 Hz, 2H), 2.78 (q, J = 7.6 Hz, 2H), 1.36 (t, J = 7.6 Hz, 3H). **^{13}C NMR (101 MHz, CDCl$_3$)** δ 143.6, 140.2, 139.5, 138.5, 138.2, 131.6, 131.2, 130.7, 130.5, 130.0, 128.7, 128.5, 127.6, 127.1, 127.0, 126.9, 126.7, 126.61, 126.55, 123.0, 122.6, 28.6, 15.7. **HRMS (EI)** calcd for $C_{28}H_{22}$ $[M]^+$: 358.1716, found 358.1713.

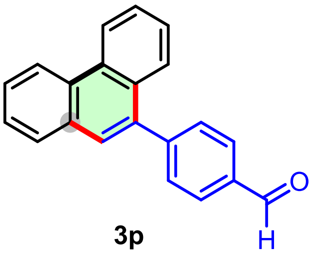

3p

4-(Phenanthren-9-yl)benzaldehyde (**3p**). Following the typical procedure described above, the reaction was carried out by the mixture of **1a** (56.0 mg, 0.2 mmol, 1.0 equiv), 4-ethynylbenzaldehyde (78.2 mg, 0.6 mmol, 3.0 equiv), $Pd(OAc)_2$ (4.5 mg, 0.02 mmol, 10 mol%), DPEPhos (21.5 mg, 0.04 mmol, 20 mol%) and K_3PO_4 (84.8 mg, 0.4 mmol, 2.0 equiv) in PhH (2.0 mL) at room temperature in nitrogen atmosphere under the irradiation of blue LED lamps for 20 hours. Column chromatography on silica gel (eluent: Petroleum ether : Ethyl acetate = 30:1) afforded the title product in 65% isolated yield (36.8 mg) as a white solid; R_f = 0.5 (Petroleum ether : Ethyl acetate = 10:1). **^{1}H NMR (600 MHz, CDCl$_3$)** δ 10.14 (s, 1H), 8.80 (d, J = 8.4 Hz, 1H), 8.74 (d, J = 8.4 Hz, 1H), 8.05 – 8.03 (m, 2H), 7.92 (dd, J = 7.8, 1.5 Hz, 1H), 7.85 (dd, J = 8.4, 1.2 Hz, 1H), 7.74 – 7.69 (m, 5H), 7.65 (ddd, J = 8.1, 6.9, 1.2 Hz, 1H), 7.56 (ddd, J = 8.1, 6.9, 1.2 Hz, 1H). **^{13}C NMR (151 MHz, CDCl$_3$)** δ 192.0, 147.3, 137.4, 135.4, 131.2, 130.8, 130.7, 130.4, 130.2, 129.8, 128.8, 127.8, 127.09, 127.06, 126.8, 126.4, 123.1, 122.6. The spectroscopic data match the reported literature[22].

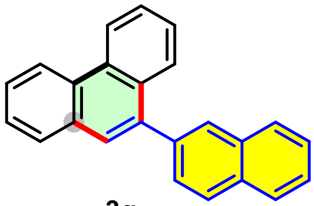

3q

9-(Naphthalen-2-yl)phenanthrene (3q). Following the typical procedure described above, the reaction was carried out by the mixture of **1a** (56.0 mg, 0.2 mmol, 1.0 equiv), 2-ethynylnaphthalene (91.3 mg, 0.6 mmol, 3.0 equiv), $Pd(OAc)_2$ (4.5 mg, 0.02 mmol, 10 mol%), DPEPhos (21.5 mg, 0.04 mmol, 20 mol%) and K_3PO_4 (84.8 mg, 0.4 mmol, 2.0 equiv) in PhH (2.0 mL) at room temperature in nitrogen atmosphere under the irradiation of blue LED lamps for 20 hours. Column chromatography on silica gel (eluent: Petroleum ether) afforded the title product in 75% isolated yield (45.7 mg) as a white solid; R_f = 0.6 (Petroleum ether). **^{1}H NMR (400 MHz, $CDCl_3$)** δ 8.82 (d, *J* = 8.4 Hz, 1H), 8.77 (d, *J* = 8.0 Hz, 1H), 8.05 (s, 1H), 8.01 – 7.93 (m, 5H), 7.81 (s, 1H), 7.73 – 7.64 (m, 4H), 7.60 – 7.53 (m, 3H). **^{13}C NMR (101 MHz, $CDCl_3$)** δ 138.7, 138.3, 133.4, 132.6, 131.5, 131.2, 130.6, 130.0, 128.7, 128.4, 128.0, 127.84, 127.76, 127.6, 127.0, 126.9, 126.6, 126.54, 126.49, 126.3, 126.1, 122.9, 122.5. The spectroscopic data match the reported literature[18].

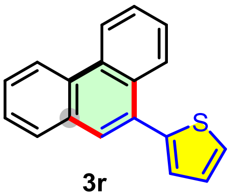

3r

2-(Phenanthren-9-yl)thiophene (3r). Following the typical procedure described above, the reaction was carried out by the mixture of **1a** (56.0 mg, 0.2 mmol, 1.0 equiv), 2-ethynylthiophene (64.9 mg, 0.6 mmol, 3.0 equiv), $Pd(OAc)_2$ (4.5 mg, 0.02 mmol, 10 mol%), DPEPhos (21.5 mg, 0.04 mmol, 20 mol%) and *t*-BuOLi (32.0 mg, 0.4 mmol, 2.0 equiv) in PhH (2.0 mL) at room temperature in nitrogen atmosphere under the irradiation of blue LED lamps for 20 hours. Column chromatography on silica gel (eluent: Petroleum ether) afforded the title product in 69% isolated yield (36.1 mg) as a yellow oil; R_f = 0.4 (Petroleum ether : Ethyl acetate = 100:1). **^{1}H NMR (400 MHz, $CDCl_3$)** δ 8.78 (d, *J* = 8.4 Hz, 1H), 8.72 (d, *J* = 8.4 Hz, 1H), 8.28 (dd, *J* = 8.4, 1.2 Hz, 1H), 7.91 (dd, *J* = 7.6, 1.2 Hz, 1H), 7.87(s, 1H), 7.73 – 7.67 (m, 2H), 7.65 – 7.60 (m, 2H), 7.47 (dd, *J* = 5.2, 1.2 Hz, 1H), 7.32 (dd, *J* = 3.6, 1.2 Hz, 1H), 7.23 (dd, *J* = 5.2, 3.6 Hz, 1H). **^{13}C NMR (101 MHz, $CDCl_3$)** δ 141.7, 131.2, 131.1, 131.0, 130.6, 130.1, 129.1, 128.7, 127.6, 127.2, 127.0, 126.9, 126.8, 126.7, 126.6, 125.5, 122.9, 122.5. The spectroscopic data match the reported literature[18].

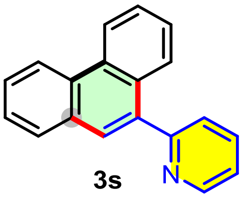

3s

2-(Phenanthren-9-yl)pyridine (3s). Following the typical procedure described above, the reaction was carried out by the mixture of **1a** (56.0 mg, 0.2 mmol, 1.0 equiv), 2-ethynylpyridine (61.9 mg, 0.6 mmol, 3.0 equiv), $Pd(OAc)_2$ (4.5mg, 0.02 mmol, 10 mol%), DPEPhos (21.5 mg, 0.04 mmol, 20 mol%) and K_3PO_4 (84.8 mg, 0.4 mmol, 2.0 equiv) in PhH (2.0 mL) at room temperature in nitrogen atmosphere under the irradiation of blue LED lamps for 20 hours. Column chromatography on silica gel (eluent: Petroleum ether : Ethyl acetate = 10:1) afforded the title product in 68% isolated yield (34.6 mg) as a brown oil; R_f = 0.6 (Petroleum ether : Ethyl acetate = 5:1). **^{1}H NMR (400 MHz, $CDCl_3$)** δ 8.84 (d, J = 4.4 Hz, 1H), 8.79 (d, J = 8.4 Hz, 1H), 8.73 (d, J = 8.0 Hz, 1H), 8.10 (d, J = 8.0 Hz, 1H), 7.94 (d, J = 7.6 Hz, 1H), 7.93 – 7.83 (m, 2H), 7.72 – 7.56 (m, 5H), 7.37 (ddd, J = 7.6, 4.8, 1.2 Hz, 1H). **^{13}C NMR (101 MHz, $CDCl_3$)** δ 159.2, 149.5, 137.1, 136.5, 131.3, 130.7, 130.4, 130.2, 129.0, 128.4, 127.0, 126.8, 126.7, 126.5, 126.4, 125.1, 122.9, 122.5, 122.1. The spectroscopic data match the reported literature[23].

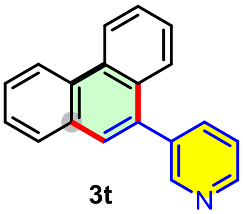

3t

3-(Phenanthren-9-yl)pyridine (3t). Following the typical procedure described above, the reaction was carried out by the mixture of **1a** (56.0 mg, 0.2 mmol, 1.0 equiv), 3-ethynylpyridine (61.9 mg, 0.6 mmol, 3.0 equiv), $Pd(OAc)_2$ (4.5 mg, 0.02 mmol, 10 mol%), DPEPhos (21.5 mg, 0.04 mmol, 20 mol%) and K_3PO_4 (84.4 mg, 0.4 mmol, 2.0 equiv) in PhH (2.0 mL) at room temperature in nitrogen atmosphere under the irradiation of blue LED lamps for 20 hours. Column chromatography on silica gel (eluent: Petroleum ether : Ethyl acetate = 10:1) afforded the title product in 75% isolated yield (38.3 mg) as a brown oil; R_f = 0.5 (Petroleum ether : Ethyl acetate = 5:1). **^{1}H NMR (400 MHz, $CDCl_3$)** δ 8.83 (dd, J = 2.4, 0.8 Hz, 1H), 8.80 (d, J = 8.4 Hz, 1H), 8.75 – 8.72 (m, 2H), 7.93 – 7.81 (m, 3H), 7.73 – 7.62 (m, 4H), 7.57 (ddd, J = 8.0, 7.2, 1.2 Hz, 1H), 7.46 (ddd, J = 7.8, 4.8, 0.8 Hz, 1H). **^{13}C NMR (101 MHz, $CDCl_3$)** δ 150.6, 148.7, 137.4, 136.4, 134.9, 131.2, 130.7, 130.6, 130.2, 128.7, 128.3, 127.1, 127.0, 126.79, 126.76, 126.2, 123.1, 123.1, 122.6. The spectroscopic data match the reported literature[18].

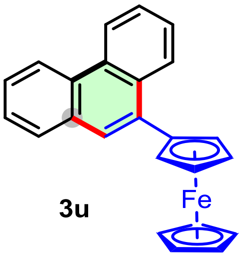

3u

2-(Phenanthren-9-yl)ferrocene (3u). Following the typical procedure described above, the reaction was carried out by the mixture of **1a** (56.0 mg, 0.2 mmol, 1.0 equiv), ethynylferrocene (126.1 mg, 0.6 mmol, 3 equiv), $Pd(OAc)_2$ (4.5 mg, 0.02 mmol, 10 mol%), DPEPhos (21.5 mg, 0.04 mmol, 20 mol%) and K_3PO_4 (84.8 mg, 0.4 mmol, 2.0 equiv) in PhH (2.0 mL) at room temperature in nitrogen atmosphere under the irradiation of blue LED lamps for 20 hours. Column chromatography on silica gel (eluent: Petroleum ether : Ethyl acetate = 70:1) afforded the title product in 67% isolated yield (48.6 mg) as a orange oil; R_f = 0.6 (Petroleum ether : Ethyl acetate = 50:1). **^{1}H NMR (600 MHz, $CDCl_3$)** δ 8.78 (d, J = 8.1 Hz, 1H), 8.73 – 8.68 (m, 2H), 8.18 (s, 1H), 7.95 (dd, J = 7.7, 1.6 Hz, 1H), 7.73 – 7.62 (m, 4H), 4.73 (t, J = 1.9 Hz, 2H), 4.45 (t, J = 1.8 Hz, 2H), 4.27 (s, 4H). **^{13}C NMR (151 MHz, $CDCl_3$)** δ 134.3, 131.6, 131.3, 130.4, 129.6, 128.4, 128.2, 126.8, 126.8, 126.2, 126.2, 126.0, 122.9, 122.5, 87.3, 70.5, 69.6, 68.1. **HRMS (ESI)** calcd for $C_{24}H_{19}Fe$ $[M+H]^+$: 363.0825, found 363.0823.

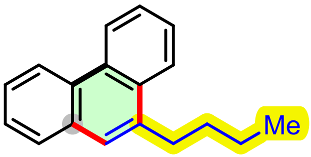

3v

9-Butylphenanthrene (3v). Following the typical procedure described above, the reaction was carried out by the mixture of **1a** (56.0 mg, 0.2 mmol, 1.0 equiv), hex-1-yne (49.3 mg, 0.6 mmol, 3.0 equiv), $Pd(OAc)_2$ (4.5 mg, 0.02 mmol, 10 mol%), DPEPhos (21.5 mg, 0.04 mmol, 20 mol%) and K_3PO_4 (84.8 mg, 0.4 mmol, 2.0 equiv) in PhH (2.0 mL) at room temperature in nitrogen atmosphere under the irradiation of blue LED lamps for 20 hours. Column chromatography on silica gel (eluent: Petroleum ether) afforded the title product in 48% isolated yield (22.5 mg) as a yellow solid. R_f = 0.7 (Petroleum ether). **^{1}H NMR (600 MHz, $CDCl_3$)** δ 8.75 (dd, J = 7.2, 2.0 Hz, 1H), 8.66 (d, J = 8.0 Hz, 1H), 8.12 (dd, J = 7.2, 2.4 Hz, 1H), 7.83 (dd, J = 7.6, 1.6 Hz, 1H), 7.65 (tt, J = 7.2, 5.2 Hz, 2H), 7.62 – 7.56 (m, 3H), 3.14 – 3.11 (m, 2H), 1.84 – 1.79 (m, 2H), 1.55 – 1.49 (m, 2H), 1.01 (t, J = 7.2 Hz, 3H). **^{13}C NMR (151 MHz, $CDCl_3$)** δ 137.0, 132.0, 131.3, 130.7, 129.6, 128.0, 126.5, 126.4, 126.0, 125.9, 125.8, 124.5, 123.2, 122.4, 33.2, 32.4, 22.9, 14.0. The spectroscopic data match the reported literature[24].

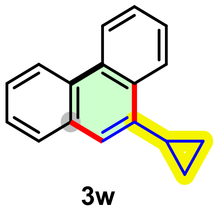

3w

9-Cyclopropylphenanthrene (3w). 1a-Br was used. Following the typical procedure described above, the reaction was carried out by the mixture of **1a-Br** (46.6 mg, 0.2 mmol, 1.0 equiv), ethynylcyclopropane (39.7 mg, 0.6 mmol, 3.0 equiv), (dppf)$PdCl_2$ (14.6 mg, 0.02 mmol, 10 mol%), DPEPhos (21.5 mg, 0.04 mmol, 20 mol%) and K_3PO_4 (84.8 mg, 0.4 mmol, 2.0 equiv) in PhH (2.0 mL) at room temperature in nitrogen atmosphere under the irradiation of blue LED lamps for 20 hours. Column chromatography on silica gel (eluent: Petroleum ether) afforded the title product in 60% isolated yield (26.2 mg) as a colorless oil; R_f = 0.7 (Petroleum ether). **^{1}H NMR (400 MHz, $CDCl_3$)** δ 8.73 (dd, *J* = 6.4, 2.4 Hz, 1H), 8.66 (d, *J* = 8.0 Hz, 1H), 8.53 – 8.51 (m, 1H), 7.83 (d, *J* = 7.6 Hz, 1H), 7.70 – 7.67 (m, 2H), 7.62 – 7.55 (m, 3H), 2.36 (p, *J* = 8.0 Hz, 1H), 1.17 – 1.06 (m, 2H), 0.86 – 0.82 (m, 2H). **^{13}C NMR (101 MHz, $CDCl_3$)** δ 137.3, 132.7, 131.9, 130.3, 129.6, 128.2, 126.6, 126.5, 126.3, 126.0, 125.1, 124.5, 122.9, 122.4, 13.8, 6.2. The spectroscopic data match the reported literature[18].

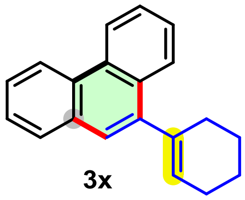

3x

9-(Cyclohex-1-en-1-yl)phenanthrene (3x). Following the typical procedure described above, the reaction was carried out by the mixture of **1a** (56.0 mg, 0.2 mmol, 1.0 equiv), 1-ethynylcyclohex-1-ene (63.7 mg, 0.6 mmol, 3.0 equiv), (dppf)$PdCl_2$ (14.6 mg, 0.02 mmol, 10 mol%), DPEPhos (21.5 mg, 0.04 mmol, 20 mol%) and K_3PO_4 (84.8 mg, 0.4 mmol, 2.0 equiv) in PhH (2.0 mL) at room temperature in nitrogen atmosphere under the irradiation of blue LED lamps for 20 hours. Column chromatography on silica gel (eluent: Petroleum ether) afforded the title product in 55% isolated yield (28.5 mg) as a colorless oil. R_f = 0.7 (Petroleum ether). **^{1}H NMR (400 MHz, $CDCl_3$)** δ 8.73 (d, *J* = 8.0 Hz, 1H), 8.67 (d, *J* = 8.0 Hz, 1H), 8.08 – 8.03 (m, 1H), 7.86 (dd, *J* = 7.8, 1.2 Hz, 1H), 7.68 – 7.56 (m, 4H), 7.55 (s, 1H), 5.87 (s, 1H), 2.45 – 2.40 (m, 2H), 2.34 – 2.29 (m, 2H), 1.93 – 1.87 (m, 2H), 1.86 – 1.80 (m, 2H). **^{13}C NMR (101 MHz, $CDCl_3$)** δ 141.5, 138.1, 131.9, 131.0, 130.5, 129.7, 128.3, 127.2, 126.6, 126.5, 126.3, 126.2, 126.1, 125.2, 122.9, 122.4, 30.9, 25.5, 23.2, 22.3. **HRMS (EI)** calcd for $C_{20}H_{18}$ $[M]^+$: 258.1403, found 258.1406.

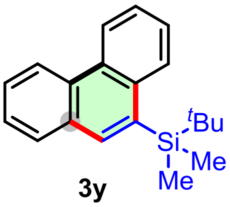

3y

***tert*-Butyldimethyl(phenanthren-9-yl)silane (3y).** Following the typical procedure described above, the reaction was carried out by the mixture of **1a** (56.0 mg, 0.2 mmol, 1.0 equiv), *tert*-butyl(ethynyl)dimethylsilane (84.2 mg, 0.6 mmol, 3.0 equiv), $Pd(OAc)_2$ (4.5 mg, 0.02 mmol, 10 mol%), DPEPhos (21.5 mg, 0.04 mmol, 20 mol%) and K_3PO_4 (84.8 mg, 0.4 mmol, 2.0 equiv) in PhH (2.0 mL) at room temperature in nitrogen atmosphere under the irradiation of blue LED lamps for 20 hours. Column chromatography on silica gel (eluent: Petroleum ether) afforded the title product in 74% isolated yield (32.2 mg) as a colorless oil; R_f = 0.7 (Petroleum ether). **^{1}H NMR (600 MHz, $CDCl_3$)** δ 8.74 (d, *J* = 8.1 Hz, 1H), 8.68 (d, *J* = 8.4 Hz, 1H), 8.19 (d, *J* = 8.1 Hz, 1H), 7.97 (s, 1H), 7.89 (d, *J* = 7.8 Hz, 1H), 7.62 (ddt, *J* = 30.3, 11.4, 7.2 Hz, 4H), 0.98 (s, 9H), 0.57 (s, 6H). **^{13}C NMR (151 MHz, $CDCl_3$)** δ 137.3, 135.6, 134.9, 130.95, 130.88, 130.2, 130.0, 128.8, 127.1, 126.5, 125.9, 125.8, 123.0, 122.3, 27.5, 18.1, -2.7. **HRMS (ESI)** calcd for $C_{20}H_{25}Si$ $[M+H]^+$: 293.1720, found 293.1718.

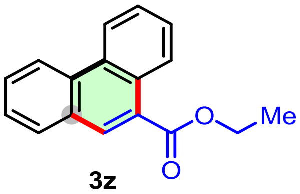

3z

Ethyl phenanthrene-9-carboxylate (3z). Following the typical procedure described above, the reaction was carried out by the mixture of **1a** (56.0 mg, 0.2 mmol, 1.0 equiv), ethyl propiolate (58.8 mg, 0.6 mmol, 3.0 equiv), $Pd(OAc)_2$ (4.5 mg, 0.02 mmol, 10 mol%), DPEPhos (21.5 mg, 0.04 mmol, 20 mol%) and K_3PO_4 (84.4 mg, 0.4 mmol, 2.0 equiv) in PhH (2.0 mL) at room temperature in nitrogen atmosphere under the irradiation of blue LED lamps for 20 hours. Column chromatography on silica gel (eluent: Petroleum ether : Ethyl acetate = 30:1) afforded the title product in 64% isolated yield (32.4 mg) as a brown oil; R_f = 0.5 (Petroleum ether : Ethyl acetate = 20:1). **^{1}H NMR (600 MHz, $CDCl_3$)** δ 8.92 – 8.91 (m, 1H), 8.75 – 8.73 (m, 1H), 8.70 (d, *J* = 8.4 Hz, 1H), 8.46 (s, 1H), 7.98 (d, *J* = 7.8 Hz, 1H), 7.75 (t, *J* = 7.5 Hz, 1H), 7.72 – 7.67 (m, 2H), 7.64 (t, *J* = 7.5 Hz, 1H), 4.53 (q, *J* = 7.2 Hz, 2H), 1.51 (t, *J* = 7.2 Hz, 3H). **^{13}C NMR (151 MHz, $CDCl_3$)** δ 167.7, 132.1, 130.7, 130.1, 129.9, 129.1, 128.9, 127.4, 127.0, 126.9, 126.67, 126.65, 122.8, 122.7, 61.2, 14.5. The spectroscopic data match the reported literature[18].

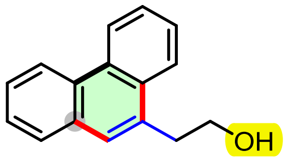

3aa

2-(Phenanthren-9-yl)ethan-1-ol (3aa). Following the typical procedure described above, the reaction was carried out by the mixture of **1a** (56.0 mg, 0.2 mmol, 1.0 equiv), but-3-yn-1-ol (42.1 mg, 0.6 mmol, 3 equiv), (dppf)$PdCl_2$ (14.6 mg, 0.02 mmol, 10 mol%), DPEPhos (21.5 mg, 0.04 mmol, 20 mol%) and K_3PO_4 (84.8 mg, 0.4 mmol, 2.0 equiv) in PhH (2.0 mL) at room temperature in nitrogen atmosphere under the irradiation of blue LED lamps for 20 hours. Column chromatography on silica gel (eluent: Petroleum ether : Ethyl acetate = 7:1) afforded the title product in 68% isolated yield (30.3 mg) as a white solid; R_f = 0.4 (Petroleum ether : Ethyl acetate = 2:1). **^{1}H NMR (400 MHz, $CDCl_3$)** δ 8.76 (d, J = 8.1 Hz, 1H), 8.67 (d, J = 8.0 Hz, 1H), 8.11 (d, J = 7.4 Hz, 1H), 7.85 (d, J = 7.5 Hz, 1H), 7.74 – 7.49 (m, 5H), 4.06 (t, J = 6.6 Hz, 2H), 3.41 (t, J = 6.6 Hz, 2H), 1.59 (s, 1H). **^{13}C NMR (151 MHz, $CDCl_3$)** δ 132.4, 131.6, 131.1, 130.9, 129.9, 128.1, 127.7, 126.8, 126.7, 126.40, 126.35, 124.2, 123.3, 122.5, 62.5, 36.6. The spectroscopic data match the reported literature[25].

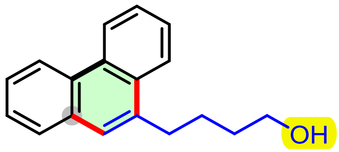

3ab

4-(Phenanthren-9-yl)butan-1-ol (3ab). Following the typical procedure described above, the reaction was carried out by the mixture of **1a** (56.0 mg, 0.2 mmol, 1.0 equiv), hex-5-yn-1-ol (58.9 mg, 0.6 mmol, 3 equiv), $Pd(OAc)_2$ (4.5 mg, 0.02 mmol, 10 mol%), DPEPhos (21.5 mg, 0.04 mmol, 20 mol%) and K_3PO_4 (84.8 mg, 0.4 mmol, 2.0 equiv) in PhH (2.0 mL) at room temperature in nitrogen atmosphere under the irradiation of blue LED lamps for 20 hours. Column chromatography on silica gel (eluent: Petroleum ether : Ethyl acetate = 5:1) afforded the title product in 63% isolated yield (31.6 mg) as a white solid; R_f = 0.5 (Petroleum ether : Ethyl acetate = 2:1). **^{1}H NMR (600 MHz, $CDCl_3$)** δ 8.74 (d, J = 7.6 Hz, 1H), 8.66 (d, J = 7.9 Hz, 1H), 8.10 (d, J = 2.0 Hz, 1H), 7.83 (dd, J = 7.6, 1.6 Hz, 1H), 7.71 – 7.55 (m, 5H), 3.73 (t, J = 6.5 Hz, 2H), 3.16 (t, J = 7.7 Hz, 2H), 1.91 (m, 2H), 1.80 – 1.72 (m, 2H), 1.41(s, 1H). **^{13}C NMR (151 MHz, $CDCl_3$)** δ 136.4, 131.8, 131.2, 130.7, 129.6, 128.0, 126.6, 126.5, 126.1, 125.9, 124.4, 123.2, 122.4, 62.8, 33.1, 32.8, 26.3. The spectroscopic data match the reported literature[26].

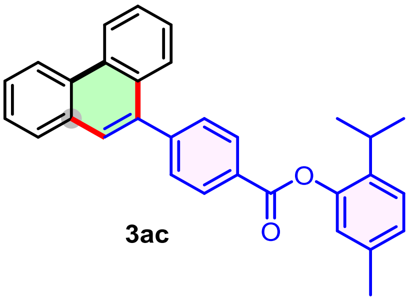

3ac

2-Isopropyl-5-methylphenyl 4-(phenanthren-9-yl)benzoate (3ac). Following the typical procedure described above, the reaction was carried out by the mixture of **1a** (56.0 mg, 0.2 mmol, 1.0 equiv), 2-isopropyl-5-methylphenyl 4-ethynylbenzoate (167.4 mg, 0.6 mmol, 3 equiv), $Pd(OAc)_2$ (4.5 mg, 0.02 mmol, 10 mol%), DPEPhos (43.1 mg, 0.08 mmol, 40 mol%) and K_3PO_4 (84.8 mg, 0.4 mmol, 2.0 equiv) in PhH (2.0 mL) at room temperature in nitrogen atmosphere under the irradiation of blue LED lamps for 20 hours. Column chromatography on silica gel (eluent: Petroleum ether : Ethyl acetate = 80:1) afforded the title product in 73% isolated yield (40.7 mg) as a white solid; R_f = 0.5 (Petroleum ether : Ethyl acetate = 50:1). **^{1}H NMR (600 MHz, $CDCl_3$)** δ 8.81 (d, *J* = 8.1 Hz, 1H), 8.75 (d, *J* = 8.1 Hz, 1H), 8.37 (d, *J* = 7.5 Hz, 2H), 7.92 (dd, *J* = 14.1, 8.1 Hz, 2H), 7.73 – 7.70 (m, 5H), 7.65 (t, *J* = 7.2 Hz, 1H), 7.58 (t, *J* = 7.5 Hz, 1H), 7.29 (d, *J* = 7.8 Hz, 1H), 7.10 (d, *J* = 7.8 Hz, 1H), 7.01 (s, 1H), 3.18 – 3.11 (m, 1H), 2.38 (s, 3H), 1.27 (d, *J* = 6.8 Hz, 6H). **^{13}C NMR (151 MHz, $CDCl_3$)** δ 165.3, 148.2, 146.4, 137.6, 137.3, 136.7, 131.3, 130.7, 130.6, 130.4, 130.23, 130.21, 128.8, 128.7, 127.8, 127.3, 127.07, 127.05, 126.8, 126.58, 126.55, 123.1, 122.9, 122.6, 27.4, 23.1, 20.9. **HRMS (ESI)** calcd for $C_{31}H_{27}O_2$ $[M+H]^+$: 431.2006, found 431.2004.

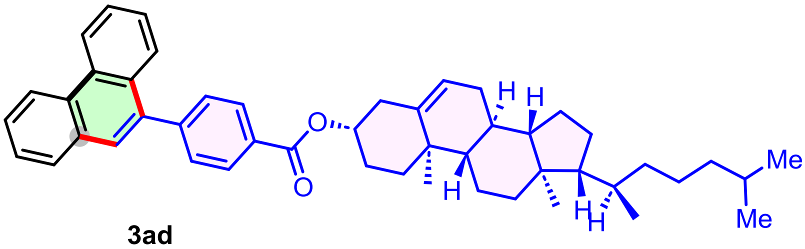

3ad

(3*S*,8*S*,9*S*,10*R*,13*R*,14*S*,17*R*)-10,13-Dimethyl-17-((*R*)-6-methylheptan-2-yl)-2,3,4,7,8,9,10,11,12,13, 14,15,16,17-tetradecahydro-1*H*-cyclopenta[*a*]phenanthren-3-yl 4-(phenanthren-9-yl)benzoate (3ad). Following the typical procedure described above, the reaction was carried out by the mixture of **1a** (56.0 mg, 0.2 mmol, 1.0 equiv), (3*S*,8*S*,9*S*,10*R*,13*R*,14*S*,17*R*)-10,13-dimethyl-17-((*R*)-6-methylheptan-2-yl)-2,3,4,7,8,9,10,11,12,13,14,15,16,17-tetradecahydro-1*H*-cyclopenta[*a*]phenanthren-3-yl 4-ethynylbenzo-ate (308.9 mg, 0.6 mmol, 3.0 equiv), $Pd(OAc)_2$ (4.5 mg, 0.02 mmol, 10 mol%), DPEPhos (21.5 mg, 0.04 mmol, 20 mol%) and K_3PO_4 (84.8 mg, 0.4 mmol, 2.0 equiv) in PhH (2.0 mL) at room temperature in nitrogen atmosphere under the irradiation of blue LED lamps for 20 hours. Column chromatography on silica gel (eluent: Petroleum ether : Ethyl acetate = 50:1) afforded the title product in

61% isolated yield (81.4 mg) as a white oil; R_f = 0.6(Petroleum ether : Ethyl acetate = 20:1). **^{1}H NMR (600 MHz, $CDCl_3$)** δ 8.79 (d, *J* = 8.4 Hz, 1H), 8.73 (d, *J* = 8.4 Hz, 1H), 8.22 – 8.17 (m, 2H), 7.91 (dd, *J* = 8.0, 1.4 Hz, 1H), 7.85 (dd, *J* = 8.4, 1.2 Hz, 1H), 7.72 – 7.66 (m, 3H), 7.66 – 7.61 (m, 3H), 7.54 (t, *J* = 7.6 Hz, 1H), 5.46 (dd, *J* = 4.8, 2.0 Hz, 1H), 4.93 (ddt, *J* = 11.8, 8.2, 4.4 Hz, 1H), 2.52 (d, *J* = 8.2 Hz, 2H), 2.07 – 2.00 (m, 3H), 1.97 – 1.93 (m, 1H), 1.86 – 1.77 (m, 2H), 1.63 – 1.46 (m, 9H), 1.42 – 1.32 (m, 4H), 1.29 – 1.24 (m, 5H), 1.10 (s, 3H), 1.02 (td, *J* = 7.1, 6.6, 4.2 Hz, 2H), 0.93 (d, *J* = 6.4 Hz, 3H), 0.88 (dd, *J* = 6.6, 2.8 Hz, 6H), 0.70 (s, 3H). **^{13}C NMR (151 MHz, $CDCl_3$)** δ 165.9, 145.4, 139.7, 137.8, 131.3, 130.6, 130.1, 130.0, 129.9, 129.6, 128.8, 127.6, 127.0, 126.9, 126.7, 126.6, 123.0, 122.8, 122.6, 74.7, 56.7, 56.1, 50.1, 42.3, 39.7, 39.5, 38.3, 37.1, 36.7, 36.2, 35.8, 31.9, 31.9, 28.2, 28.0, 27.9, 24.3, 23.8, 22.8, 22.6, 21.1, 19.4, 18.7, 11.9. **HRMS (ESI)** calcd for $C_{48}H_{59}O_2$ $[M+H]^+$: 667.4510, found 667.4508.

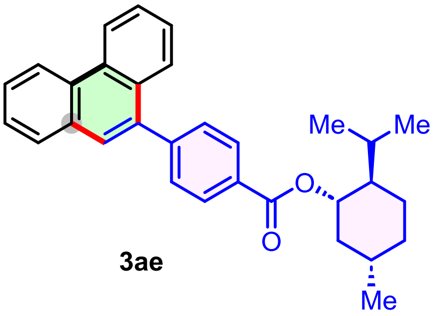

3ae

(1*S*,2*R*,5*S*)-2-Isopropyl-5-methylcyclohexyl 4-(phenanthren-9-yl)benzoate (3ae). Following the typical procedure described above, the reaction was carried out by the mixture of **1a** (56.0 mg, 0.2 mmol, 1.0 equiv), (1*S*,2*R*,5*S*)-2-isopropyl-5-methylcyclohexyl 4-ethynylbenzoate (170.6 mg, 0.6 mmol, 3.0 equiv), $Pd(OAc)_2$ (4.5 mg, 0.02 mmol, 10 mol%), DPEPhos (21.5 mg, 0.04 mmol, 20 mol%) and K_3PO_4 (84.8 mg, 0.4 mmol, 2.0 equiv) in PhH (2.0 mL) at room temperature in nitrogen atmosphere under the irradiation of blue LED lamps for 20 hours. Column chromatography on silica gel (eluent: Petroleum ether) afforded the title product in 64% isolated yield (56.6 mg) as a white solid; M.p. 121.2 – 125.6 °C. R_f = 0.6 (Petroleum ether : Ethyl acetate = 70:1). **^{1}H NMR (400 MHz, $CDCl_3$)** δ 8.79 (d, *J* = 8.4 Hz, 1H), 8.74 (d, *J* = 8.0 Hz, 1H), 8.21 (d, *J* = 8.0 Hz, 2H), 7.89 (dd, *J* = 16.0, 8.0 Hz, 2H), 7.71 – 7.63 (m, 6H), 7.55 (t, *J* = 7.6 Hz, 1H), 5.02 (td, *J* = 10.8, 4.4 Hz, 1H), 2.21 (dd, *J* = 12.0, 4.0 Hz, 1H), 2.09 – 2.02 (m, 1H), 1.79 – 1.74 (m, 2H), 1.65 – 1.58 (m, 1H), 1.35 – 1.26 (m, 1H), 1.23 – 1.12 (m, 1H), 0.97 (d, *J* = 6.8 Hz, 6H), 0.86 (d, *J* = 7.2 Hz, 3H). **^{13}C NMR (101 MHz, $CDCl_3$)** δ 166.0, 145.4, 137.8, 131.3, 130.6, 130.1, 129.8, 129.6, 128.8, 127.6, 127.0, 126.9, 126.65, 126.59, 123.0, 122.5, 74.9, 47.3, 41.0, 34.3, 31.5, 26.5, 23.6, 22.1, 20.8, 16.5. **HRMS (ESI)** calcd for $C_{31}H_{36}NO_2$ $[M+NH_4]^+$: 454.2741, found 454.2749.

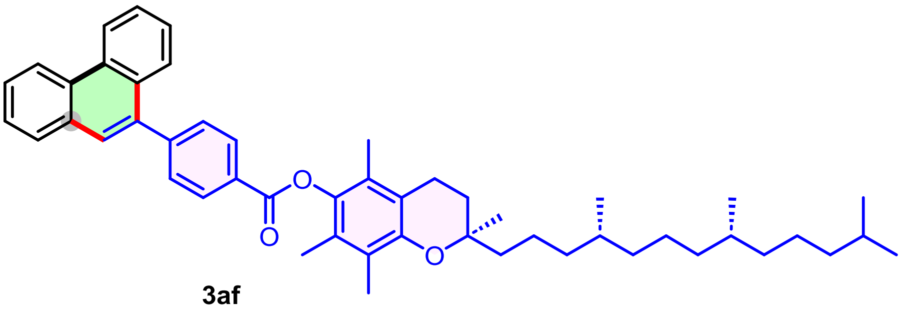

3af

(*R*)-2,5,7,8-Tetramethyl-2-((4*R*,8*R*)-4,8,12-trimethyltridecyl)chroman-6-yl 4-(phenanthren-9-yl)benzoate (3af). Following the typical procedure described above, the reaction was carried out by the mixture of **1a** (56.0 mg, 0.2 mmol, 1.0 equiv), (*R*)-2,5,7,8-tetramethyl-2-((4*R*,8*R*)-4,8,12-trimethyltridecyl)chroman-6-yl 4-ethynylbenzoate (335.3 mg, 0.6 mmol, 3 equiv), $Pd(OAc)_2$ (4.5 mg, 0.02 mmol, 10 mol%), DPEPhos (21.5 mg, 0.04 mmol, 20 mol%) and K_3PO_4 (84.8 mg, 0.4 mmol, 2.0 equiv) in PhH (2.0 mL) at room temperature in nitrogen atmosphere under the irradiation of blue LED lamps for 20 hours. Column chromatography on silica gel (eluent: Petroleum ether : Ethyl acetate = 50:1) afforded the title product in 59% isolated yield (83.9 mg) as a white oil; R_f = 0.6(Petroleum ether : Ethyl acetate = 20:1). **^{1}H NMR (600 MHz, $CDCl_3$)** δ 8.81 (d, *J* = 8.3 Hz, 1H), 8.75 (d, *J* = 8.3 Hz, 1H), 8.40 (d, *J* = 7.9 Hz, 2H), 7.95 – 7.90 (m, 2H), 7.76 – 7.69 (m, 5H), 7.65 (t, *J* = 7.4 Hz, 1H), 7.60 – 7.56 (m, 1H), 2.66 (t, *J* = 6.9 Hz, 2H), 2.16 (s, 3H), 2.14 (s, 3H), 2.09 (s, 3H), 1.96 – 1.71 (m, 3H), 1.63 – 1.53 (m, 4H), 1.46 – 1.35 (m, 4H), 1.32 – 1.24 (m, 10H), 1.16 – 1.06 (m, 5H), 0.87 (d, *J* = 6.8 Hz, 12H). **^{13}C NMR (151 MHz, $CDCl_3$)** δ 165.1, 149.5, 146.2, 140.7, 137.7, 131.3, 130.7, 130.6, 130.3, 130.2, 130.2, 128.8, 128.6, 127.7, 127.0, 127.0, 126.9, 126.7, 126.7, 126.6, 125.2, 123.2, 123.0, 122.6, 117.5, 75.1, 40.4, 39.7, 39.4, 37.5, 37.3, 32.8, 31.3, 29.7, 28.0, 24.8, 24.5, 24.2, 23.7, 22.7, 22.6, 21.0, 20.7, 19.8, 19.7, 13.1, 12.3, 11.9. **HRMS (ESI)** calcd for $C_{50}H_{66}NO_3$ $[M+NH_4]^+$: 728.5037, found 728.5037.

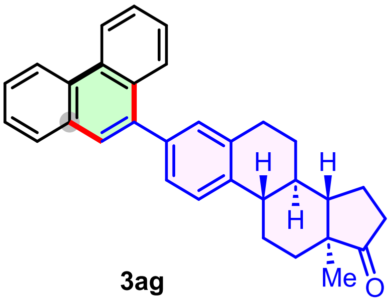

3ag

(8*R*,9*S*,13*S*,14*S*)-13-Methyl-3-(phenanthren-9-yl)-6,7,8,9,11,12,13,14,15,16-decahydro-17*H*-cyclopenta[*a*]phenanthren-17-one (3x). Following the typical procedure described above, the reaction was carried out by the mixture of 1a (56.0 mg, 0.2 mmol, 1.0 equiv), (8*R*,9*S*,13*S*,14*S*)-3-ethynyl-13-methyl-6,7,8,9,11,12,13,14,15,16-decahydro-17*H*-cyclopenta[*a*]phenanthren-17-one (167.04 mg, 0.6 mmol, 3.0 equiv), $Pd(OAc)_2$ (4.5 mg, 0.02 mmol, 10 mol%), DPEPhos (21.5 mg, 0.04 mmol, 20 mol%) and K_3PO_4 (84.8 mg, 0.4 mmol, 2.0 equiv) in PhH (2.0 mL) at room temperature in nitrogen atmosphere under the irradiation of blue LED lamps for 20 hours. Column chromatography on silica gel (eluent:

Petroleum ether) afforded the title product in 71% isolated yield (61.2 mg) as a white solid; M.p. 107.5 - 109.7 °C. R_f = 0.6(Petroleum ether : Ethyl acetate = 50:1). **^{1}H NMR (400 MHz, $CDCl_3$)** δ 8.78 (d, *J* = 8.4 Hz, 1H), 8.72 (d, *J* = 8.0 Hz, 1H), 7.98 (d, *J* = 8.4 Hz, 1H), 7.88 (d, *J* = 7.6 Hz, 1H), 7.68 – 7.59 (m, 4H), 7.54 (t, *J* = 7.6 Hz, 1H), 7.43 (t, *J* = 8.0 Hz, 1H), 7.36 – 7.29 (m, 2H), 3.03 – 2.93 (m, 2H), 2.59 – 2.39 (m, 4H), 2.29 – 1.97 (m, 6H), 1.78 – 1.65 (m, 3H), 0.98 (s, 3H).8.78 (d, *J* = 8.4 Hz, 1H), 8.72 (d, *J* = 8.0 Hz, 1H), 7.98 (d, *J* = 8.4 Hz, 1H), 7.88 (d, *J* = 7.6 Hz, 1H), 7.68 – 7.59 (m, 4H), 7.54 (t, *J* = 7.6 Hz, 1H), 7.43 (t, *J* = 8.0 Hz, 1H), 7.36 – 7.29 (m, 2H), 3.03 – 2.93 (m, 2H), 2.59 – 2.39 (m, 4H), 2.29 – 1.97 (m, 6H), 1.78 – 1.65 (m, 3H), 0.98 (s, 3H).**^{13}C NMR (101 MHz, $CDCl_3$)** δ 220.9, 138.9, 138.6, 138.2, 136.5, 131.6, 131.1, 130.6, 130.5, 129.8, 128.6, 127.5, 127.4, 127.0, 126.8, 126.5, 126.40, 126.37, 125.2, 122.9, 122.5, 50.5, 48.0, 44.5, 38.2, 35.9, 31.6, 29.5, 26.6, 25.8, 21.6, 13.9. **HRMS (ESI)** calcd for $C_{32}H_{31}O$ $[M+H]^+$: 431.2369, found 431.2370.

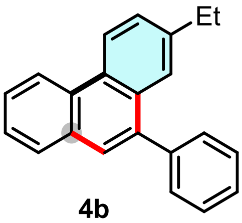

4b

2-Ethyl-10-phenylphenanthrene (4b). Following the typical procedure described above, the reaction was carried out by the mixture of **1b** (61.6 mg, 0.2 mmol, 1.0 equiv), ethynylbenzene (61.2 mg, 0.6 mmol, 3.0 equiv), (dppf)$PdCl_2$ (14.6 mg, 0.02 mmol, 10 mol%), DPEPhos (21.5 mg, 0.04 mmol, 20 mol%) and K_3PO_4 (84.8 mg, 0.4 mmol, 2.0 equiv) in PhH (2.0 mL) at room temperature in nitrogen atmosphere under the irradiation of blue LED lamps for 20 hours. Column chromatography on silica gel (eluent: Petroleum ether) afforded the title product in 65% isolated yield (36.7mg) and as a yellow oil; R_f = 0.6 (Petroleum ether). **^{1}H NMR (400 MHz, $CDCl_3$)** δ 8.75 (d, *J* = 4.0 Hz, 1H), 8.73 (d, *J* = 3.6 Hz, 1H), 7.92 (d, *J* = 7.6 Hz, 1H), 7.79 (s, 1H), 7.72 – 7.67 (m, 2H), 7.65 – 7.56 (m, 6H), 7.53 – 7.50 (m, 1H), 2.82 (q, *J* = 7.6 Hz, 2H), 1.32 (t, *J* = 7.6 Hz, 3H). **^{13}C NMR (101 MHz, $CDCl_3$)** δ 142.6, 140.9, 138.6, 131.2, 130.03, 129.97, 128.7, 128.6, 128.3, 127.6, 127.2, 127.0, 126.5, 126.4, 125.3, 122.9, 122.3, 29.0, 15.7. **HRMS (EI)** calcd for $C_{22}H_{18}$ $[M]^+$: 282.1403, found 282.1408.

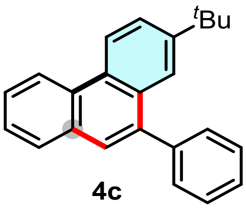

4c

2-(*tert*-Butyl)-10-phenylphenanthrene (4c). Following the typical procedure described above, the reaction was carried out by the mixture of **1c** (67.2 mg, 0.2 mmol, 1.0 equiv), ethynylbenzene (61.2 mg,

0.6 mmol, 3.0 equiv), $Pd(OAc)_2$ (4.5 mg, 0.02 mmol, 10 mol%), DPEPhos (21.6 mg, 0.04 mmol, 20 mol%) and K_3PO_4 (84.8 mg, 0.4 mmol, 2.0 equiv) in PhH (2.0 mL) at room temperature in nitrogen atmosphere under the irradiation of blue LED lamps for 20 hours. Column chromatography on silica gel (eluent: Petroleum ether) afforded the title product in 57% isolated yield (35.4 mg) as a brown oil; R_f = 0.6 (Petroleum ether). **^{1}H NMR (400 MHz, $CDCl_3$)** δ 8.74 (t, *J* = 8.0 Hz, 2H), 8.00 (d, *J* = 2.0 Hz, 1H), 7.92 (dd, *J* = 8.0, 1.6 Hz, 1H), 7.79 (dd, *J* = 8.8, 2.0 Hz, 1H), 7.72 – 7.66 (m, 2H), 7.64 – 7.55 (m, 5H), 7.52 – 7.48 (m, 1H), 1.39 (s, 9H). **^{13}C NMR (101 MHz, $CDCl_3$)** δ 149.2, 140.9, 138.9, 131.3, 130.8, 130.0, 129.8, 128.6, 128.4, 128.2, 127.5, 127.3, 126.43, 126.39, 124.6, 122.7, 122.6, 122.3, 34.9, 31.3. The spectroscopic data match the reported literature[18].

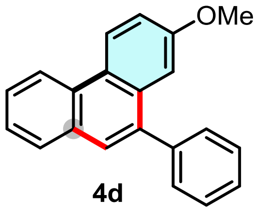

4d

2-Methoxy-10-phenylphenanthrene (4d). Following the typical procedure described above, the reaction was carried out by the mixture of **1d** (62.0 mg, 0.2 mmol, 1.0 equiv), ethynylbenzene (61.2 mg, 0.6 mmol, 3.0 equiv), (dppf)$PdCl_2$ (14.6 mg, 0.02 mmol, 10 mol%), DPEPhos (21.5 mg, 0.04 mmol, 20 mol%) and K_3PO_4 (84.8 mg, 0.4 mmol, 2.0 equiv) in PhH (2.0 mL) at room temperature in nitrogen atmosphere under the irradiation of blue LED lamps for 20 hours. Column chromatography on silica gel (eluent: Petroleum ether) afforded the title product in 66% isolated yield (37.6 mg) as a brown oil; R_f = 0.5 (Petroleum ether). **^{1}H NMR (400 MHz, $CDCl_3$)** δ 8.72 (d, *J* = 9.2 Hz, 1H), 8.66 (d, *J* = 8.4 Hz, 1H), 7.92 (d, *J* = 7.6 Hz, 1H), 7.74 (s, 1H), 7.68 (t, *J* = 7.6 Hz, 1H), 7.60 (dt, *J* = 17.6, 7.2 Hz, 5H), 7.51 (t, *J* = 7.2 Hz, 1H), 7.41 (d, *J* = 2.8 Hz, 1H), 7.35 (dd, *J* = 9.2, 2.4 Hz, 1H), 3.84 (s, 3H). **^{13}C NMR (101 MHz, $CDCl_3$)** δ 158.1, 140.8, 138.1, 132.5, 130.5, 130.0, 129.8, 128.6, 128.3, 128.1, 127.3, 126.6, 125.8, 124.9, 124.5, 122.0, 116.3, 113.3, 107.8, 55.2. The spectroscopic data match the reported literature[18].

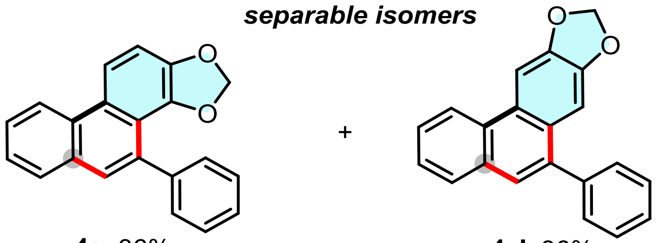

4e, 36% **4e'**, 36%

4-Phenylphenanthro[1,2-*d*][1,3]dioxole (4e Contains 14% of **4e').** Following the typical procedure described above, the reaction was carried out by the mixture of **1e** (64.8 mg, 0.2 mmol, 1.0 equiv), ethynylbenzene (61.2 mg, 0.6 mmol, 3.0 equiv), $Pd(OAc)_2$ (4.5 mg, 0.02 mmol, 10 mol%), DPEPhos

(21.5 mg, 0.04 mmol, 20 mol%) and K_3PO_4 (84.8 mg, 0.4 mmol, 2.0 equiv) in PhH (2.0 mL) at room temperature in nitrogen atmosphere under the irradiation of blue LED lamps for 20 hours. Column chromatography on silica gel (eluent: Petroleum ether/ Ethyl acetate = 50:1) afforded the title product **4e** in 36% isolated (21.5 mg) as a colorless oil and **4e'** in 36% isolated yield (21.5 mg) as a colorless oil; R_f = 0.5 (Petroleum ether/ Ethyl acetate = 20:1). **^{1}H NMR (400 MHz, CDCl$_3$)** δ 8.58 (d, *J* = 8.4 Hz, 1H), 8.51 (d, *J* = 8.4 Hz, 0.14H), 8.35 (d, *J* = 8.7 Hz, 1H), 8.12 (s, 0.15H), 7.87 (d, *J* = 8.0 Hz, 0.15H), 7.81 (d, *J* = 7.8 Hz, 1H), 7.61 (d, *J* = 8.4 Hz, 1H), 7.59 – 7.47 (m, 4.9H), 7.48 – 7.39 (m, 3.17H), 7.32 – 7.24 (m, 1.52H), 6.08 (s, 0.3H), 5.88 (s, 2H). **^{13}C NMR (101 MHz, CDCl$_3$)** δ 145.6, 141.9, 134.7, 130.6, 130.0, 129.9, 129.6, 129.5, 128.8, 128.4, 127.3, 127.1, 127.0, 126.7, 126.3, 122.5, 117.0, 109.2, 101.0. **HRMS (EI)** calcd for $C_{21}H_{14}O_2$ $[M]^+$: 298.0988, found 298.0990.

6-Phenylphenanthro[2,3-*d*][1,3]dioxole (4e'). **^{1}H NMR (400 MHz, CDCl$_3$)** δ 8.50 (d, *J* = 8.4 Hz, 1H), 8.11 (s, 1H), 7.86 (d, *J* = 8.0 Hz, 1H), 7.64 – 7.38 (m, 8H), 7.25 (s, 1H), 6.09 (s, 2H). **^{13}C NMR (101 MHz, CDCl$_3$)** δ 147.8, 147.5, 141.1, 138.4, 131.0, 129.9, 129.6, 128.6, 128.3, 127.6, 127.4, 126.9, 126.3, 126.00, 125.98, 122.3, 104.5, 101.4, 101.0. **HRMS (EI)** calcd for $C_{21}H_{14}O_2$ $[M]^+$: 298.0988, found 298.0994.

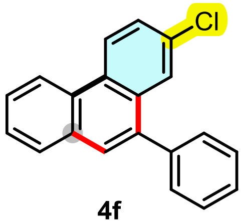

4f

2-Chloro-10-phenylphenanthrene (4f). Following the typical procedure described above, the reaction was carried out by the mixture of **1f** (62.9 mg, 0.2 mmol, 1.0 equiv), ethynylbenzene (61.2 mg, 0.6 mmol, 3.0 equiv), (dppf)$PdCl_2$ (14.6 mg, 0.02 mmol, 10 mol%), DPEPhos (21.5 mg, 0.04 mmol, 20 mol%) and K_3PO_4 (84.8 mg, 0.4 mmol, 2.0 equiv) in PhH (2.0 mL) at room temperature in nitrogen atmosphere under the irradiation of blue LED lamps for 20 hours. Column chromatography on silica gel (eluent: Petroleum ether) afforded the title product in 74% isolated yield (42.7 mg) as a yellow oil; R_f = 0.6 (eluent: Petroleum ether). **^{1}H NMR (400 MHz, CDCl$_3$)** δ 8.69 (d, *J* = 8.8 Hz, 1H), 8.66 (d, *J* = 8.0 Hz, 1H), 7.91 – 7.89 (m, 2H), 7.72 – 7.60 (m, 4H), 7.57 – 7.48 (m, 5H). **^{13}C NMR (101 MHz, CDCl$_3$)** δ 140.0, 137.8, 132.6, 132.3, 131.4, 129.9, 129.5, 128.9, 128.8, 128.7, 128.5, 127.6, 127.1, 127.0, 126.9, 126.0, 124.5. The spectroscopic data match the reported literature[18].

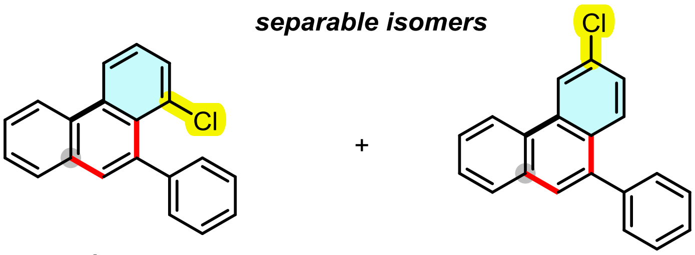

4g, 42%   **4g'**, 28%

1-Chloro-10-phenylphenanthrene (4g). Following the typical procedure described above, the reaction was carried out by the mixture of **1h** (62.9 mg, 0.2 mmol, 1.0 equiv), ethynylbenzene (61.2 mg, 0.6 mmol, 3.0 equiv), $Pd(OAc)_2$ (4.5 mg, 0.02 mmol, 10 mol%), DPEPhos (21.5 mg, 0.04 mmol, 20 mol%) and K_3PO_4 (84.8 mg, 0.4 mmol, 2.0 equiv) in PhH (2.0 mL) at room temperature in nitrogen atmosphere under the irradiation of blue LED lamps for 20 hours. Column chromatography on silica gel (eluent: Petroleum ether) afforded the title product **4g** in 42% isolated yield (24.3 mg) as a yellow oil and **4g'** in 28% isolated yield as a yellow oil (16.2 mg); R_f = 0.6 (eluent: Petroleum ether). **^{1}H NMR (400 MHz, $CDCl_3$)** δ 8.76 (dd, J = 8.0, 1.6 Hz, 1H), 8.69 (d, J = 8.0 Hz, 1H), 7.86 (dd, J = 7.6, 1.6 Hz, 1H), 7.71 – 7.61 (m, 4H), 7.57 (t, J = 8.0 Hz, 1H), 7.45 – 7.37 (m, 5H). **^{13}C NMR (101 MHz, $CDCl_3$)** δ 143.8, 137.1, 133.3, 132.5, 131.8, 130.9, 130.1, 129.8, 129.3, 128.5, 128.0, 127.54, 127.48, 127.1, 126.7, 126.5, 122.9, 122.1. The spectroscopic data match the reported literature[23].

3-Chloro-10-phenylphenanthrene (4g'). **^{1}H NMR (400 MHz, $CDCl_3$)** δ 8.73 (d, J = 2.0 Hz, 1H), 8.63 (d, J = 8.0 Hz, 1H), 7.90 (dd, J = 7.6, 1.6 Hz, 1H), 7.85 (d, J = 8.8 Hz, 1H), 7.71 – 7.63 (m, 3H), 7.52 (d, J = 4.0 Hz, 4H), 7.49 – 7.45 (m, 2H). **^{13}C NMR (101 MHz, $CDCl_3$)** δ 140.3, 138.3, 132.6, 131.9, 131.8, 130.0, 129.4, 128.9, 128.7, 128.5, 128.4, 127.7, 127.6, 127.5, 126.90, 126.85, 122.6, 122.5. The spectroscopic data match the reported literature[23].

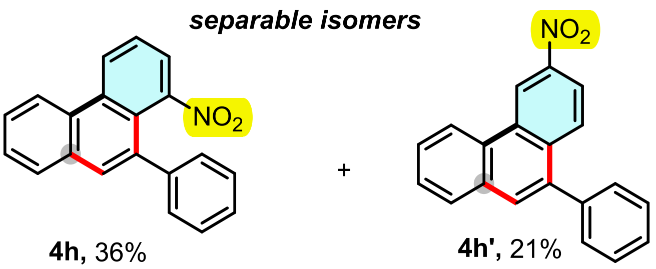

4h, 36%   **4h'**, 21%

1-Nitro-10-phenylphenanthrene (4h). Following the typical procedure described above, the reaction was carried out by the mixture of **1h** (65.0 mg, 0.2 mmol, 1.0 equiv), ethynylbenzene (61.2 mg, 0.6 mmol, 3.0 equiv), $Pd(OAc)_2$ (4.5 mg, 0.02 mmol, 10 mol%), DPEPhos (21.5 mg, 0.04 mmol, 20 mol%) and K_3PO_4 (84.8 mg, 0.4 mmol, 2.0 equiv) in PhH (2.0 mL) at room temperature in nitrogen atmosphere under the irradiation of blue LED lamps for 20 hours. Column chromatography on silica gel (eluent: Petroleum ether/ Ethyl acetate = 20:1) afforded the title product **4h** in 36% isolated yield (21.6 mg) as a white solid and **4h'** in 21% isolated yield (12.6 mg) as a white solid; R_f = 0.5 (Petroleum ether/ Ethyl acetate = 10:1).

^{1}H NMR (400 MHz, CDCl$_3$) δ 9.00 (d, J = 8.4 Hz, 1H), 8.71 (d, J = 8.0 Hz, 1H), 7.93 (dd, J = 17.2, 7.6 Hz, 2H), 7.84 (s, 1H), 7.73 (p, J = 7.6 Hz, 3H), 7.42 (s, 5H). **^{13}C NMR (101 MHz, CDCl$_3$)** δ 143.4, 140.7, 134.9, 133.2, 133.0, 131.5, 128.9, 128.6, 128.3, 128.2, 127.8, 127.7, 127.0, 125.4, 123.6, 122.8. **HRMS (ESI)** calcd for $C_{20}H_{14}NO_2$ $[M+H]^+$: 300.1019, found 300.1020.

3-Nitro-10-phenylphenanthrene(4h'). **^{1}H NMR (600 MHz, CDCl$_3$)** δ 9.66 (s, 1H), 8.78 (d, J = 8.4 Hz, 1H), 8.29 (d, J = 9.0 Hz, 1H), 8.04 (d, J = 9.0 Hz, 1H), 7.96 (d, J = 7.8 Hz, 1H), 7.88 (s, 1H), 7.79 (t, J = 7.8 Hz, 1H), 7.73 (t, J = 7.2 Hz, 1H), 7.53 (dq, J = 16.8, 9.0, 8.4 Hz, 5H). **^{13}C NMR (151 MHz, CDCl$_3$)** δ 145.8, 139.5, 138.1, 134.8, 131.9, 131.4, 130.4, 130.0, 129.9, 129.0, 128.6, 128.3, 128.2, 128.0, 127.8, 122.8, 120.1, 119.2. **HRMS (ESI)** calcd for $C_{20}H_{14}NO_2$ $[M+H]^+$: 300.1019, found 300.1022.

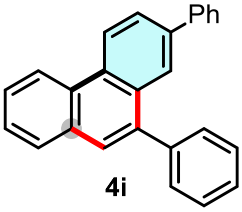

4i

2,10-Diphenylphenanthrene (4i). Following the typical procedure described above, the reaction was carried out by the mixture of **1i** (71.2 mg, 0.2 mmol, 1.0 equiv), ethynylbenzene (61.2 mg, 0.6 mmol, 3.0 equiv), Pd(OAc)$_2$ (4.5 mg, 0.02 mmol, 10 mol%), DPEPhos (21.5 mg, 0.04 mmol, 20 mol%) and K_3PO_4 (84.8 mg, 0.4 mmol, 2.0 equiv) in PhH (2.0 mL) at room temperature in nitrogen atmosphere under the irradiation of blue LED lamps for 20 hours. Column chromatography on silica gel (eluent: Petroleum ether) afforded the title product in 76% isolated yield (50.2 mg) as a colorless oil; R_f = 0.5 (Petroleum ether/ Ethyl acetate = 100:1). **^{1}H NMR (400 MHz, CDCl3)** δ 8.85 (d, J = 8.4 Hz, 1H), 8.75 (d, J = 8.0 Hz, 1H), 8.17 (s, 1H), 7.93 (t, J = 8.0 Hz, 2H), 7.74 – 7.68 (m, 2H), 7.63 (dd, J = 12.4, 7.2 Hz, 5H), 7.55 (t, J = 7.2 Hz, 2H), 7.48 – 7.44 (m, 3H), 7.36 (t, J = 7.6 Hz, 1H). **^{13}C NMR (101 MHz, CDCl$_3$)** δ 141.0, 140.7, 139.1, 138.9, 131.6, 131.4, 130.0, 129.79, 129.75, 128.8, 128.7, 128.4, 128.0, 127.42, 127.37, 127.3, 126.9, 126.7, 125.7, 125.0, 123.5, 122.6. **HRMS (EI)** calcd for $C_{26}H_{18}$ $[M]^+$: 330.1403, found 330.1408.

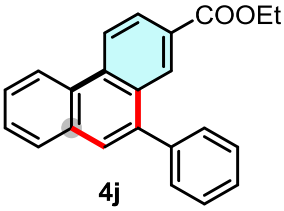

4j

Ethyl 10-phenylphenanthrene-2-carboxylate (4j). Following the typical procedure described above, the reaction was carried out by the mixture of **1j** (70.4 mg, 0.2 mmol, 1.0 equiv), ethynylbenzene (61.2 mg, 0.6 mmol, 3.0 equiv), Pd(OAc)$_2$ (4.5 mg, 0.02 mmol, 10 mol%), DPEPhos (21.5 mg, 0.04 mmol, 20

mol%) and K_3PO_4 (84.8 mg, 0.4 mmol, 2.0 equiv) in PhH (2.0 mL) at room temperature in nitrogen atmosphere under the irradiation of blue LED lamps for 20 hours. Column chromatography on silica gel (eluent: Petroleum ether/ Ethyl acetate = 75:1) afforded the title product in 50% isolated yield (32.6 mg) as a colorless oil; R_f = 0.5 (Petroleum ether/ Ethyl acetate = 50:1). **^{1}H NMR (400 MHz, $CDCl_3$)** δ 8.82 (d, J = 8.4 Hz, 1H), 8.75 (d, J = 8.0 Hz, 1H), 8.68 (s, 1H), 8.28 (d, J = 8.8 Hz, 1H), 7.92 (d, J = 7.6 Hz, 1H), 7.75 (s, 1H), 7.69 (p, J = 7.2 Hz, 2H), 7.61 – 7.48 (m, 5H), 4.39 (q, J = 6.8 Hz, 2H), 1.39 (t, J = 7.2 Hz, 3H). **^{13}C NMR (101 MHz, $CDCl_3$)** δ 166.8, 140.0, 139.2, 133.6, 132.4, 130.6, 130.0, 129.3, 129.2, 128.8, 128.5, 128.3, 127.9, 127.7, 126.9, 126.3, 123.15, 123.10, 61.1, 14.3. The spectroscopic data match the reported literature[27].

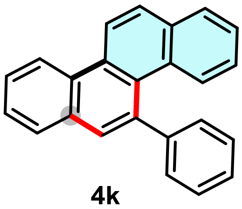

4k

5-Phenylchrysene (4k). Following the typical procedure described above, the reaction was carried out by the mixture of **1k** (66.0 mg, 0.2 mmol, 1.0 equiv), ethynylbenzene (61.2 mg, 0.6 mmol, 3.0 equiv), $Pd(OAc)_2$ (4.5 mg, 0.02 mmol, 10 mol%), DPEPhos (21.5 mg, 0.04 mmol, 20 mol%) and K_3PO_4 (84.8 mg, 0.4 mmol, 2.0 equiv) in PhH (2.0 mL) at room temperature in nitrogen atmosphere under the irradiation of blue LED lamps for 20 hours. Column chromatography on silica gel (eluent: Petroleum ether) afforded the title product in 73% isolated yield (44.4 mg) as a white solid; R_f = 0.5 (eluent: Petroleum ether). **^{1}H NMR (400 MHz, $CDCl_3$)** δ 8.79 (d, J = 8.8 Hz, 2H), 8.03 (d, J = 9.2 Hz, 1H), 7.96 (t, J = 8.8 Hz, 2H), 7.87 – 7.83 (m, 2H), 7.72 (t, J = 7.6 Hz, 1H), 7.66 (t, J = 7.2 Hz, 1H), 7.51 – 7.46 (m, 6H), 7.16 (t, J = 7.6 Hz, 1H). **^{13}C NMR (101 MHz, $CDCl_3$)** δ 145.5, 138.4, 133.3, 131.4, 130.8, 130.5, 130.0, 129.9, 129.0, 128.9, 128.8, 128.3, 128.1, 128.0, 127.4, 126.9, 126.72, 126.66, 125.7, 124.5, 123.1, 121.2. The spectroscopic data match the reported literature[27].

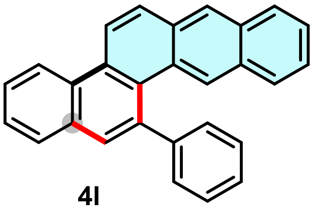

4l

13-Phenylbenzo[*c*]tetraphene (4l). Following the typical procedure described above, the reaction was carried out by the mixture of **1l** (76.1 mg, 0.2 mmol, 1.0 equiv), ethynylbenzene (61.2 mg, 0.6 mmol, 3.0 equiv), $Pd(OAc)_2$ (4.5 mg, 0.02 mmol, 10 mol%), DPEPhos (21.5 mg, 0.04 mmol, 20 mol%) and K_3PO_4 (84.8 mg, 0.4 mmol, 2.0 equiv) in PhH (2.0 mL) at room temperature in nitrogen atmosphere under the

irradiation of blue LED lamps for 20 hours. Column chromatography on silica gel (eluent: Petroleum ether/ Ethyl acetate = 100:1) afforded the title product in 67% isolated yield (47.5 mg) as a white solid; R_f = 0.6 (eluent: Petroleum ether/ Ethyl acetate = 80:1). **^{1}H NMR (600 MHz, $CDCl_3$)** δ 8.77 (d, *J* = 8.4 Hz, 1H), 8.69 (d, *J* = 9.3 Hz, 1H), 8.41 (s, 1H), 8.26 (s, 1H), 8.10 (d, *J* = 9.3 Hz, 1H), 8.01 – 7.96 (m, 2H), 7.93 (s, 1H), 7.72 (ddd, *J* = 8.4, 6.8, 1.4 Hz, 1H), 7.66 (ddd, *J* = 7.9, 6.8, 1.1 Hz, 1H), 7.53 (q, *J* = 3.0, 2.6 Hz, 5H), 7.47 (ddd, *J* = 8.1, 4.6, 3.1 Hz, 1H), 7.36 (dd, *J* = 3.5, 1.1 Hz, 2H). **^{13}C NMR (151 MHz, $CDCl_3$)** δ 145.5, 138.7, 131.71, 131.67, 131.0, 130.6, 130.4, 130.0, 129.6, 129.22, 129.16, 129.02, 128.97, 128.6, 128.5, 128.4, 127.4, 127.1, 127.0, 126.70, 126.69, 125.9, 125.8, 125.1, 123.2, 121.3. **HRMS (EI)** calcd for $C_{28}H_{18}$ $[M]^+$: 354.1403, found 354.1399.

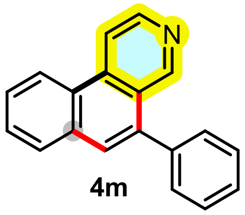

4m

5-Phenylbenzo[*f*]isoquinoline (4m). Following the typical procedure described above, the reaction was carried out by the mixture of **1m** (56.2 mg, 0.2 mmol, 1.0 equiv), ethynylbenzene (61.2 mg, 0.6 mmol, 3.0 equiv), $Pd(OAc)_2$ (4.5 mg, 0.02 mmol, 10 mol%), DPEPhos (21.5 mg, 0.04 mmol, 20 mol%) and K_3PO_4 (84.8 mg, 0.4 mmol, 2.0 equiv) in PhH (2.0 mL) at room temperature in nitrogen atmosphere under the irradiation of blue LED lamps for 20 hours. Column chromatography on silica gel (eluent: Petroleum ether/ Ethyl acetate = 20:1) afforded the title product in 75% isolated yield (38.8 mg) as a yellow solid; R_f = 0.5 (Petroleum ether/ Ethyl acetate = 10:1). **^{1}H NMR (400 MHz, $CDCl_3$)** δ 9.29 (s, 1H), 8.78 (d, *J* = 5.6 Hz, 1H), 8.72 – 8.70 (m, 1H), 8.49 (d, *J* = 5.6 Hz, 1H), 7.95 – 7.93 (m, 1H), 7.77 – 7.71 (m, 3H), 7.59 – 7.48 (m, 5H). **^{13}C NMR (101 MHz, $CDCl_3$)** δ 150.5, 145.0, 138.9, 137.7, 135.2, 133.0, 130.0, 128.9, 128.8, 128.7, 128.5, 128.0, 127.9, 127.1, 125.9, 123.0, 116.0. **HRMS (ESI)** calcd for $C_{19}H_{14}N$ $[M+H]^+$:256.1121, found 256.1123

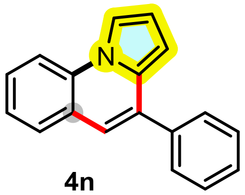

4n

4-Phenylpyrrolo[1,2-*a*]quinoline (4n). Following the typical procedure described above, the reaction was carried out by the mixture of **1n** (53.8 mg, 0.2 mmol, 1.0 equiv), ethynylbenzene (61.2 mg, 0.6 mmol, 3.0 equiv), $Pd(OAc)_2$ (4.5 mg, 0.02 mmol, 10 mol%), DPEPhos (21.5 mg, 0.04 mmol, 20 mol%) and K_3PO_4 (84.8 mg, 0.4 mmol, 2.0 equiv) in PhH (2.0 mL) at room temperature in nitrogen atmosphere under the irradiation of blue LED lamps for 20 hours. Column chromatography on silica gel (eluent: Petroleum

ether/ Ethyl acetate = 20:1) afforded the title product in 70% isolated yield (37.7 mg) as a yellow solid; R_f = 0.5 (Petroleum ether/ Ethyl acetate = 10:1). **^{1}H NMR (400 MHz, $CDCl_3$)** δ 7.96 – 7.91 (m, 2H), 7.77 – 7.75 (m, 2H), 7.70 (dd, *J* = 8.0, 1.6 Hz, 1H), 7.55 – 7.45 (m, 4H), 7.35 (td, *J* = 7.6, 1.2 Hz, 1H), 7.03 (s, 1H), 6.85 (d, *J* = 3.2 Hz, 1H), 6.67 (d, *J* = 4.0 Hz, 1H). **^{13}C NMR (101 MHz, $CDCl_3$)** δ 139.0, 132.7, 132.6, 130.8, 128.7, 128.5, 128.3, 128.0, 127.6, 124.2, 123.7, 118.0, 114.0, 112.7, 112.6, 103.3. The spectroscopic data match the reported literature[28].

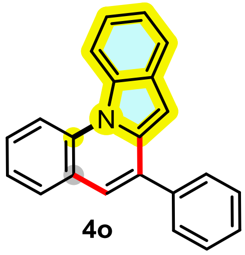

4o

6-Phenylindolo[1,2-*a*]quinoline (4o). Following the typical procedure described above, the reaction was carried out by the mixture of **1o** (63.8 mg, 0.2 mmol, 1.0 equiv), ethynylbenzene (61.2 mg, 0.6 mmol, 3.0 equiv), $Pd(OAc)_2$ (4.5 mg, 0.02 mmol, 10 mol%), DPEPhos (21.5 mg, 0.04 mmol, 20 mol%) and K_3PO_4 (84.8 mg, 0.4 mmol, 2.0 equiv) in PhH (2.0 mL) at room temperature in nitrogen atmosphere under the irradiation of blue LED lamps for 20 hours. Column chromatography on silica gel (eluent: Petroleum ether/ Ethyl acetate = 20:1) afforded the title product in 45% isolated yield (26.4 mg) as a yellow oil; R_f = 0.5 (Petroleum ether/ Ethyl acetate = 10:1). **^{1}H NMR (400 MHz, $CDCl_3$)** δ 8.62 (d, *J* = 8.4 Hz, 1H), 8.52 (d, *J* = 8.4 Hz, 1H), 7.84 (d, *J* = 7.6 Hz, 1H), 7.76 – 7.73 (m, 2H), 7.70 (dd, *J* = 7.6, 1.6 Hz, 1H), 7.64 – 7.60 (m, 1H), 7.54 (dd, *J* = 8.0, 6.4 Hz, 2H), 7.50 – 7.43 (m, 2H), 7.39 (t, *J* = 7.2 Hz, 1H), 7.34 (t, *J* = 7.6 Hz, 1H), 7.13 (s, 1H), 6.88 (s, 1H). **^{13}C NMR (101 MHz, $CDCl_3$)** δ 138.7, 136.5, 136.1, 133.3, 132.9, 130.1, 128.9, 128.6, 128.5, 128.4, 128.2, 124.5, 122.9, 122.8, 121.91, 121.87, 121.3, 115.2, 114.3, 97.7. The spectroscopic data match the reported literature[28].

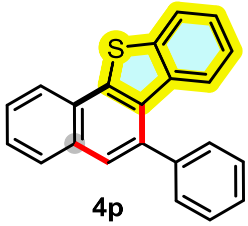

4p

6-Phenylbenzo[*b*]naphtho[2,1-*d*]thiophene (4p). Following the typical procedure described above, the reaction was carried out by the mixture of **1p** (67.2 mg, 0.2 mmol, 1.0 equiv), ethynylbenzene (61.2 mg, 0.6 mmol, 3.0 equiv), $Pd(OAc)_2$ (4.5 mg, 0.02 mmol, 10 mol%), DPEPhos (21.5 mg, 0.04 mmol, 20 mol%) and K_3PO_4 (84.8 mg, 0.4 mmol, 2.0 equiv) in PhH (2.0 mL) at room temperature in nitrogen atmosphere under the irradiation of blue LED lamps for 20 hours. Column chromatography on silica gel (eluent:

Petroleum ether/ Ethyl acetate = 50:1) afforded the title product in 38% isolated yield (23.6 mg) as a yellow solid; R_f = 0.6 (Petroleum ether/ Ethyl acetate = 20:1). **^{1}H NMR (400 MHz, CDCl$_3$)** δ 8.21 (d, *J* = 8.0 Hz, 1H), 7.95 (t, *J* = 7.2 Hz, 2H), 7.68 (s, 1H), 7.66 – 7.60 (m, 2H), 7.55 (s, 5H), 7.39 – 7.35 (m, 1H), 7.16 – 7.11 (m, 2H). **^{13}C NMR (101 MHz, CDCl$_3$)** δ 141.2, 139.1, 138.1, 137.3, 136.6, 131.3, 130.7, 129.3, 128.5, 128.1, 127.8, 126.8, 126.7, 126.6, 125.5, 124.7, 124.3, 123.9, 122.7. The spectroscopic data match the reported literature[23].

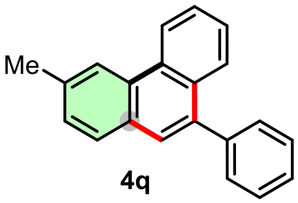

4q

3-Methyl-9-phenylphenanthrene (4q). Following the typical procedure described above, the reaction was carried out by the mixture of **1q** (58.8 mg, 0.2 mmol, 1.0 equiv), ethynylbenzene (61.2 mg, 0.6 mmol, 3.0 equiv), $Pd(OAc)_2$ (4.5 mg, 0.02 mmol, 10 mol%), DPEPhos (21.5 mg, 0.04 mmol, 20 mol%) and K_3PO_4 (84.8 mg, 0.4 mmol, 2.0 equiv) in PhH (2.0 mL) at room temperature in nitrogen atmosphere under the irradiation of blue LED lamps for 20 hours. Column chromatography on silica gel (eluent: Petroleum ether) afforded the title product in 68% isolated yield (36.5 mg) as a yellow oil; R_f = 0.6 (Petroleum ether). **^{1}H NMR (400 MHz, CDCl$_3$)** δ 8.80 (d, *J* = 8.4 Hz, 1H), 8.55 (s, 1H), 7.95 (d, *J* = 8.0 Hz, 1H), 7.82 (d, *J* = 8.0 Hz, 1H), 7.70 – 7.66 (m, 2H), 7.60 – 7.53 (m, 5H), 7.51 – 7.47 (m, 2H), 2.68 (s, 3H). **^{13}C NMR (101 MHz, CDCl$_3$)** δ 140.9, 137.7, 136.3, 131.2, 130.3, 130.1, 129.9, 129.4, 128.6, 128.5, 128.2, 127.3, 127.2, 126.8, 126.3, 122.8, 122.2, 22.2. The spectroscopic data match the reported literature[18].

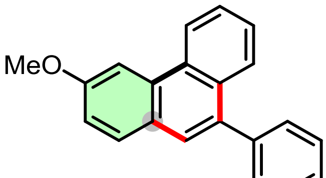

4r

3-Methoxy-9-phenylphenanthrene (4r). Following the typical procedure described above, the reaction was carried out by the mixture of **1r** (62.0 mg, 0.2 mmol, 1.0 equiv), ethynylbenzene (61.2 mg, 0.6 mmol, 3.0 equiv), $Pd(OAc)_2$ (4.5 mg, 0.02 mmol, 10 mol%), DPEPhos (21.5 mg, 0.04 mmol, 20 mol%) and K_3PO_4 (84.8 mg, 0.4 mmol, 2.0 equiv) in PhH (2.0 mL) at room temperature in nitrogen atmosphere under the irradiation of blue LED lamps for 20 hours. Column chromatography on silica gel (eluent: Petroleum ether) afforded the title product in 70% isolated yield (39.8 mg) as a colorless oil; R_f = 0.5 (Petroleum ether). **^{1}H NMR (400 MHz, CDCl$_3$)** δ 8.71 (d, *J* = 8.4 Hz, 1H), 8.11 – 8.10 (m, 1H), 7.94 (d, *J* = 8.0 Hz, 1H), 7.83 (d, *J* = 8.8 Hz, 1H), 7.68 – 7.65 (m, 2H), 7.57 – 7.50 (m, 5H), 7.46 (t, *J* = 6.8 Hz, 1H), 7.28 (dd, *J* = 8.8, 2.0 Hz, 1H), 4.06 (s, 3H). **^{13}C NMR (101 MHz, CDCl$_3$)** δ 160.0, 158.5, 140.9, 136.4, 131.4,

131.2, 130.12, 130.09, 130.0, 128.3, 127.2, 126.9, 126.5, 126.2, 126.0, 122.9, 117.0, 103.8, 55.5. The spectroscopic data match the reported literature[18].

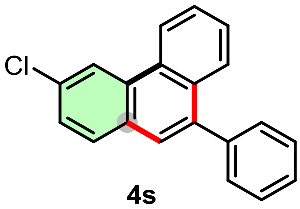

4s

3-Chloro-9-phenylphenanthrene (4s). Following the typical procedure described above, the reaction was carried out by the mixture of **1s** (62.9 mg, 0.2 mmol, 1.0 equiv), ethynylbenzene (61.2 mg, 0.6 mmol, 3.0 equiv), $Pd(OAc)_2$ (4.5 mg, 0.02 mmol, 10 mol%), DPEPhos (21.5 mg, 0.04 mmol, 20 mol%) and K_3PO_4 (84.8 mg, 0.4 mmol, 2.0 equiv) in PhH (2.0 mL) at room temperature in nitrogen atmosphere under the irradiation of blue LED lamps for 20 hours. Column chromatography on silica gel (eluent: Petroleum ether) afforded the title product in 68% isolated yield (39.3 mg) as a yellow oil; R_f = 0.6 (Petroleum ether). **^{1}H NMR (400 MHz, CDCl$_3$)** δ 8.68 (d, *J* = 6.8 Hz, 2H), 7.94 (d, *J* = 8.4 Hz, 1H), 7.82 (d, *J* = 8.4 Hz, 1H), 7.69 (t, *J* = 7.6 Hz, 1H), 7.65 (s, 1H), 7.59 – 7.44 (m, 7H).**^{13}C NMR (101 MHz, CDCl$_3$)** δ 140.4, 139.1, 132.5, 131.3, 131.0, 130.7, 130.0, 129.9, 129.8, 129.6, 128.3, 128.0, 127.5, 127.3, 127.1, 127.0, 126.74, 126.72, 122.9, 122.2. The spectroscopic data match the reported literature[18].

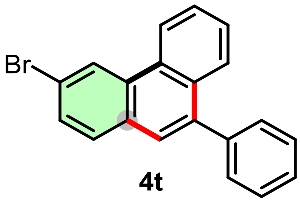

4t

3-Bromo-9-phenylphenanthrene (4t). Following the typical procedure described above, the reaction was carried out by the mixture of **1t** (71.8 mg, 0.2 mmol, 1.0 equiv), ethynylbenzene (61.2 mg, 0.6 mmol, 3.0 equiv), $Pd(OAc)_2$ (4.5 mg, 0.02 mmol, 10 mol%), DPEPhos (21.5 mg, 0.04 mmol, 20 mol%) and K_3PO_4 (84.8 mg, 0.4 mmol, 2.0 equiv) in PhH (2.0 mL) at room temperature in nitrogen atmosphere under the irradiation of blue LED lamps for 20 hours. Column chromatography on silica gel (eluent: Petroleum ether) afforded the title product in 55% isolated yield (36.7 mg) as a yellow oil; R_f = 0.6 (Petroleum ether). **^{1}H NMR (400 MHz, CDCl$_3$)** δ 8.86 (d, *J* = 1.6 Hz, 1H), 8.68 (dd, *J* = 8.4, 1.2 Hz, 1H), 7.93 (dd, *J* = 8.4, 1.2 Hz, 1H), 7.76 (d, *J* = 8.4 Hz, 1H), 7.72 – 7.66 (m, 2H), 7.63 (s, 1H), 7.59 – 7.47 (m, 6H).^{13}C NMR (101 MHz, CDCl$_3$) δ 140.4, 139.3, 131.3, 131.3, 130.1, 130.0, 130.0, 129.9, 129.5, 128.3, 127.5, 127.1, 127.0, 126.8, 126.8, 125.4, 122.9, 120.8. **HRMS (EI)** calcd for $C_{20}H_{13}Br$ $[M]^+$: 332.0195, found 332.0198.

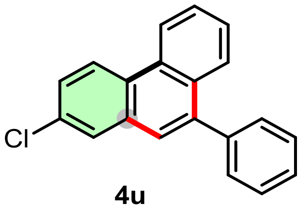

4u

2-Chloro-9-phenylphenanthrene (4u). Following the typical procedure described above, the reaction was carried out by the mixture of **1u** (62.9 mg, 0.2 mmol, 1.0 equiv), ethynylbenzene (61.2 mg, 0.6 mmol, 3.0 equiv), $Pd(OAc)_2$ (4.5 mg, 0.02 mmol, 10 mol%), DPEPhos (21.5 mg, 0.04 mmol, 20 mol%) and K_3PO_4 (84.8 mg, 0.4 mmol, 2.0 equiv) in PhH (2.0 mL) at room temperature in nitrogen atmosphere under the irradiation of blue LED lamps for 20 hours. Column chromatography on silica gel (eluent: Petroleum ether) afforded the title product in 71% isolated yield (41.0 mg) as a yellow oil; R_f = 0.6 (Petroleum ether). **^{1}H NMR (400 MHz, $CDCl_3$)** δ 8.71 (d, J = 8.4 Hz, 1H), 8.63 (d, J = 8.8 Hz, 1H), 7.93 (d, J = 8.4 Hz, 1H), 7.86 (d, J = 2.0 Hz, 1H), 7.69 (t, J = 7.6 Hz, 1H), 7.62 – 7.47 (m, 8H). **^{13}C NMR (101 MHz, $CDCl_3$)** δ 140.3, 140.1, 132.5, 131.0, 130.2, 129.9, 128.3, 128.2, 127.6, 127.5, 127.1, 127.0, 126.8, 126.7, 126.3, 124.2, 122.8. The spectroscopic data match the reported literature[29].

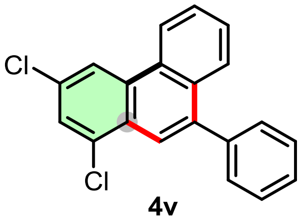

4v

1,3-Dichloro-9-phenylphenanthrene (4v). Following the typical procedure described above, the reaction was carried out by the mixture of **1v** (69.8 mg, 0.2 mmol, 1.0 equiv), ethynylbenzene (61.2 mg, 0.6 mmol, 3.0 equiv), $Pd(OAc)_2$ (4.5 mg, 0.02 mmol, 10 mol%), DPEPhos (21.5 mg, 0.04 mmol, 20 mol%) and K_3PO_4 (84.8 mg, 0.4 mmol, 2.0 equiv) in PhH (2.0 mL) at room temperature in nitrogen atmosphere under the irradiation of blue LED lamps for 20 hours. Column chromatography on silica gel (eluent: Petroleum ether) afforded the title product in 45% isolated yield (29.1 mg) as a colorless oil; R_f = 0.5 (eluent: Petroleum ether). **^{1}H NMR (400 MHz, $CDCl_3$)** δ 8.65 (d, J = 8.4 Hz, 1H), 8.61 (s, 1H), 8.09 (s, 1H), 7.94 (d, J = 8.0 Hz, 1H), 7.72 – 7.69 (m, 2H), 7.60 (t, J = 7.6 Hz, 1H), 7.55 – 7.49 (m, 5H). **^{13}C NMR (101 MHz, $CDCl_3$)** δ 140.3, 140.2, 133.5, 132.0, 131.7, 131.3, 130.0, 129.4, 128.4, 127.72, 127.68, 127.4, 127.3, 127.2, 123.2, 122.6, 121.3. **HRMS (EI)** calcd for $C_{20}H_{12}Cl_2$ $[M]^+$: 322.0311, found 322.0316.

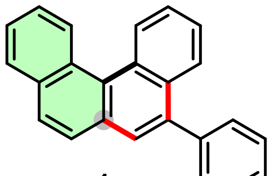

4w

5-Phenylbenzo[*c*]phenanthrene (4w). Following the typical procedure described above, the reaction was carried out by the mixture of **1w** (69.8 mg, 0.2 mmol, 1.0 equiv), ethynylbenzene (61.2 mg, 0.6 mmol, 3.0 equiv), $Pd(OAc)_2$ (4.5 mg, 0.02 mmol, 10 mol%), DPEPhos (21.5 mg, 0.04 mmol, 20 mol%) and K_3PO_4 (84.8 mg, 0.4 mmol, 2.0 equiv) in PhH (2.0 mL) at room temperature in nitrogen atmosphere under the irradiation of blue LED lamps for 20 hours. Column chromatography on silica gel (eluent: Petroleum ether) afforded the title product in 47% isolated yield (28.6 mg) as a white solid; R_f = 0.5 (eluent: Petroleum ether). **^{1}H NMR (400 MHz, $CDCl_3$)** δ 9.22 (d, *J* = 8.4 Hz, 1H), 9.18 (d, *J* = 8.4 Hz, 1H), 8.08 (ddd, *J* = 17.6, 8.0, 1.6 Hz, 2H), 7.94 (d, *J* = 8.4 Hz, 1H), 7.85 (d, *J* = 8.4 Hz, 1H), 7.82 (s, 1H), 7.73 (dtd, *J* = 8.0, 6.4, 1.6 Hz, 2H), 7.68 – 7.63 (m, 3H), 7.59 (td, *J* = 7.6, 7.2, 5.6 Hz, 3H), 7.53 – 7.49 (m, 1H). **^{13}C NMR (101 MHz, $CDCl_3$)** δ 140.5, 139.0, 133.5, 132.1, 130.7, 130.3, 130.04, 130.01, 128.6, 128.4, 128.2, 127.9, 127.7, 127.4, 126.9, 126.74, 126.66, 126.2, 125.91, 125.87, 125.8. The spectroscopic data match the reported literature[27].

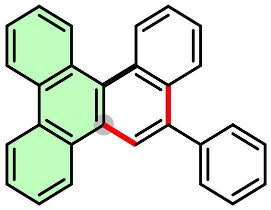

4x

6-Phenylbenzo[*g*]chrysene (4x). Following the typical procedure described above, the reaction was carried out by the mixture of **1x** (76.0 mg, 0.2 mmol, 1.0 equiv), ethynylbenzene (61.2 mg, 0.6 mmol, 3.0 equiv), $Pd(OAc)_2$ (4.5 mg, 0.02 mmol, 10 mol%), DPEPhos (21.5 mg, 0.04 mmol, 20 mol%) and K_3PO_4 (84.8 mg, 0.4 mmol, 2.0 equiv) in PhH (2.0 mL) at room temperature in nitrogen atmosphere under the irradiation of blue LED lamps for 20 hours. Column chromatography on silica gel eluent: Petroleum ether/ Ethyl acetate = 50:1) afforded the title product in 61% isolated yield (43.2 mg) as a yellow oil; R_f = 0.6 (Hexane: Ethyl acetate = 20:1). **^{1}H NMR (400 MHz, $CDCl_3$)** δ 9.01 (d, *J* = 8.5 Hz, 1H), 8.98 – 8.91 (m, 1H), 8.77 – 8.70 (m, 2H), 8.69 – 8.64 (m, 1H), 8.56 (s, 1H), 8.06 (d, *J* = 8.3 Hz, 1H), 7.73 – 7.53 (m, 11H). **^{13}C NMR (101 MHz, $CDCl_3$)** δ 140.9, 139.4, 132.1, 130.9, 130.6, 130.1, 130.1, 129.6, 129.4, 129.2, 128.7, 128.4, 127.5, 127.4, 127.3, 127.2, 126.7, 126.6, 126.4, 126.1, 125.9, 125.8, 123.7, 123.5, 123.1, 121.6. The spectroscopic data match the reported literature[30].

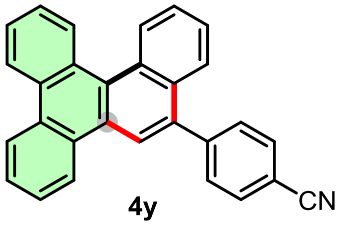

4y

4-(Benzo[*g*]chrysen-6-yl)benzonitrile (4y). Following the typical procedure described above, the reaction was carried out by the mixture of **1x** (76.0 mg, 0.2 mmol, 1.0 equiv), 4-ethynylbenzonitrile (76.3 mg, 0.6 mmol, 3.0 equiv), $Pd(OAc)_2$ (4.5 mg, 0.02 mmol, 10 mol%), DPEPhos (21.5 mg, 0.04 mmol, 20 mol%) and K_3PO_4 (84.8 mg, 0.4 mmol, 2.0 equiv) in PhH (2.0 mL) at room temperature in nitrogen atmosphere under the irradiation of blue LED lamps for 20 hours. Column chromatography on silica gel eluent: Petroleum ether/ Ethyl acetate = 10:1) afforded the title product in 64% isolated yield (48.6 mg) as a white solid; R_f = 0.6 (Hexane: Ethyl acetate = 6:1). **^{1}H NMR (400 MHz, $CDCl_3$)** δ 9.00 (d, *J* = 8.4 Hz, 1H), 8.91 (d, *J* = 8.0 Hz, 1H), 8.78 - 8.73 (m, 2H), 8.63 (d, *J* = 7.2 Hz, 1H), 8.52 (s, 1H), 7.92 - 7.87 (m, 3H), 7.80 (d, *J* = 8.0 Hz, 2H), 7.76 – 7.65 (m, 5H), 7.56 (t, *J* = 7.6 Hz, 1H). **^{13}C NMR (101 MHz, $CDCl_3$)** δ 145.8, 137.3, 132.3, 131.4, 131.1, 130.9, 130.7, 130.1, 129.5, 129.4, 129.01, 128.98, 127.6, 127.49, 127.46, 127.2, 127.0, 126.4, 126.3, 126.2, 125.6, 123.6, 123.2, 121.9, 119.0, 111.3. **HRMS (EI)** calcd for $C_{29}H_{17}N$ $[M]^+$: 379.1356, found 379.1356.

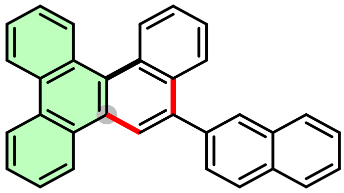

4z

6-(Naphthalen-2-yl)benzo[*g*]chrysene (4z). Following the typical procedure described above, the reaction was carried out by the mixture of **1x** (76.0 mg, 0.2 mmol, 1.0 equiv), 2-ethynylnaphthalene (91.3 mg, 0.6 mmol, 3.0 equiv), $Pd(OAc)_2$ (4.5 mg, 0.02 mmol, 10 mol%), DPEPhos (21.5 mg, 0.04 mmol, 20 mol%) and K_3PO_4 (84.8 mg, 0.4 mmol, 2.0 equiv) in PhH (2.0 mL) at room temperature in nitrogen atmosphere under the irradiation of blue LED lamps for 20 hours. Column chromatography on silica gel (eluent: Petroleum ether/ Ethyl acetate = 40:1) afforded the title product in 56% isolated yield (45.3 mg) as a white solid; R_f = 0.6 (Hexane: Ethyl acetate = 20:1). **^{1}H NMR (400 MHz, $CDCl_3$)** δ 9.03 (d, *J* = 8.4 Hz, 1H), 8.96 (dd, *J* = 7.6, 1.8 Hz, 1H), 8.79 – 8.71 (m, 2H), 8.71 – 8.65 (m, 2H), 8.16 (d, *J* = 1.6 Hz, 1H), 8.10 – 8.03 (m, 2H), 7.99 (td, *J* = 6.8, 3.6 Hz, 2H), 7.81 (dd, *J* = 8.4, 1.6 Hz, 1H), 7.73 – 7.64 (m, 5H), 7.61 – 7.58 (m, 2H), 7.55 – 7.51 (m, 1H). **^{13}C NMR (101 MHz, $CDCl_3$)** δ 139.3, 138.4, 133.5, 132.7, 132.3, 130.9, 130.7, 130.1, 129.6, 129.4, 129.2, 128.8, 128.4, 128.1, 127.8, 127.8, 127.4, 127.3, 127.2, 126.9, 126.6, 126.4, 126.4, 126.2, 126.0, 125.9, 123.7, 123.5, 123.1, 121.9. The spectroscopic data match the reported literature[30].

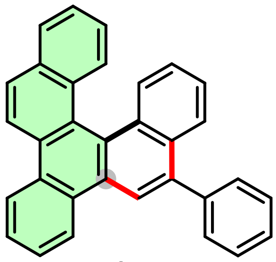

4aa

6-phenylnaphtho[1,2-g]chrysene (4aa). Following the typical procedure described above, the reaction was carried out by the mixture of **1y** (76.0 mg, 0.2 mmol, 1.0 equiv), ethynylbenzene (61.2 mg, 0.6 mmol, 3.0 equiv), $Pd(OAc)_2$ (4.5 mg, 0.02 mmol, 10 mol%), DPEPhos (21.5 mg, 0.04 mmol, 20 mol%) and K_3PO_4 (84.8 mg, 0.4 mmol, 2.0 equiv) in PhH (2.0 mL) at room temperature in nitrogen atmosphere under the irradiation of blue LED lamps for 20 hours. Column chromatography on silica gel (eluent: Petroleum ether/ Ethyl acetate = 70:1) afforded the title product in 61% isolated yield (49.5mg) as a white solid; R_f = 0.4 (Hexane: Ethyl acetate = 50:1). **^{1}H NMR (400 MHz, $CDCl_3$)** δ 8.74 – 8.71 (m, 3H), 8.68 (s, 1H), 8.21 (dd, *J* = 10.8, 9.2 Hz, 2H), 8.09 (d, *J* = 8.8 Hz, 1H), 8.00 (t, *J* = 8.0 Hz, 2H), 7.79 – 7.76 (m, 2H), 7.74 – 7.67 (m, 2H), 7.63 (t, *J* = 7.2 Hz, 2H), 7.57 – 7.50 (m, 2H), 7.43 (ddd, *J* = 8.4, 6.8, 1.2 Hz, 1H), 7.30 – 7.22 (m, 2H). **^{13}C NMR (151 MHz, $CDCl_3$)** δ 140.8, 139.6, 132.3, 131.7, 131.2, 130.8, 130.3, 130.2, 130.0, 129.9, 129.6, 129.2, 128.54, 128.47, 127.8, 127.6, 127.5, 127.3, 127.2, 126.0, 125.94, 125.90, 125.6, 125.3, 124.7, 124.5, 123.42, 123.37, 121.0, 120.5. **HRMS (EI)** calcd for $C_{32}H_{20}$ $[M]^+$: 404.1560, found 404.1562.

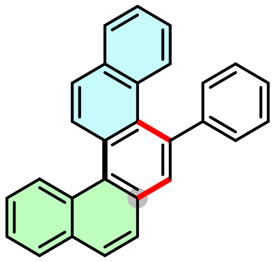

4ab

13-Phenylbenzo[*c*]chrysene (4ab). Following the typical procedure described above, the reaction was carried out by the mixture of **1z** (76.0 mg, 0.2 mmol, 1.0 equiv), ethynylbenzene (61.2 mg, 0.6 mmol, 3.0 equiv), $Pd(OAc)_2$ (4.5 mg, 0.02 mmol, 10 mol%), DPEPhos (21.5 mg, 0.04 mmol, 20 mol%) and K_3PO_4 (84.8 mg, 0.4 mmol, 2.0 equiv) in PhH (2.0 mL) at room temperature in nitrogen atmosphere under the irradiation of blue LED lamps for 20 hours. Column chromatography on silica gel (eluent: Petroleum ether/ Ethyl acetate = 50:1) afforded the title product in 58% isolated yield (41.2 mg) as a white solid; R_f = 0.5 (Hexane: Ethyl acetate = 30:1). **^{1}H NMR (600 MHz, $CDCl_3$)** δ 9.00 (t, *J* = 8.7 Hz, 2H), 8.04 (d, *J* = 7.6 Hz, 1H), 7.95 – 7.91 (m, 3H), 7.85 – 7.81 (m, 3H), 7.71 – 7.64 (m, 2H), 7.51 – 7.46 (m, 6H), 7.14 (ddd, *J* = 8.4, 6.9, 1.5 Hz, 1H). **^{13}C NMR (151 MHz, $CDCl_3$)** δ 145.0, 138.9, 133.6, 132.7, 130.4, 130.33,

130.29, 130.2, 129.9, 129.4, 129.0, 128.9, 128.8, 128.7, 128.6, 128.0, 127.5, 127.2, 127.1, 126.7, 126.3, 126.09, 126.07, 125.9, 124.3. **HRMS (EI)** calcd for $C_{28}H_{18}$ $[M]^+$: 354.1403, found 354.1400.

Unsuccessful substrate

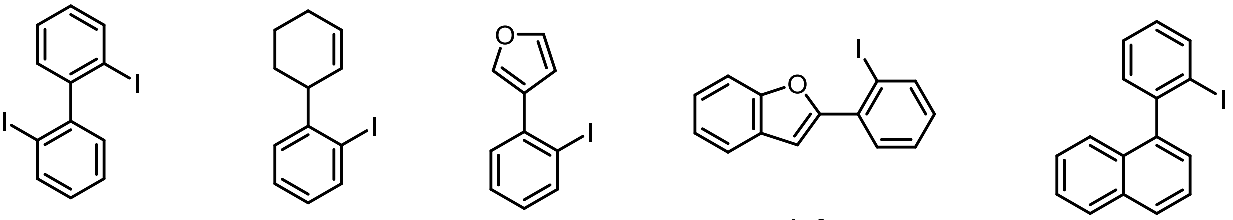

1ab 1ac 1ad 1af 1ag

For these substrates, no target product was obtained, with only starting materials remaining or the reaction resulting in a complex mixture.

Synthetic applications for the synthesis of PAHs

Synthesis of the PAH 6

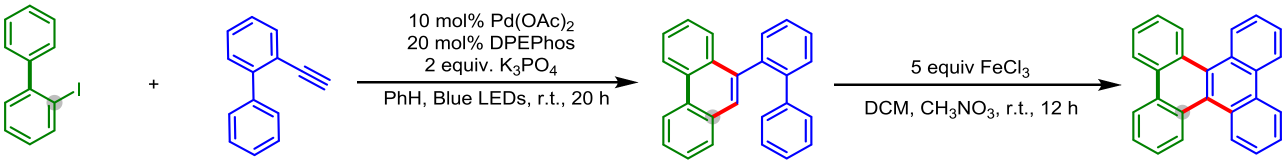

An oven-dried 20 mL reaction flask was charged with 2-iodo-1,1'-biphenyl **1a** (280.2 mg, 1.0 mmol, 1.0 equiv.), 2-ethynyl-1,1'-biphenyl (534.6 mg, 3 mmol, 3.0 equiv.), $Pd(OAc)_2$ (22.5 mg, 0.1 mmol, 10 mol%), DPEPhos (107.8 mg, 0.2 mmol, 20 mol%) and K_3PO_4 (424.4 mg, 2 mmol, 2.0 equiv.). It was directly transferred in a nitrogen-filled glovebox with caps. In the glovebox, 10 mL of degassed PhH were added to the vial. The vial was tightly sealed, transferred out of glovebox and stirred at room temperature under the irradiation of blue LED lamps for 20 hours. After completion of the reaction, the resulting mixture was diluted with acetone (20 mL), filtered (Celite), and concentrated under a reduced pressure. The residue was purified by column chromatography on silica gel (Petroleum ether) to afford **5** in 79% isolated yield (261 mg). **9-([1,1'-Biphenyl]-2-yl)phenanthrene** (**5**). **^{1}H NMR (600 MHz, $CDCl_3$)** δ 8.68 (t, J = 6.9 Hz, 2H), 7.79 – 7.77 (m, 1H), 7.70 (d, J = 8.4 Hz, 1H), 7.65 – 7.56 (m, 6H), 7.51 – 7.50 (m, 2H), 7.45 (dt, J = 8.1, 4.2 Hz, 1H), 7.19 – 7.18 (m, 2H), 7.07 – 6.94 (m, 3H). **^{13}C NMR (151 MHz, $CDCl_3$)** δ 142.0, 141.3, 138.9, 138.1, 131.7, 131.5, 131.4, 130.3, 130.2, 129.8, 128.9, 128.7, 128.5, 127.9, 127.6, 127.2, 127.1, 126.6, 126.4, 126.34, 126.27, 126.2, 122.6, 122.4. The spectroscopic data match the reported literature[31].

To a stirred solution of **5** (165.2 mg, 0.5 mmol) in CH_3NO_2(8 ml) and DCM (4 ml) at room temperature

was added $FeCl_3$ (405.5 mg, 2.5 mmol, 5 equiv), the mixture was stirred under nitrogen atmosphere at room temperature for 12 hour. After filtration and concentration, the crude was purified by column chromatography on silica gel (Hexane: Ethyl acetate = 20:1), yielding the desired compound **6** as a white soild (141.2 mg, 86% yield). **Dibenzo[*g,p*]chrysene** (**6**). **^{1}H NMR (600 MHz, $CDCl_3$)** δ 8.71 (ddd, *J* = 7.6, 5.5, 1.3 Hz, 8H), 7.69 (ddd, *J* = 8.1, 6.9, 1.3 Hz, 4H), 7.64 (ddd, *J* = 8.3, 6.9, 1.3 Hz, 4H). **^{13}C NMR (151 MHz, $CDCl_3$)** δ 130.8, 129.2, 128.9, 127.5, 126.5, 123.6. The spectroscopic data match the reported literature[32].

Synthesis of the PAH 9

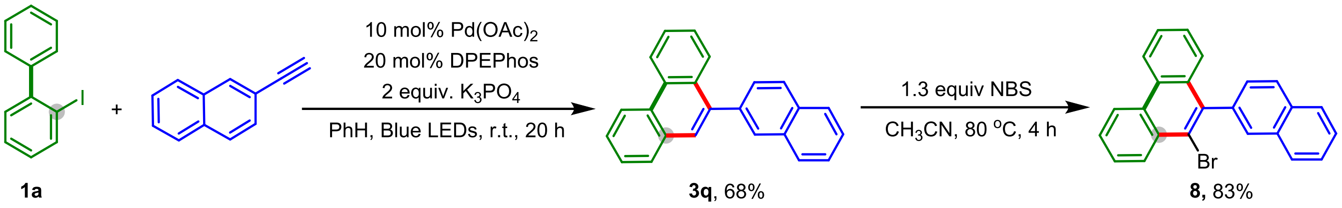

9-(Naphthalen-2-yl)phenanthrene (3q). An oven-dried 100 mL reaction flask was charged with 2-iodo-1,1'-biphenyl **1a** (840.3 mg, 3.0 mmol, 1.0 equiv.), 2-ethynylnaphthalene (1.37 g, 9 mmol, 3.0 equiv.), $Pd(OAc)_2$ (67.4 mg, 0.3 mmol, 10 mol%), DPEPhos (323.4 mg, 0.6 mmol, 20 mol%) and K_3PO_4 (1.27 g, 6 mmol, 2.0 equiv.). It was directly transferred in a nitrogen-filled glovebox with caps. In the glovebox, 30 mL of degassed PhH were added to the vial. The vial was tightly sealed, transferred out of glovebox and stirred at room temperature under the irradiation of blue LED lamps for 20 hours. After completion of the reaction, the resulting mixture was diluted with acetone (30 mL), filtered (Celite), and concentrated under a reduced pressure. The residue was purified by column chromatography on silica gel (Petroleum ether) to afford **3q** in 68% isolated yield (621.1 mg).

To a stirred solution of **3q** (608.8 mg, 2 mmol) in MeCN (10 mL) at room temperature was added NBS (462.8 mg, 2.6 mmol, 1.3 equiv), the mixture was stirred at room temperature for 4 hours. After filtration and concentration, the crude was purified by column chromatography on silica gel (eluent: Petroleum ether), yielding the desired compound **8** as a white solid (636.3 mg, 83% yield). **9-Bromo-10-(naphthalen-2-yl)phenanthrene (8). ^{1}H NMR (400 MHz, $CDCl_3$)** δ 8.77 (t, *J* = 7.3 Hz, 2H), 8.61 – 8.54 (m, 1H), 8.04 (d, *J* = 8.4 Hz, 1H), 7.99 (d, *J* = 7.5 Hz, 1H), 7.91 (d, *J* = 7.9 Hz, 1H), 7.85 (s, 1H), 7.75 (td, *J* = 7.7, 6.6, 3.6 Hz, 2H), 7.71 – 7.65 (m, 1H), 7.58 (pd, *J* = 6.9, 5.9, 3.3 Hz, 2H), 7.48 (dd, *J* = 8.3, 1.8 Hz, 1H), 7.43 (d, *J* = 5.5 Hz, 2H). **^{13}C NMR (151 MHz, $CDCl_3$)** δ 141.0, 139.6, 132.6, 130.9, 130.4, 130.0, 129.7, 128.9, 128.4, 127.9, 127.7, 127.7, 127.4, 127.0, 126.8, 123.6, 122.6. **HRMS (EI)** calcd for $C_{24}H_{25}Br$ $[M]^+$: 382.0352, found 382.0354.

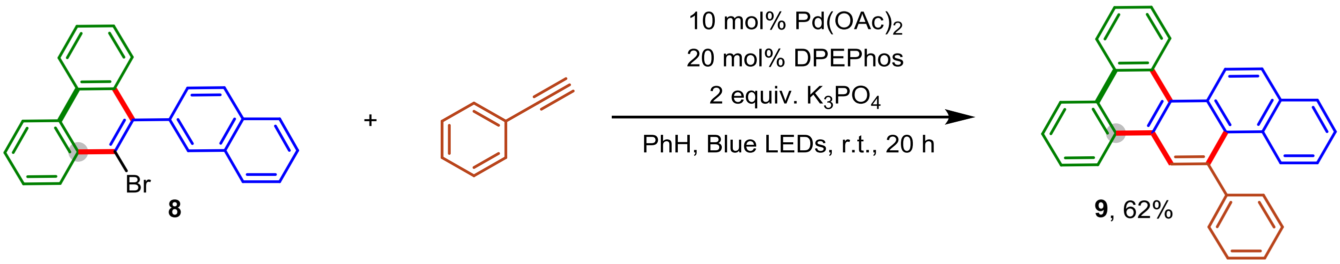

Following the typical procedure described above, the reaction was carried out by the mixture of 9-bromo-10-(naphthalen-2-yl)phenanthrene **8** (76.7 mg, 0.2 mol, 1.0 equiv.), ethynylbenzene (61.3 g, 0.6 mmol, 3.0 equiv.), Pd(OAc)$_2$ (4.5 mg, 0.02 mmol, 10 mol%), DPEPhos (21.6 mg, 0.6 mmol, 20 mol%) and K$_3$PO$_4$ (84.9 mg, 0.4 mmol, 2.0 equiv.) in PhH (2.0 mL) at room temperature in nitrogen atmosphere under the irradiation of blue LED lamps for 20 hours. Column chromatography on silica gel (eluent: Petroleum ether/ Ethyl acetate = 80:1) afforded the title product **9** in 62% isolated yield (50.2 mg) as a white solid; M.p. 219.3 – 221.1 ^{o}C. R$_f$ = 0.5 (Hexane: Ethyl acetate = 60:1). **15-Phenylbenzo[*f*]picene (9).** **^{1}H NMR (400 MHz, CDCl$_3$)** δ 8.83 (d, *J* = 9.2 Hz, 1H), 8.79 – 8.64 (m, 4H), 8.56 (s, 1H), 7.98 – 7.81 (m, 3H), 7.73 – 7.47 (m, 10H), 7.16 (t, *J* = 8.0 Hz, 1H). **^{13}C NMR (101 MHz, CDCl$_3$)** δ 145.4, 139.2, 132.8, 131.2, 130.4, 130.3, 130.1, 129.4, 129.3, 129.1, 129.0, 128.8, 127.7, 127.6, 127.5, 127.4, 127.4, 127.2, 126.9, 126.2, 126.1, 126.0, 125.0, 124.6, 123.7, 123.6, 123.1. **HRMS (EI)** calcd for $C_{32}H_{20}$ [M]$^+$: 404.1560, found 404.1562.

Synthesis of the PAH 12

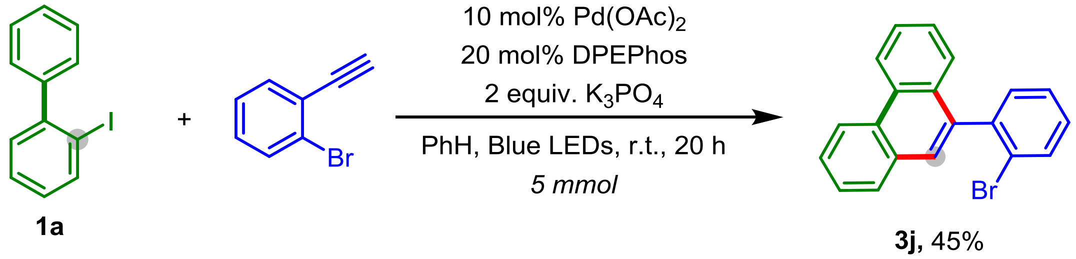

An oven-dried 100 mL reaction flask was charged with 2-iodo-1,1'-biphenyl **1a** (1.40 g, 5.0 mmol, 1.0 equiv.), 1-bromo-2-ethynylbenzene **2j** (2.72 g, 15 mmol, 3.0 equiv.), Pd(OAc)$_2$ (112.3 mg, 0.5 mmol, 10 mol%), DPEPhos (538.9 mg, 1 mmol, 20 mol%) and K$_3$PO$_4$ (2.12 g, 10 mmol, 2.0 equiv.). It was directly transferred in a nitrogen-filled glovebox with caps. In the glovebox, 50 mL of degassed PhH were added to the vial. The vial was tightly sealed, transferred out of glovebox and stirred at room temperature under the irradiation of blue LED lamps for 20 hours. After completion of the reaction, the resulting mixture was diluted with acetone (100 mL), filtered (Celite), and concentrated under a reduced pressure. The residue was purified by column chromatography on silica gel (Petroleum ether) to afford **3j** in 45% isolated yield (749.9 mg).

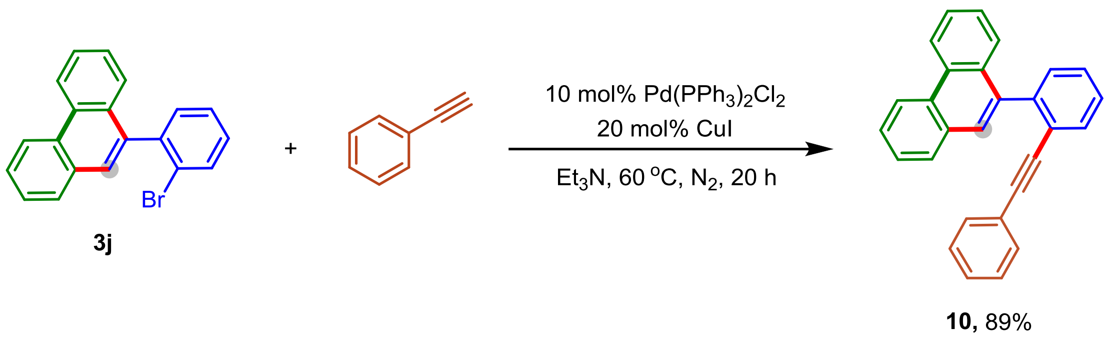

An oven-dried 20 mL reaction flask was charged with 9-(2-bromophenyl)phenanthrene **3j** (666.6 mg, 2.0 mmol, 1.0 equiv.), ethynylbenzene **2a** (245.3 mg, 2.4 mmol, 1.2 equiv.), $Pd(PPh_3)_2Cl_2$ (140.4 mg, 0.2 mmol, 10 mol%) and CuI (76.2 mg, 0.4 mmol, 20 mol%). It was directly transferred in a nitrogen-filled glovebox with caps. In the glovebox, 8 mL of degassed Et_3N were added to the vial. The vial was tightly sealed, transferred out of glovebox and stirred at 60 °C for 20 hours. After completion of the reaction, the resulting mixture was diluted with acetone (30 mL), filtered (Celite), and concentrated under a reduced pressure. The residue was purified by column chromatography on silica gel (eluent: Petroleum ether/ Ethyl acetate = 50:1) to afford **10** in 89% isolated yield (631.1 mg). **^{1}H NMR (400 MHz, $CDCl_3$)** δ 8.81 (dd, J = 16.7, 8.3 Hz, 2H), 7.93 (d, J = 7.8 Hz, 1H), 7.80 (d, J = 7.4 Hz, 2H), 7.77 – 7.62 (m, 4H), 7.58 – 7.47 (m, 4H), 7.14 – 7.02 (m, 3H), 6.76 – 6.69 (m, 2H). **^{13}C NMR (101 MHz, $CDCl_3$)** δ 143.1, 137.5, 132.0, 131.5, 131.1, 131.0, 130.6, 130.3, 130.1, 128.7, 128.3, 128.1, 127.9, 127.8, 127.5, 127.4, 126.7, 126.6, 126.4, 126.3, 123.8, 122.9, 122.7, 122.6, 93.2, 88.9. The spectroscopic data match the reported literature[33].

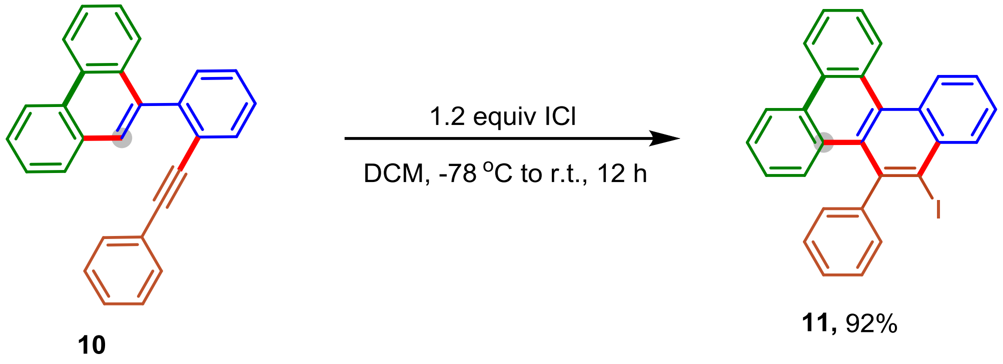

To a stirred solution of **10** (354.5 mg, 1 mmol) in DCM (10 mL) at -78 °C was added ICl (210.6 mg, 1.3 mmol, 1.3 equiv, ICl dissolved in 3ml DCM), the mixture was stirred at room temperature for 12 hours. After filtration and concentration, the crude was purified by column chromatography on silica gel (eluent: Petroleum ether/ Ethyl acetate = 50:1), yielding the desired compound **11** as a yellow solid (442.1 mg, 92% yield).

^{1}H NMR (400 MHz, $CDCl_3$) δ 8.70 – 8.60 (m, 3H), 8.54 (d, J = 8.2 Hz, 2H), 7.66 (ddq, J = 20.3, 8.6, 7.1 Hz, 4H), 7.54 (d, J = 8.6 Hz, 1H), 7.48 – 7.42 (m, 4H), 7.42 – 7.36 (m, 2H), 7.09 (ddd, J = 8.4, 6.9, 1.3 Hz, 1H). **^{13}C NMR (101 MHz, $CDCl_3$)** δ 147.7, 142.7, 134.7, 133.6, 131.8, 131.2, 130.6, 130.6, 129.9, 129.7, 129.4, 129.2, 129.0, 128.6, 128.4, 128.3, 127.9, 127.6, 127.2, 126.6, 126.5, 126.4, 125.7, 123.5,

123.0, 107.3. **HRMS (EI)** calcd for $C_{28}H_{17}I$ $[M]^+$: 480.0369, found 480.0372.

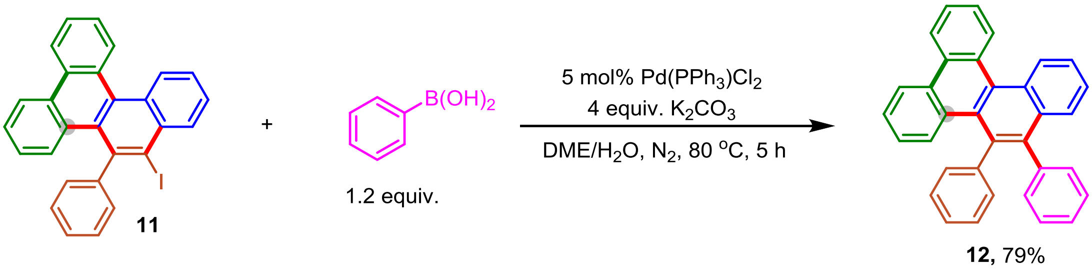

An oven-dried 4 mL reaction flask was charged with 6-iodo-5-phenylbenzo[*g*]chrysene **11** (96.1 mg, 0.2 mmol, 1.0 equiv.), phenylboronic acid (29.3 mg, 0.24 mmol, 1.2 equiv.), $Pd(PPh_3)_2Cl_2$ (7.1 mg, 0.01 mmol, 5 mol%) and K_2CO_3 (110.4 mg, 0.8 mmol, 4 equiv.). It was directly transferred in a nitrogen-filled glovebox with caps. In the glovebox, 2 mL of degassed DME/H_2O (1:1) were added to the vial. The vial was tightly sealed, transferred out of glovebox and stirred at 80 °C for 5 hours. After completion of the reaction, the resulting mixture was diluted with acetone (3 mL), filtered (Celite), and concentrated under a reduced pressure. The residue was purified by column chromatography on silica gel (eluent: Petroleum ether/ Ethyl acetate = 50:1) to afford **12** in 79% isolated yield (68.1 mg). **5,6-diphenylbenzo[g]chrysene (12).** ^{1}**H NMR (400 MHz, CDCl$_3$)** δ 9.02 – 8.95 (m, 2H), 8.84 (dd, *J* = 7.6, 1.7 Hz, 1H), 8.74 (d, *J* = 8.2 Hz, 1H), 7.94 (dd, *J* = 8.3, 1.4 Hz, 1H), 7.85 (ddd, *J* = 9.1, 7.6, 1.6 Hz, 2H), 7.78 – 7.75 (m, 2H), 7.71 – 7.58 (m, 4H), 7.55 – 7.40 (m, 5H), 7.37 – 7.32 (m, 2H), 7.26 – 7.20 (m, 2H). ^{13}**C NMR (101 MHz, CDCl$_3$)** δ 142.7, 139.4, 138.3, 135.8, 132.3, 131.9, 131.6, 131.4, 131.2, 130.8, 130.6, 129.8, 129.5, 129.3, 128.8, 128.5, 128.4, 127.8, 127.6, 127.3, 127.2, 126.9, 126.8, 126.5, 126.4, 126.2, 126.1, 126.1, 125.5, 125.5, 123.6, 123.1. The spectroscopic data match the reported literature[34].

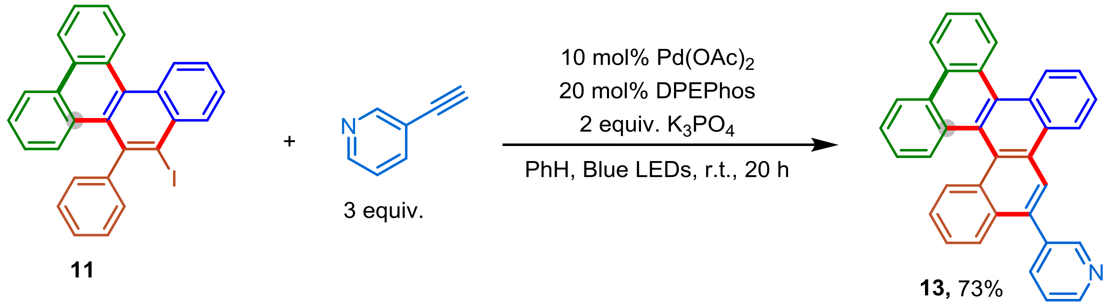

Following the typical procedure described above, the reaction was carried out by the mixture of 6-iodo-5-phenylbenzo[*g*]chrysene **11** (96.1 mg, 0.2 mmol, 1.0 equiv.), 3-ethynylpyridine (61.9 mg, 0.6 mmol, 3.0 equiv.), $Pd(OAc)_2$ (4.5 mg, 0.02 mmol, 10 mol%), DPEPhos (21.6 mg, 0.6 mmol, 20 mol%) and K_3PO_4 (84.9 mg, 0.4 mmol, 2.0 equiv.) in PhH (2.0 mL) at room temperature in nitrogen atmosphere under the irradiation of blue LED lamps for 20 hours. Column chromatography on silica gel (eluent: Petroleum ether/ Ethyl acetate = 5:1) afforded the title product **13** in 73% isolated yield (66.5 mg) as a yellow solid; R_f = 0.6 (Hexane: Ethyl acetate = 4:1). **3-(tribenzo[c,g,p]chrysen-10-yl)pyridine (13).** ^{1}**H**

NMR (400 MHz, $CDCl_3$) δ 9.03 (s, 1H), 8.86 – 8.72 (m, 5H), 8.70 (s, 1H), 8.61 – 8.57 (m, 1H), 8.36 (d, *J* = 8.6 Hz, 1H), 8.07 (t, *J* = 8.3 Hz, 2H), 7.89 (dd, *J* = 8.3, 1.3 Hz, 1H), 7.77 – 7.61 (m, 4H), 7.55 (ddd, *J* = 8.2, 6.0, 1.3 Hz, 2H), 7.44 (ddd, *J* = 8.2, 6.8, 1.2 Hz, 1H), 7.29 (ddd, *J* = 8.2, 6.9, 1.2 Hz, 1H), 7.26 – 7.21 (m, 1H). **^{13}C NMR (101 MHz, $CDCl_3$)** δ 150.4, 148.5, 137.7, 136.7, 135.7, 131.3, 131.3, 131.1, 131.0, 130.4, 130.3, 130.2, 129.8, 129.6, 129.0, 129.0, 128.9, 128.6, 128.2, 127.1, 126.7, 126.7, 126.6, 126.5, 126.4, 126.1, 125.8, 125.5, 125.3, 125.0, 123.6, 123.6, 123.4, 123.3, 122.3. **HRMS (EI)** calcd for $C_{35}H_{21}N$ $[M]^+$: 455.1669, found 455.1665.

Synthesis of 9-iodophenanthrene 16.

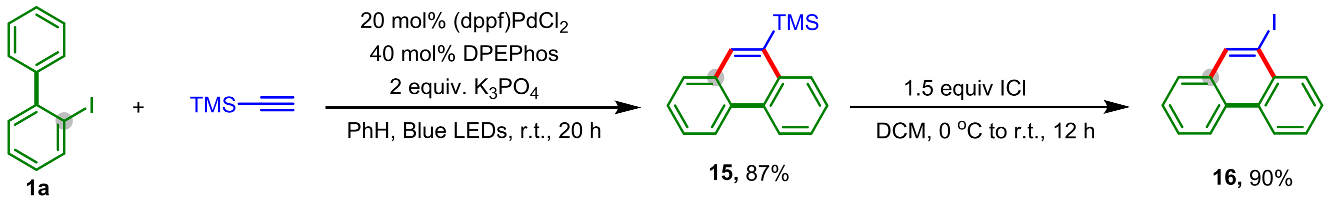

Following the typical procedure described above, the reaction was carried out by the mixture of 2-iodo-1,1'-biphenyl **1a** (140.1 mg, 0.5 mmol, 1.0 equiv.), ethynyltrimethylsilane (147.4 g, 1.5 mmol, 3.0 equiv.), $Pd(OAc)_2$ (11.3 mg, 0.05 mmol, 10 mol%), DPEPhos (53.9 mg, 0.1 mmol, 20 mol%) and K_3PO_4 (212.1 mg, 1 mmol, 2.0 equiv.) in PhH (5.0 mL) at room temperature in nitrogen atmosphere under the irradiation of blue LED lamps for 20 hours. Column chromatography on silica gel (eluent: Petroleum ether) afforded the title product **15** (contains a minor quantity of unreacted **1a**) in 79% isolated yield (222.8 mg) as a yellow oil. The reactants and products in this reaction exhibit similar polarities, which complicates their separation. To achieve a clean spectrum, the reaction conditions were modified by increasing the catalyst and ligand loadings, as well as by using 10 equivalents of **2a**.The reaction was carried out by the mixture of 2-iodo-1,1'-biphenyl **1a** (280.1 mg, 1.0 mmol, 1.0 equiv.), ethynyltrimethylsilane (982.2 mg, 10 mmol, 10.0 equiv.), $dppfPdCl_2$ (146.4 mg, 0.2 mmol, 20 mol%), DPEPhos (215.6 mg, 0.4 mmol, 40 mol%) and K_3PO_4 (424.4 mg, 2 mmol, 2.0 equiv.) in PhH (10.0 mL) at room temperature in nitrogen atmosphere under the irradiation of blue LED lamps for 20 hours. Column chromatography on silica gel (eluent: Petroleum ether) afforded the title product **15** in 87% isolated yield (217.9 mg) as a colorless liquid; R_f = 0.7 (Petroleum ether). **Trimethyl(phenanthren-9-yl)silane (15). ^1H NMR (400 MHz, $CDCl_3$)** δ 8.78 – 8.76 (m, 1H), 8.69 (d, *J* = 8.4 Hz, 1H), 8.19 – 8.17 (m, 1H), 7.98 (s, 1H), 7.91 (d, *J* = 7.6 Hz, 1H), 7.65 (dq, *J* = 15.2, 7.2 Hz, 4H), 0.55 (s, 9H). **^{13}C NMR (101 MHz, $CDCl_3$)** δ 136.6, 135.2, 134.9, 131.1, 130.9, 130.0, 128.9, 128.8, 127.1, 126.6, 126.2, 125.9, 123.3, 122.4, 0.2. The spectroscopic data match the reported literature[35].

To a stirred solution of **15** (125.2 mg, 0.5 mmol) in DCM (5 mL) at 0 °C was added ICl (122.3 mg, 0.75 mmol, 1.5 equiv, ICl dissolved in 2ml DCM), the mixture was stirred at room temperature for 12 hours. After filtration and concentration, the crude was purified by column chromatography on silica gel (eluent: Petroleum ether), yielding the desired compound **16** as a yellow oil (136.9 mg, 90% yield). **9-Iodophenanthrene (16).** **^{1}H NMR (400 MHz, $CDCl_3$)** δ 8.77 – 8.59 (m, 2H), 8.44 (s, 1H), 8.28 – 8.15 (m, 1H), 7.77 (dd, J = 8.0, 1.5 Hz, 1H), 7.73 – 7.65 (m, 3H), 7.60 (ddd, J = 8.1, 7.0, 1.2 Hz, 1H). **^{13}C NMR (151 MHz, $CDCl_3$)** δ 138.6, 133.2, 133.0, 132.1, 130.6, 130.3, 127.8, 127.6, 127.5, 127.3, 127.1, 122.8, 122.7, 98.7. The spectroscopic data match the reported literature[36].

Synthesis of unbranched PAHs 17-23.

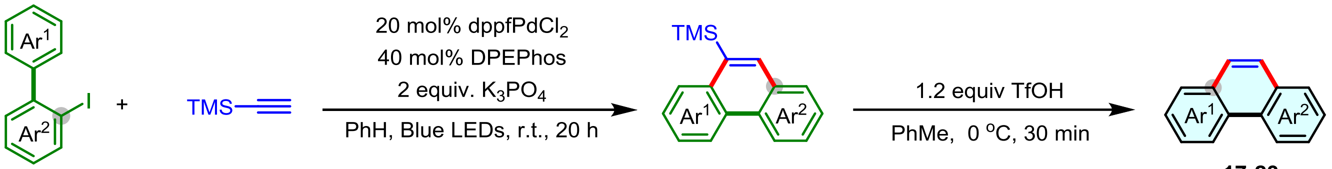

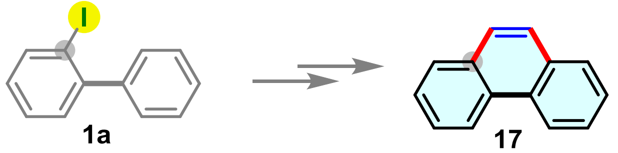

Following the typical procedure described above, the reaction was carried out by the mixture of 2-iodo-1,1'-biphenyl **1a** (280.1 mg, 1.0 mmol, 1.0 equiv.), ethynyltrimethylsilane (982.2 mg, 10 mmol, 10.0 equiv.), $dppfPdCl_2$ (146.4 mg, 0.2 mmol, 20 mol%), DPEPhos (215.6 mg, 0.4 mmol, 40 mol%) and K_3PO_4 (424.4 mg, 2 mmol, 2.0 equiv.) in PhH (10.0 mL) at room temperature in nitrogen atmosphere under the irradiation of blue LED lamps for 20 hours. Column chromatography on silica gel (eluent: Petroleum ether) afforded the title product **15** in 87% isolated yield (217.9 mg) as a colorless liquid. The obtained product is directly put into the next reaction. To a stirred solution of trimethyl(phenanthren-9-yl)silane (165.2 mg, 0.5 mmol) in PhMe (5 mL) at 0 °C was added TfOH (90.1 mg, 0.6 mmol, 1.2 equiv), the mixture was stirred at 0 °C for 30 minutes. After filtration and concentration, the crude was purified by column chromatography on silica gel (eluent: Petroleum ether), yielding the desired compound **17** as a white solid (98.1 mg, 76% yield). The overall yield of the two-step reaction is 66%. **Phenanthrene (17).** **^{1}H NMR (400 MHz, $CDCl_3$)** δ 8.71 (d, J = 8.0 Hz, 2H), 7.91 (d, J = 8.0 Hz, 2H), 7.76 (s, 2H), 7.67 (t, J = 7.2 Hz, 2H), 7.61 (t, J = 7.2 Hz, 2H). **^{13}C NMR (101 MHz, $CDCl_3$)** δ 132.0, 130.3, 128.5, 126.9, 126.5, 122.6. The spectroscopic data match the reported literature[37].

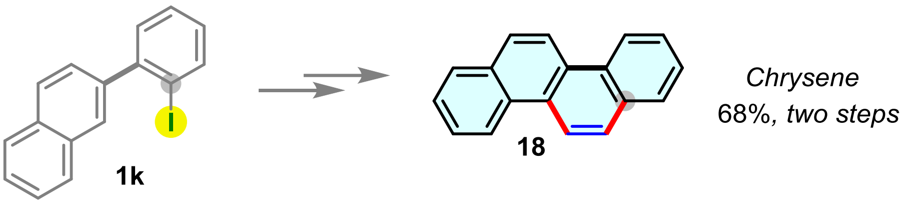

Following the typical procedure described above, the reaction was carried out by the mixture of 2-(2-iodophenyl)naphthalene **1k** (165.1 mg, 0.5 mmol, 1.0 equiv.), ethynyltrimethylsilane (491.1 mg, 5 mmol, 10.0 equiv.), dppfPdCl_2 (71.2 mg, 0.1 mmol, 20 mol%), DPEPhos (107.8 mg, 0.2 mmol, 40 mol%) and K_3PO_4 (212.2 mg, 1 mmol, 2.0 equiv.) in PhH (5.0 mL) at room temperature in nitrogen atmosphere under the irradiation of blue LED lamps for 20 hours. Column chromatography on silica gel (eluent: Petroleum ether) afforded the title product benzo[*c*]phenanthren-5-yltrimethylsilane in 76% isolated yield (114.2 mg) as a colorless liquid. The obtained product is directly put into the next reaction. To a stirred solution of chrysen-5-yltrimethylsilane (60.1 mg, 0.2 mmol) in PhMe (2 mL) at 0 °C was added TfOH (36.1 mg, 0.24 mmol, 1.2 equiv), the mixture was stirred at 0 °C for 30 minutes. After filtration and concentration, the crude was purified by column chromatography on silica gel (eluent: Petroleum ether), yielding the desired compound **18** as a white solid (40.6 mg, 89% yield). The overall yield of the two-step reaction is 68%. **Chrysene (18).** **^{1}H NMR (600 MHz, $CDCl_3$)** δ 8.80 (d, *J* = 8.4 Hz, 2H), 8.74 (d, *J* = 9.0 Hz, 2H), 8.01 (t, *J* = 9.3 Hz, 4H), 7.72 (ddd, *J* = 8.4, 6.6, 1.5 Hz, 2H), 7.65 (t, *J* = 7.2 Hz, 2H). **^{13}C NMR (151 MHz, $CDCl_3$)** δ 132.2, 130.6, 128.6, 128.2, 127.3, 126.7, 126.4, 123.1, 121.2. The spectroscopic data match the reported literature[37].

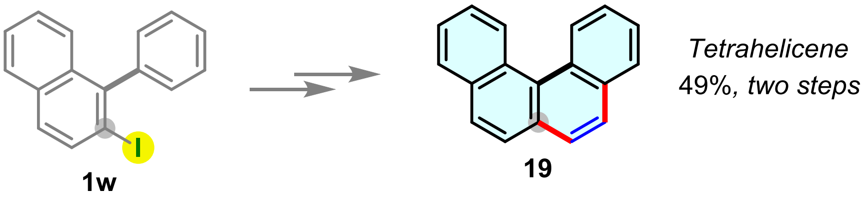

Following the typical procedure described above, the reaction was carried out by the mixture of 2-iodo-1-phenylnaphthalene **1w** (165.1 mg, 0.5 mmol, 1.0 equiv.), ethynyltrimethylsilane (491.1 mg, 5 mmol, 10.0 equiv.), dppfPdCl_2 (71.2 mg, 0.1 mmol, 20 mol%), DPEPhos (107.8 mg, 0.2 mmol, 40 mol%) and K_3PO_4 (212.2 mg, 1 mmol, 2.0 equiv.) in PhH (5.0 mL) at room temperature in nitrogen atmosphere under the irradiation of blue LED lamps for 20 hours. Column chromatography on silica gel (eluent: Petroleum ether) afforded the title product benzo[*c*]phenanthren-5-yltrimethylsilane in 59% isolated yield (87.2 mg) as a colorless liquid. The obtained product is directly put into the next reaction. To a stirred solution of benzo[*c*]phenanthren-5-yltrimethylsilane (60.1 mg, 0.2 mmol) in PhMe (2 mL) at 0 °C was added TfOH (36.1 mg, 0.24 mmol, 1.2 equiv), the mixture was stirred at 0 °C for 30 minutes. After filtration and concentration, the crude was purified by column chromatography on silica gel (eluent: Petroleum ether), yielding the desired compound **19** as a white solid (37.5 mg, 82% yield). The overall yield of the two-

step reaction is 49%. **Tetrahelicene (19).** **^{1}H NMR (600 MHz, $CDCl_3$)** δ 9.16 (d, *J* = 8.4 Hz, 2H), 8.04 (dd, *J* = 7.8, 1.5 Hz, 2H), 7.92 (d, *J* = 8.4 Hz, 2H), 7.84 (d, *J* = 8.4 Hz, 2H), 7.70 (ddd, *J* = 8.4, 6.9, 1.5 Hz, 2H), 7.64 (ddd, *J* = 7.8, 6.6, 1.2 Hz, 2H). **^{13}C NMR (151 MHz, $CDCl_3$)** δ 133.5, 131.0, 130.3, 128.6, 127.9, 127.5, 127.4, 126.9, 126.2, 125.9. The spectroscopic data match the reported literature[37].

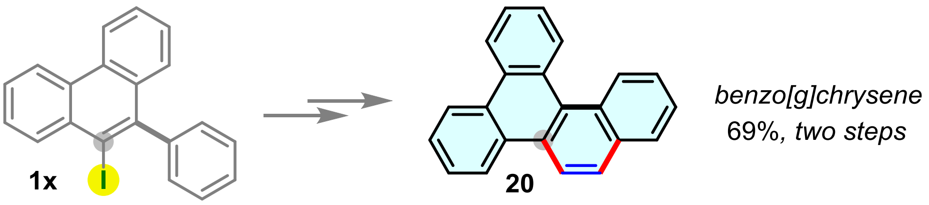

Following the typical procedure described above, the reaction was carried out by the mixture of 9-iodo-10-phenylphenanthrene **1x** (190.2 mg, 0.5 mmol, 1.0 equiv.), ethynyltrimethylsilane (491.1 mg, 5 mmol, 10.0 equiv.), dppfPdCl_2 (71.2 mg, 0.1 mmol, 20 mol%), DPEPhos (107.8 mg, 0.2 mmol, 40 mol%) and K_3PO_4 (212.2 mg, 1 mmol, 2.0 equiv.) in PhH (5.0 mL) at room temperature in nitrogen atmosphere under the irradiation of blue LED lamps for 20 hours. Column chromatography on silica gel (eluent: Hexane: Ethyl acetate = 80:1) afforded the title product benzo[*g*]chrysen-6-yltrimethylsilane in 77% isolated yield (134.9 mg) as a colorless liquid. The obtained product is directly put into the next reaction. To a stirred solution of benzo[*g*]chrysen-6-yltrimethylsilane (70.1 mg, 0.2 mmol) in PhMe (2 mL) at 0 °C was added TfOH (36.1 mg, 0.24 mmol, 1.2 equiv), the mixture was stirred at 0 °C for 30 minutes. After filtration and concentration, the crude was purified by column chromatography on silica gel (eluent: Hexane: Ethyl acetate = 50:1), yielding the desired compound **20** as a white solid (50.1 mg, 90% yield). The overall yield of the two-step reaction is 69%. **Benzo[*g*]chrysene (20).** **^{1}H NMR (600 MHz, $CDCl_3$)** δ 8.96 (d, *J* = 8.4 Hz, 1H), 8.92 (d, *J* = 8.1 Hz, 1H), 8.75 (d, *J* = 8.1 Hz, 1H), 8.73 – 8.71 (m, 1H), 8.67 – 8.66 (m, 1H), 8.62 (d, *J* = 8.7 Hz, 1H), 8.02 (t, *J* = 8.1 Hz, 2H), 7.73 – 7.69 (m, 3H), 7.63 (dq, *J* = 20.4, 7.5, 7.2 Hz, 3H). **^{13}C NMR (151 MHz, $CDCl_3$)** δ 133.5, 130.8, 130.2, 130.0, 129.8, 129.44, 129.38, 128.4, 128.1, 128.0, 127.7, 127.31, 127.28, 127.1, 126.6, 126.1, 126.0, 125.8, 123.7, 123.5, 123.1, 120.7. The spectroscopic data match the reported literature[38].

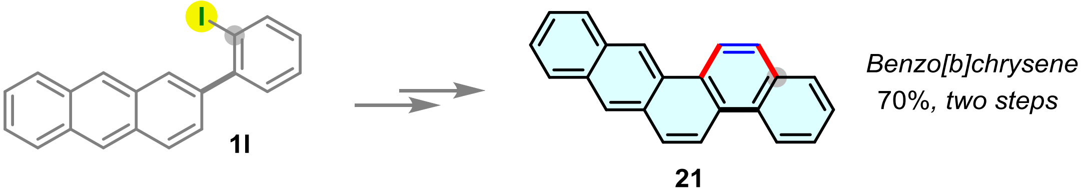

Following the typical procedure described above, the reaction was carried out by the mixture of 2-(2-iodophenyl)anthracene **1l** (190.2 mg, 0.5 mmol, 1.0 equiv.), ethynyltrimethylsilane (491.1 mg, 5 mmol, 10.0 equiv.), dppfPdCl_2 (71.2 mg, 0.1 mmol, 20 mol%), DPEPhos (107.8 mg, 0.2 mmol, 40 mol%) and K_3PO_4 (212.2 mg, 1 mmol, 2.0 equiv.) in PhH (5.0 mL) at room temperature in nitrogen atmosphere under

the irradiation of blue LED lamps for 20 hours. Column chromatography on silica gel (eluent: Hexane: Ethyl acetate = 100:1) afforded the title product benzo[*c*]tetraphen-13-yltrimethylsilane in 83% isolated yield (145.5 mg) as a colorless liquid. The obtained product is directly put into the next reaction. To a stirred solution of benzo[*c*]tetraphen-13-yltrimethylsilane (70.1 mg, 0.2 mmol) in PhMe (2 mL) at 0 °C was added TfOH (36.1 mg, 0.24 mmol, 1.2 equiv), the mixture was stirred at 0 °C for 30 minutes. After filtration and concentration, the crude was purified by column chromatography on silica gel (eluent: Hexane: Ethyl acetate = 80:1), yielding the desired compound **21** as a white solid (46.8 mg, 84% yield, M.p. 207.7 – 209.4 °C.). The overall yield of the two-step reaction is 70%. **Benzo[*b*]tetraphene (21).** **^{1}H NMR (400 MHz, CDCl$_3$)** δ 9.32 (s, 1H), 8.93 (d, *J* = 8.8 Hz, 1H), 8.78 (d, *J* = 8.4 Hz, 1H), 8.65 (d, *J* = 9.2 Hz, 1H), 8.52 (s, 1H), 8.20 – 8.17 (m, 1H), 8.13 – 8.09 (m, 3H), 8.03 (d, *J* = 8.0 Hz, 1H), 7.75 – 7.71 (m, 1H), 7.65 (t, *J* = 7.6 Hz, 1H), 7.57 (dt, *J* = 9.6, 5.2 Hz, 2H). **^{13}C NMR (151 MHz, CDCl$_3$)** δ 132.5, 132.0, 131.8, 130.59, 130.58, 129.2, 128.6, 128.3, 127.81, 127.78, 127.5, 126.8, 126.7, 126.3, 125.8, 125.7, 123.2, 122.2, 121.4, 121.3. **HRMS (EI)** calcd for $C_{22}H_{14}$ [M]$^+$: 278.1090, found 278.1088.

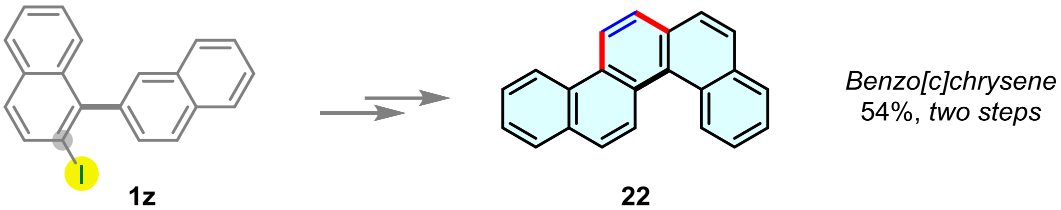

1z **22**

Following the typical procedure described above, the reaction was carried out by the mixture of 9-iodo-10-(naphthalen-2-yl)phenanthrene **1z** (190.2 mg, 0.5 mmol, 1.0 equiv.), ethynyltrimethylsilane (491.1 mg, 5 mmol, 10.0 equiv.), dppfPdCl$_2$ (71.2 mg, 0.1 mmol, 20 mol%), DPEPhos (107.8 mg, 0.2 mmol, 40 mol%) and K_3PO_4 (212.2 mg, 1 mmol, 2.0 equiv.) in PhH (5.0 mL) at room temperature in nitrogen atmosphere under the irradiation of blue LED lamps for 20 hours. Column chromatography on silica gel (eluent: Hexane: Ethyl acetate = 70:1) afforded the title product trimethyl(naphtho[1,2-*g*]chrysen-6-yl)silane in 67% isolated yield (117.4 mg) as a white solid. The obtained product is directly put into the next reaction. To a stirred solution of trimethyl(naphtho[1,2-*g*]chrysen-6-yl)silane (70.1 mg, 0.2 mmol) in PhMe (2 mL) at 0 °C was added TfOH (36.1 mg, 0.24 mmol, 1.2 equiv), the mixture was stirred at 0 °C for 30 minutes. After filtration and concentration, the crude was purified by column chromatography on silica gel (eluent: Hexane: Ethyl acetate = 60:1), yielding the desired compound **22** as a white solid (45.1 mg, 81% yield). The overall yield of the two-step reaction is 54%. **Benzo[*c*]chrysene (22).** **^{1}H NMR (400 MHz, CDCl$_3$)** δ 9.07 (t, *J* = 8.8 Hz, 2H), 8.84 (d, *J* = 8.8 Hz, 2H), 8.07 – 8.01 (m, 3H), 7.97 (d, *J* = 9.1 Hz, 1H), 7.93 – 7.87 (m, 2H), 7.76 – 7.64 (m, 4H). **^{13}C NMR (101 MHz, CDCl$_3$)** δ 133.59, 131.57, 131.0, 130.5, 130.2, 130.0, 128.50, 128.47, 128.1, 128.0, 127.5, 127.0, 126.63, 126.61, 126.56, 126.4, 126.2, 126.1, 125.9, 123.3, 121.8. The spectroscopic data match the reported literature[40].

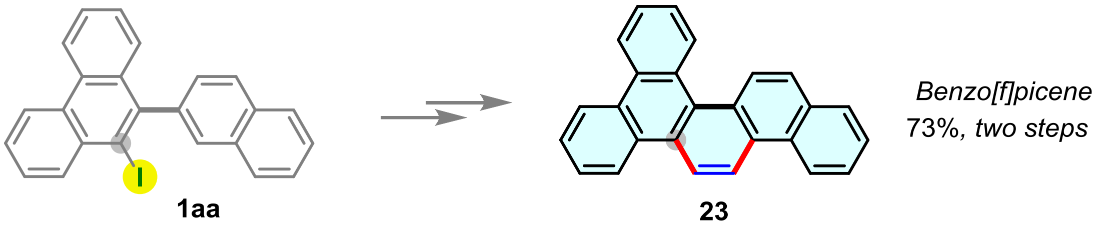

Following the typical procedure described above, the reaction was carried out by the mixture of 9-iodo-10-(naphthalen-2-yl)phenanthrene **1aa** (215.2 mg, 0.5 mmol, 1.0 equiv.), ethynyltrimethylsilane (491.1 mg, 5 mmol, 10.0 equiv.), dppfPdCl_2 (71.2 mg, 0.1 mmol, 20 mol%), DPEPhos (107.8 mg, 0.2 mmol, 40 mol%) and K_3PO_4 (212.2 mg, 1 mmol, 2.0 equiv.) in PhH (5.0 mL) at room temperature in nitrogen atmosphere under the irradiation of blue LED lamps for 20 hours. Column chromatography on silica gel (eluent: Hexane: Ethyl acetate = 80:1) afforded the title product benzo[*f*]picen-15-yltrimethylsilane in 82% isolated yield (164.3 mg) as a colorless liquid. The obtained product is directly put into the next reaction. To a stirred solution of benzo[*f*]picen-15-yltrimethylsilane (80.1 mg, 0.2 mmol) in PhMe (2 mL) at 0 °C was added TfOH (36.1 mg, 0.24 mmol, 1.2 equiv), the mixture was stirred at 0 °C for 30 minutes. After filtration and concentration, the crude was purified by column chromatography on silica gel (eluent: Hexane: Ethyl acetate = 50:1), yielding the desired compound **23** as a white solid (58.5 mg, 89% yield). The overall yield of the two-step reaction is 73%. **Benzo[*f*]picene (23).** **^{1}H NMR (400 MHz, $CDCl_3$)** δ 8.82 (td, *J* = 10.0, 9.6, 6.4 Hz, 4H), 8.75 – 8.65 (m, 4H), 7.98 (d, *J* = 7. 8 Hz, 1H), 7.87 (d, *J* = 9.2 Hz, 1H), 7.70 (td, *J* = 8.0, 6.0 Hz, 5H), 7.65 – 7.61 (m, 1H). **^{13}C NMR (101 MHz, $CDCl_3$)** δ 131.5, 131.0, 130.20, 130.18, 130.00, 129.98, 129.6, 129.4, 128.5, 128.4, 128.1, 128.0, 127.4, 127.2, 126.81, 126.78, 126.7, 126.6, 126.0, 125.9, 123.6, 123.5, 123.14, 123.08, 122.1, 120.9. The spectroscopic data match the reported literature[39].

Reaction of aryl diiodobenzene 24 and subsequent transformation of its product.

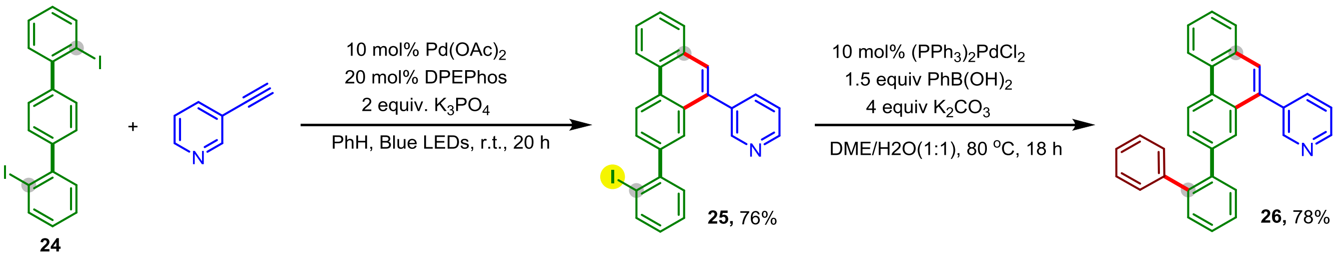

Following the typical procedure described above, the reaction was carried out by the mixture of **24** (482.1 mg, 1 mmol, 1.0 equiv), 3-ethynylpyridine (309.6 mg, 3 mmol, 3.0 equiv), $Pd(OAc)_2$ (22.4 mg, 0.1 mmol, 10 mol%), DPEPhos (107.8 mg, 0.2 mmol, 20 mol%) and K_3PO_4 (424.4 mg, 2 mmol, 2.0 equiv) in PhH (10.0 mL) at room temperature in nitrogen atmosphere under the irradiation of blue LED lamps for 20

hours. Column chromatography on silica gel (eluent: Petroleum ether/ Ethyl acetate = 7:1) afforded the title product in 76% isolated yield (347.5 mg) as a yellow solid; R_f = 0.5 (eluent: Petroleum ether/ Ethyl acetate = 5:1). **3-(7-(2-Iodophenyl)phenanthren-9-yl)pyridine(25).** ^{1}**H NMR (400 MHz, CDCl$_3$)** δ 8.92 – 8.82 (m, 2H), 8.77 (d, *J* = 8.4 Hz, 1H), 8.70 (d, *J* = 4.8 Hz, 1H), 7.95 (dd, *J* = 11.6, 8.0 Hz, 3H), 7.80 (s, 1H), 7.75 – 7.64 (m, 4H), 7.46 – 7.32 (m, 3H), 7.05 (t, *J* = 7.2 Hz, 1H). ^{13}**C NMR (101 MHz, CDCl$_3$)** δ 150.5, 148.7, 146.0, 142.5, 139.5, 137.5, 136.2, 135.0, 131.3, 130.3, 130.2, 130.0, 129.9, 129.9, 129.0, 128.8, 128.2, 128.0, 127.2, 127.2, 126.8, 123.2, 122.9, 122.7, 98.6. **HRMS (ESI)** calcd for $C_{25}H_{17}IN$ $[M+H]^+$: 458.0400, found 458.0396.

To a stirred solution of **25** (228.7 mg, 0.5 mmol) in DME and H_2O (1:1, 5 ml) at room temperature was added $Pd(PPh_3)_2Cl_2$(35.1 mg, 0.05 mmol, 10 mol%), K_2CO_3(276 mg, 2 mmol, 4 equiv) and phenylboronic acid (91.4 mg, 0.75 mmol, 1.5 equiv)) the mixture was stirred at 80 °C in nitrogen atmosphere for 18 hour. After filtration and concentration, the crude was purified by column chromatography on silica gel (Hexane: Ethyl acetate = 3:1), yielding the desired compound **26** as a yellow oil (158.9 mg, 78% yield). **3-(7-([1,1'-Biphenyl]-2-yl)phenanthren-9-yl)pyridine(26).** ^{1}**H NMR (400 MHz,CDCl$_3$)** δ 8.72 (dd, *J* = 16.0, 8.4 Hz, 2H), 8.68 – 8.62 (m, 1H), 8.58 – 8.51 (m, 1H), 7.88 (dd, *J* = 7.8, 1.6 Hz, 1H), 7.69 (dd, *J* = 8.6, 1.6 Hz, 2H), 7.65 – 7.60 (m, 2H), 7.50 – 7.43 (m, 5H), 7.33 – 7.29 (m, 1H), 7.21 (d, *J* = 2.8 Hz, 4H), 7.14 – 7.09 (m, 2H). ^{13}**C NMR (101 MHz, CDCl$_3$)** δ 150.0, 148.2, 141.4, 140.7, 140.0, 139.7, 137.5, 134.8, 131.2, 130.8, 130.7, 129.9, 129.1, 128.7, 128.4, 128.3, 128.0, 128.0, 127.7, 127.6, 127.1, 126.9, 126.4, 123.1, 123.0, 122.5. **HRMS (ESI)** calcd for $C_{31}H_{22}N$ $[M+H]^+$: 408.1747, found 408.1745.

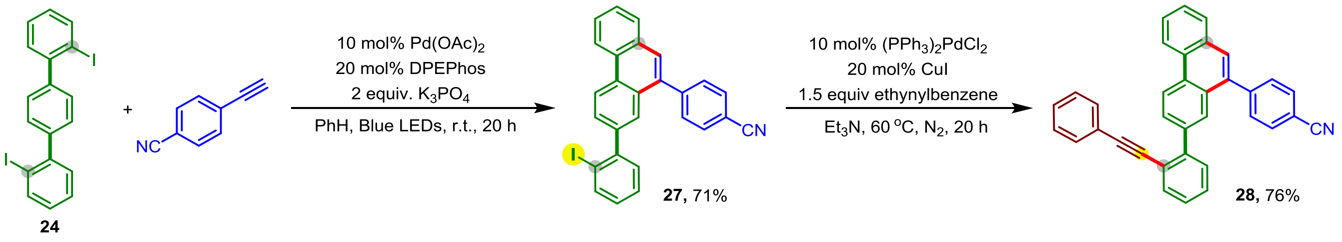

Following the typical procedure described above, the reaction was carried out by the mixture of **24** (482.1 mg, 1 mmol, 1.0 equiv), 4-ethynylbenzonitrile (381.5 mg, 3 mmol, 3.0 equiv), $Pd(OAc)_2$ (22.4 mg, 0.1 mmol, 10 mol%), DPEPhos (107.8 mg, 0.2 mmol, 20 mol%) and K_3PO_4 (424.4 mg, 2 mmol, 2.0 equiv) in PhH (10.0 mL) at room temperature in nitrogen atmosphere under the irradiation of blue LED lamps for 20 hours. Column chromatography on silica gel (eluent: Petroleum ether/ Ethyl acetate = 20:1) afforded the title product in 71% isolated yield (341.8 mg) as a yellow solid; R_f = 0.6 (eluent: Petroleum ether/ Ethyl acetate = 20:1). **4-(7-(2-Iodophenyl)phenanthren-9-yl)benzonitrile (27).** ^{1}**H NMR (400 MHz, CDCl$_3$)** δ 8.85 (d, *J* = 8.5 Hz, 1H), 8.77 (d, *J* = 8.2 Hz, 1H), 7.95 (dd, *J* = 18.3, 7.9 Hz, 2H), 7.83 – 7.78 (m, 2H), 7.77 – 7.64 (m, 7H), 7.40 (t, *J* = 7.4 Hz, 1H), 7.33 (dd, *J* = 7.6, 1.8 Hz, 1H), 7.06 (td, *J* = 7.7, 1.8 Hz, 1H). ^{13}**C NMR (101 MHz, CDCl$_3$** δ 146.0, 145.4, 142.5, 139.6, 136.9, 132.2, 131.2, 130.9,

130.2, 130.0, 129.9, 129.6, 129.0, 128.9, 128.4, 128.2, 128.1, 127.4, 127.3, 126.8, 123.0, 122.7, 118.9, 111.3, 98.6. **HRMS (EI)** calcd for $C_{27}H_{16}IN$ $[M]^+$:481.0322, found 481.0321.

To a stirred solution of **27** (240.7 mg, 0.5 mmol) in Et_3N (5 ml) at room temperature was added $Pd(PPh_3)_2Cl_2$(35.1 mg, 0.05 mmol, 10 mol%), CuI (19.1 mg, 0.1 mmol, 20 mol%) and ethynylbenzene (75.6 mg, 0.75 mmol, 1.5 equiv)) the mixture was stirred at 60 °C in nitrogen atmosphere for 20 hour. After filtration and concentration, the crude was purified by column chromatography on silica gel (Hexane: Ethyl acetate = 10:1), yielding the desired compound **28** as a yellow oil (172.4 mg, 76% yield). **4-(7-(2-(Phenylethynyl)phenyl)phenanthren-9-yl)benzonitrile** (**28**). **^{1}H NMR (400 MHz, $CDCl_3$)** δ 8.90 (d, *J* = 8.4 Hz, 1H), 8.81 (d, *J* = 8.4 Hz, 1H), 8.13 (d, *J* = 2.0 Hz, 1H), 8.02 (dd, *J* = 8.4, 2.0 Hz, 1H), 7.94 (dd, *J* = 8.0, 1.6 Hz, 1H), 7.76 (ddd, *J* = 8.4, 7.2, 1.6 Hz, 1H), 7.72 – 7.65 (m, 3H), 7.61 – 7.53 (m, 4H), 7.49 – 7.35 (m, 3H), 7.28 (m, 5H). **HRMS (ESI)** calcd for $C_{35}H_{22}N$ $[M+H]^+$: 456.1747, found 456.1749.

Experimental Procedures for the Mechanistic Studies

Control experiments:

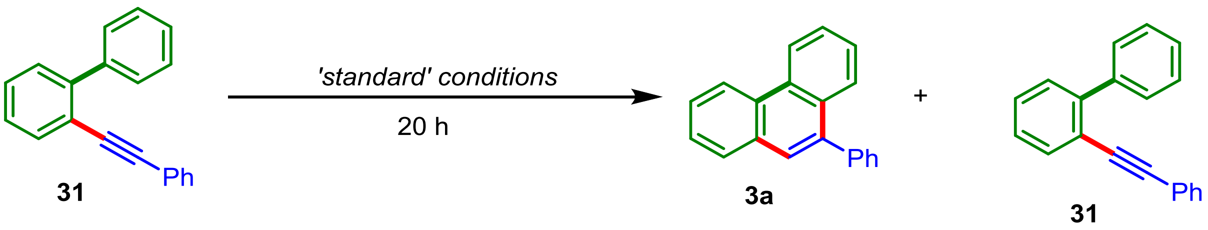

'standard' conditions **3a,** 0% and **31,** 98%
carried out in 80 °C **3a,** 0% and **31,** 97%

(1) The reaction was carried out by the mixture of **31** (50.9 mg, 0.2 mmol, 1.0 equiv), $Pd(OAc)_2$ (4.5 mg, 0.02 mmol, 10 mol%), DPEPhos (21.5 mg, 0.04 mmol, 20 mol%) and K_3PO_4 (84.8 mg, 0.4 mmol, 2.0 equiv) in PhH (2.0 mL) at room temperature in nitrogen atmosphere under the irradiation of blue LED lamps for 20 hours. No target product **3a** is generated, and the materials **31** are recovered at 98%.

The reaction was carried out by the mixture of **31** (50.9 mg, 0.2 mmol, 1.0 equiv), $Pd(OAc)_2$ (4.5 mg, 0.02 mmol, 10 mol%), DPEPhos (21.5 mg, 0.04 mmol, 20 mol%) and K_3PO_4 (84.8 mg, 0.4 mmol, 2.0 equiv) in PhH (2.0 mL) at 80 °C in nitrogen atmosphere for 20 hours. No target product **3a** is generated, and the materials **31** are recovered at 97%.

The Radical Trapping Experiment with TEMPO

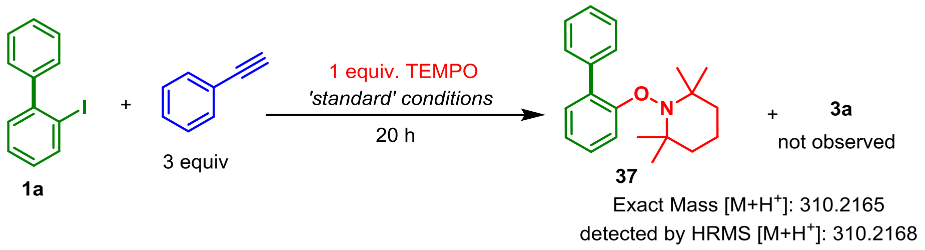

Following the typical procedure described above, the reaction was carried out by the mixture of **1a** (56.0 mg, 0.2 mmol, 1.0 equiv), ethynylbenzene (61.3 mg, 0.6 mmol, 3.0 equiv), TEMPO(31.3 mg, 0.2 mmol, 1.0 equiv), $Pd(OAc)_2$ (4.5 mg, 0.02 mmol, 10 mol%), DPEPhos (21.5 mg, 0.04 mmol, 20 mol%) and K_3PO_4 (84.8 mg, 0.4 mmol, 2.0 equiv) in PhH (2.0 mL) at room temperature in nitrogen atmosphere under the irradiation of blue LED lamps for 20 hours. The intermediate generated from substrate **1a** was captured by TEMPO, and the resulting product **37** ($[M+H]^+$) was detected by high-resolution mass spectrometry. **HRMS (ESI)** calcd for $C_{21}H_{28}NO$ $[M+H]^+$: 310.2165, found 310.2168. **(Supplementary Fig. 5)**

Supplementary Fig. 5. HRMS of the radical trapping experiment with TEMPO

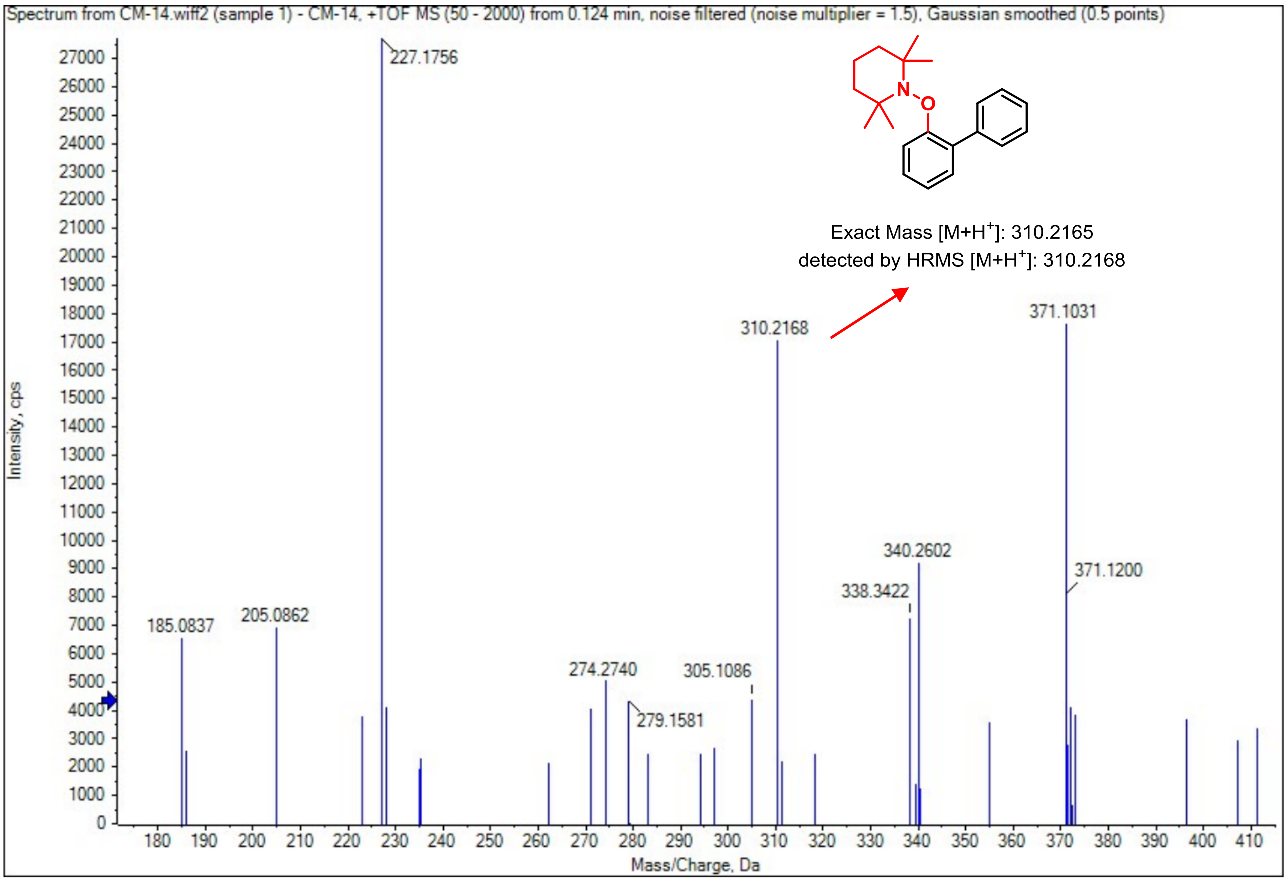

Radical clock experiments

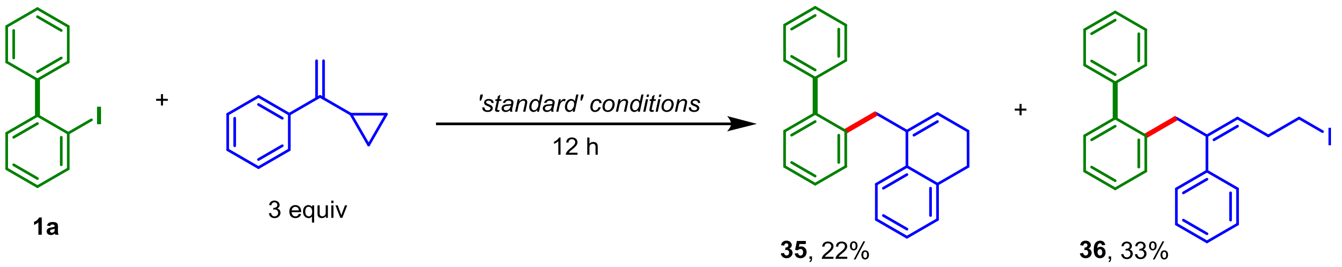

Following the typical procedure described above, the reaction was carried out by the mixture of **1a** (56.0 mg, 0.2 mmol, 1.0 equiv), (1-cyclopropylvinyl)benzene (86.5 mg, 0.6 mmol, 3.0 equiv), $Pd(OAc)_2$ (4.5 mg, 0.02 mmol, 10 mol%), DPEPhos (21.5 mg, 0.04 mmol, 20 mol%) and K_3PO_4 (84.8 mg, 0.4 mmol, 2.0 equiv) in PhH (2.0 mL) at room temperature in nitrogen atmosphere under the irradiation of blue LED lamps for 12 hours. Column chromatography on silica gel eluent: Petroleum ether) afforded the title product **35** in 22% isolated yield (13.1 mg) and product **36** in 33% isolated yield (28.1 mg) as acolorless oil; R_f = 0.6 (Petroleum ether). **4-([1,1'-biphenyl]-2-ylmethyl)-1,2-dihydronaphthalene.** **^{1}H NMR (600 MHz, $CDCl_3$)** δ 7.40 (d, *J* = 4.3 Hz, 4H), 7.35 (t, *J* = 4.3 Hz, 1H), 7.32 – 7.29 (m, 4H), 7.15 – 7.06 (m, 3H), 6.99 (d, *J* = 7.4 Hz, 1H), 5.69 (t, *J* = 4.7 Hz, 1H), 3.72 (d, *J* = 2.2 Hz, 2H), 2.78 (t, *J* = 8.0 Hz, 2H), 2.30 (m, 2H). **^{13}C NMR (151 MHz, $CDCl_3$)** δ 142.1, 141.7, 137.0, 136.6, 135.9, 134.9, 130.0, 129.8, 129.0, 128.1, 127.5, 127.4, 127.3, 126.9, 126.6, 126.2, 126.1, 122.9, 36.4, 28.3, 23.2. **HRMS (EI)** calcd for $C_{23}H_{20}$ $[M]^+$: 296.1560, found 296.1559. **2-(5-iodo-2-phenylpent-2-en-1-yl)-1,1'-biphenyl.** **^{1}H NMR (400 MHz, $CDCl_3$)** δ 7.39 – 7.33 (m, 2H), 7.30 (dd, *J* = 7.0, 1.7 Hz, 1H), 7.27 – 7.23 (m, 2H), 7.14 (d, *J* = 4.8 Hz, 8H), 5.77 (t, *J* = 7.1 Hz, 1H), 3.69 (s, 2H), 3.05 (td, *J* = 7.3, 1.7 Hz, 2H), 2.60 (qd, *J* = 7.2, 1.7 Hz, 2H). **^{13}C NMR (101 MHz, $CDCl_3$)** δ 142.2, 141.9, 141.6, 140.2, 136.4, 129.8, 129.2, 129.0, 128.2, 128.2, 128.1, 127.5, 127.0, 127.0, 126.3, 126.0, 33.7, 32.8, 4.9. **HRMS (EI)** calcd for $C_{23}H_{21}I$ $[M]^+$: 424.0682, found 424.0686.

Light On/Off Experiment

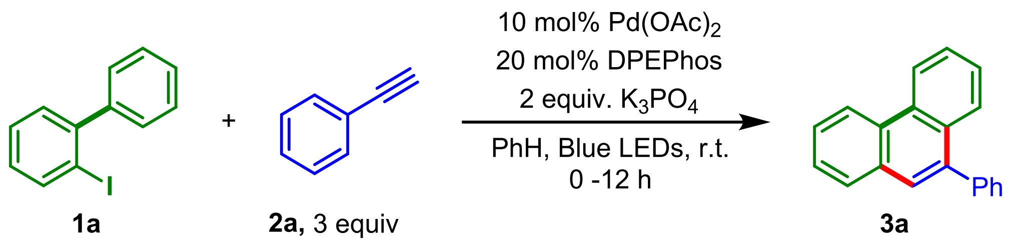

Following the typical procedure described above, the reaction was carried out by the mixture of **1a** (56.0 mg, 0.2 mmol, 1.0 equiv), ethynylbenzene (61.3 mg, 0.6 mmol, 3.0 equiv), $Pd(OAc)_2$ (4.5 mg, 0.02 mmol, 10 mol%), DPEPhos (21.5 mg, 0.04 mmol, 20 mol%) and K_3PO_4 (84.8 mg, 0.4 mmol, 2.0 equiv) in PhH (2.0 mL) at room temperature in nitrogen atmosphere under the irradiation of blue LED lamps. The reaction mixture was stirred under alternating periods (2 h) of irradiation and darkness. Control the reaction time for 2.0 h, 4.0 h, 6.0 h, 8.0 h, 10.0 h, and 12.0 h. After completion of the reaction, the resulting mixture was diluted with acetone (5.0 mL), filtered (Celite), and concentrated under a reduced pressure. After the insoluble molecular sieves were removed through a Nylon synringe filter, the filtrate was analyzed by ^1H NMR to determine the yield of the corresponding product using CH_2Br_2 as an internal standard. The results are list in below picture (**Supplementary Fig. 6**).

Supplementary Fig. 6. Light On/Off experiment

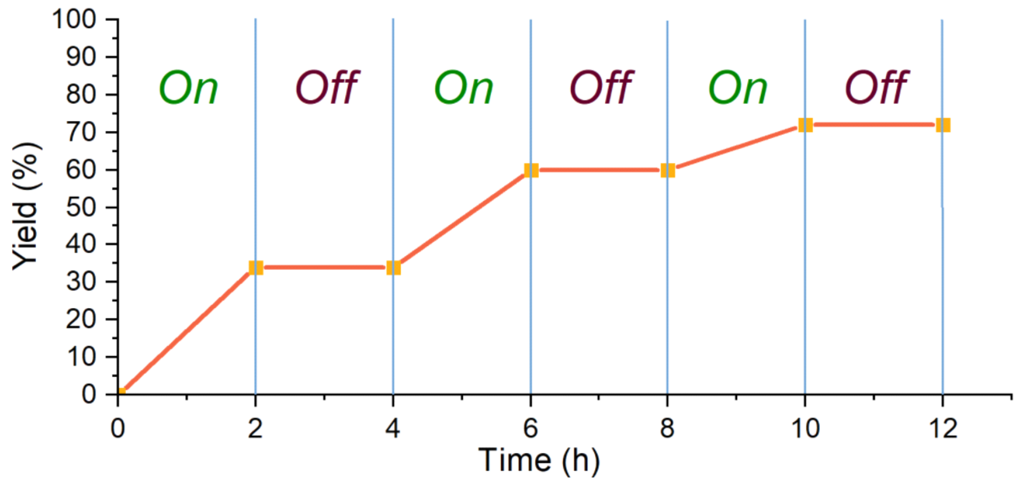

Light On/Off experiment

EPR Experiment

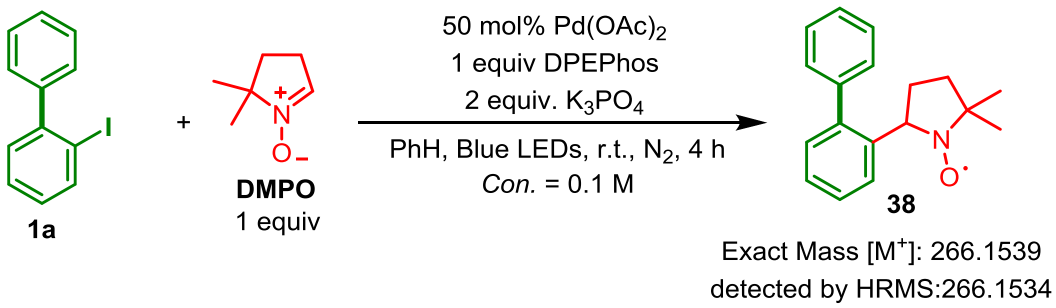

An oven-dried 4.0 mL vial was charged with 2-aryl iodinated arenes (28 mg, 0.1 mmol, 1.0 equiv.), $Pd(OAc)_2$ (11.3 mg, 0.05 mmol, 50 mol%), DPEPhos (53.8 mg, 0.1 mmol, 1 equiv.), DMPO (11.3 mg, 0.1 mmol, 1 equiv.) and K_3PO_4 (43 mg, 0.2 mmol, 2.0 equiv.). It was directly transferred in a nitrogen-filled glovebox with caps. In the glovebox, 1 mL of degassed benzene (PhH) were added to the vial. The vial was tightly sealed, transferred out of glovebox and stirred at room temperature under the irradiation of blue LEDs lamps for 4 hours. After the reaction finished, the electron paramagnetic resonance (EPR) analysis of the reaction mixture revealed a strong radical signal **(Supplementary Fig. 7)**, which would be assigned to the trapped cyclohexyl radical by DMPO according to the simulated spectrum **(Supplementary Fig. 8)** (fitting result: g =2.0040, aN = 9.95 G, aH = 0.98 G).

Supplementary Fig. 7. EPR spectra

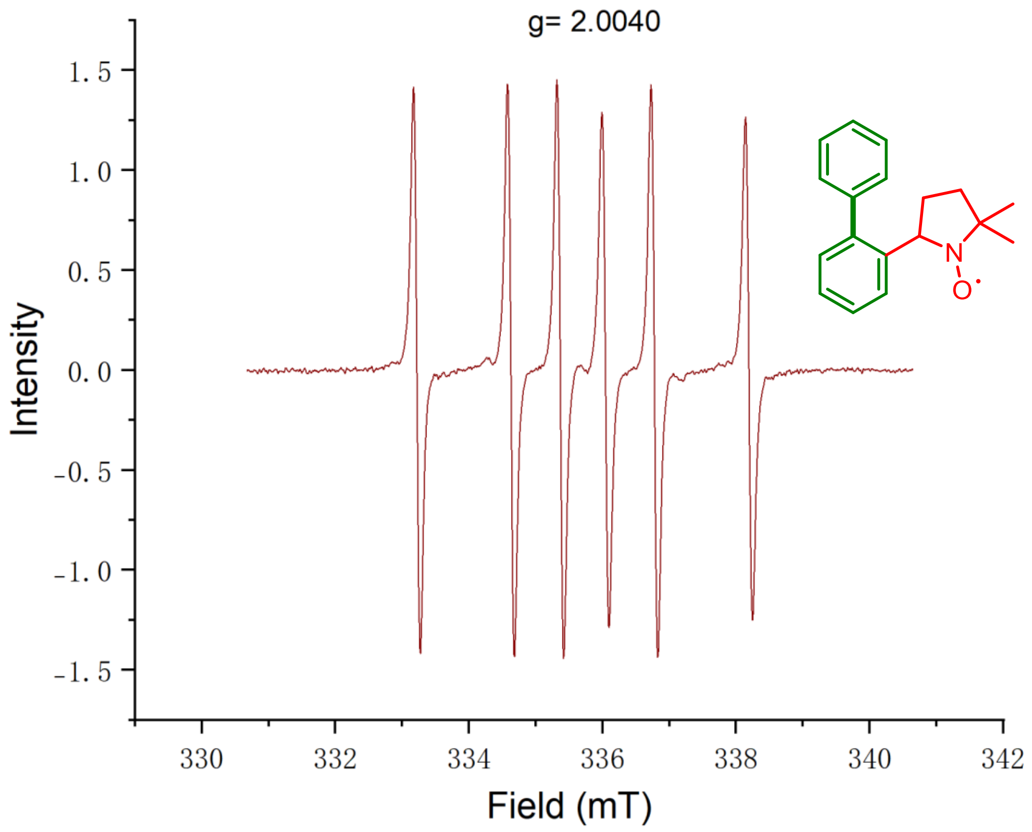

Supplementary Fig. 8. HRMS of the EPR experiment

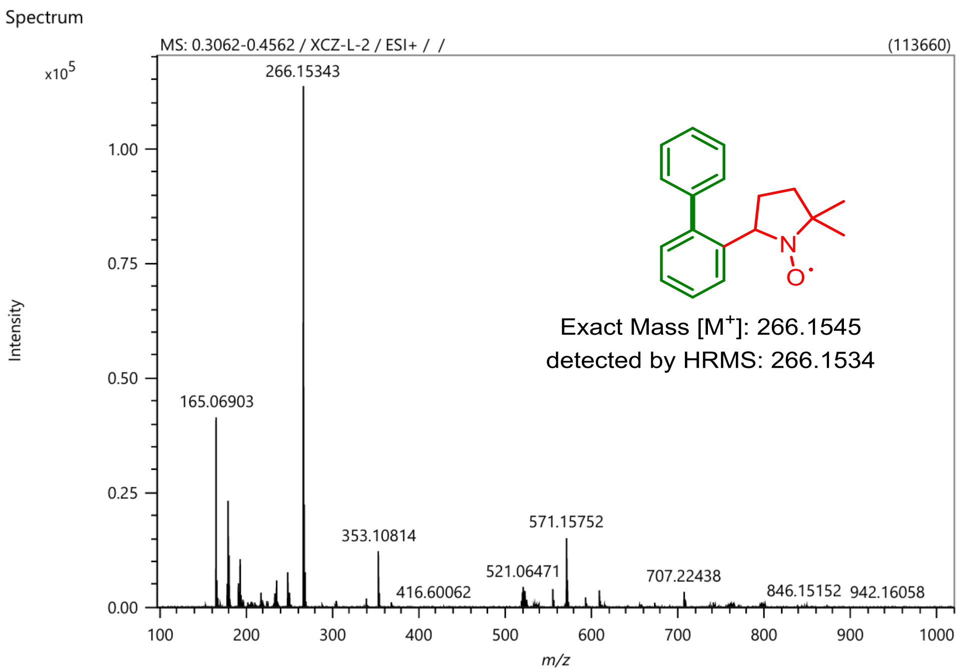

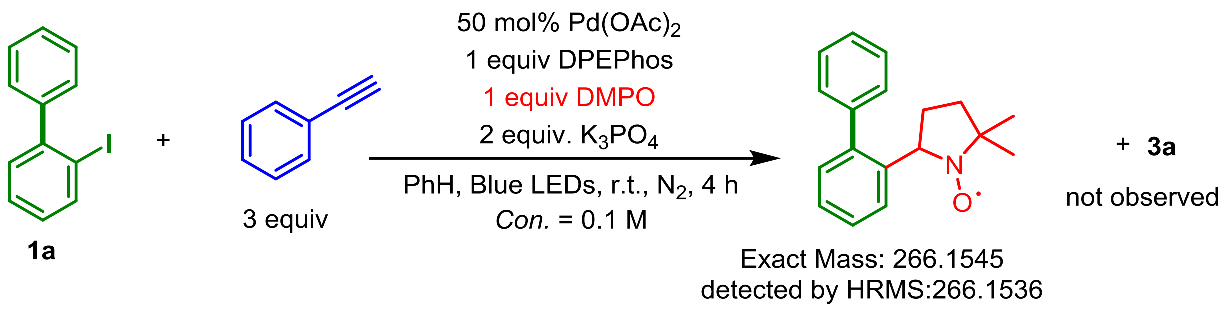

An oven-dried 4.0 mL vial was charged with 2-aryl iodinated arenes (28 mg, 0.1 mmol, 1.0 equiv.), terminal alkyne (34 mg, 0.3 mmol, 3.0 equiv.), $Pd(OAc)_2$ (11.3 mg, 0.05 mmol, 50 mol%), DPEPhos (53.8 mg, 0.1 mmol, 1 equiv.), DMPO (11.3 mg, 0.1 mmol, 1 equiv.) and K_3PO_4 (43 mg, 0.2 mmol, 2.0 equiv.). It was directly transferred in a nitrogen-filled glovebox with caps. In the glovebox, 1 mL of degassed benzene (PhH) were added to the vial. The vial was tightly sealed, transferred out of glovebox and stirred at room temperature under the irradiation of blue LEDs lamps for 4 hours. After the reaction finished, the electron paramagnetic resonance (EPR) analysis of the reaction mixture revealed a strong radical signal **(Supplementary Fig. 9)**, which would be assigned to the trapped cyclohexyl radical by DMPO according to the simulated spectrum **(Supplementary Fig. 10)** (fitting result: g =2.0040, aN = 9.95 G, aH = 0.98 G).

Supplementary Fig. 9. EPR spectra

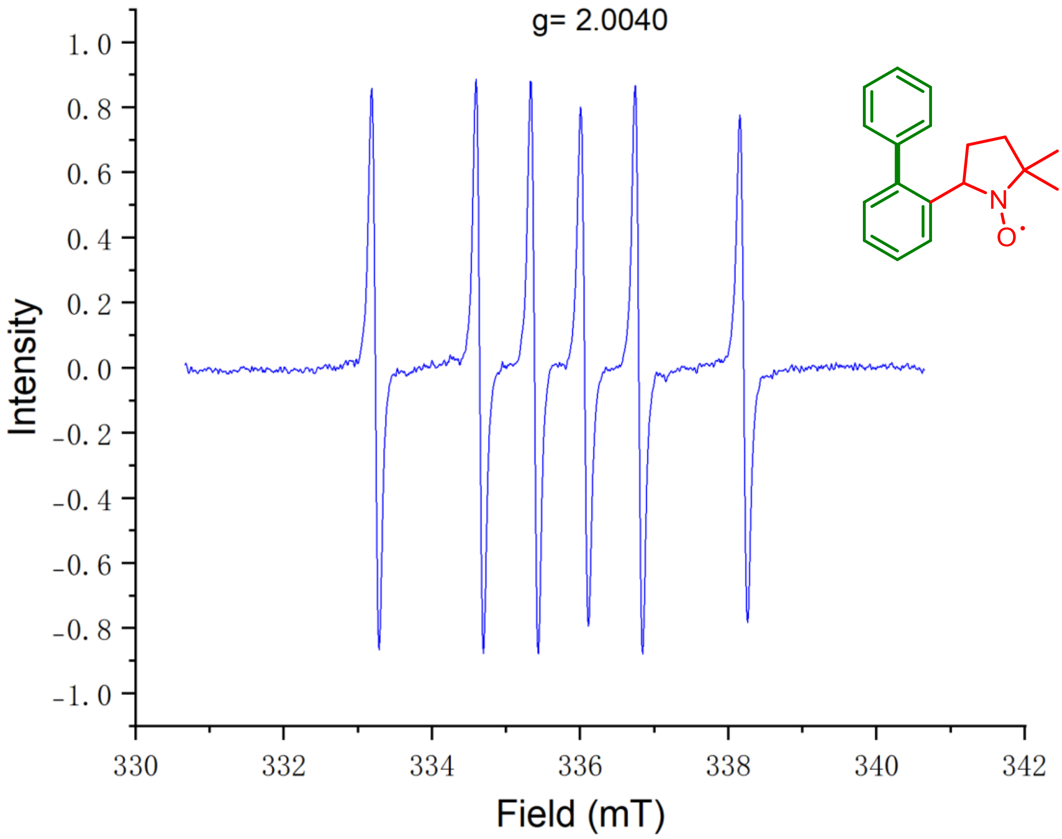

Supplementary Fig. 10. HRMS of the EPR experiment

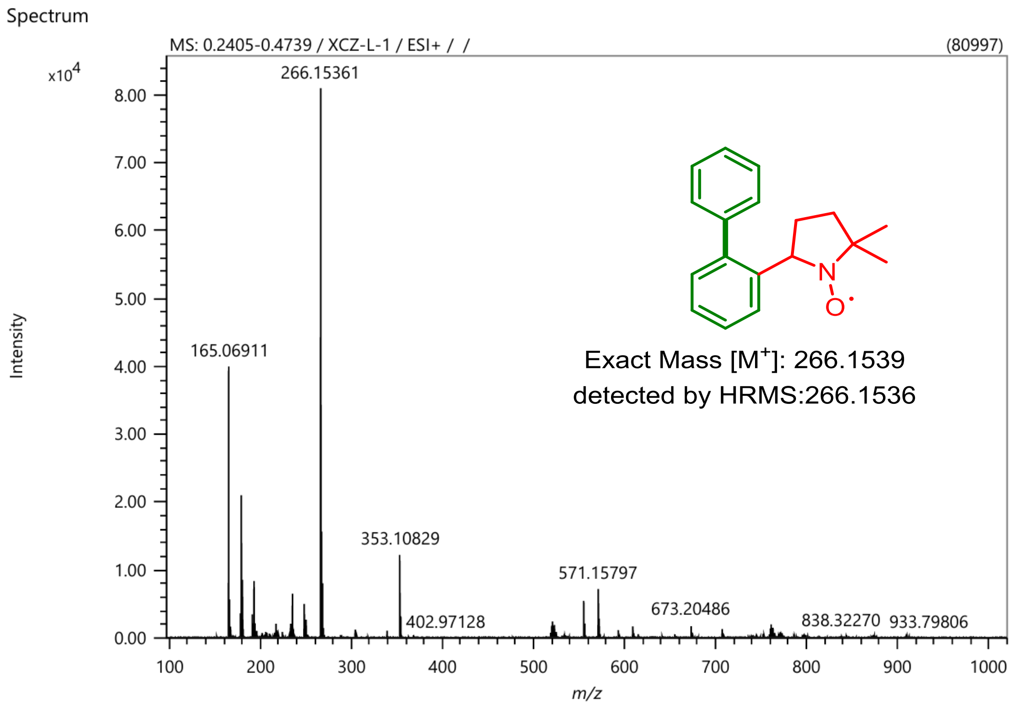

Competitive KIE Experiment

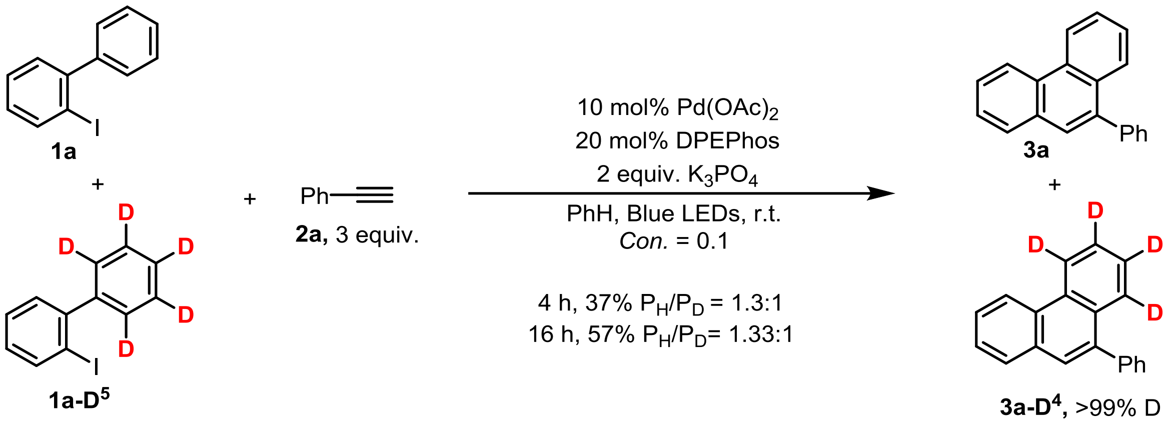

Following the typical procedure described above, the reaction was carried out by the mixture of **1a** (28.0 mg, 0.1 mmol, 1.0 equiv), **1a-D⁵** (28.5 mg, 0.1 mmol, 1.0 equiv), (ethynylbenzene (61.3 mg, 0.6 mmol, 3.0 equiv), $Pd(OAc)_2$ (4.5 mg, 0.02 mmol, 10 mol%), DPEPhos (21.5 mg, 0.04 mmol, 20 mol%) and K_3PO_4 (84.8 mg, 0.4 mmol, 2.0 equiv) in PhH (2.0 mL) at room temperature in nitrogen atmosphere under the irradiation of blue LED lamps. Control the reaction time for 4 hours and 16 hours. Column chromatography on silica gel eluent: Petroleum ether) afforded the title product **3a** and **3a-D^4** (>99%) isolated yield as a colorless oil; R_f = 0.6 (Petroleum ether). The KIE between **3a** and **3a-D^4** was determined by ^{1}H NMR. **^{1}H NMR (400 MHz, $CDCl_3$)** δ 8.79 (d, *J* = 8.4 Hz, 1H), 8.74 (d, *J* = 8.4 Hz, 1.8H), 7.92 (t, *J* = 8.4 Hz, 2.8H), 7.72 – 7.59 (m, 6.4H), 7.60 – 7.44 (m, 10.2H). **^{13}C NMR (101 MHz, $CDCl_3$)** δ 140.8, 138.7, 131.5, 131.1, 130.0, 128.6, 128.3, 127.5, 127.3, 126.9, 126.8, 126.6, 126.5, 126.4, 122.9, 122.5.

Synthesis of 3a-D^4

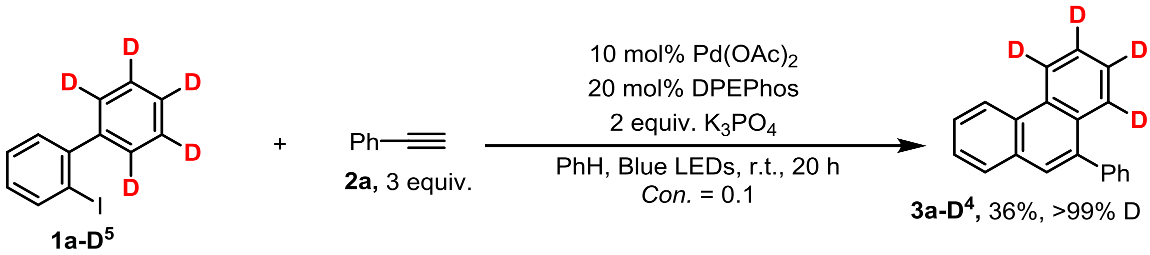

Following the typical procedure described above, the reaction was carried out by the mixture of **1a-D^5** (57.1 mg, 0.2 mmol, 0.2 mmol), ethynylbenzene (61.3 mg, 0.6 mmol, 3.0 equiv), $Pd(OAc)_2$ (4.5 mg, 0.02 mmol, 10 mol%), DPEPhos (21.5 mg, 0.04 mmol, 20 mol%) and K_3PO_4 (84.8 mg, 0.4 mmol, 2.0 equiv) in PhH (2.0 mL) at room temperature in nitrogen atmosphere under the irradiation of blue LED lamps for 20 h. Column chromatography on silica gel eluent: Petroleum ether) afforded the title product **3a-D^4** 36%(18.6 mg) (D >99%) isolated yield as colorless oil; R_f = 0.6 (Petroleum ether). **^{1}H NMR (400 MHz, $CDCl_3$)** δ 8.73 (d, *J* = 8.4 Hz, 1H), 7.91 (dd, *J* = 7.6, 1.6 Hz, 1H), 7.73 – 7.61 (m, 3H), 7.59 – 7.45 (m, 5H). **^{13}C NMR (101 MHz, $CDCl_3$)** δ 140.8, 138.7, 131.5, 131.0, 130.5, 130.0, 129.9, 128.6, 128.3, 127.5,

127.3, 126.8, 126.6, 122.5. **HRMS (EI)** calcd for $C_{20}H_{14}D_4$ $[M]^+$: 258.1341, found 258.1345.

Intermolecular parallel KIE experiments with 2-iodo-1,1'-biphenyl **(1a)** and 2-iodo-1,1'-biphenyl-2'-*d5*(**1a-D⁵**).

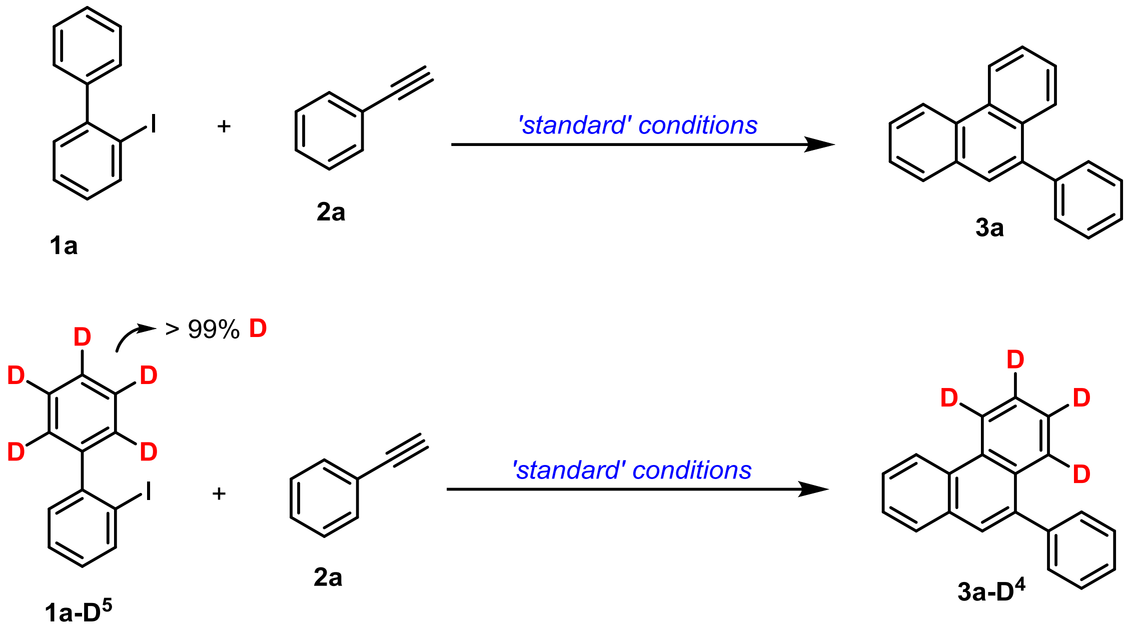

Reactions were performed with 2-iodo-1,1'-biphenyl **(1a)** (28.1 mg, 0.1 mmol) or 2-iodo-1,1'-biphenyl-2'-*d5* (**1a-D**^{5}) (28.6 mg, 0.1 mmol), $Pd(OAc)_2$ (2.3 mg, 0.01 mmol) and DPEPhos (10.8 mg, 0.02 mmol) and K_3PO_3 (43.2 mg, 0.2 mmol), 1 mL of degassed benzene, following the general procedure of the desaturation reaction. After the reaction proceeded for the corresponding period of time, the reaction solution was filtered through celite and the crude 1H NMR was taken using CH_2Br_2 as the internal standard **(Supplementary Fig. 11-12).**

**1a(M)**	**Time**(min)	**3a**	Initial rate(M/min)
0.1	10	0.007	0.00044
	20	0.013	
	30	0.017	
	40	0.021	
	50	0.025	

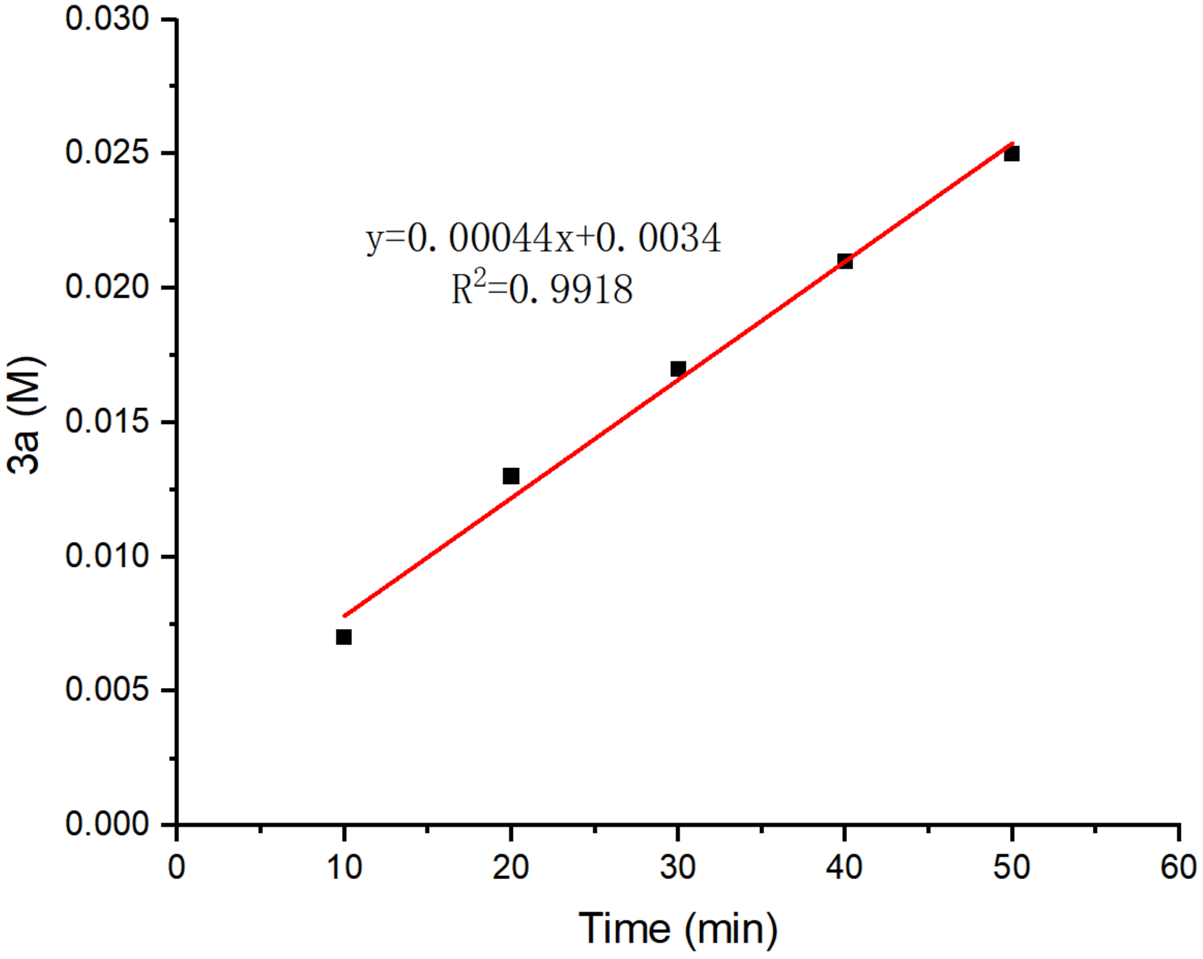

Supplementary Fig. 11. Initial rate data for the desaturation of 3a at 1a(0.1M)

**1a(M)**	**Time**(min)	**3a-D[4]**	Initial rate(M/min)
0.1	10	0.002	0.00034
	20	0.006	
	30	0.009	
	40	0.012	
	50	0.016	

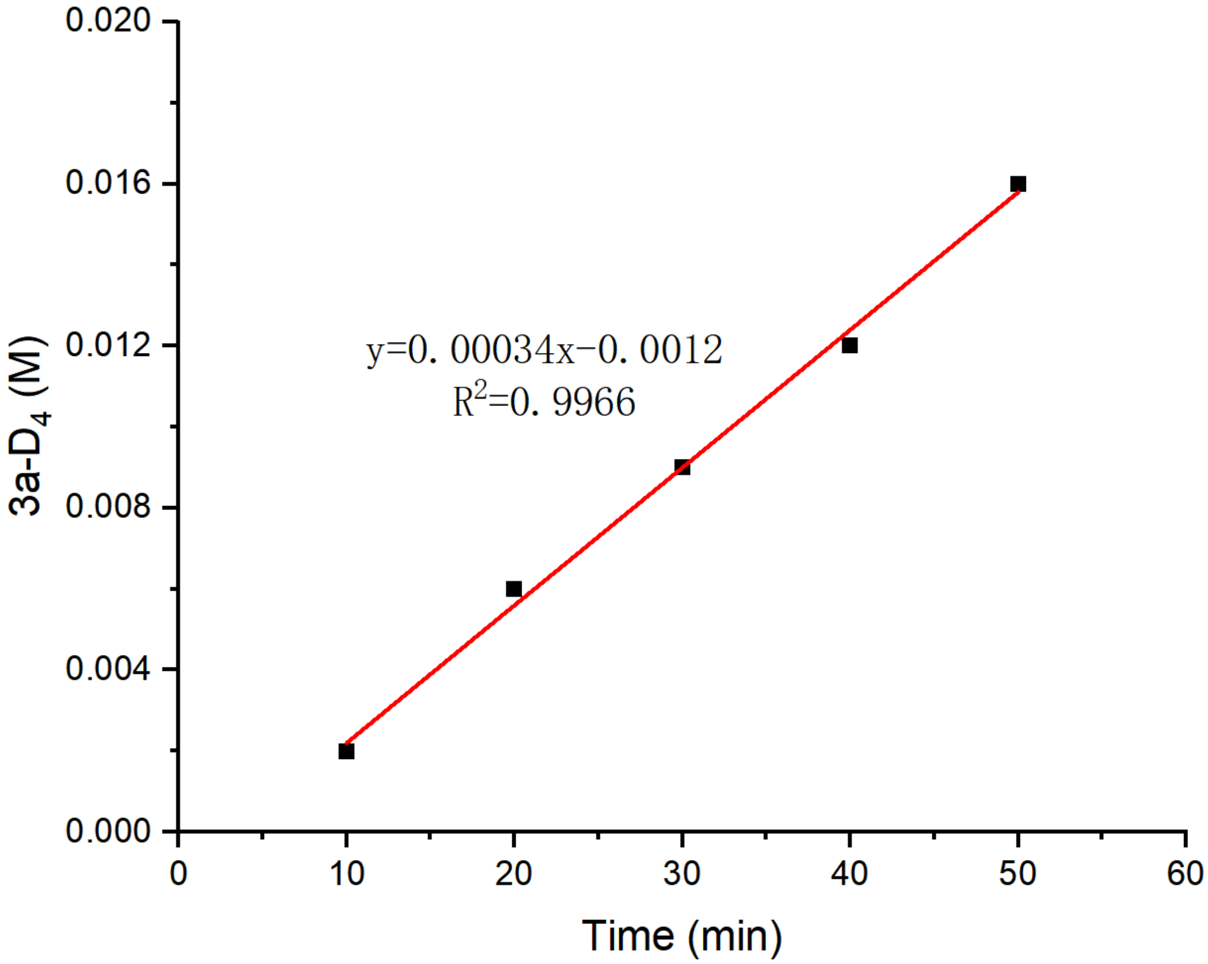

Supplementary Fig. 12. Initial rate data for the desaturation of 3a-D_4 at 1a (0.1M)

Typical procedure for the synthesis of 1a-D

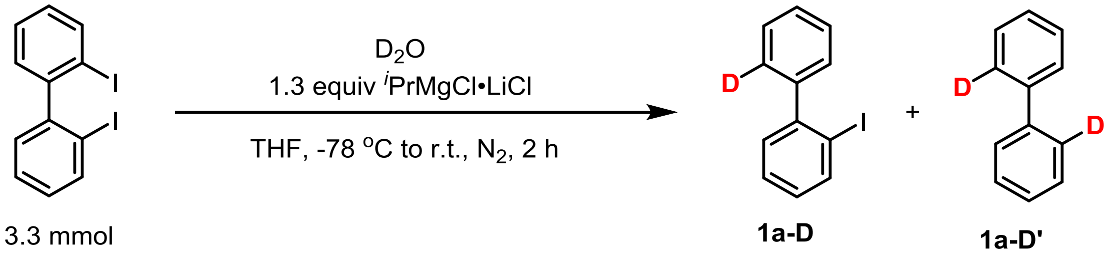

1a-D : 1a-D' = 3.33 : 1

To a stirred solution of 2,2'-diiodo-1,1'-biphenyl (1.34 g, 3.3 mmol) in THF (10 mL) under a nitrogen atmosphere at -78 ℃, ^{i}PrMgCl • LiCl (3.3 mL, 4.3 mmol, 1.3 equiv) was added. The mixture was stirred at -78 ℃ for 2 hours. Deuterium water (0.2 ml D_2O) was added dropwise to quench the reaction. After filtration and concentration, the crude product was purified by column chromatography on silica gel (eluent: Petroleum ether), yielding the desired compound **1a-D** (361.8 mg, 39% yield, containing 23 mol %

inseparable 2,2'-dideutero-1,1'-biphenyl) as a white solid. The extent of D incorporation (~100%) was calculated by NMR analysis. **2-iodo-1,1'-biphenyl-2'-*d*.** **^{1}H NMR (400 MHz, $CDCl_3$)** δ 7.99 (d, *J* = 7.6 Hz, 1H), 7.53 – 7.31 (m, 6H), 7.06 (t, *J* = 7.8 Hz, 1H). **1,1'-biphenyl-2,2'-*d*$_2$.** **^{1}H NMR (400 MHz, $CDCl_3$)** δ 7.63 (d, *J* = 7.6 Hz, 2H), 7.53 – 7.31 (m, 6H). **2-iodo-1,1'-biphenyl-2'-*d*.** and **1,1'-biphenyl-2,2'-*d*$_2$.** **^{13}C NMR (101 MHz, CDCl3)** δ 146.5, 144.1, 139.4, 130.0, 129.2, 128.9 (t, *J* = 24.5 Hz), 128.7, 128.7, 128.7, 128.6, 128.1, 127.9, 127.8, 127.6, 127.2, 127.1, 98.6. The spectroscopic data match the reported literature[41].

Competitive KIE Experiment of intermolecular products

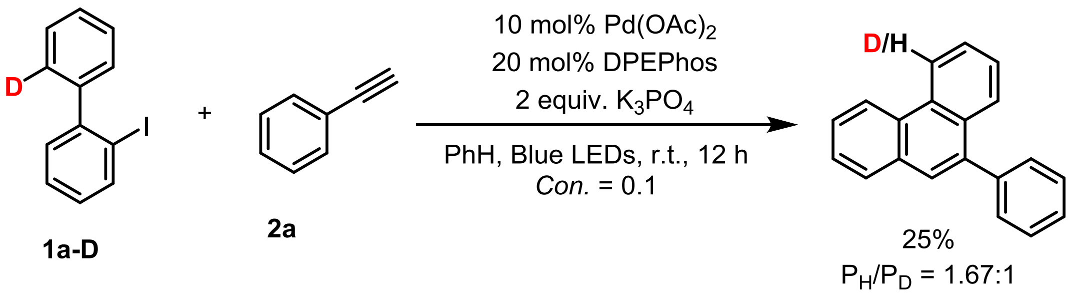

Following the typical procedure described above, the reaction was carried out by the mixture of **1a-D:1a-D'** (3.33:1) (73.1 mg, 0.2 mmol, 0.2 mmol), ethynylbenzene (61.3 mg, 0.6 mmol, 3.0 equiv), $Pd(OAc)_2$ (4.5 mg, 0.02 mmol, 10 mol%), DPEPhos (21.5 mg, 0.04 mmol, 20 mol%) and K_3PO_4 (84.8 mg, 0.4 mmol, 2.0 equiv) in PhH (2.0 mL) at room temperature in nitrogen atmosphere under the irradiation of blue LED lamps for 12 h. Column chromatography on silica gel eluent: Petroleum ether) afforded the title product **3a-D** 25%(12.8 mg) isolated yield as a colorless oil; R_f = 0.6 (Petroleum ether). **^{1}H NMR (400 MHz, $CDCl_3$)** δ 8.79 (d, *J* = 8.2 Hz, 1H), 8.73 (d, *J* = 8.4 Hz, 2H), 7.91 (t, *J* = 8.1 Hz, 3H), 7.71 – 7.60 (m, 7H), 7.57 – 7.43 (m, 10H). **^{13}C NMR (101 MHz, $CDCl_3$)** δ 140.8, 138.7, 131.5, 131.1, 130.0, 128.6, 128.3, 128.2, 127.9, 127.5, 127.3, 126.9, 126.8, 126.6, 126.5, 126.4, 126.3, 122.9, 122.5.

NMR Spectra

Supplementary Fig. 13. ^{1}H NMR of 1l (400 MHz, $CDCl_3$)

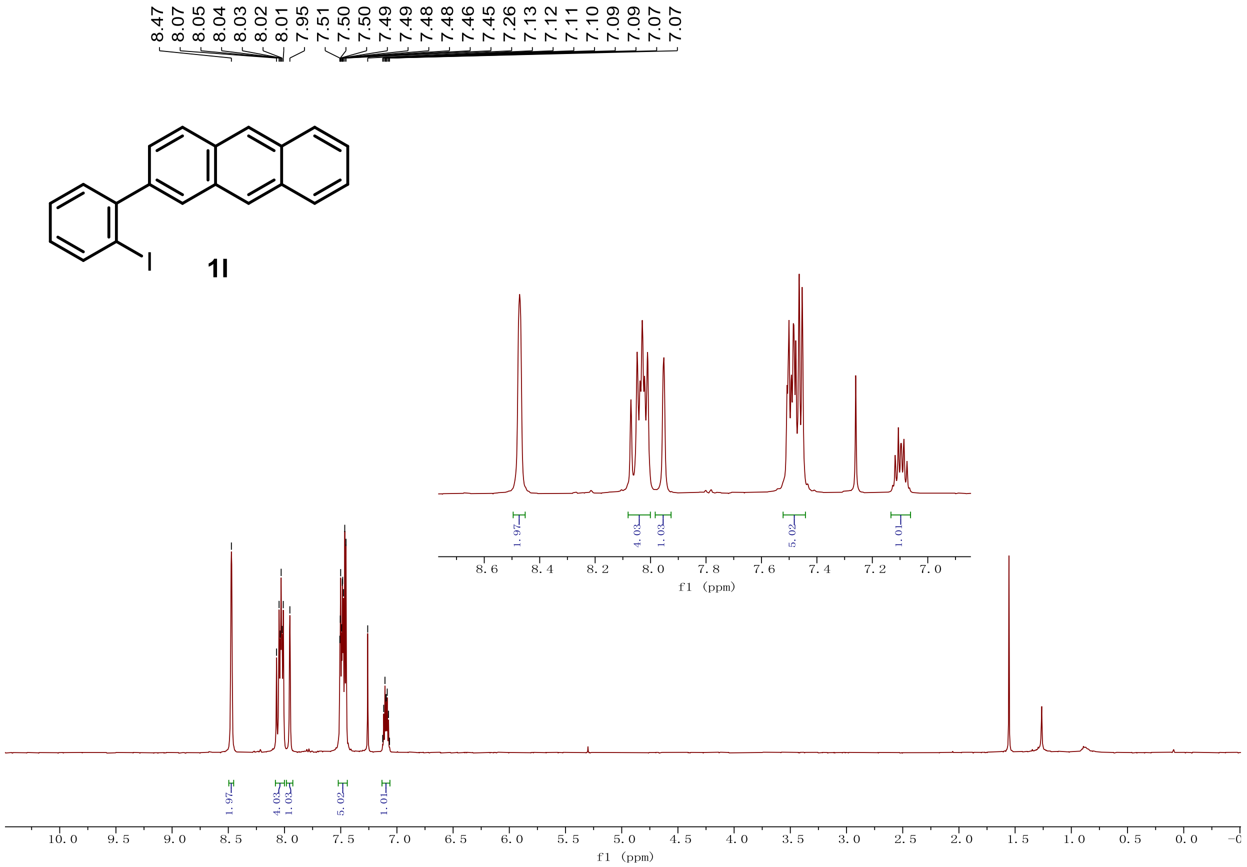

Supplementary Fig. 14. ^{13}C NMR of 1l (101 MHz, $CDCl_3$)

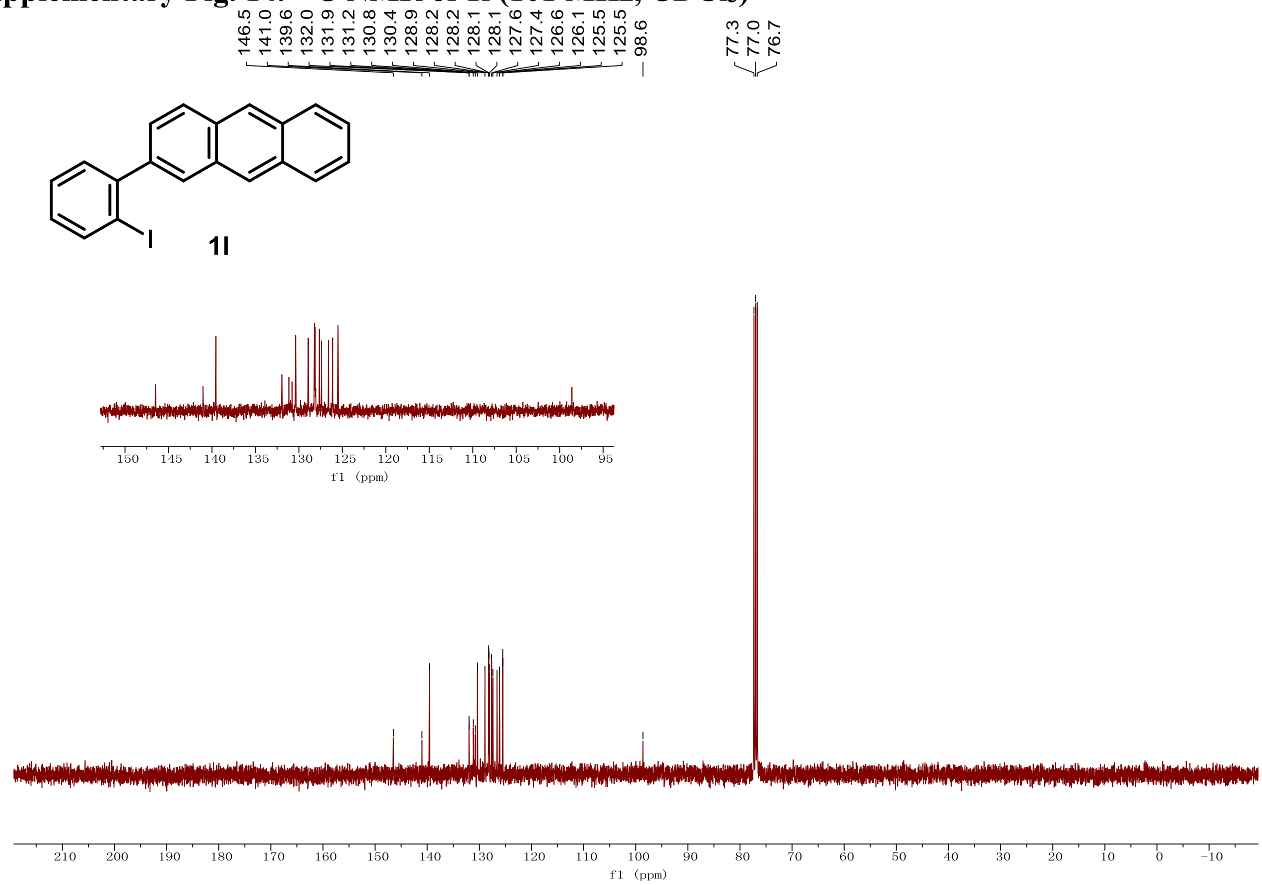

Supplementary Fig. 15. ^{1}H NMR of 1aa (400 MHz, $CDCl_3$)

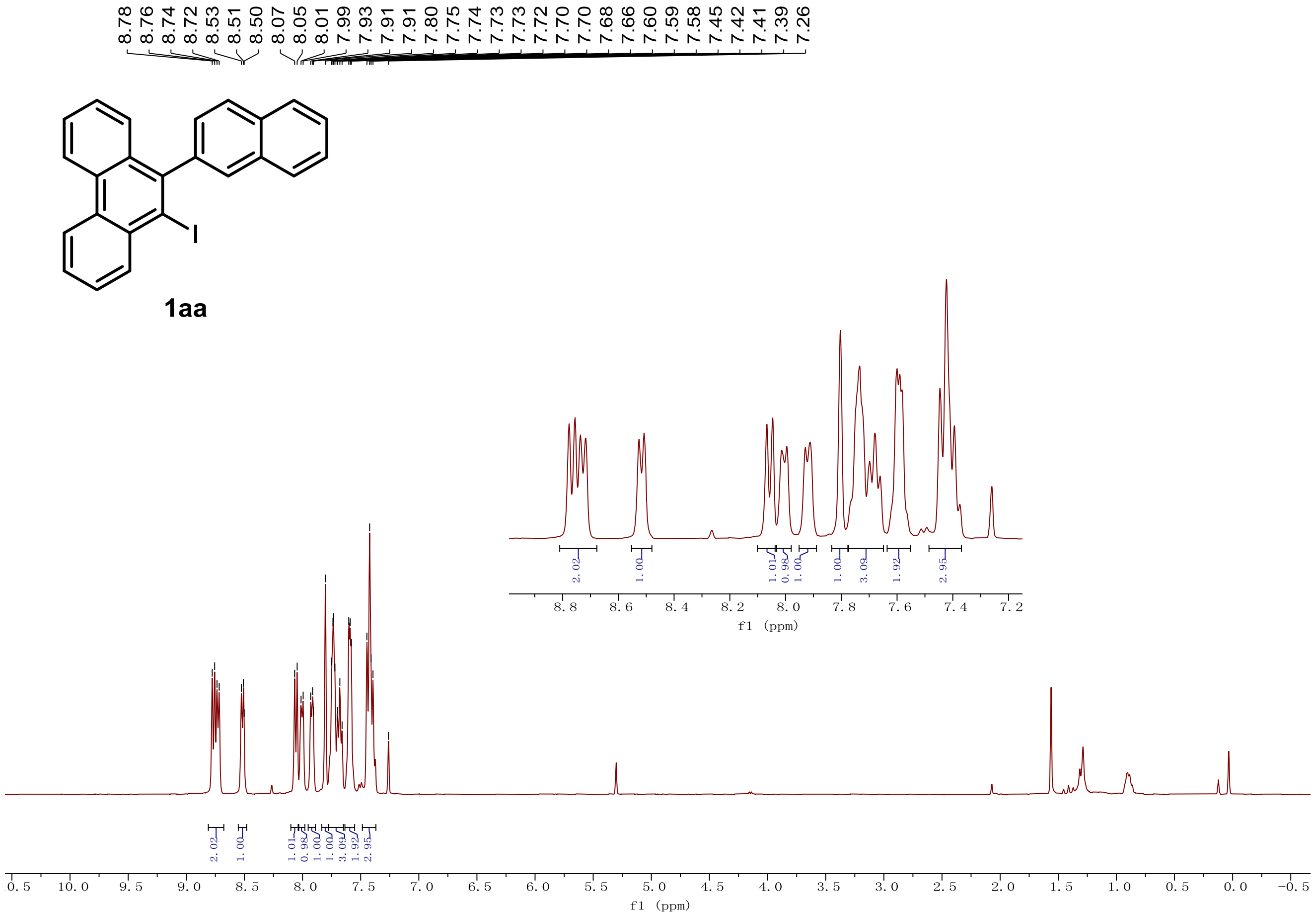

Supplementary Fig. 16. ^{13}C NMR of 1aa (101 MHz, $CDCl_3$)

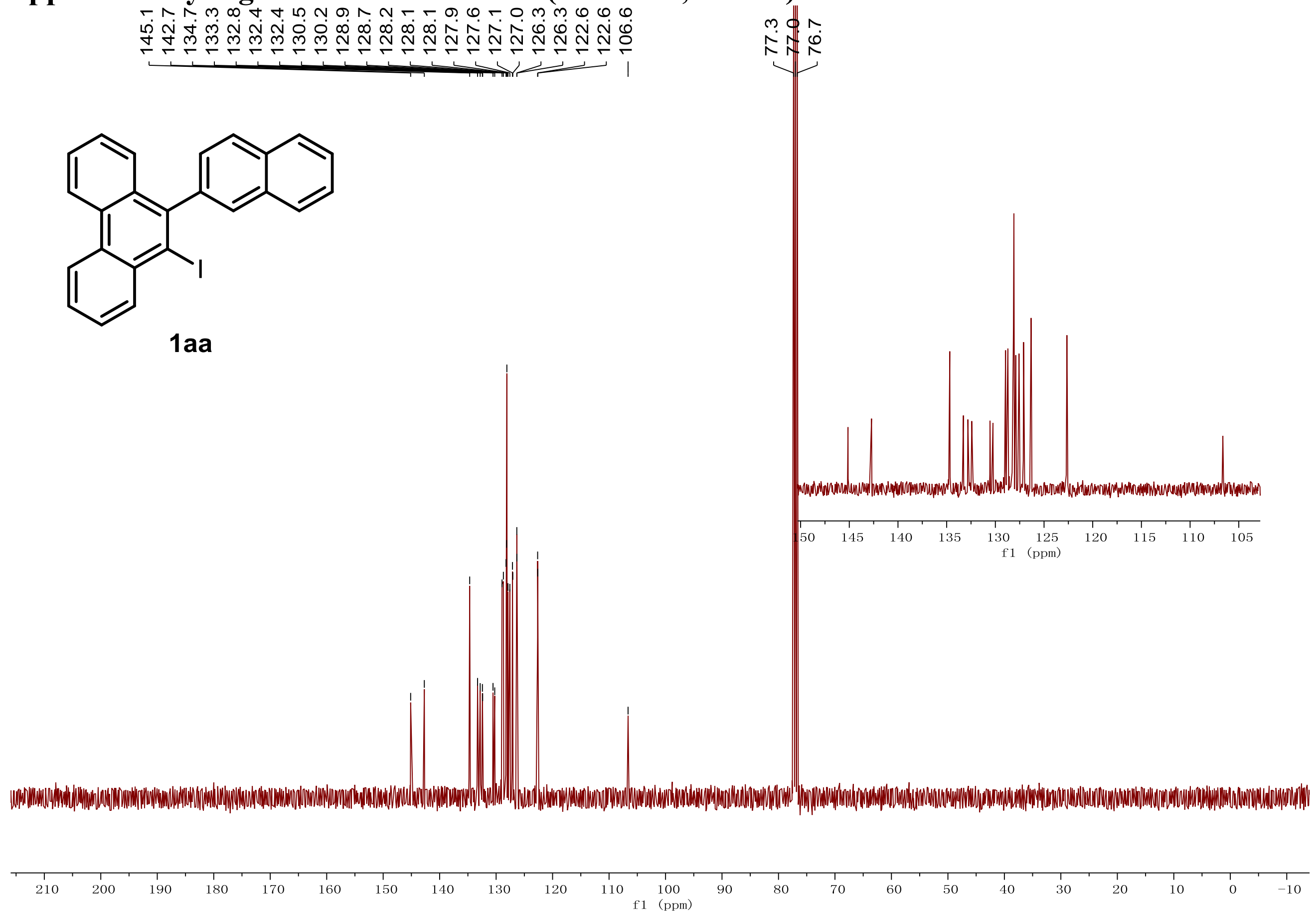

Supplementary Fig. 17. ^{1}H NMR of 3a (400 MHz, $CDCl_3$)

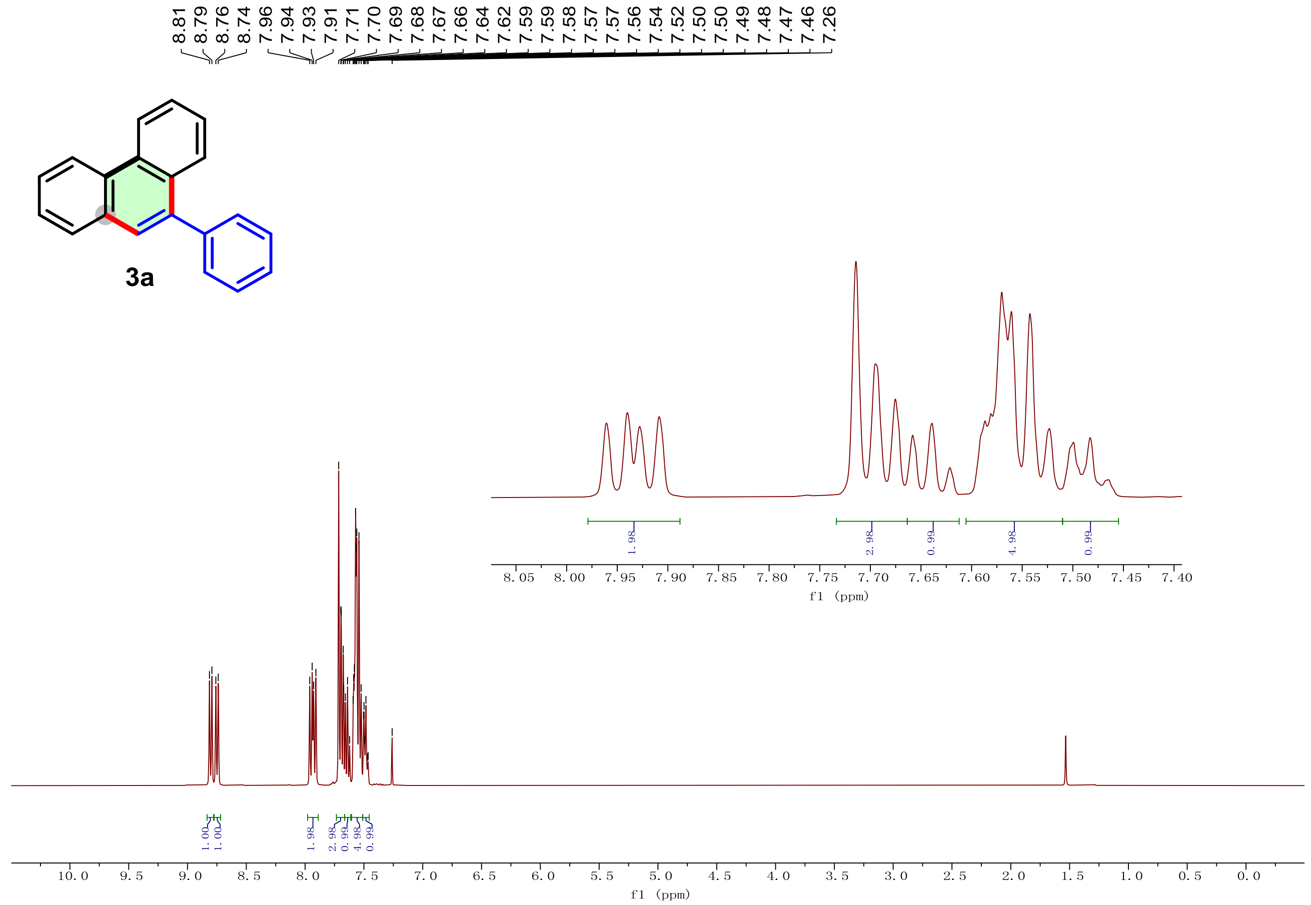

Supplementary Fig. 18. ^{13}C NMR of 3a (101 MHz, $CDCl_3$)

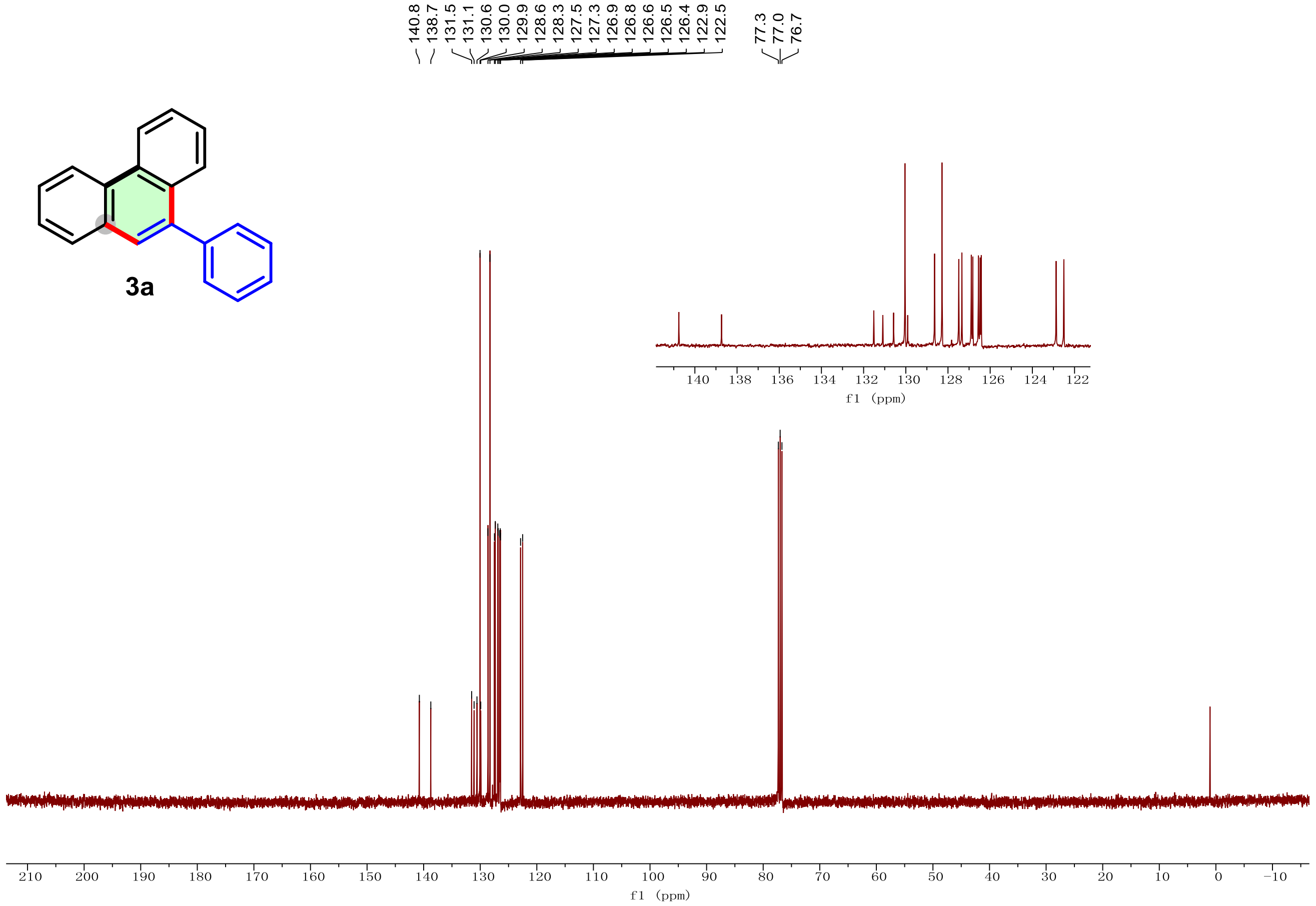

Supplementary Fig. 19. ^{1}H NMR of 3b (400 MHz, $CDCl_3$)

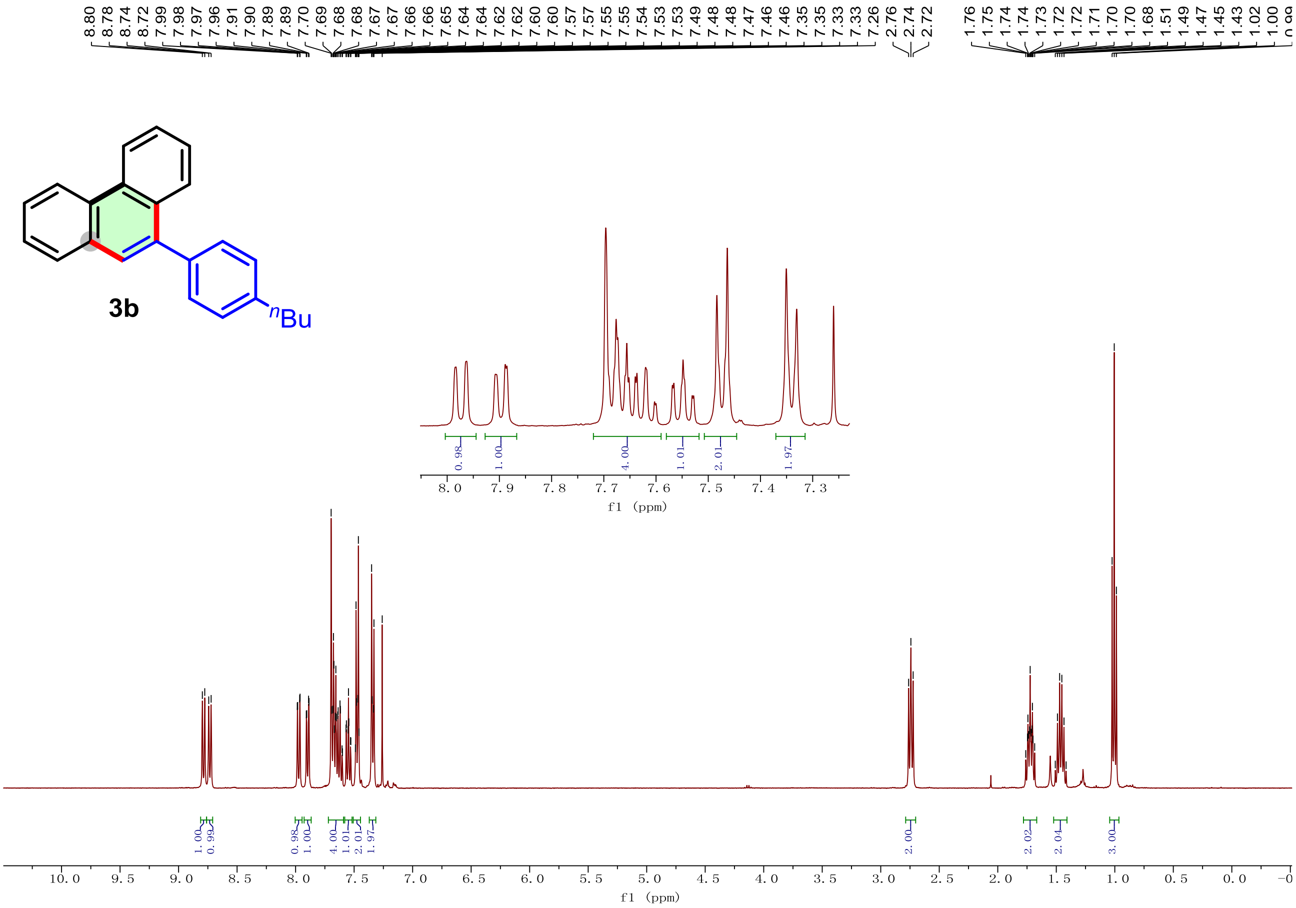

Supplementary Fig. 20. ^{13}C NMR of 3b (101 MHz, $CDCl_3$)

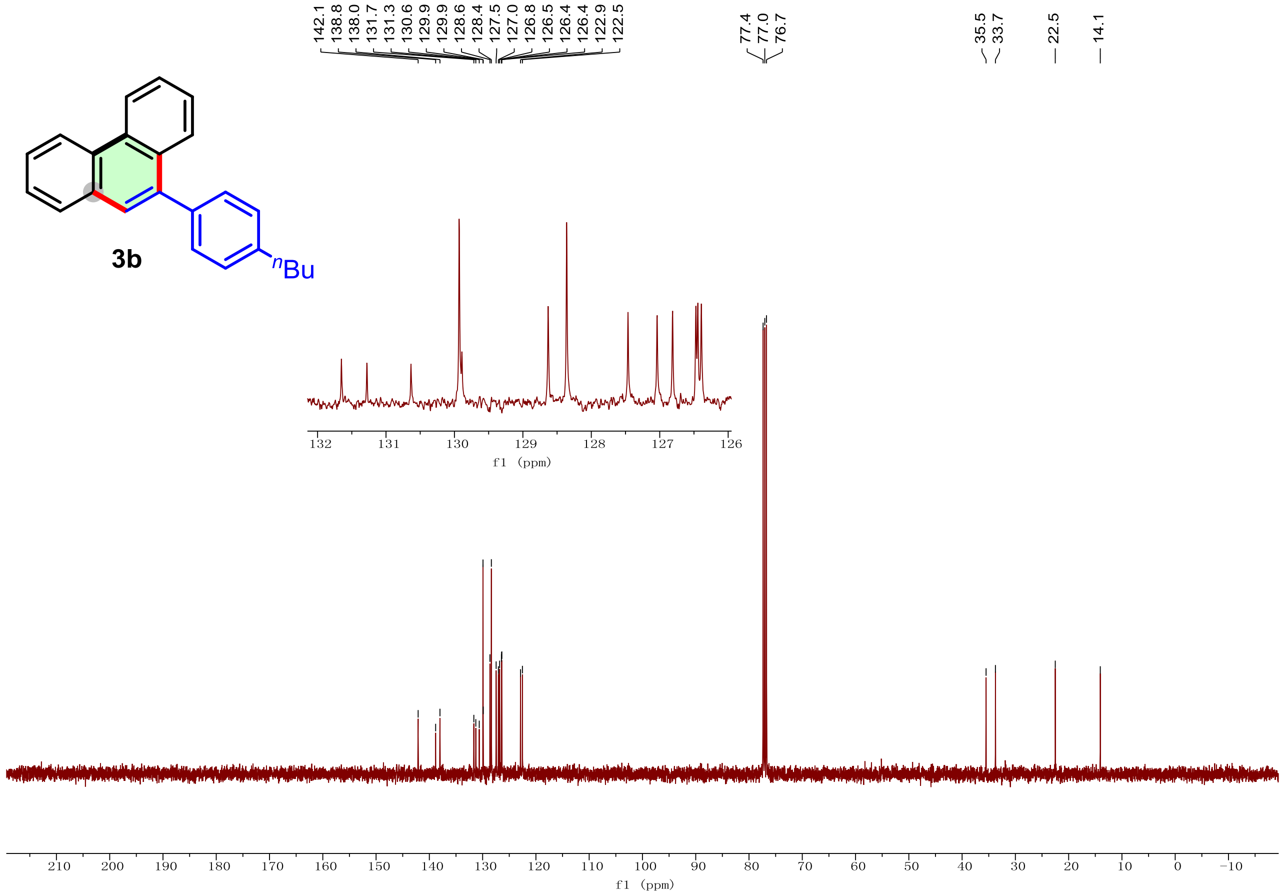

Supplementary Fig. 21. ^{1}H NMR of 3c (400 MHz, $CDCl_3$)

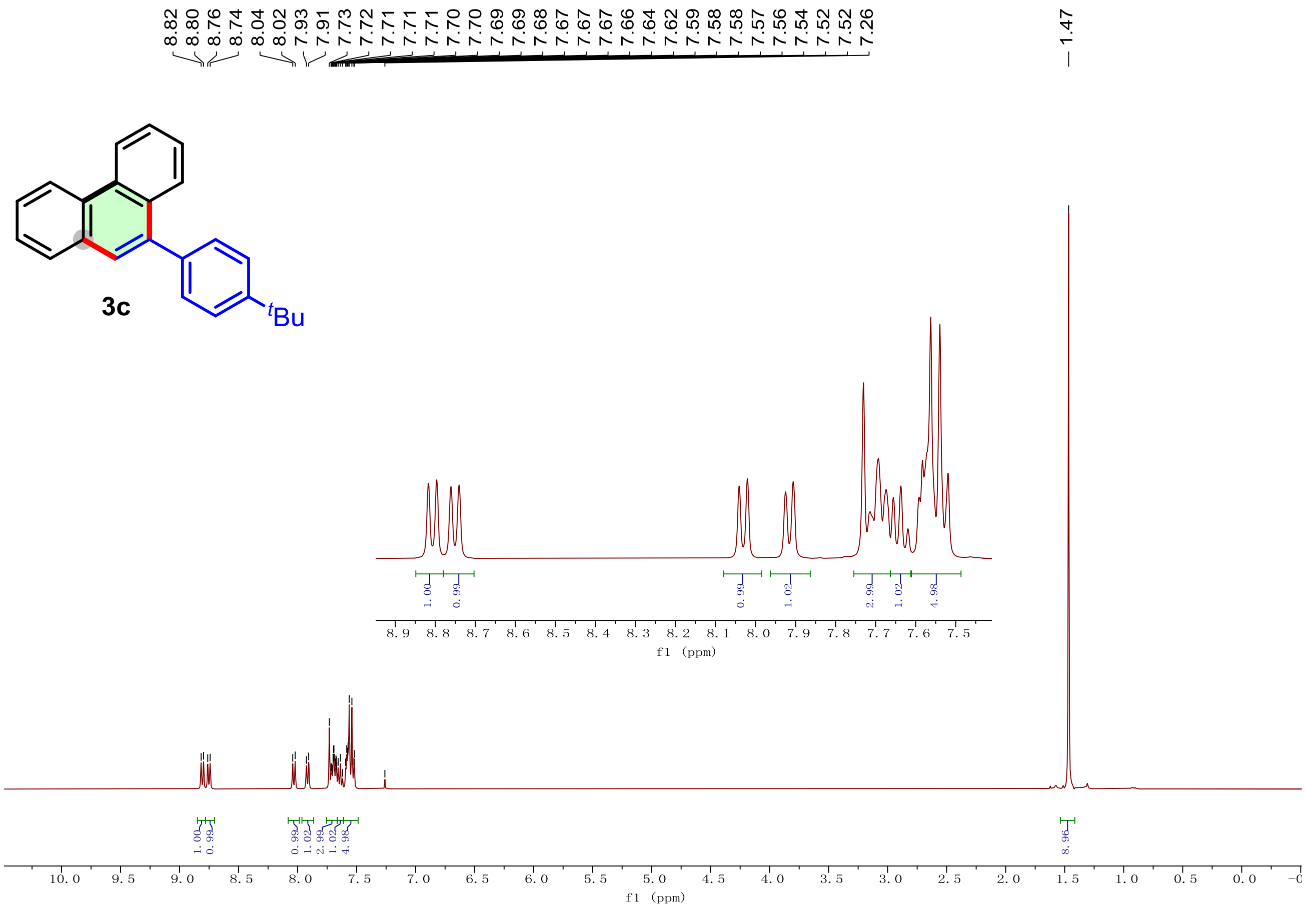

Supplementary Fig. 22. ^{13}C NMR of 3c (101 MHz, $CDCl_3$)

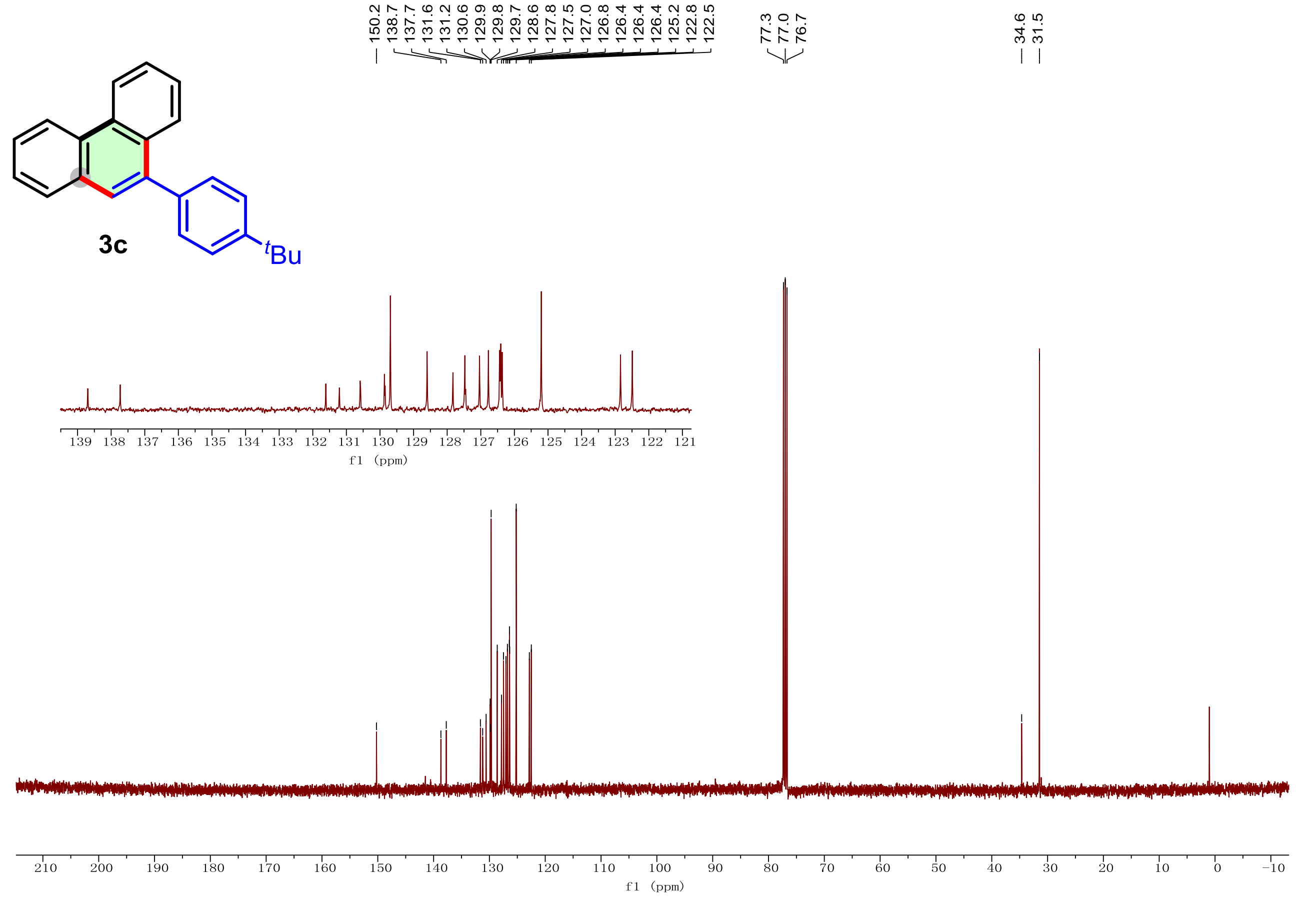

Supplementary Fig. 23. ^{1}H NMR of 3d (400 MHz, CDCl$_3$)

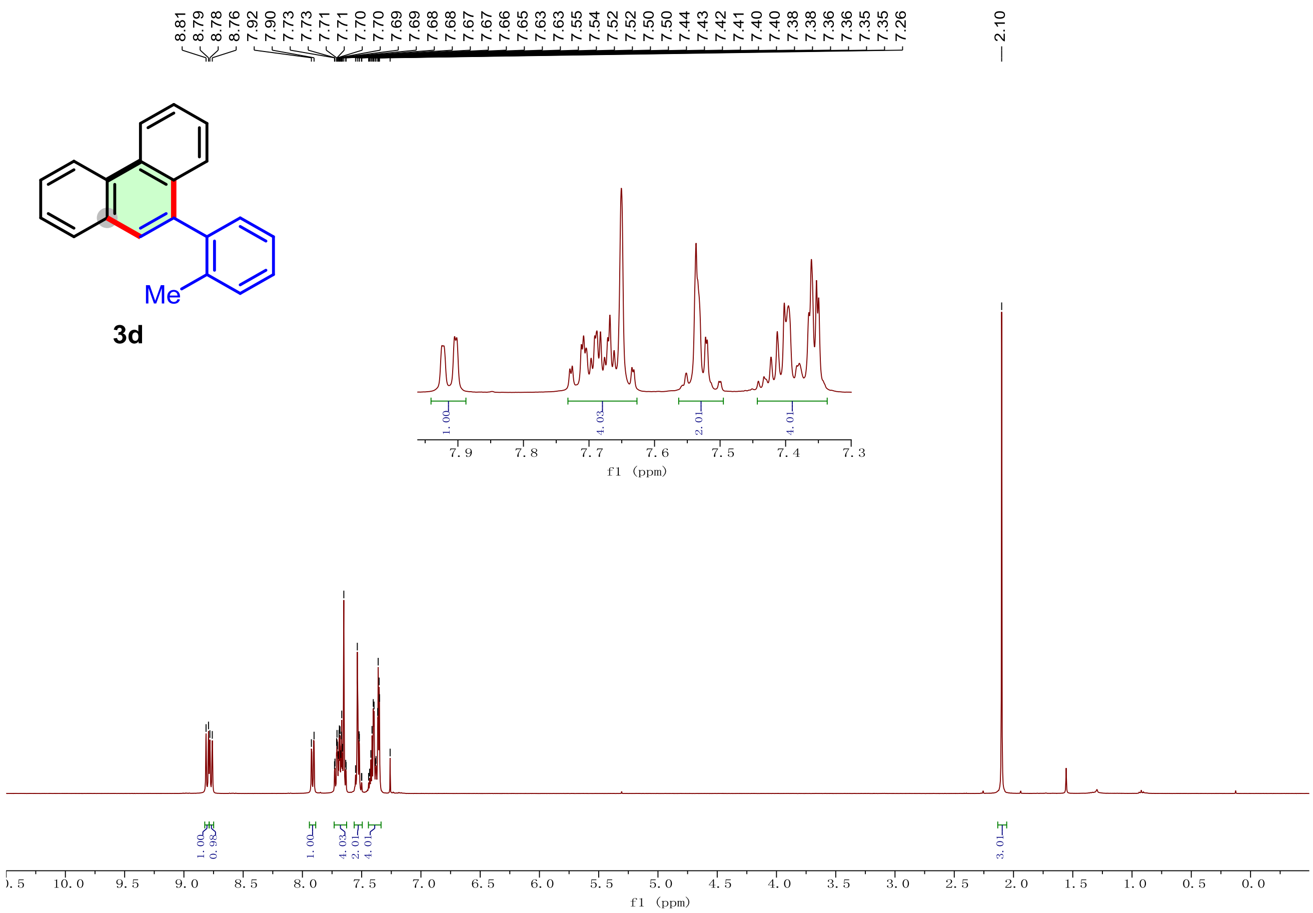

Supplementary Fig. 24. ^{13}C NMR of 3d (101 MHz, CDCl$_3$)

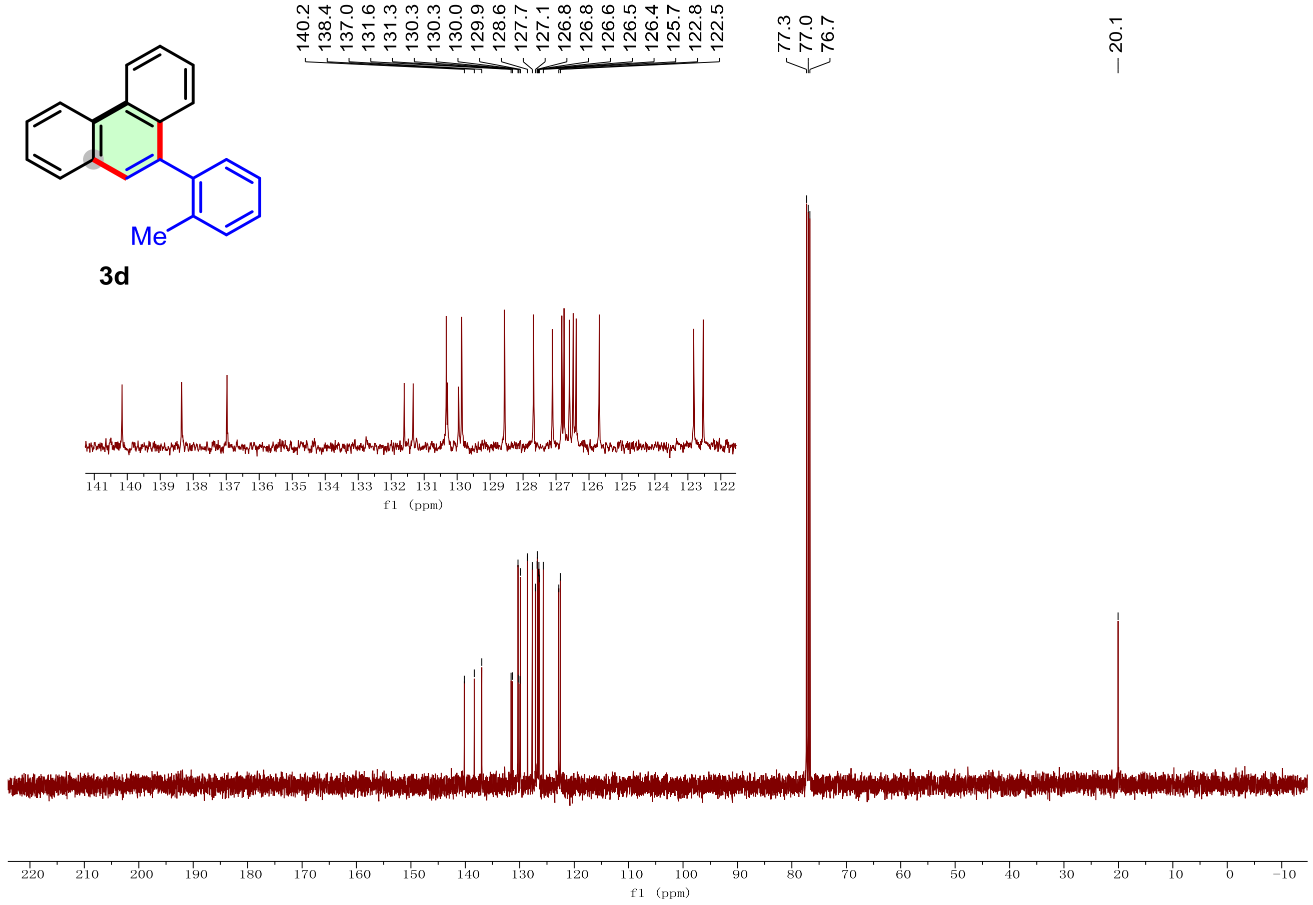

Supplementary Fig. 25. ^{1}H NMR of 3e (400 MHz, $CDCl_3$)

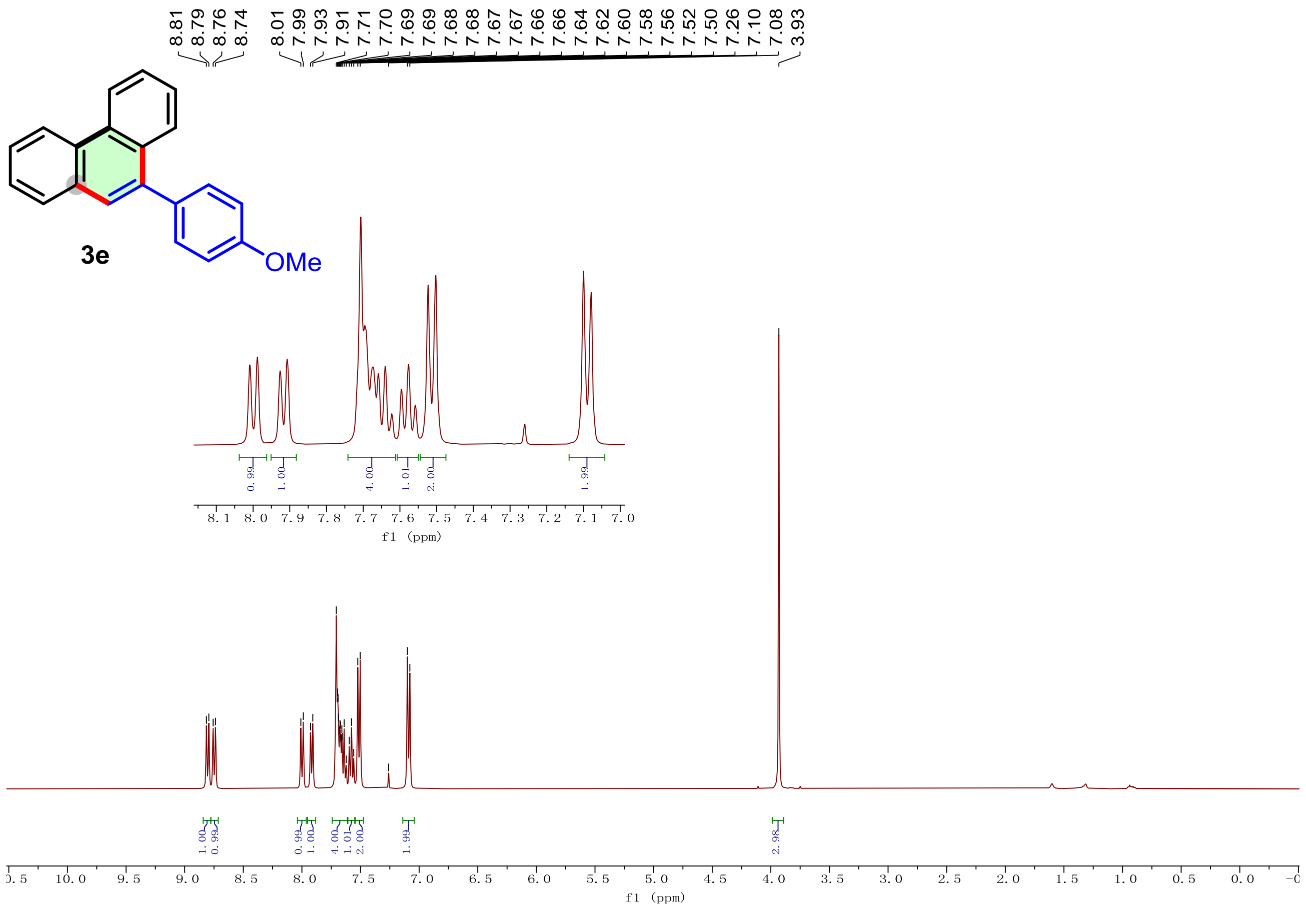

Supplementary Fig. 26. ^{13}C NMR of 3e(101 MHz, $CDCl_3$)

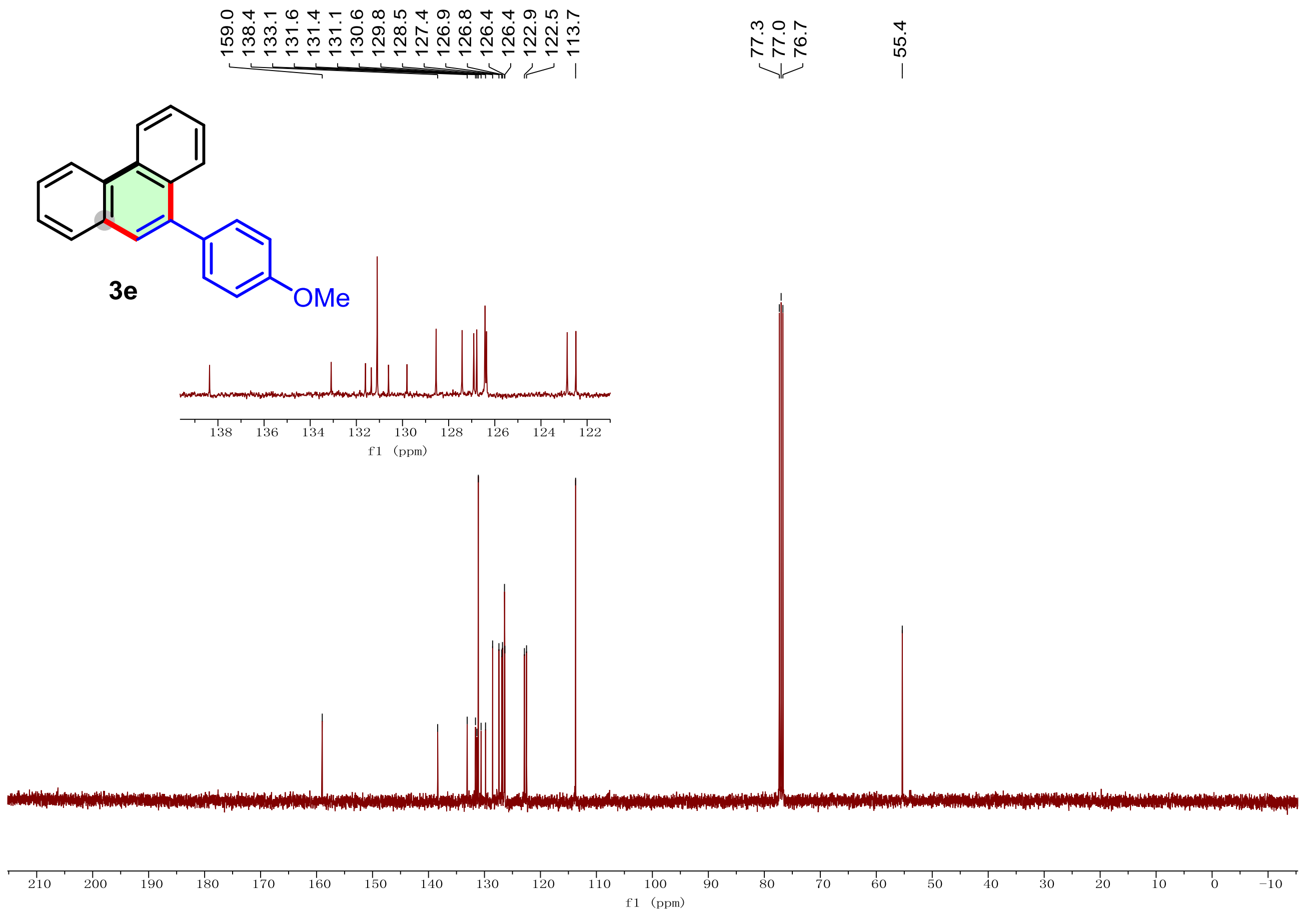

Supplementary Fig. 27. ^{1}H NMR of 3f(400 MHz, $CDCl_3$)

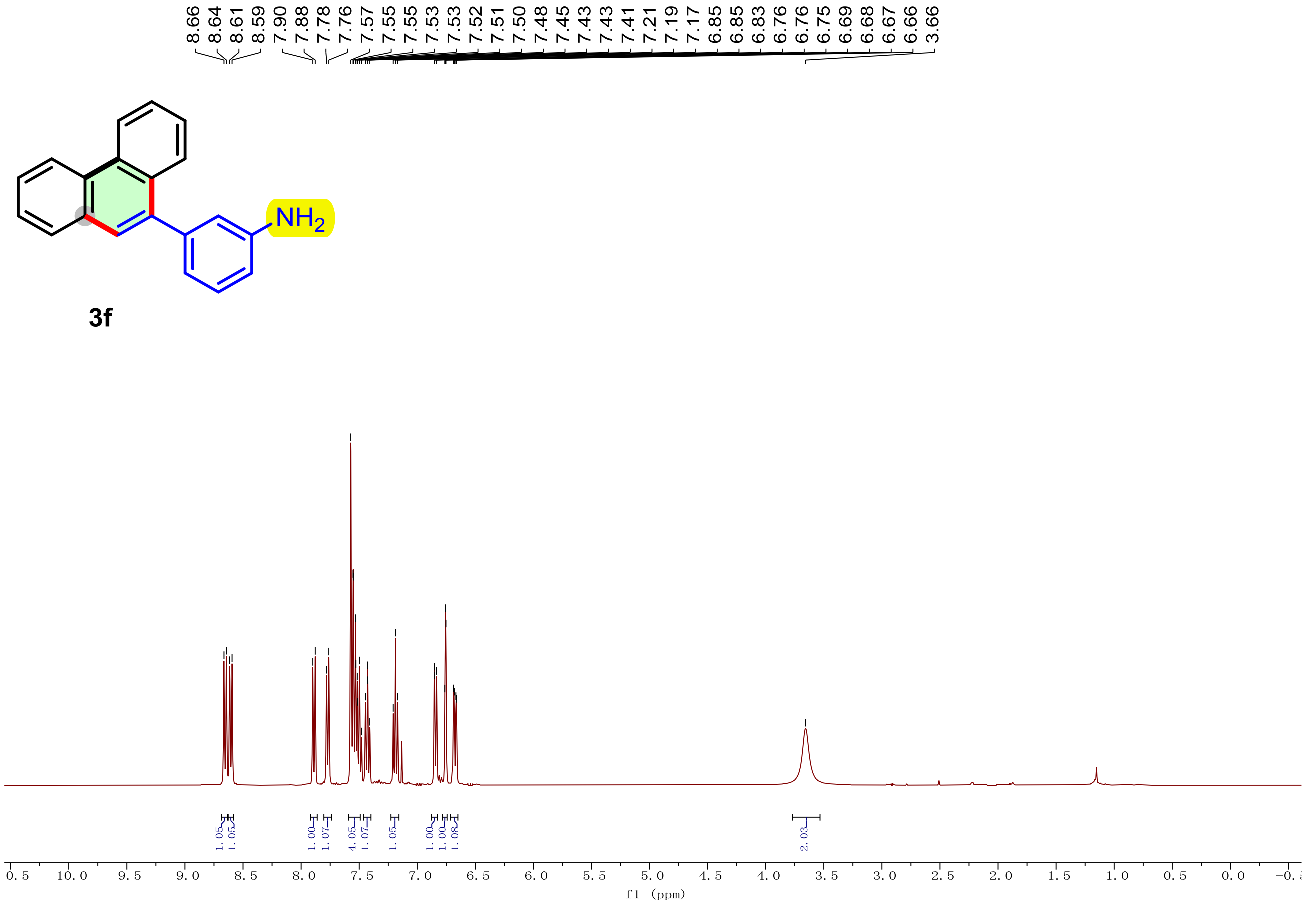

Supplementary Fig. 28. ^{13}C NMR of 3f (101 MHz, $CDCl_3$)

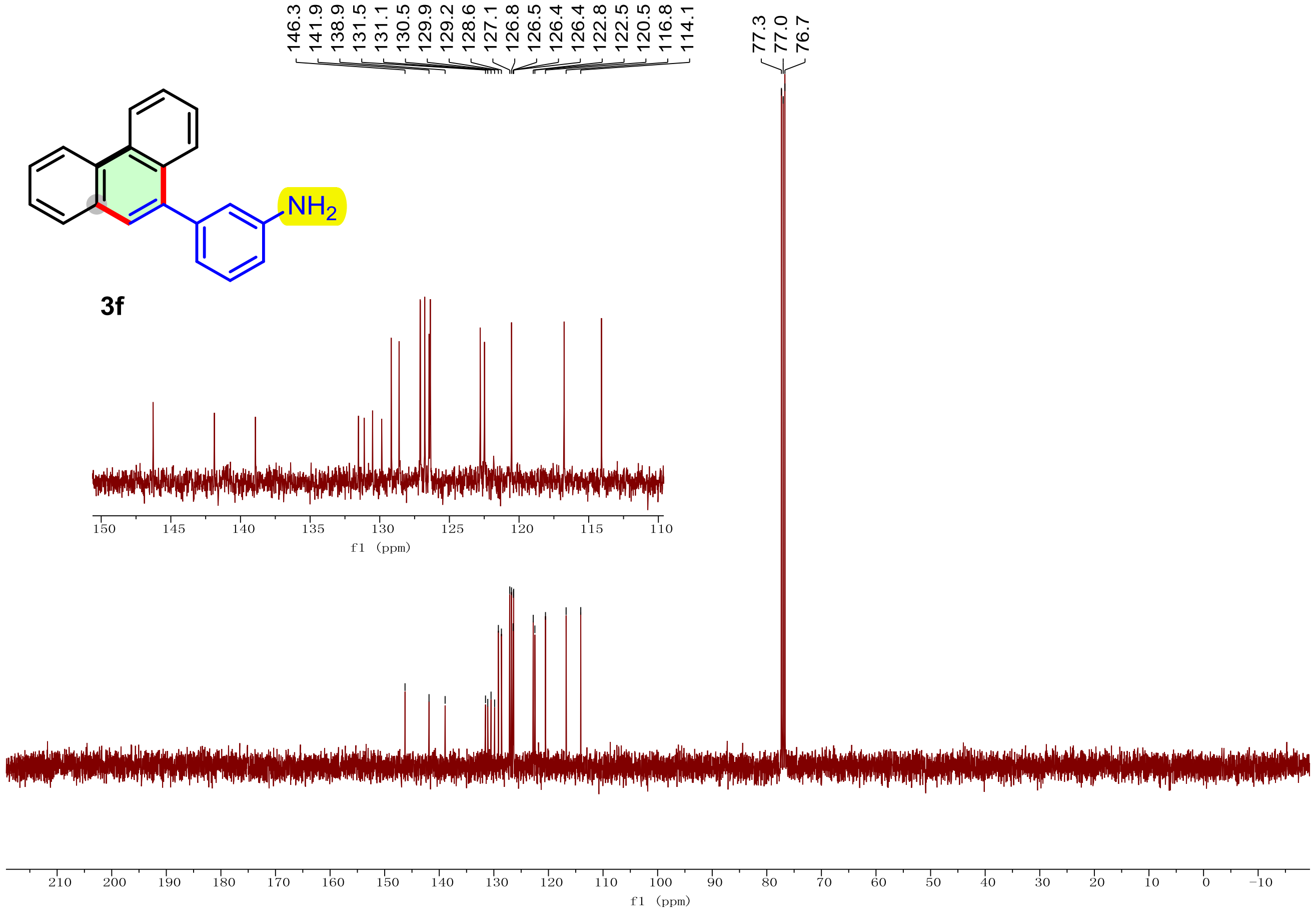

Supplementary Fig. 29. ¹H NMR of 3g (400 MHz, CDCl₃)

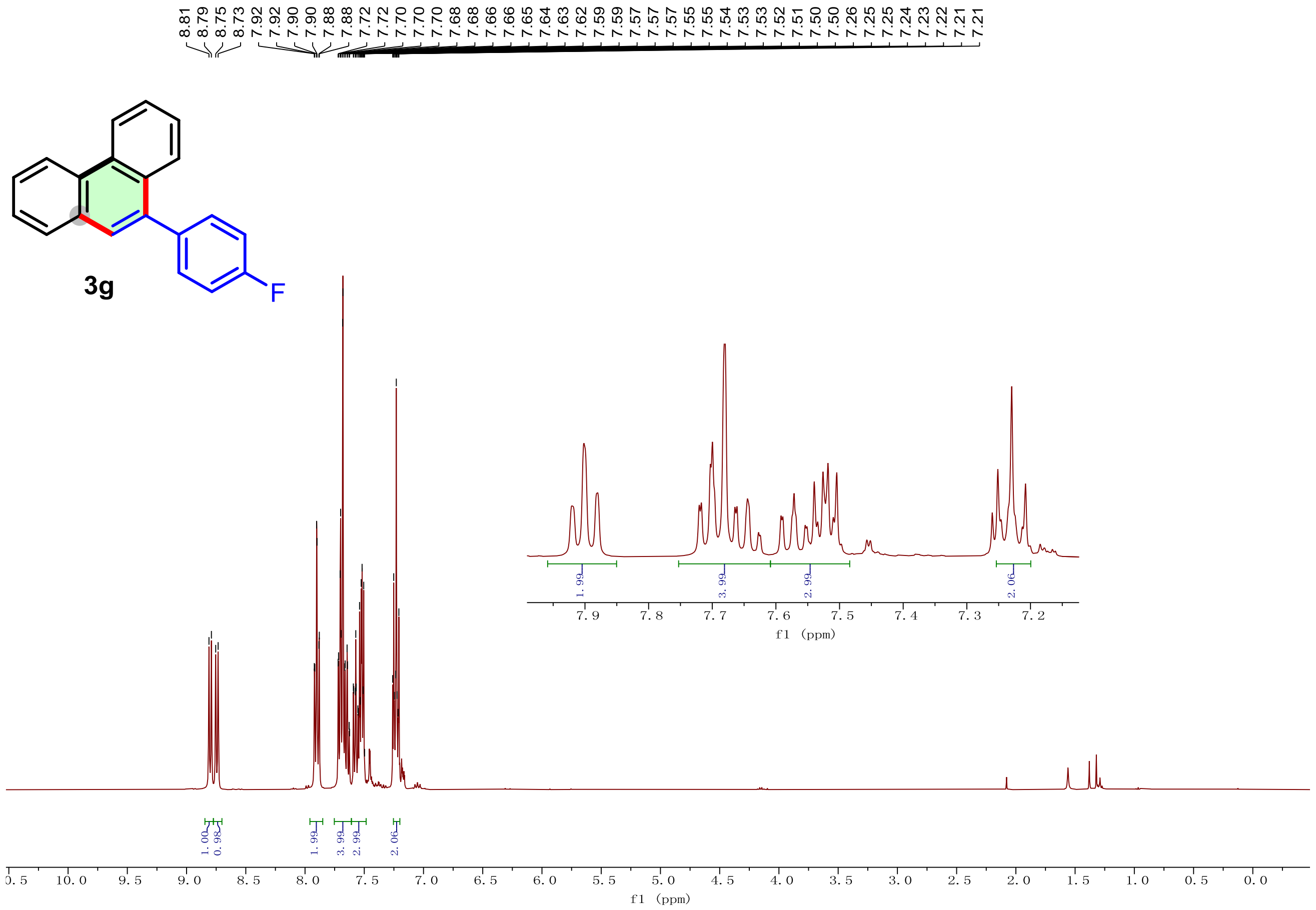

Supplementary Fig. 30. ¹³C NMR of 3g (101 MHz, CDCl₃)

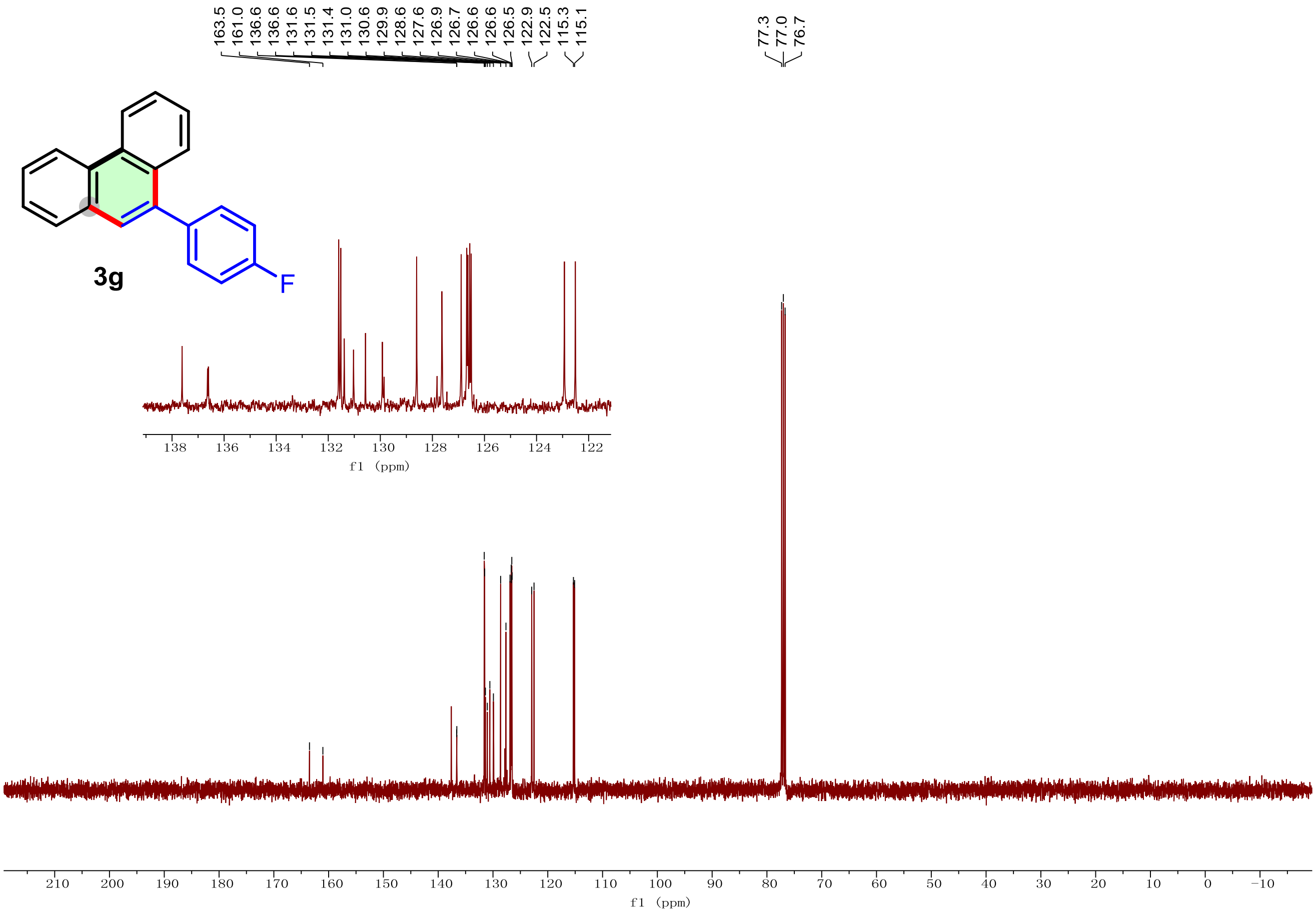

Supplementary Fig. 31. ^{19}F NMR of 3g (282 MHz, $CDCl_3$)

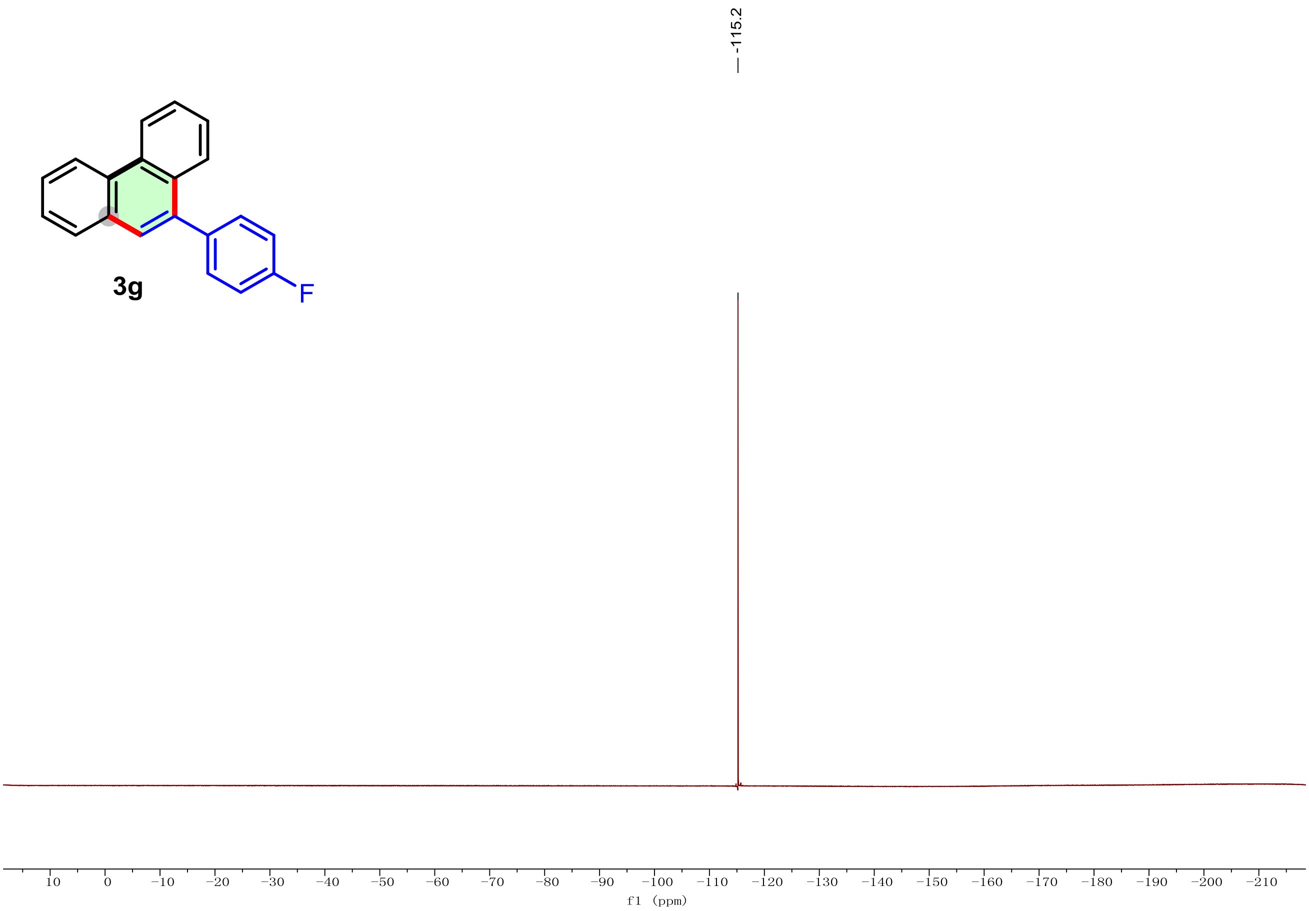

Supplementary Fig. 32. ^{1}H NMR of 3h (400 MHz, $CDCl_3$)

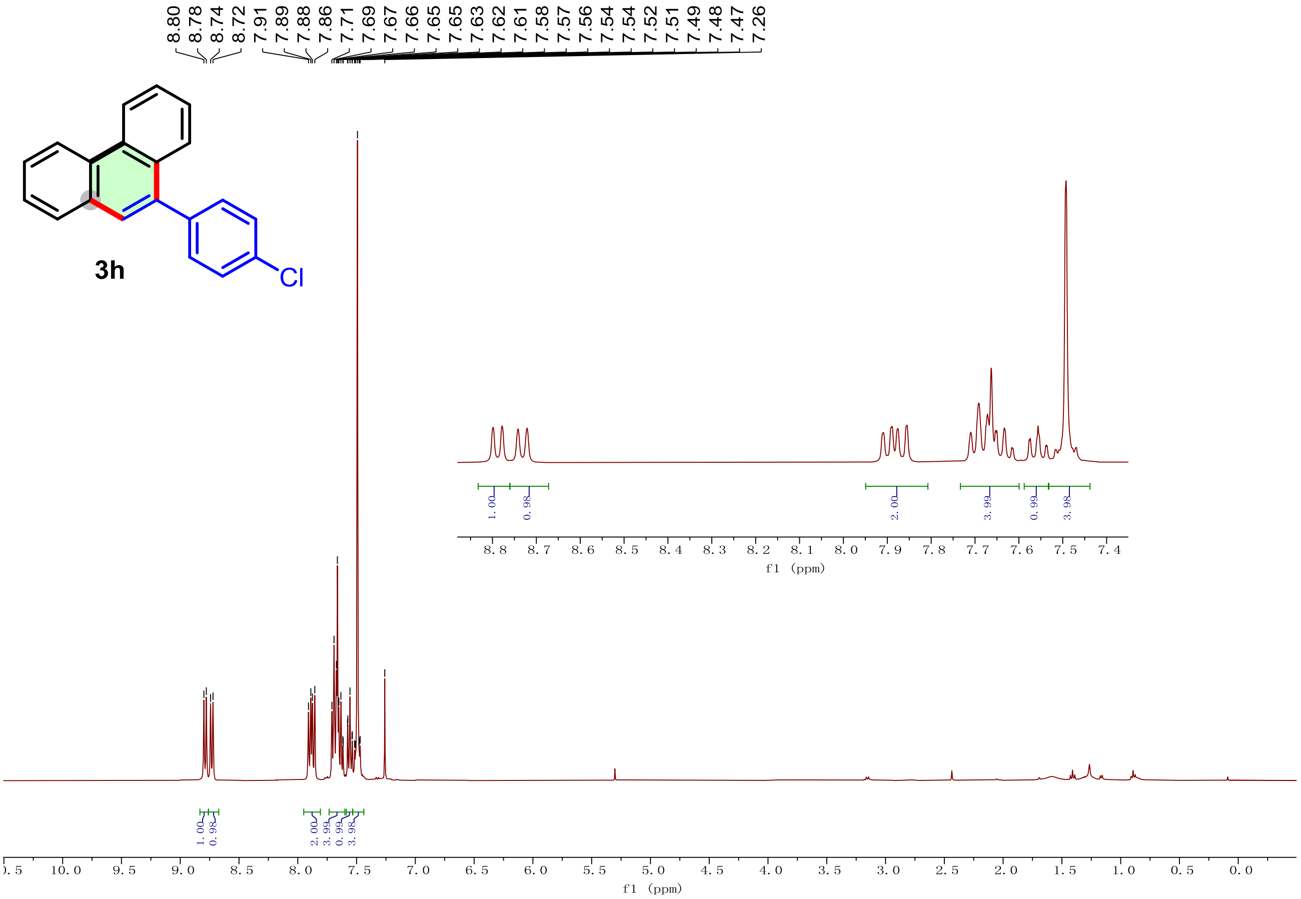

Supplementary Fig. 33. ^{13}C NMR of 3h (101 MHz, $CDCl_3$)

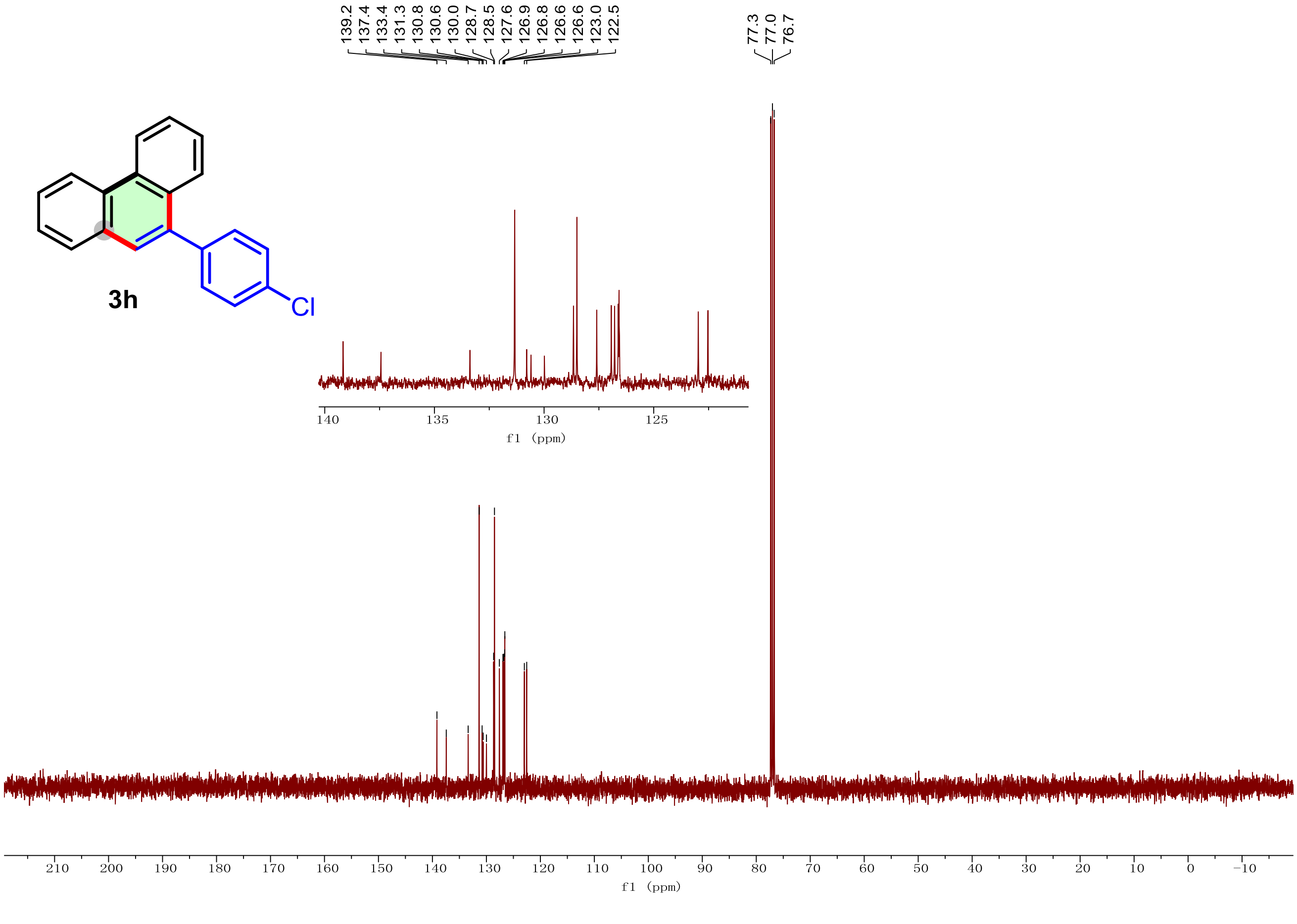

Supplementary Fig. 34. ^{1}H NMR of 3i (300 MHz, $CDCl_3$)

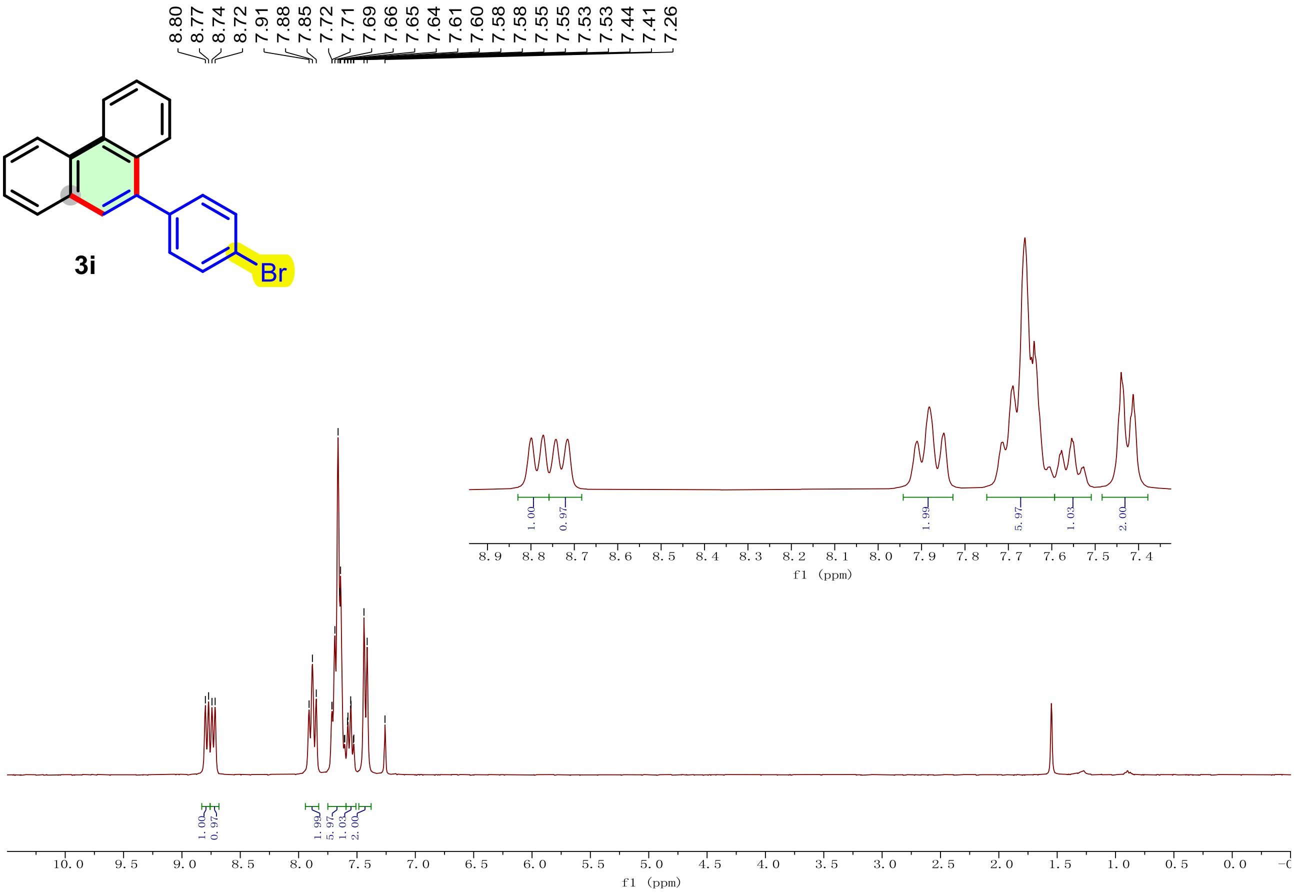

Supplementary Fig. 35. ^{13}C NMR of 3i (101 MHz, $CDCl_3$)

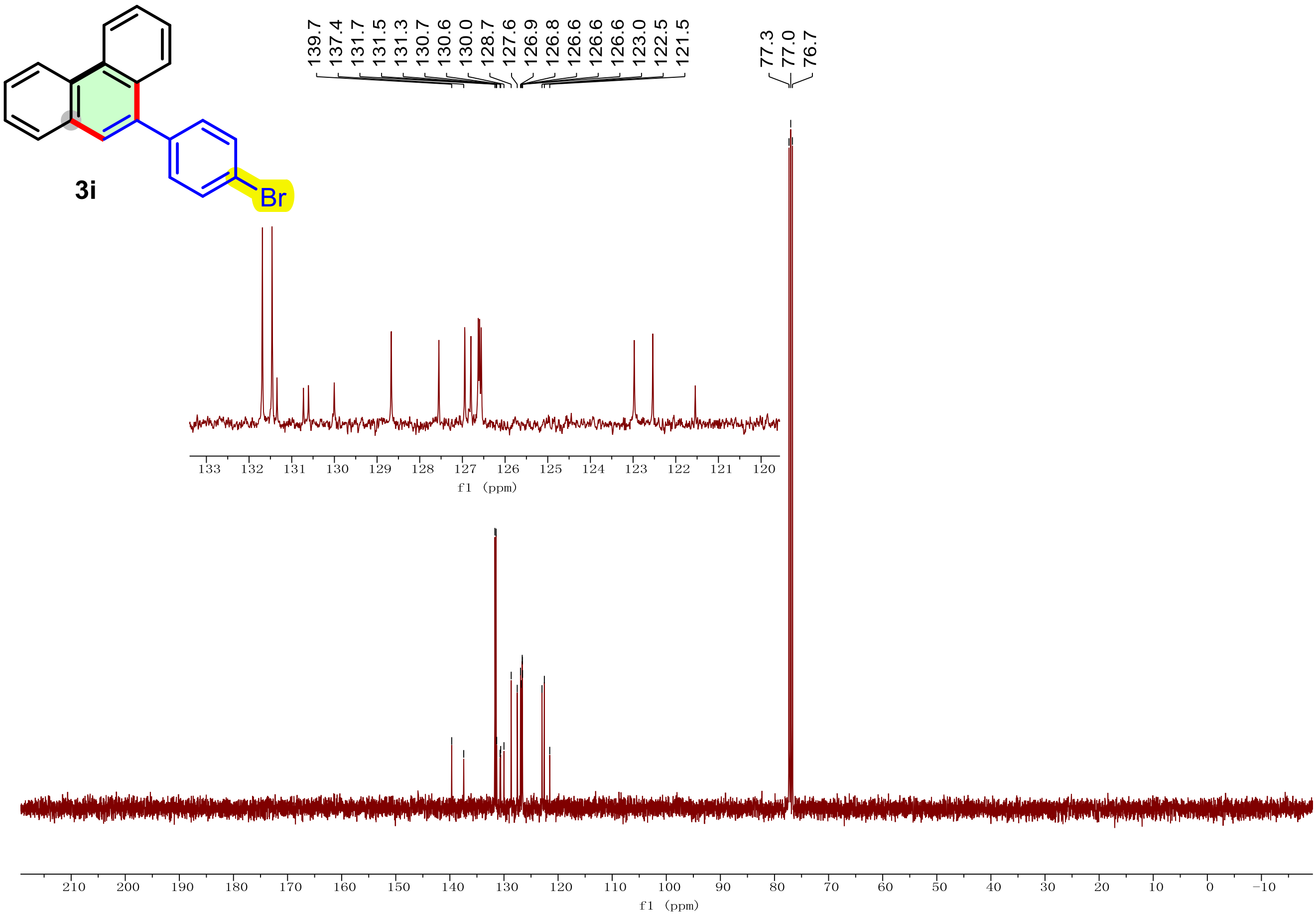

Supplementary Fig. 36. ^{1}H NMR of 3j (400 MHz, $CDCl_3$)

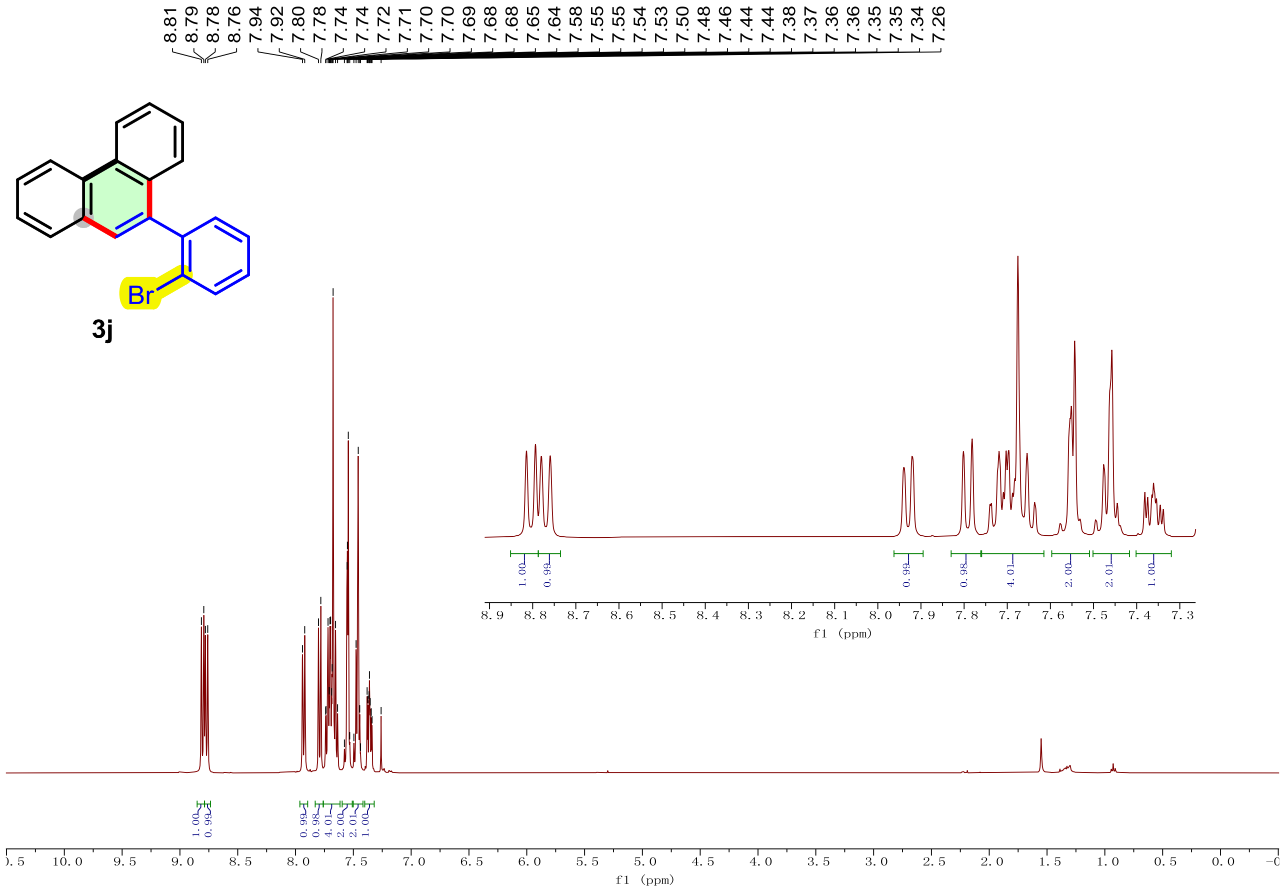

Supplementary Fig. 37. ^{13}C NMR of 3j (101 MHz, $CDCl_3$)

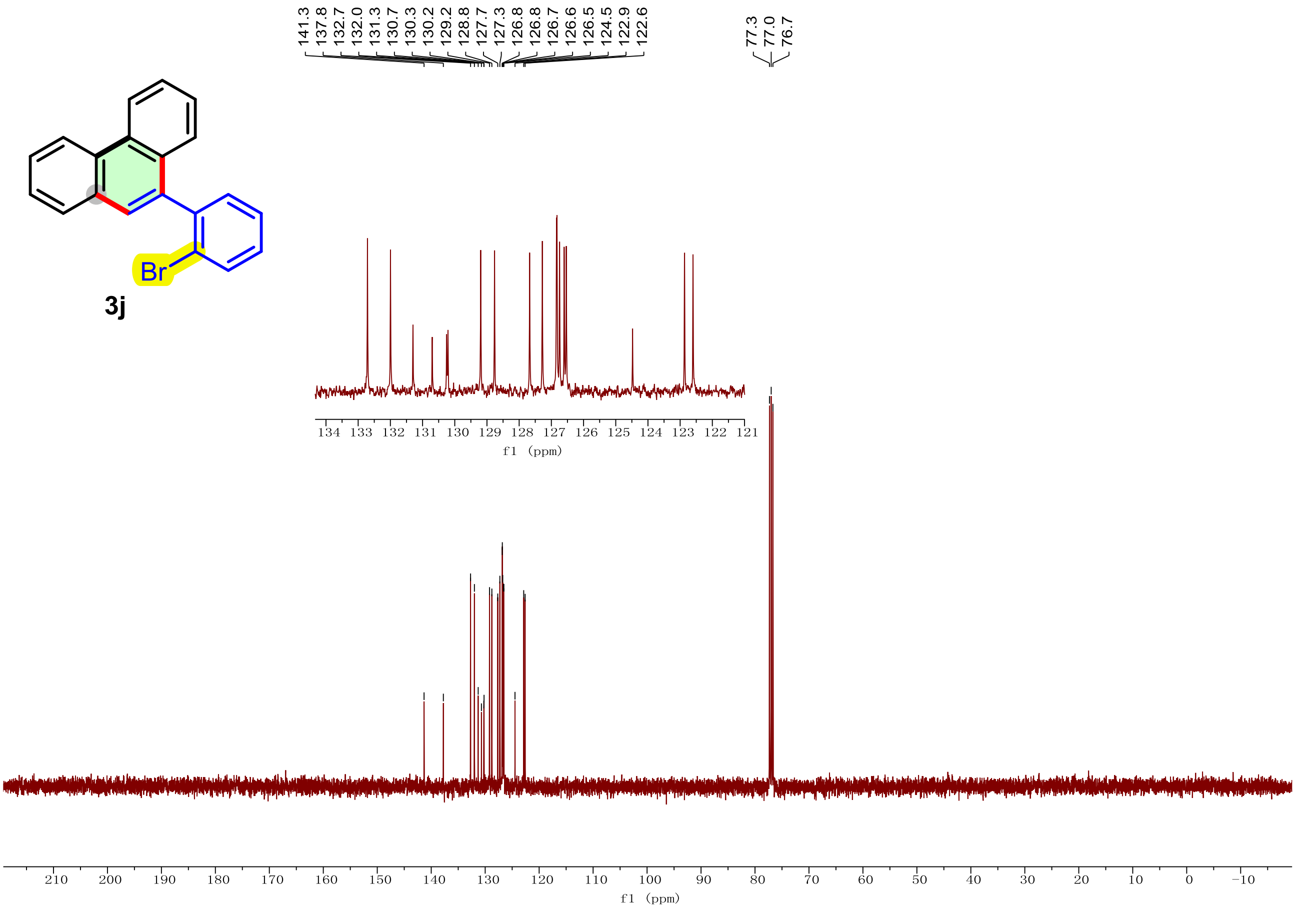

Supplementary Fig. 38. ^{1}H NMR of 3k (600 MHz, $CDCl_3$)

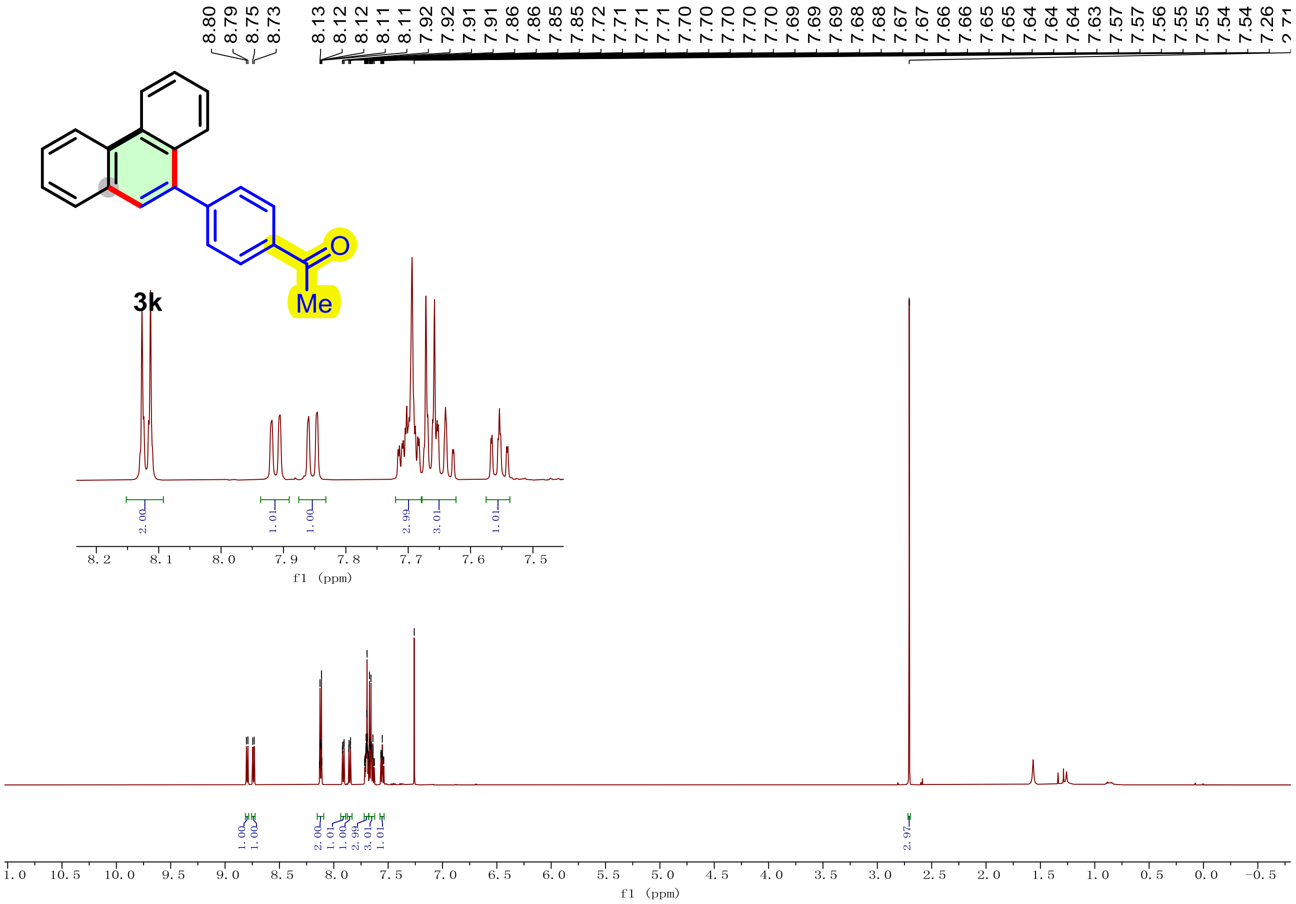

Supplementary Fig. 39. ^{13}C NMR of 3k (151 MHz, $CDCl_3$)

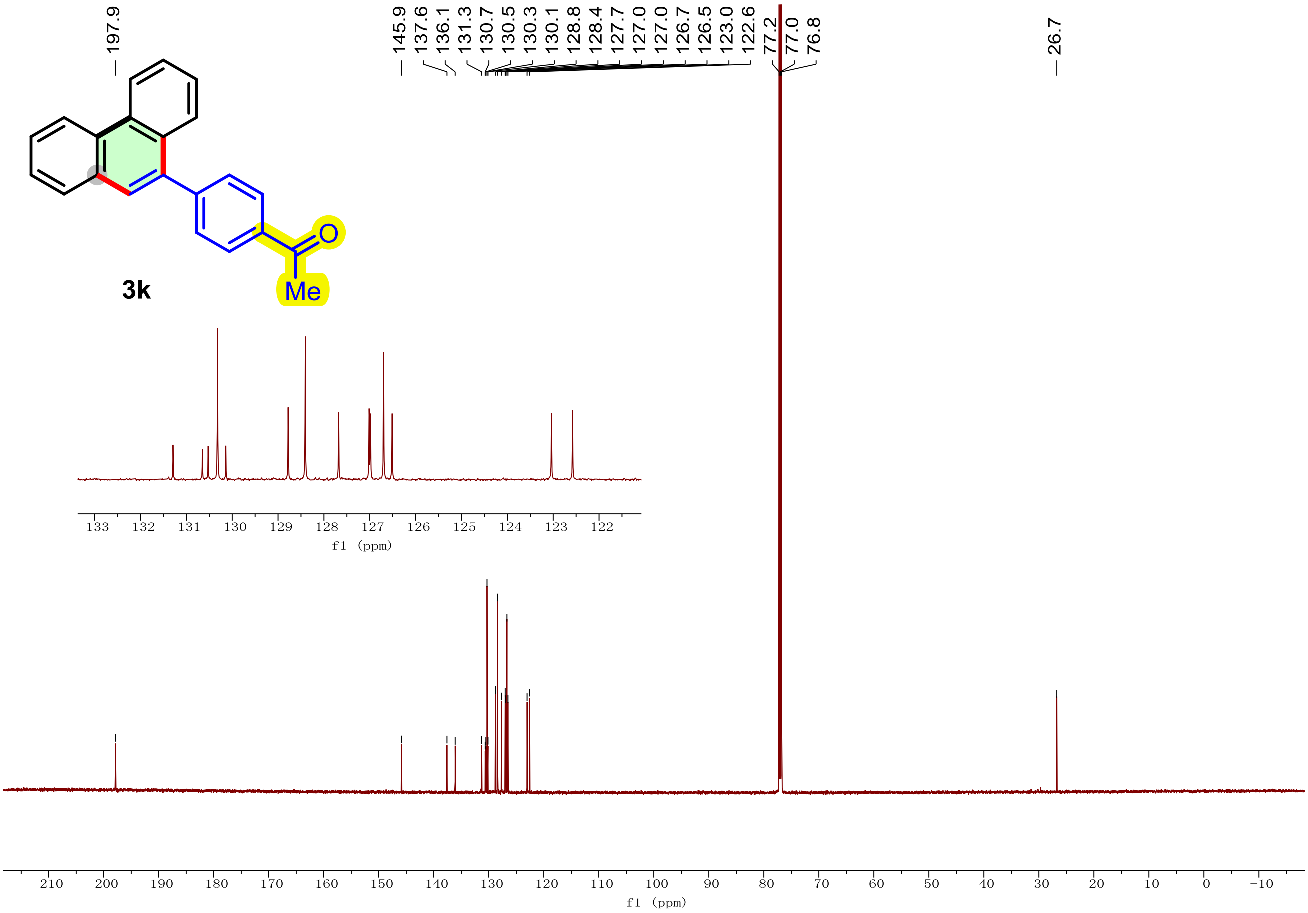

Supplementary Fig. 40. ^{1}H NMR of 3l (400 MHz, $CDCl_3$)

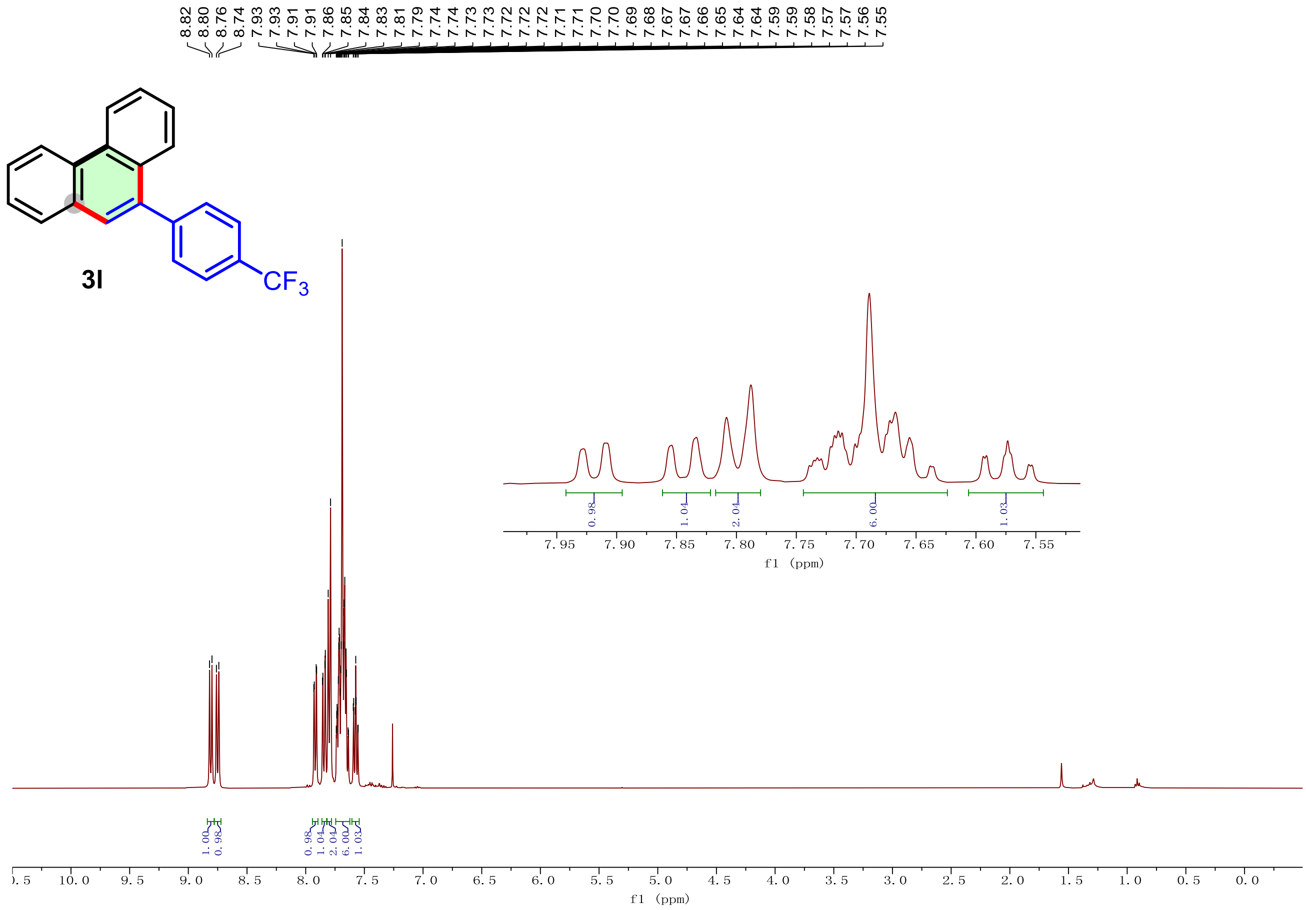

Supplementary Fig. 41. ^{13}C NMR of 3l (101 MHz, $CDCl_3$)

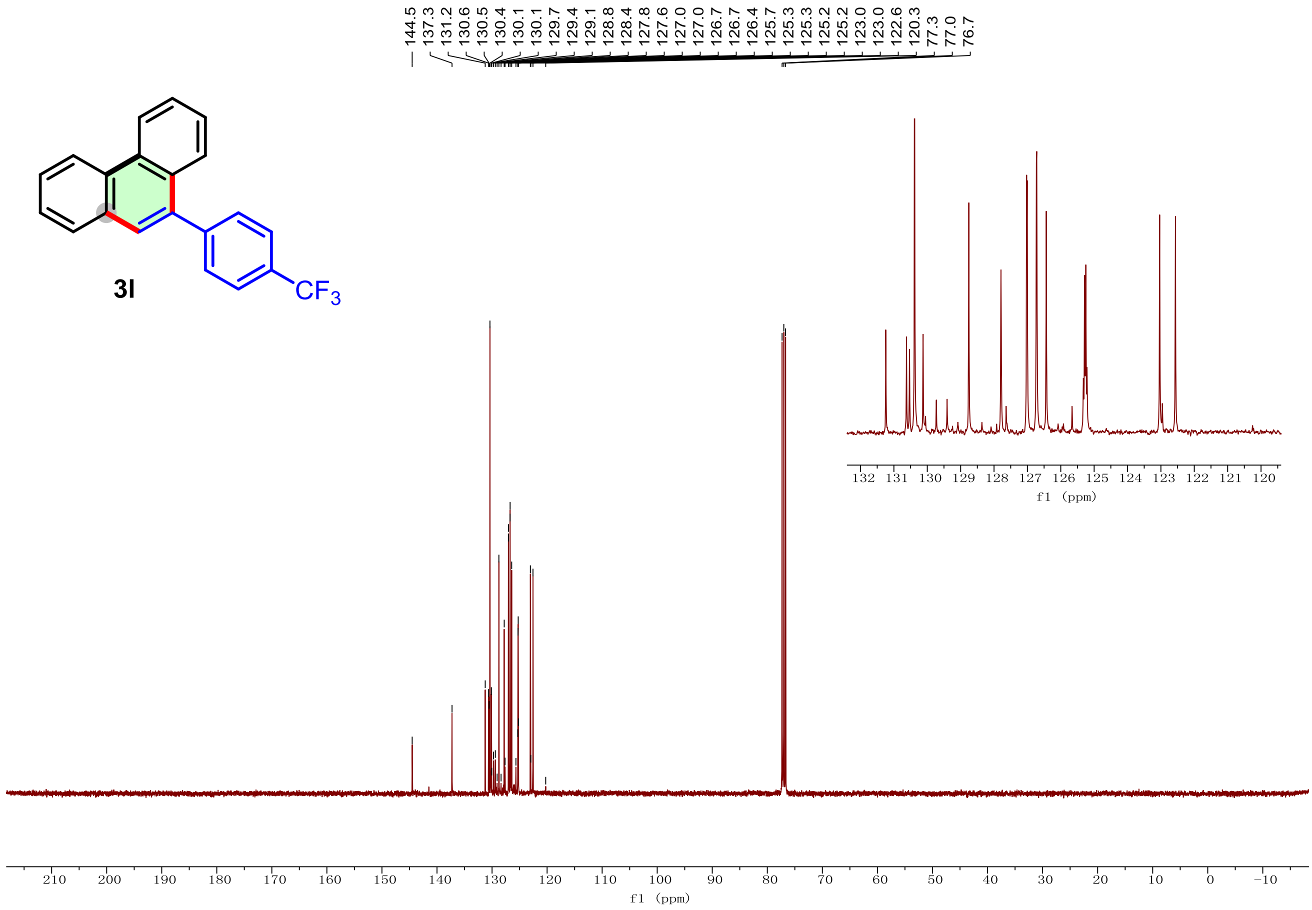

Supplementary Fig.42. ^{19}F NMR of 3l (282 MHz, CDCl$_3$)

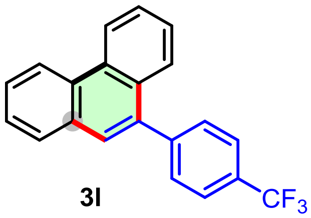

3l

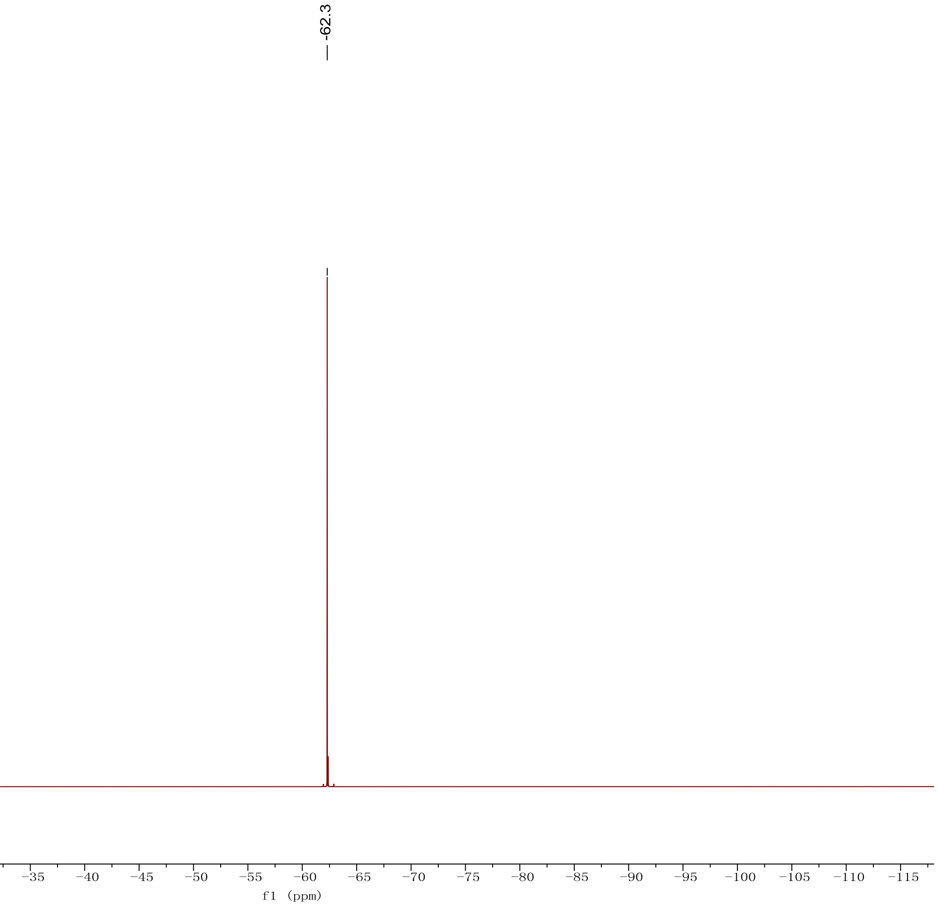

Supplementary Fig. 43. ^{1}H NMR of 3m (400 MHz, $CDCl_3$)

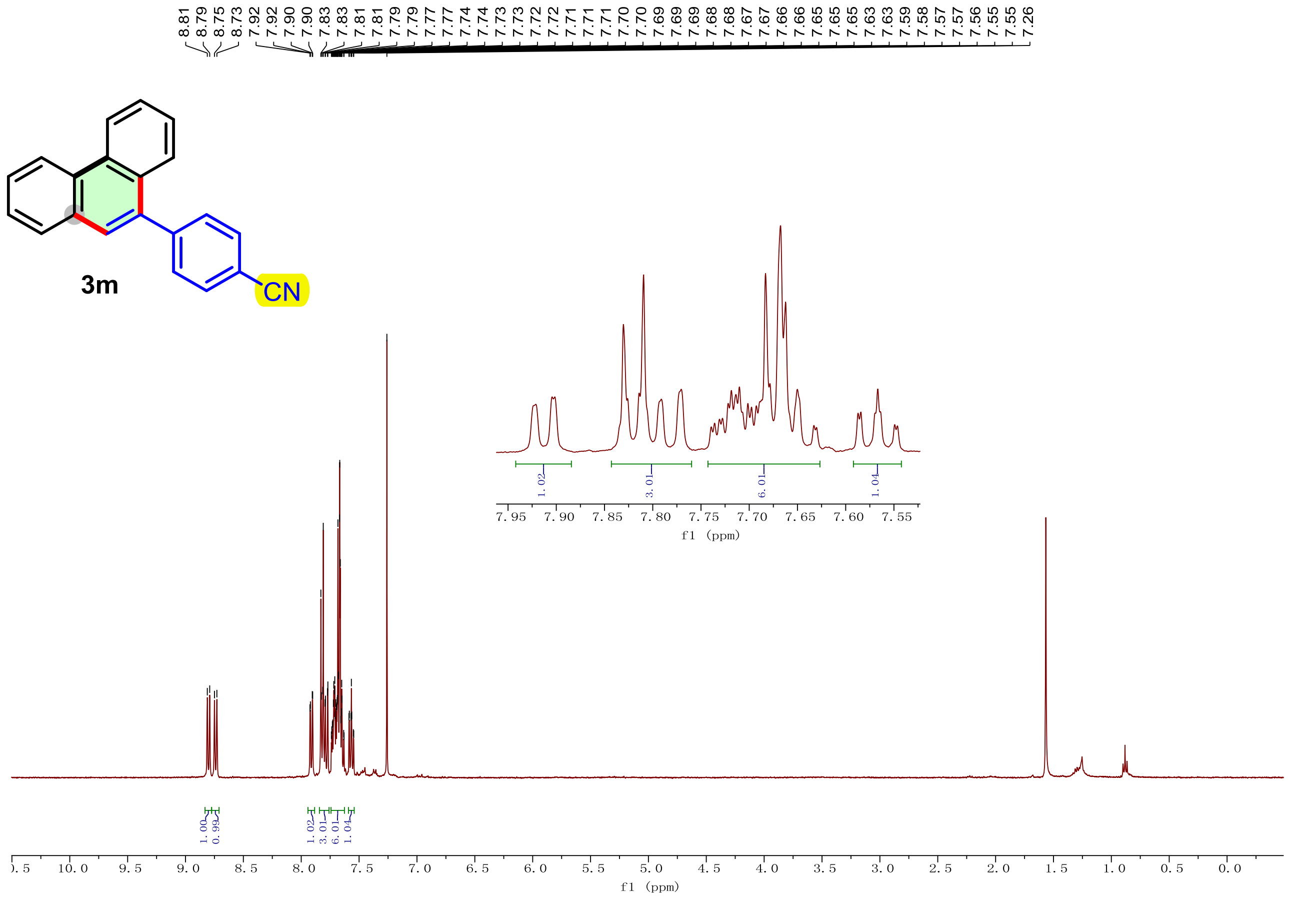

Supplementary Fig. 44. ^{13}C NMR of 3m (101 MHz, $CDCl_3$)

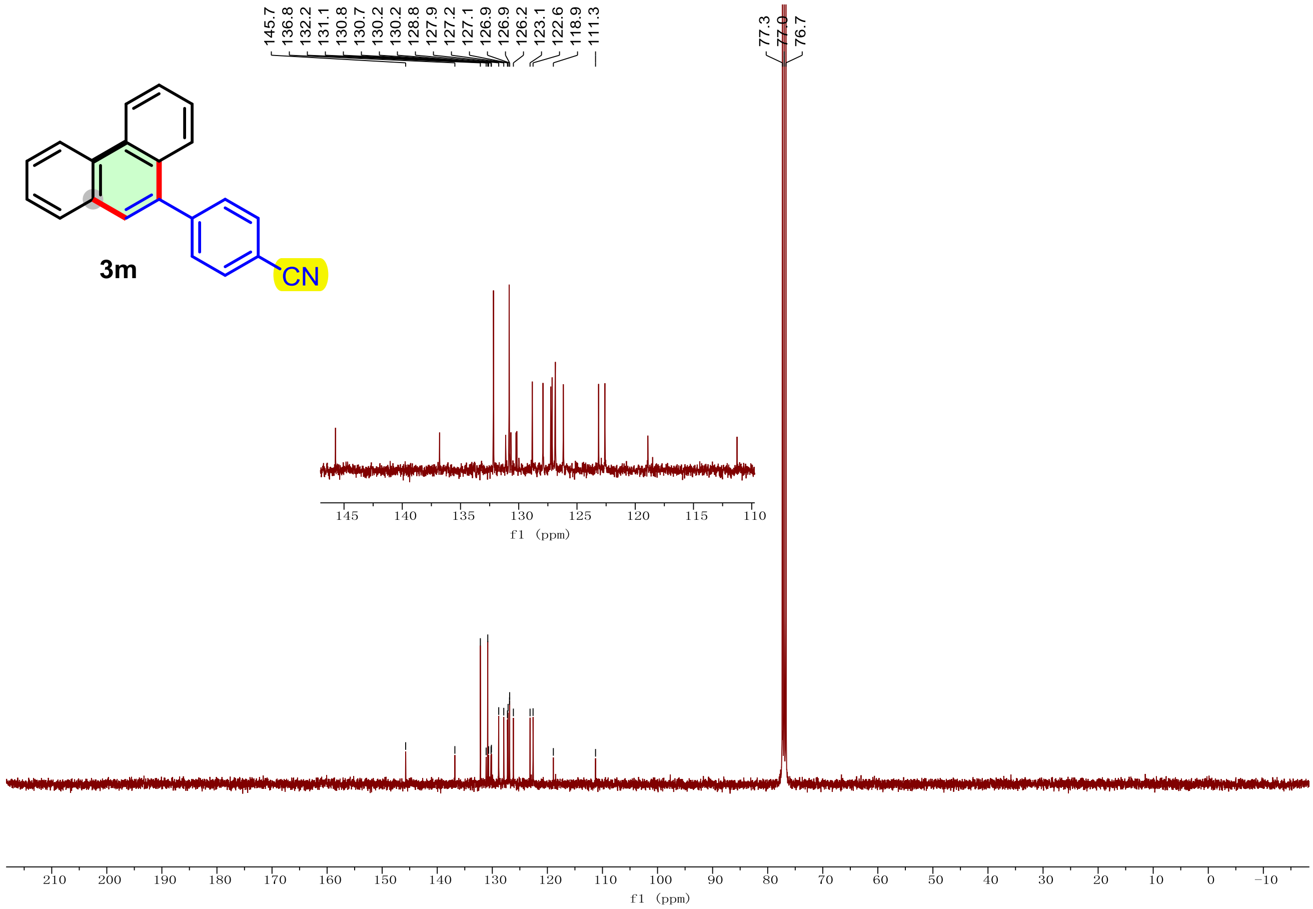

Supplementary Fig. 45. ¹H NMR of 3n (400 MHz, $CDCl_3$)

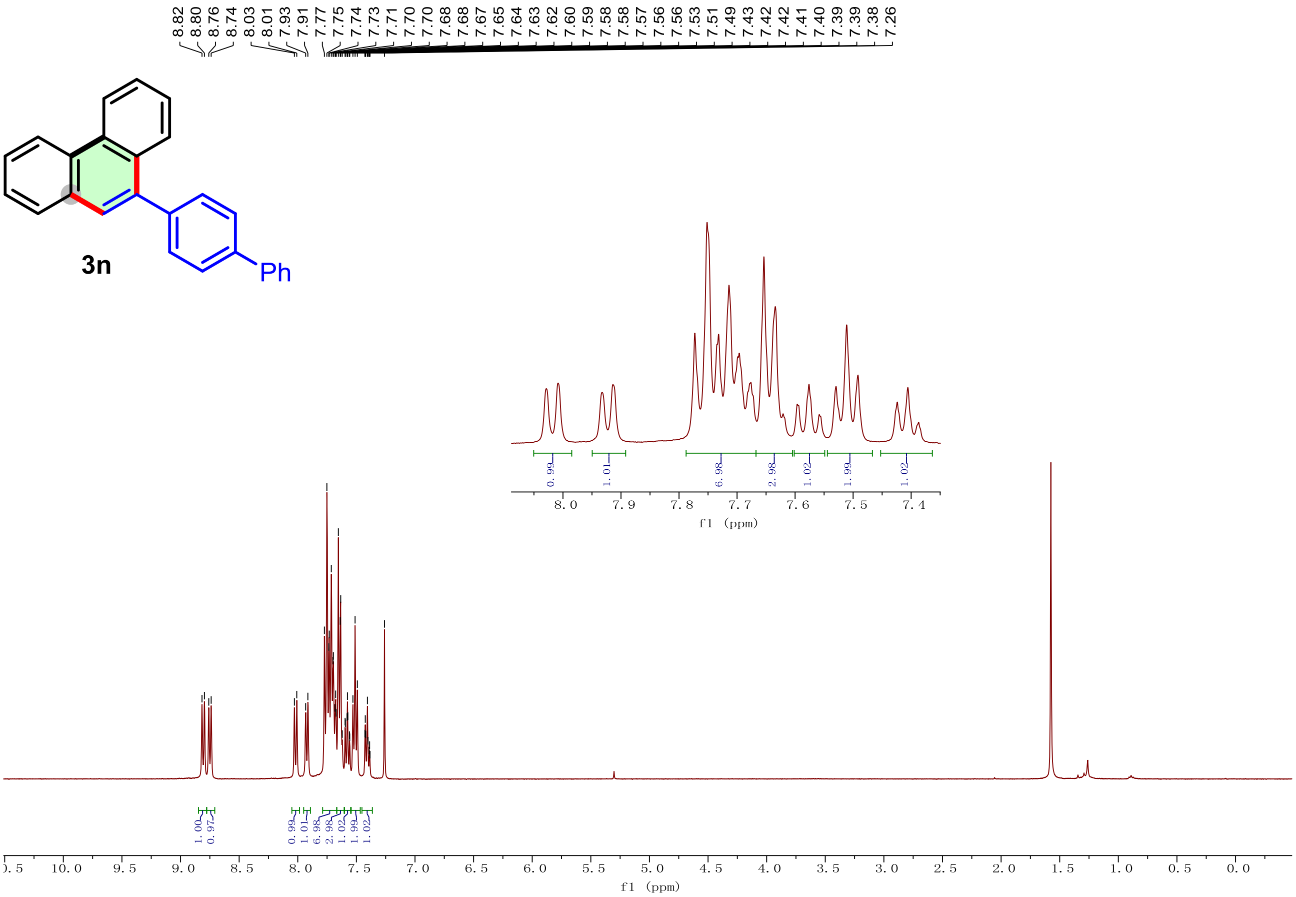

Supplementary Fig. 46. ¹³C NMR of 3n (101 MHz, $CDCl_3$)

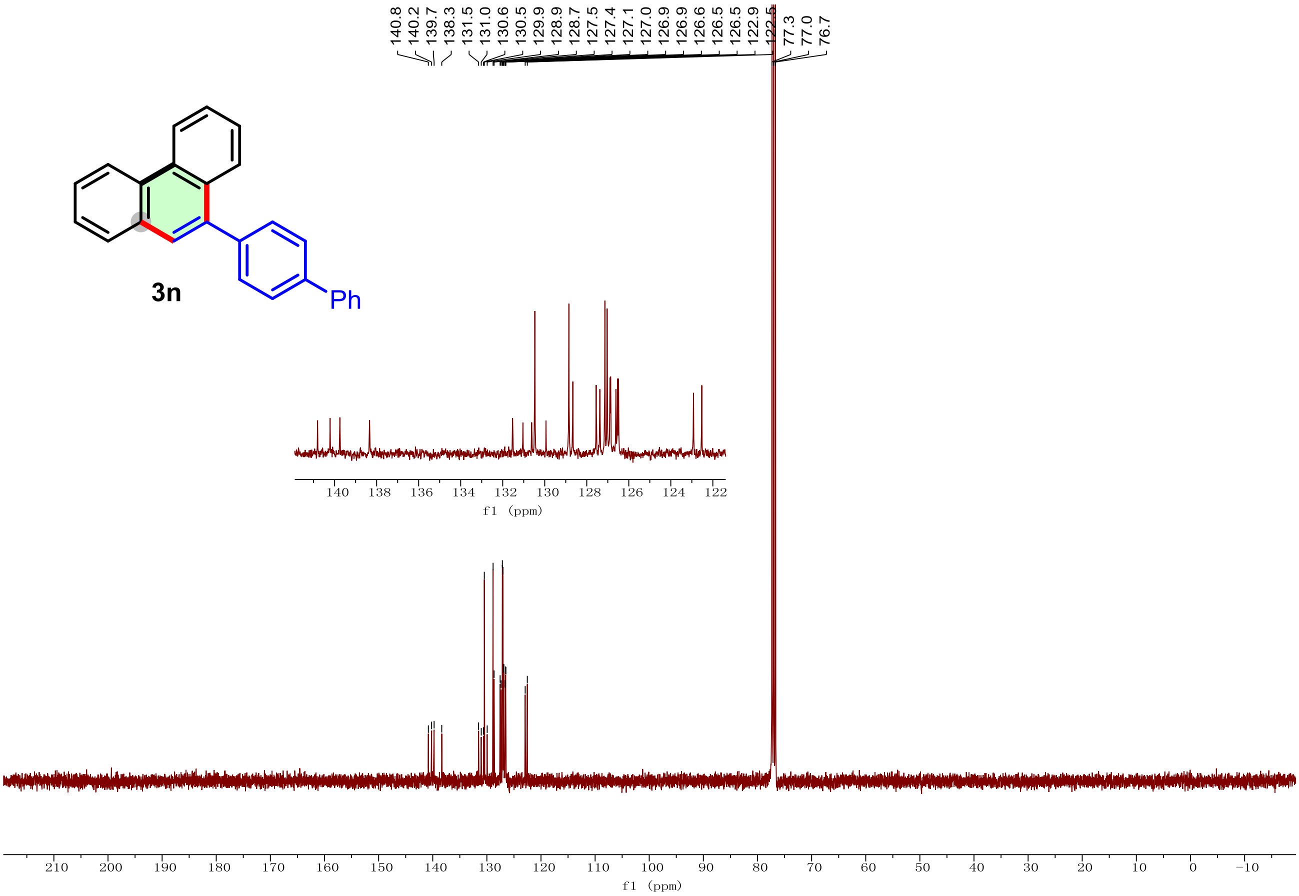

Supplementary Fig. 47. ^{1}H NMR of 3o (400 MHz, $CDCl_3$)

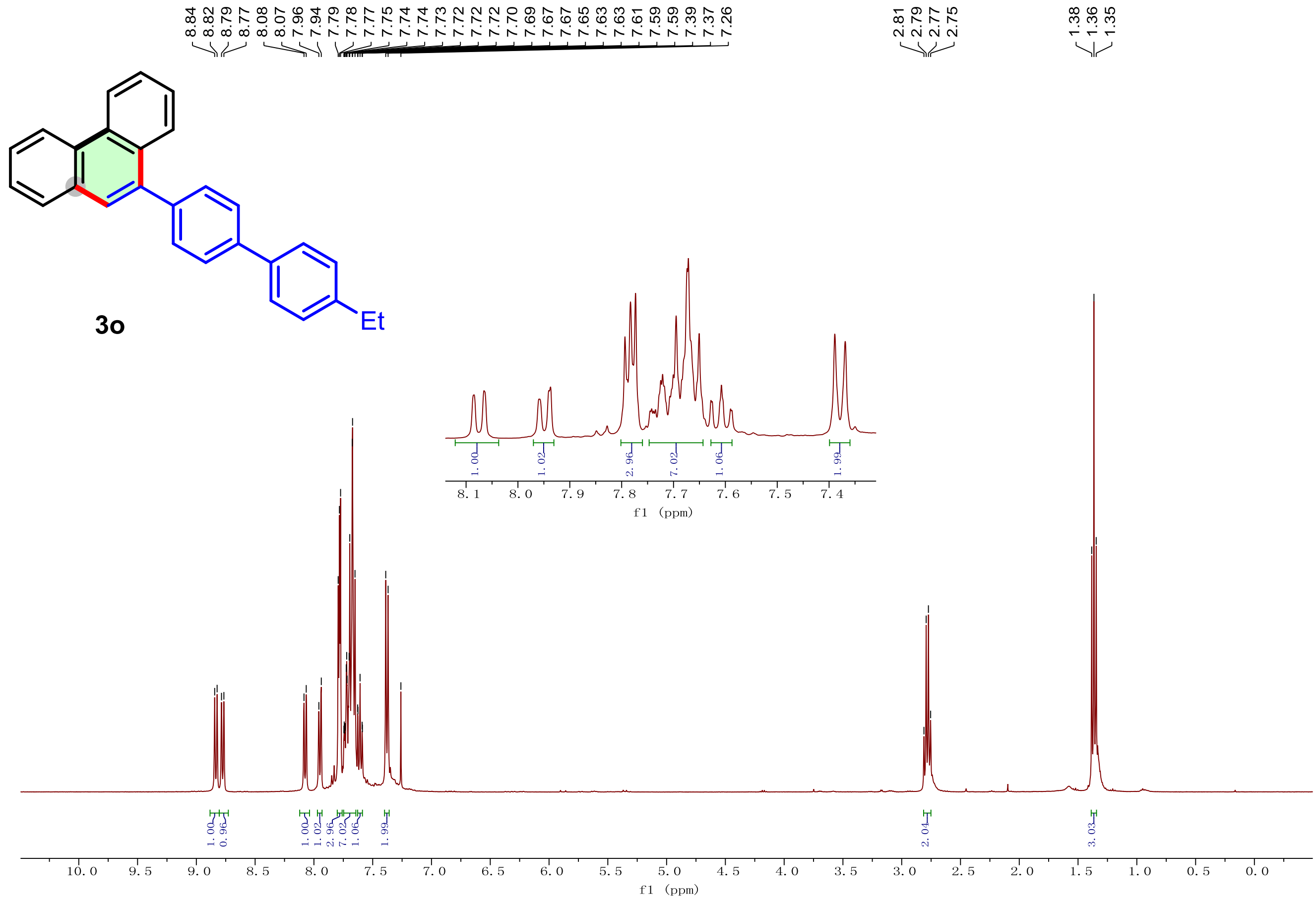

Supplementary Fig. 48. ^{13}C NMR of 3o (101 MHz, $CDCl_3$)

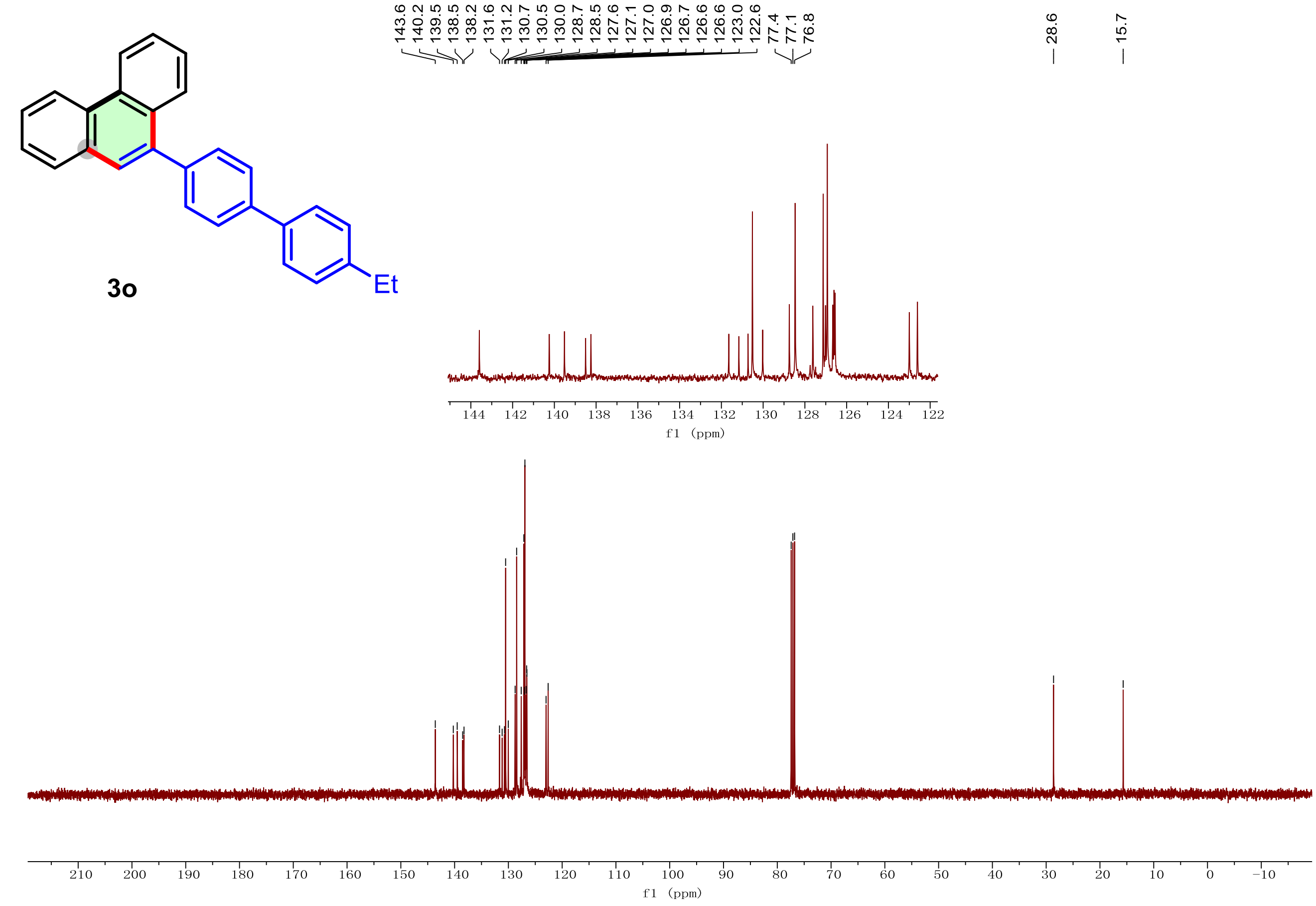

Supplementary Fig. 49. ^{1}H NMR of 3p (600 MHz, $CDCl_3$)

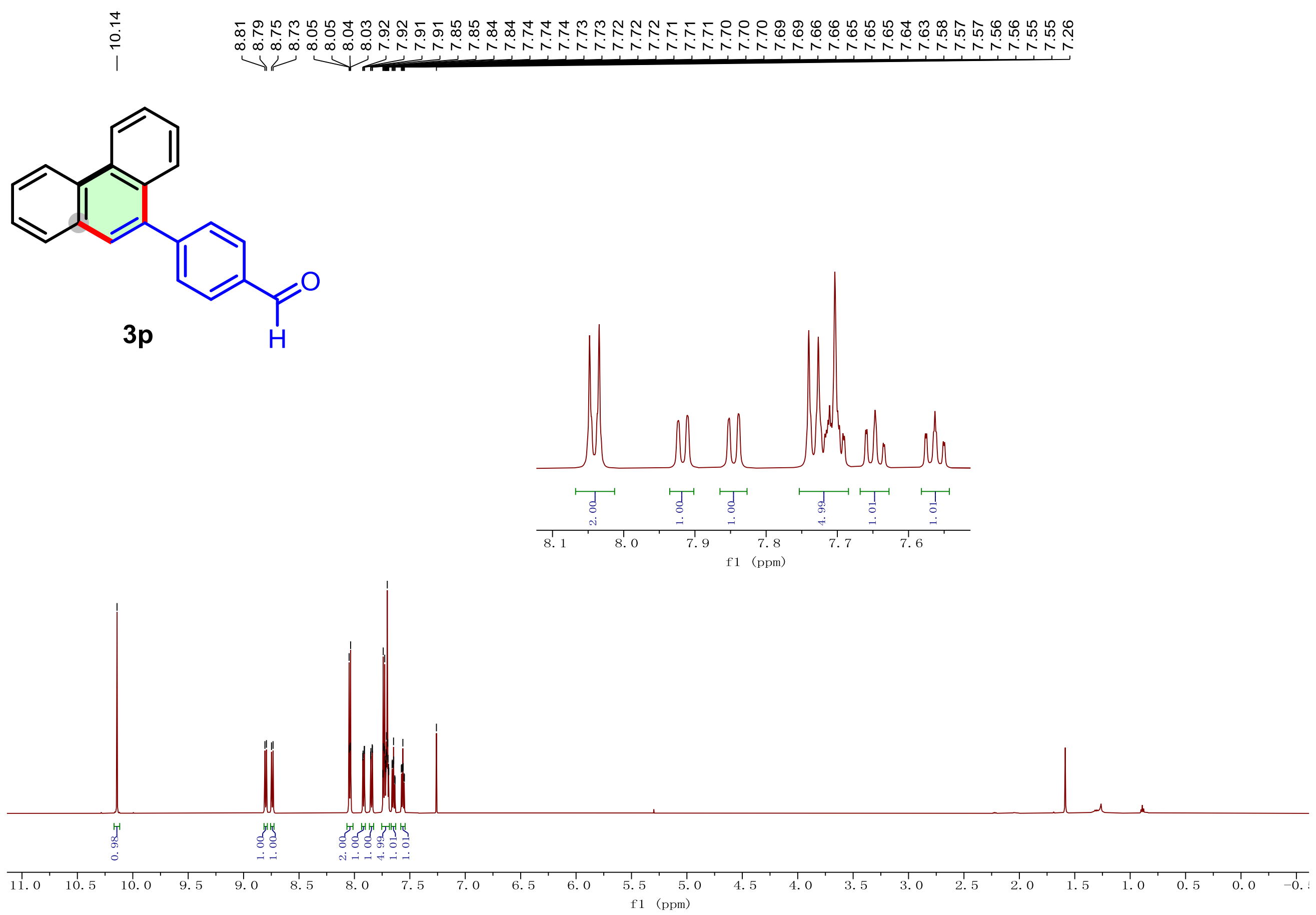

Supplementary Fig. 50. ^{13}C NMR of 3p (151 MHz, $CDCl_3$)

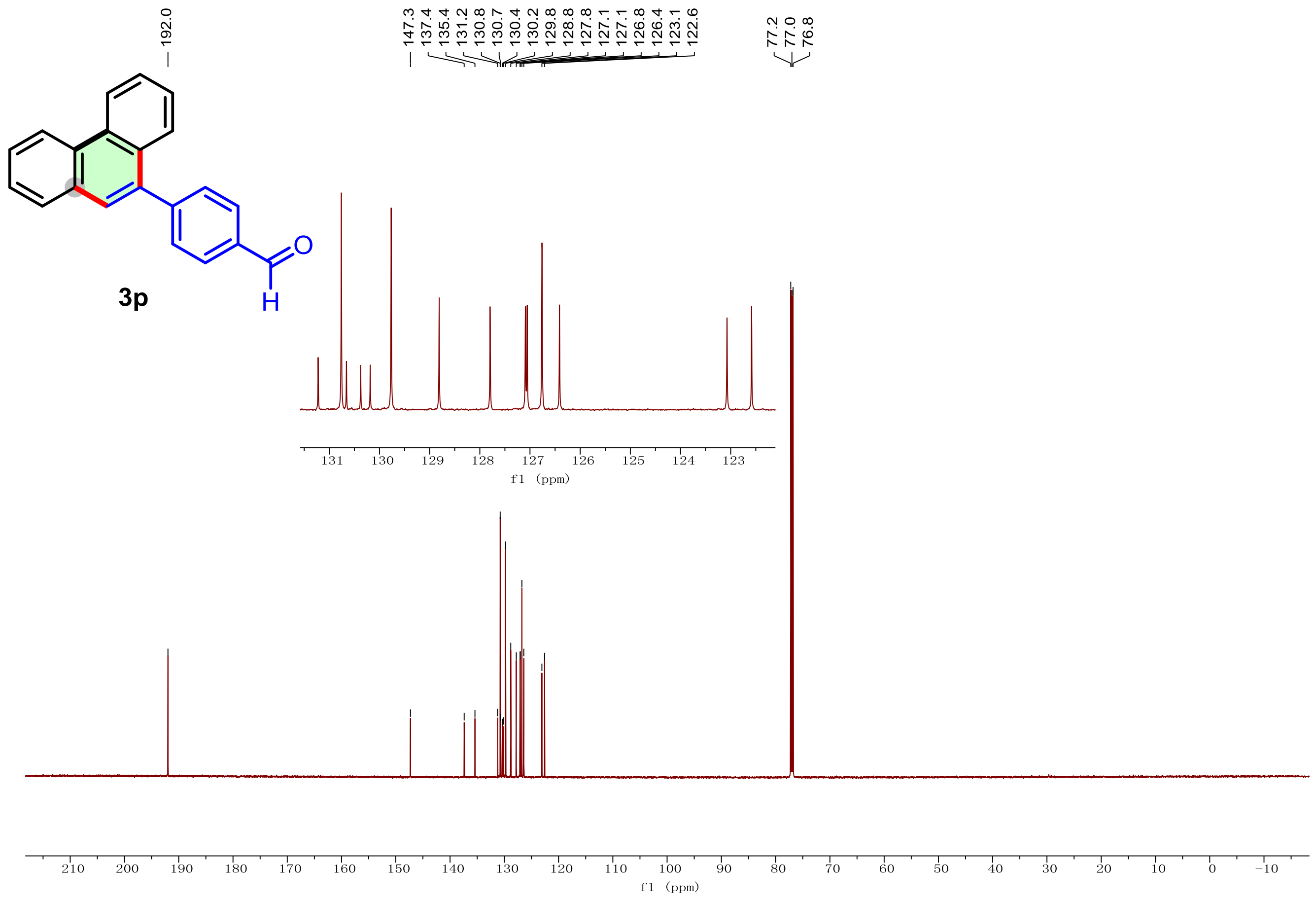

Supplementary Fig. 51. ^{1}H NMR of 3q (600 MHz, CDCl$_3$)

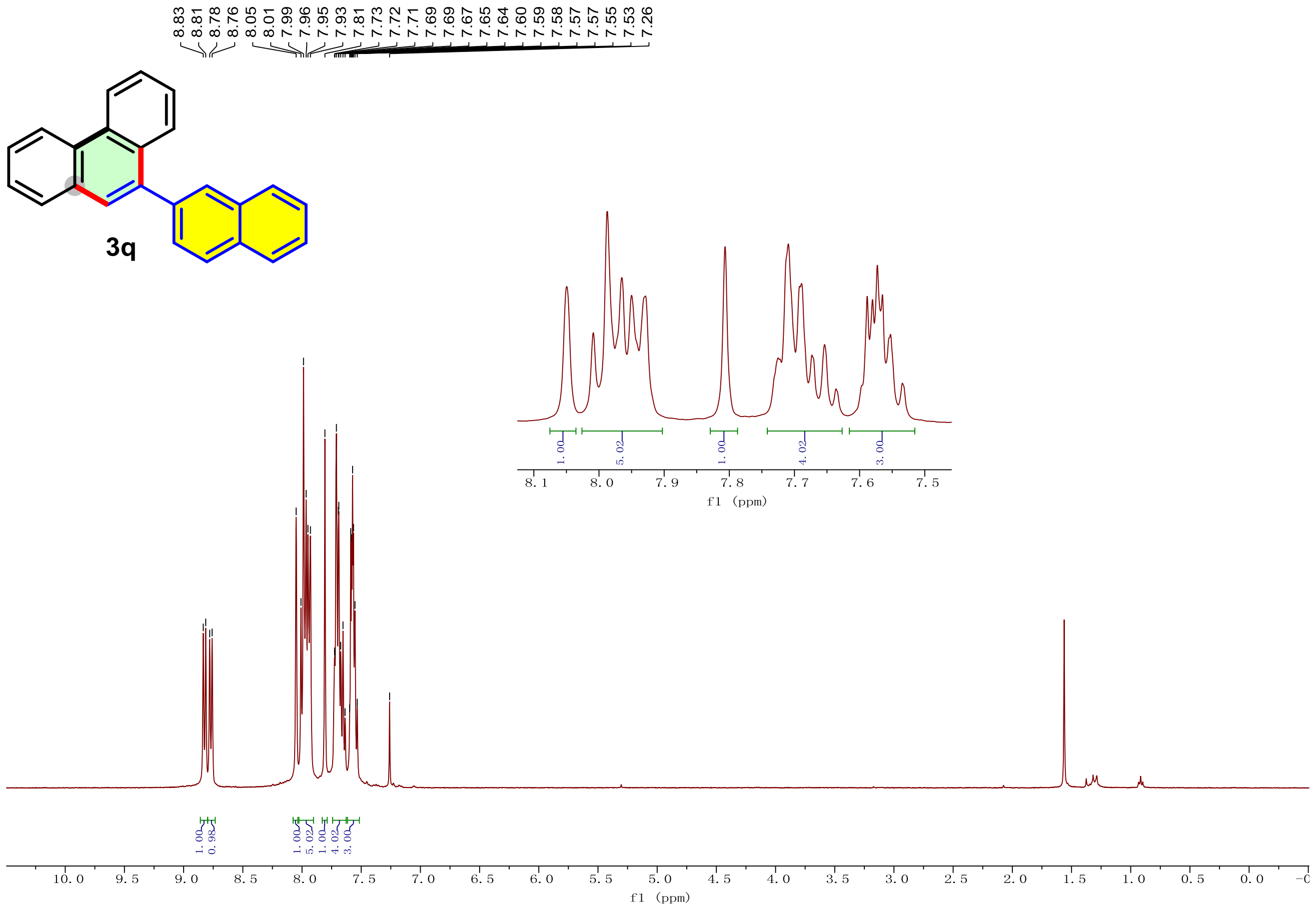

Supplementary Fig. 52. ^{13}C NMR of 3q (101 MHz, CDCl$_3$)

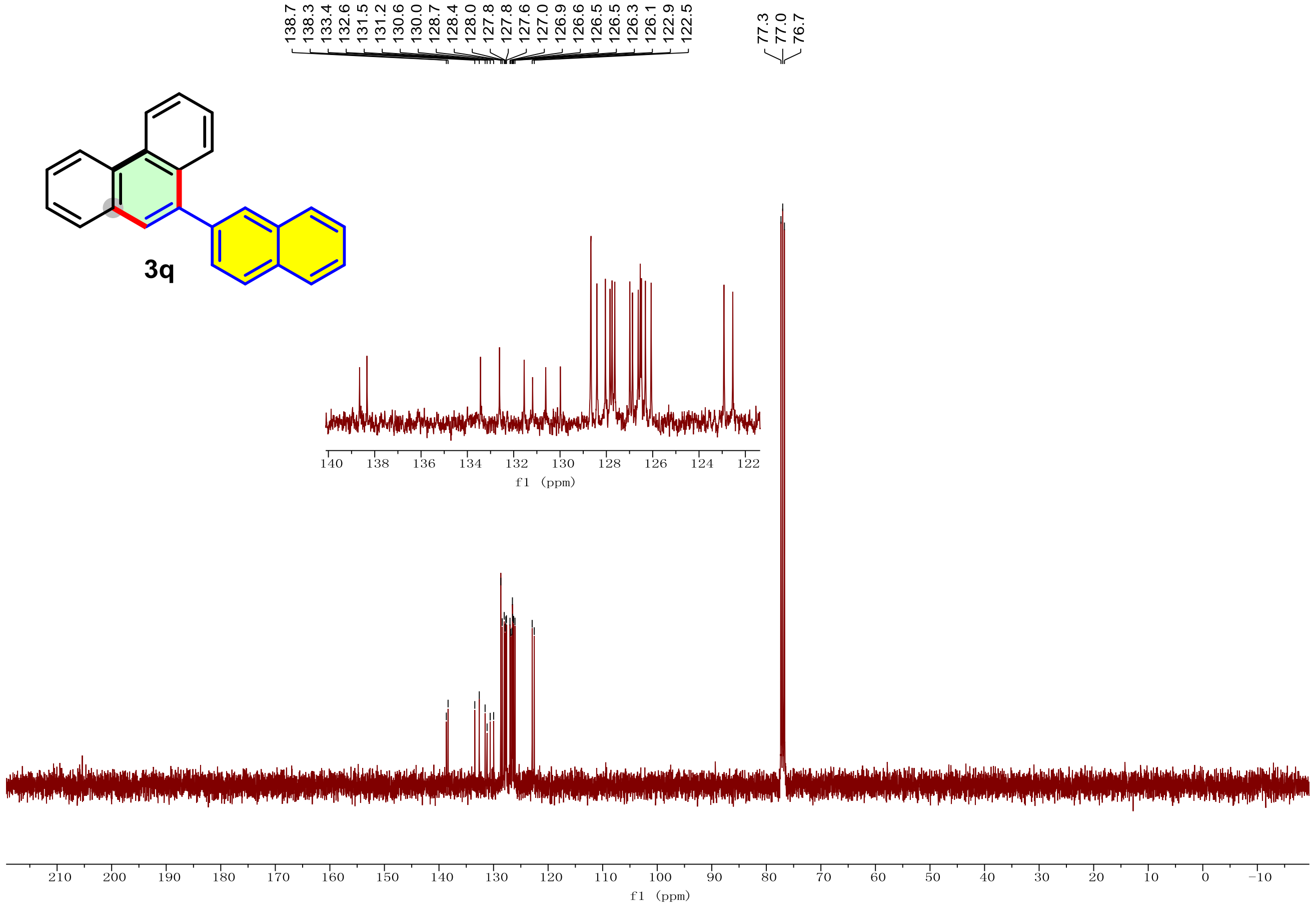

Supplementary Fig. 53. ^{1}H NMR of 3r (400 MHz, $CDCl_3$)

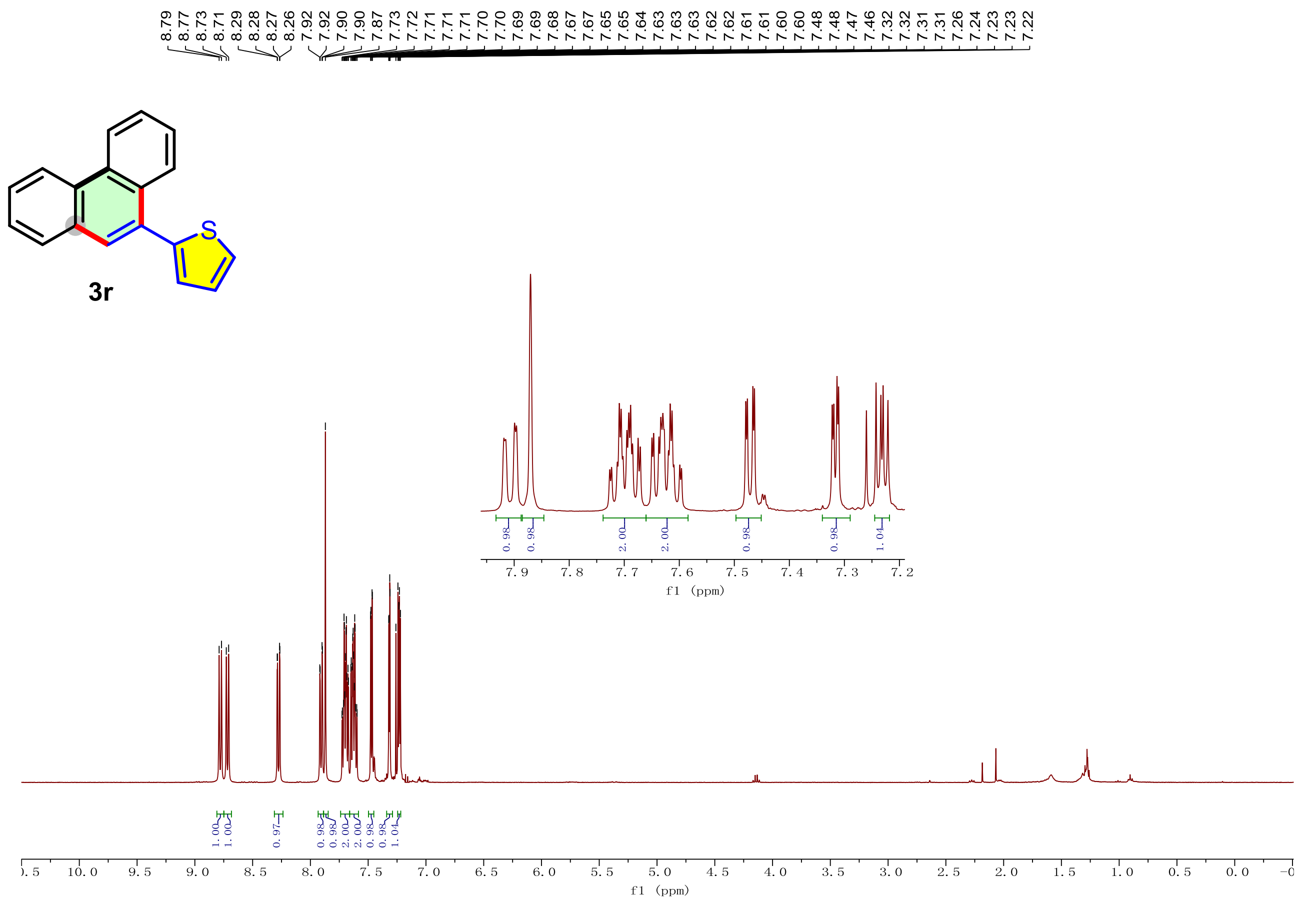

Supplementary Fig. 54. ^{13}C NMR of 3r (101 MHz, $CDCl_3$)

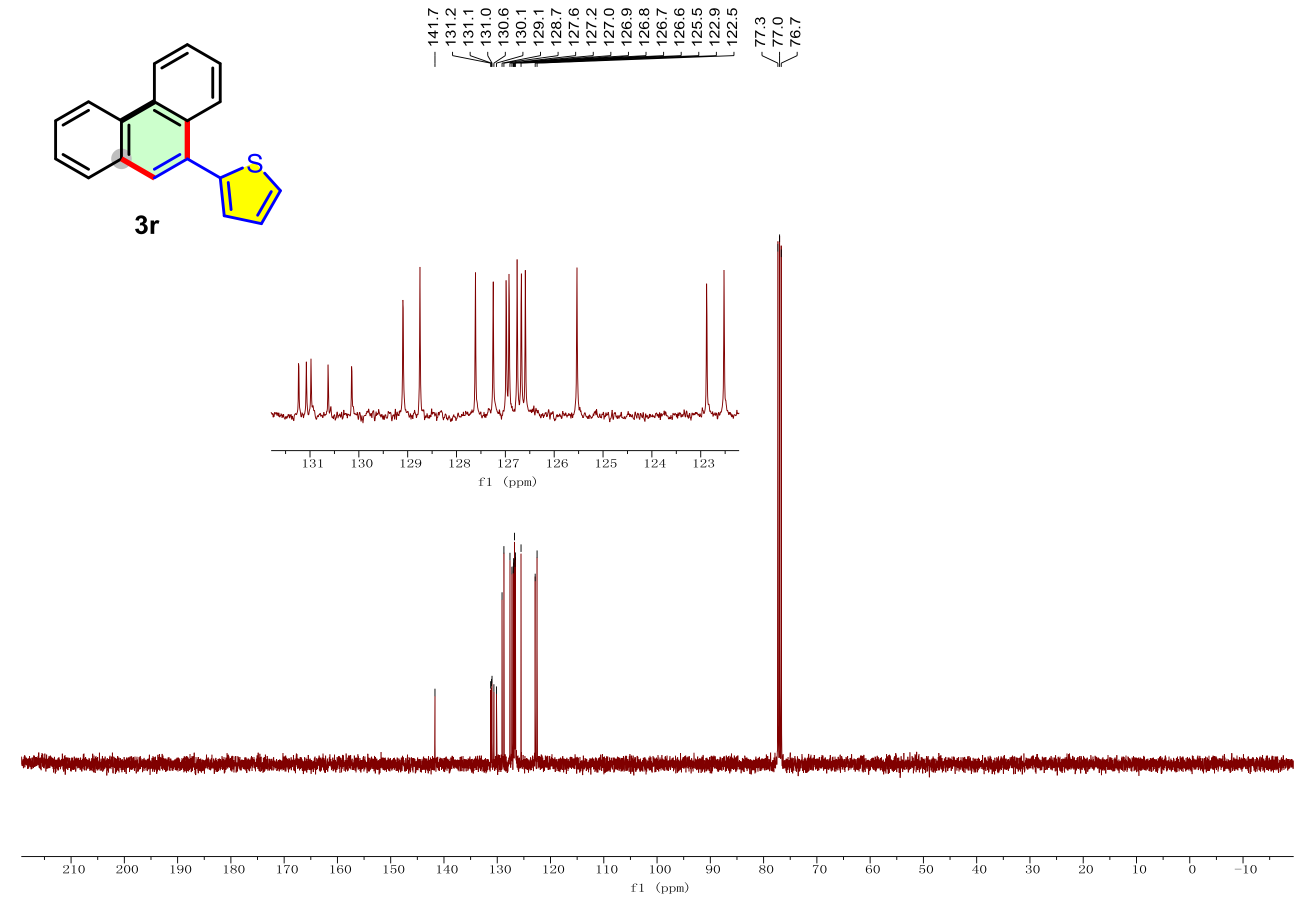

Supplementary Fig. 55. ^{1}H NMR of 3s (400 MHz, $CDCl_3$)

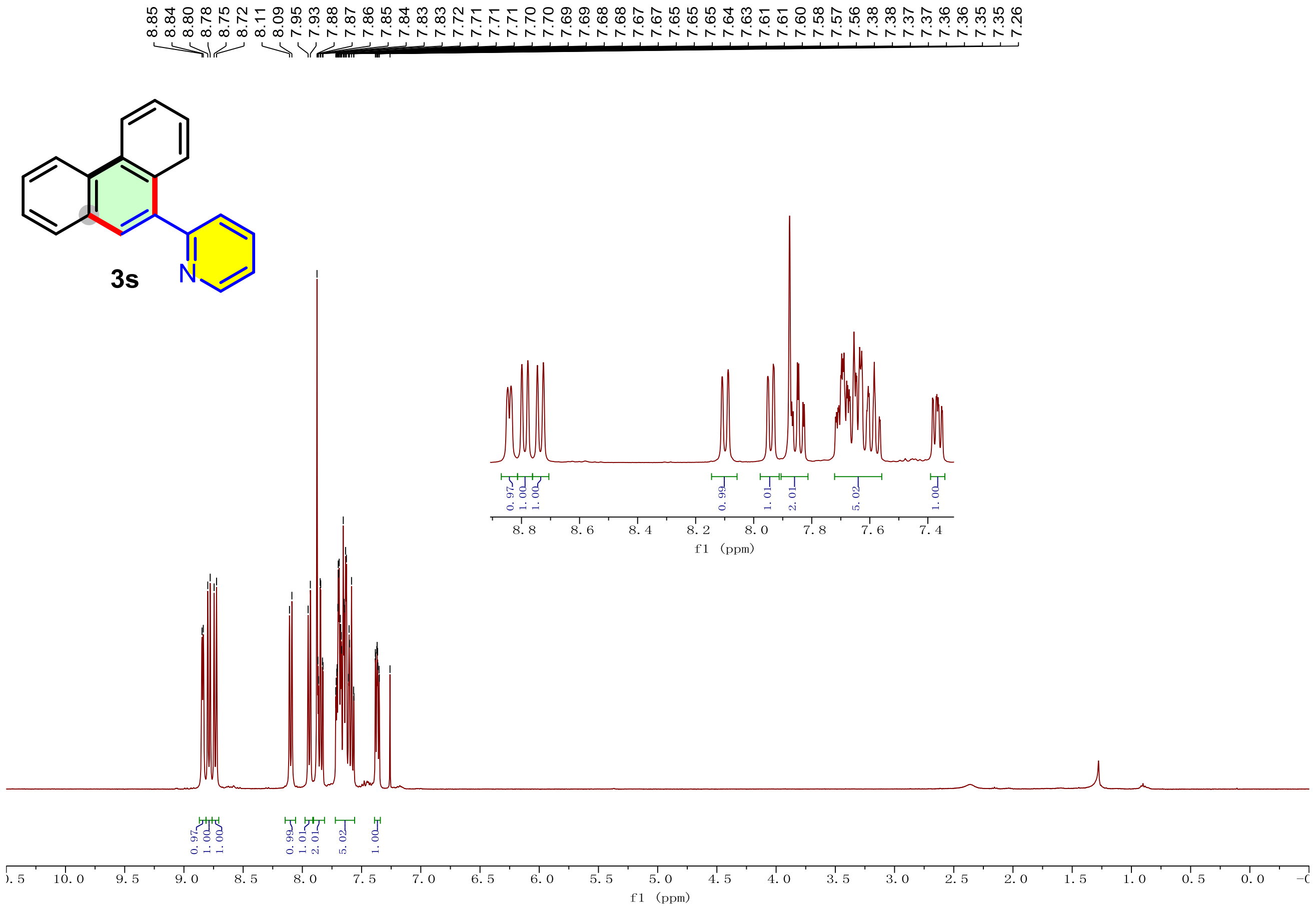

Supplementary Fig. 56. ^{13}C NMR of 3s (101 MHz, $CDCl_3$)

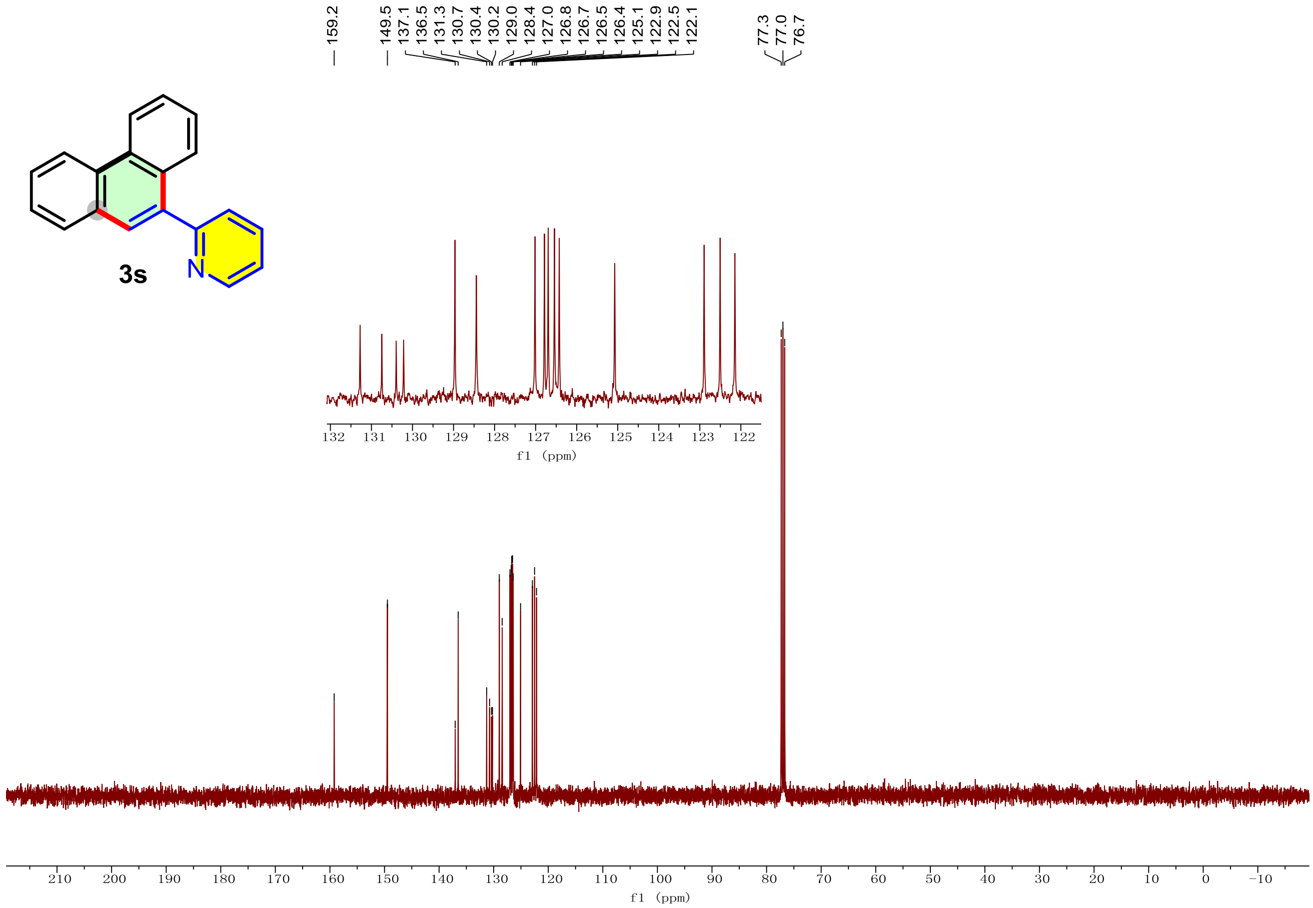

Supplementary Fig. 57. ^{1}H NMR of 3t (400 MHz, $CDCl_3$)

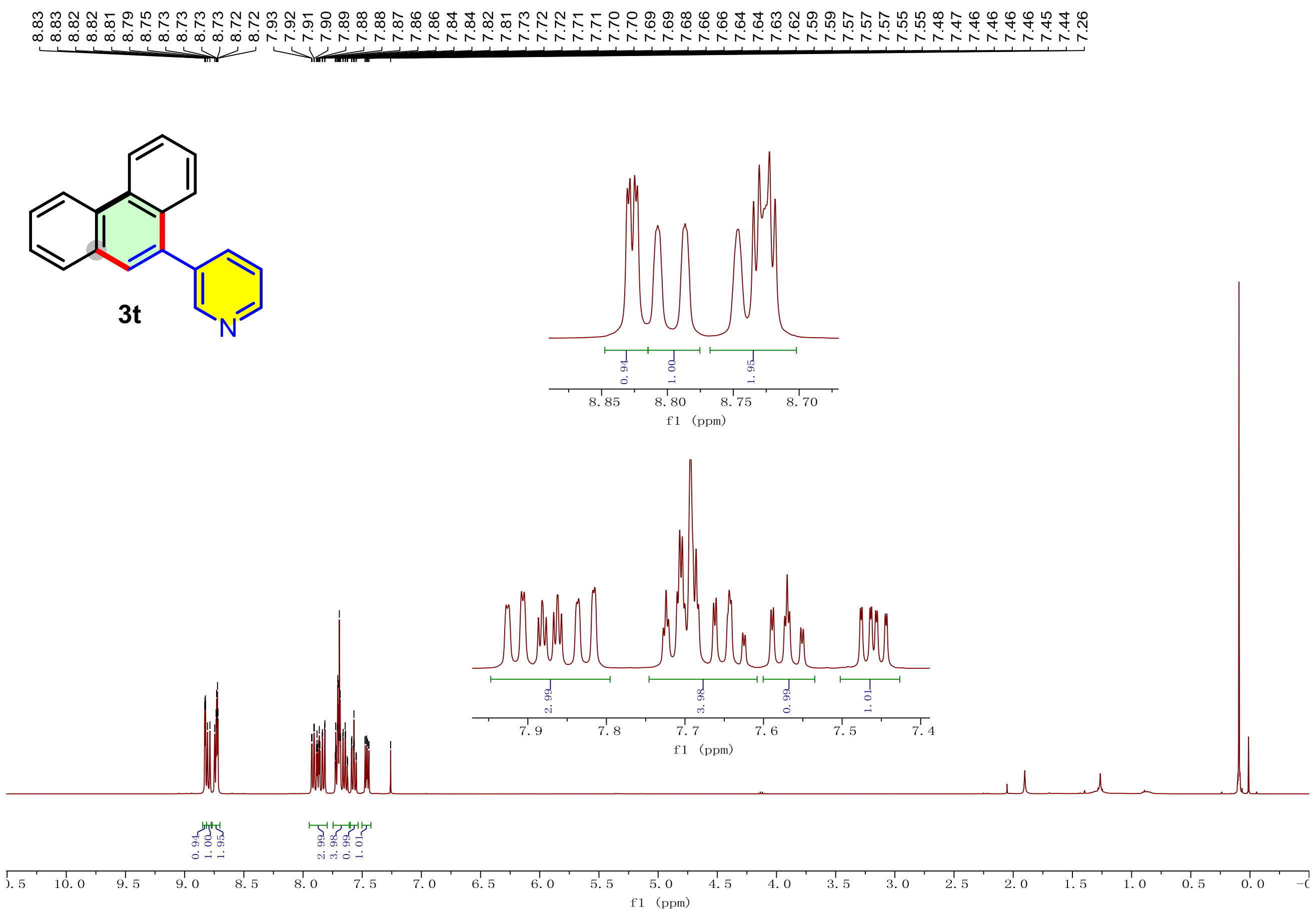

Supplementary Fig. 58. ^{13}C NMR of 3t (101 MHz, $CDCl_3$)

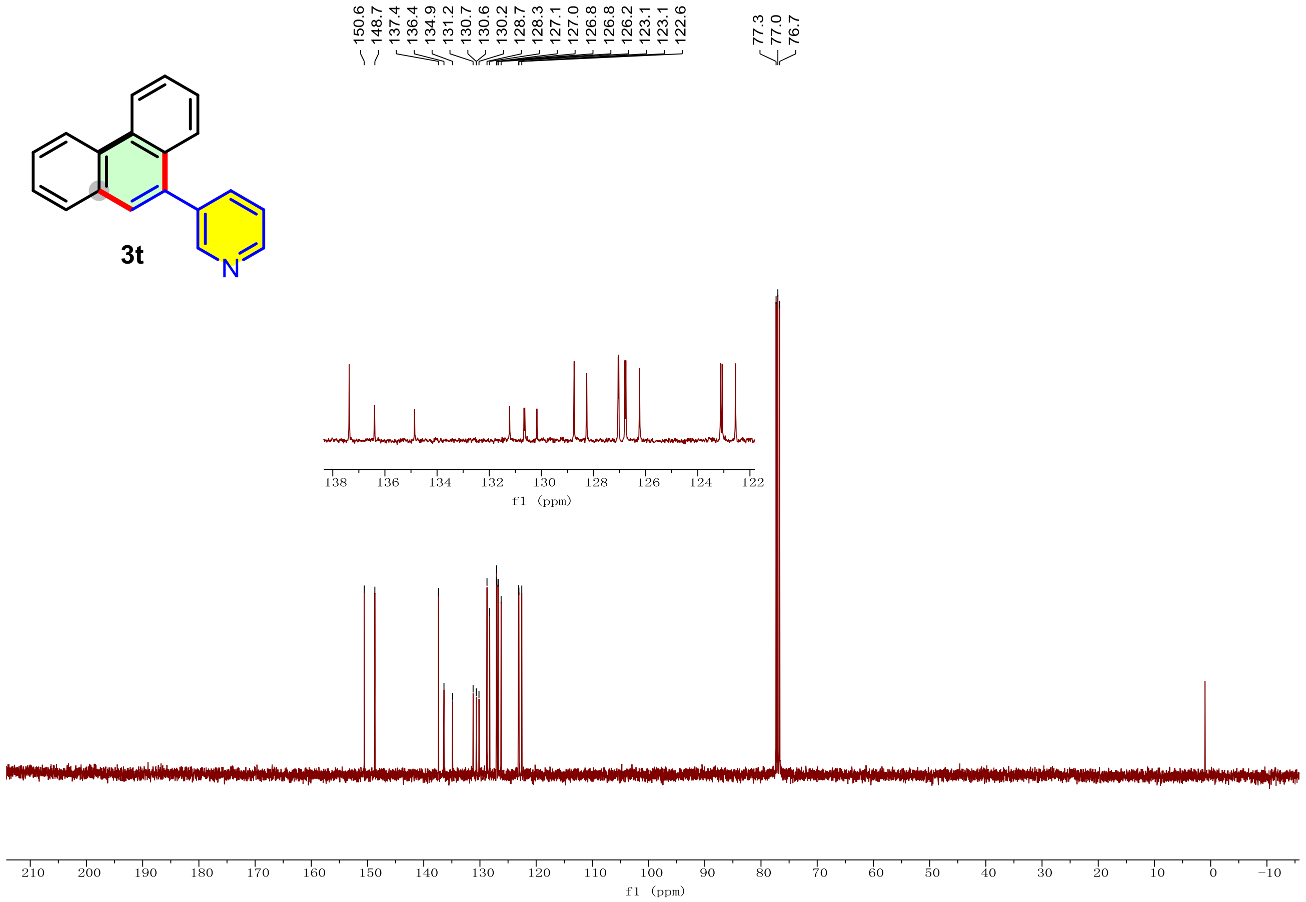

Supplementary Fig. 59. ^{1}H NMR of 3u (600 MHz, $CDCl_3$)

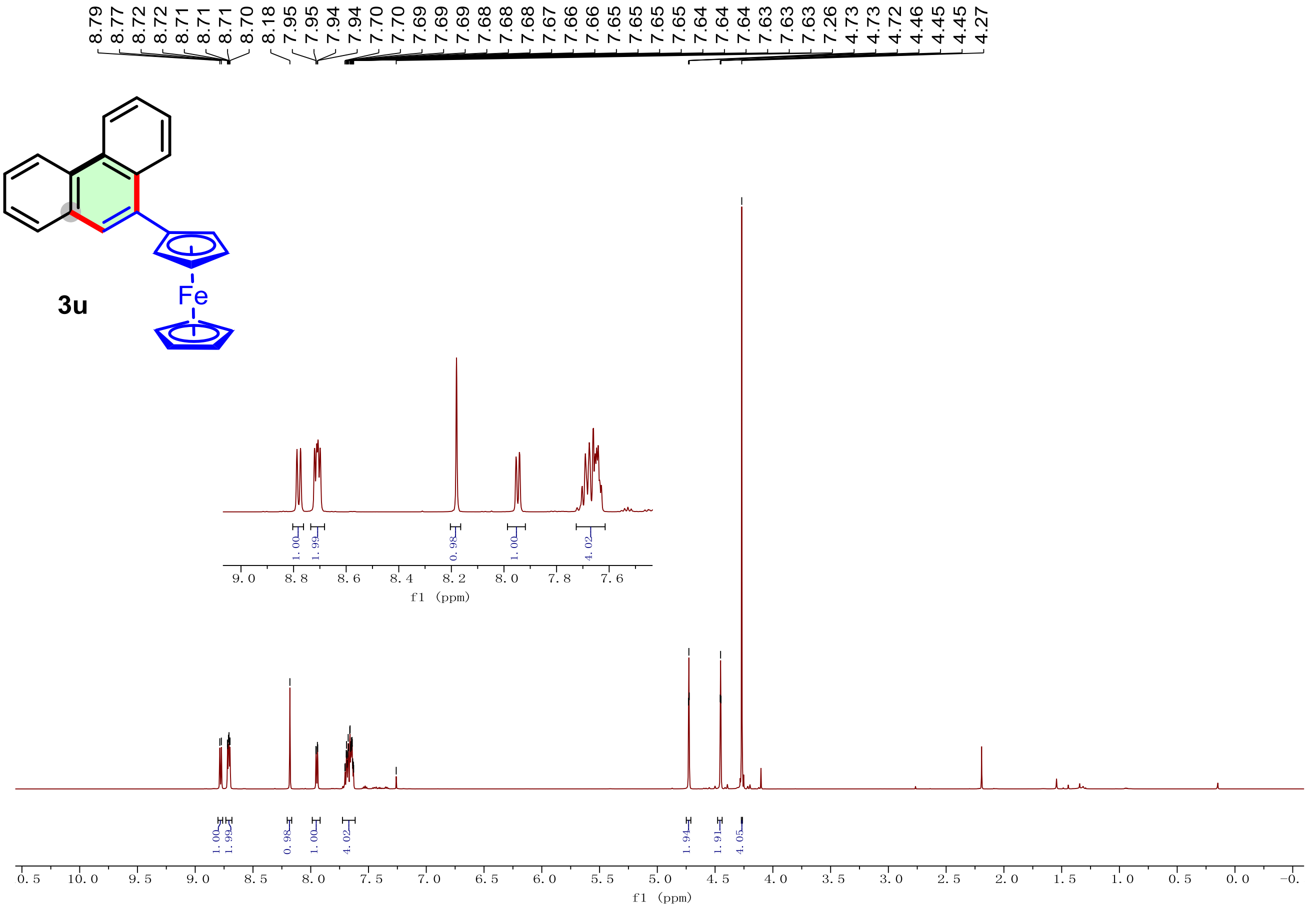

Supplementary Fig. 60. ^{13}C NMR of 3u (151 MHz, $CDCl_3$)

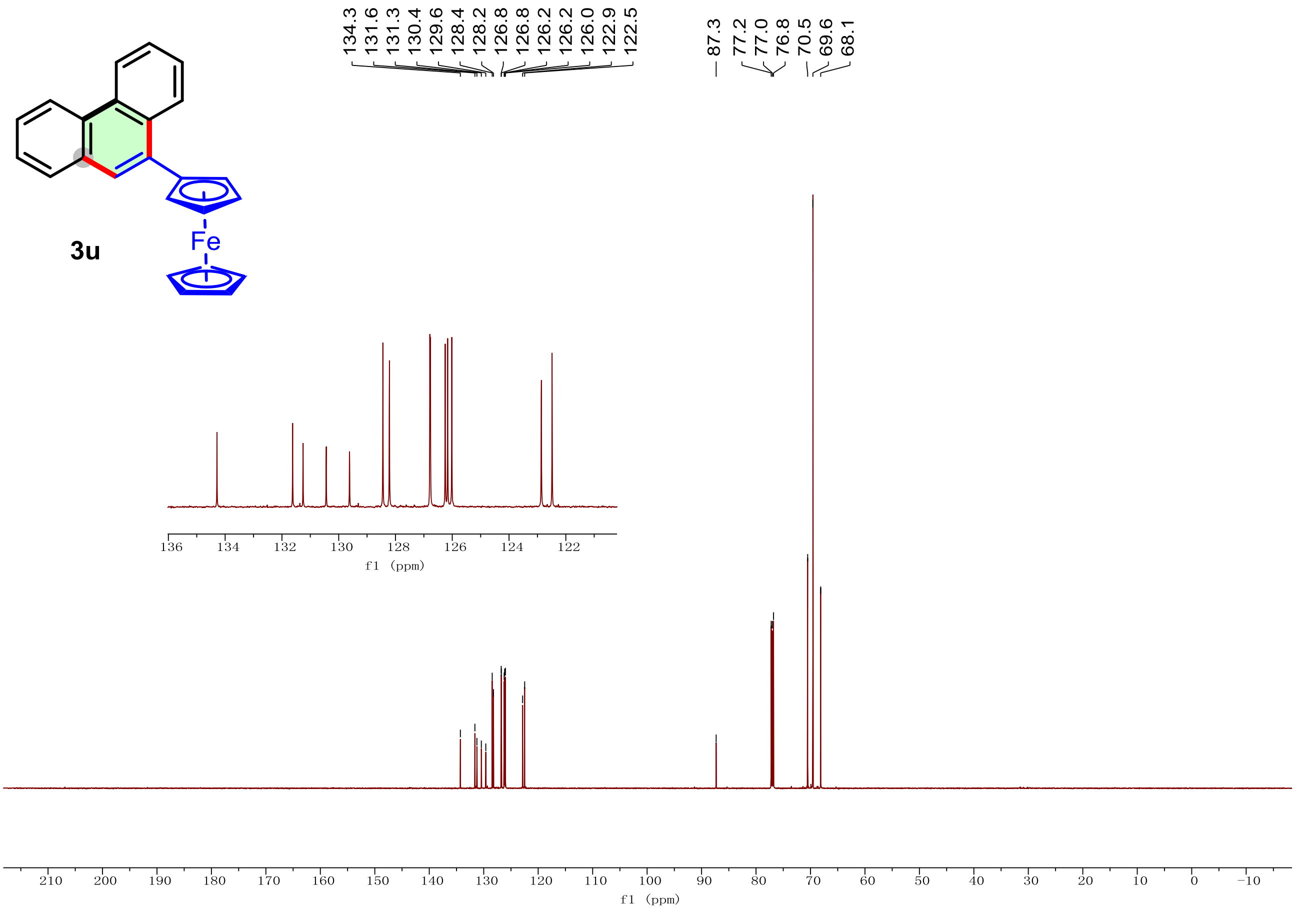

Supplementary Fig. 61. ^{1}H NMR of 3v (600 MHz, $CDCl_3$)

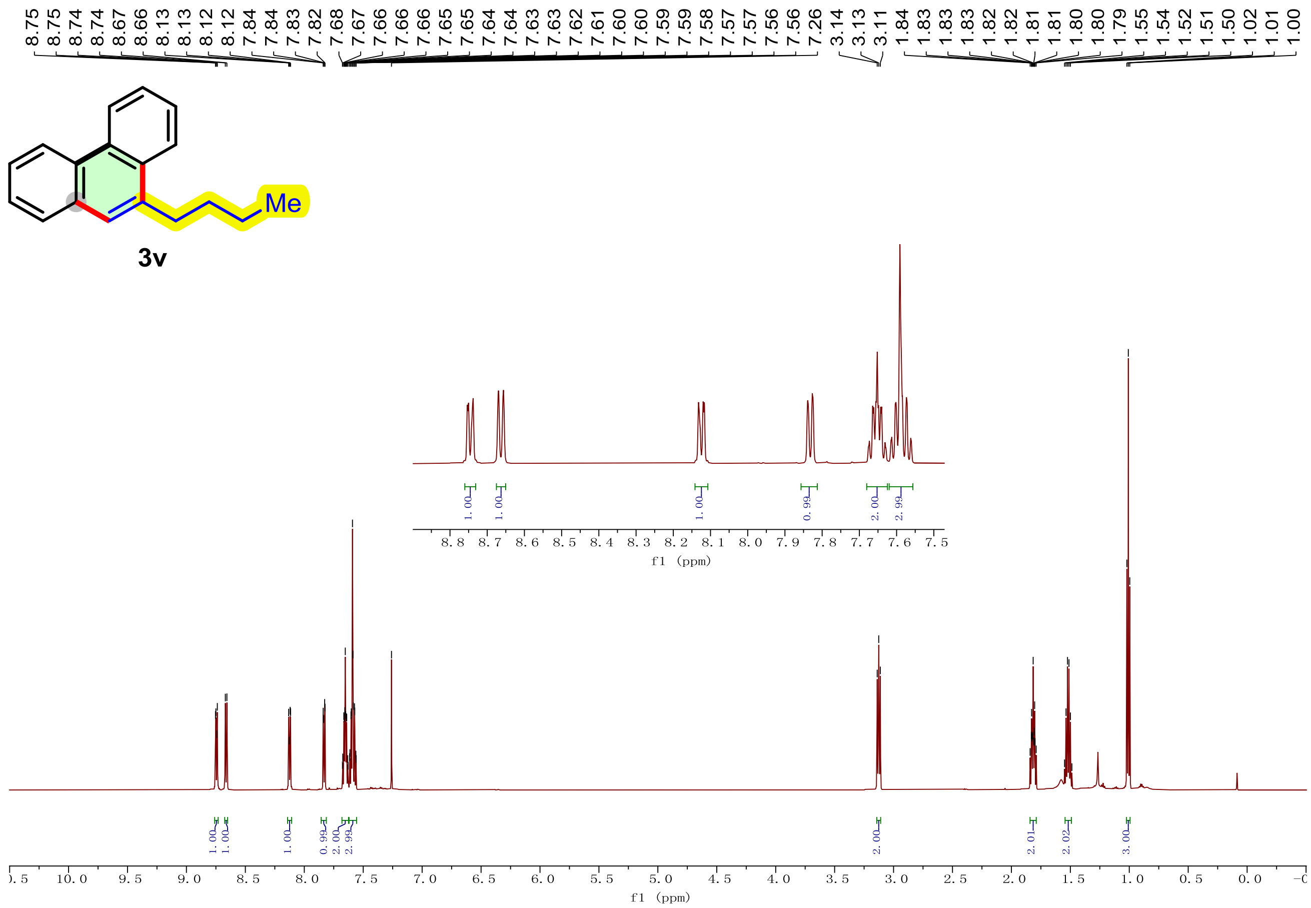

Supplementary Fig. 62. ^{13}C NMR of 3u (151 MHz, $CDCl_3$)

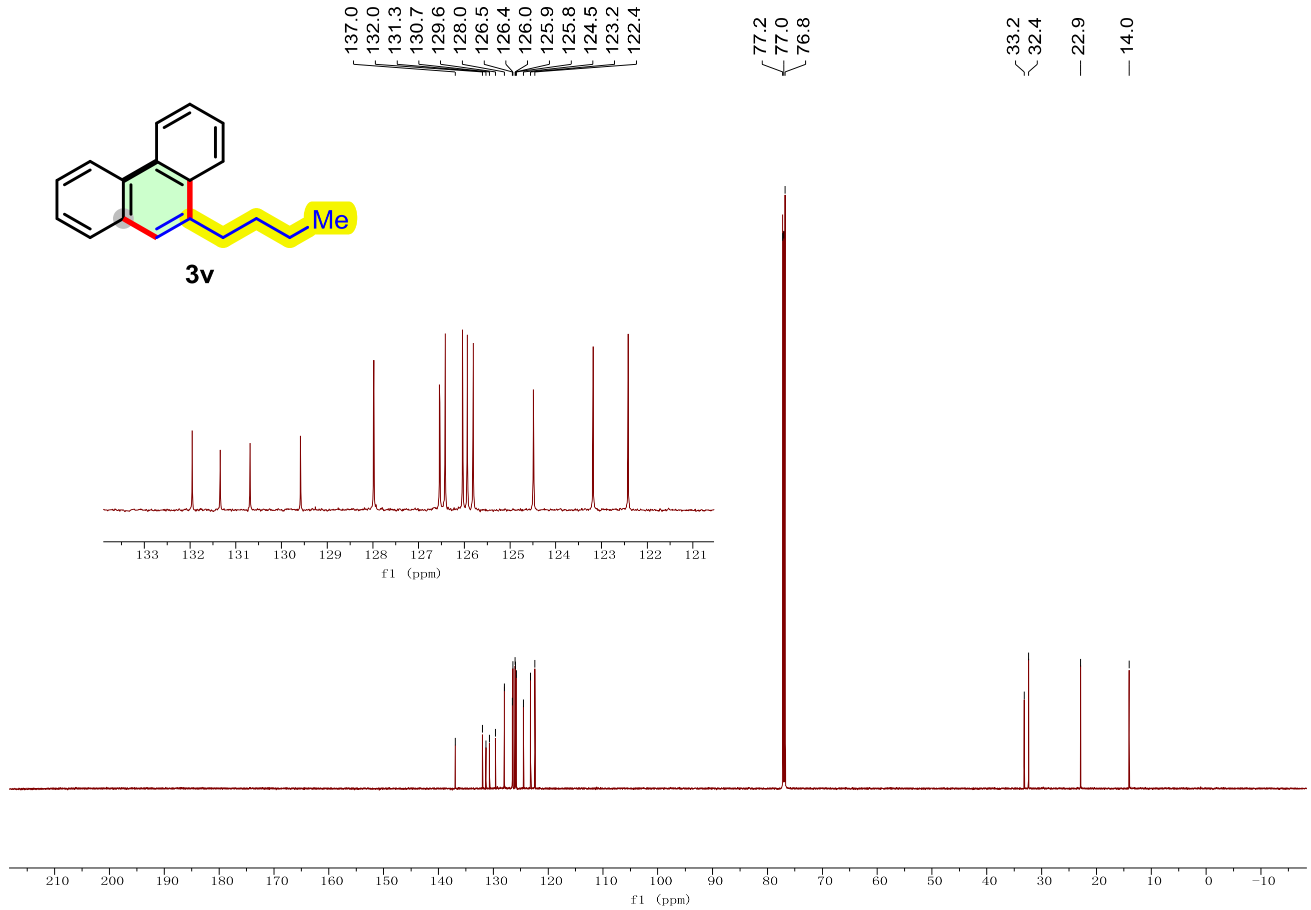

Supplementary Fig. 63. ^{1}H NMR of 3w (400 MHz, $CDCl_3$)

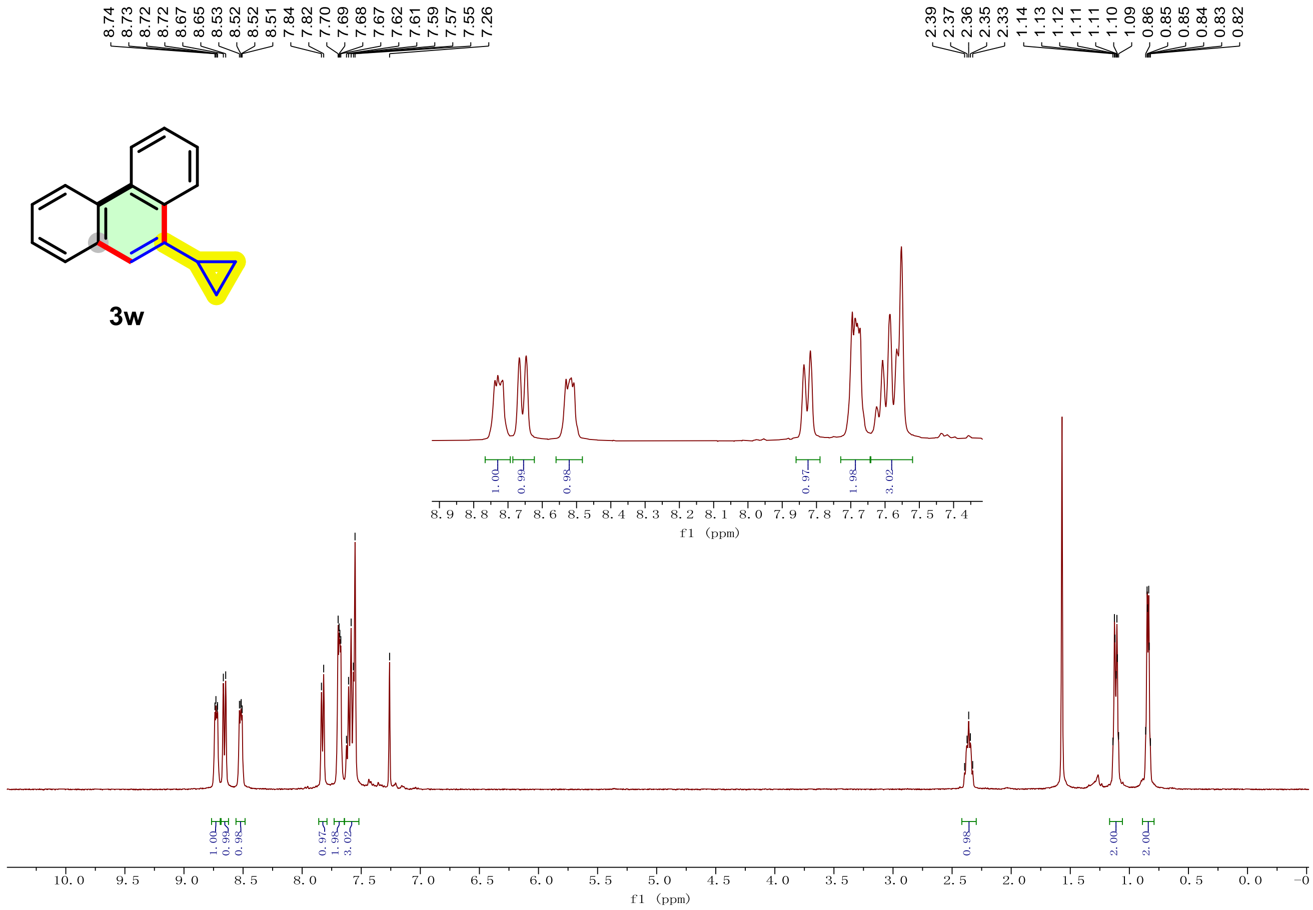

Supplementary Fig. 64. ^{13}C NMR of 3w (101 MHz, $CDCl_3$)

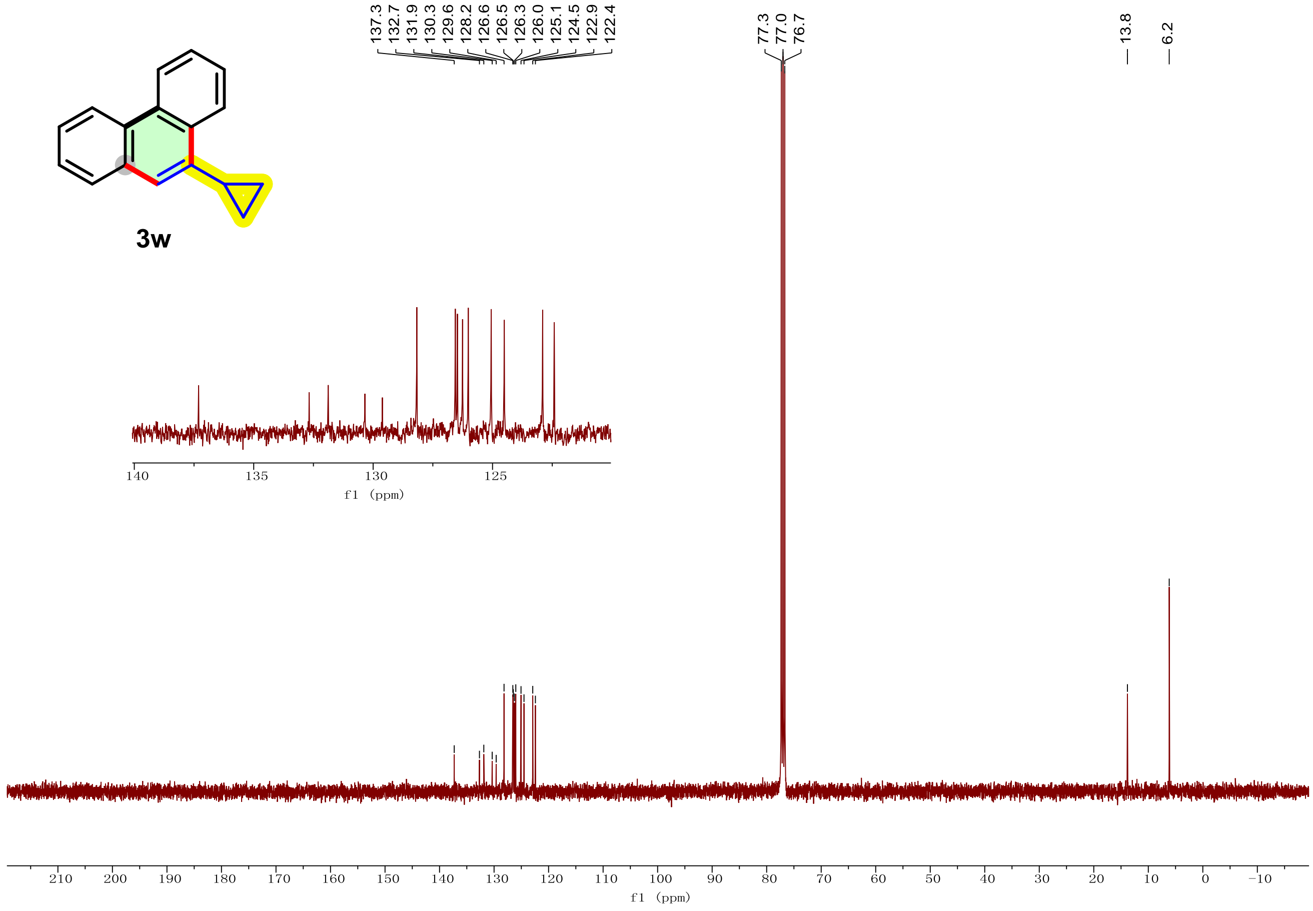

Supplementary Fig. 65. ^{1}H NMR of 3x (400 MHz, $CDCl_3$)

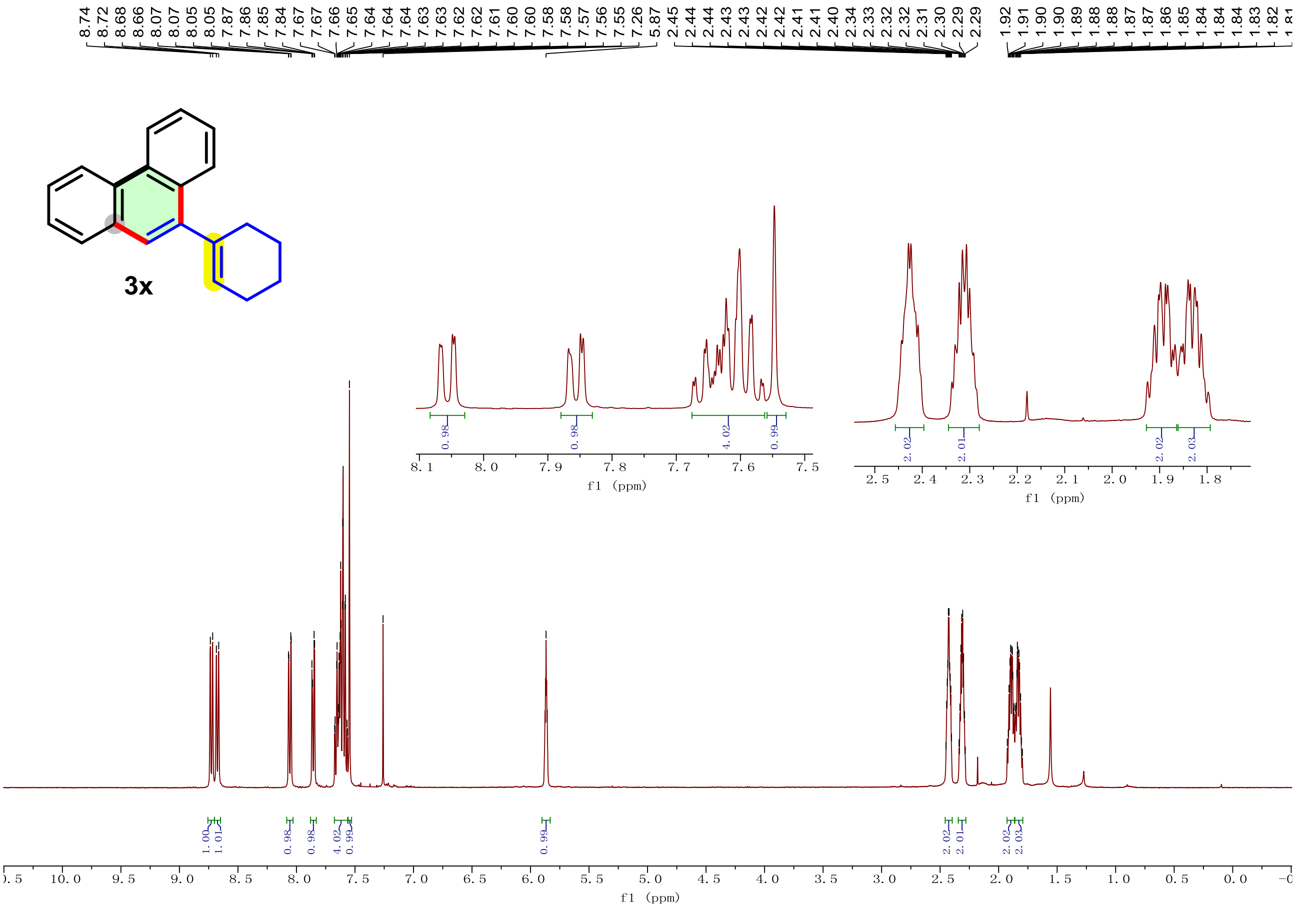

Supplementary Fig. 66. ^{13}C NMR of 3x (101 MHz, $CDCl_3$)

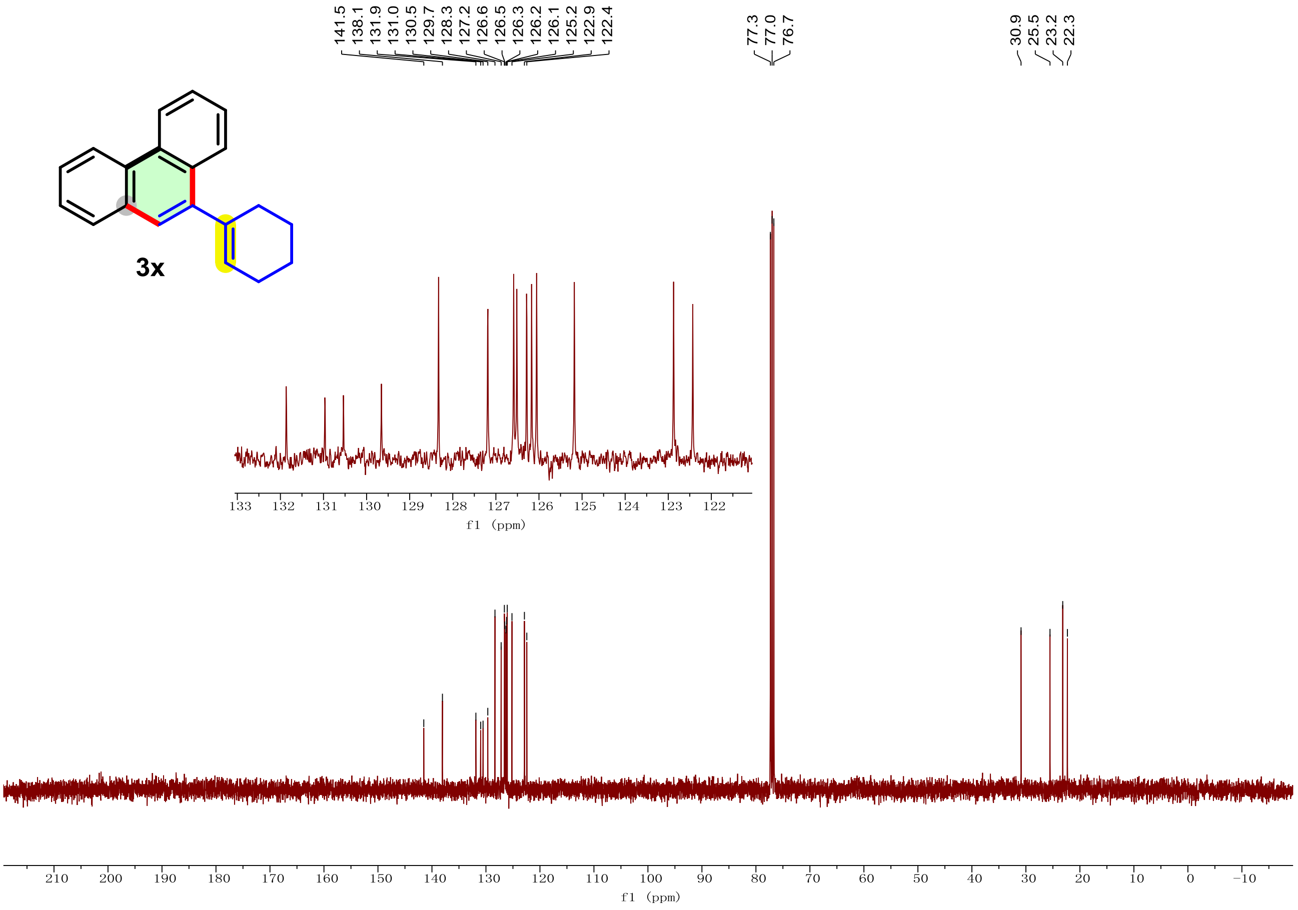

Supplementary Fig. 67. ¹H NMRof 3y (600 MHz, CDCl₃)

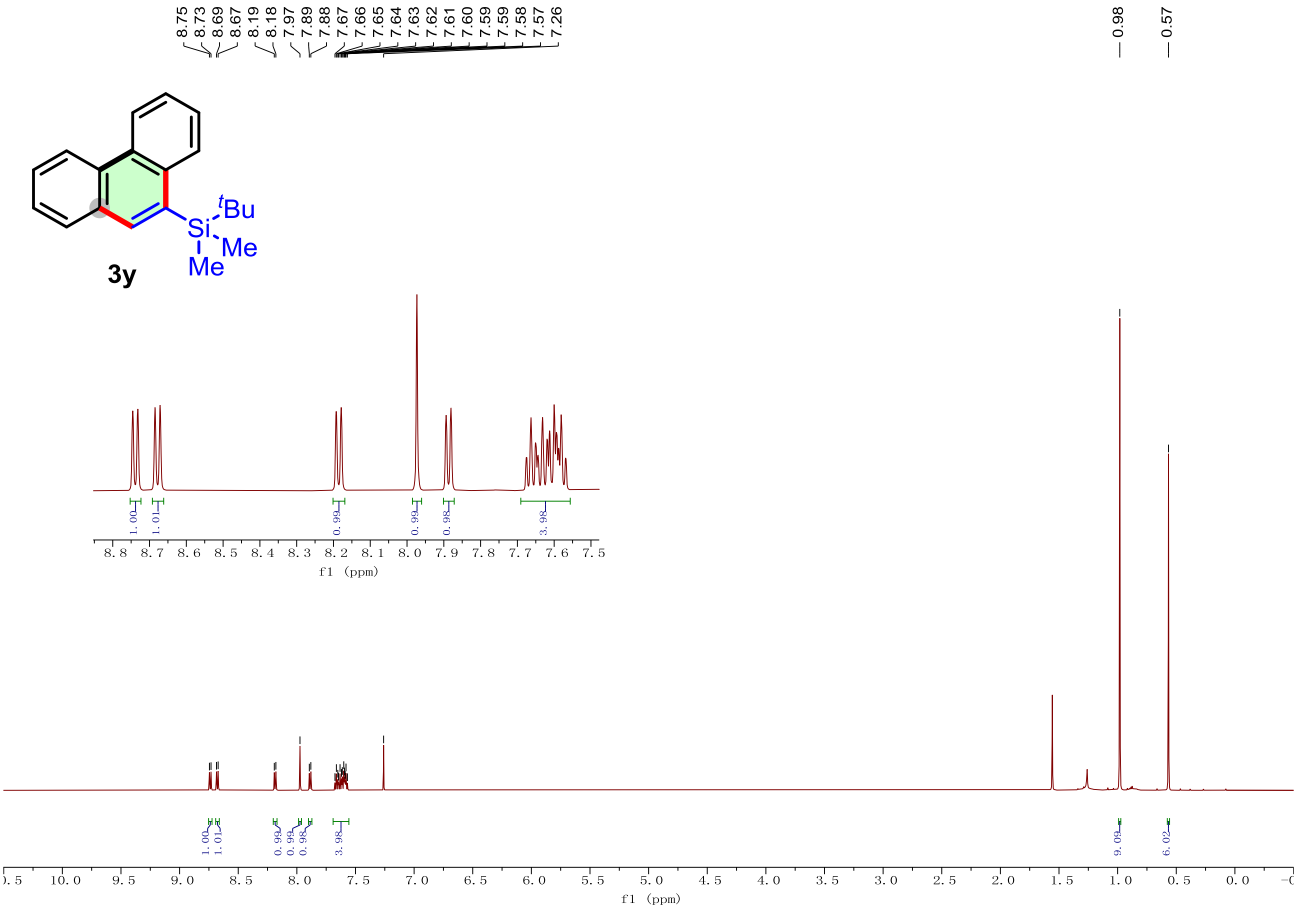

Supplementary Fig. 68. ¹³C NMR of 3y (151 MHz, CDCl₃)

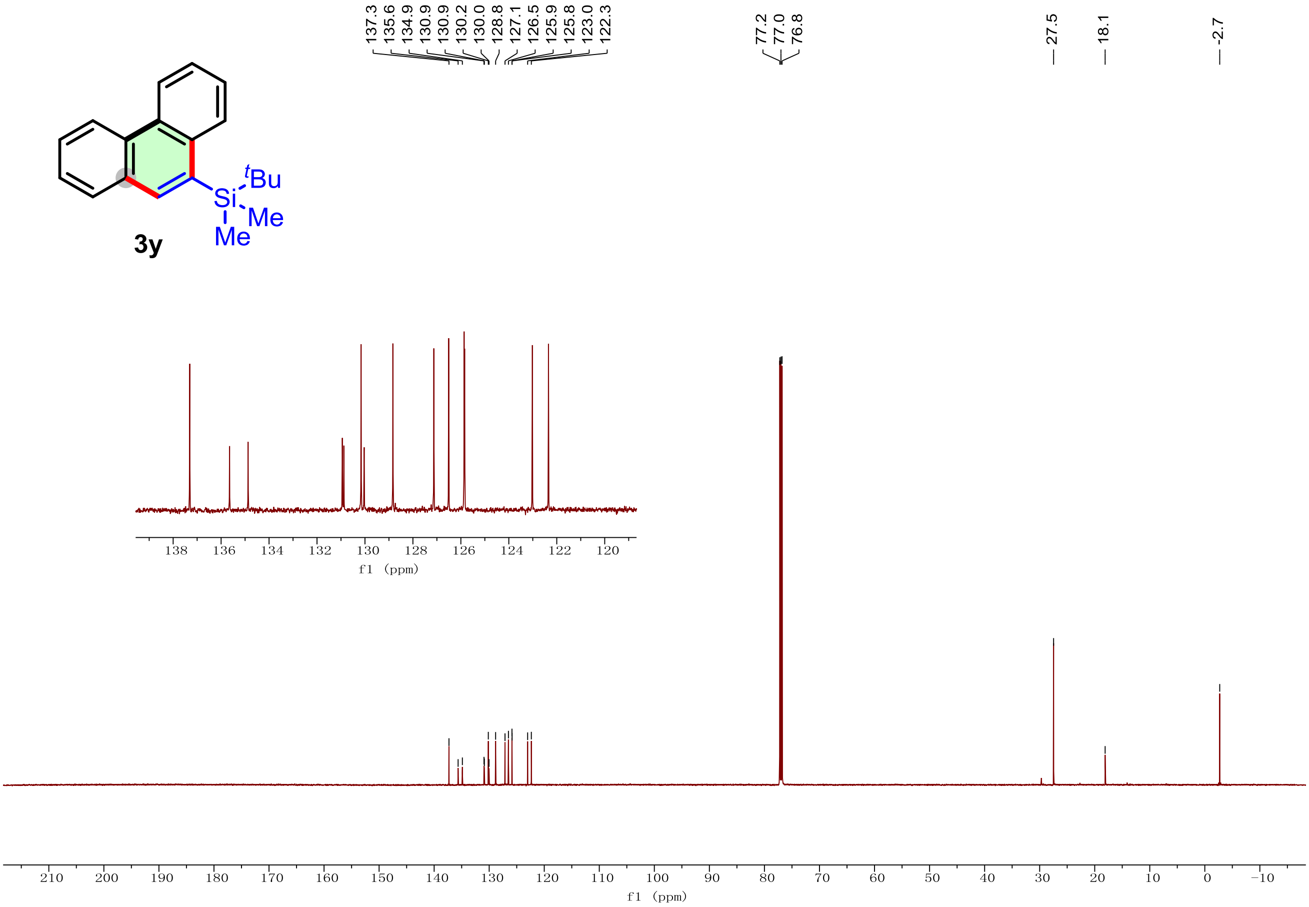

Supplementary Fig. 69. ^{1}H NMR of 3z (600 MHz, $CDCl_3$)

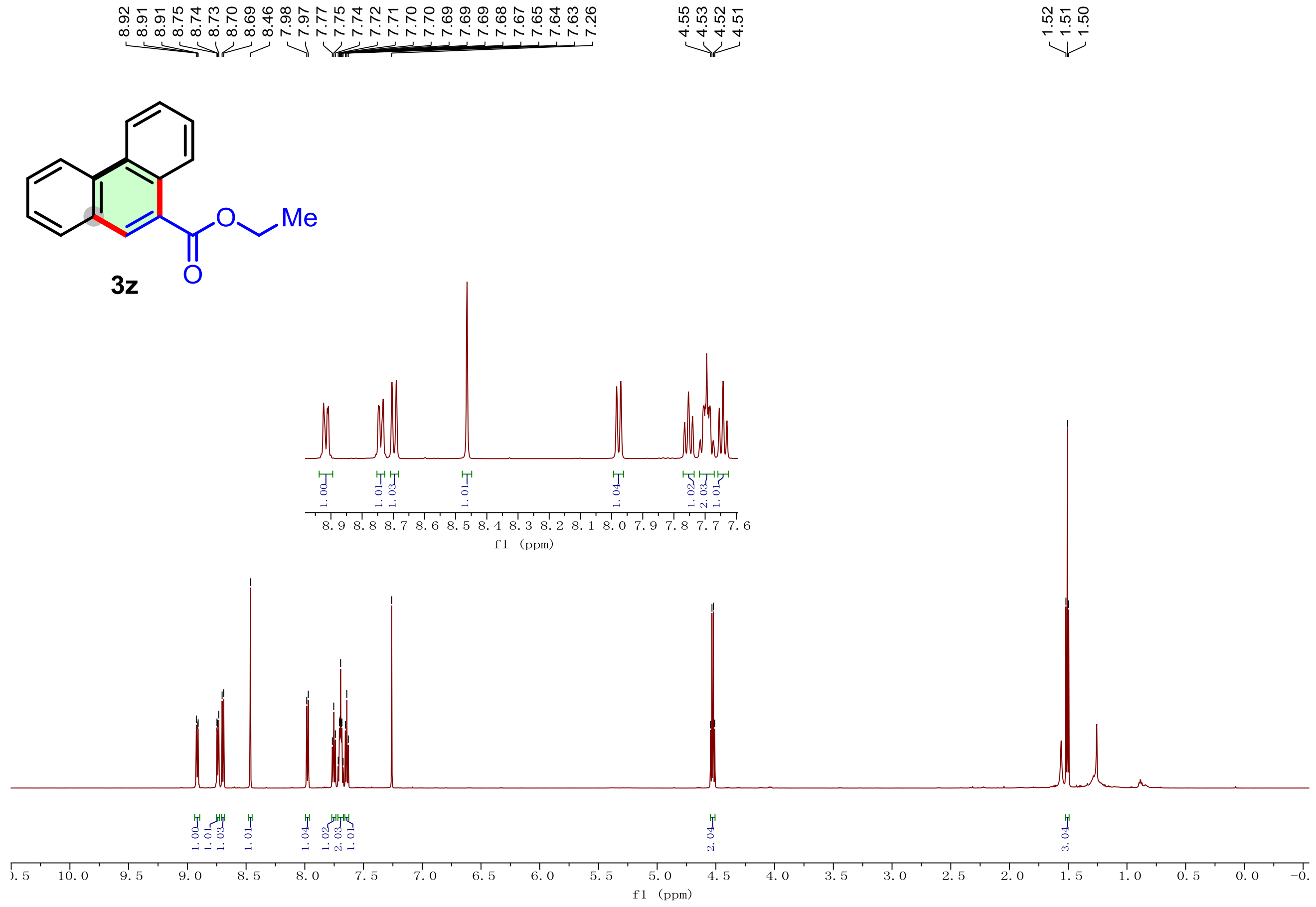

Supplementary Fig. 70. ^{13}C NMR of 3z (151 MHz, $CDCl_3$)

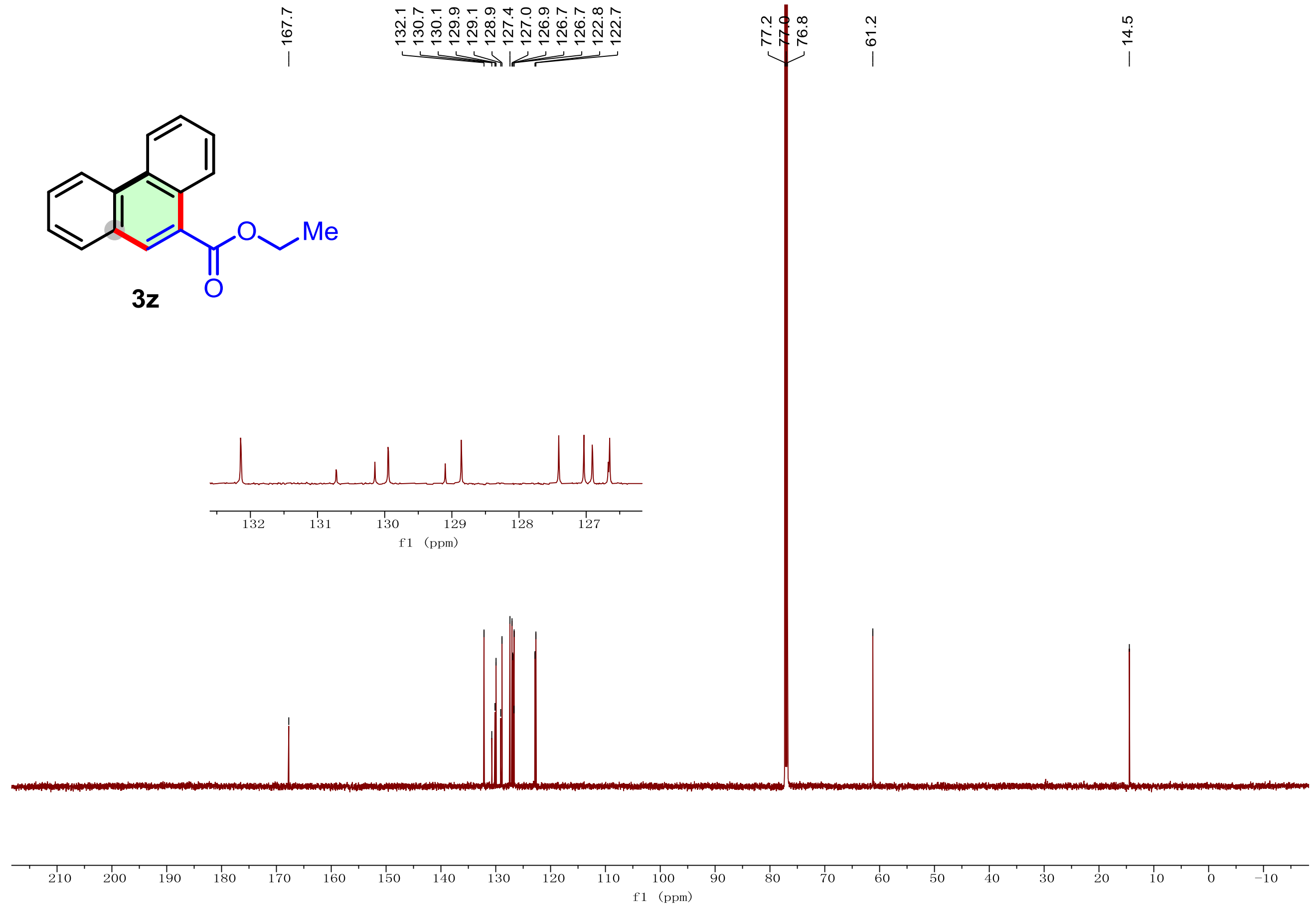

Supplementary Fig. 71. ^{1}H NMR of 3aa (400 MHz, $CDCl_3$)

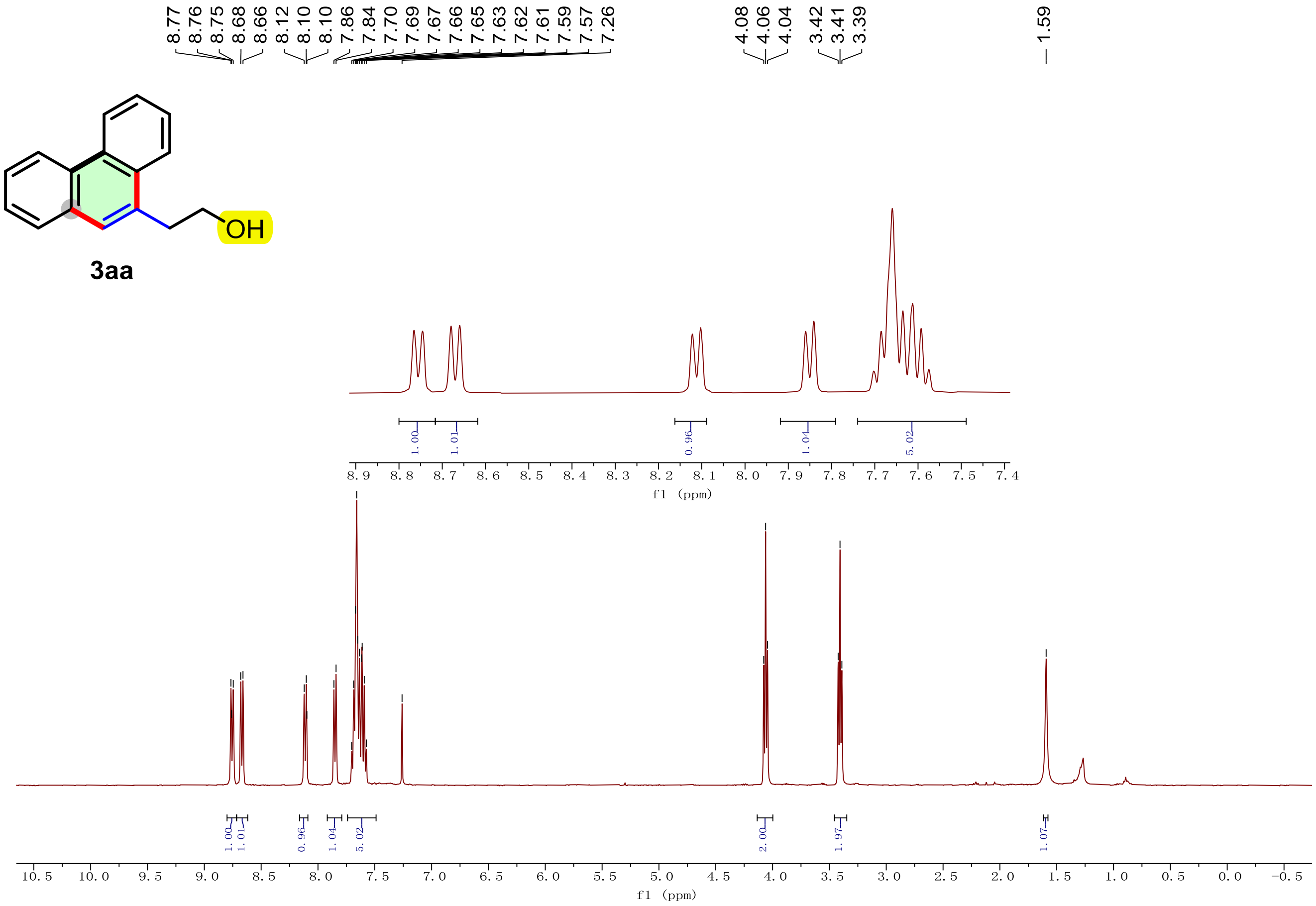

Supplementary Fig. 72. ^{13}C NMR of 3aa (151 MHz, $CDCl_3$)

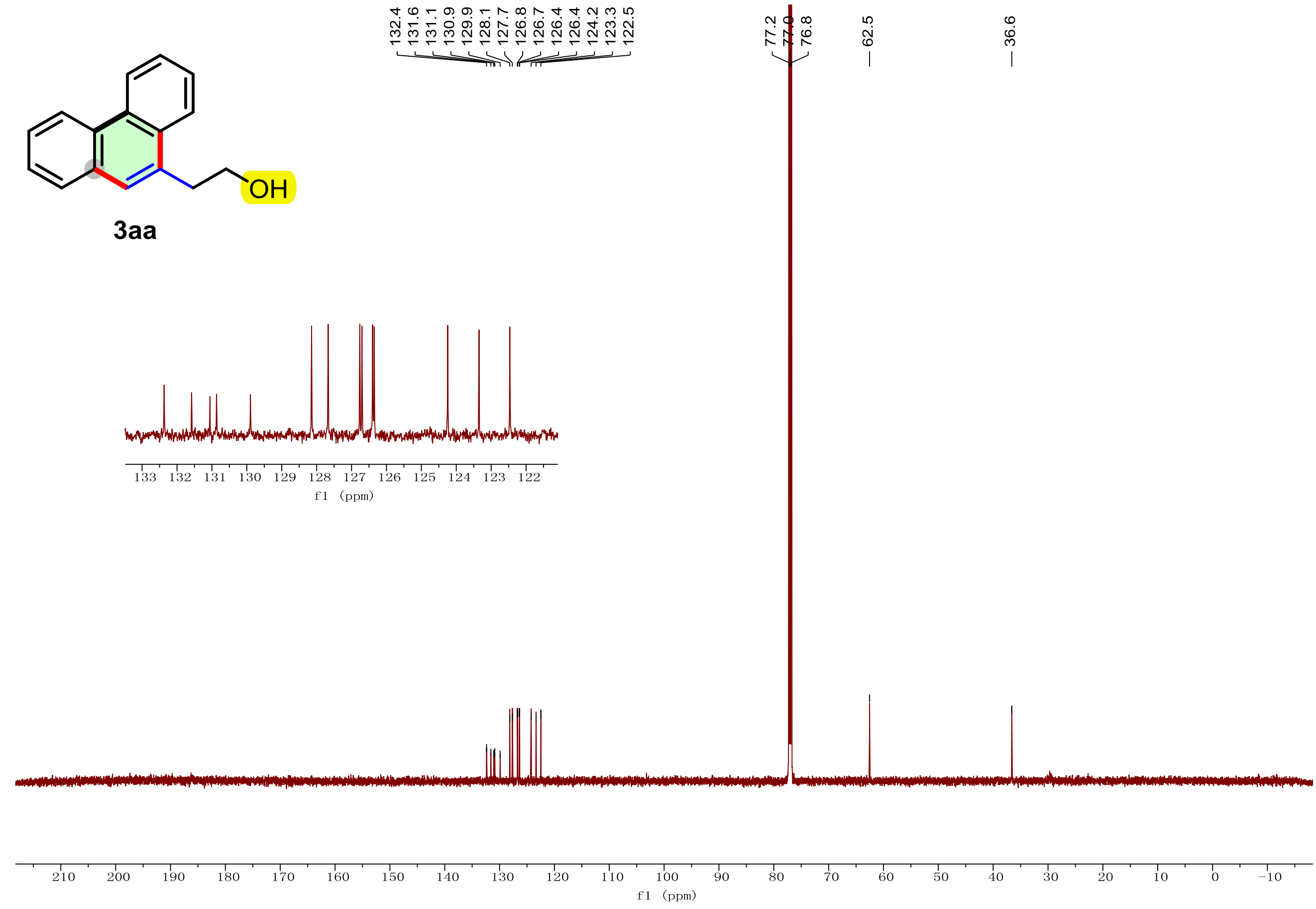

Supplementary Fig. 73. ^{1}H NMR of 3ab (600 MHz, $CDCl_3$)

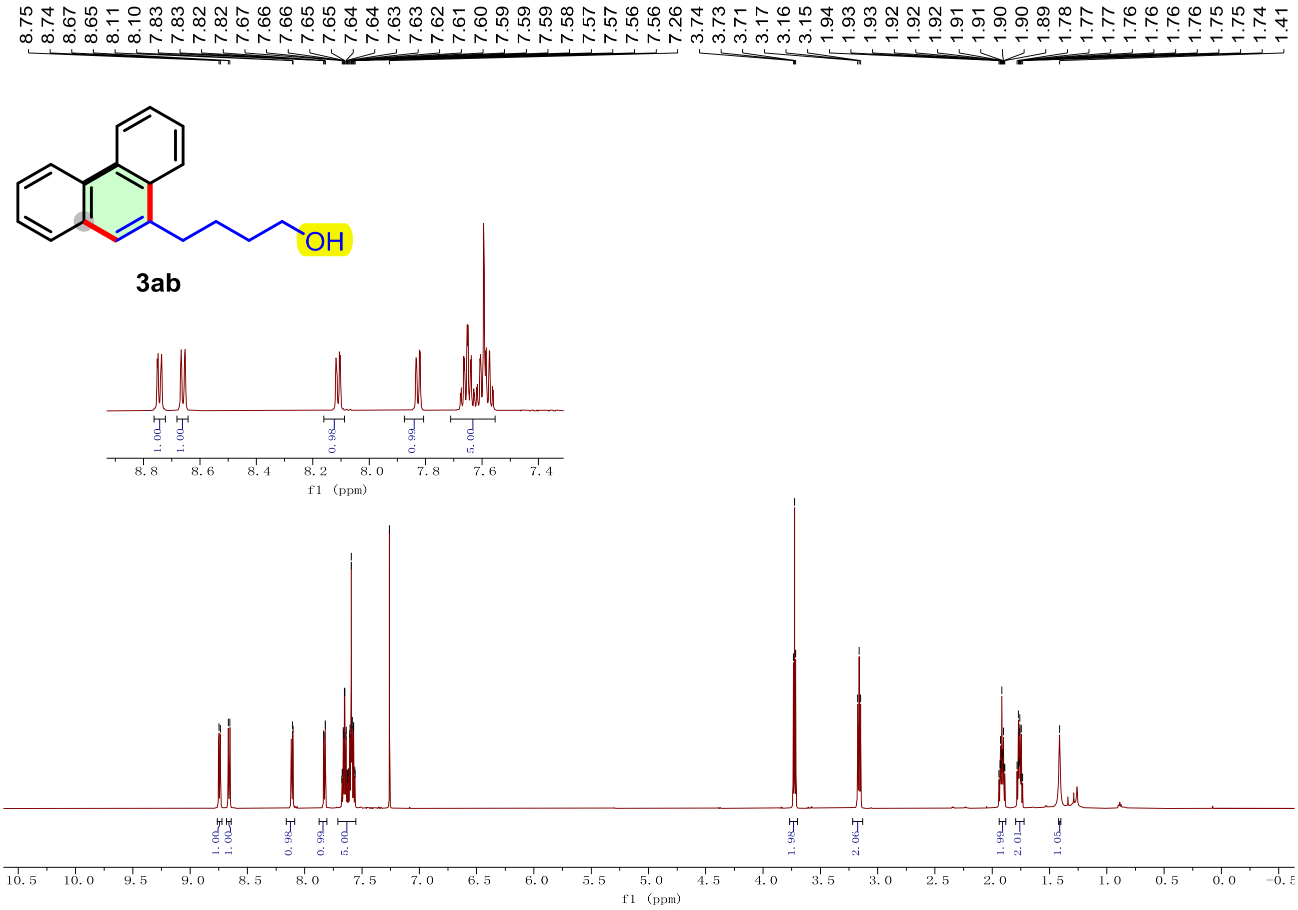

Supplementary Fig. 74. ^{13}C NMR of 3ab (151 MHz, $CDCl_3$)

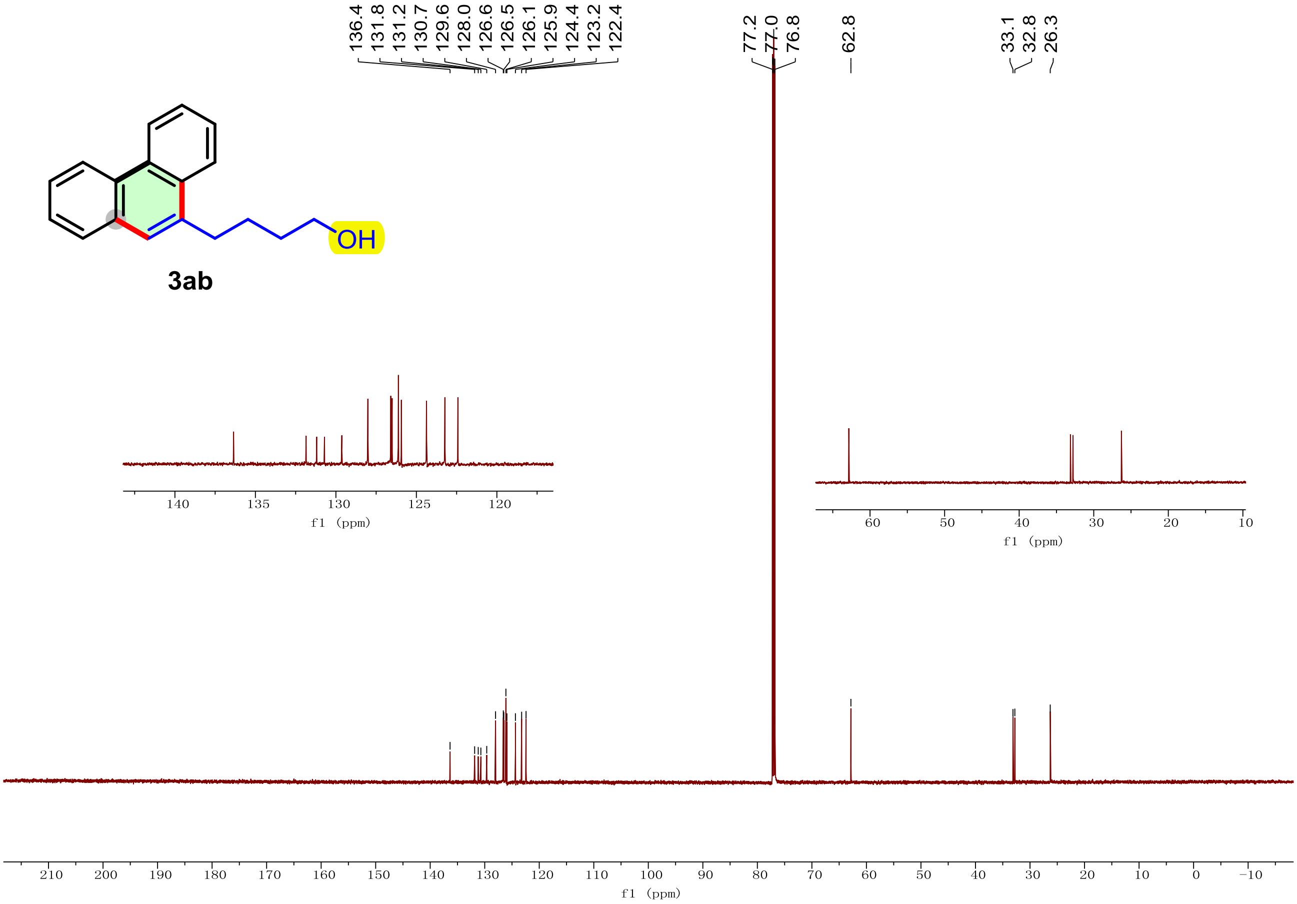

Supplementary Fig. 75. ^{1}H NMR of 3ac (600 MHz, $CDCl_3$)

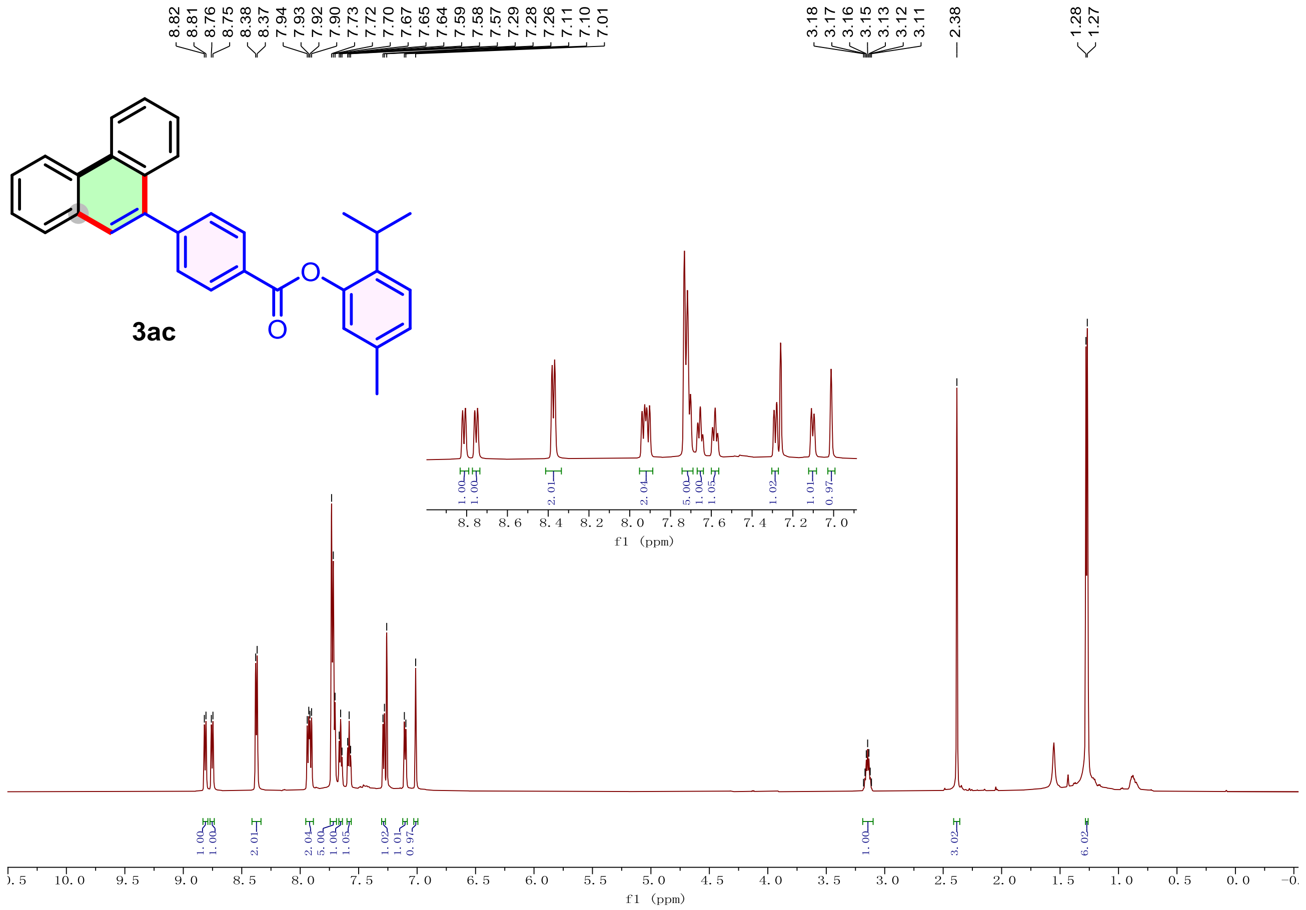

Supplementary Fig. 76. ^{13}C NMR of 3ac (151 MHz, $CDCl_3$)

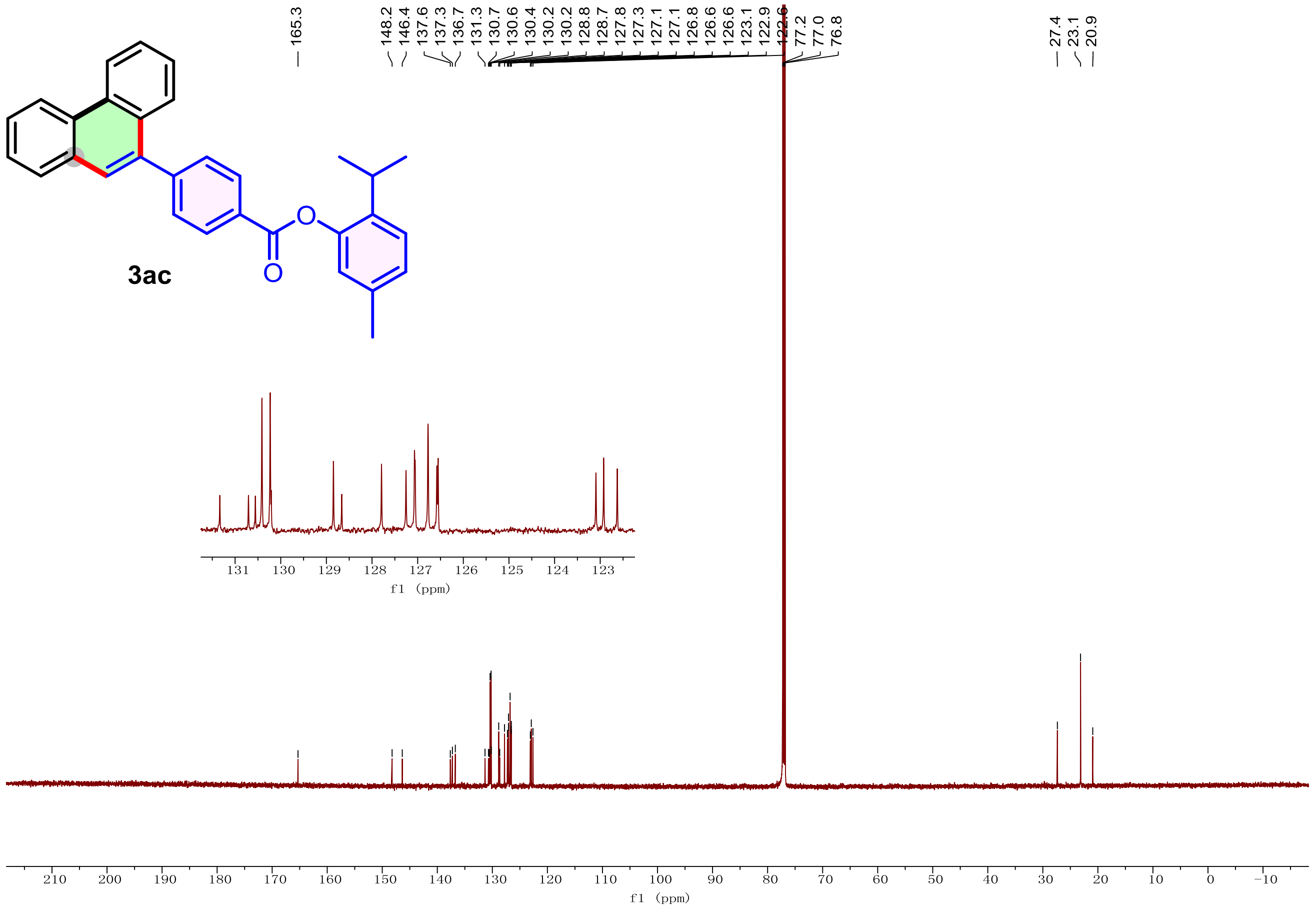

Supplementary Fig. 77. ^{1}H NMR of 3ad (600 MHz, $CDCl_3$)

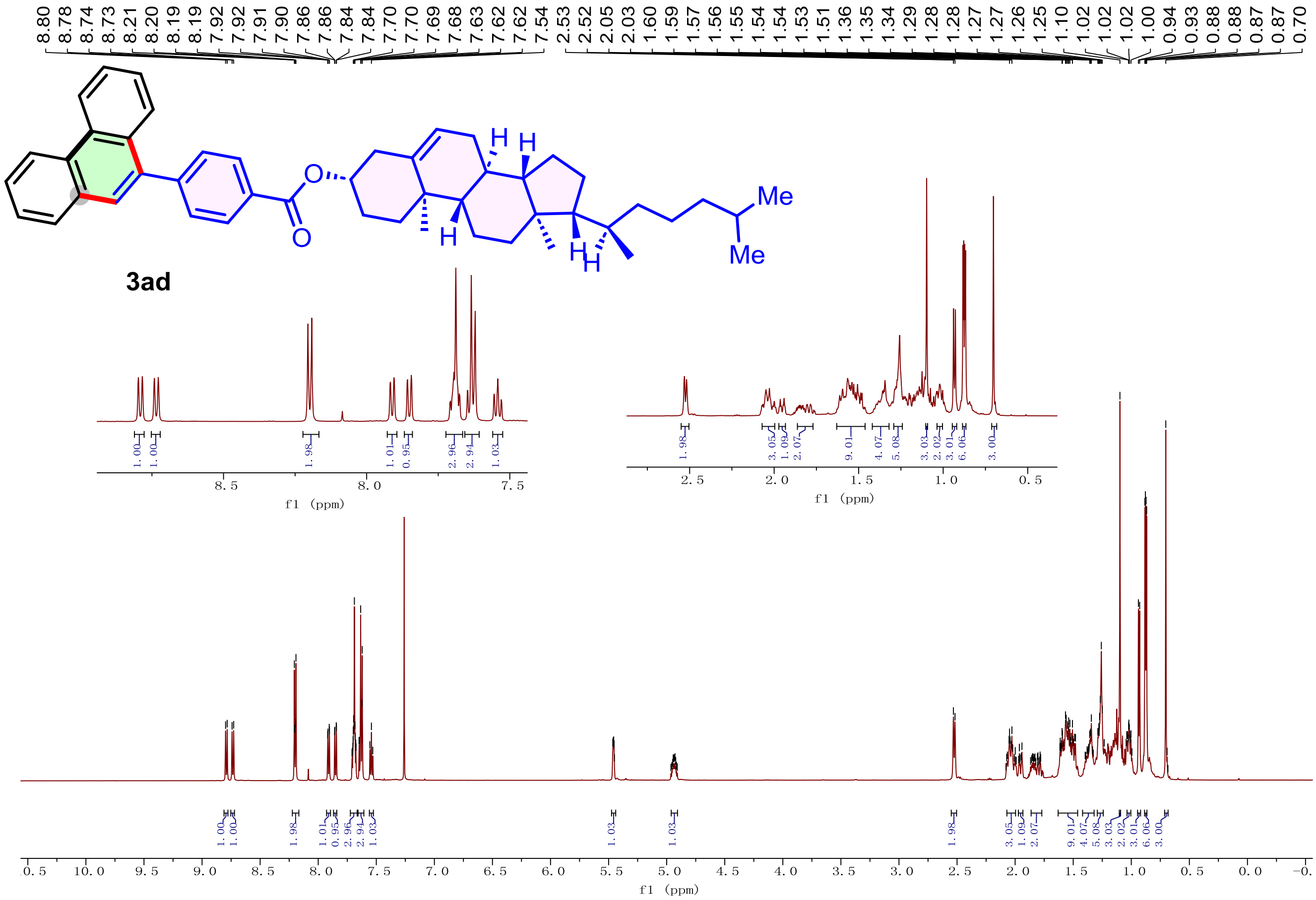

Supplementary Fig. 78. ^{13}C NMR of 3ad (151 MHz, $CDCl_3$)

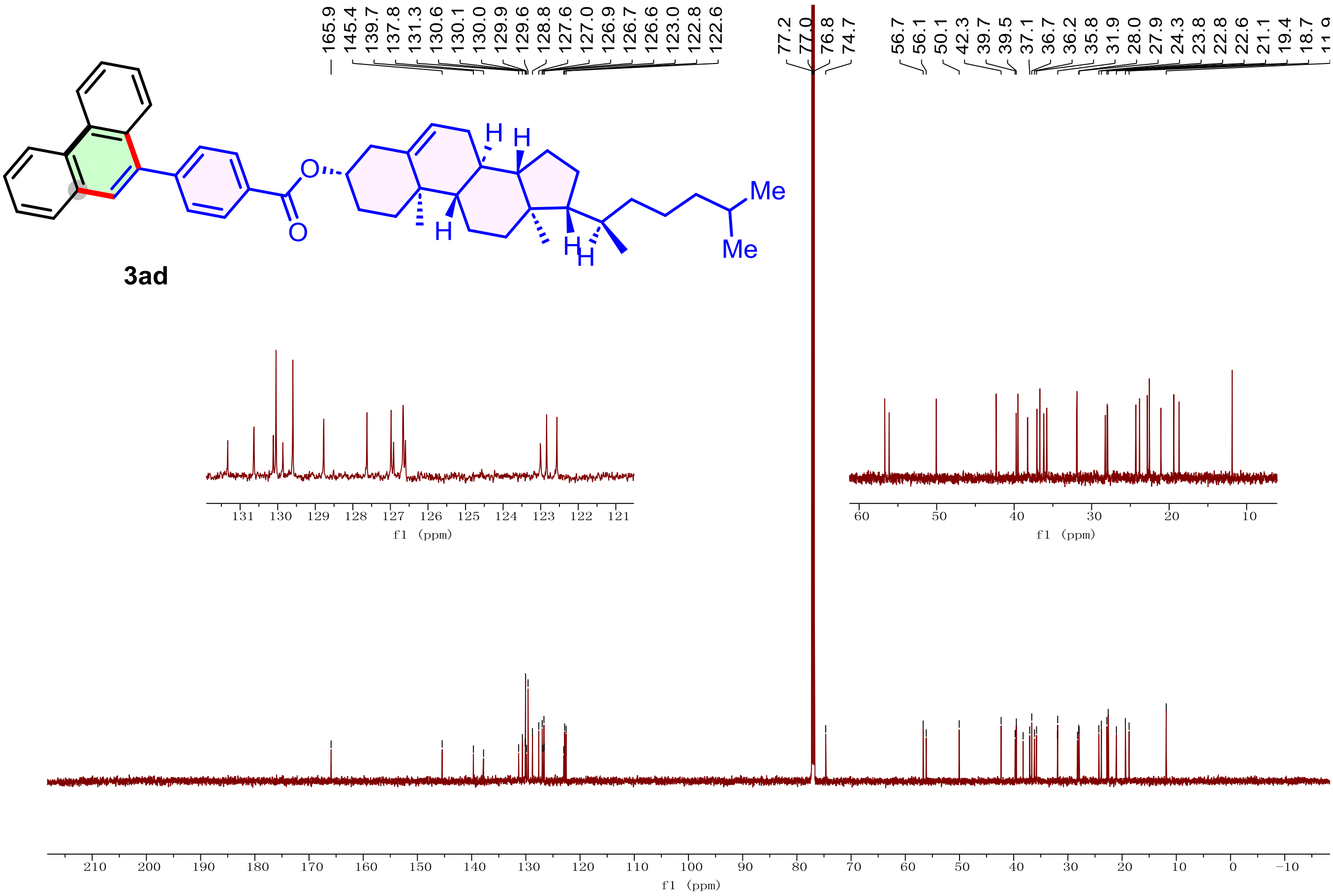

Supplementary Fig. 79. ^{1}H NMR of 3ae (400 MHz, $CDCl_3$)

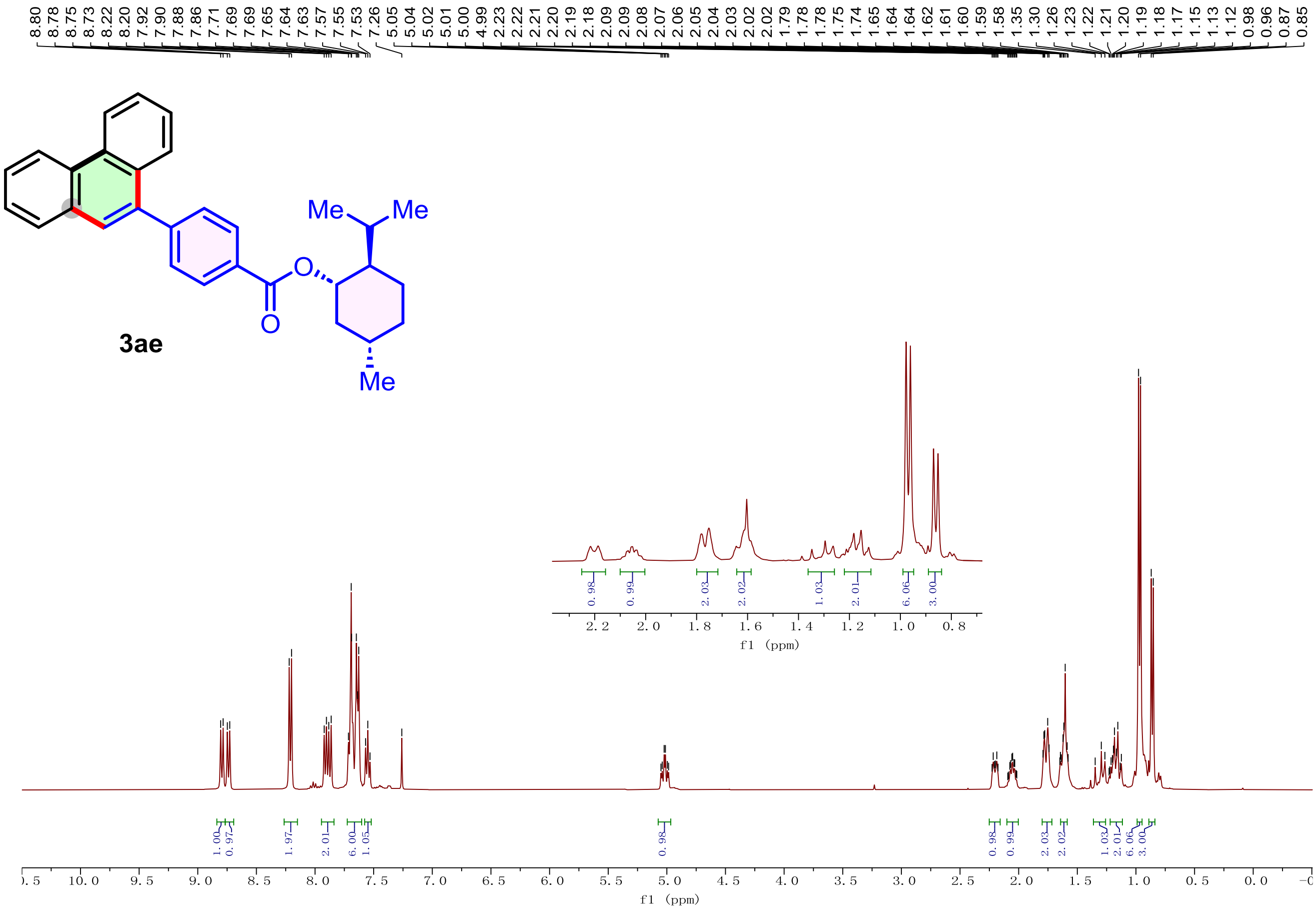

Supplementary Fig. 80. ^{13}C NMR of 3ae (101 MHz, $CDCl_3$)

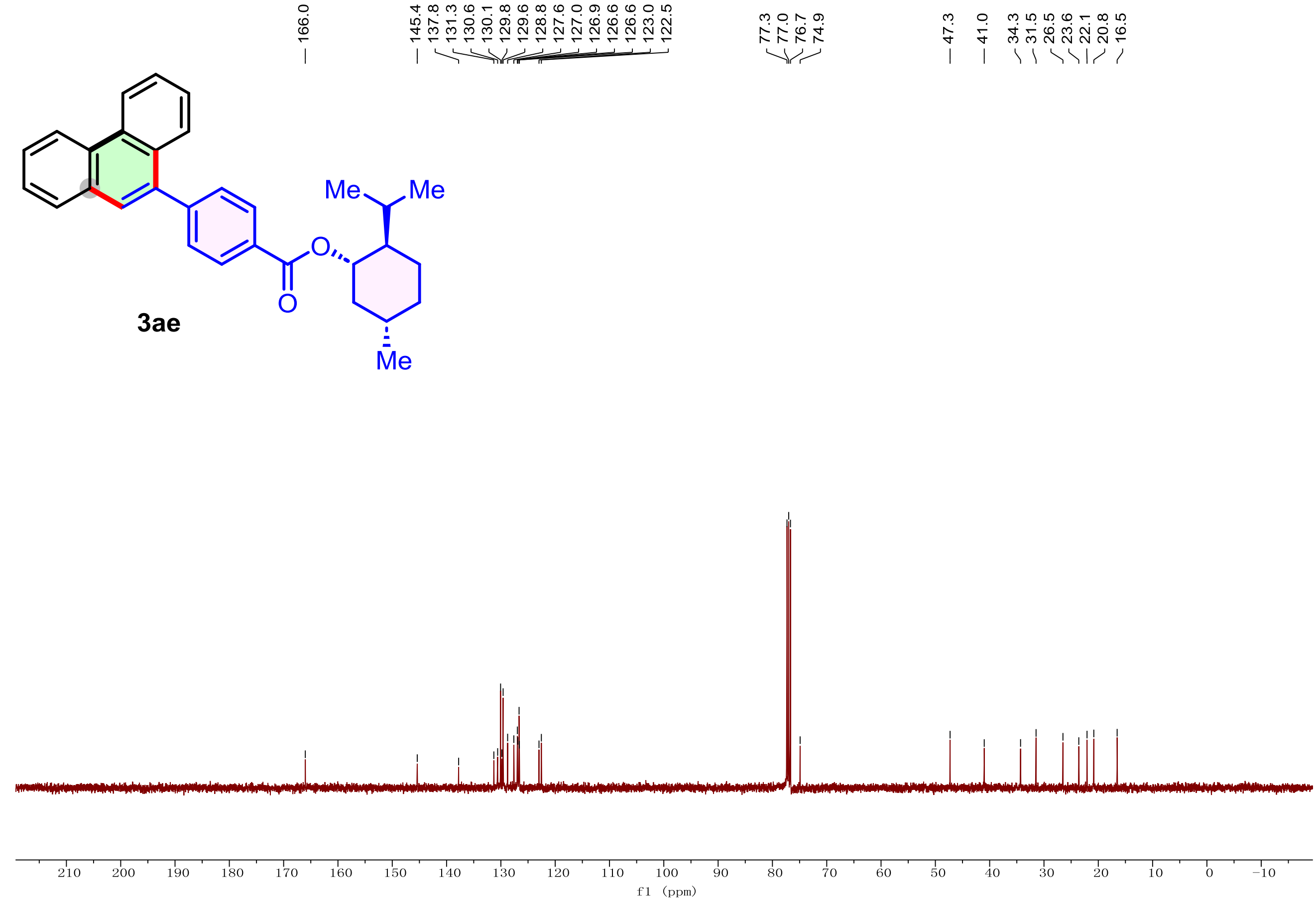

Supplementary Fig. 81. ^{1}H NMR of 3af (600 MHz, $CDCl_3$)

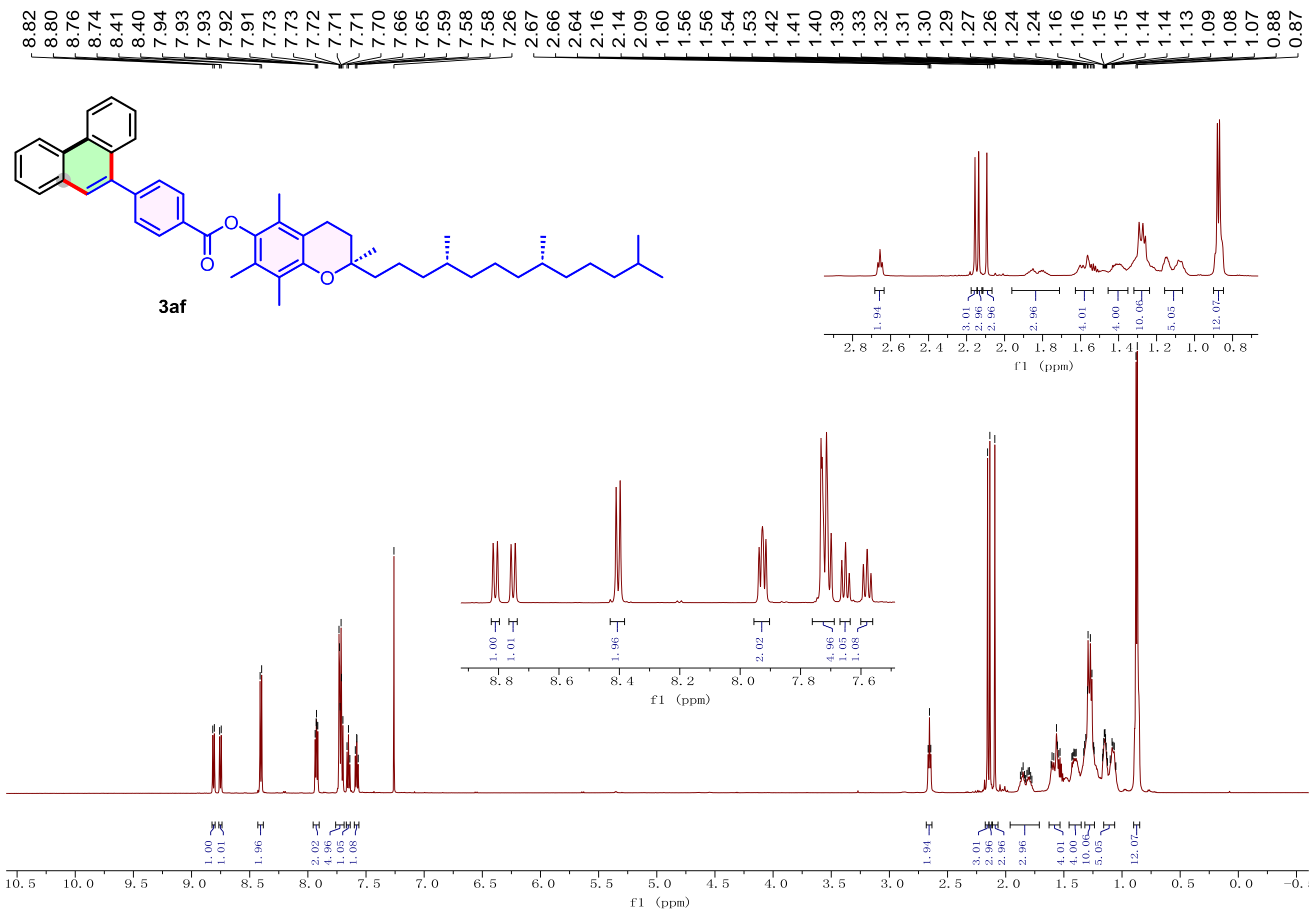

Supplementary Fig. 82. ^{13}C NMR of 3af (151 MHz, $CDCl_3$)

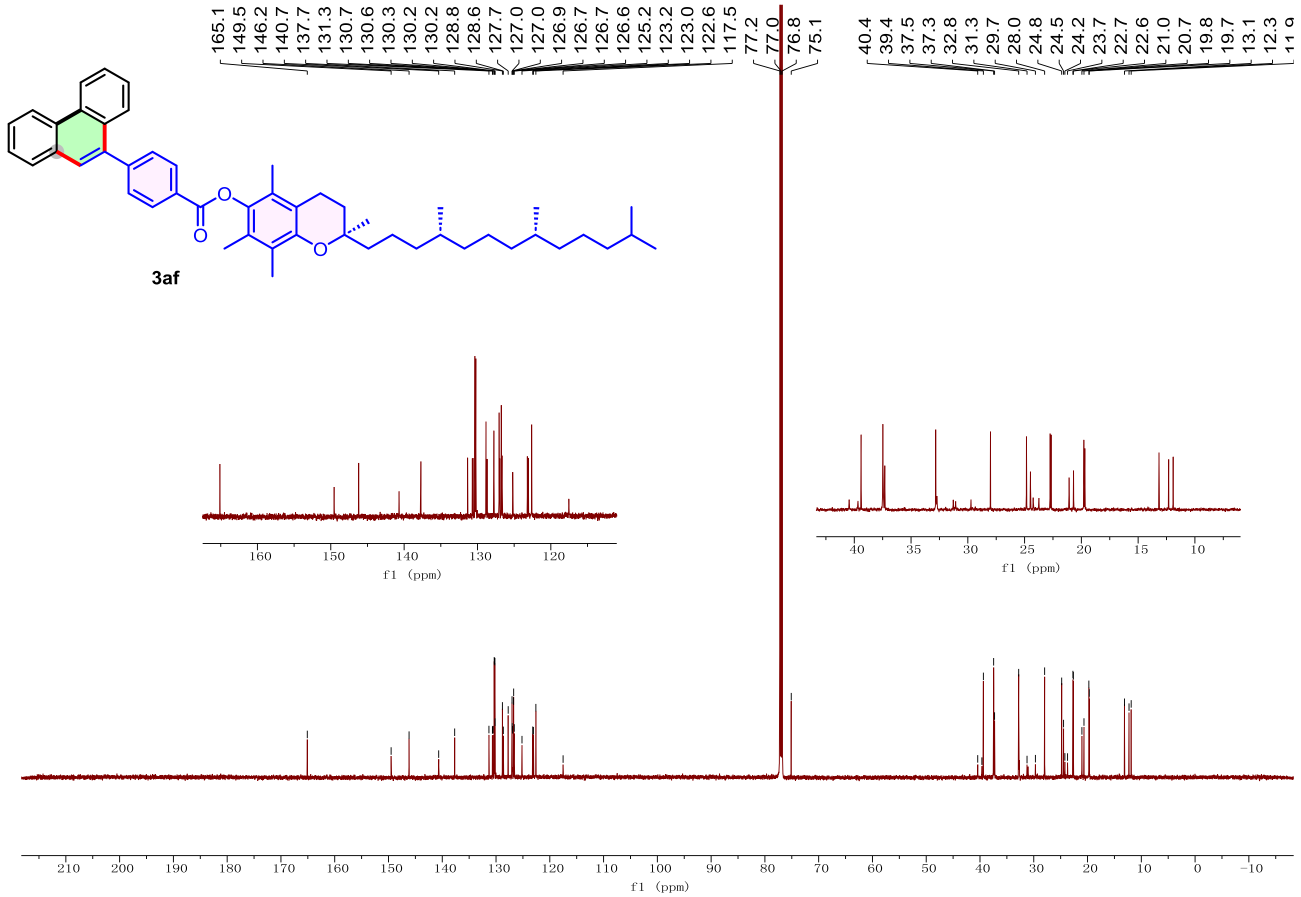

Supplementary Fig. 83. ^{1}H NMR of 3ag (400 MHz, $CDCl_3$)

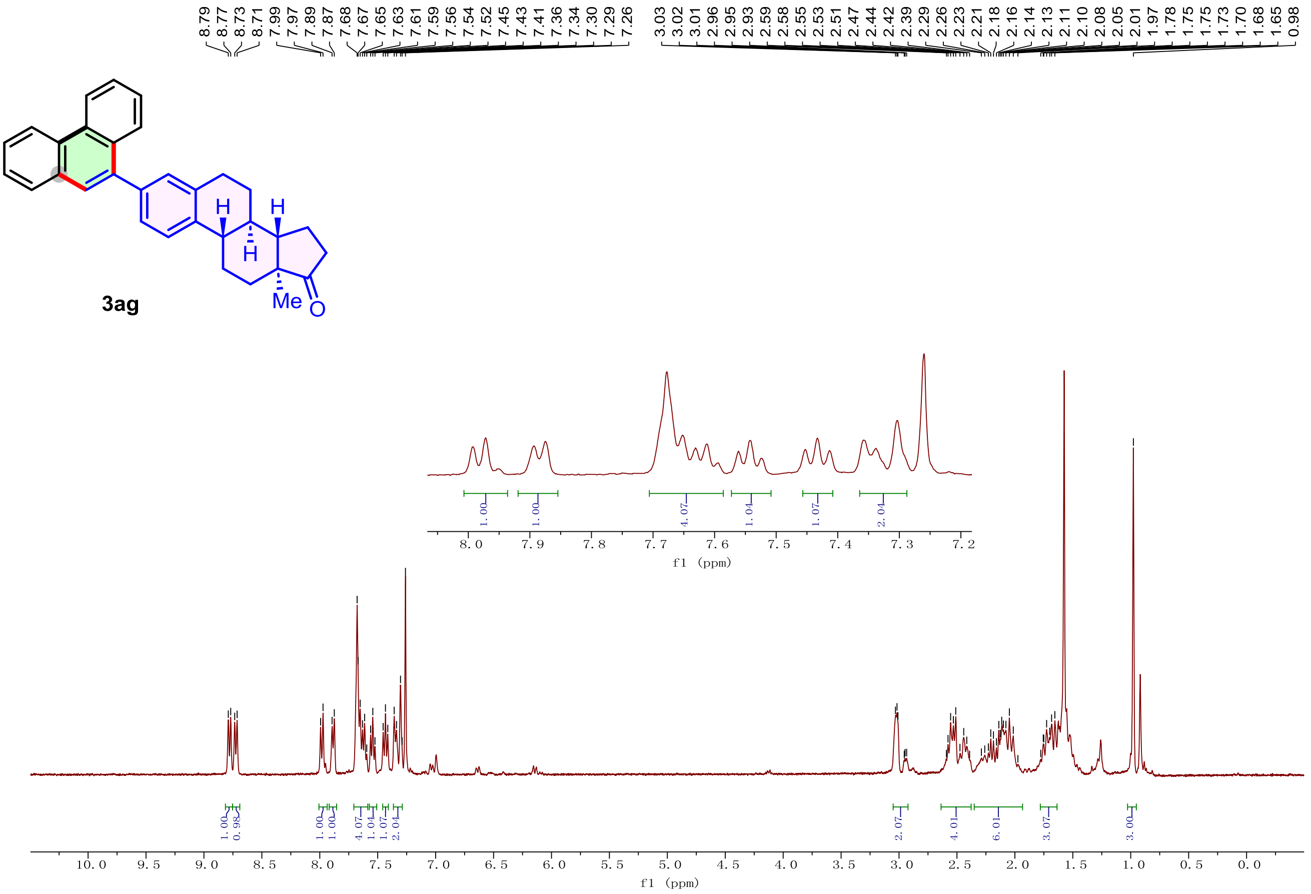

Supplementary Fig. 84. ^{13}C NMR of 3ag (101 MHz, $CDCl_3$)

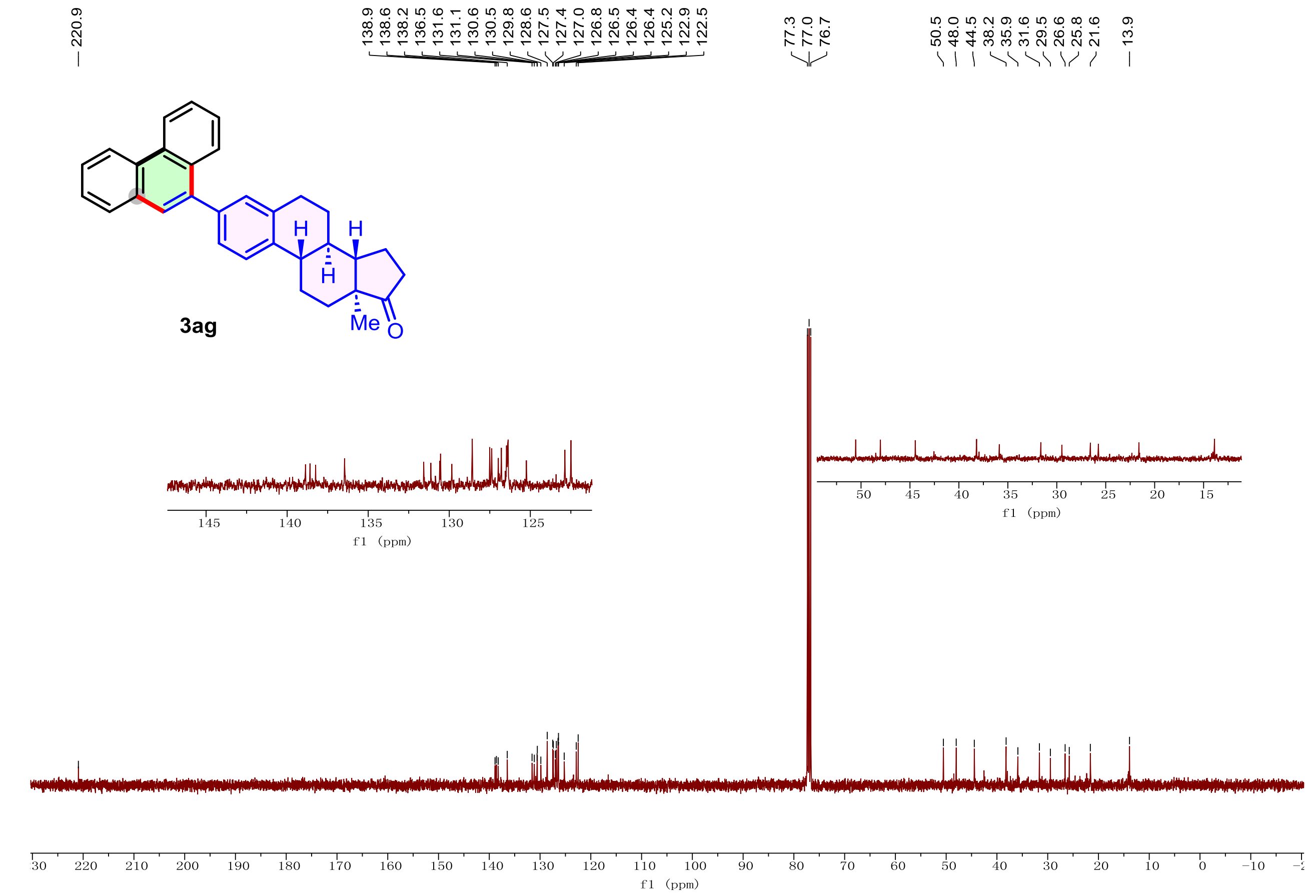

Supplementary Fig. 85. ^{1}H NMR of 4b (400 MHz, $CDCl_3$)

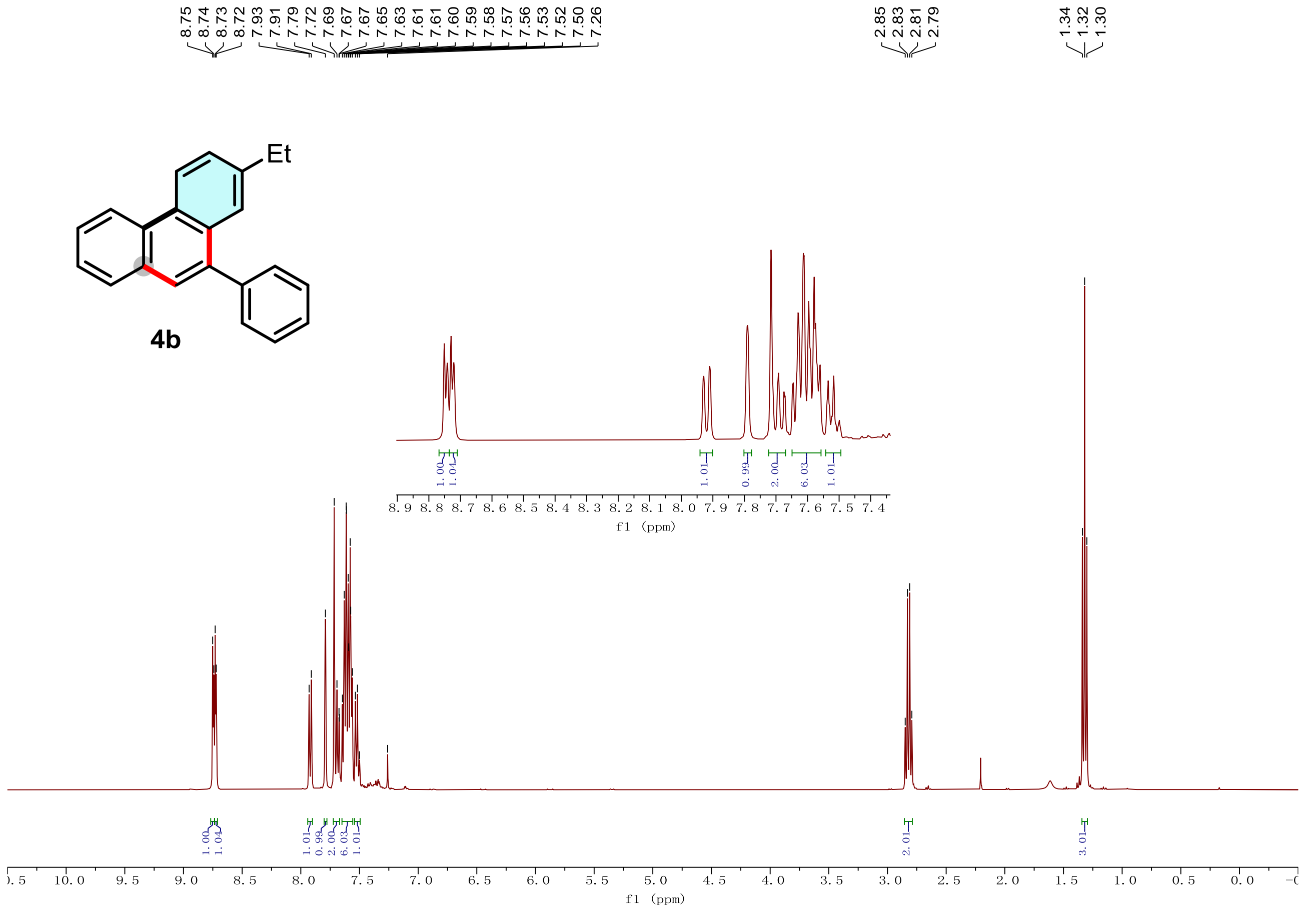

Supplementary Fig. 86. ^{13}C NMR of 4b (101 MHz, $CDCl_3$)

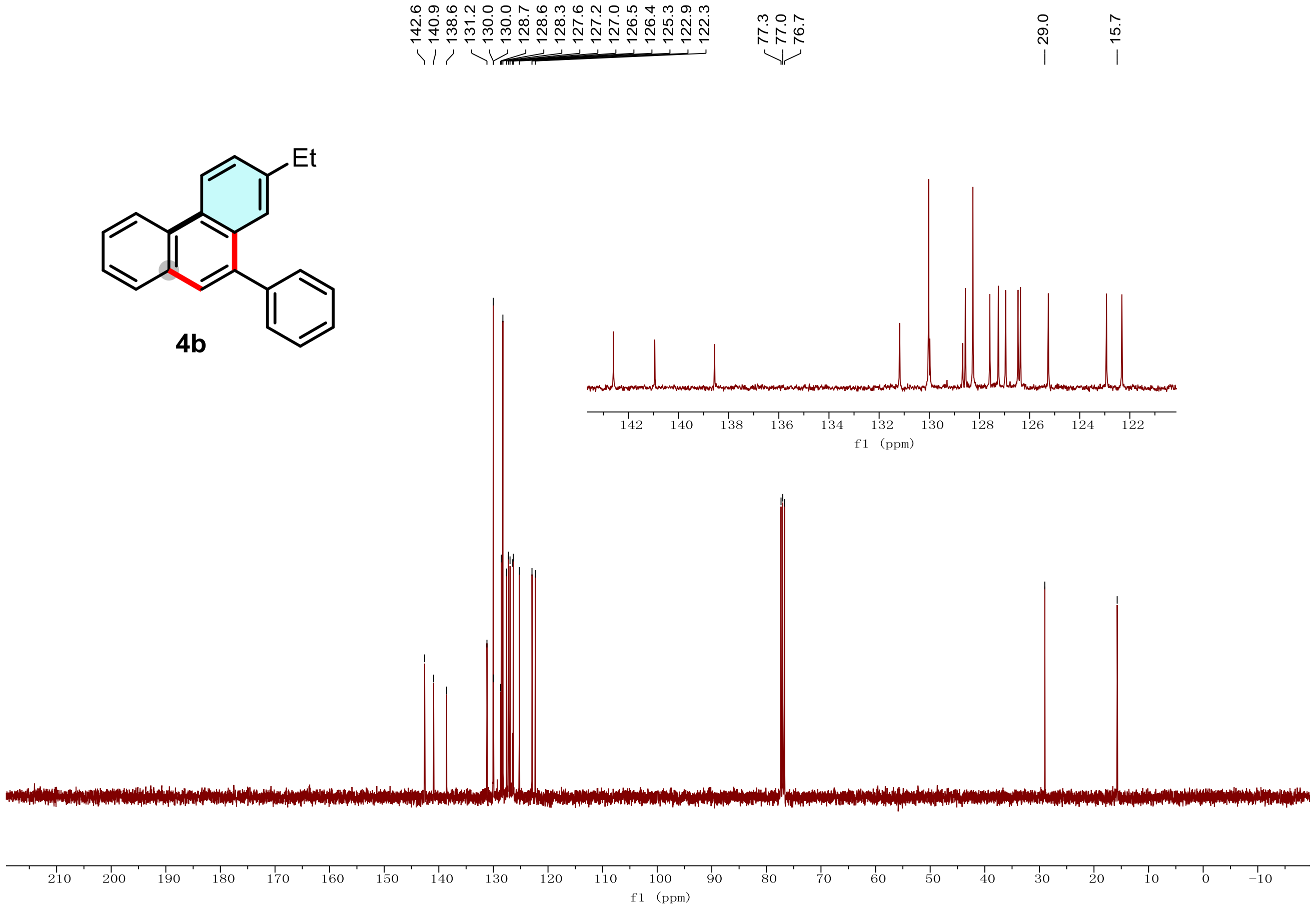

Supplementary Fig. 87. ^{1}H NMR of 4c (400 MHz, $CDCl_3$)

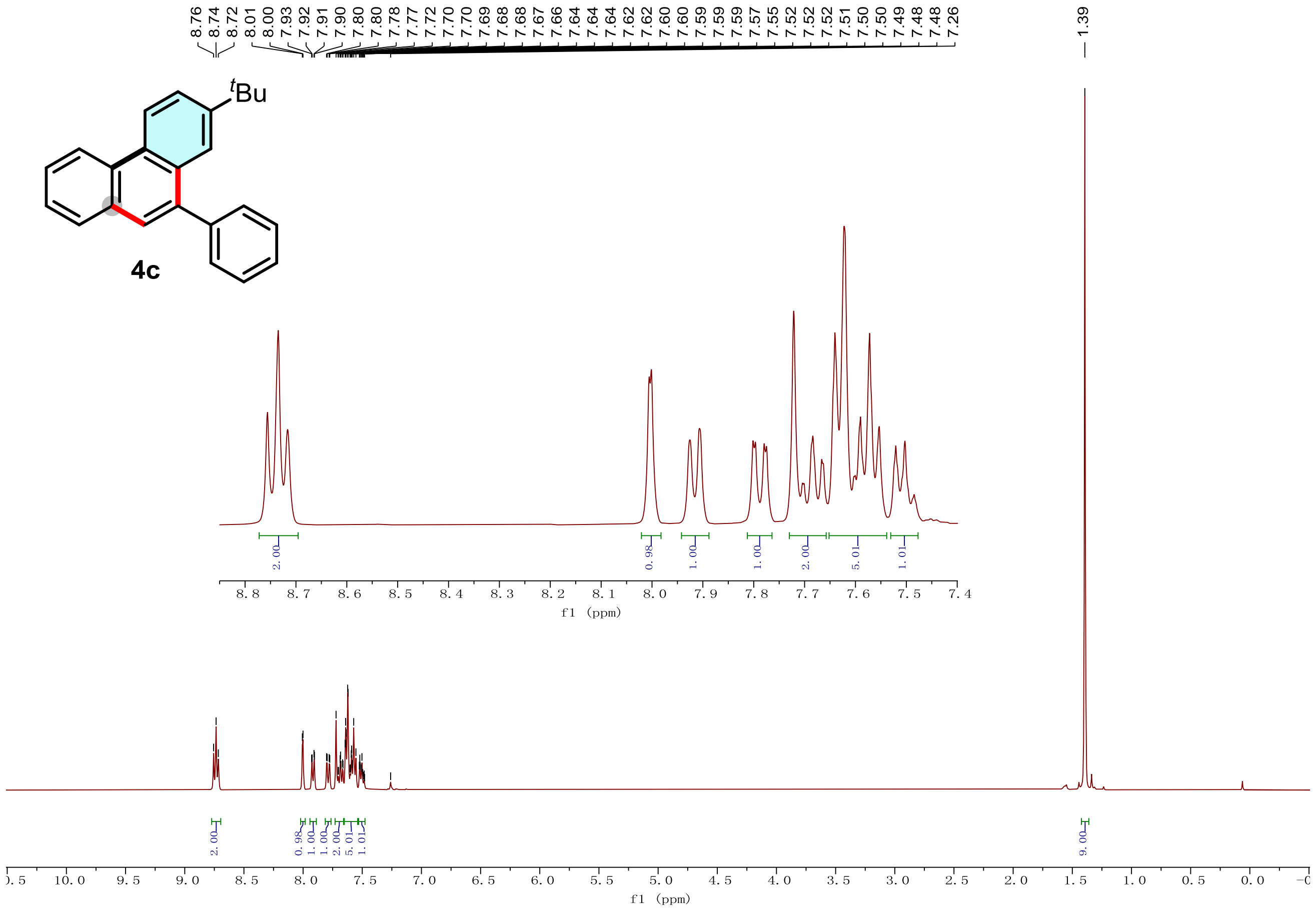

Supplementary Fig. 88. ^{13}C NMR of 4c (101 MHz, $CDCl_3$)

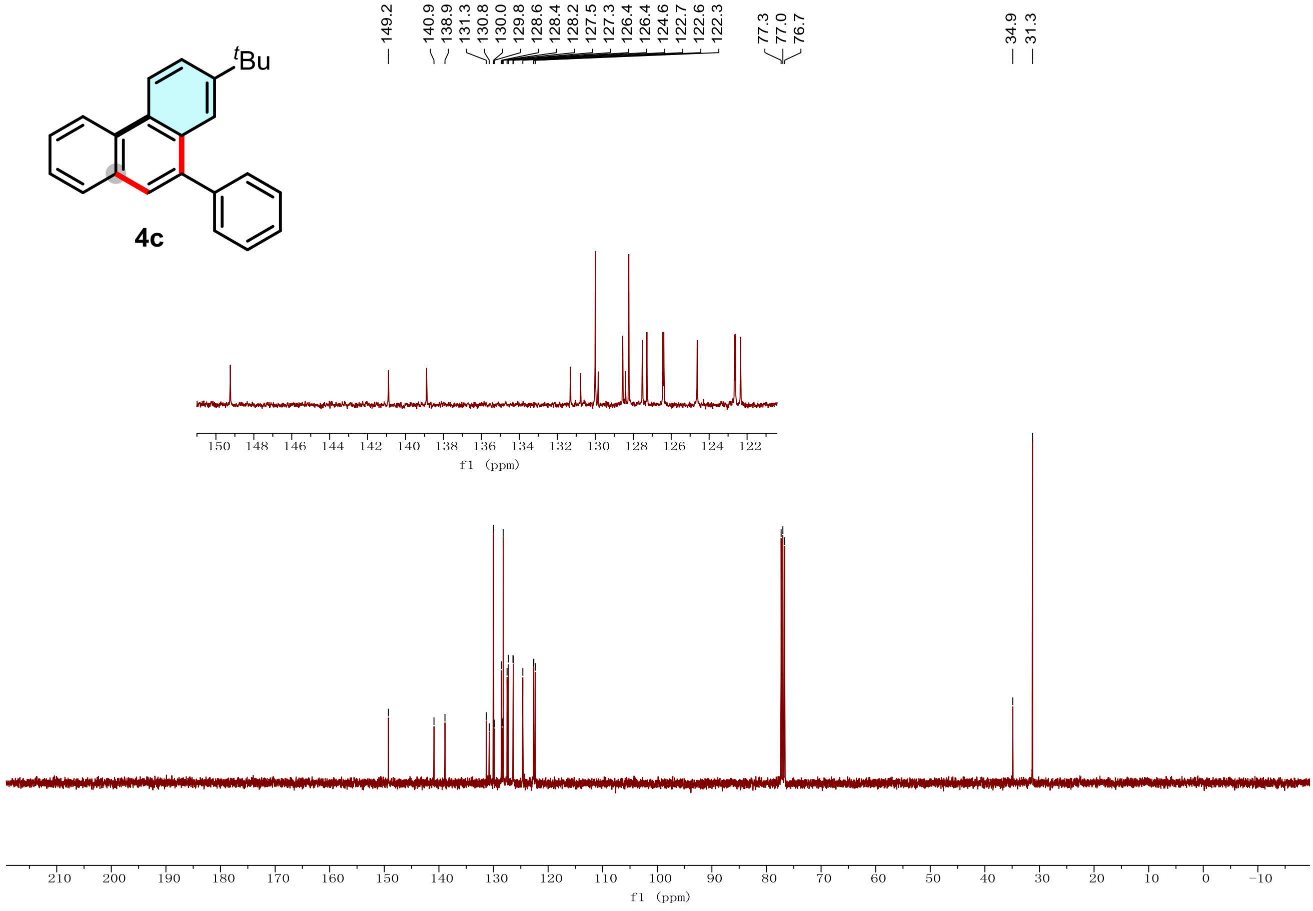

Supplementary Fig. 89. ^{1}H NMR of 4d (400 MHz, $CDCl_3$)

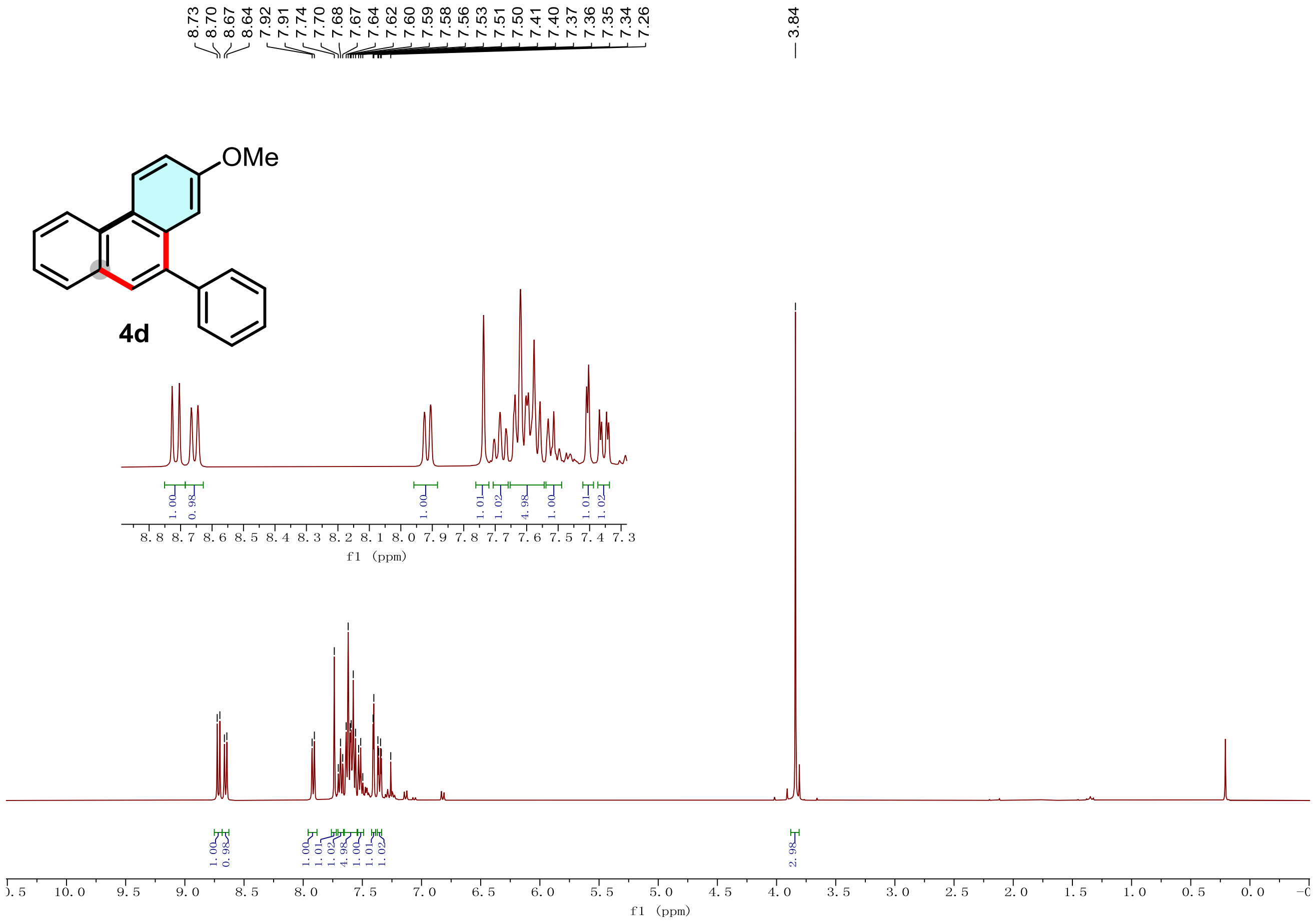

Supplementary Fig. 90. ^{13}C NMR of 4d (101 MHz, $CDCl_3$)

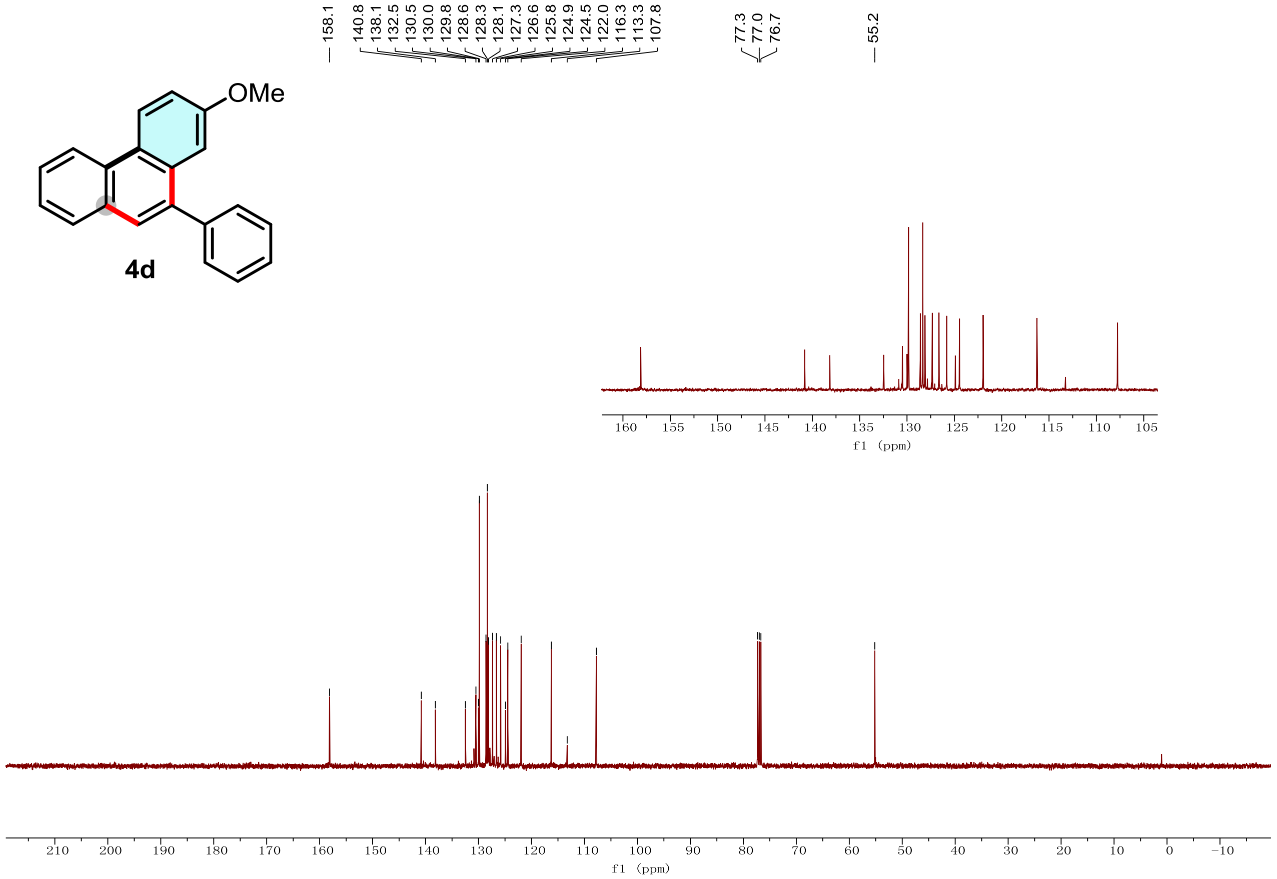

Supplementary Fig.91. ^{1}H NMR of 4e (400 MHz, $CDCl_3$)

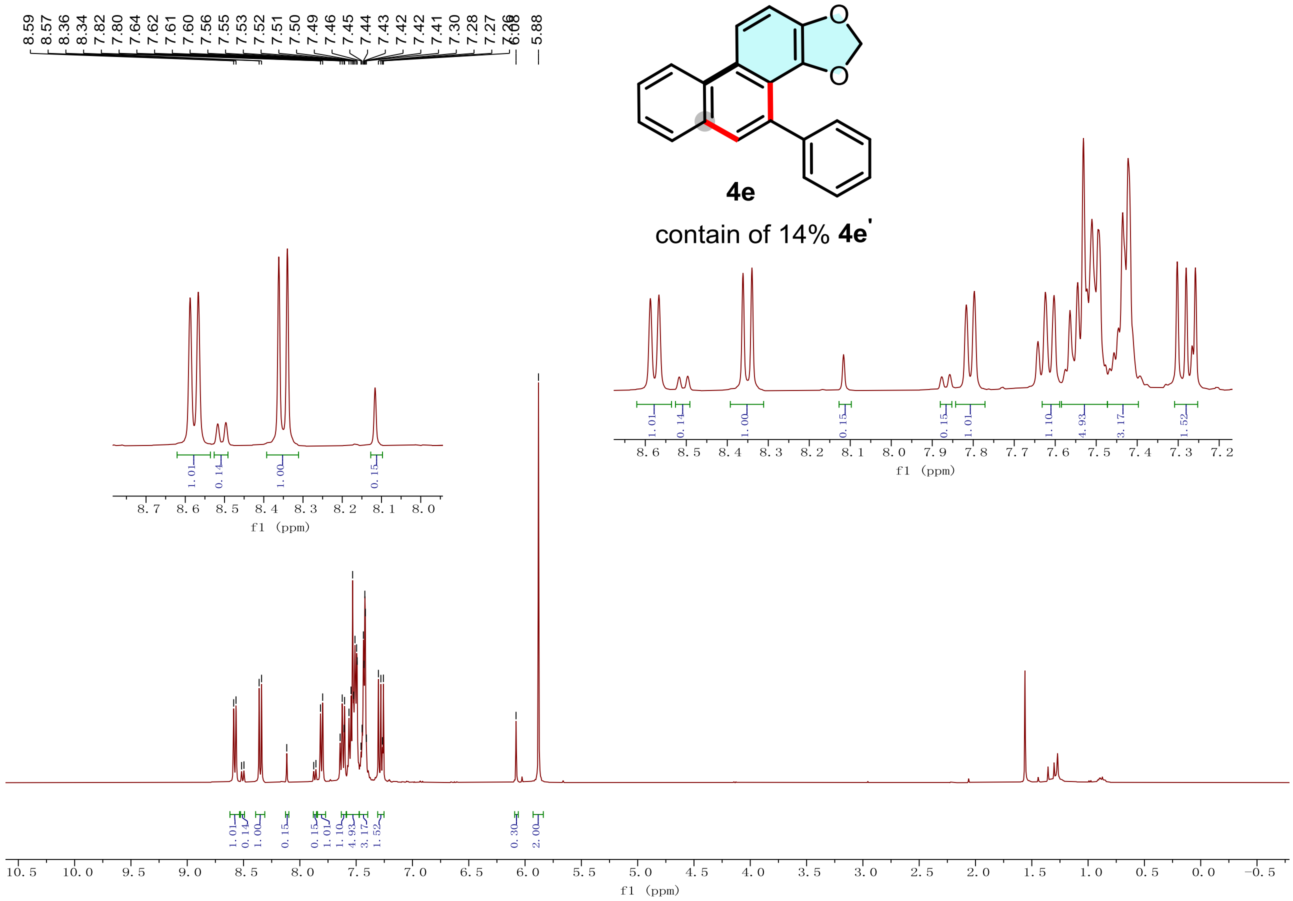

Supplementary Fig. 92. ^{13}C NMR of 4e (101 MHz, $CDCl_3$)

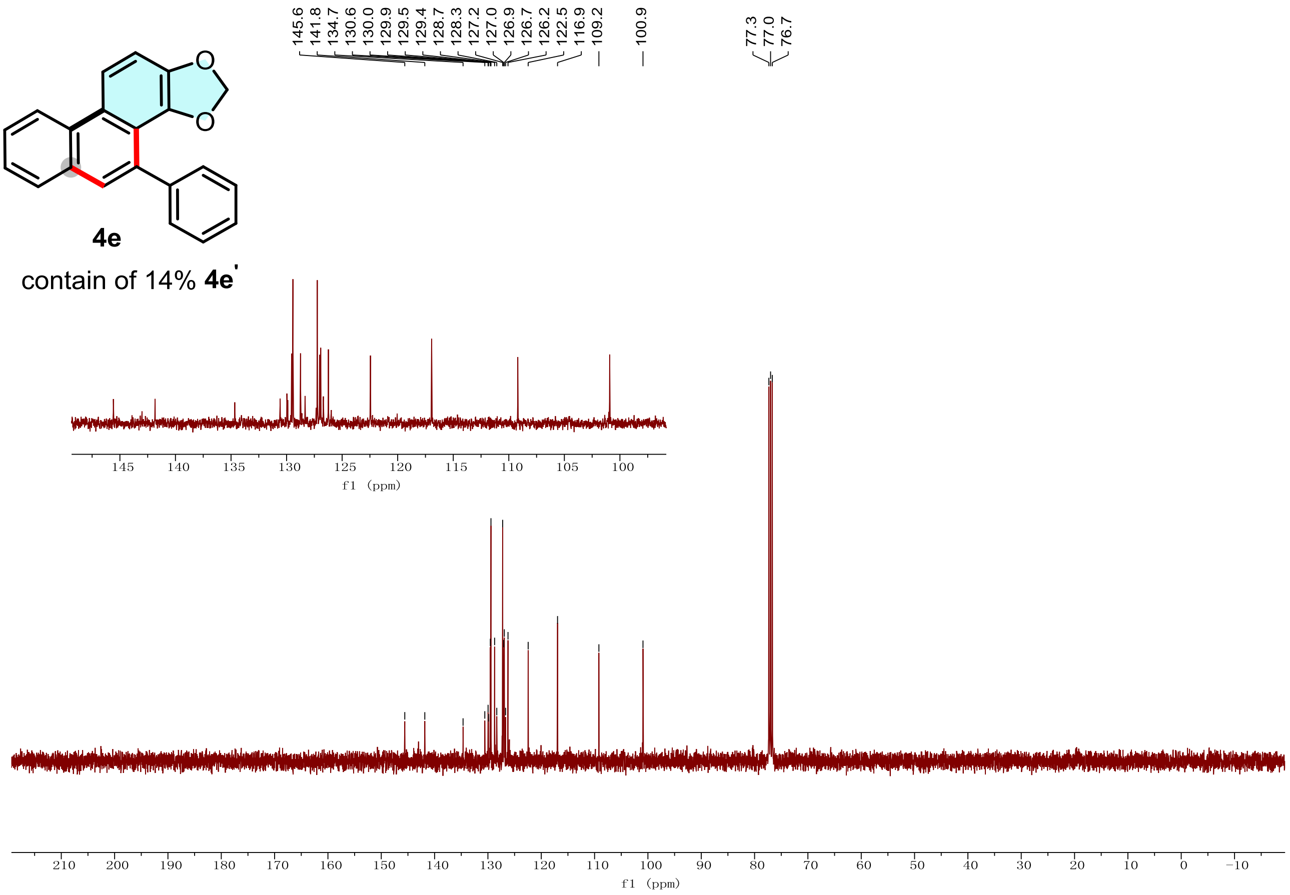

Supplementary Fig. 93. ^{1}H NMR of 4e' (400 MHz, $CDCl_3$)

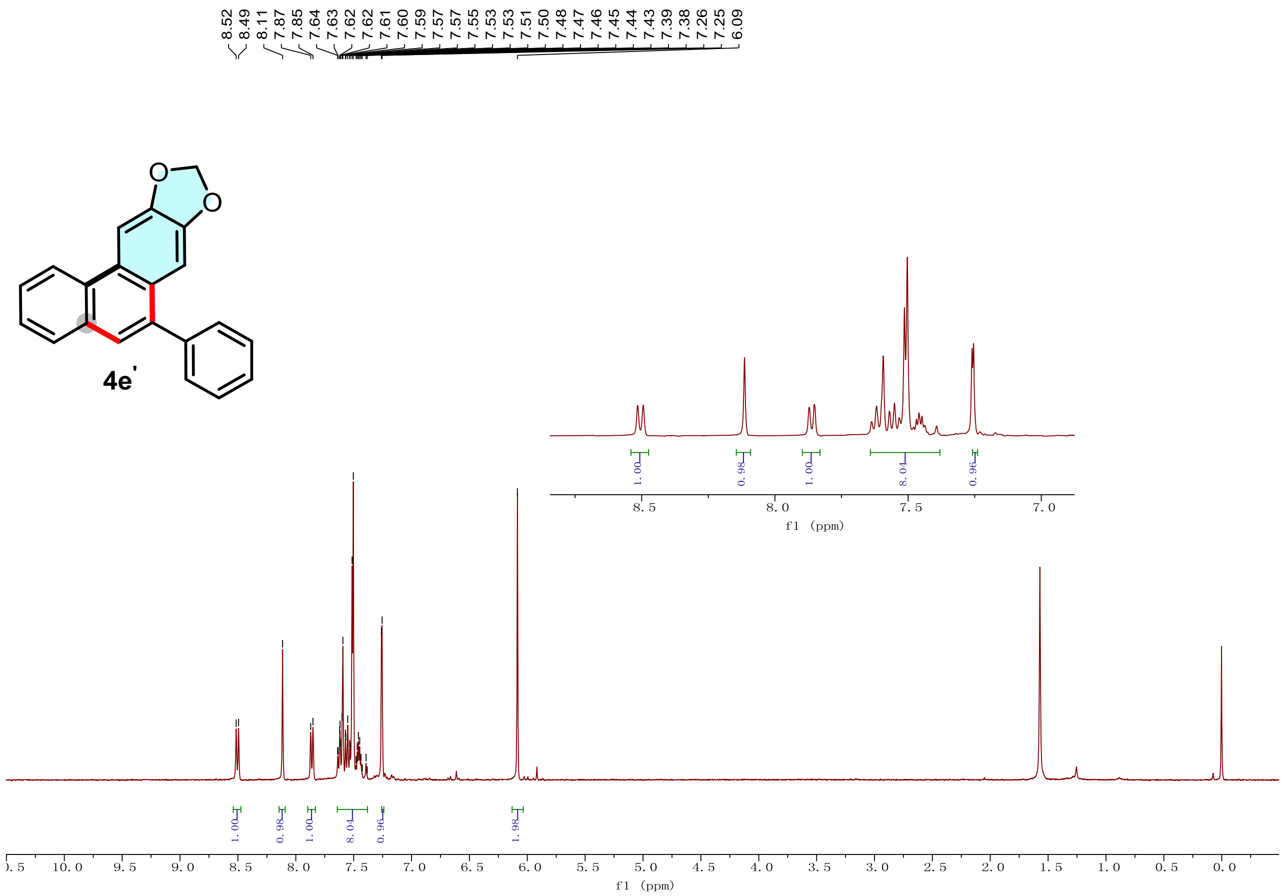

Supplementary Fig. 94. ^{13}C NMR of 4e' (101 MHz, $CDCl_3$)

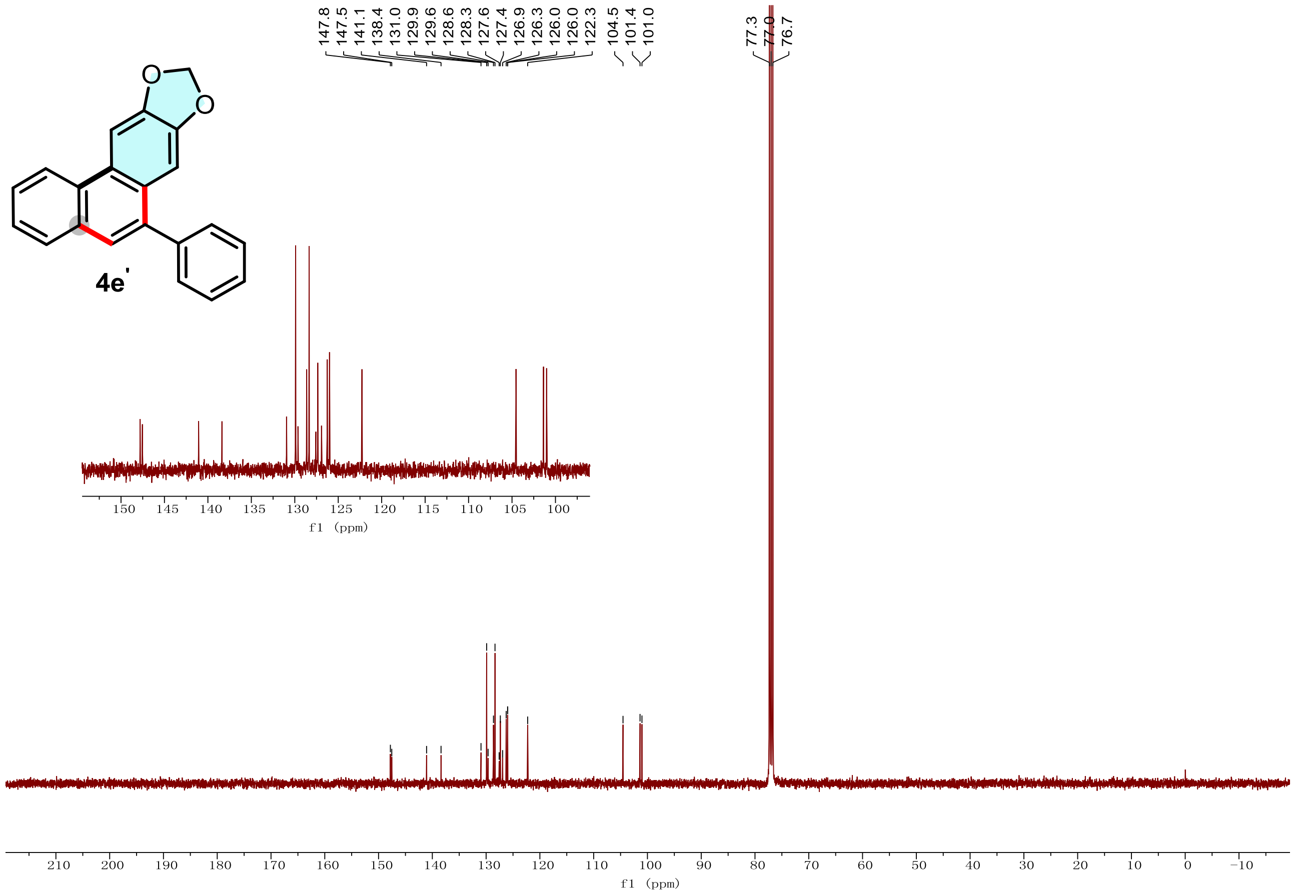

Supplementary Fig. 95. ^{1}H NMR of 4f (400 MHz, $CDCl_3$)

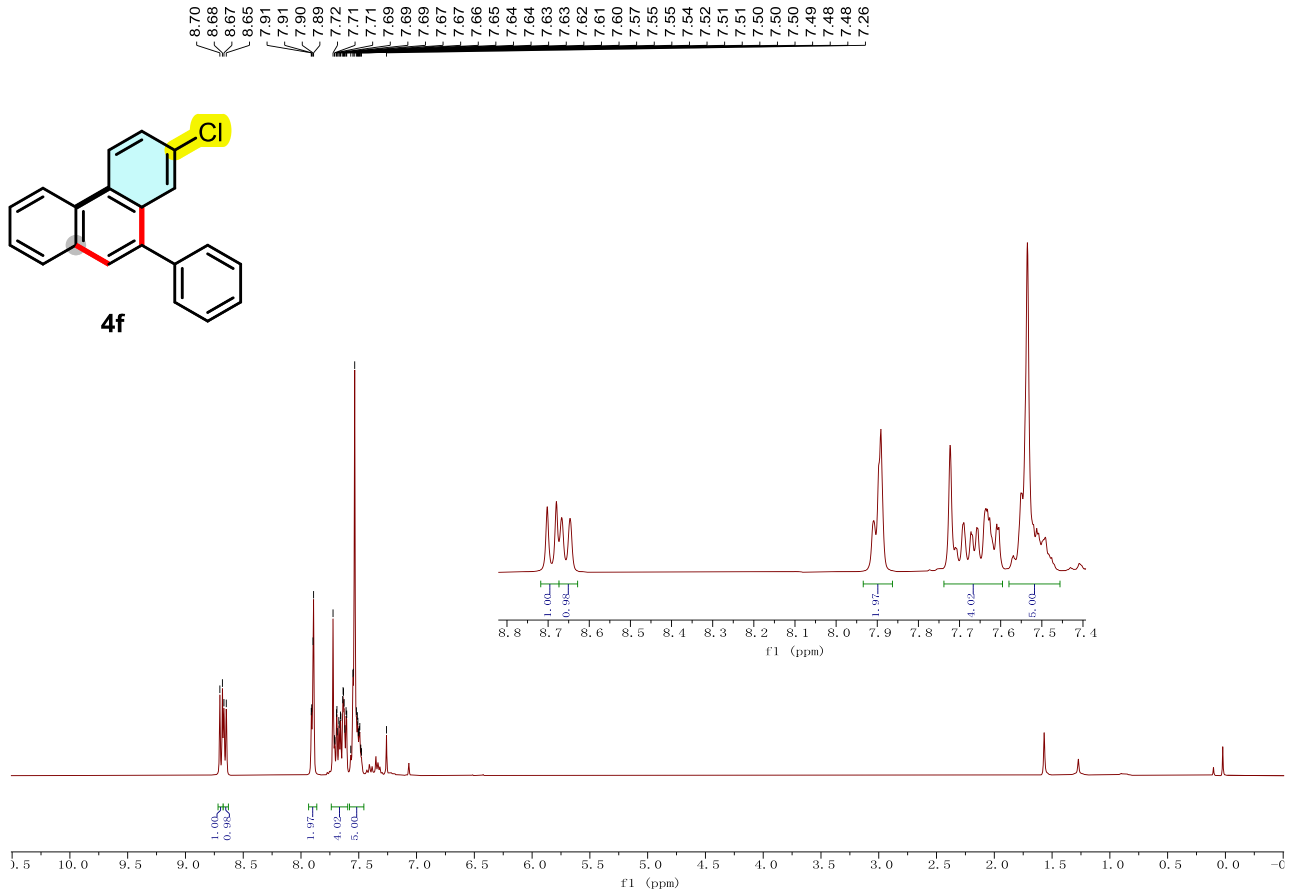

Supplementary Fig. 96. ^{13}C NMR of 4f (101 MHz, $CDCl_3$)

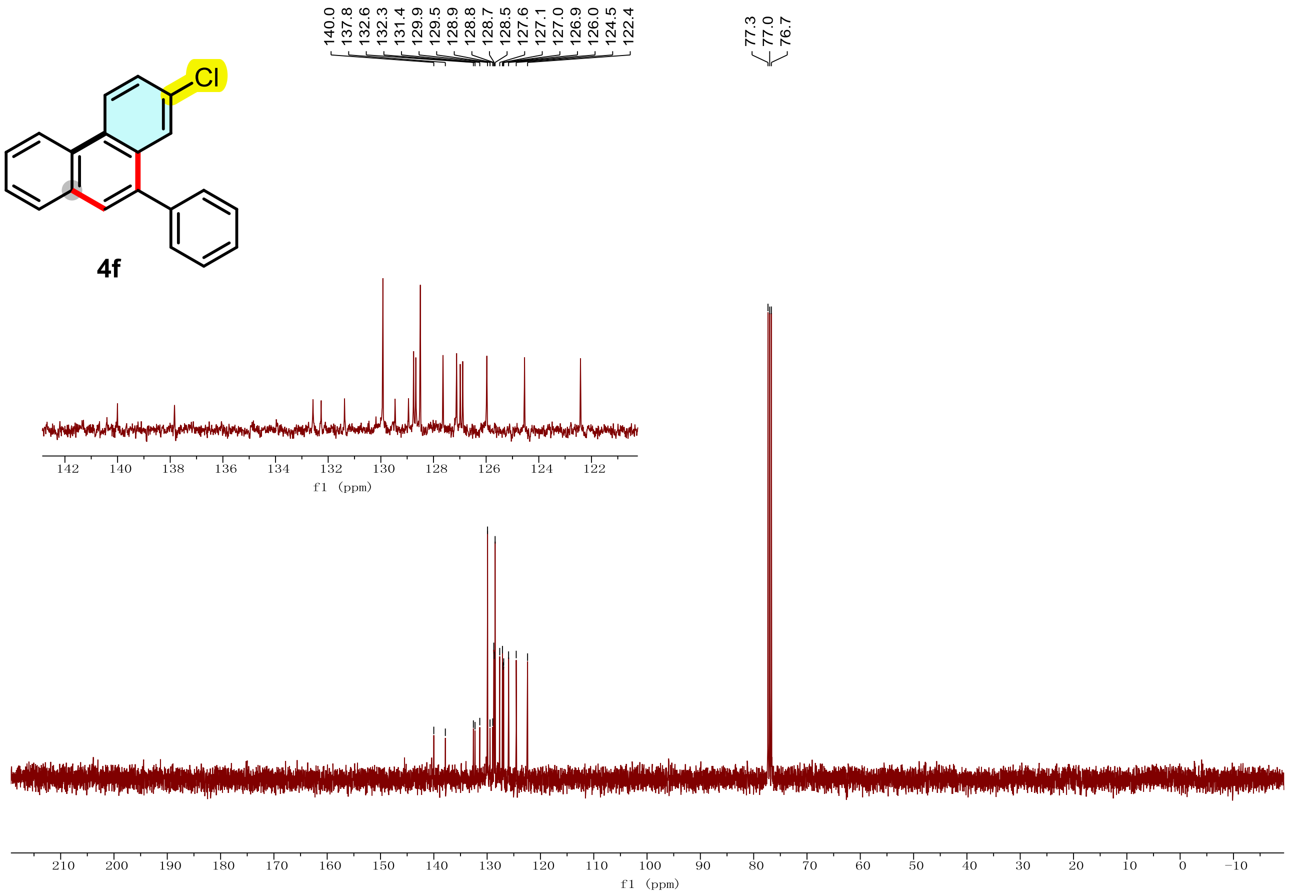

Supplementary Fig. 97. ^{1}H NMR of 4g (400 MHz, $CDCl_3$)

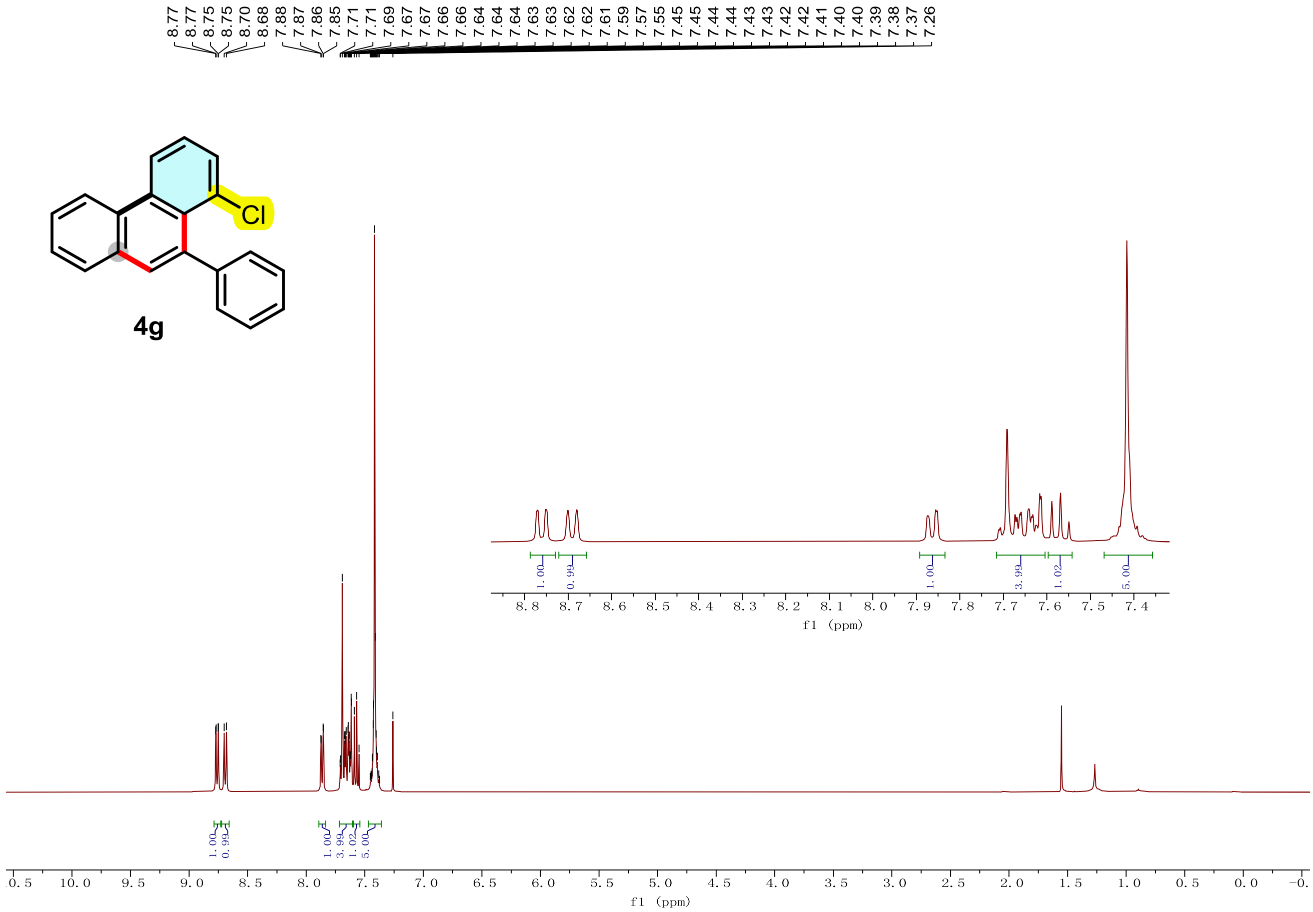

Supplementary Fig. 98. ^{13}C NMR of 4g (101 MHz, $CDCl_3$)

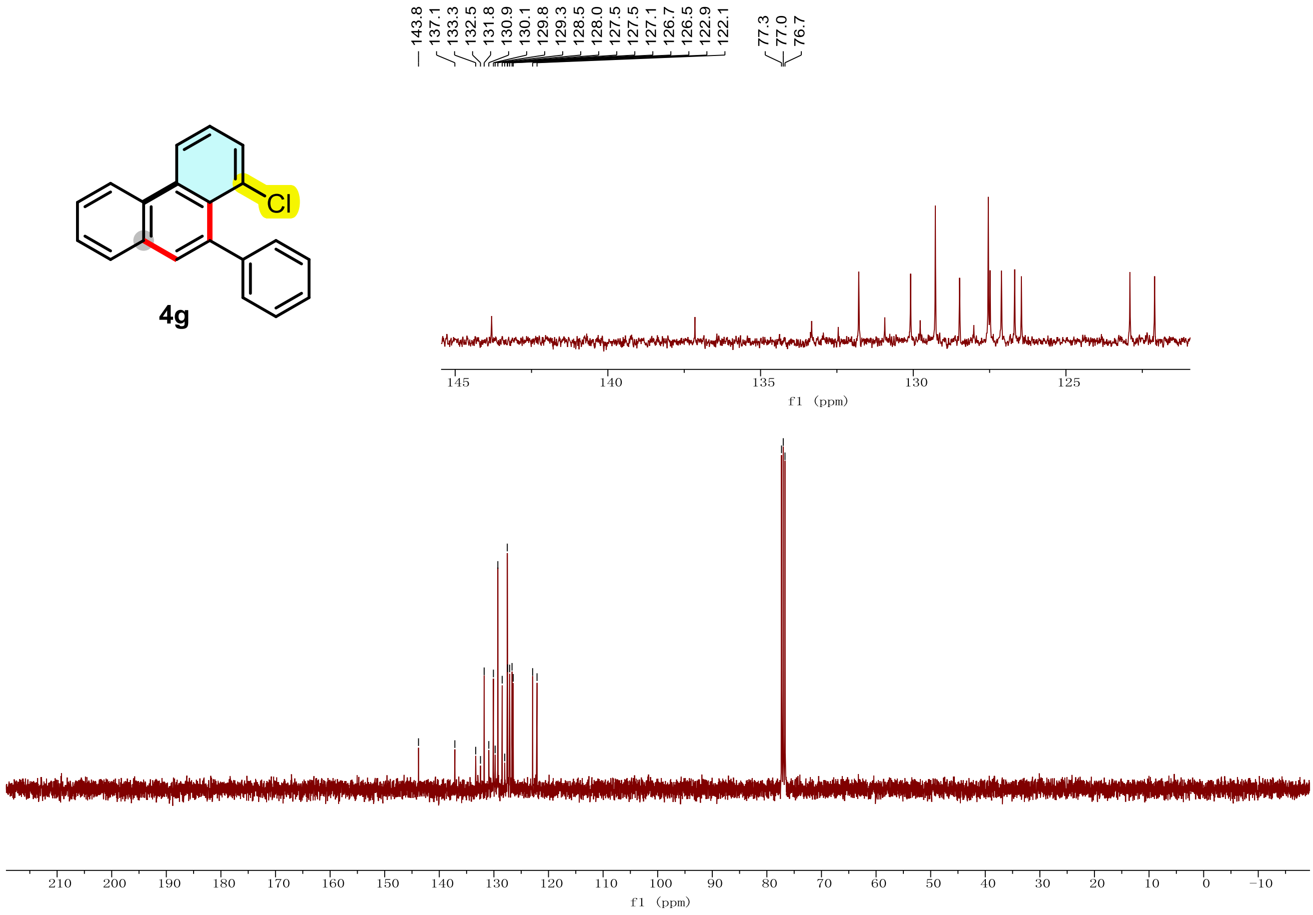

Supplementary Fig. 99. ^{1}H NMR of 4g' (400 MHz, $CDCl_3$)

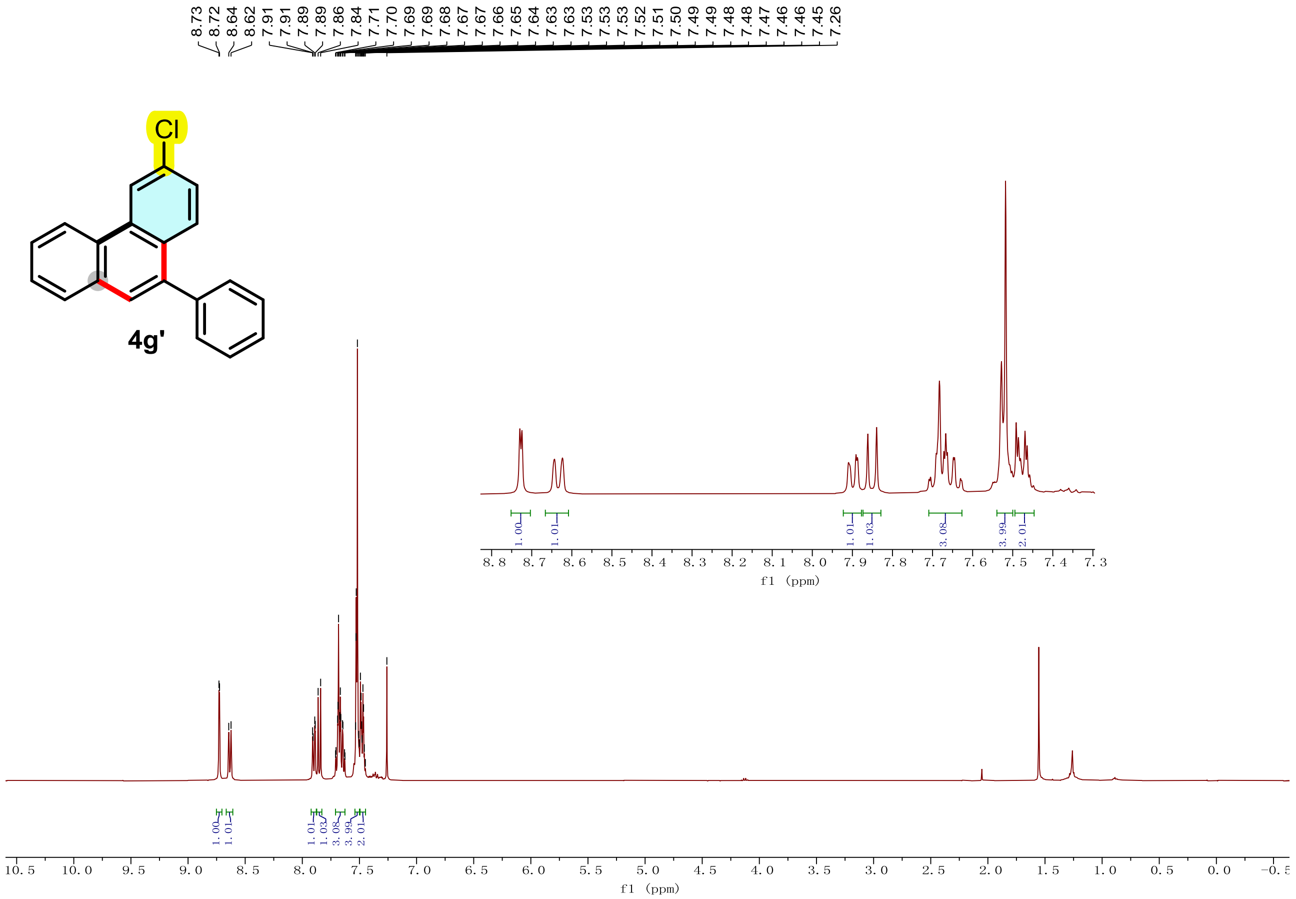

Supplementary Fig. 100. ^{13}C NMR of 4g' (101 MHz, $CDCl_3$)

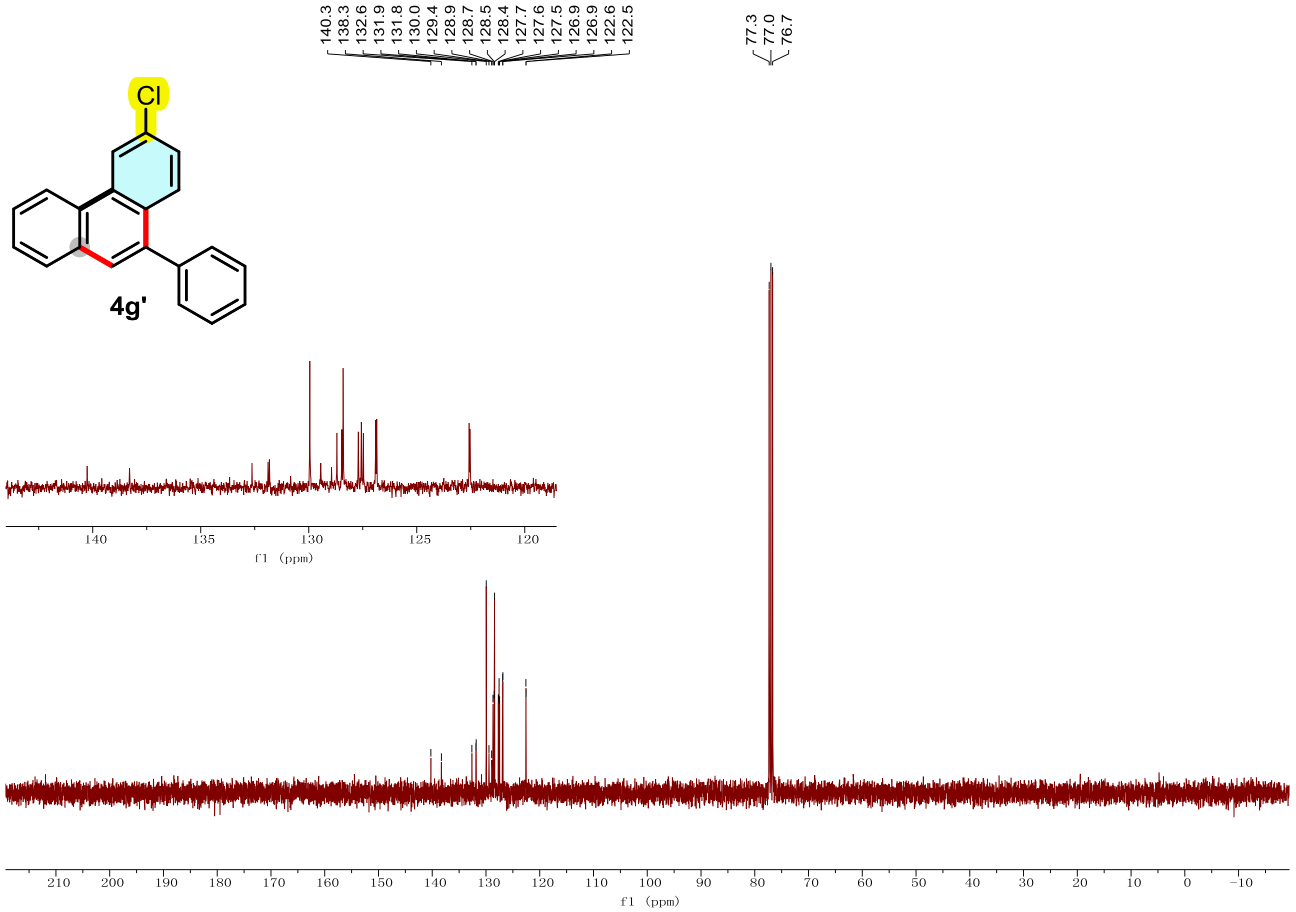

Supplementary Fig. 101. ^{1}H NMR of 4h (400 MHz, $CDCl_3$)

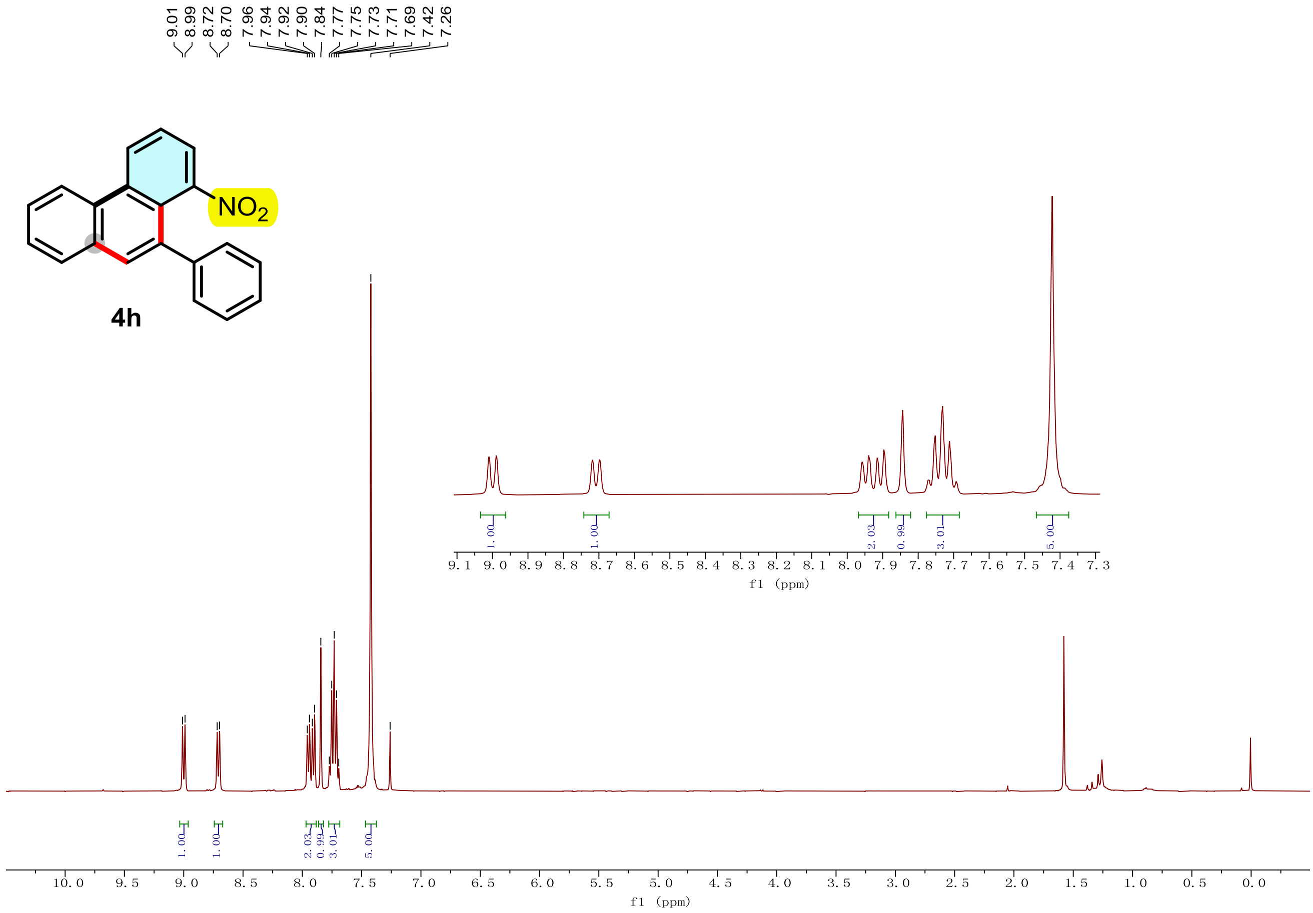

Supplementary Fig.102. ^{13}C NMR of 4h (101 MHz, $CDCl_3$)

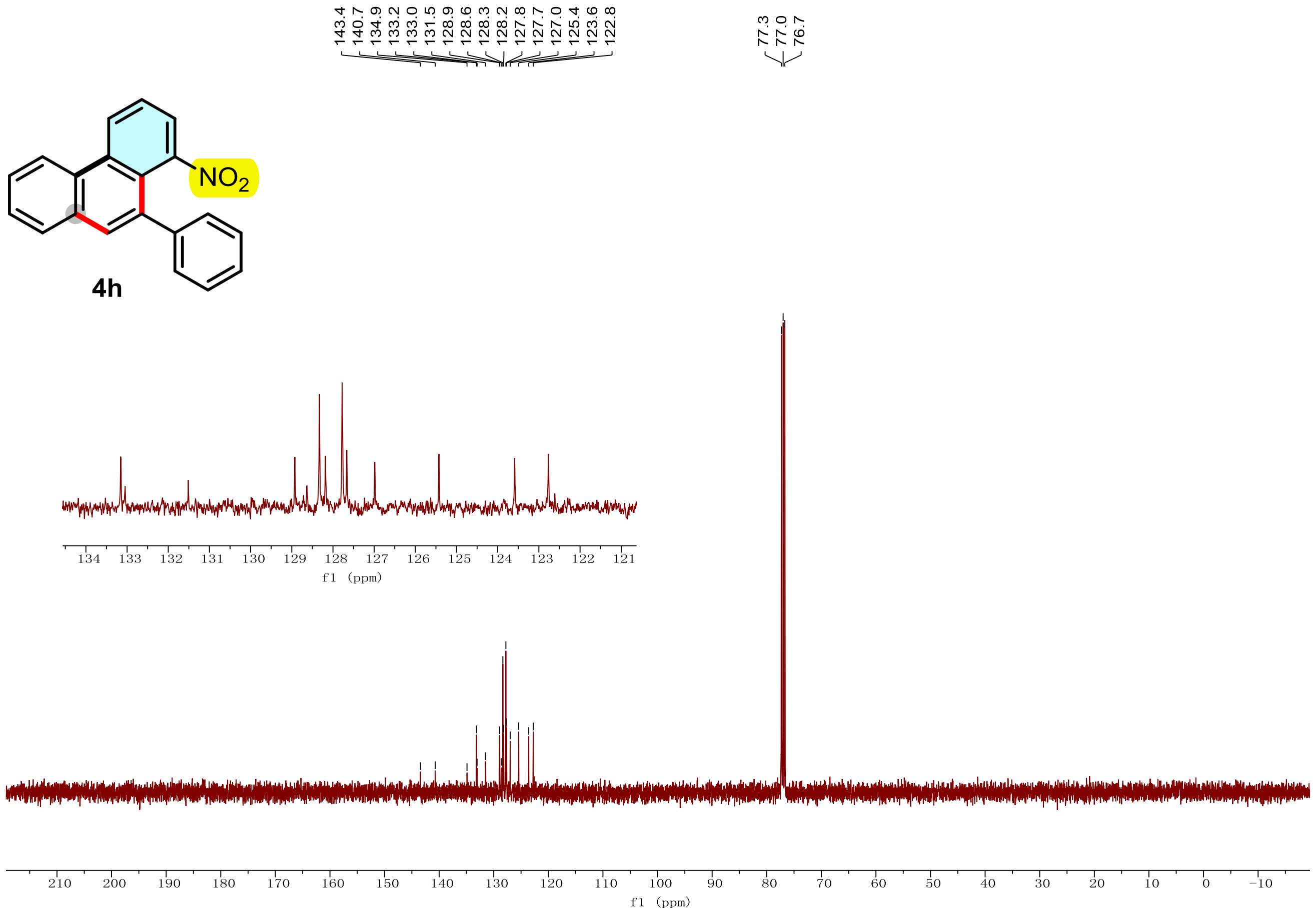

Supplementary Fig. 103. 4h', ^{1}H NMR of 4h' (600 MHz, $CDCl_3$)

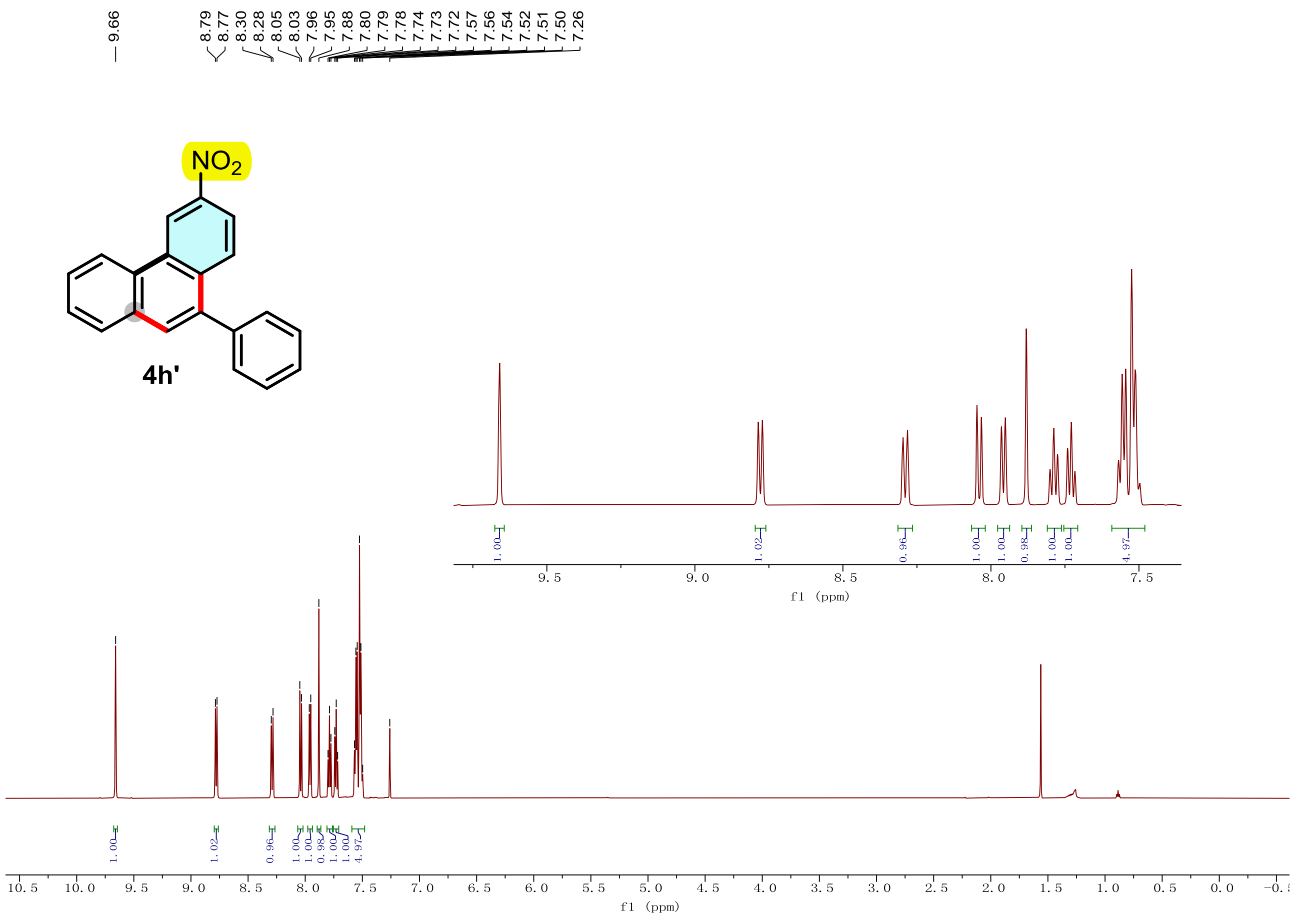

Supplementary Fig. 104. ^{13}C NMR of 4h' (151 MHz, $CDCl_3$)

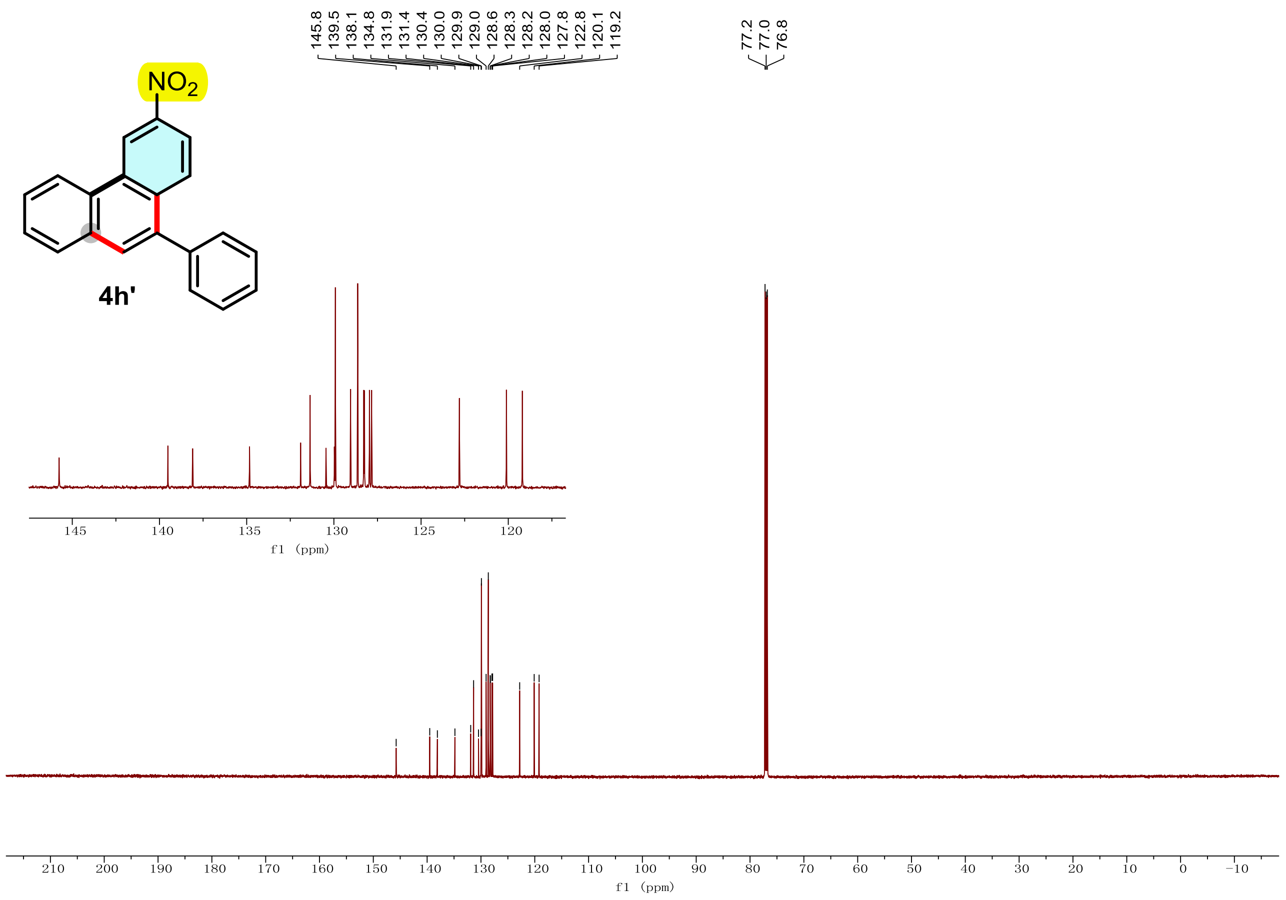

Supplementary Fig. 105. ^{1}H NMR of 4i (400 MHz, $CDCl_3$)

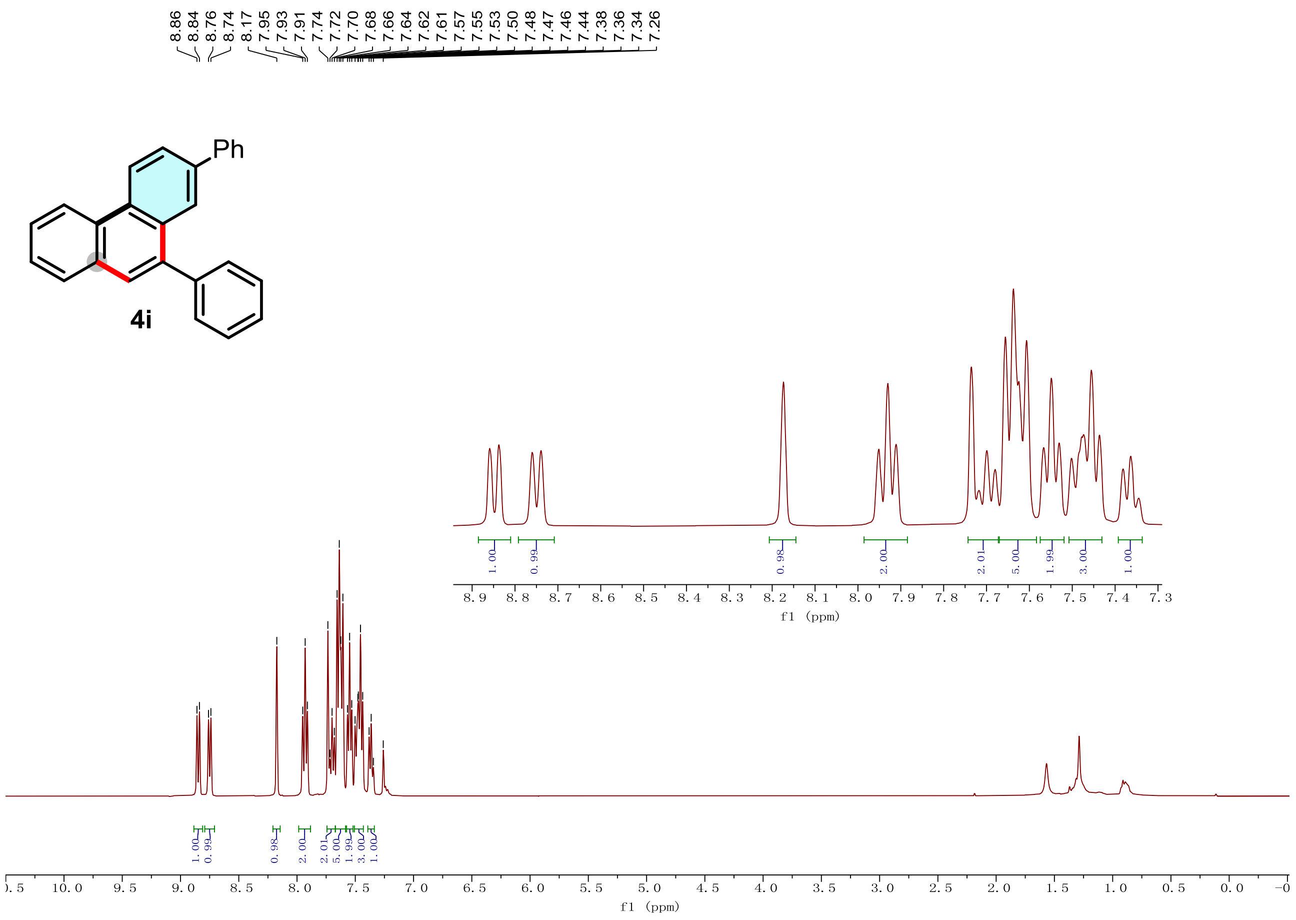

Supplementary Fig. 106. ^{13}C NMR of 4i (101 MHz, $CDCl_3$)

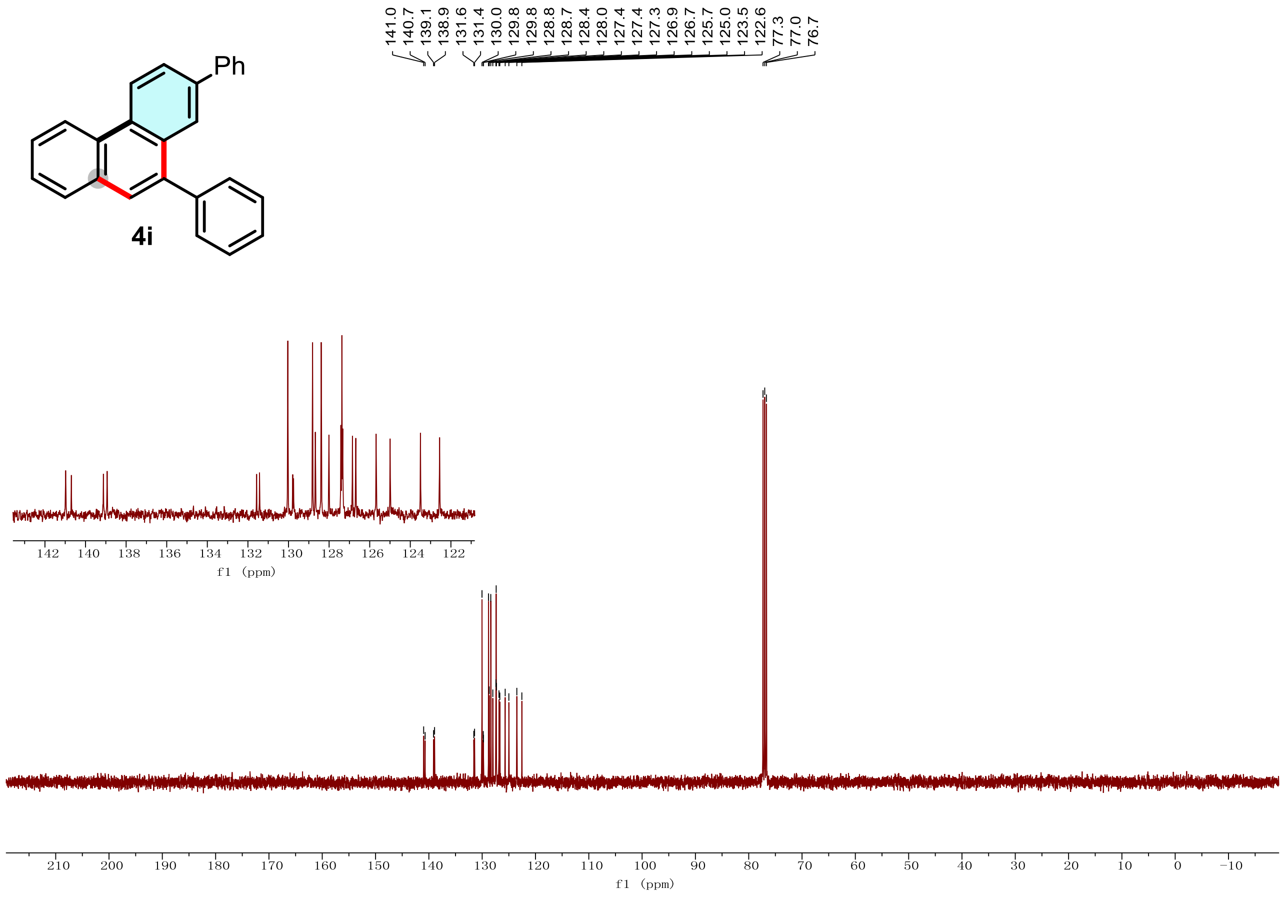

Supplementary Fig. 107. ^{1}H NMR of 4j (400 MHz, $CDCl_3$)

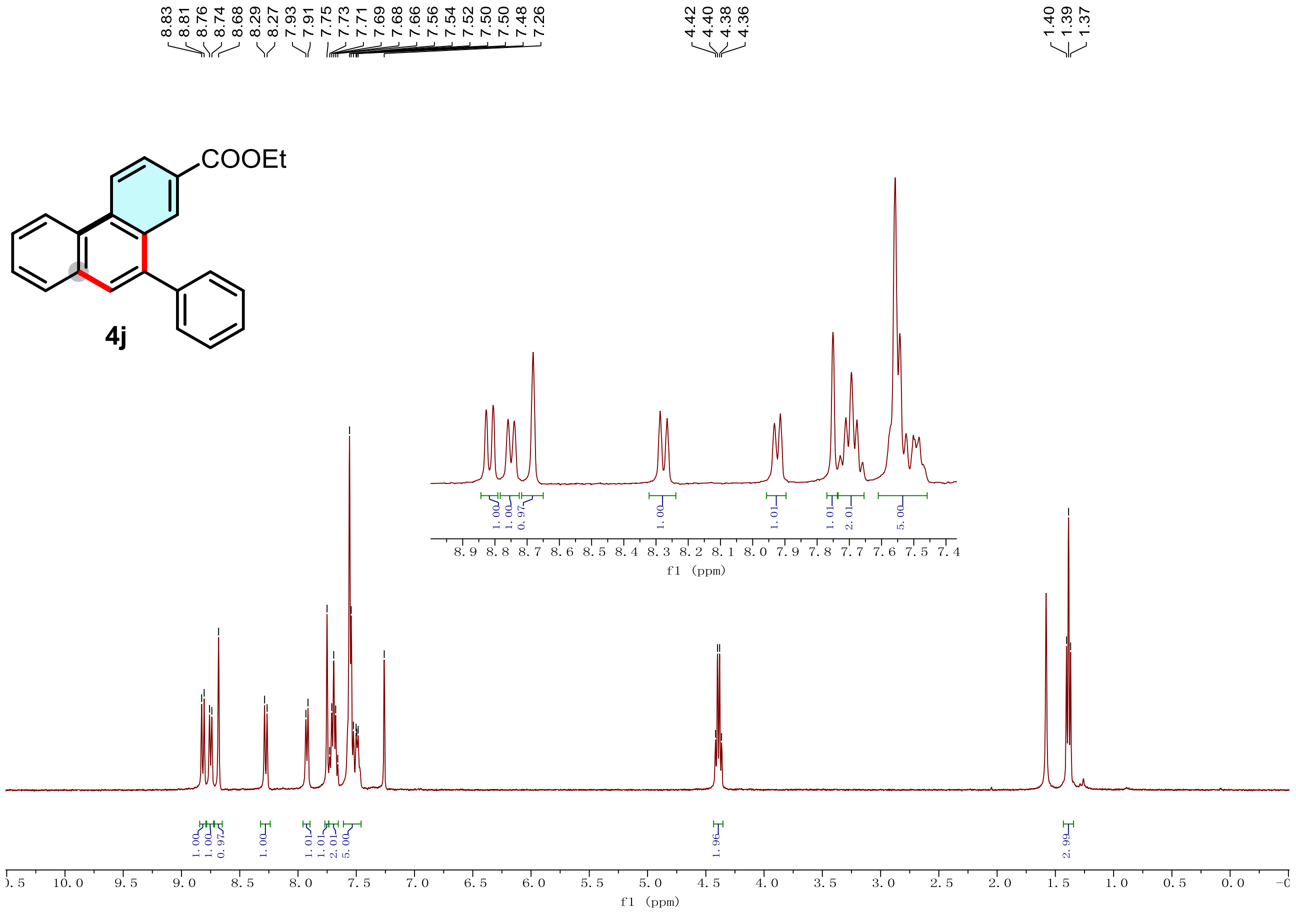

Supplementary Fig. 108. ^{13}C NMR of 4j (101 MHz, $CDCl_3$)

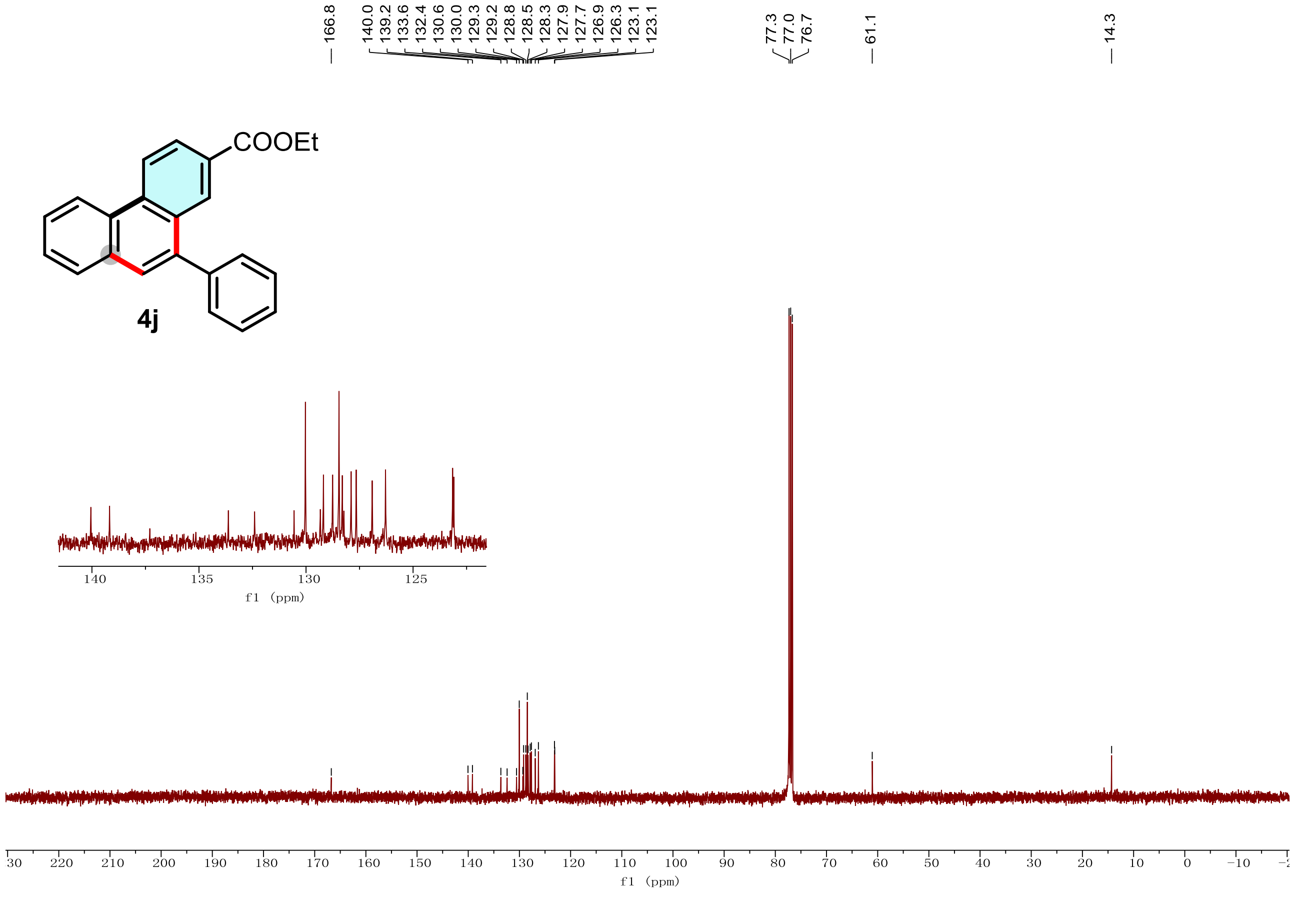

Supplementary Fig. 109. ^{1}H NMR of 4k (400 MHz, $CDCl_3$)

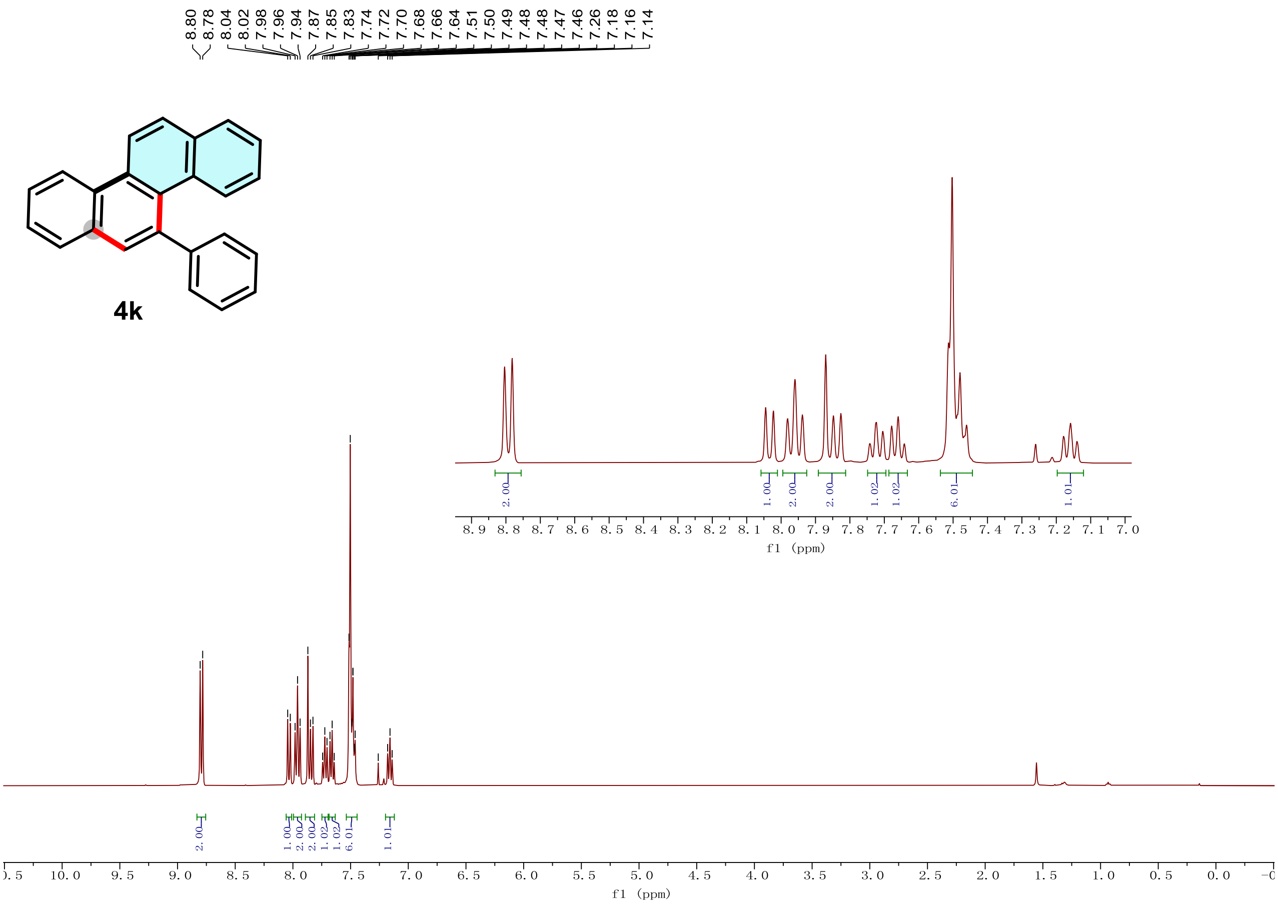

Supplementary Fig. 110. ^{13}C NMR of 4k (101 MHz, $CDCl_3$)

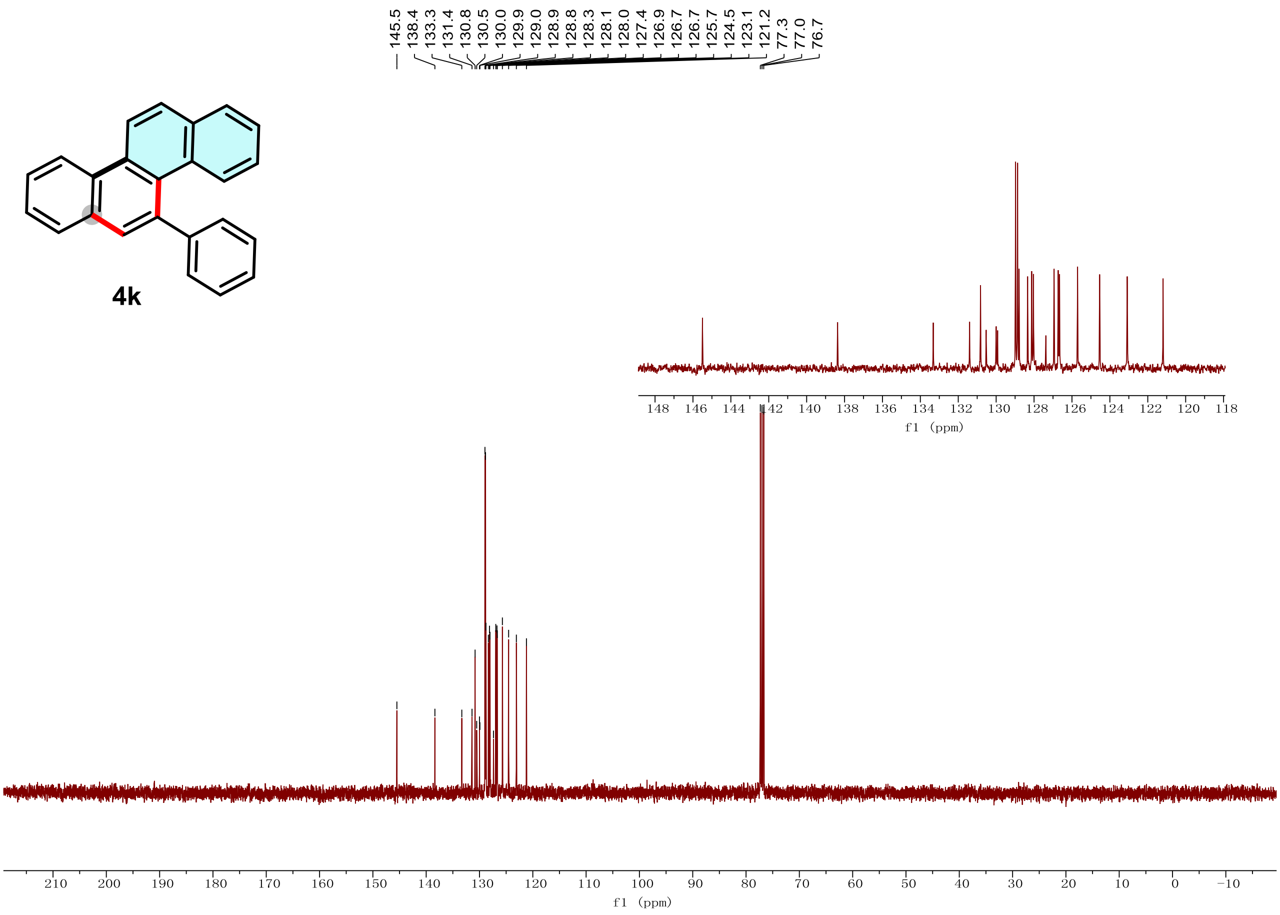

Supplementary Fig. 111. ^{1}H NMR of 4l (600 MHz, CDCl$_3$)

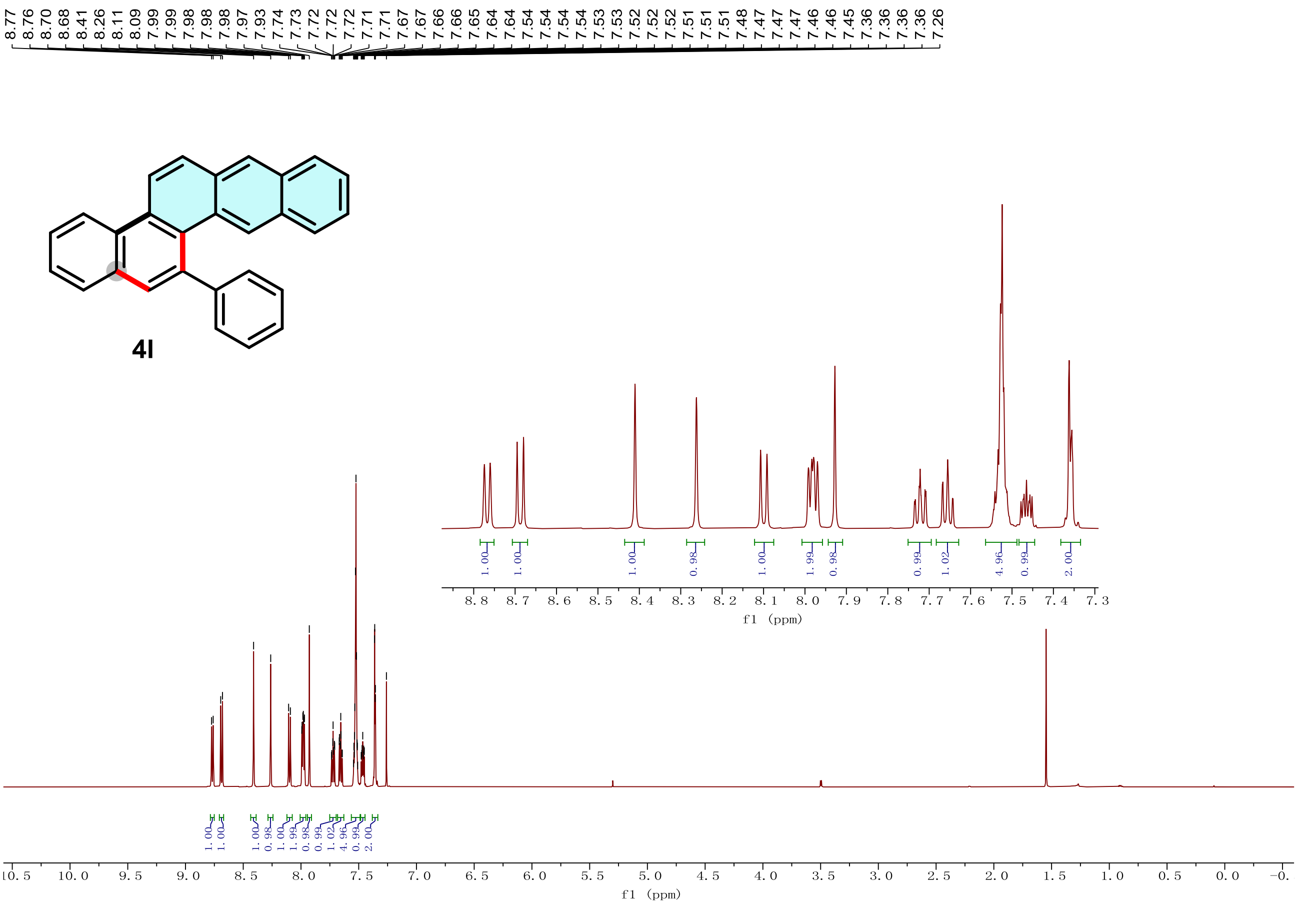

Supplementary Fig. 112. ^{13}C NMR of 4l (151 MHz, CDCl$_3$)

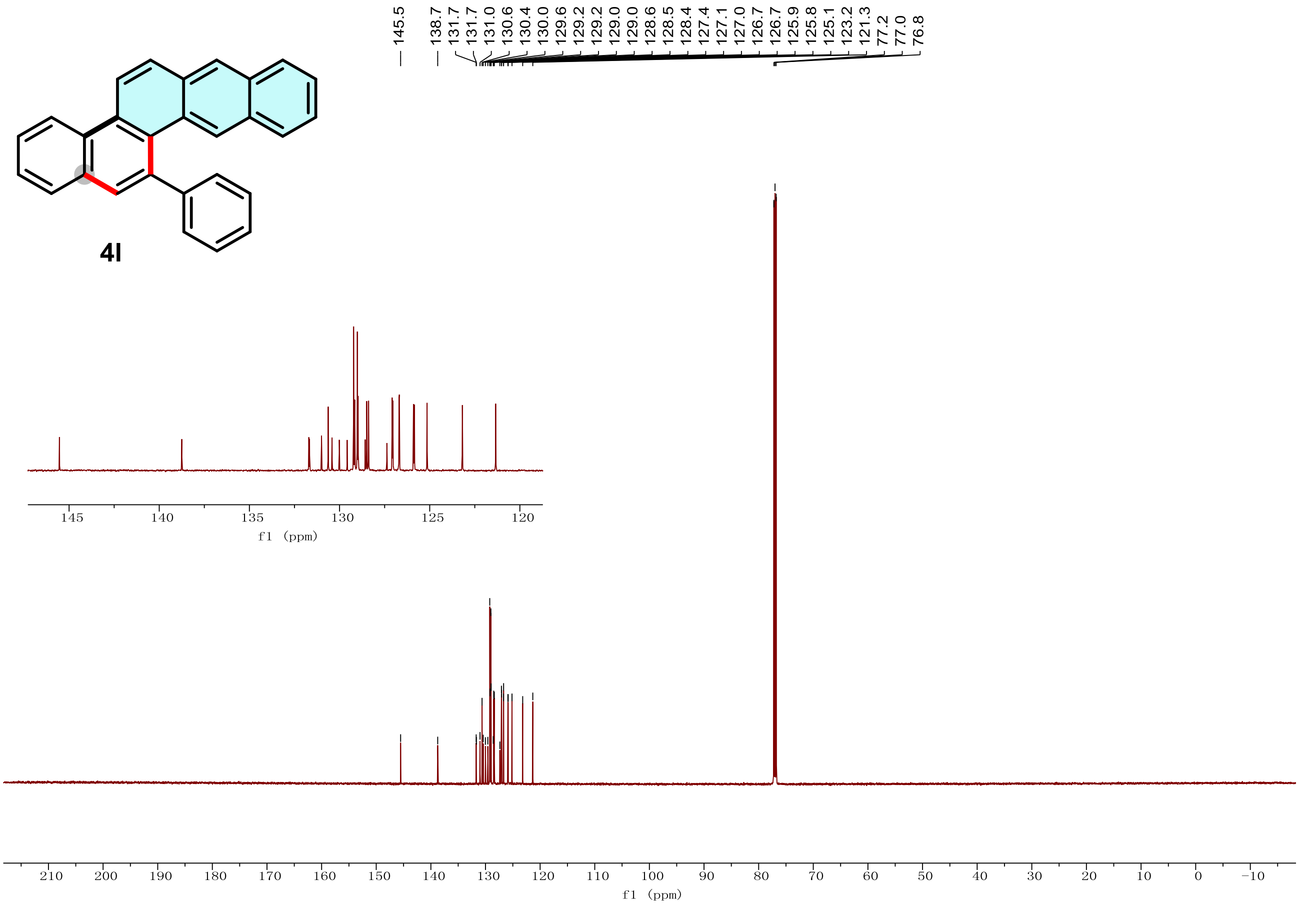

Supplementary Fig. 113. ^{1}H NMR of 4m (400 MHz, $CDCl_3$)

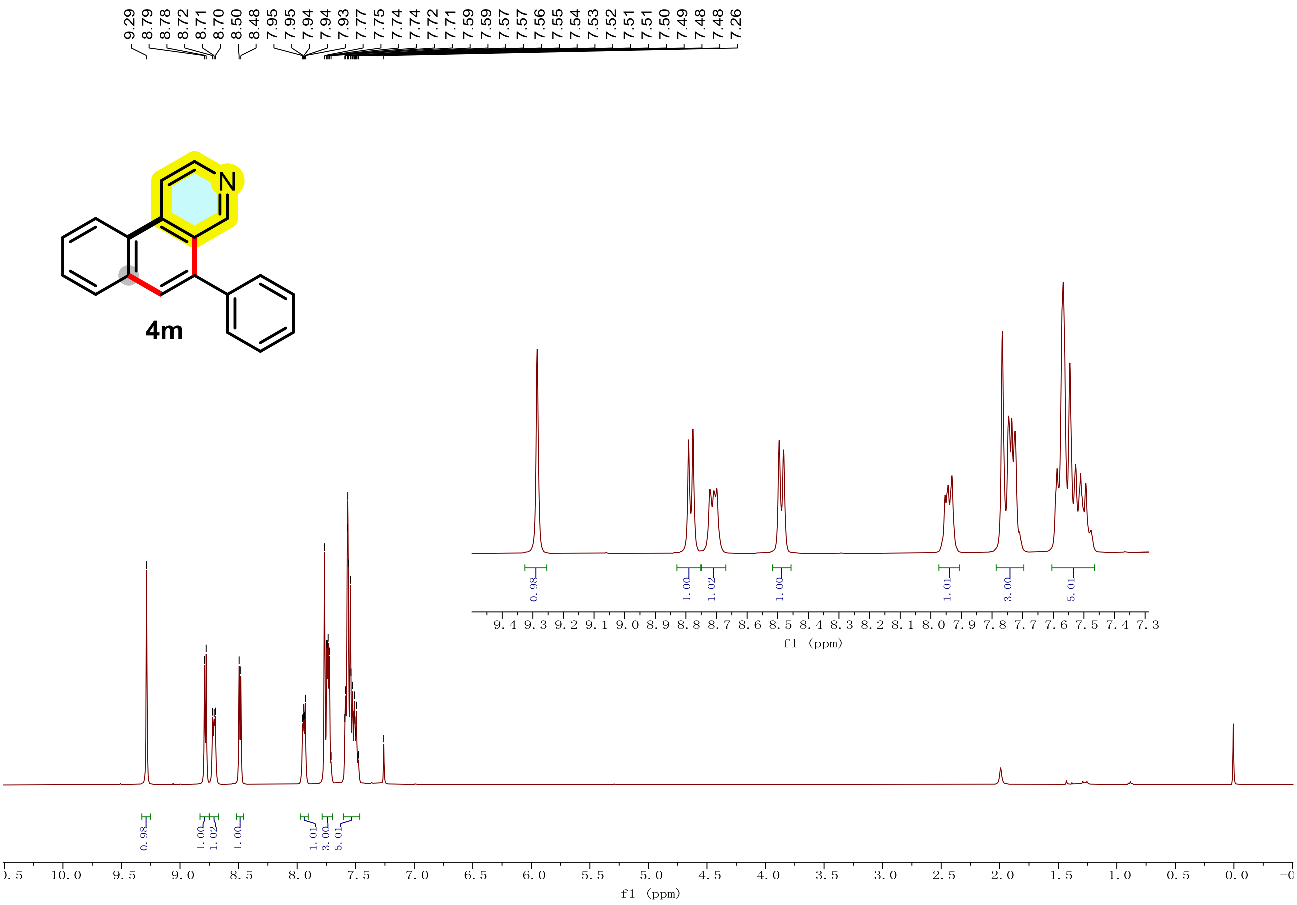

Supplementary Fig. 114. ^{13}C NMR of 4m (101 MHz, $CDCl_3$)

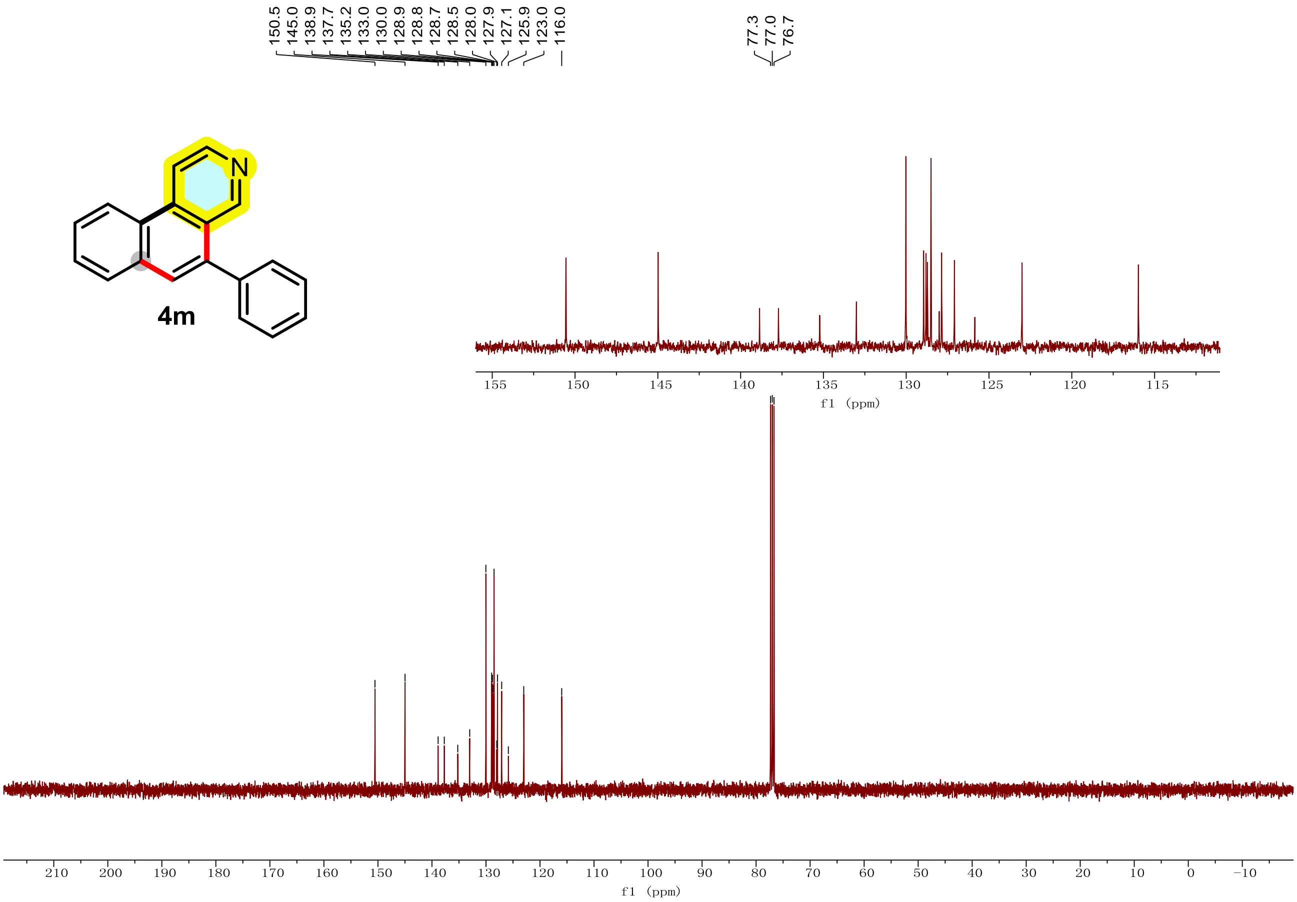

Supplementary Fig. 115. ^{1}H NMR of 4n (400 MHz, $CDCl_3$)

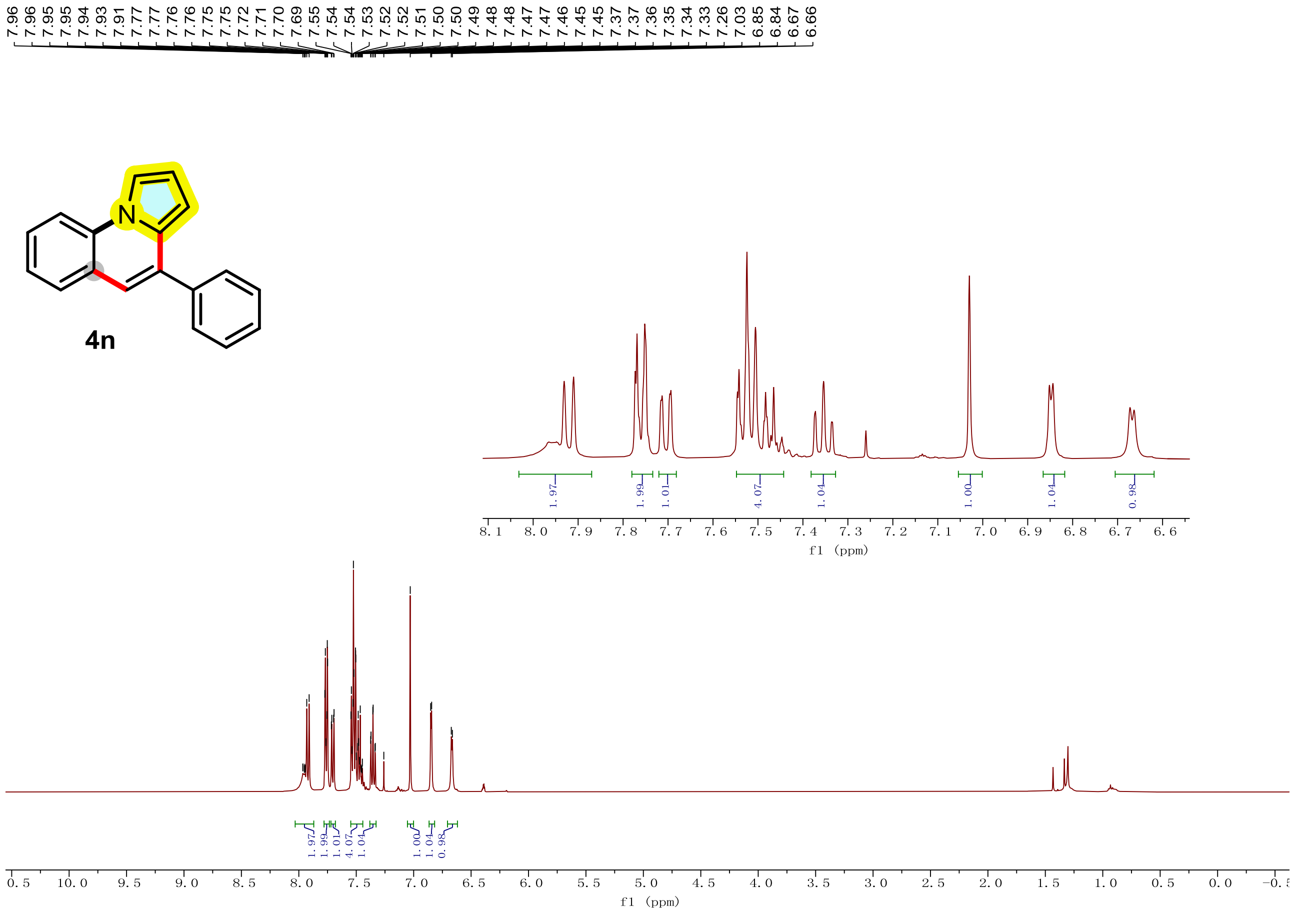

Supplementary Fig. 116. ^{13}C NMR of 4n (101 MHz, $CDCl_3$)

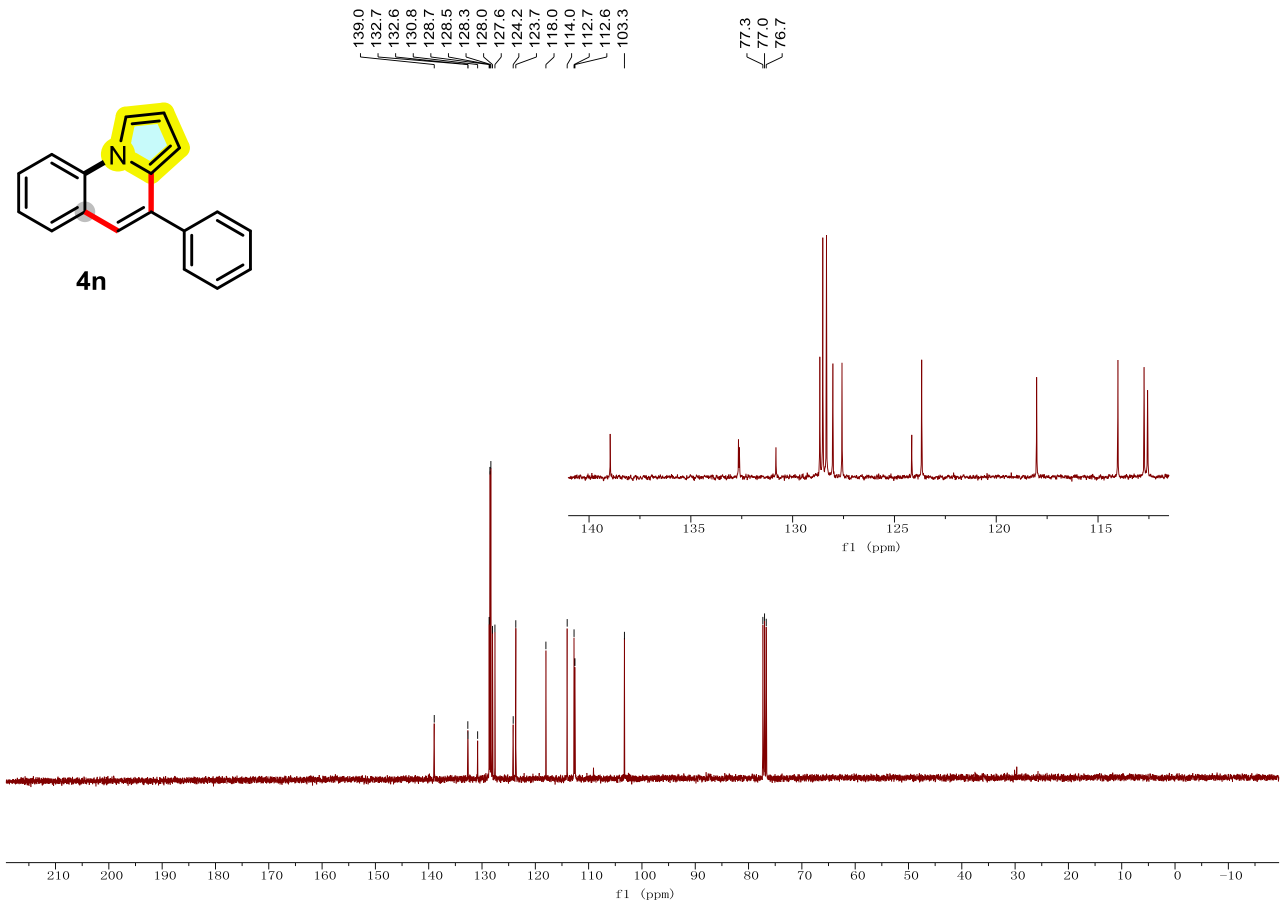

Supplementary Fig. 117. ^{1}H NMR of 4o (400 MHz, $CDCl_3$)

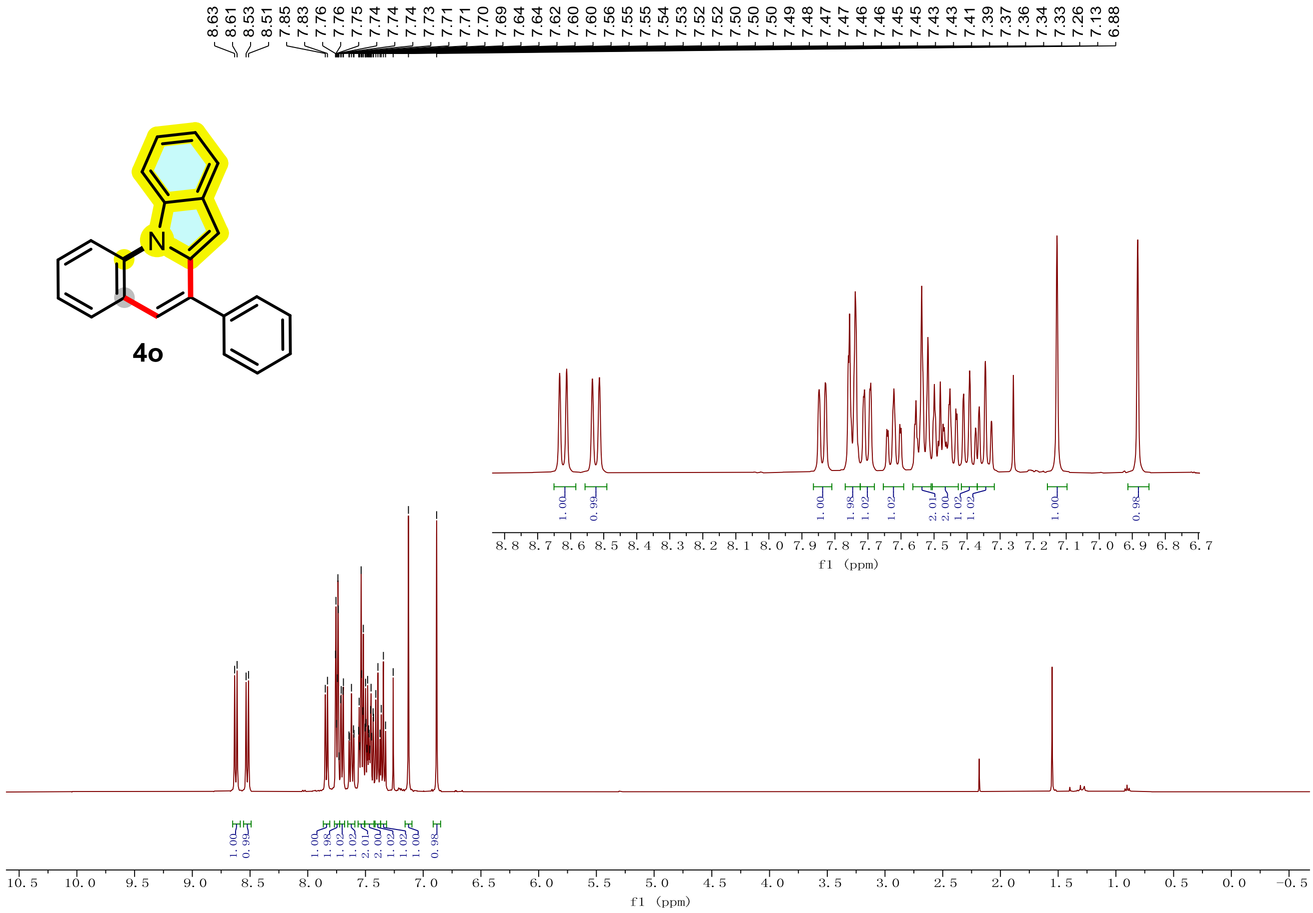

Supplementary Fig. 118. ^{13}C NMR of 4o (101 MHz, $CDCl_3$)

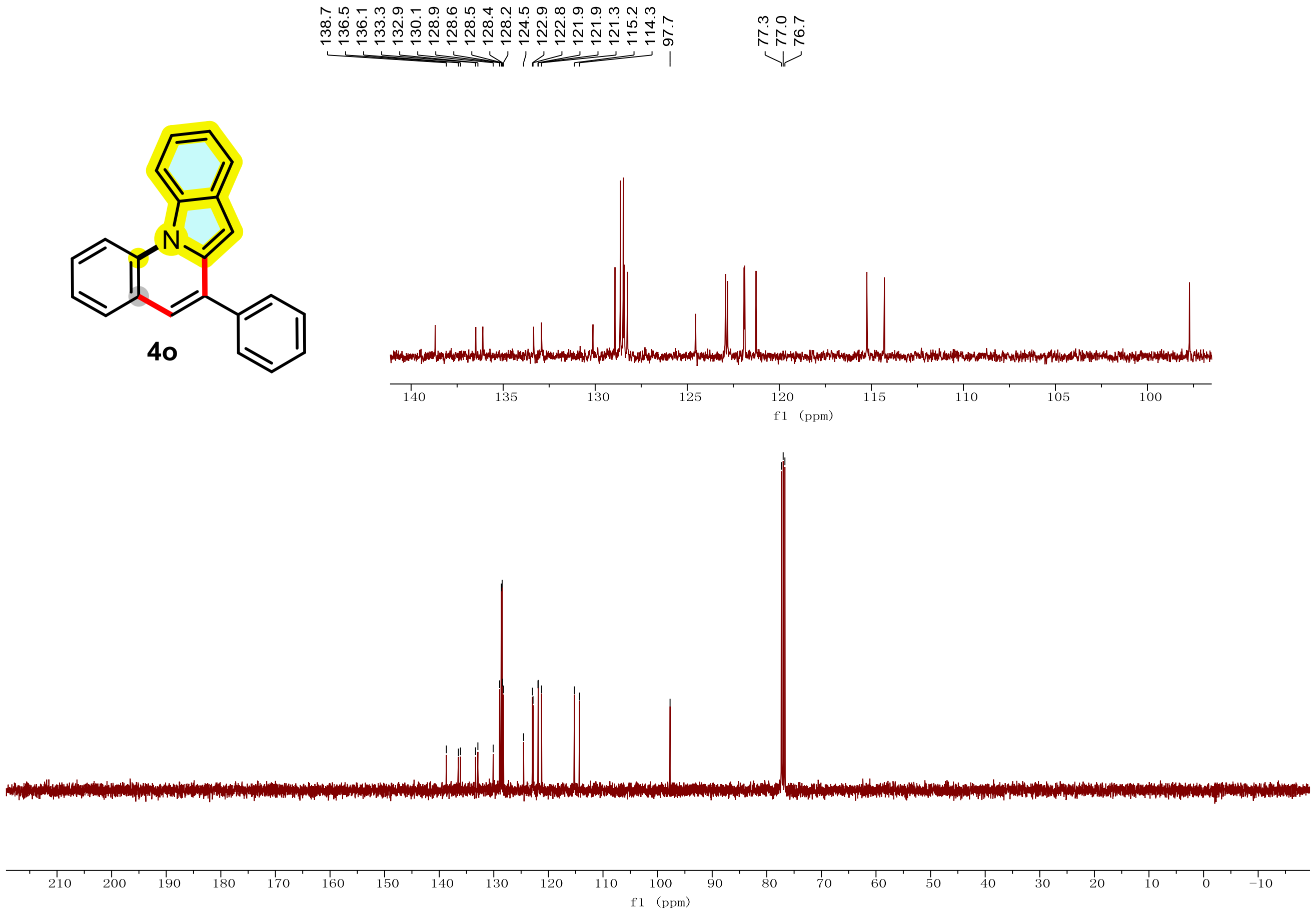

Supplementary Fig. 119. ^{1}H NMR of 4p (400 MHz, $CDCl_3$)

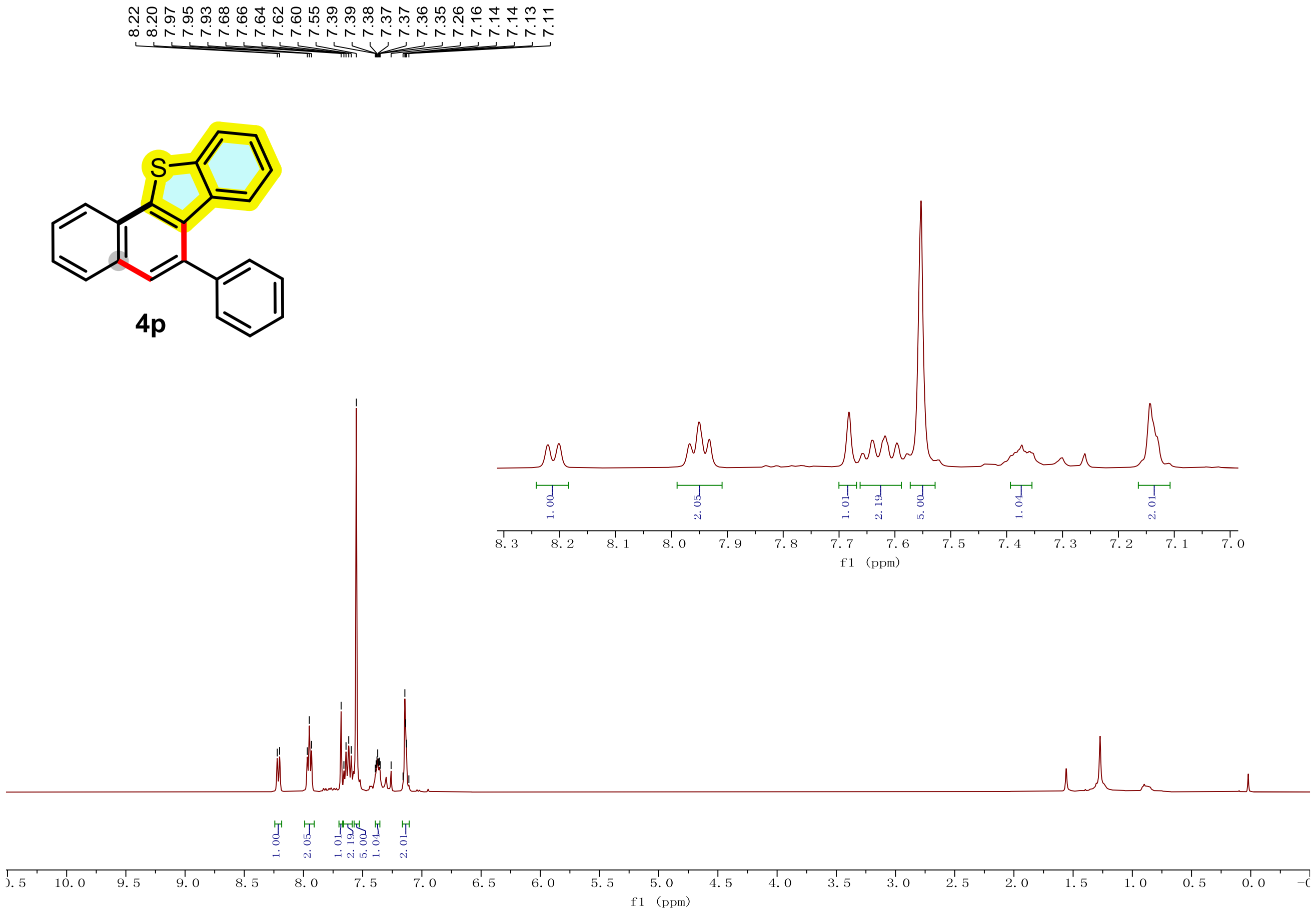

Supplementary Fig. 120. ^{13}C NMR of 4p (101 MHz, $CDCl_3$)

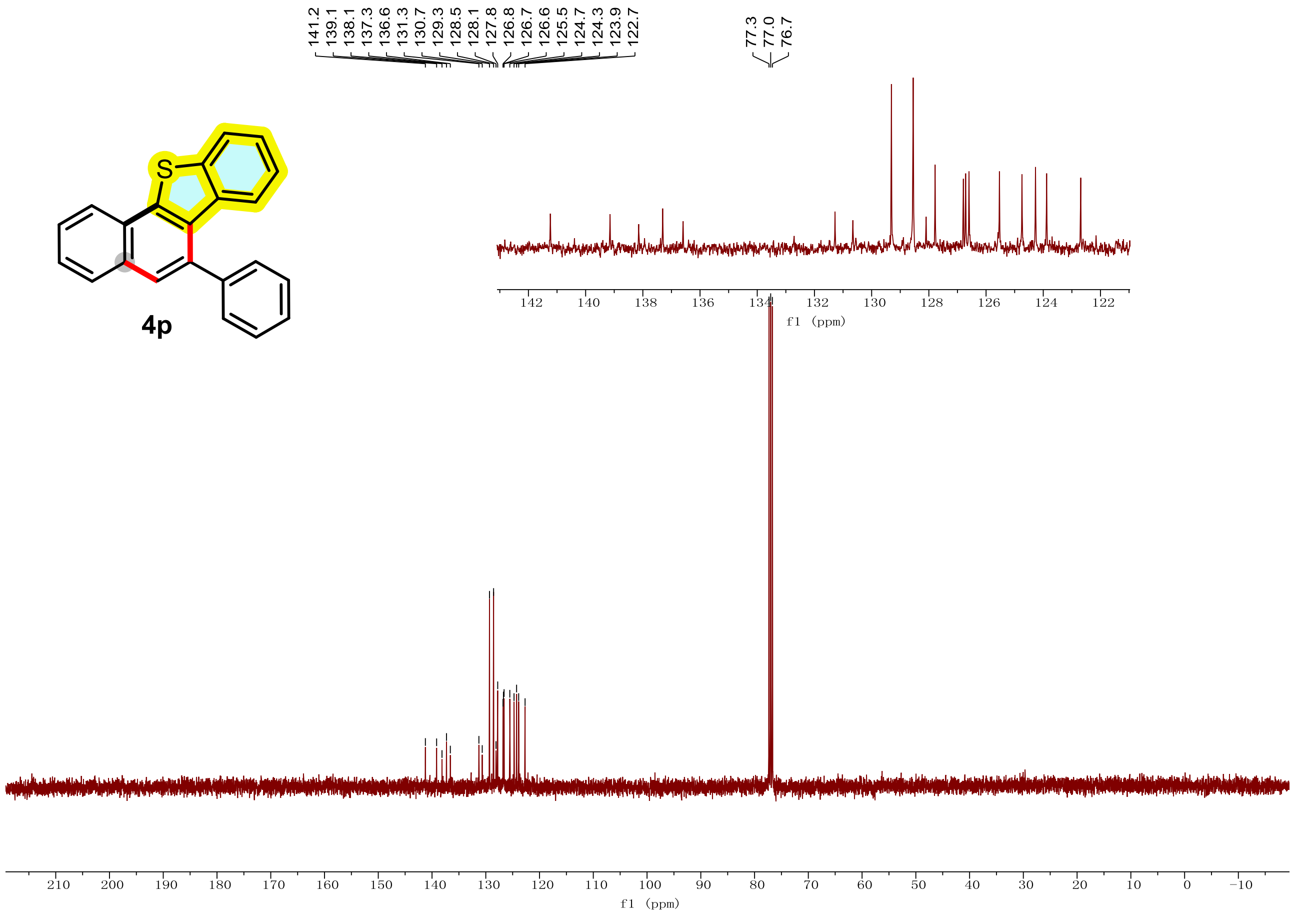

Supplementary Fig. 121. ^{1}H NMR of 4q (400 MHz, $CDCl_3$)

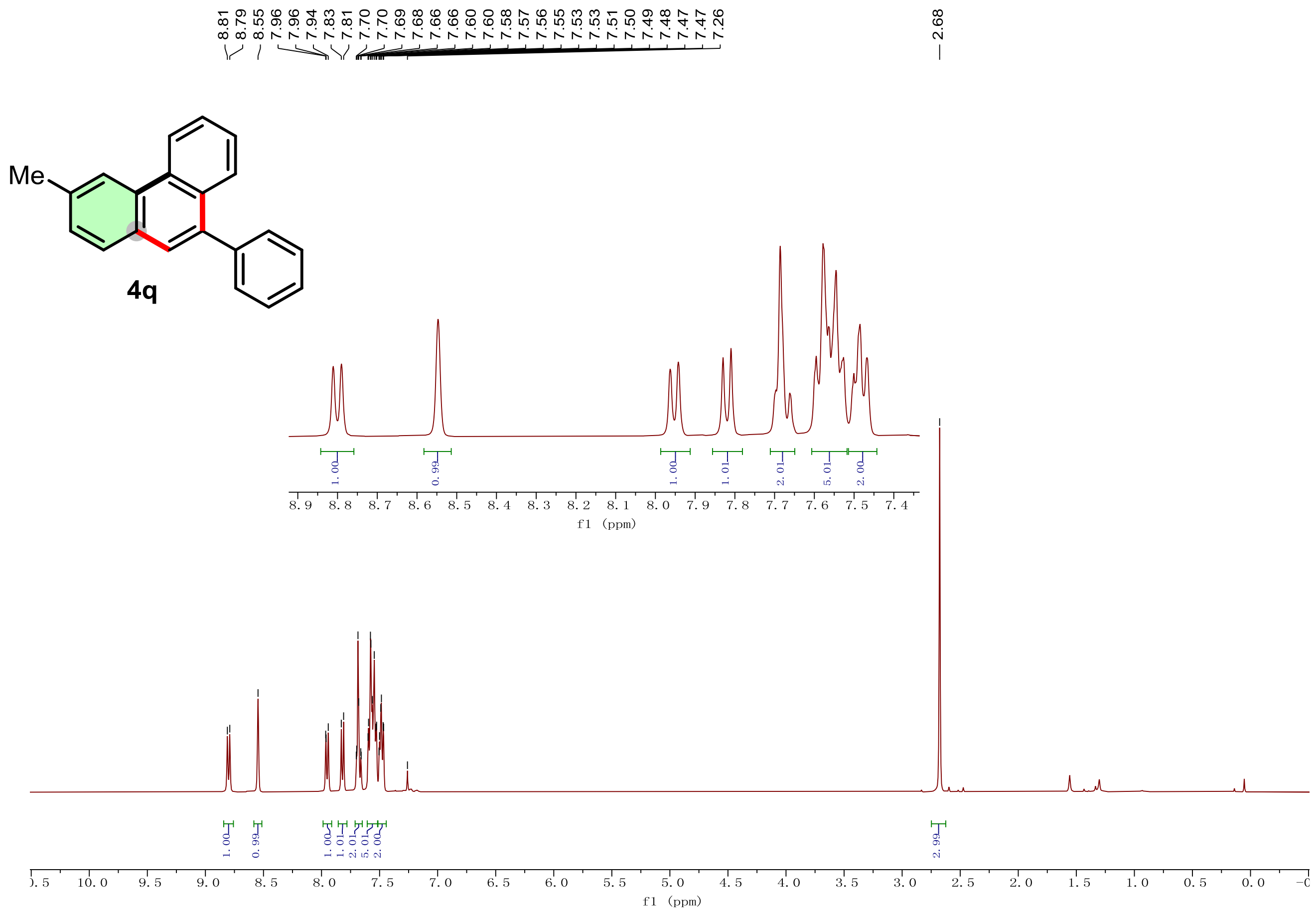

Supplementary Fig. 122. ^{13}C NMR of 4q (101 MHz, $CDCl_3$)

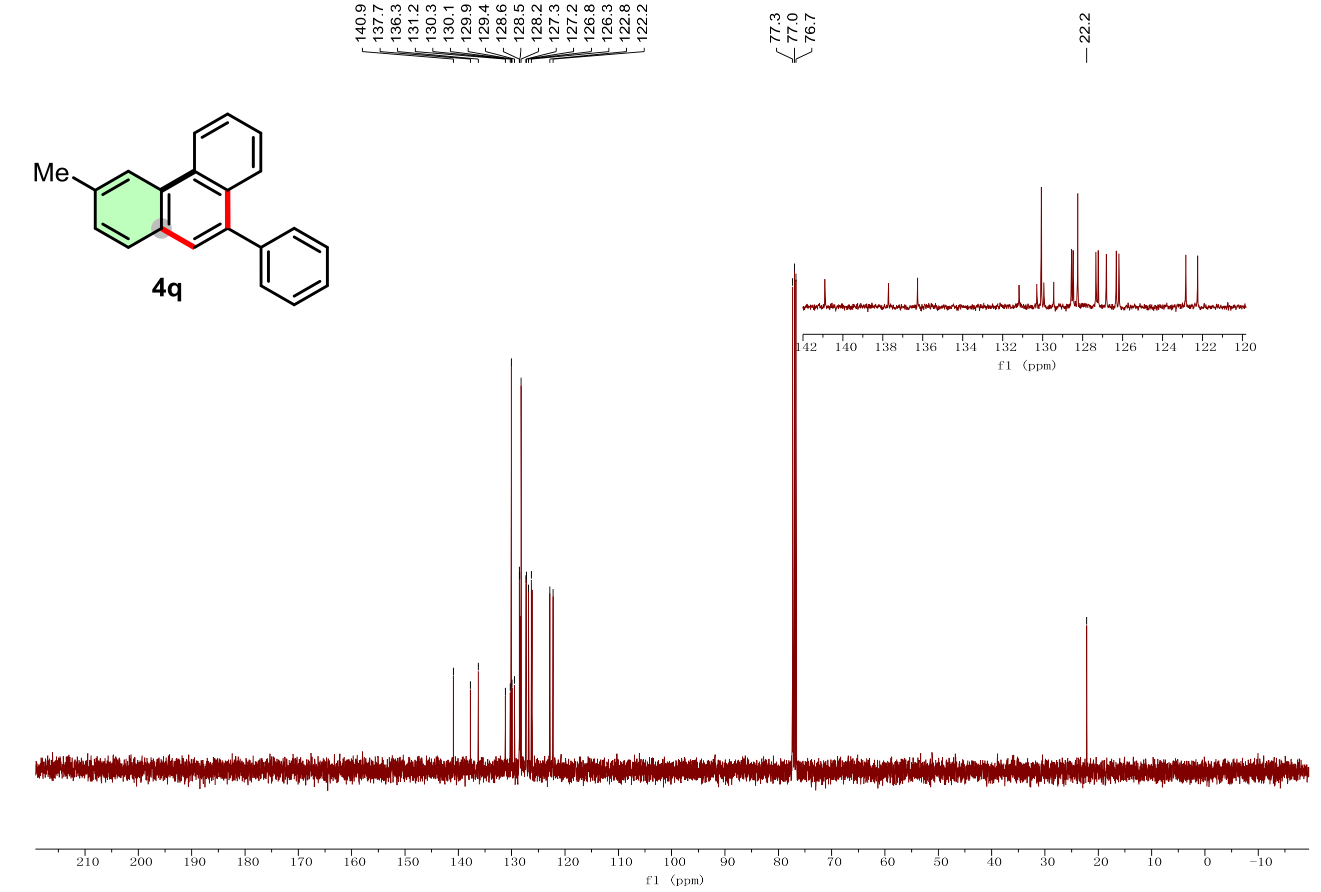

Supplementary Fig. 123. ^{1}H NMR of 4r (400 MHz, $CDCl_3$)

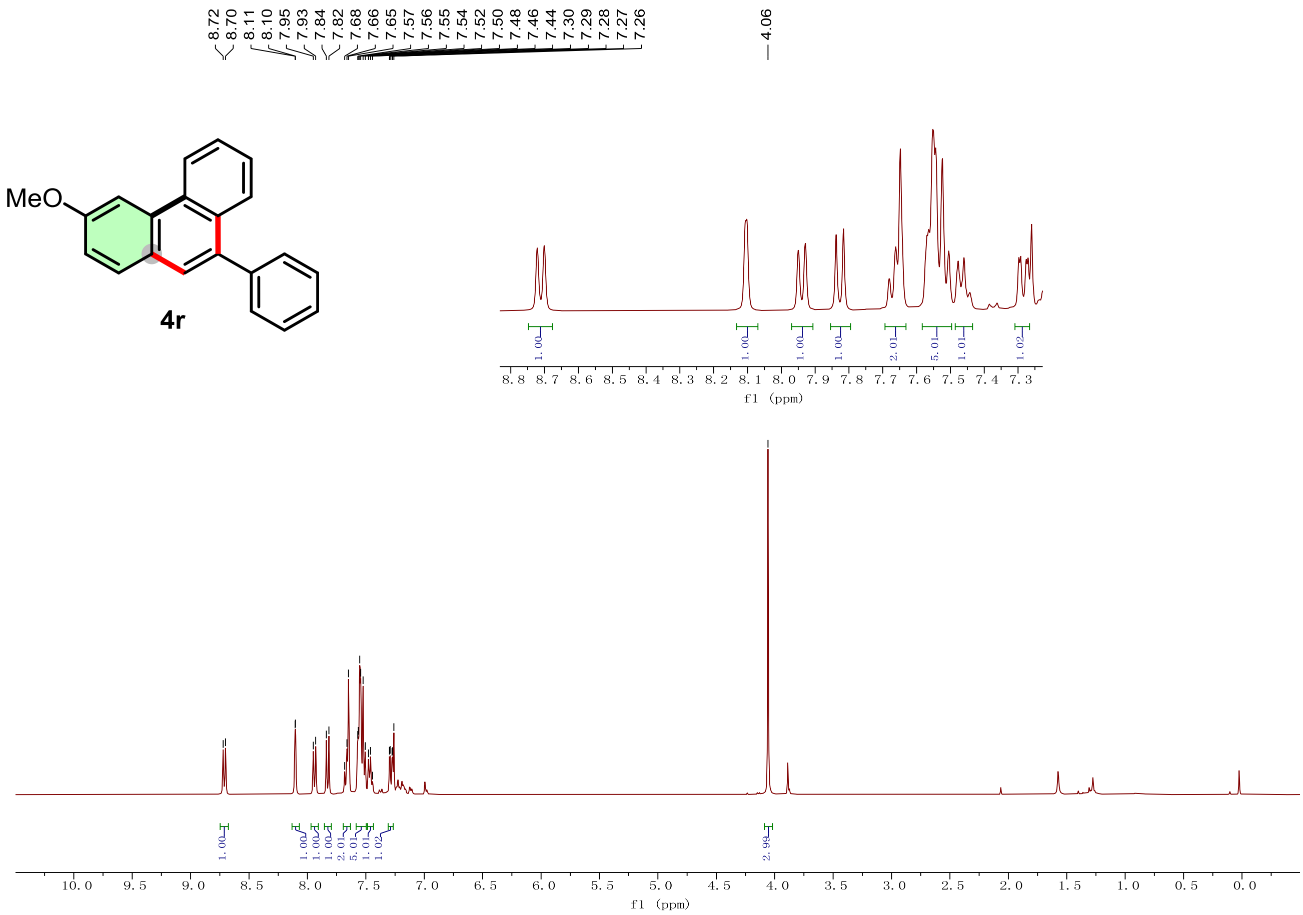

Supplementary Fig. 124. ^{13}C NMR of 4r (101 MHz, $CDCl_3$)

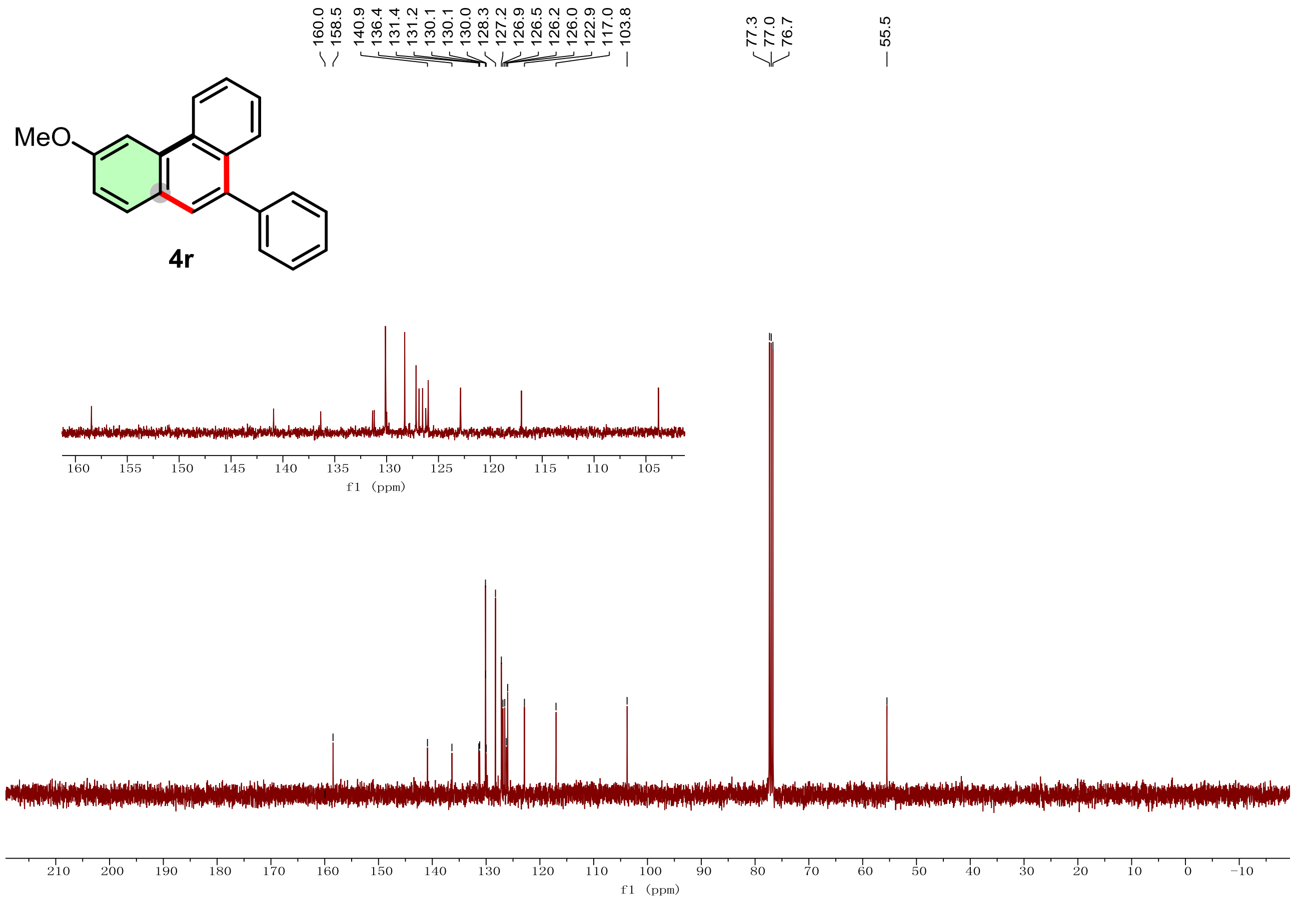

Supplementary Fig. 125. ^{1}H NMR of 4s (400 MHz, $CDCl_3$)

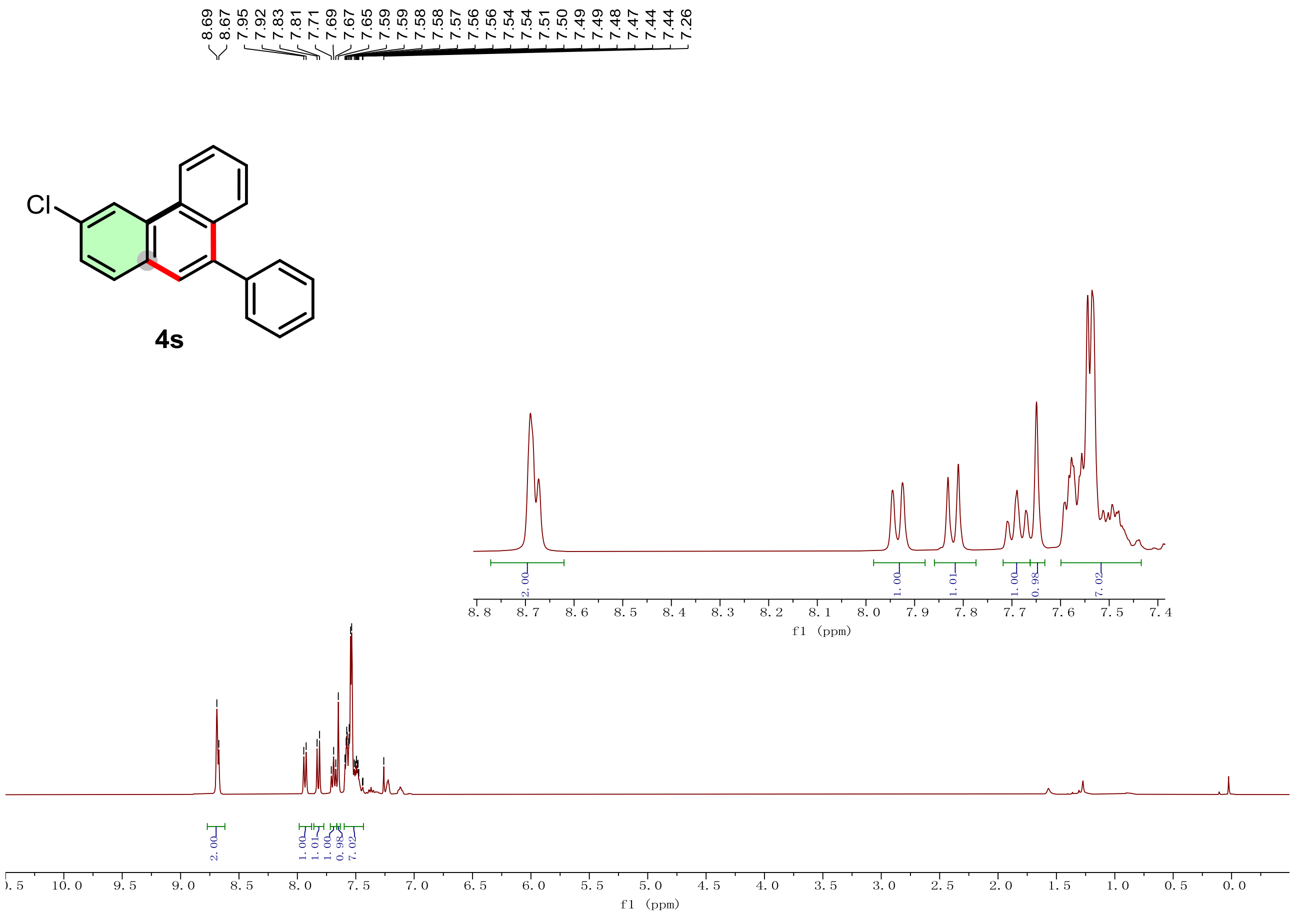

Supplementary Fig. 126. ^{13}C NMR of 4s (101 MHz, $CDCl_3$)

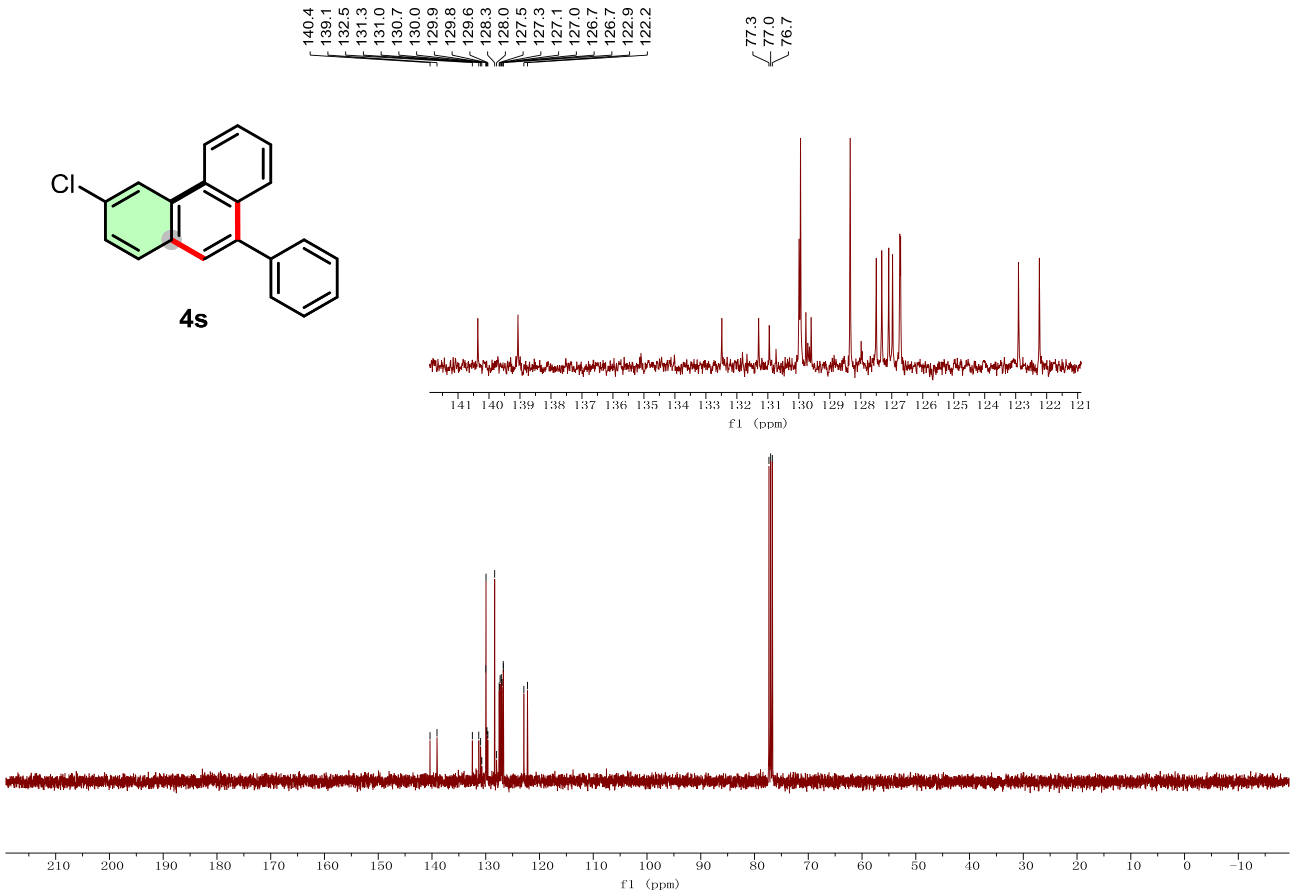

Supplementary Fig. 127. ^{1}H NMR of 4t (400 MHz, $CDCl_3$)

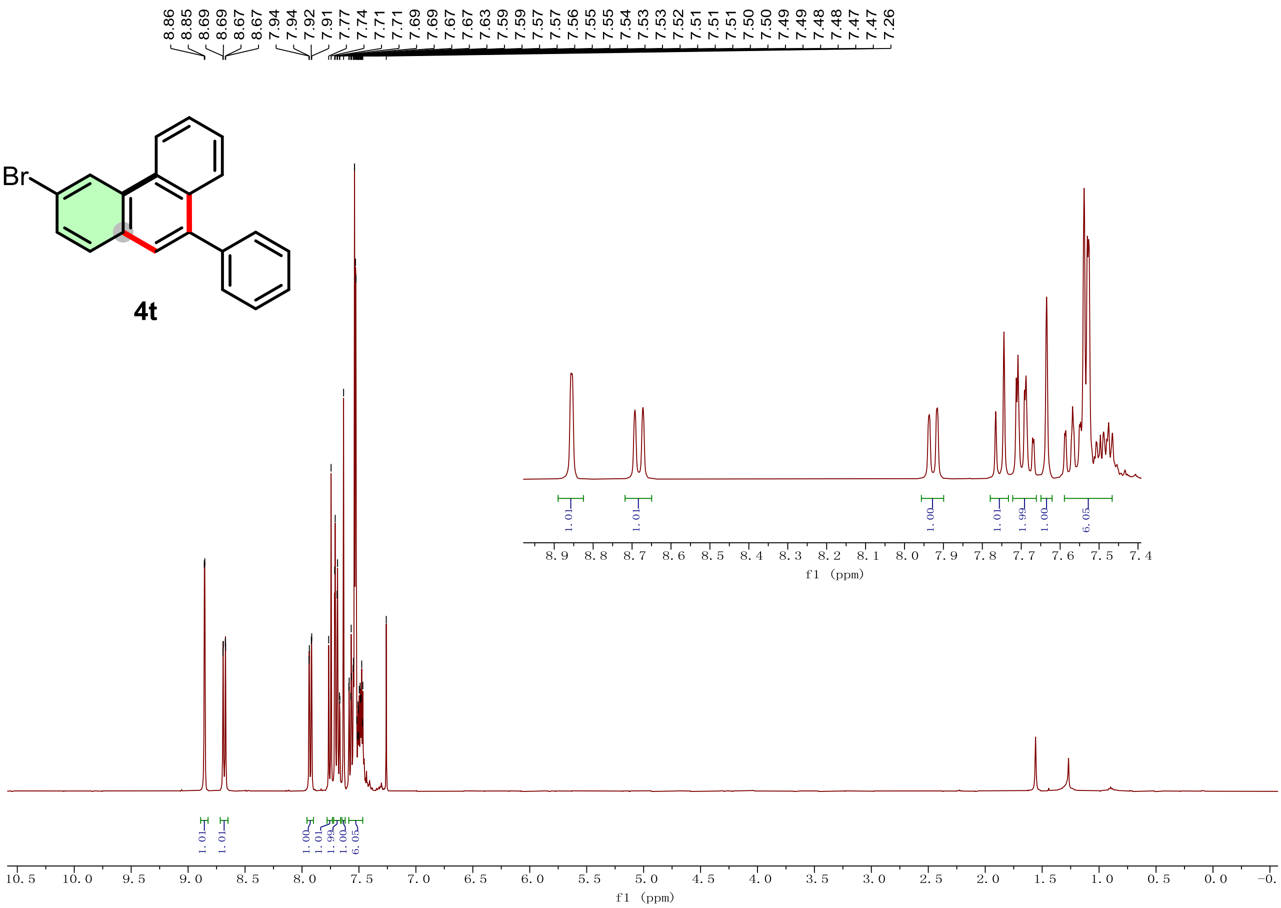

Supplementary Fig. 128. ^{13}C NMR of 4t (101 MHz, $CDCl_3$)

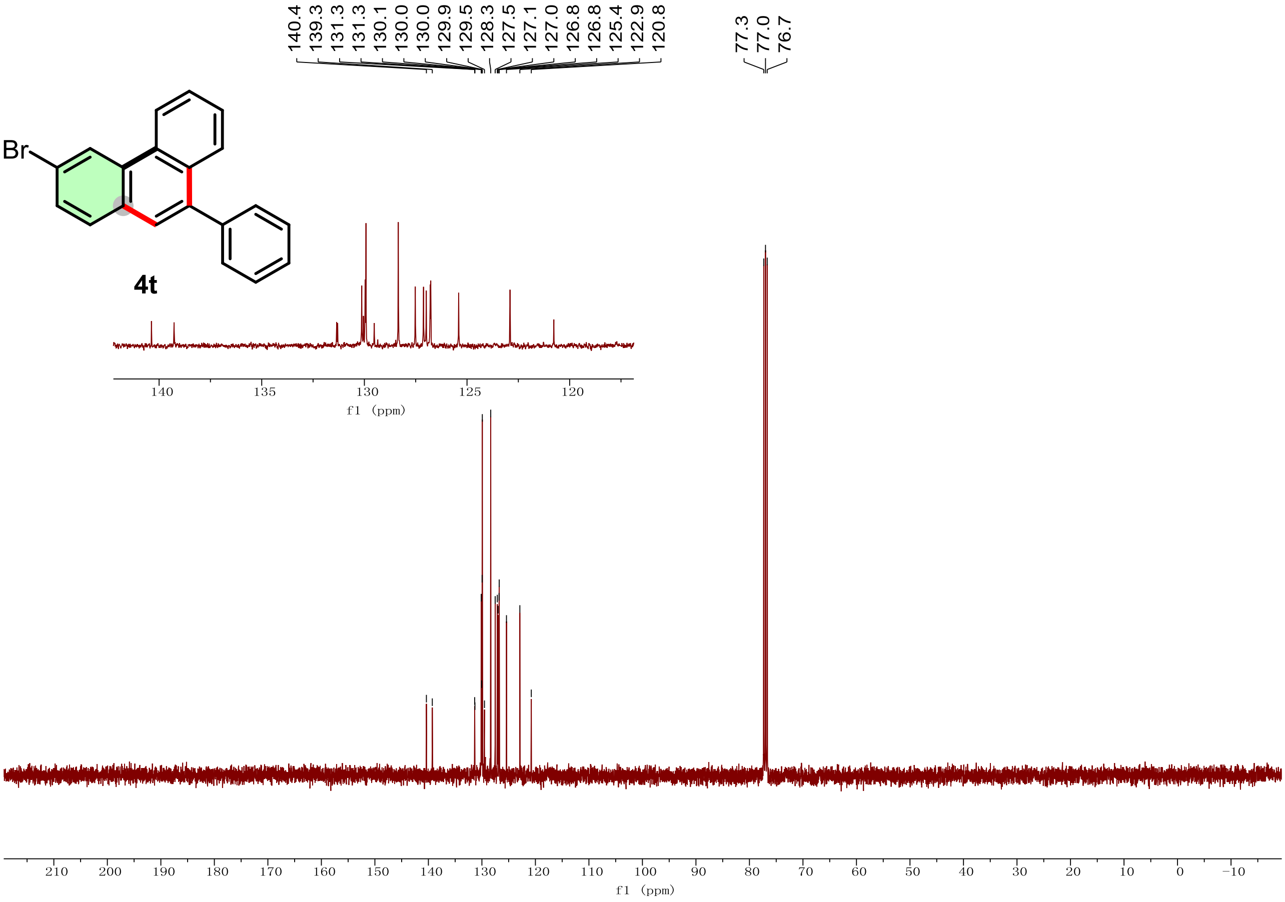

Supplementary Fig. 129. ^{1}H NMR of 4u (400 MHz, $CDCl_3$)

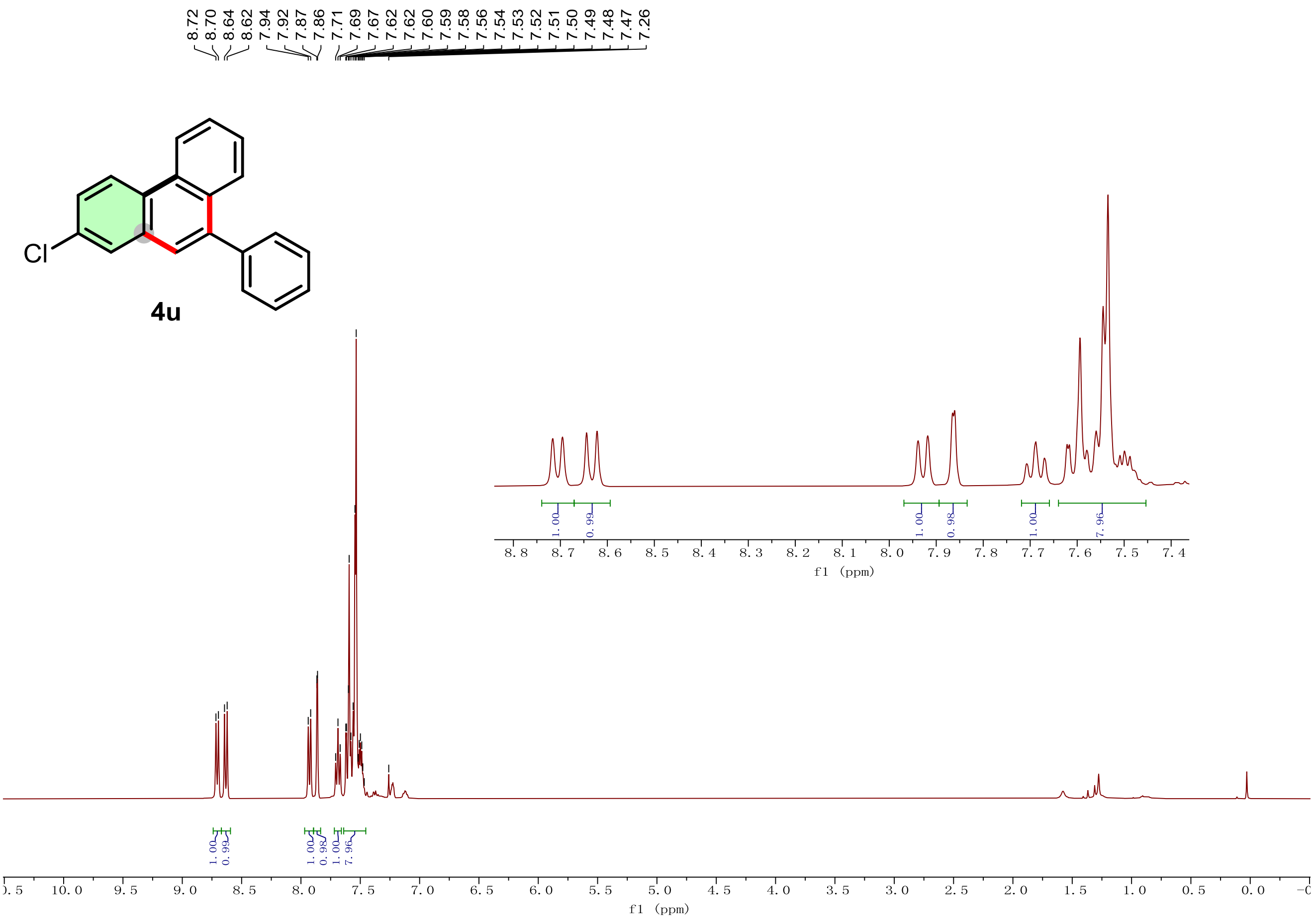

Supplementary Fig. 130. ^{13}C NMR of 4u (101 MHz, $CDCl_3$)

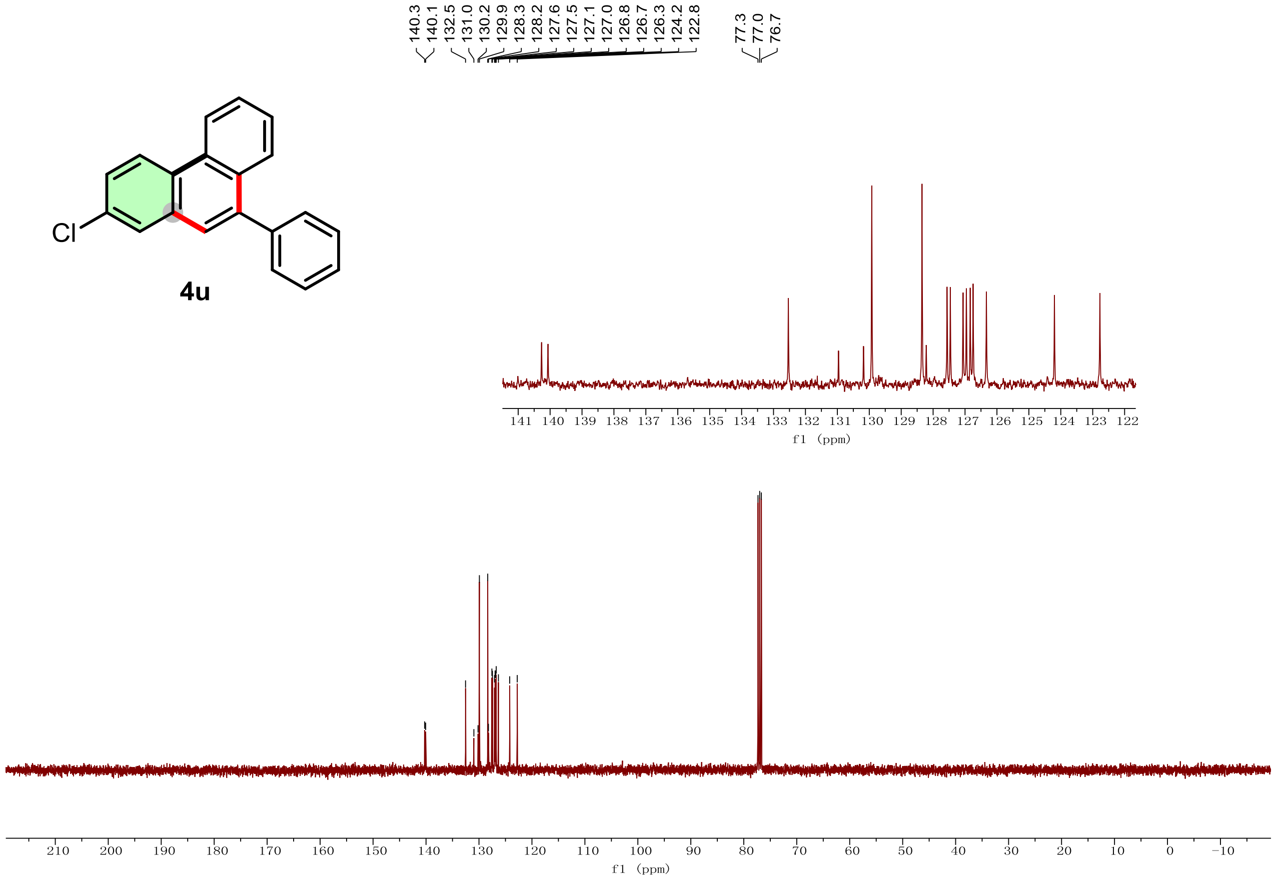

Supplementary Fig. 131. ^{1}H NMR of 4v (400 MHz, $CDCl_3$)

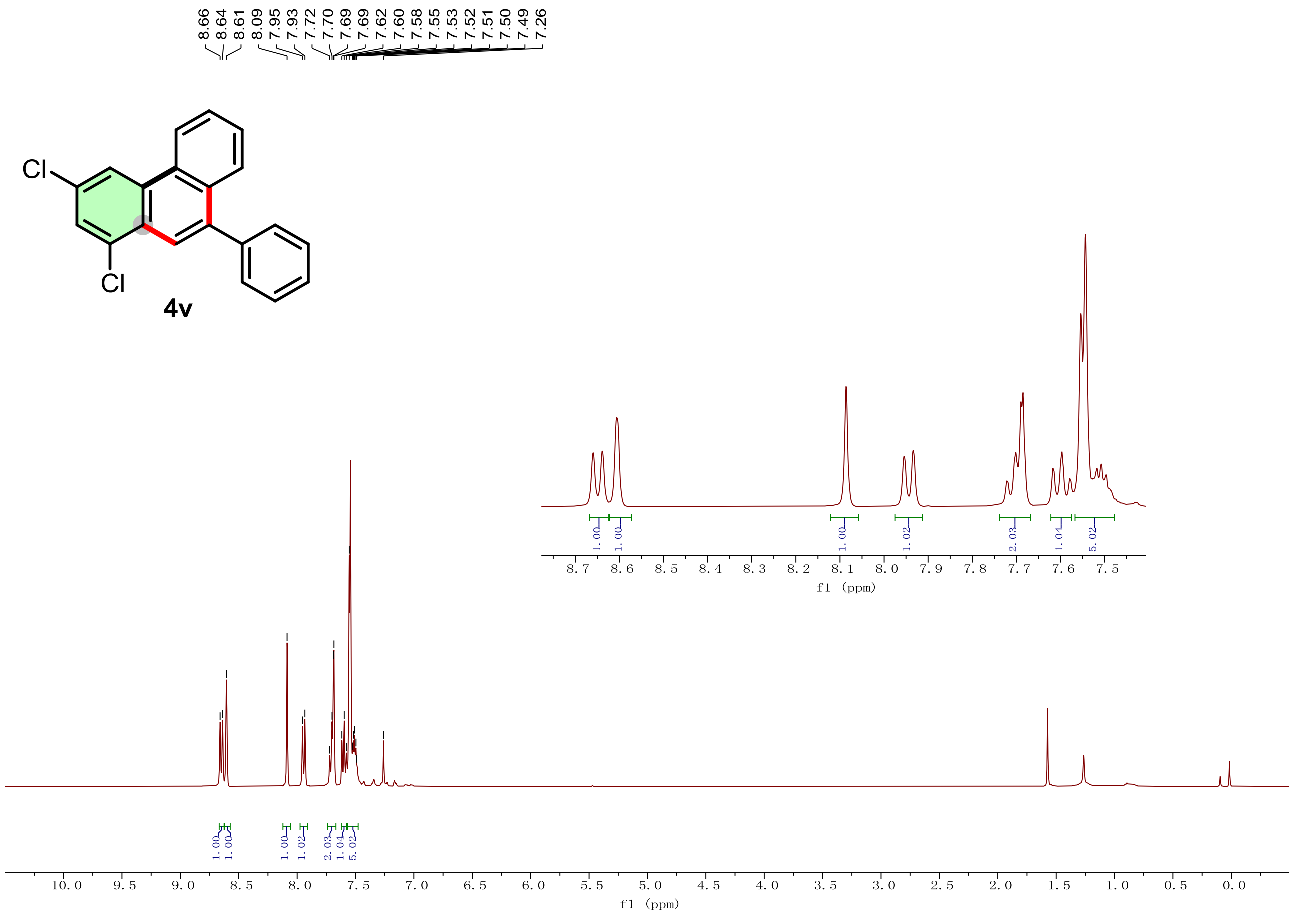

Supplementary Fig. 132. ^{13}C NMR of 4v (101 MHz, $CDCl_3$)

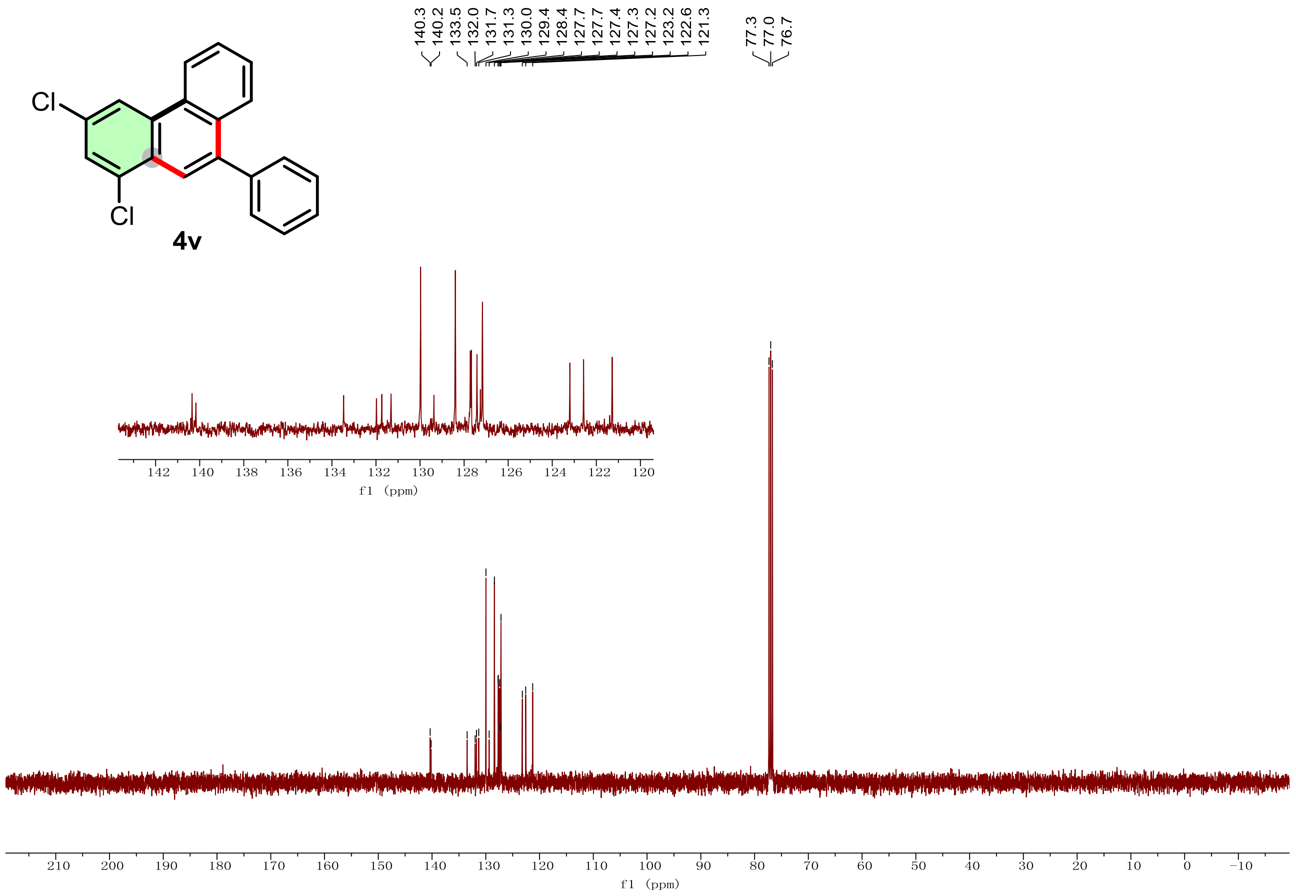

Supplementary Fig. 133. ^{1}H NMR of 4w (400 MHz, $CDCl_3$)

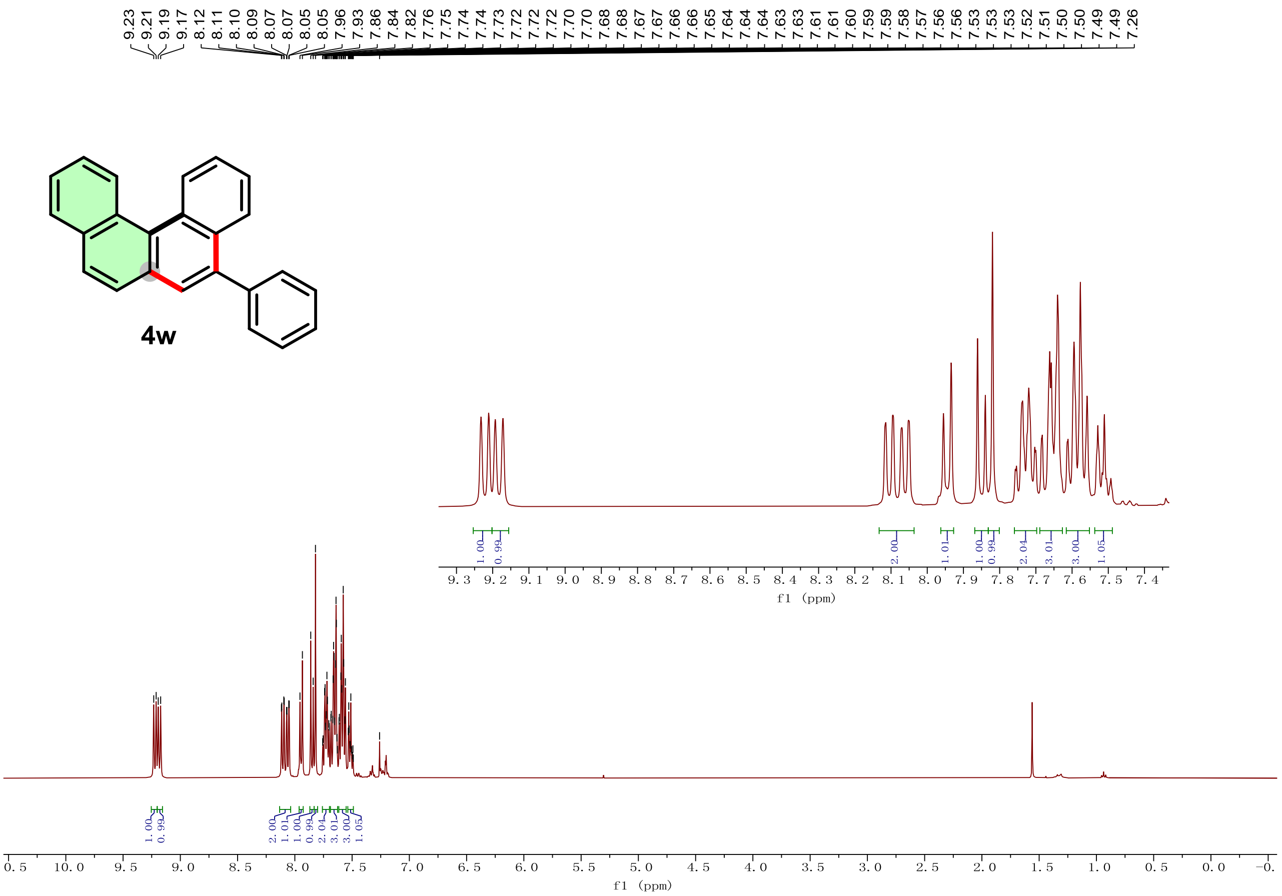

Supplementary Fig. 134. ^{13}C NMR of 4w (101 MHz, $CDCl_3$)

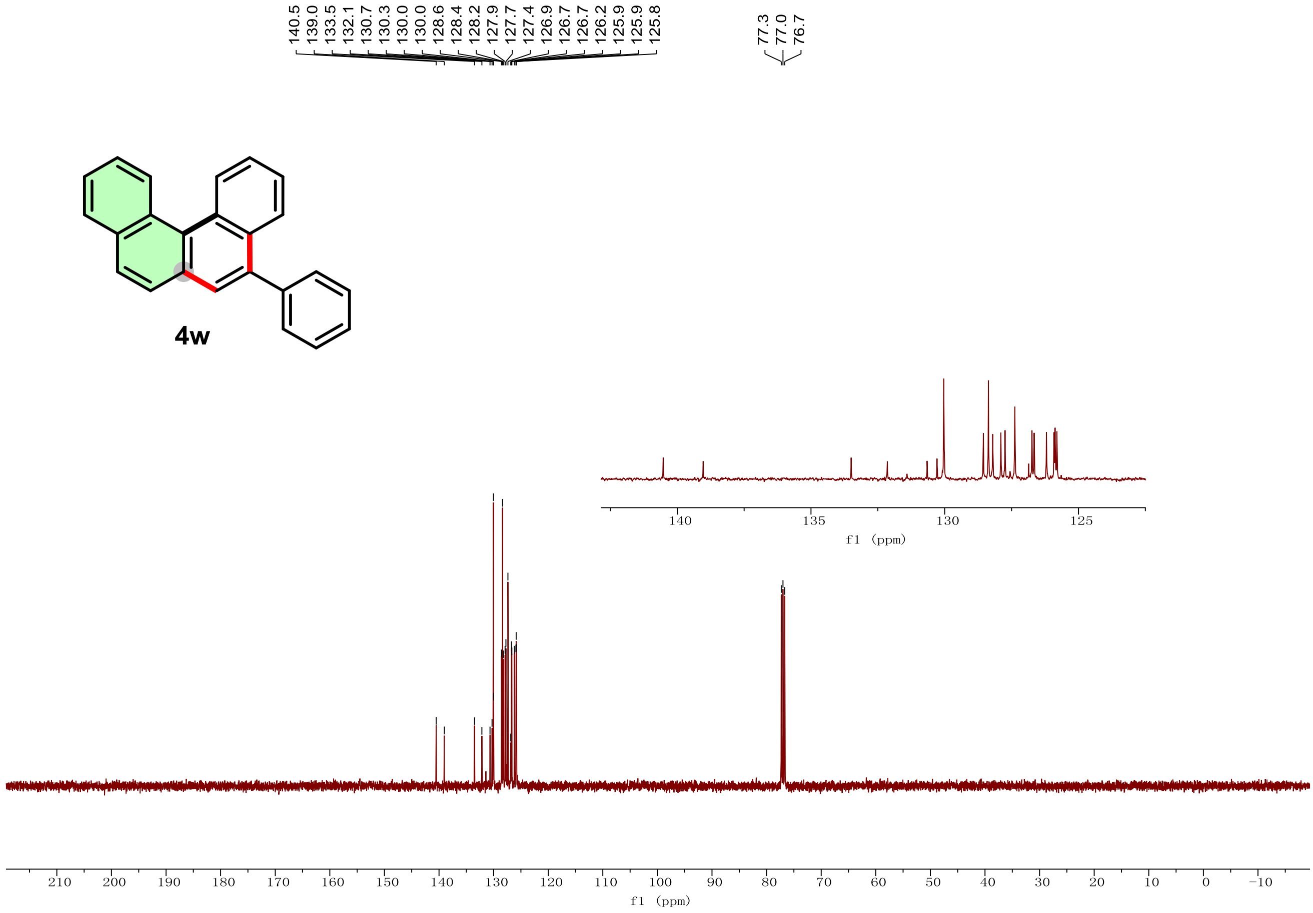

Supplementary Fig. 135. ^{1}H NMR of 4x (400 MHz, $CDCl_3$)

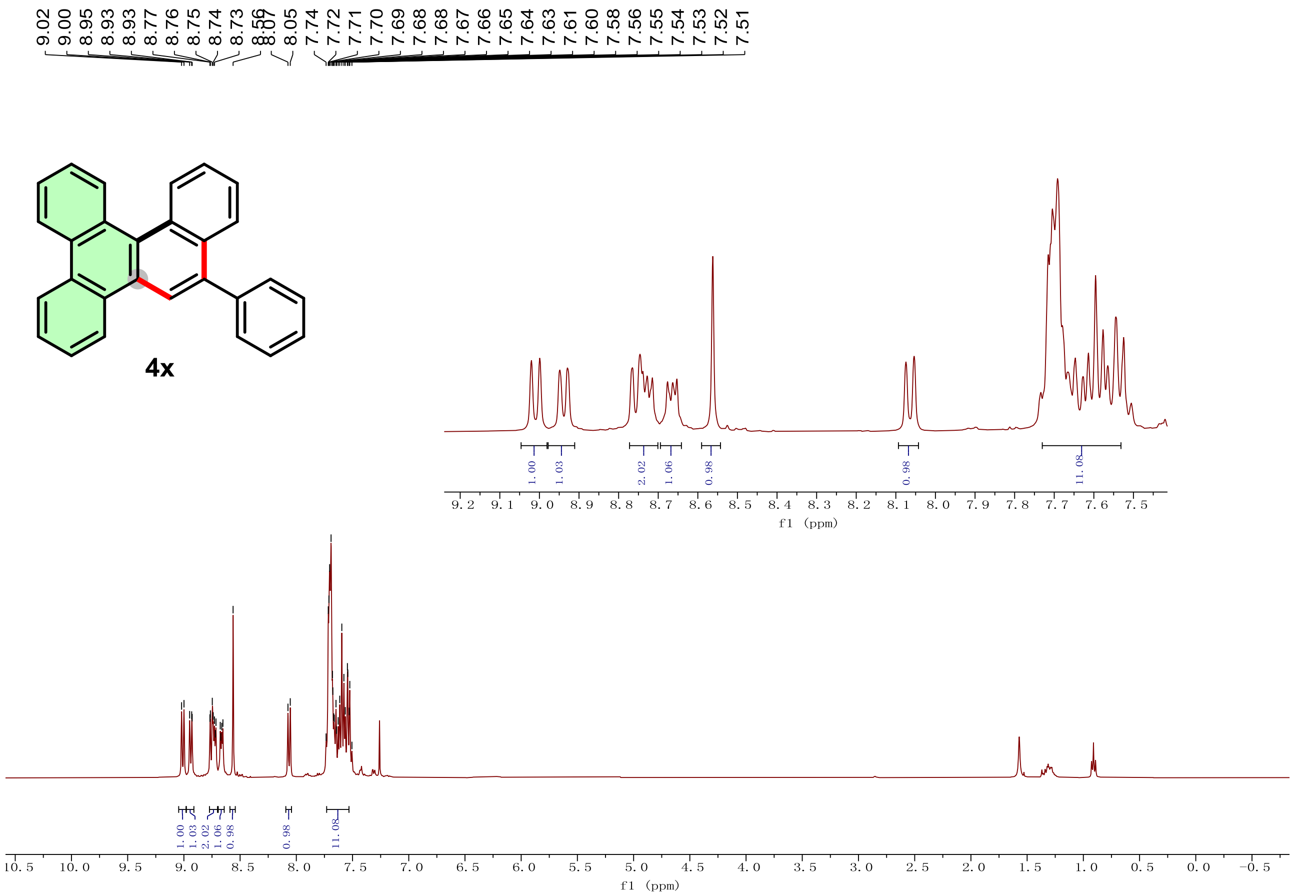

Supplementary Fig. 136. ^{13}C NMR of 4x (101 MHz, $CDCl_3$)

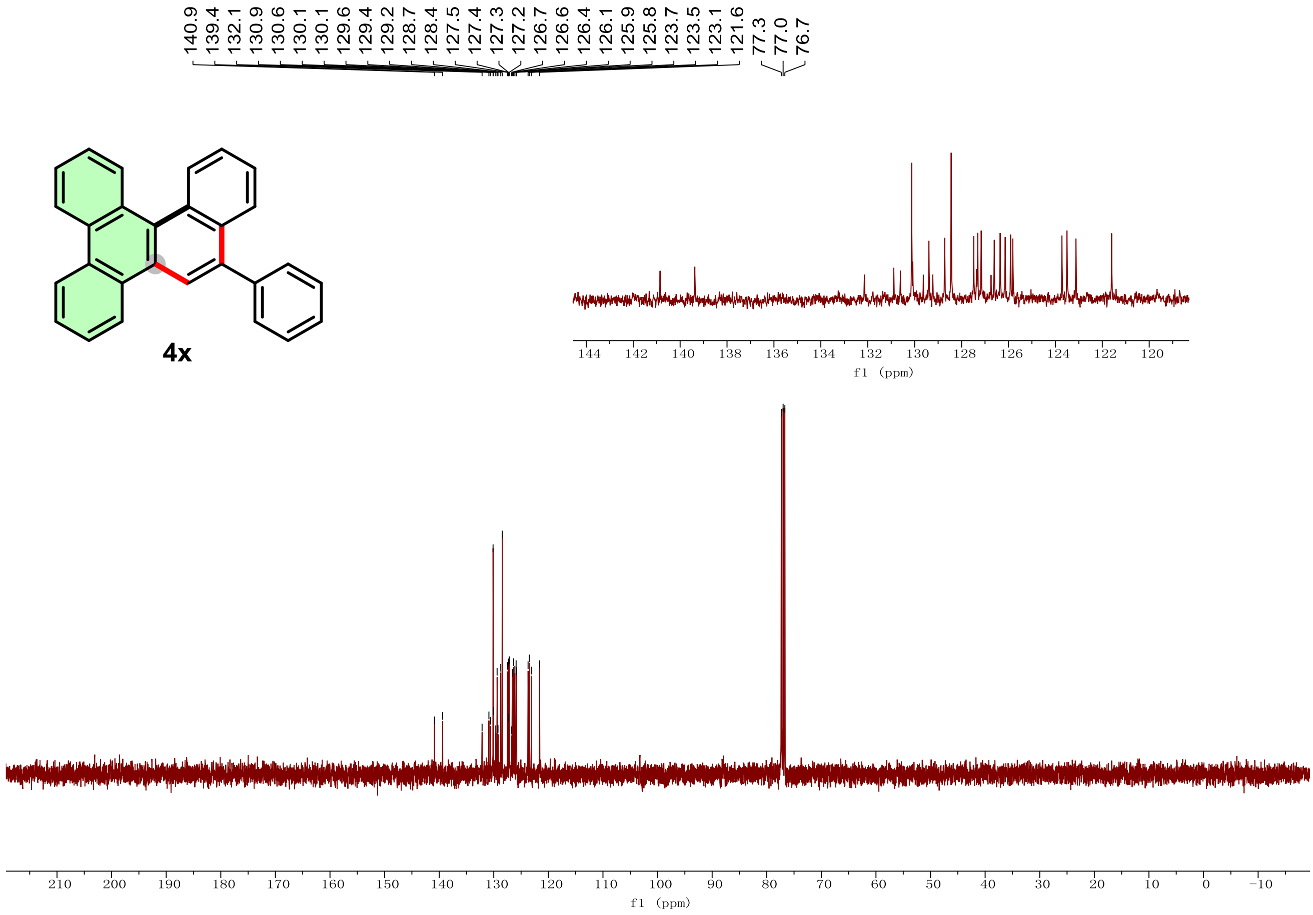

Supplementary Fig. 137. ^{1}H NMR of 4y (400 MHz, $CDCl_3$)

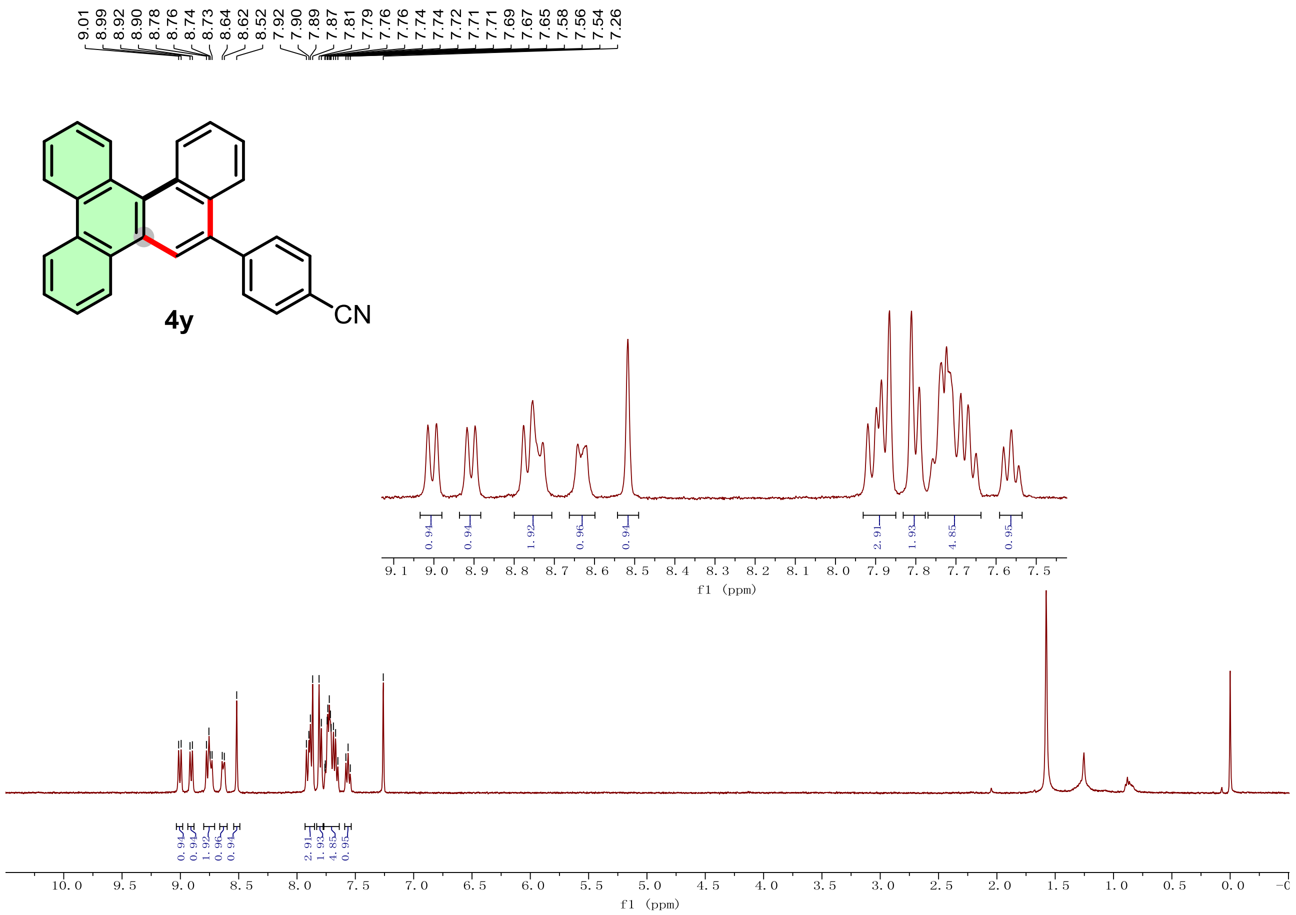

Supplementary Fig. 138. ^{13}C NMR of 4y (101 MHz, $CDCl_3$)

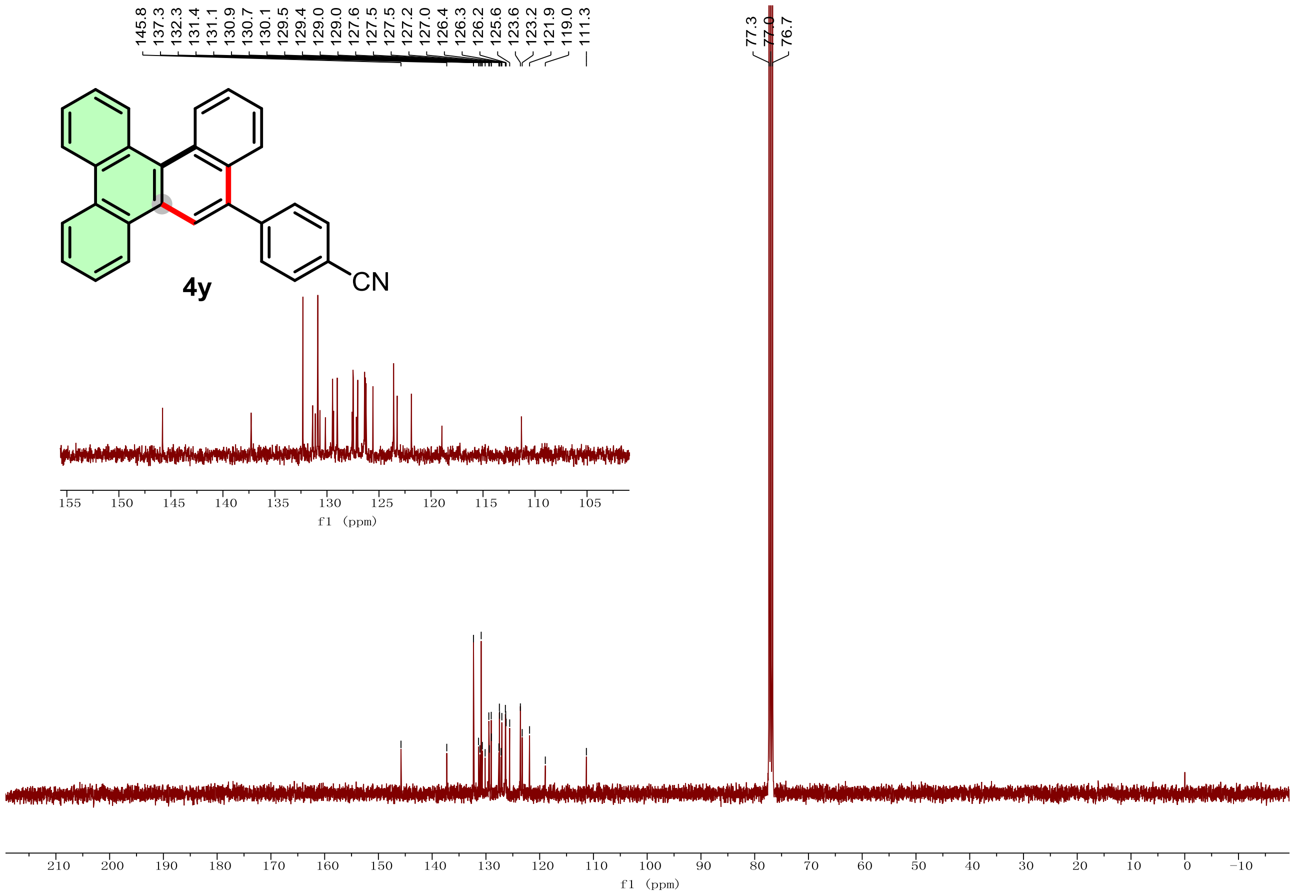

Supplementary Fig. 139. ¹H NMR of 4z (400 MHz, $CDCl_3$)

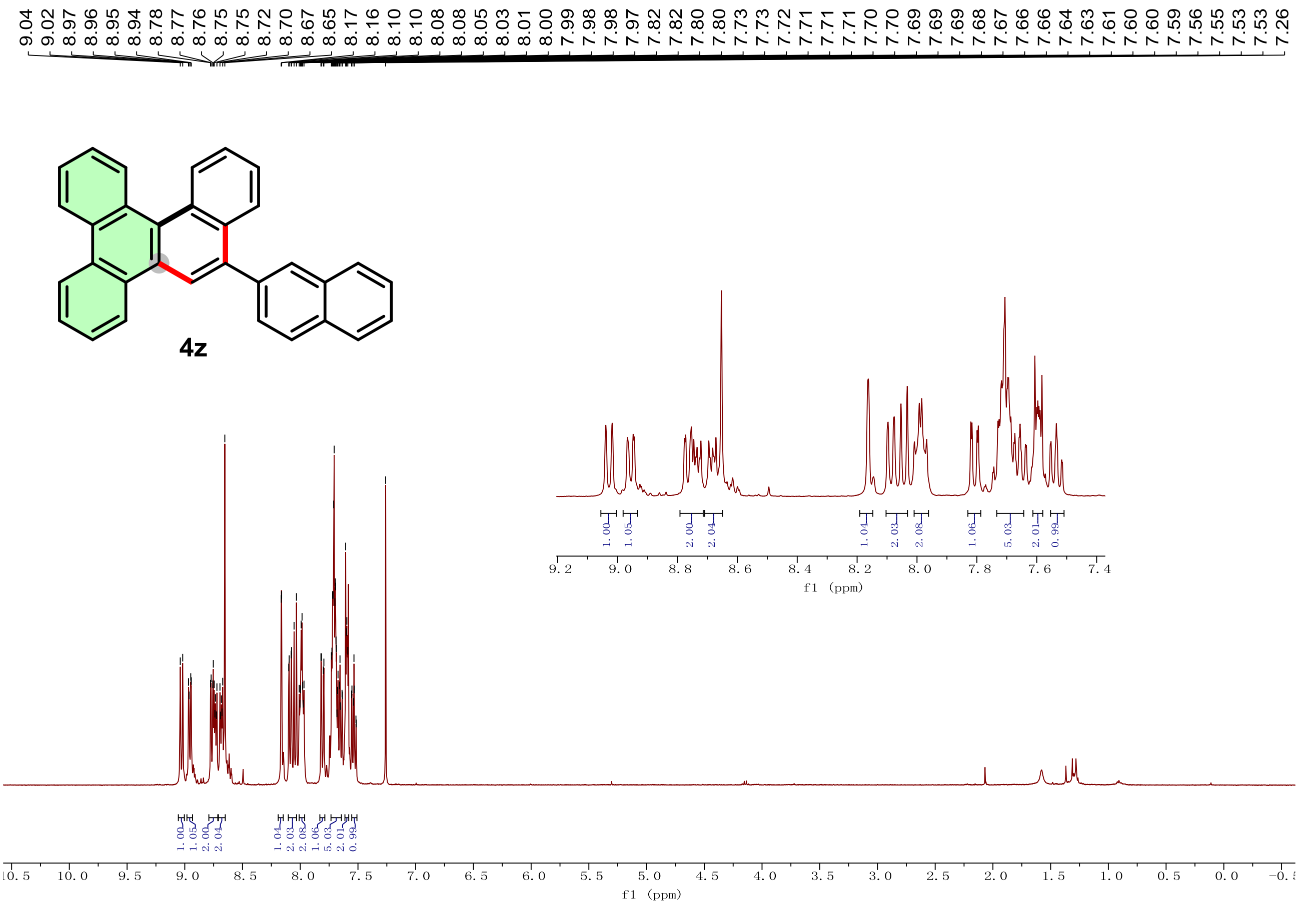

Supplementary Fig. 140. ¹³C NMR of 4z (101 MHz, $CDCl_3$)

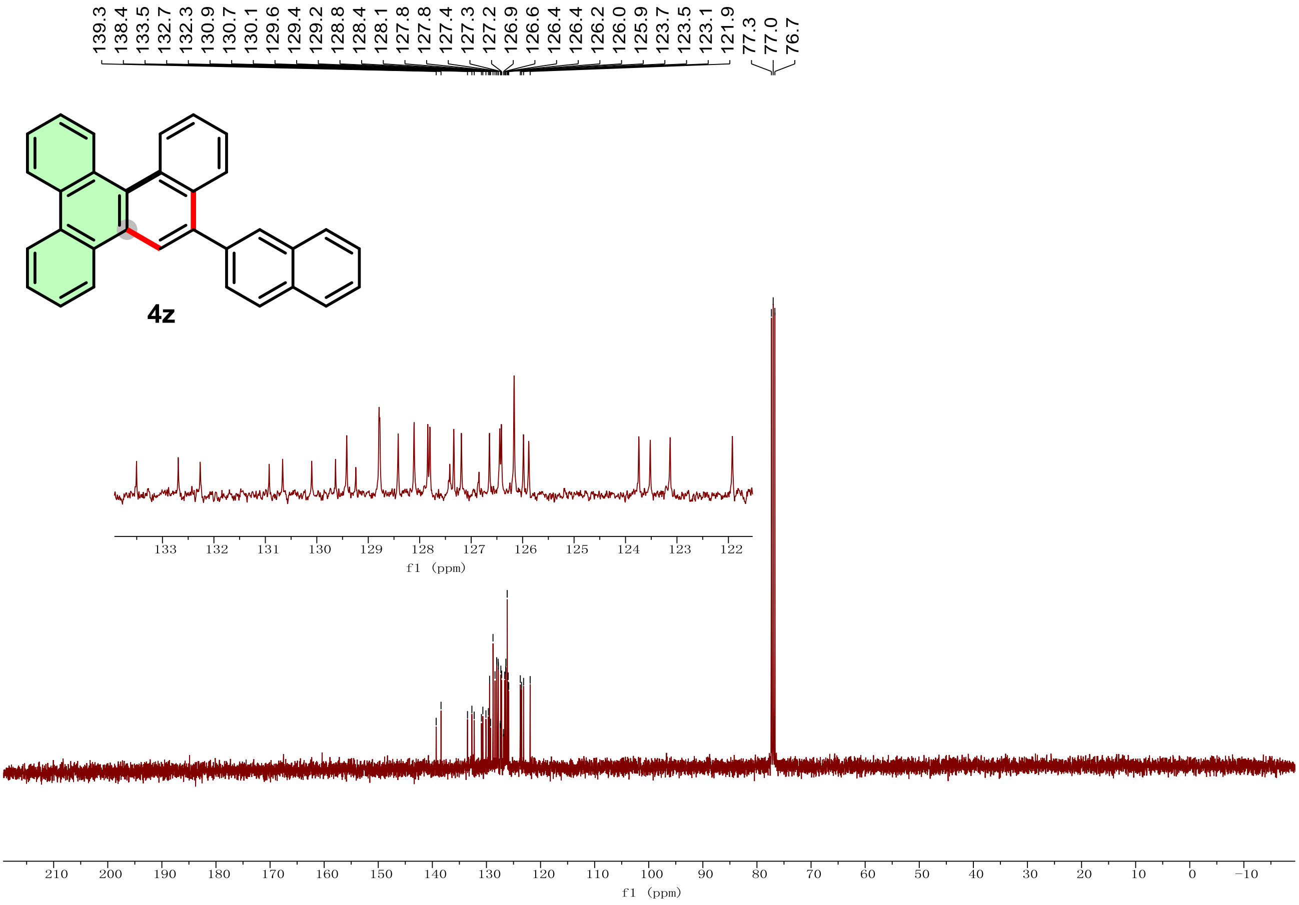

Supplementary Fig. 141. ^{1}H NMR of 4aa (400 MHz, $CDCl_3$)

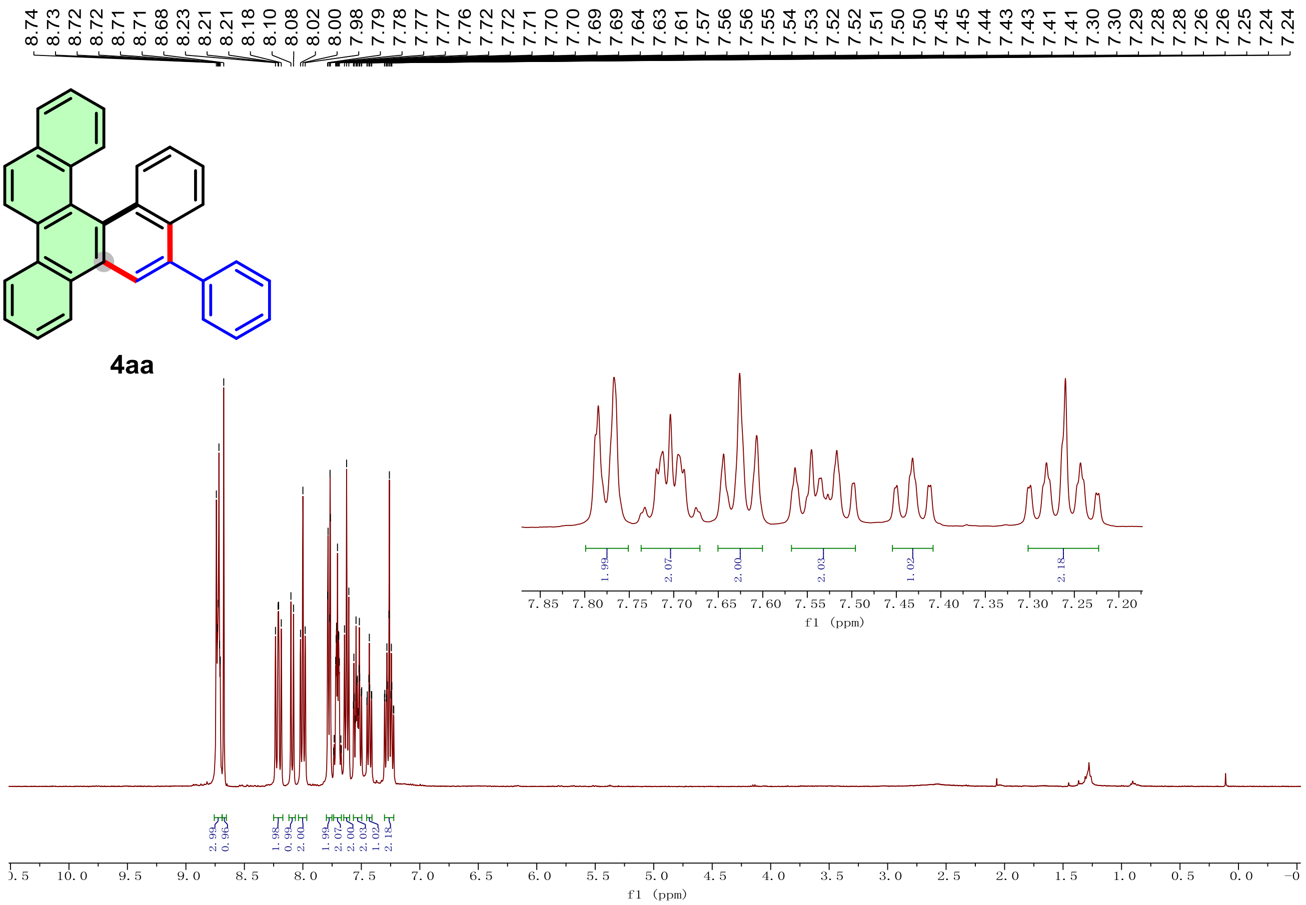

Supplementary Fig. 142. ^{13}C NMR of 4aa(101 MHz, $CDCl_3$)

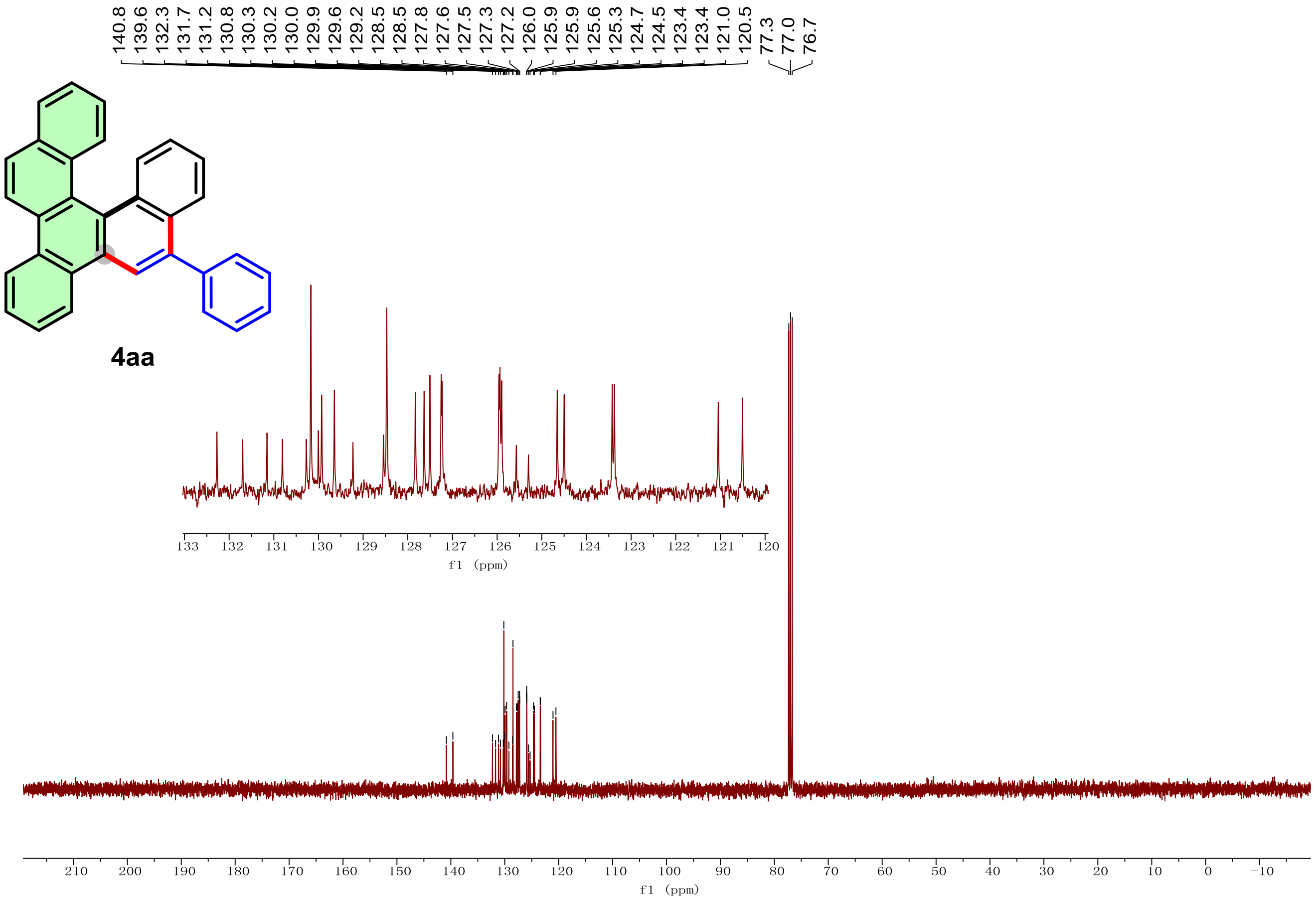

Supplementary Fig. 143. ^{1}H NMR of 4ab (600 MHz, $CDCl_3$)

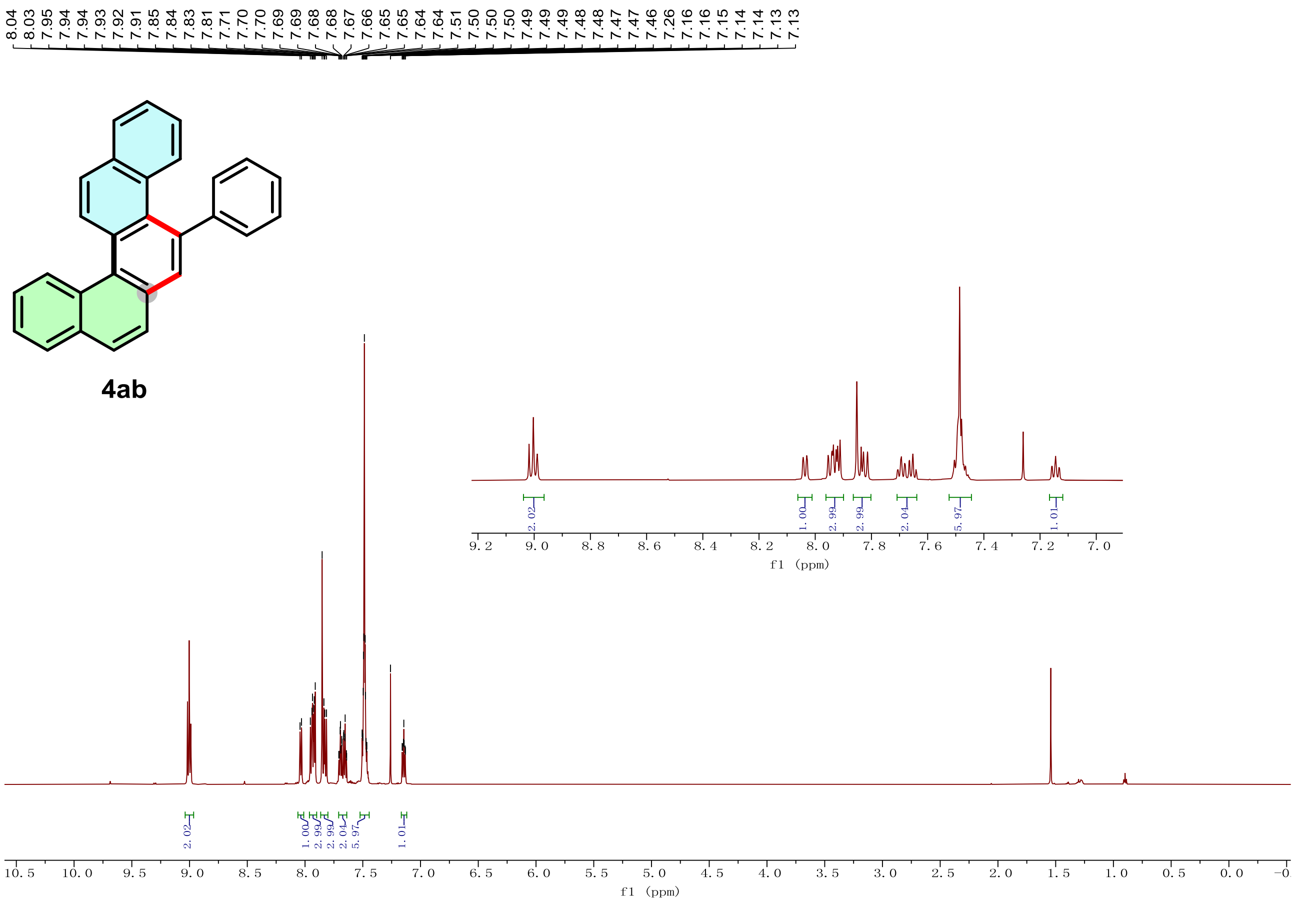

Supplementary Fig. 144. ^{13}C NMR of 4ab (151 MHz, $CDCl_3$)

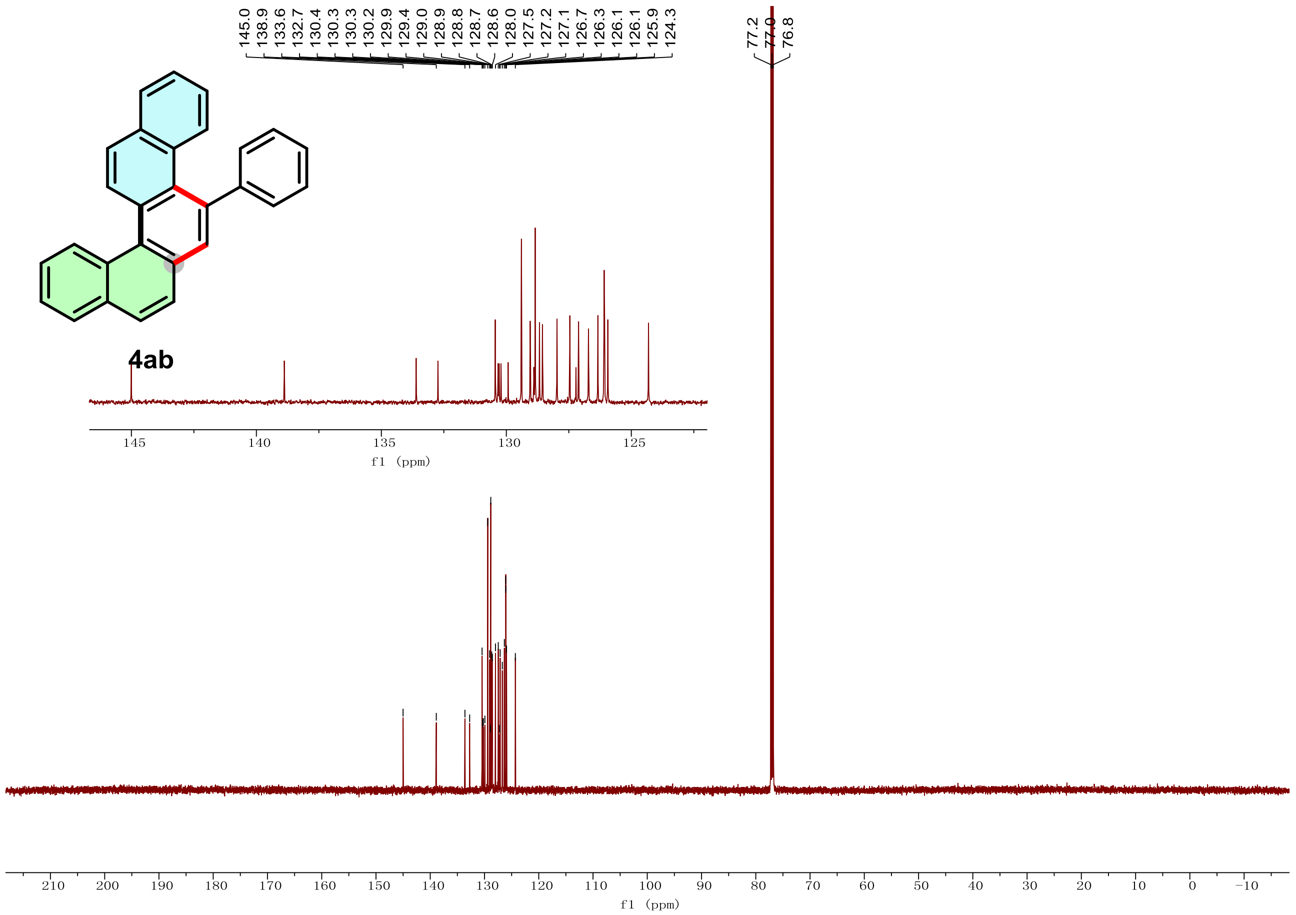

Supplementary Fig. 145. ^{1}H NMR of 5 (600 MHz, $CDCl_3$)

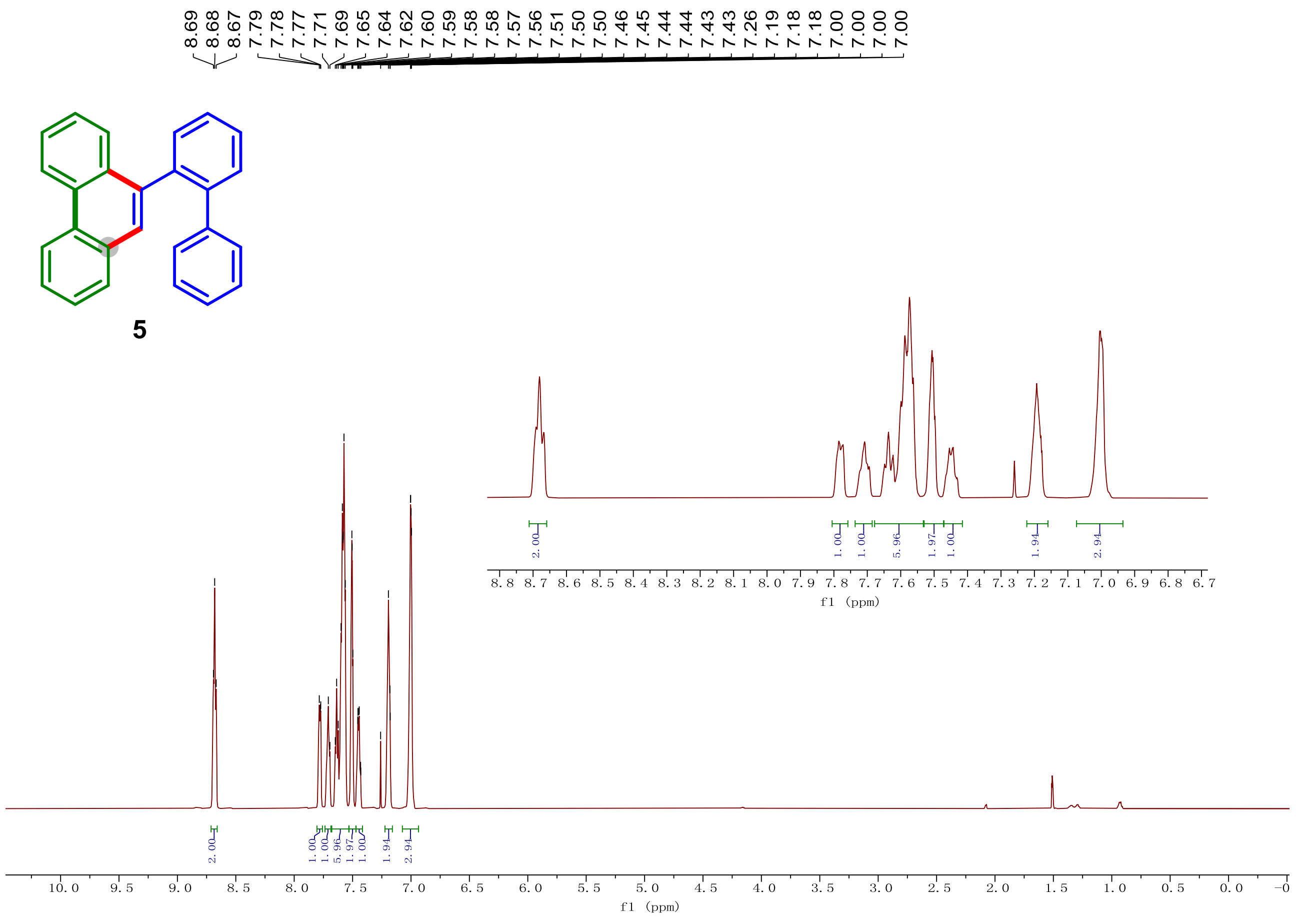

Supplementary Fig. 146. ^{13}C NMR of 5 (151 MHz, $CDCl_3$)

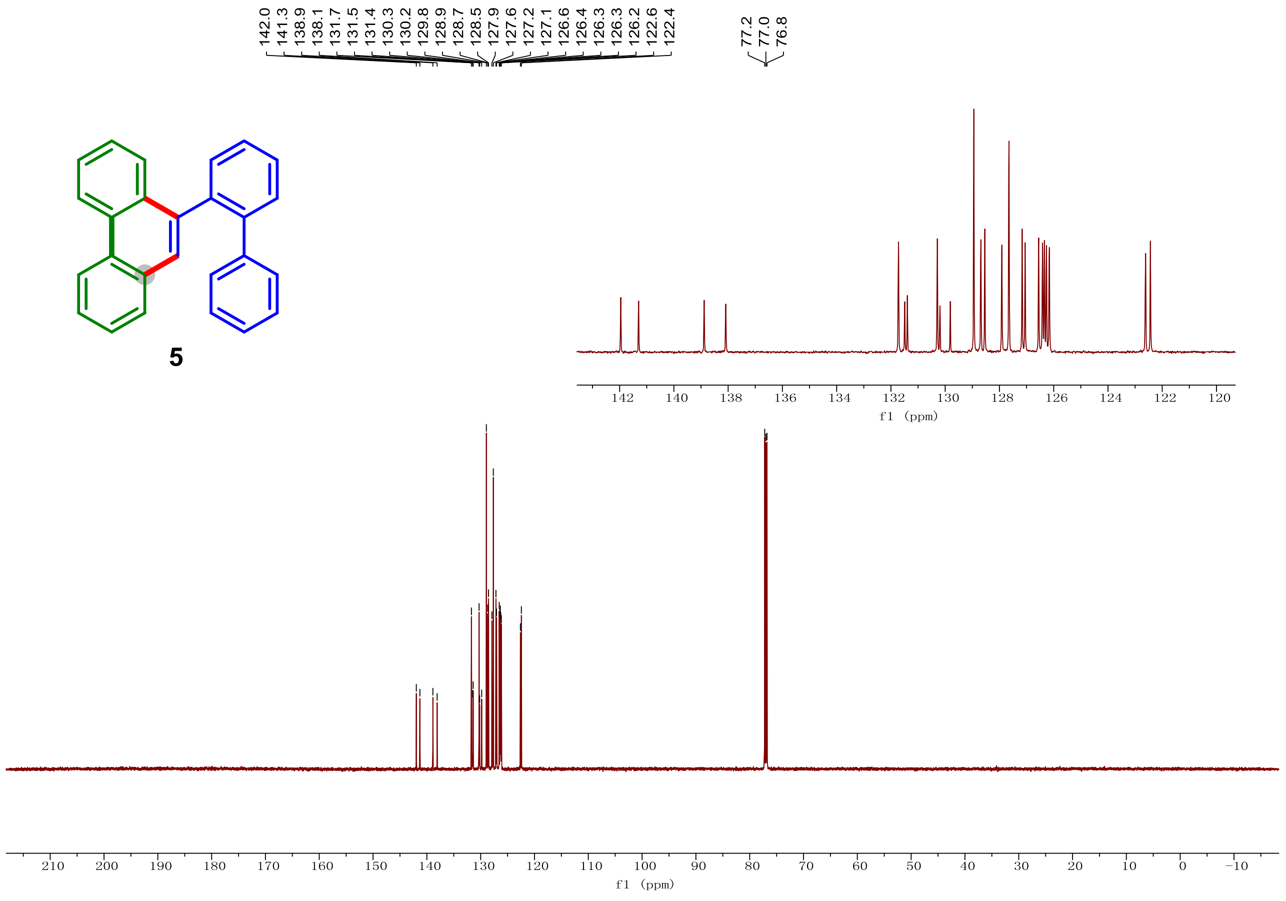

Supplementary Fig. 147. ^{1}H NMR of 6 (600 MHz, $CDCl_3$)

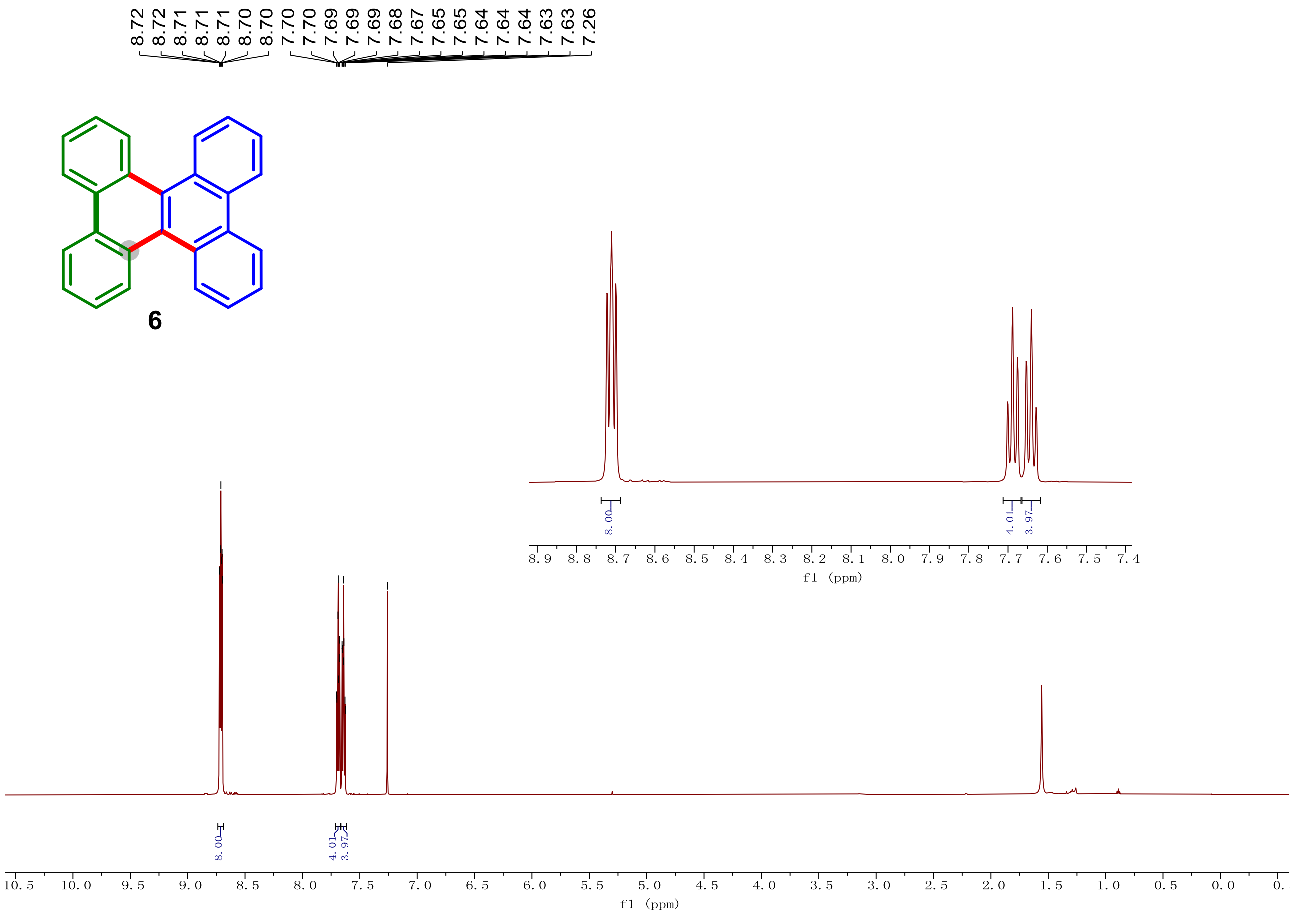

Supplementary Fig. 148. ^{13}C NMR of 6 (151 MHz, $CDCl_3$)

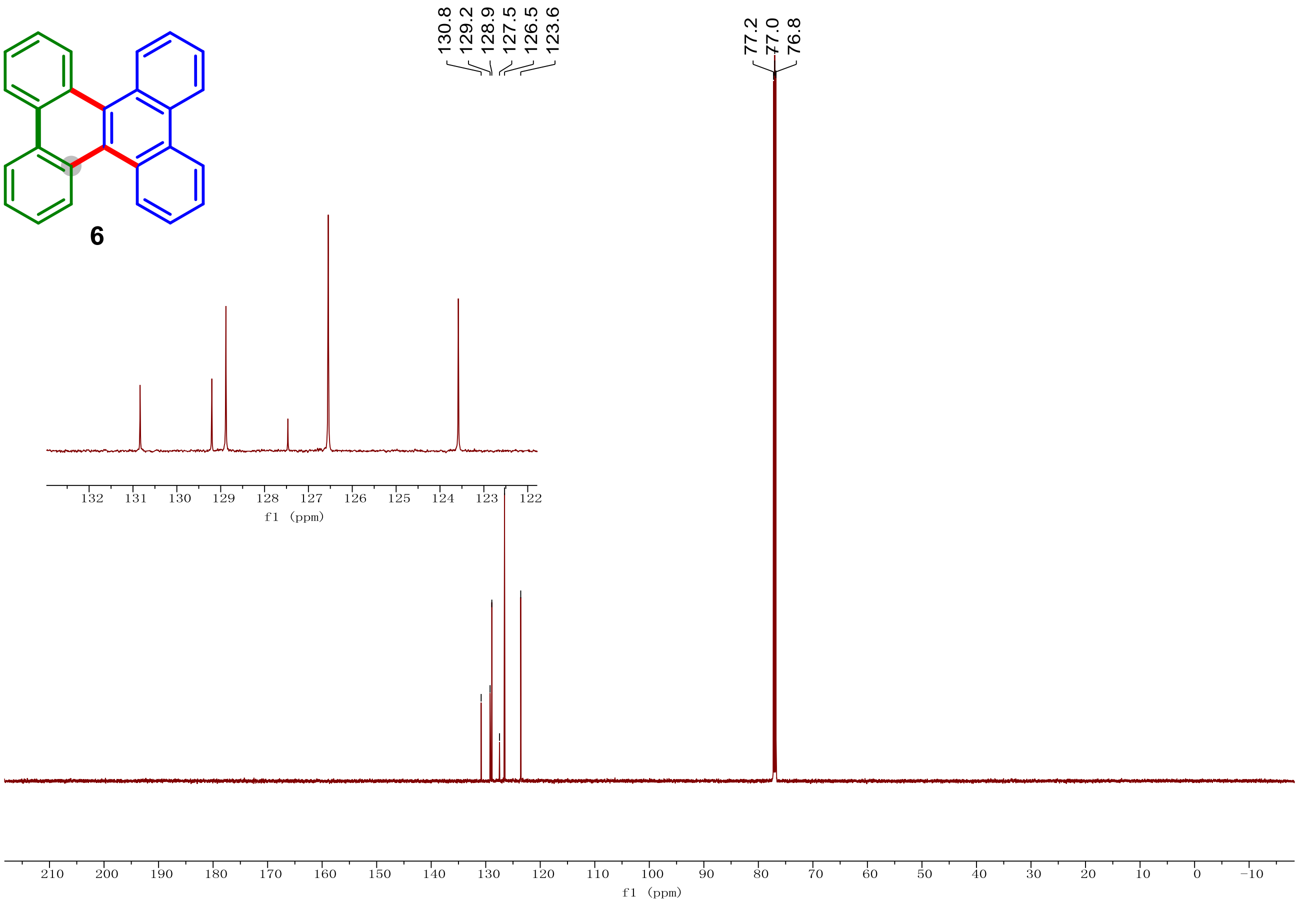

Supplementary Fig. 149. ^{1}H NMR of 8 (400 MHz, CDCl$_3$)

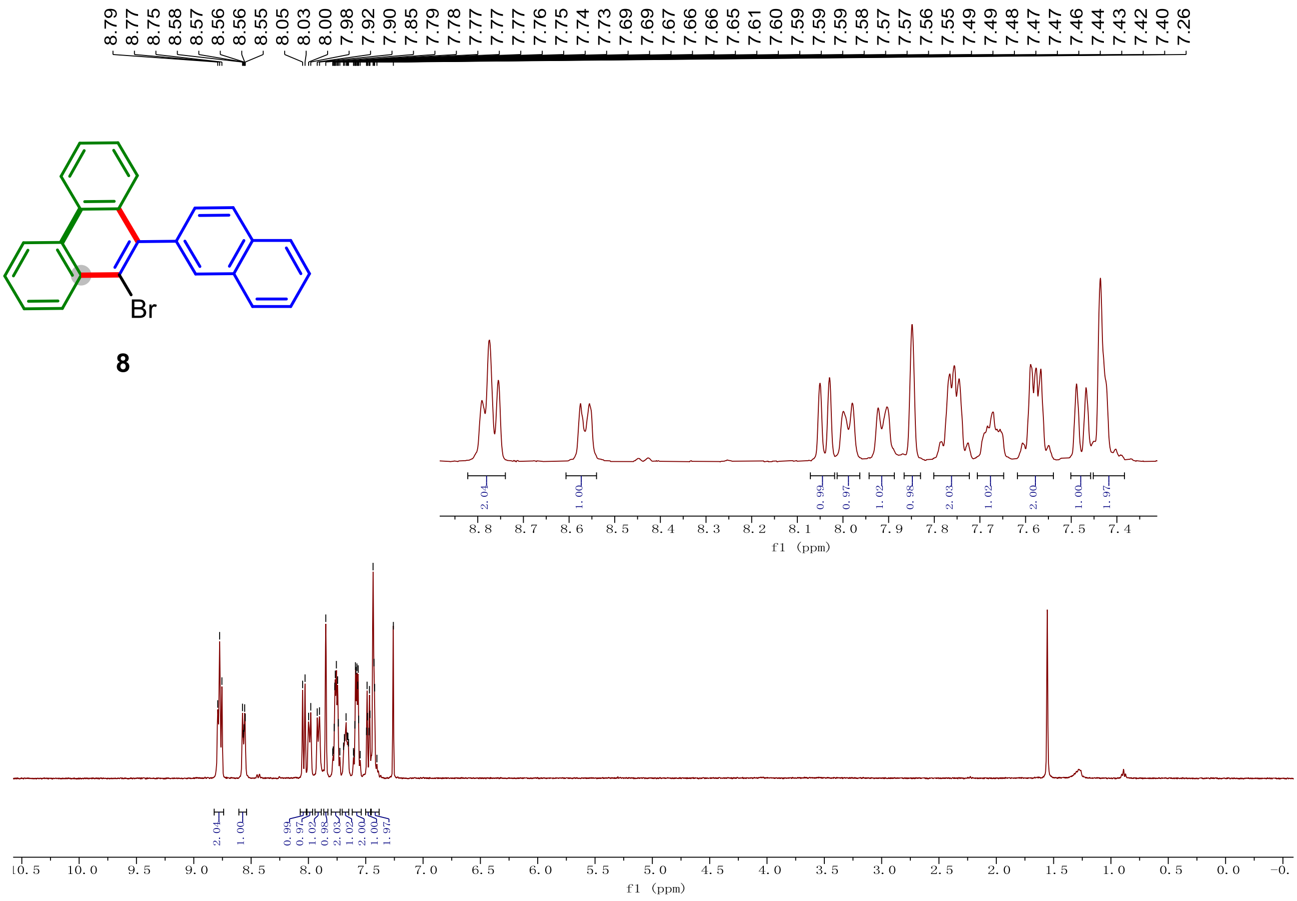

Supplementary Fig. 150. ^{13}C NMR of 8 (151 MHz, CDCl$_3$)

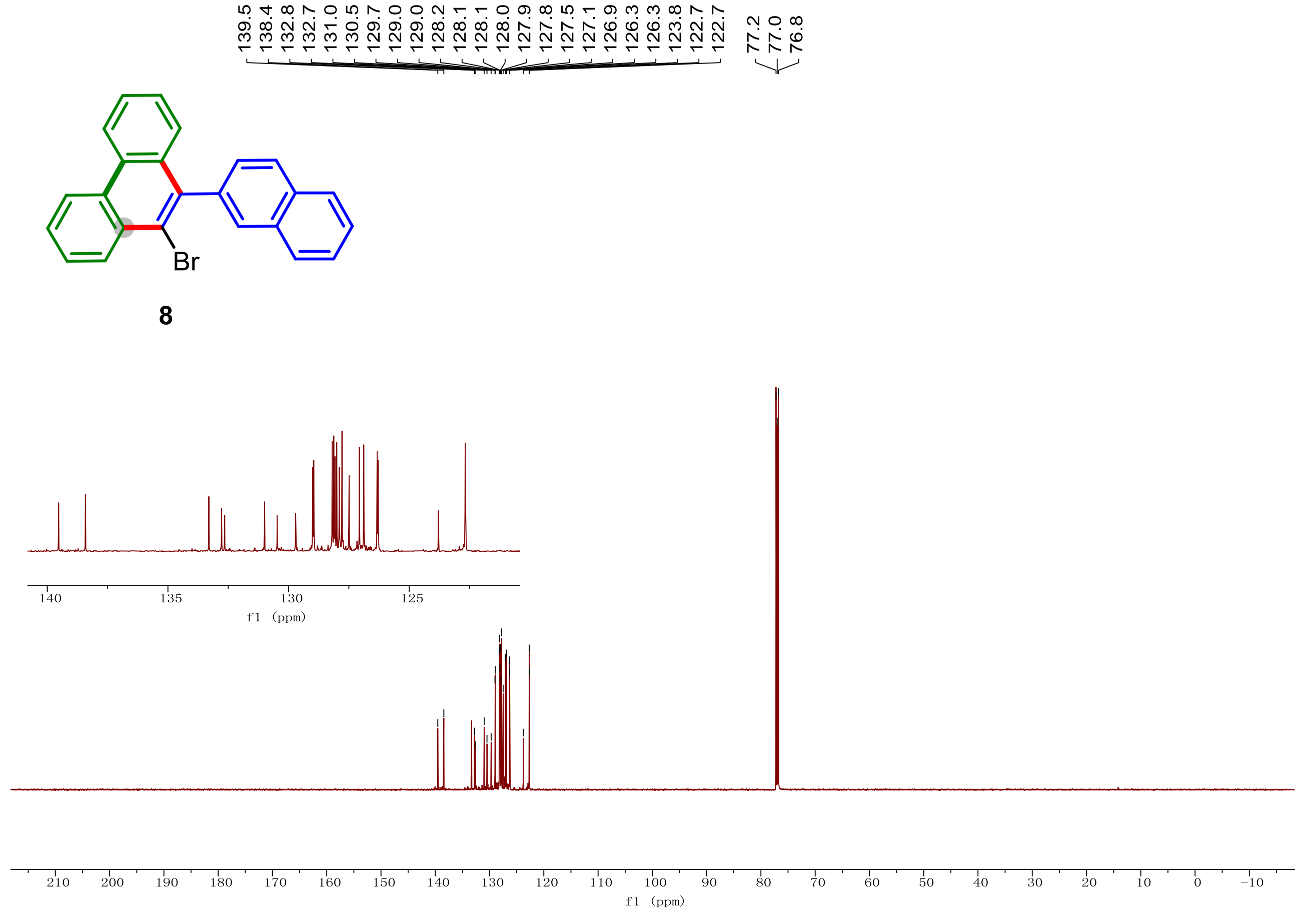

Supplementary Fig. 151. ^{1}H NMR of 9 (400 MHz, $CDCl_3$)

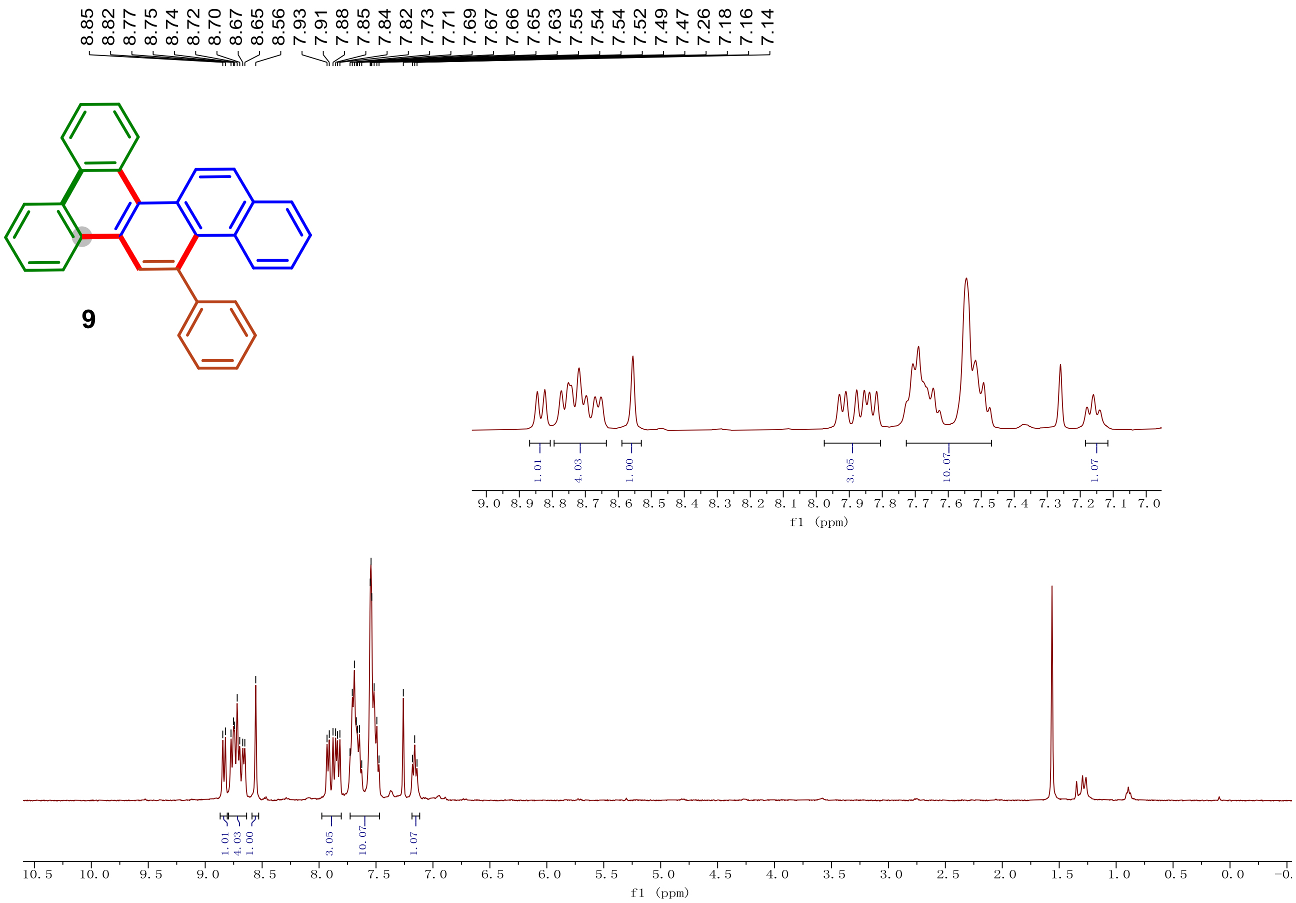

Supplementary Fig. 152. ^{13}C NMR of 9 (101 MHz, $CDCl_3$)

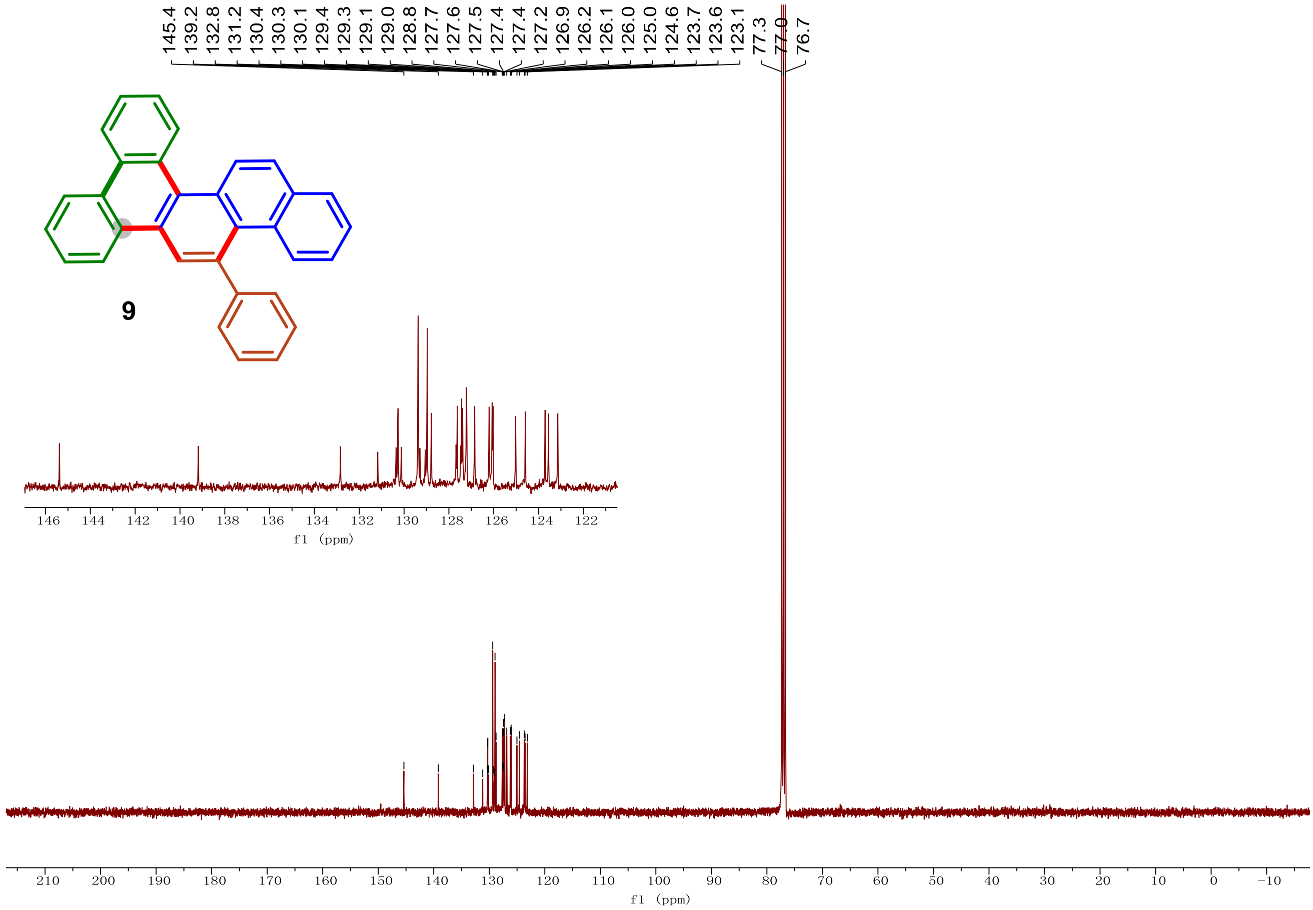

Supplementary Fig. 153. ¹H NMR of 10 (400 MHz, $CDCl_3$)

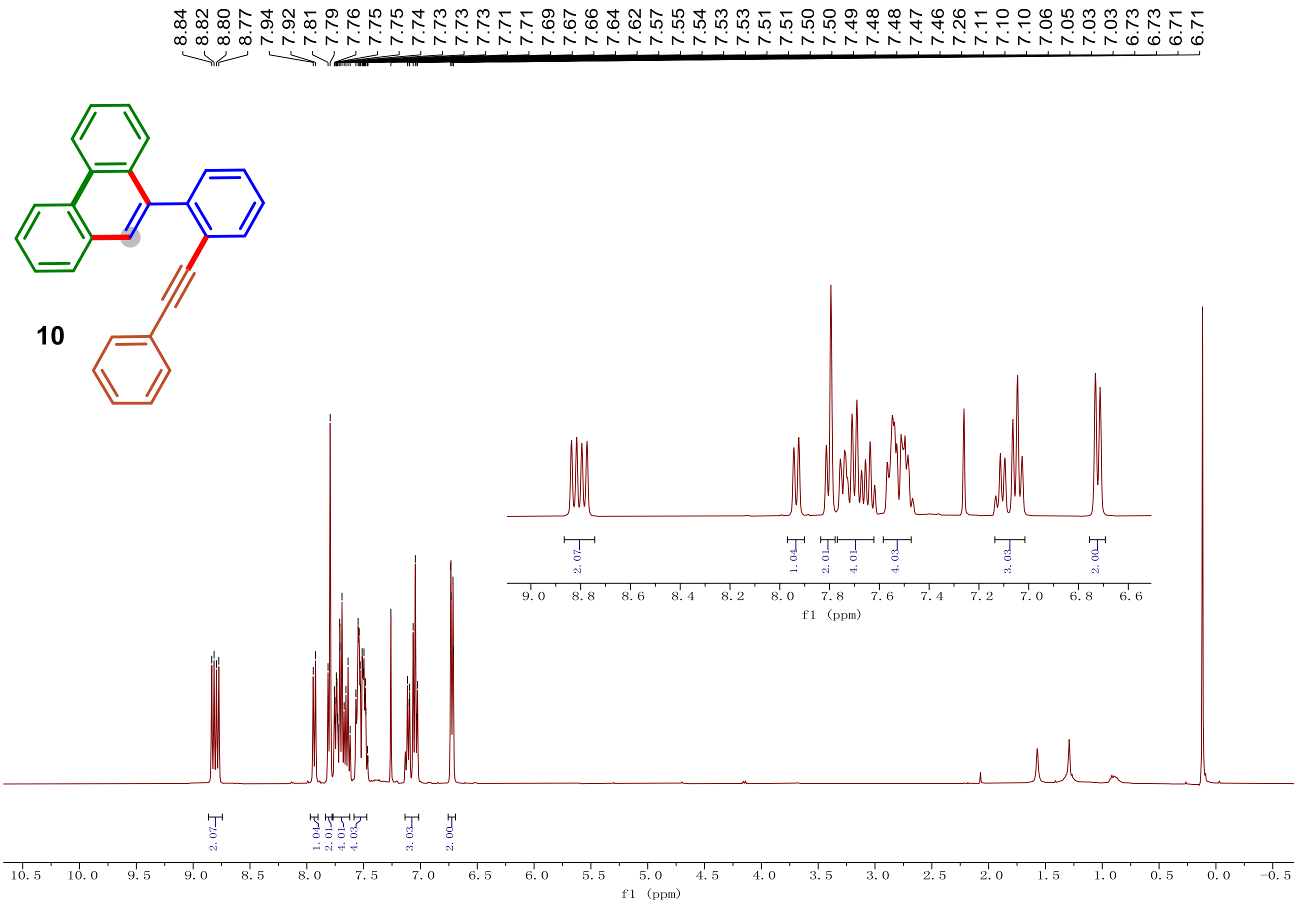

Supplementary Fig. 154. ¹³C NMR of 10 (101 MHz, $CDCl_3$)

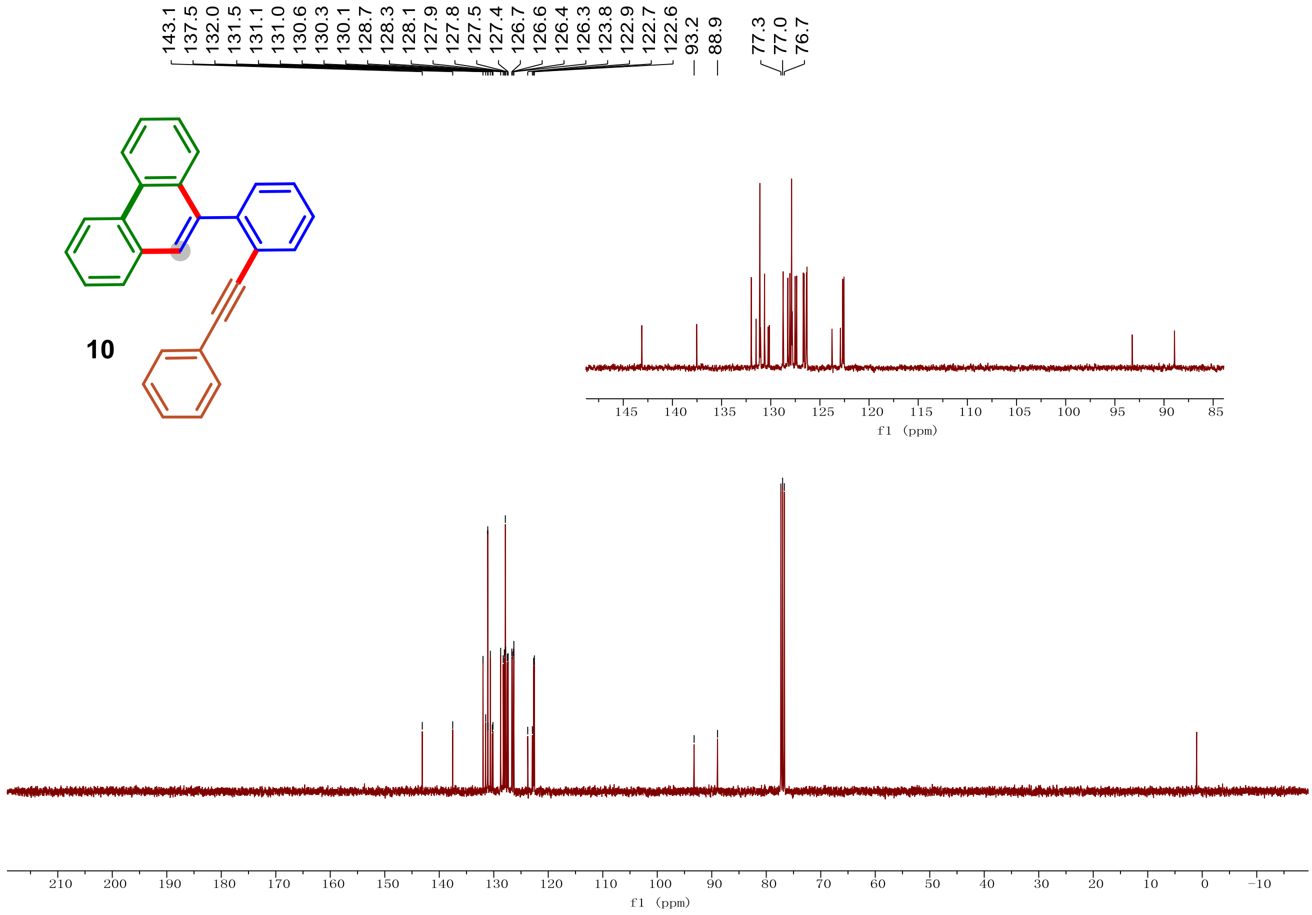

Supplementary Fig. 155. ^{1}H NMR of 11 (400 MHz, $CDCl_3$)

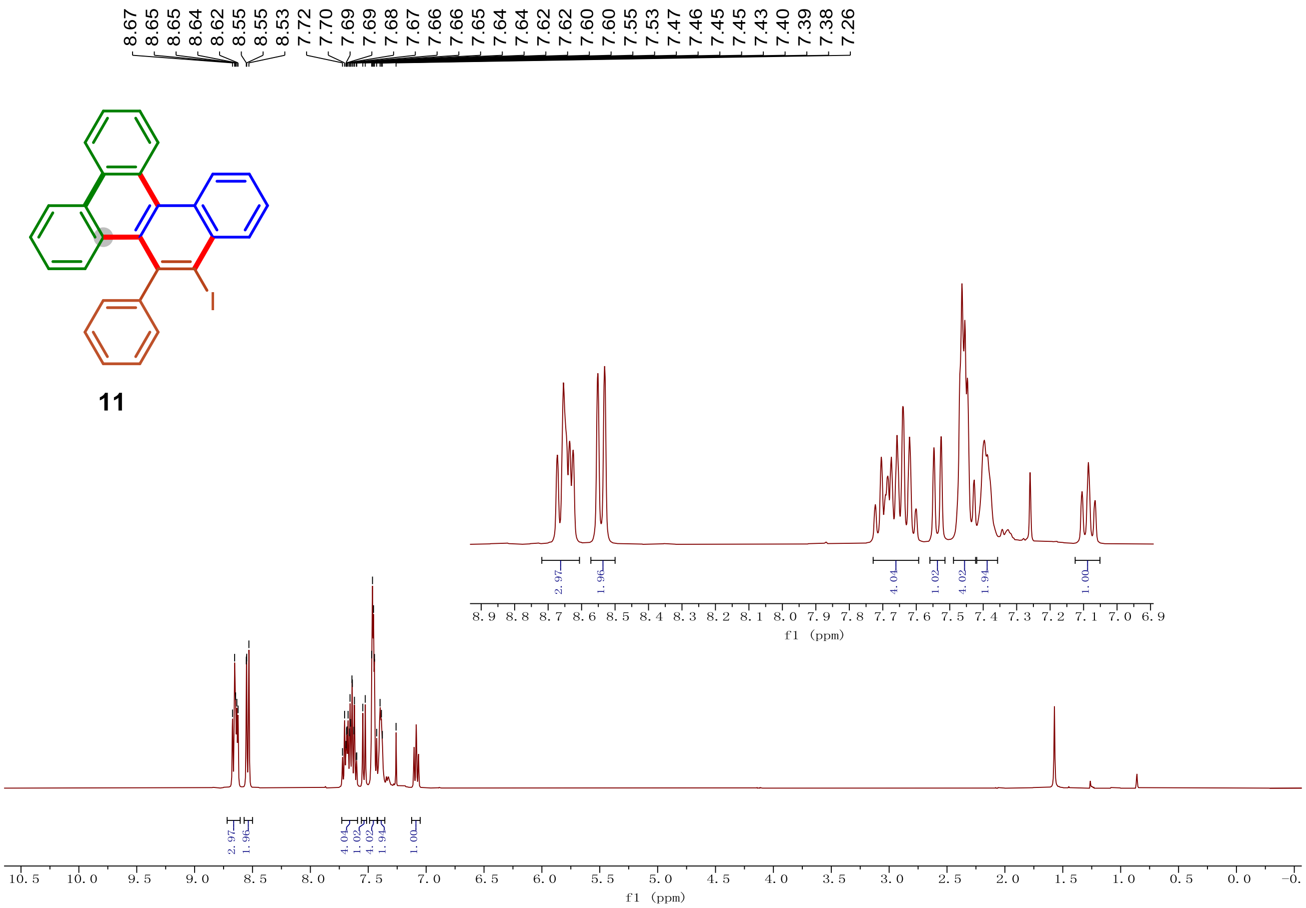

Supplementary Fig. 156. ^{13}C NMR of 11 (101 MHz, $CDCl_3$)

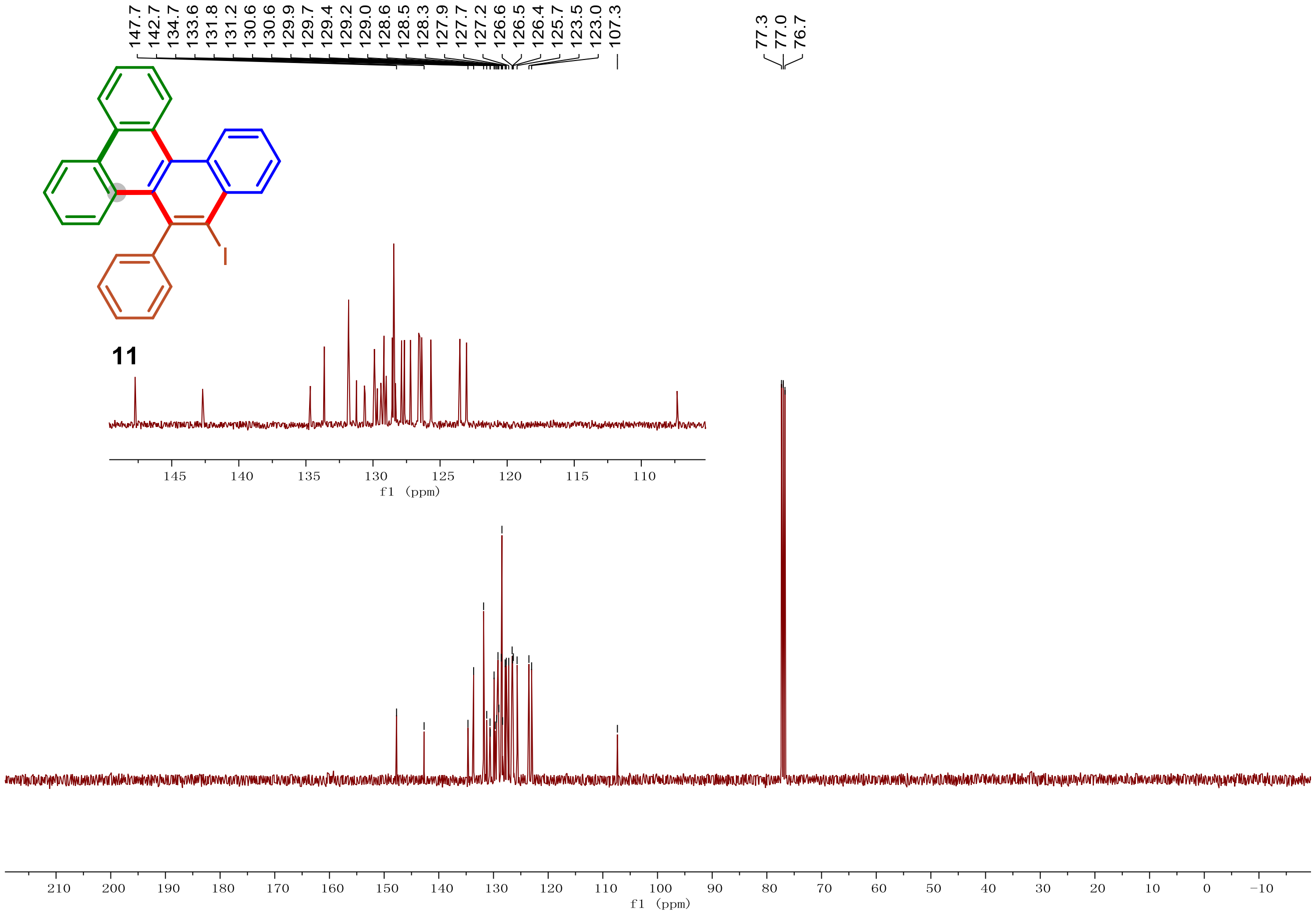

Supplementary Fig. 157. ^{1}H NMR of 12 (400 MHz, $CDCl_3$)

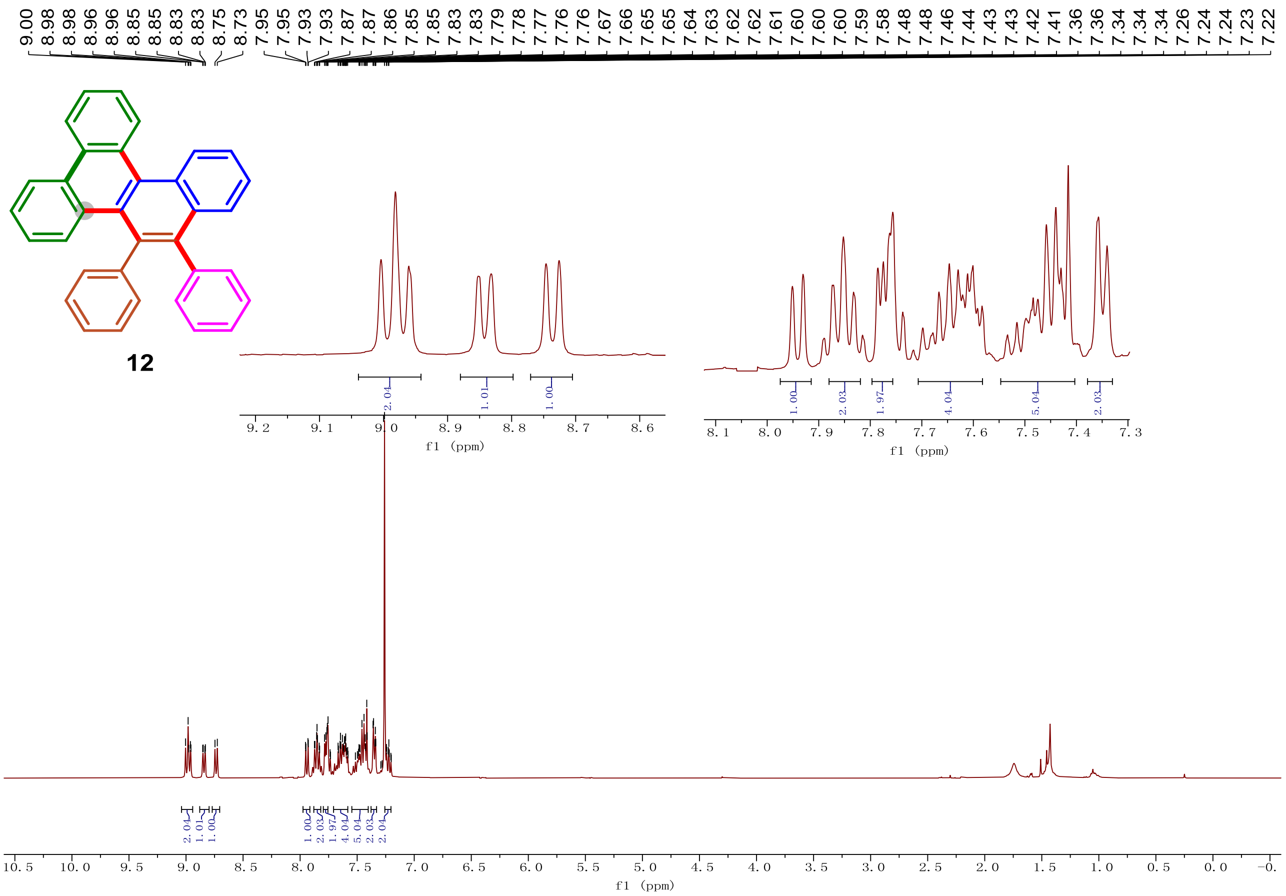

Supplementary Fig. 158. ^{13}C NMR of 12 (101 MHz, $CDCl_3$)

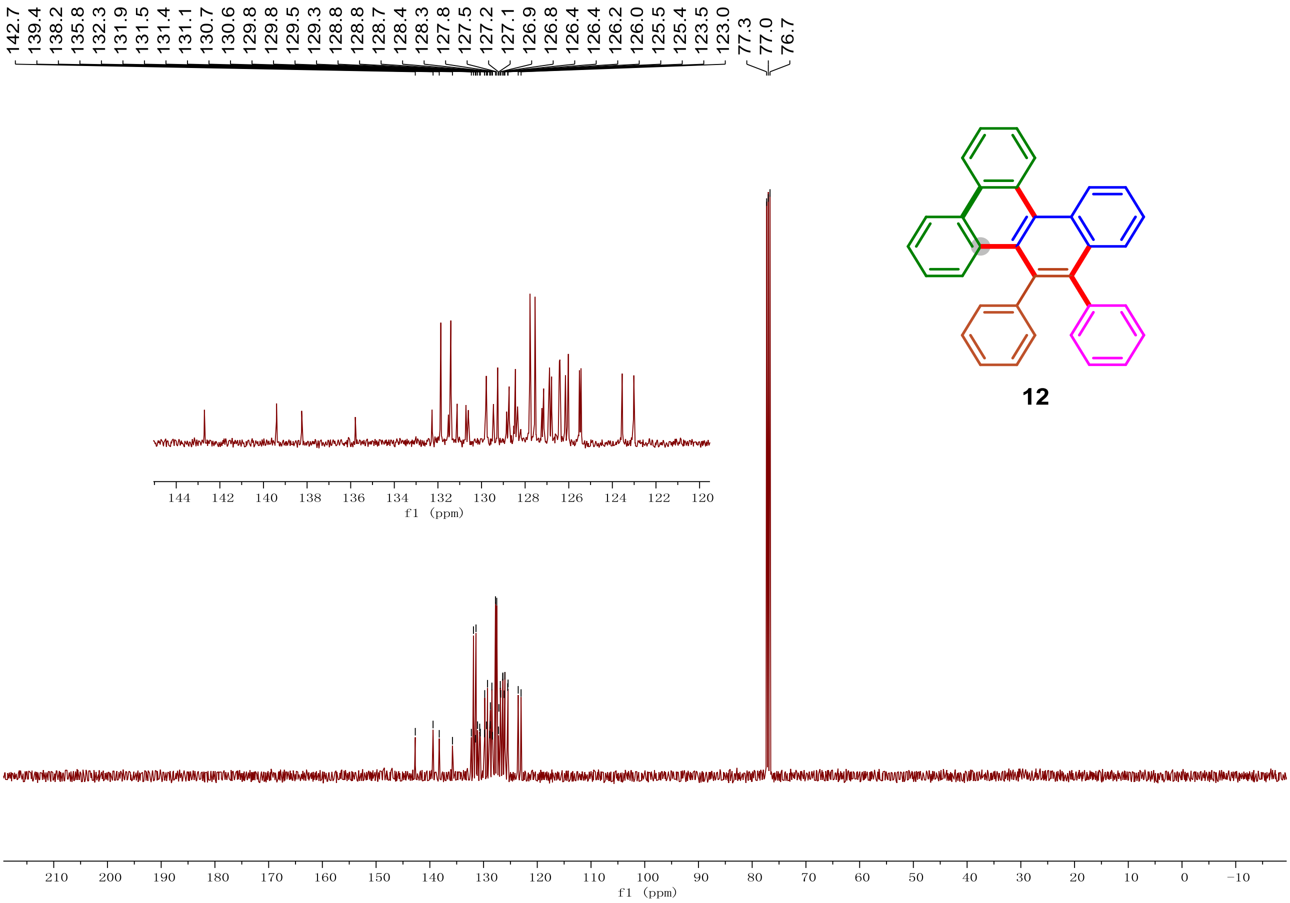

Supplementary Fig. 159. ^{1}H NMR of 13 (400 MHz, $CDCl_3$)

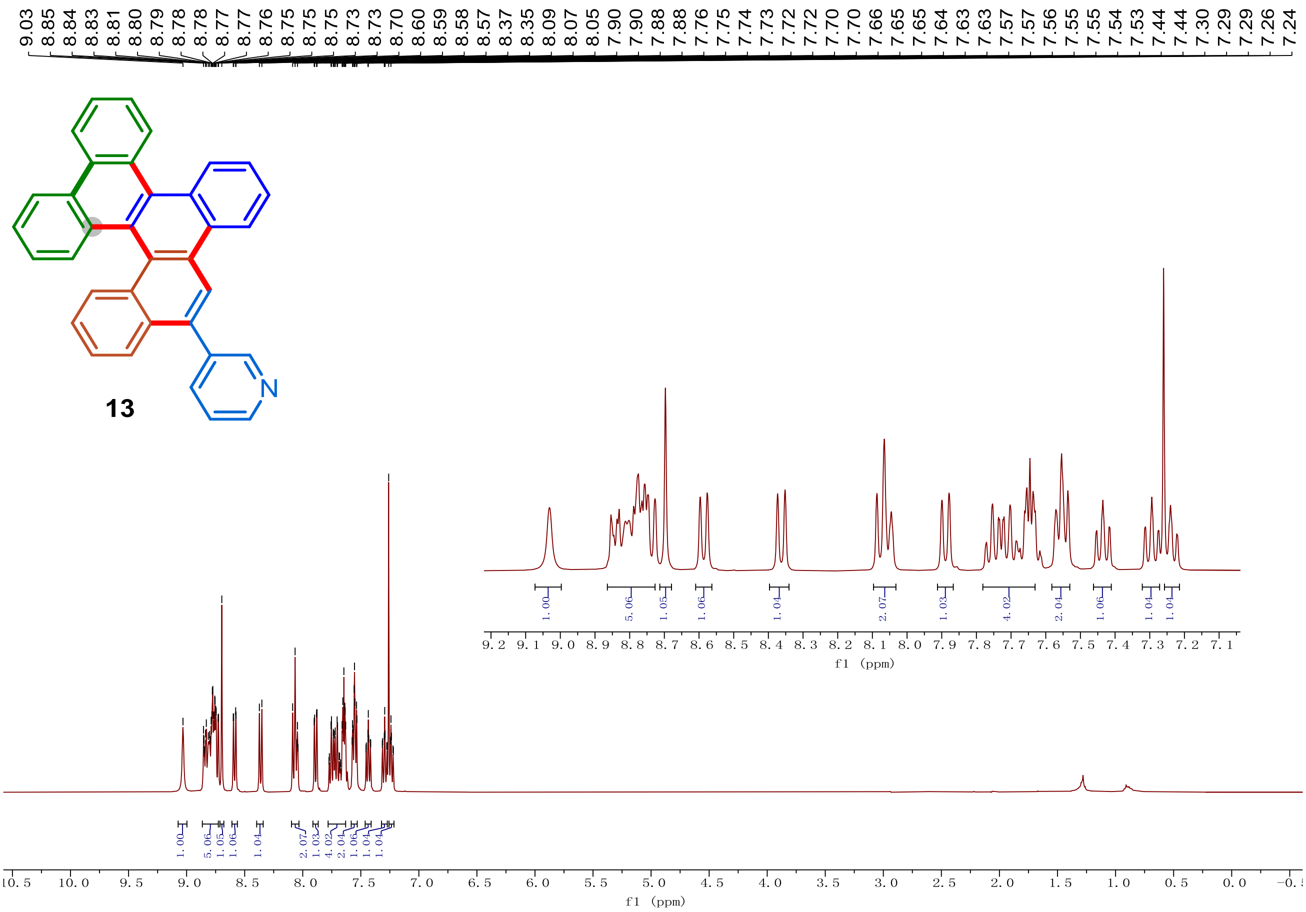

Supplementary Fig. 160. ^{13}C NMR of 13 (101 MHz, $CDCl_3$)

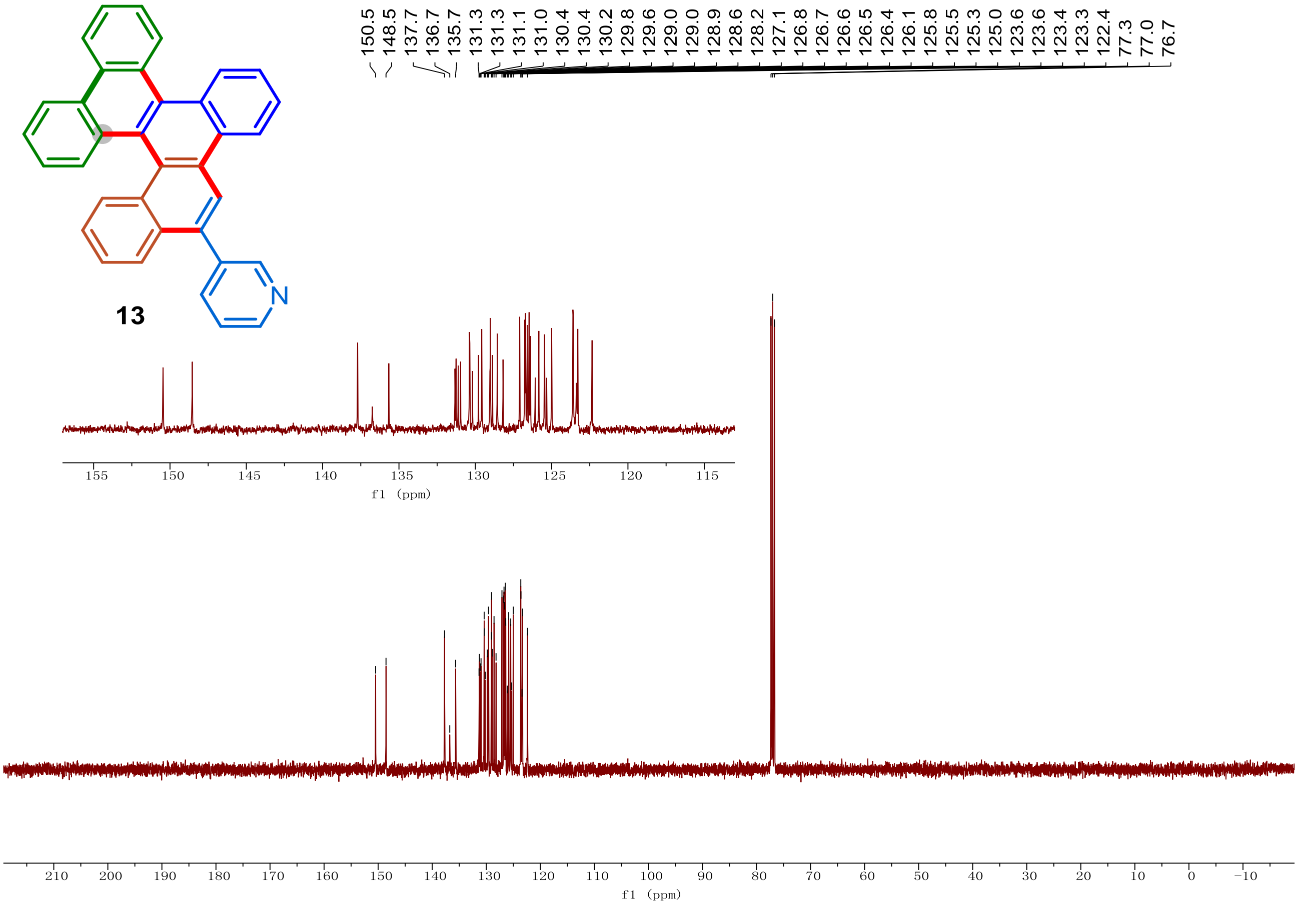

Supplementary Fig. 161. ^{1}H NMR of 15 (400 MHz, $CDCl_3$)

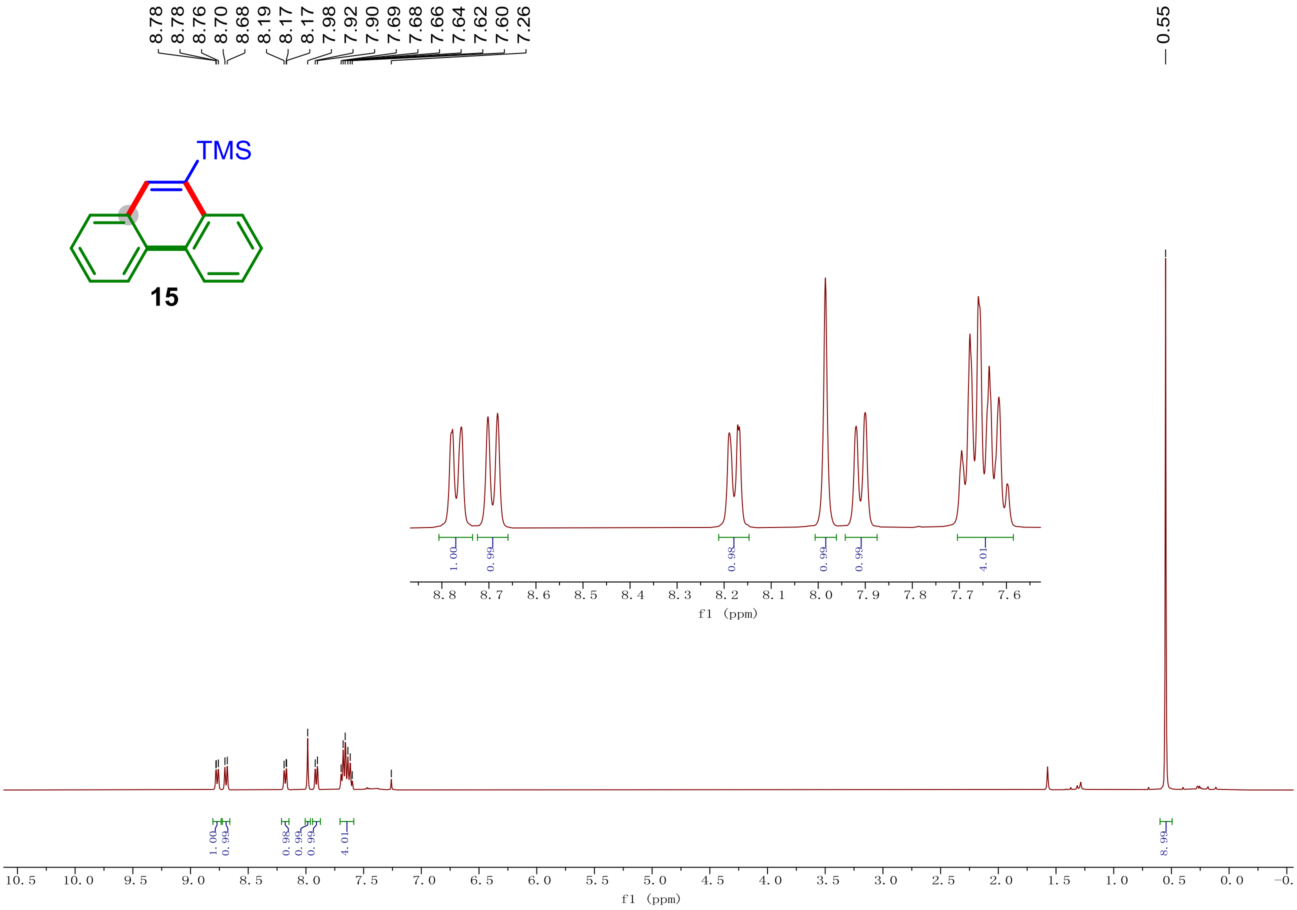

Supplementary Fig. 162. ^{13}C NMR of 15 (101 MHz, $CDCl_3$)

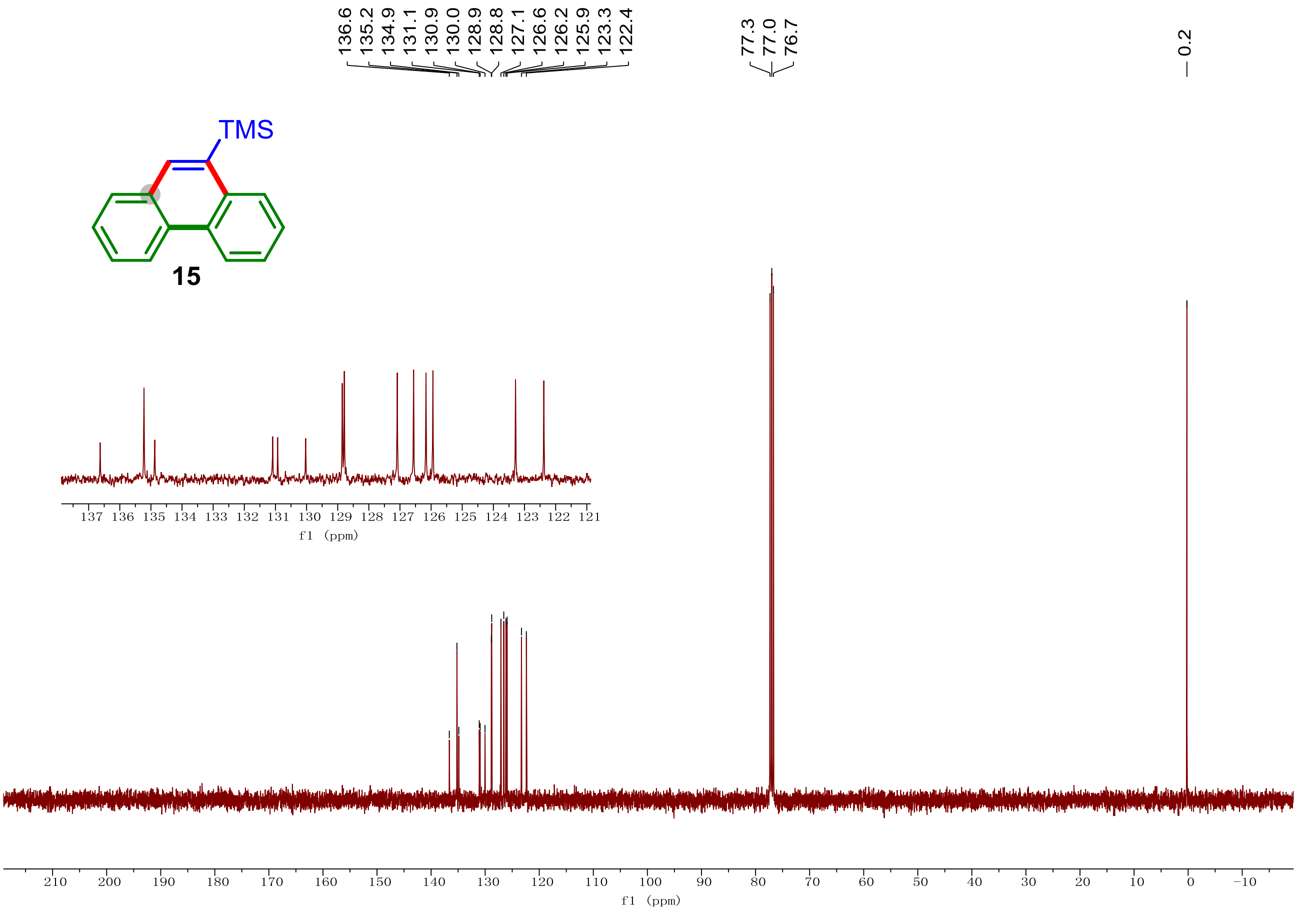

Supplementary Fig. 163. ^{1}H NMR of 16 (400 MHz, $CDCl_3$)

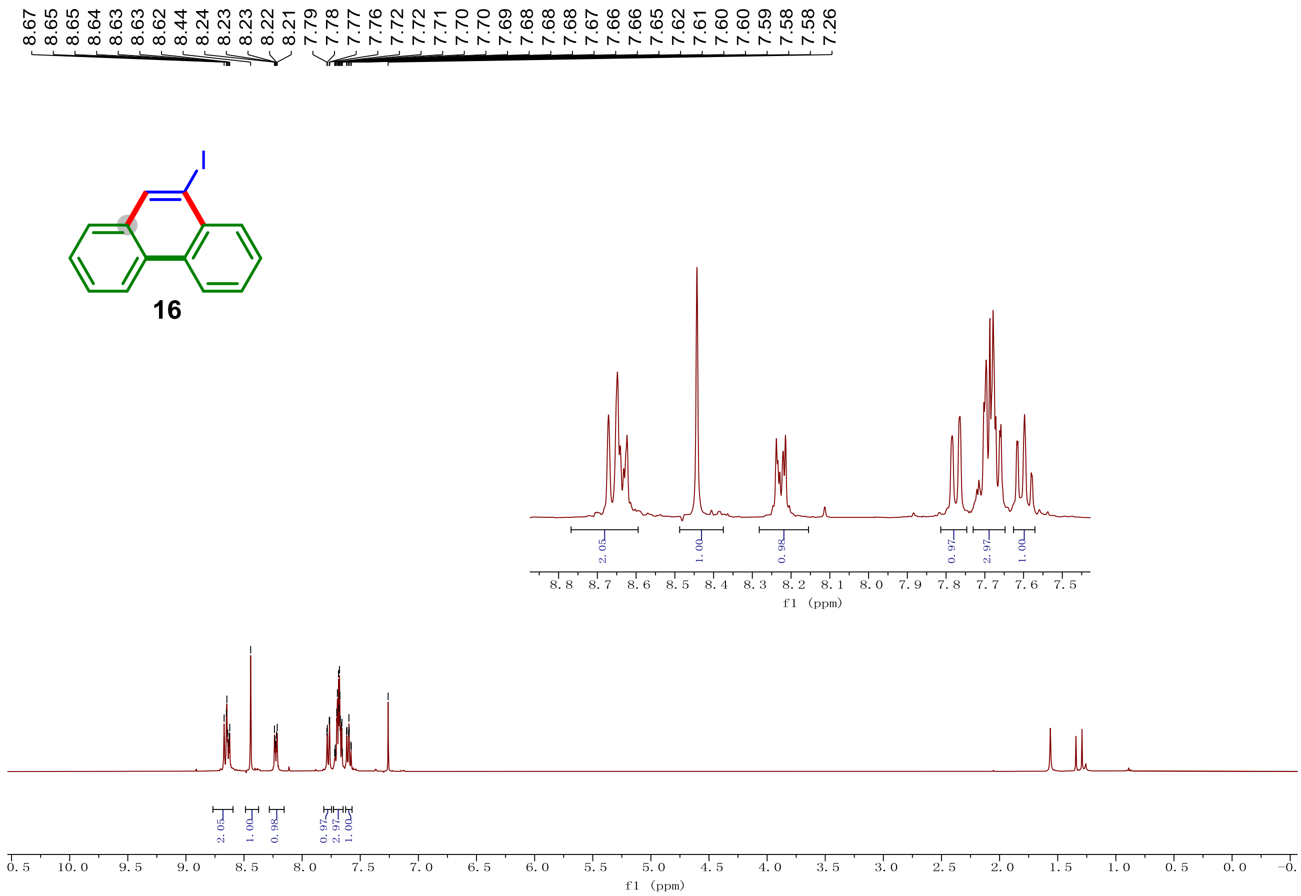

Supplementary Fig. 164. ^{13}C NMR of 16 (151 MHz, $CDCl_3$)

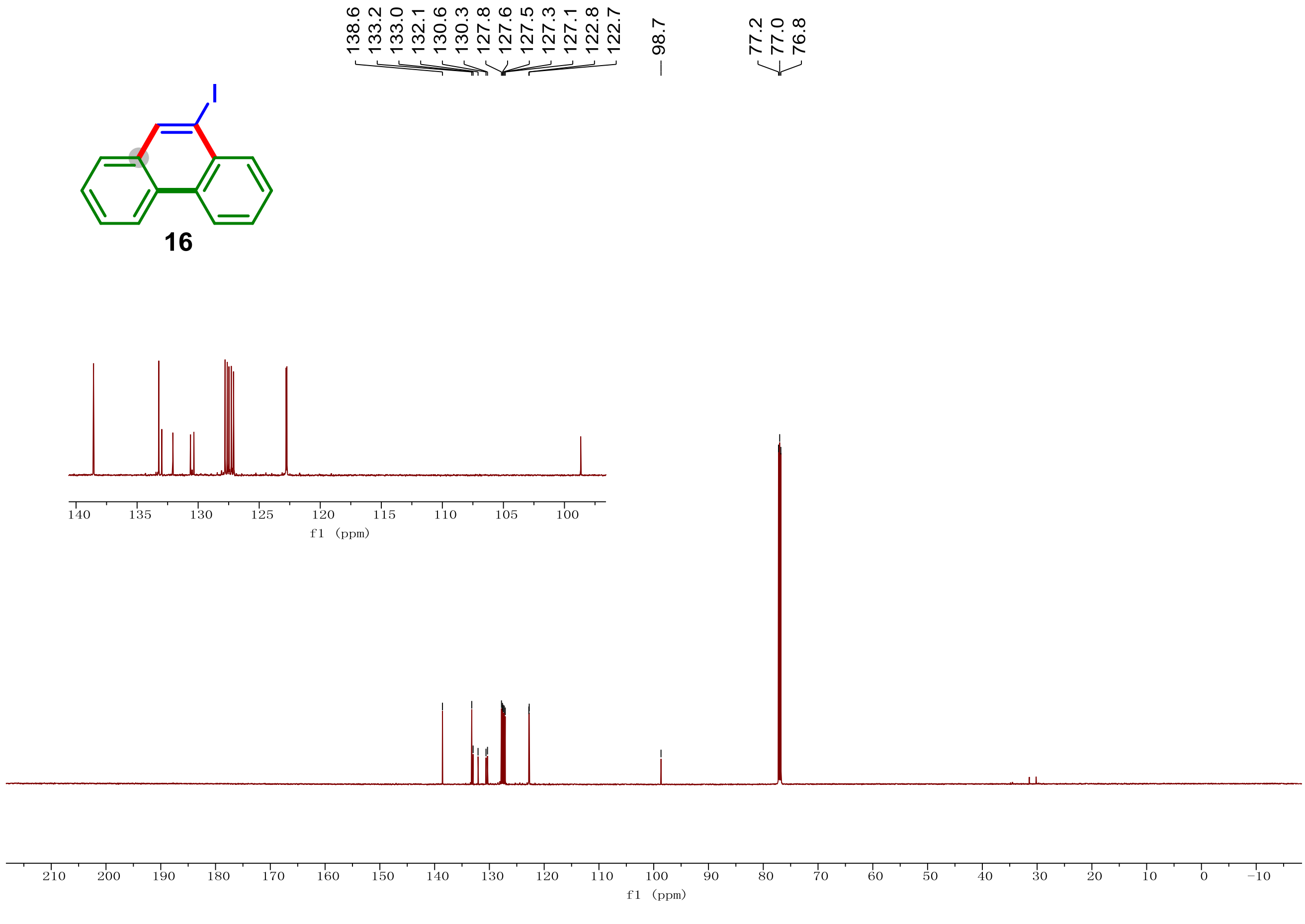

Supplementary Fig. 165. ^{1}H NMR of 17 (400 MHz, $CDCl_3$)

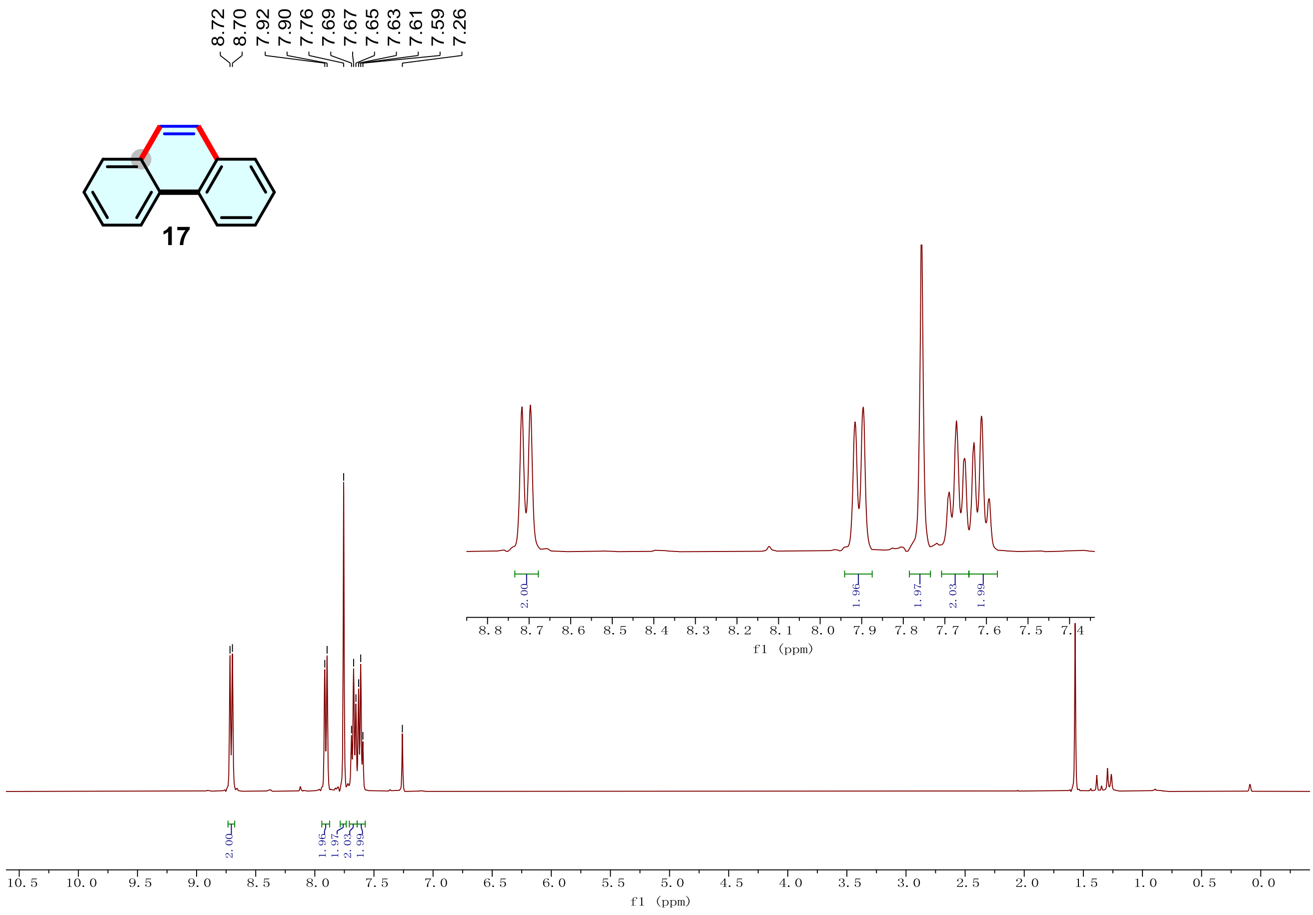

Supplementary Fig. 166. ^{13}C NMR of 17 (101 MHz, $CDCl_3$)

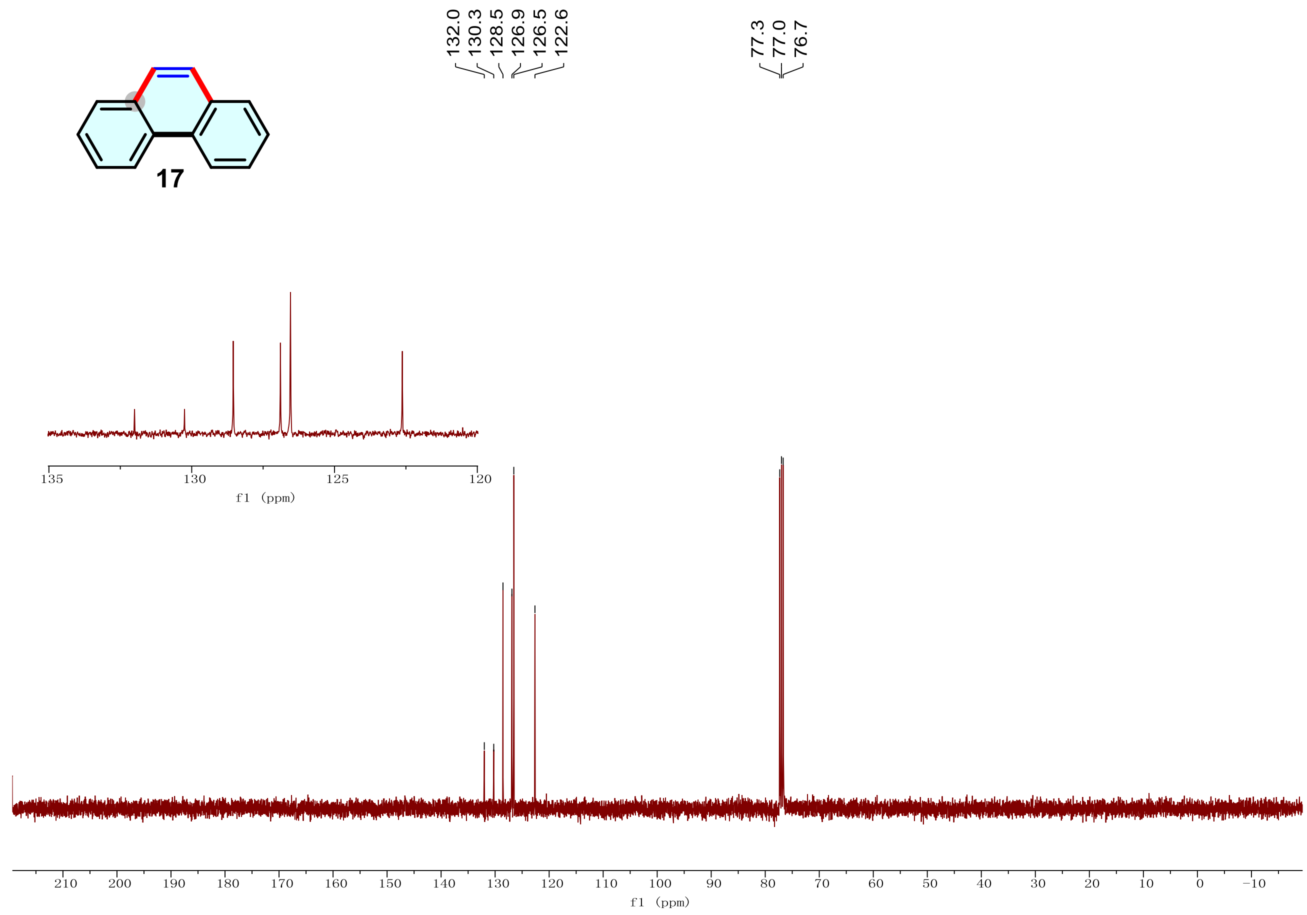

Supplementary Fig. 167. ^{1}H NMR of 18 (600 MHz, $CDCl_3$)

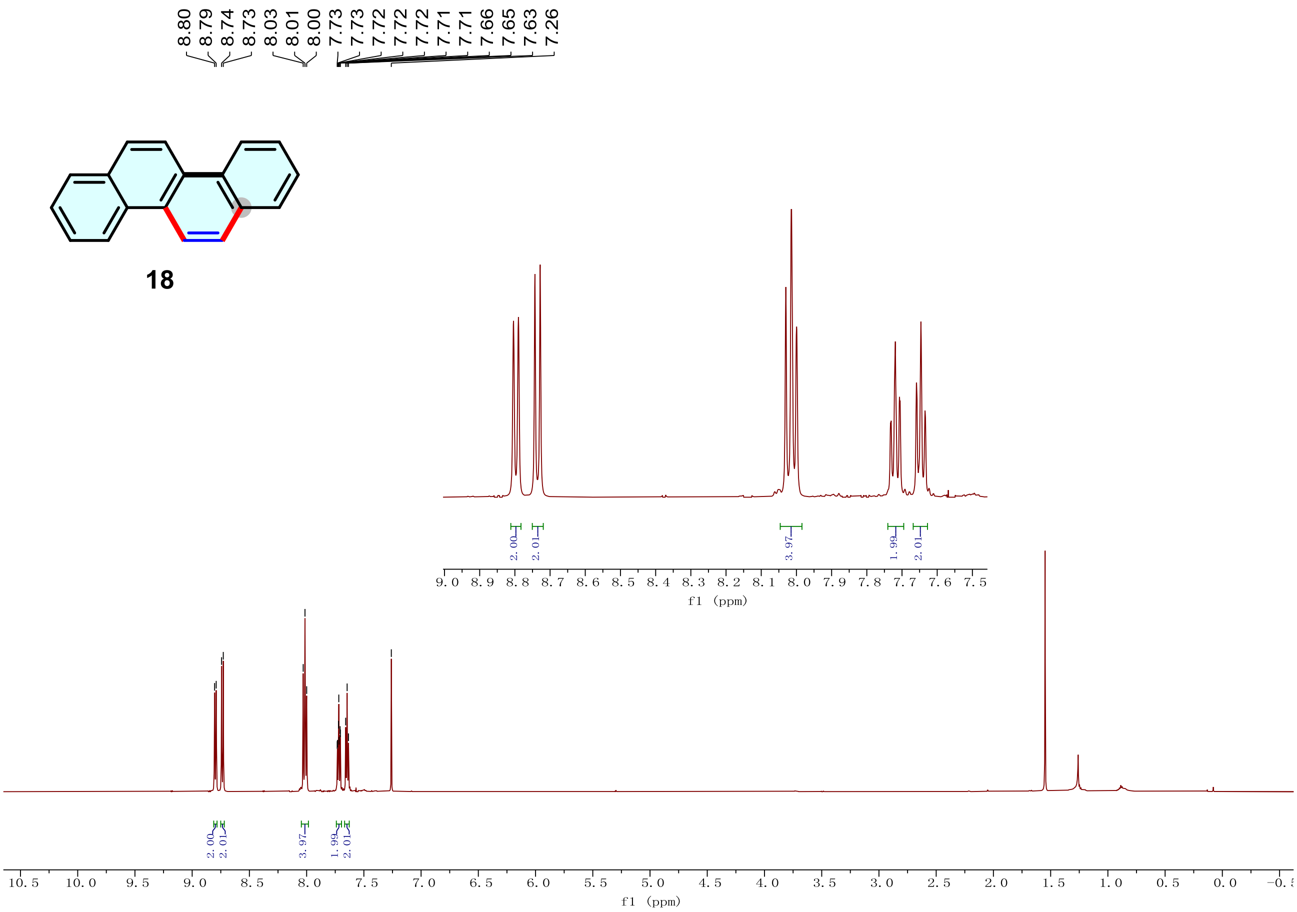

Supplementary Fig. 168. ^{13}C NMR of 18 (151 MHz, $CDCl_3$)

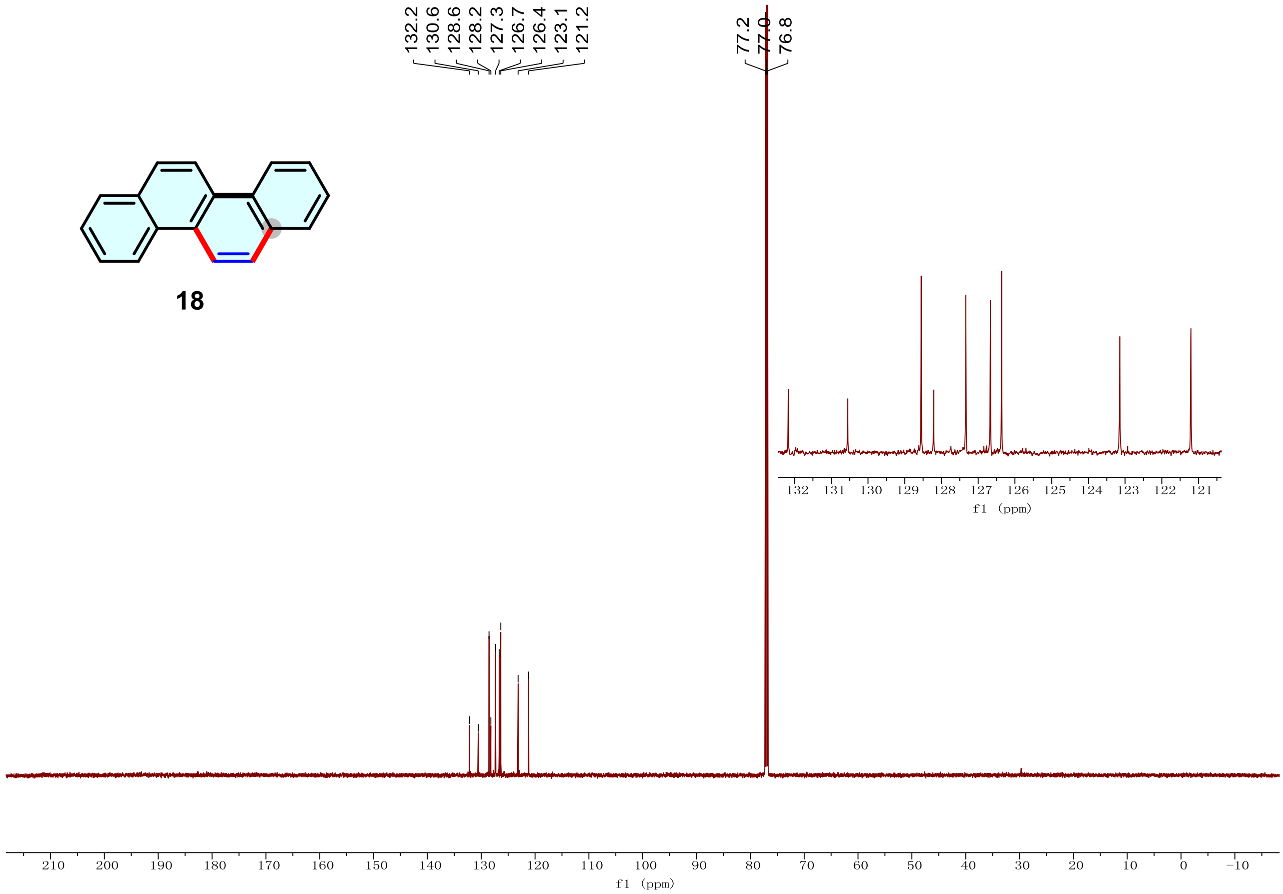

Supplementary Fig. 169. ^{1}H NMR of 19 (600 MHz, $CDCl_3$)

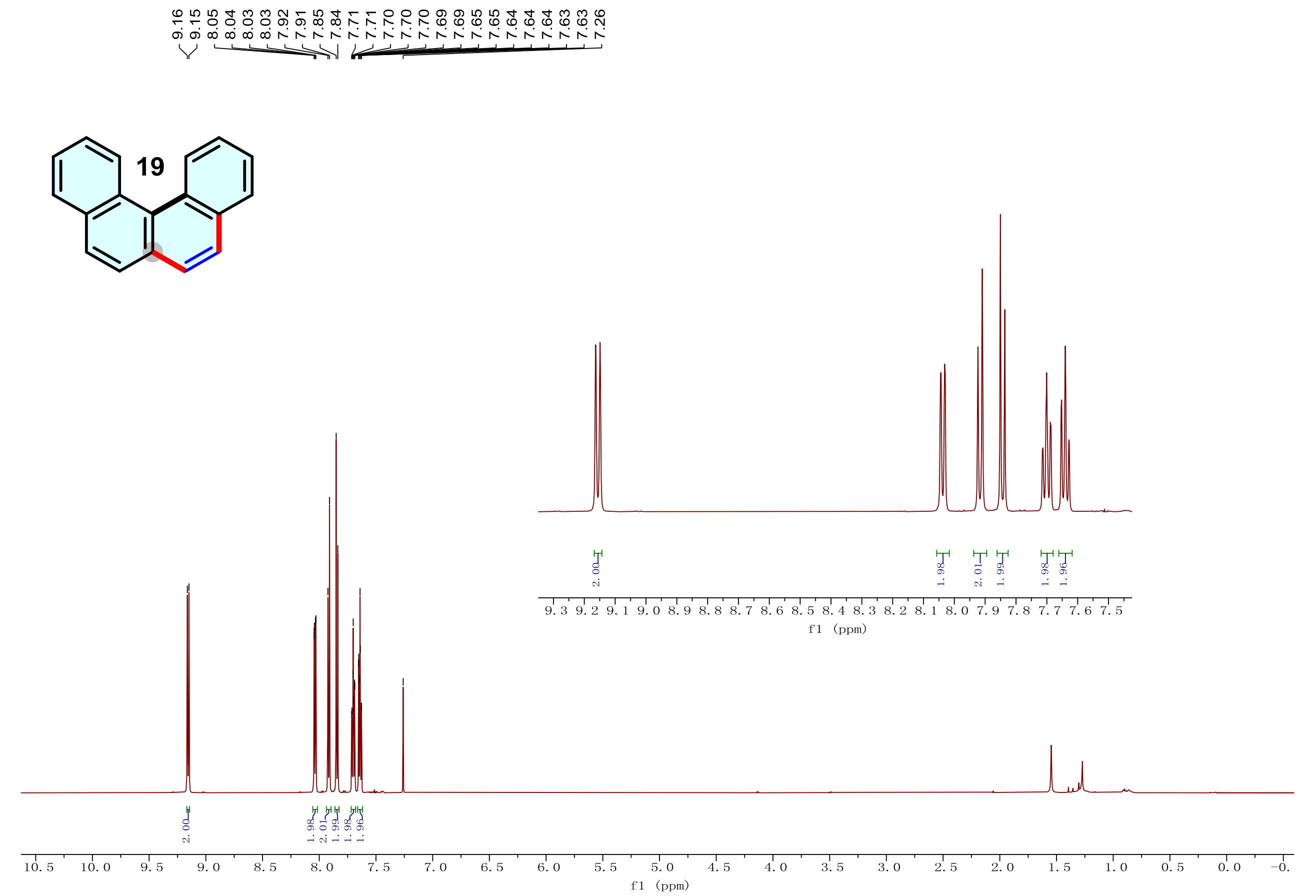

Supplementary Fig. 170. ^{13}C NMR of 19 (151 MHz, $CDCl_3$)

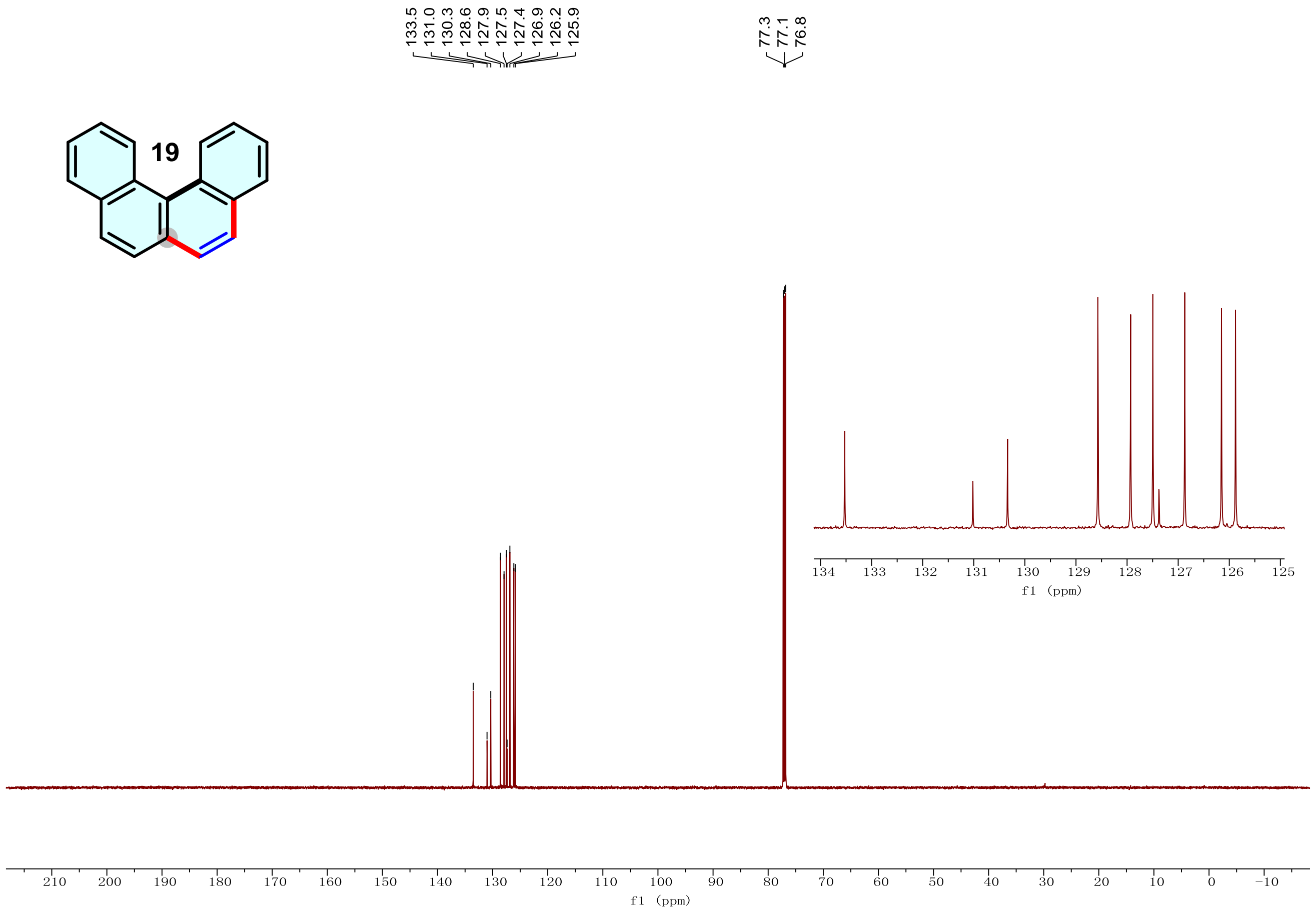

Supplementary Fig. 171. ^{1}H NMR of 20 (600 MHz, $CDCl_3$)

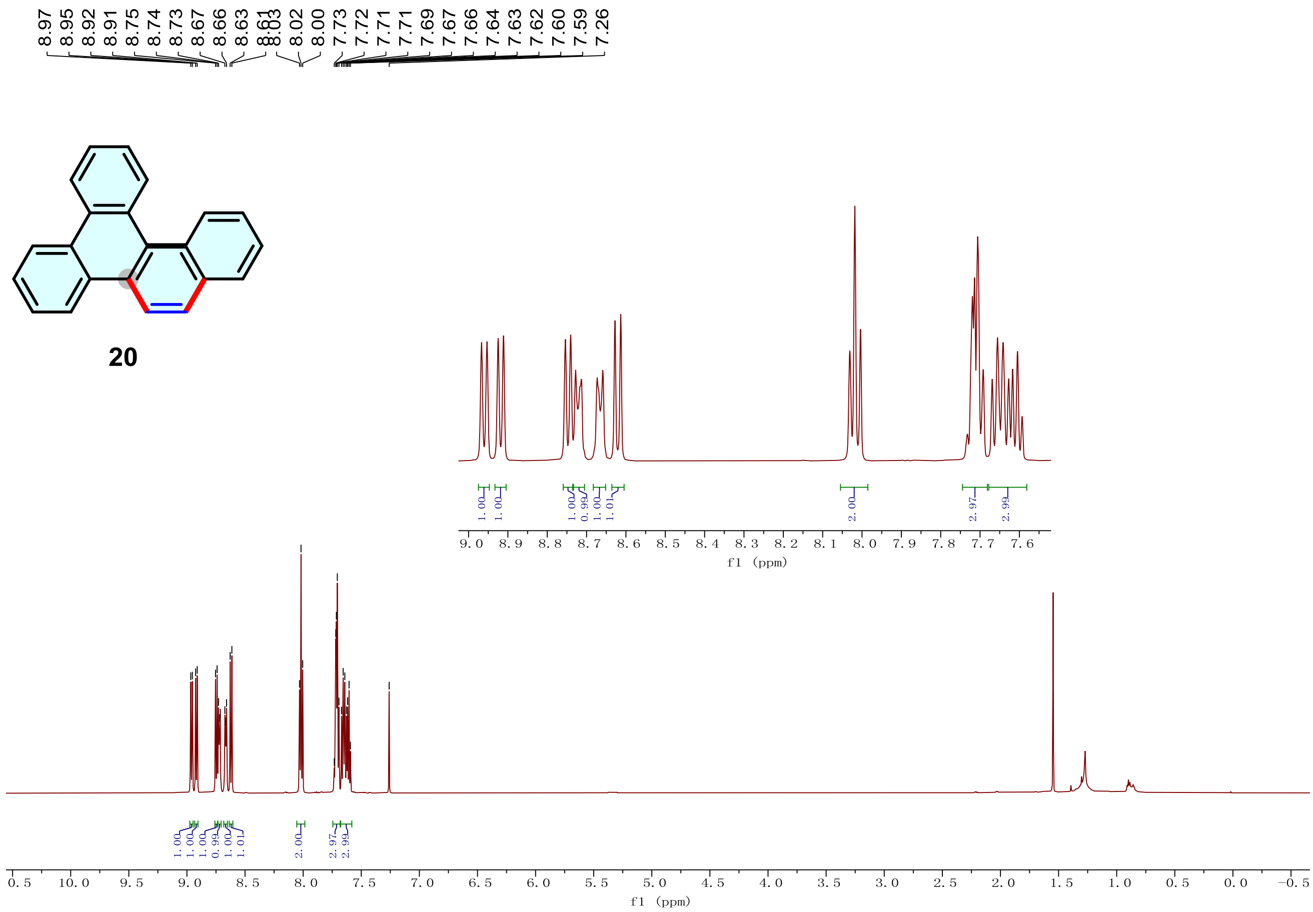

Supplementary Fig. 172. ^{13}C NMR of 20 (151 MHz, $CDCl_3$)

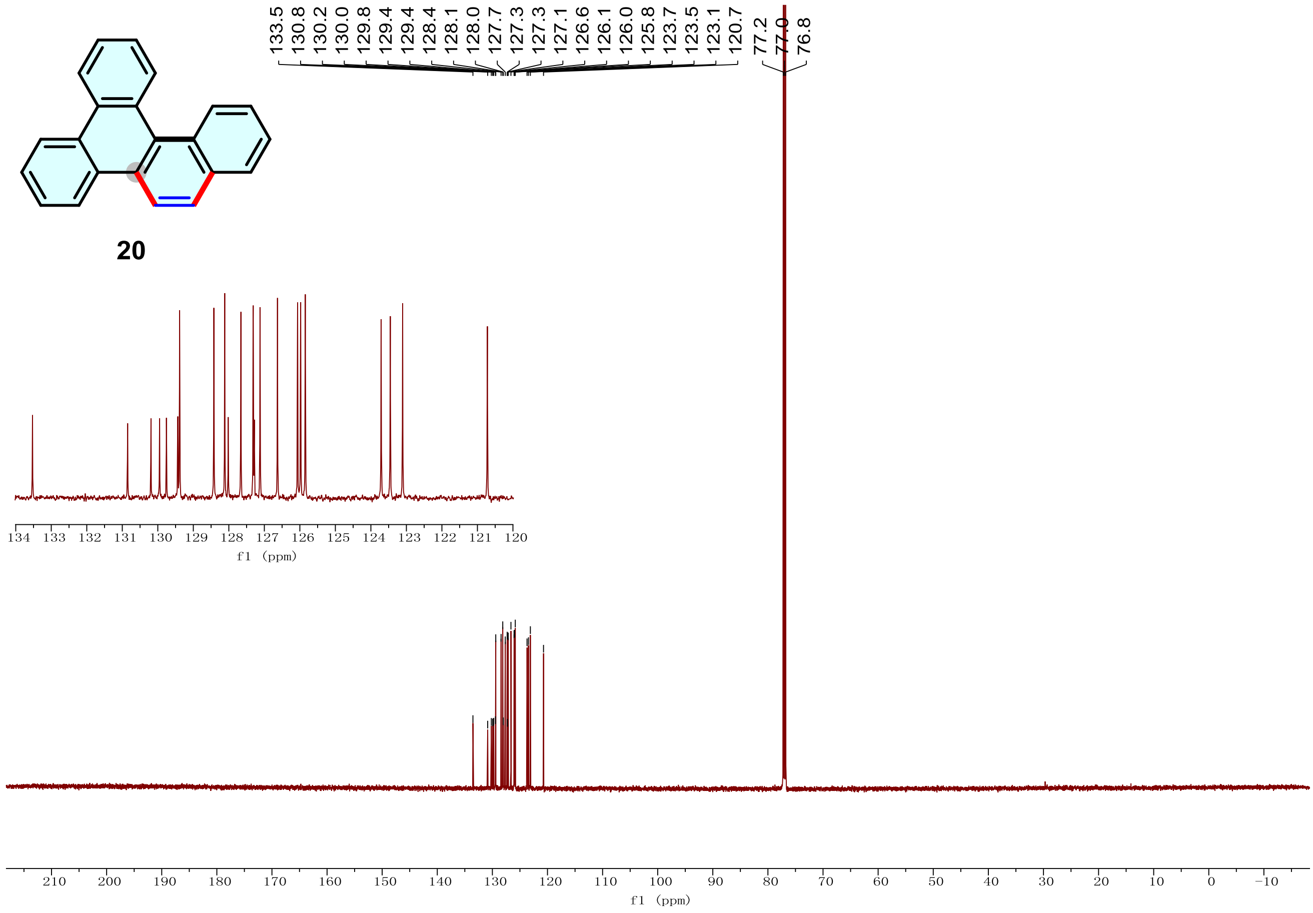

Supplementary Fig. 173. ^{1}H NMR of 21 (400 MHz, $CDCl_3$)

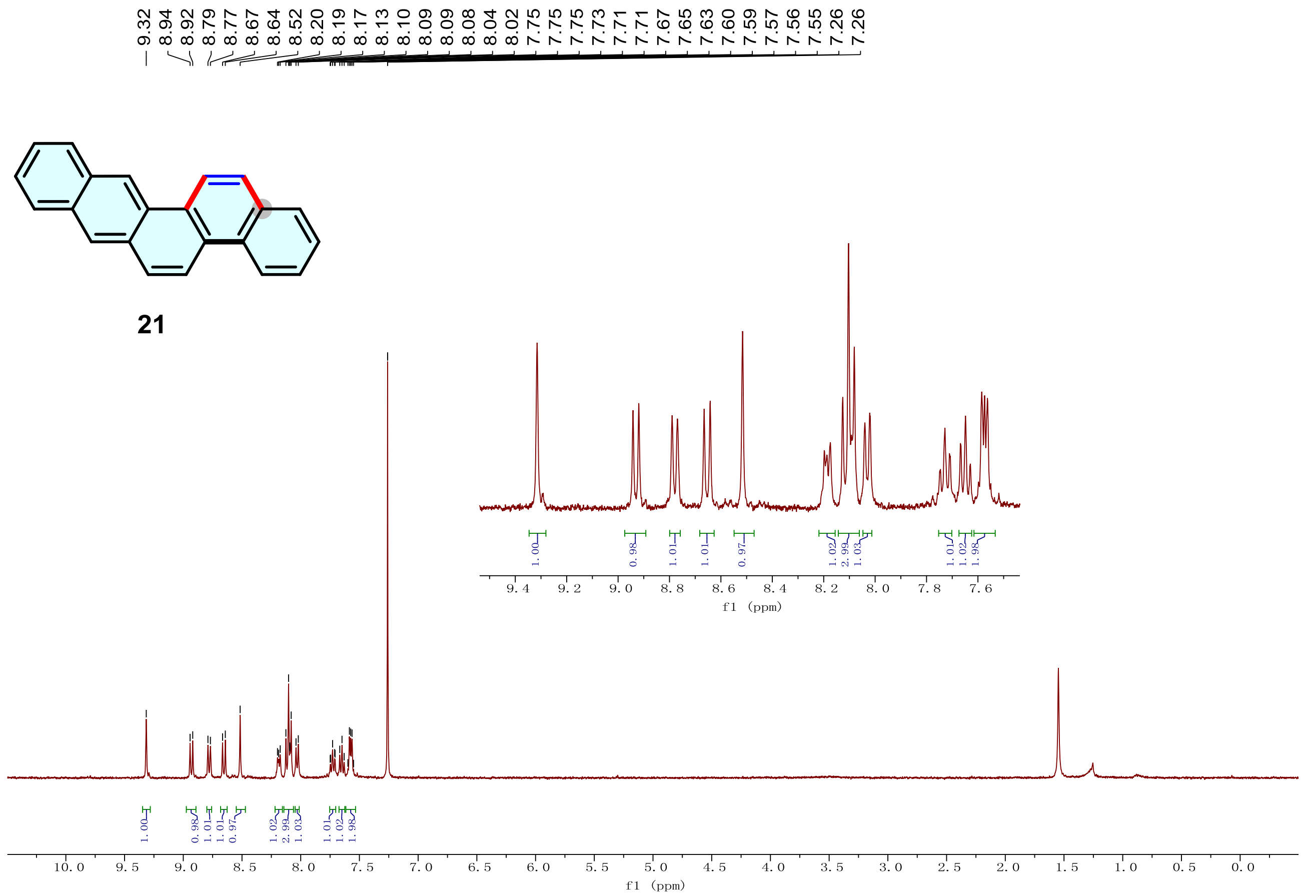

Supplementary Fig. 174. ^{13}C NMR of 21 (151 MHz, $CDCl_3$)

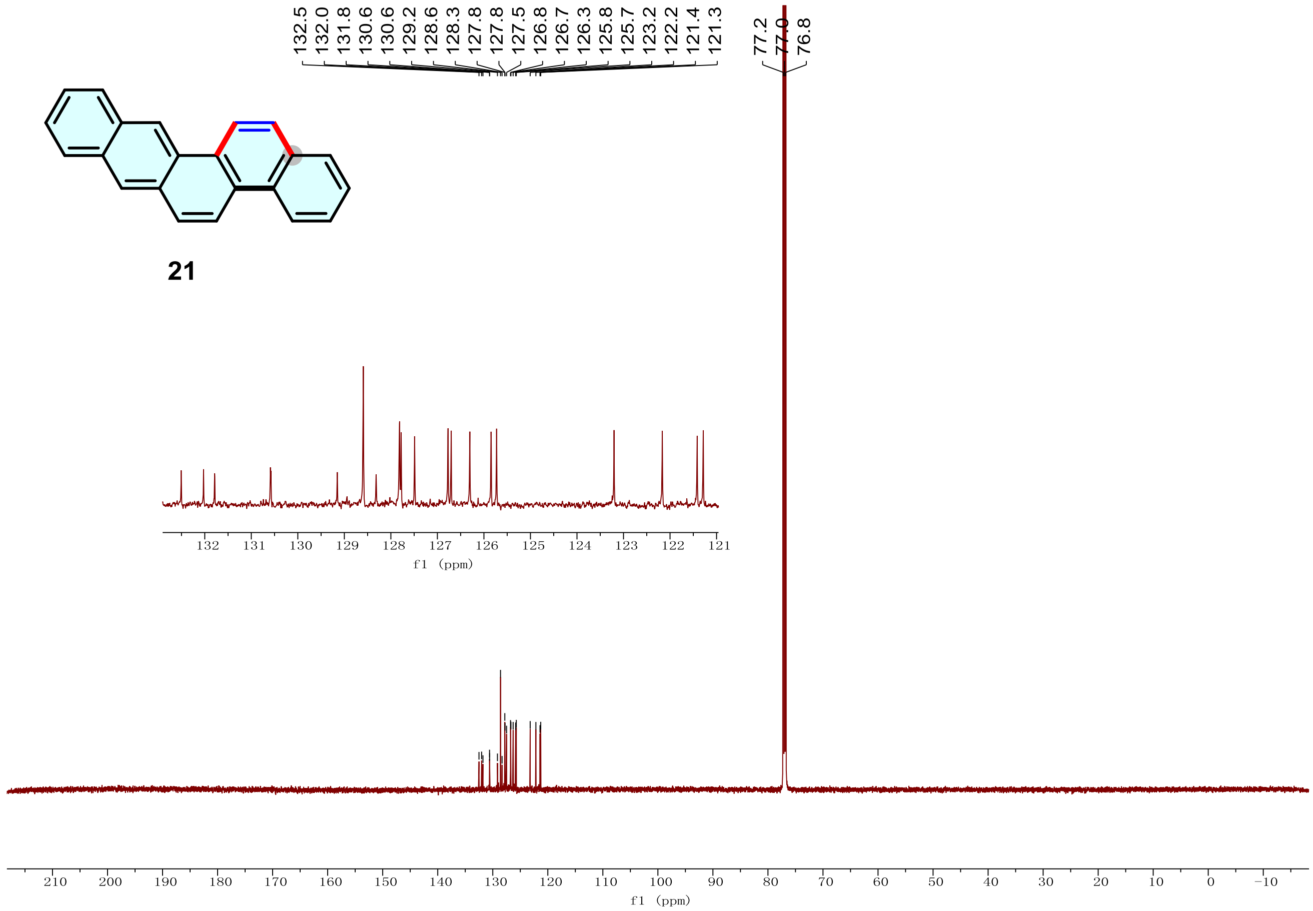

Supplementary Fig. 175. ^{1}H NMR of 22 (400 MHz, $CDCl_3$)

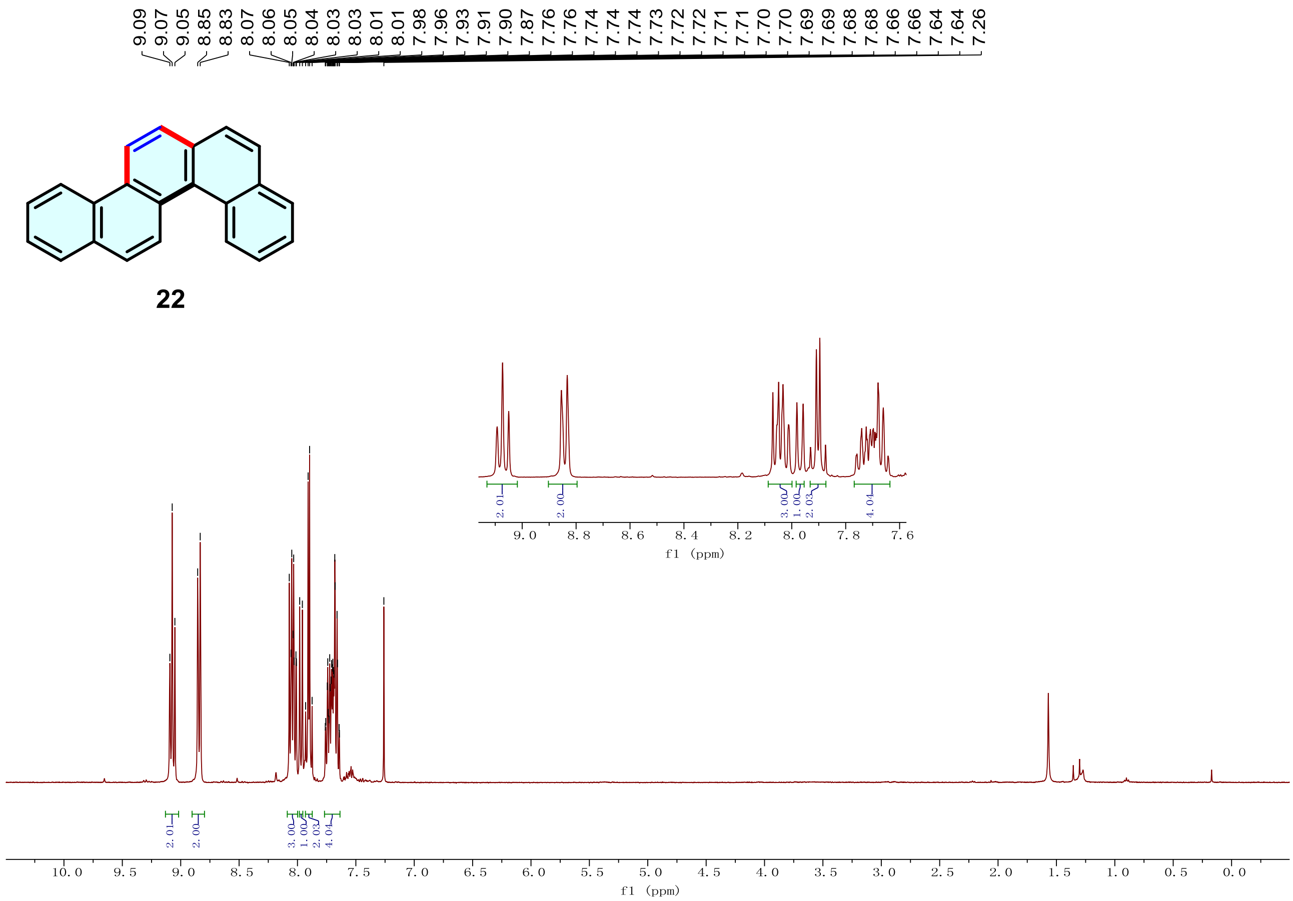

Supplementary Fig. 176. ^{13}C NMR of 22 (101 MHz, $CDCl_3$)

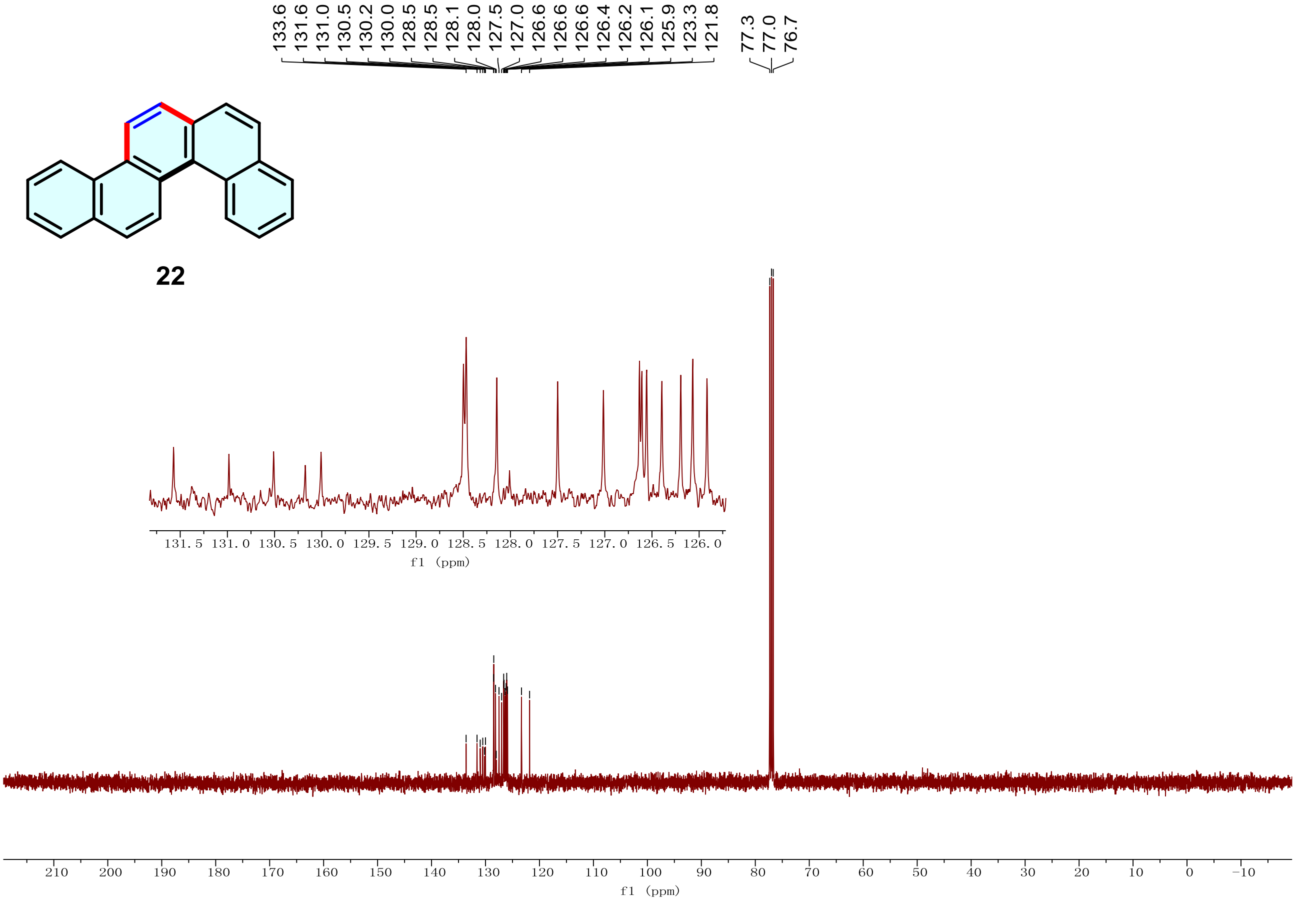

Supplementary Fig. 177. ^{1}H NMR of 23 (400 MHz, CDCl$_3$)

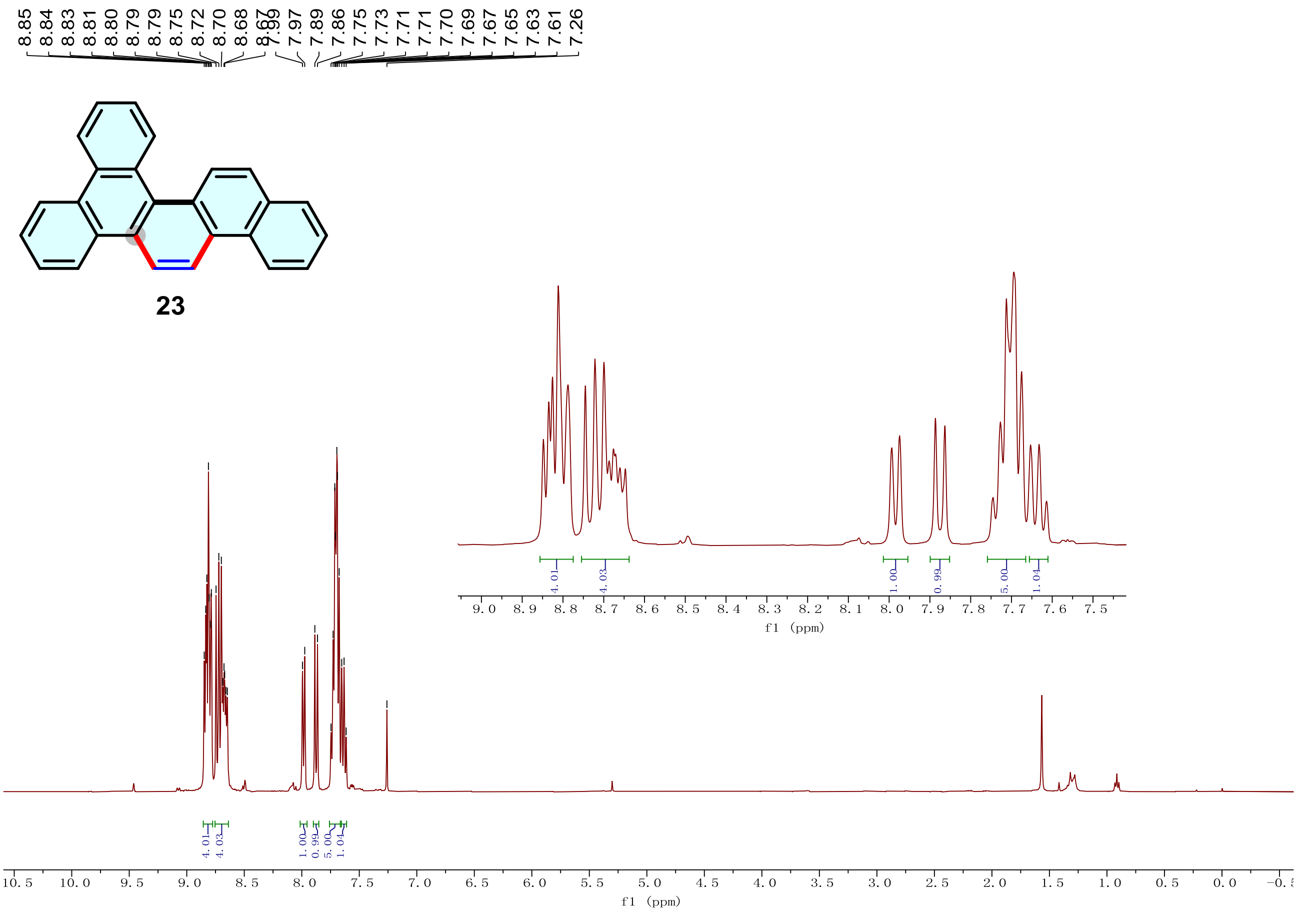

Supplementary Fig. 178. ^{13}C NMR of 23 (101 MHz, CDCl$_3$)

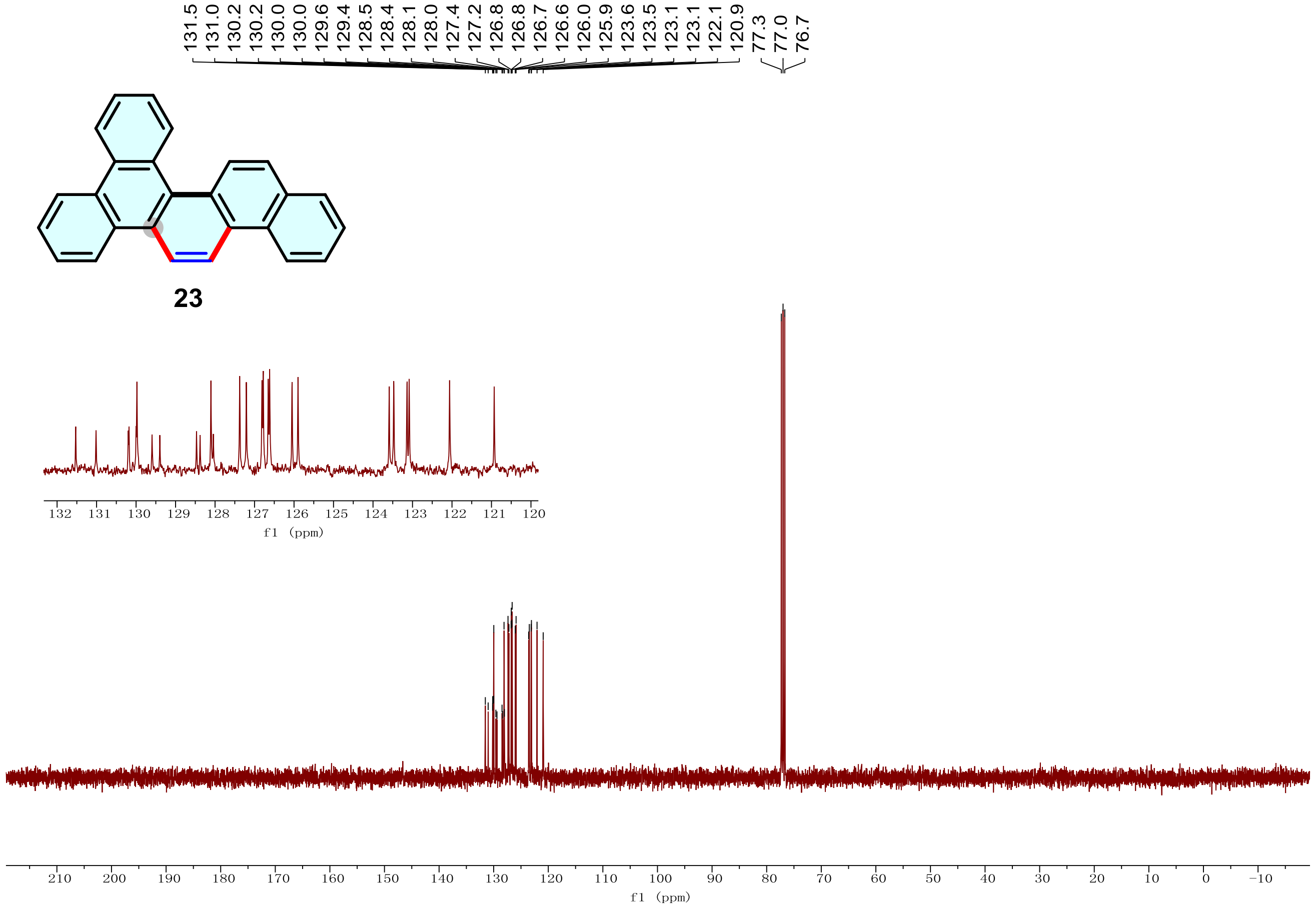

Supplementary Fig. 179. ^{1}H NMR of 25 (400 MHz, $CDCl_3$)

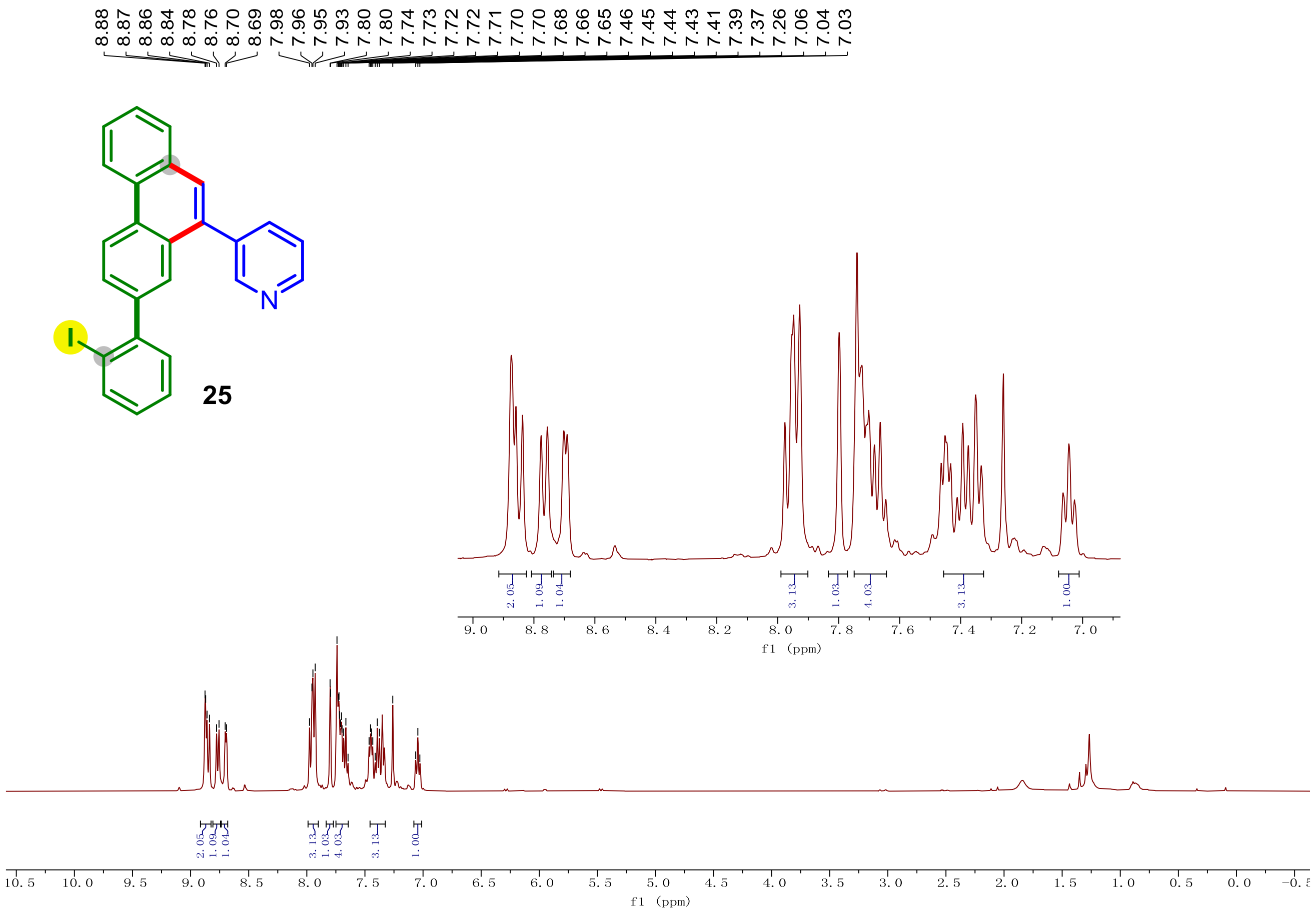

Supplementary Fig. 180. ^{13}C NMR of 25 (101 MHz, $CDCl_3$)

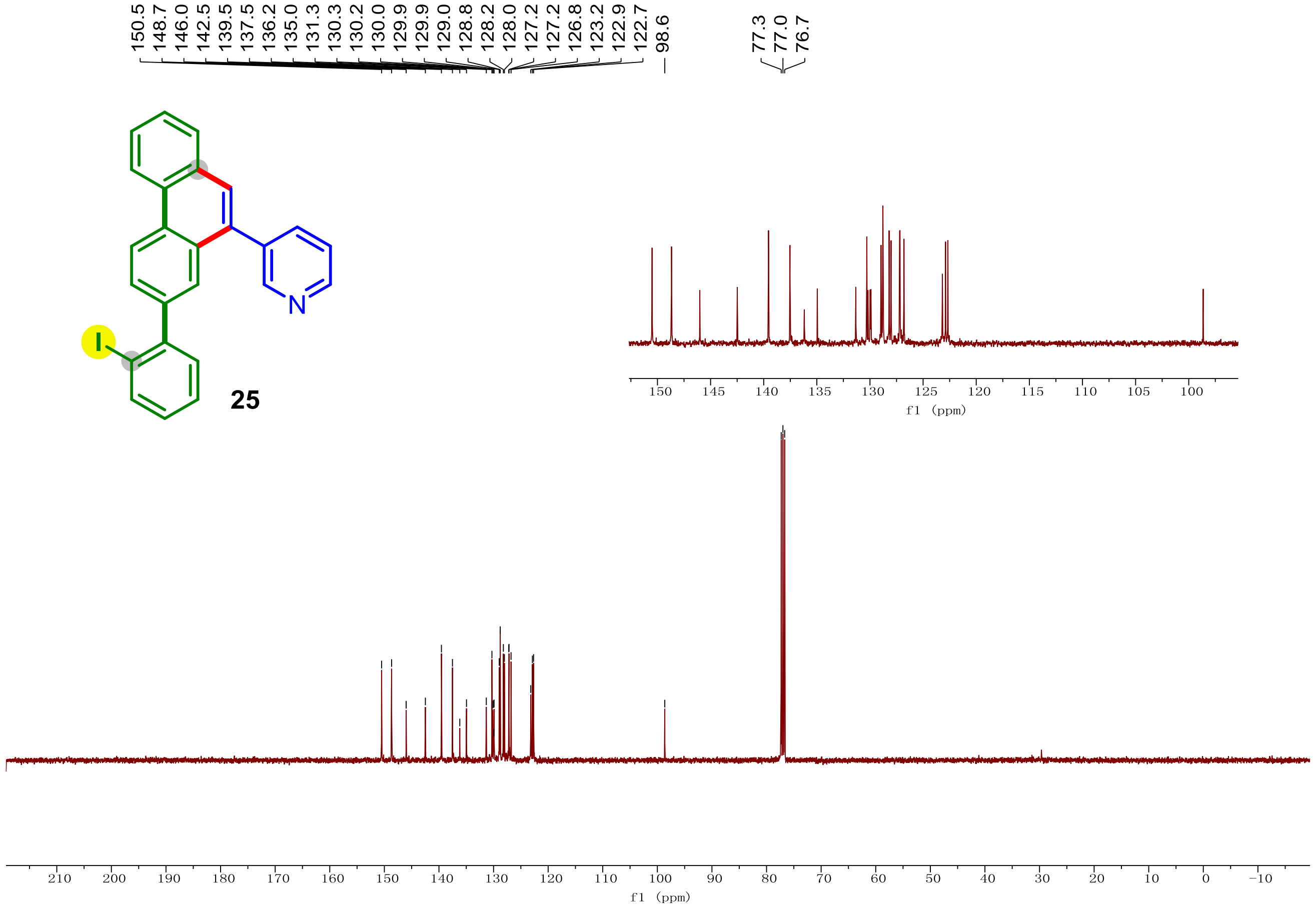

Supplementary Fig. 181. ^{1}H NMR of 26 (400 MHz, CDCl3)

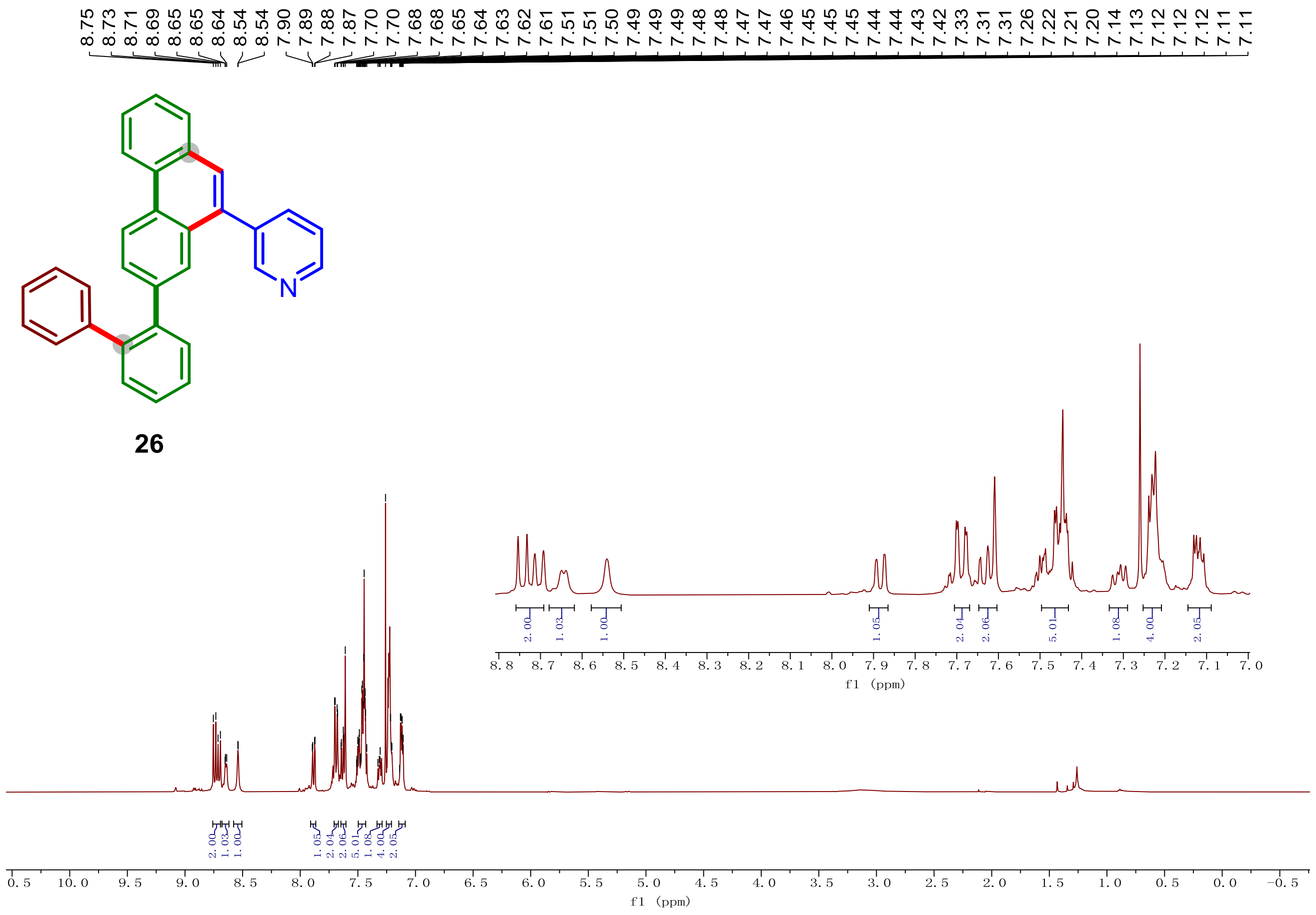

Supplementary Fig. 182. ^{13}C NMR of 26 (101 MHz, CDCl3)

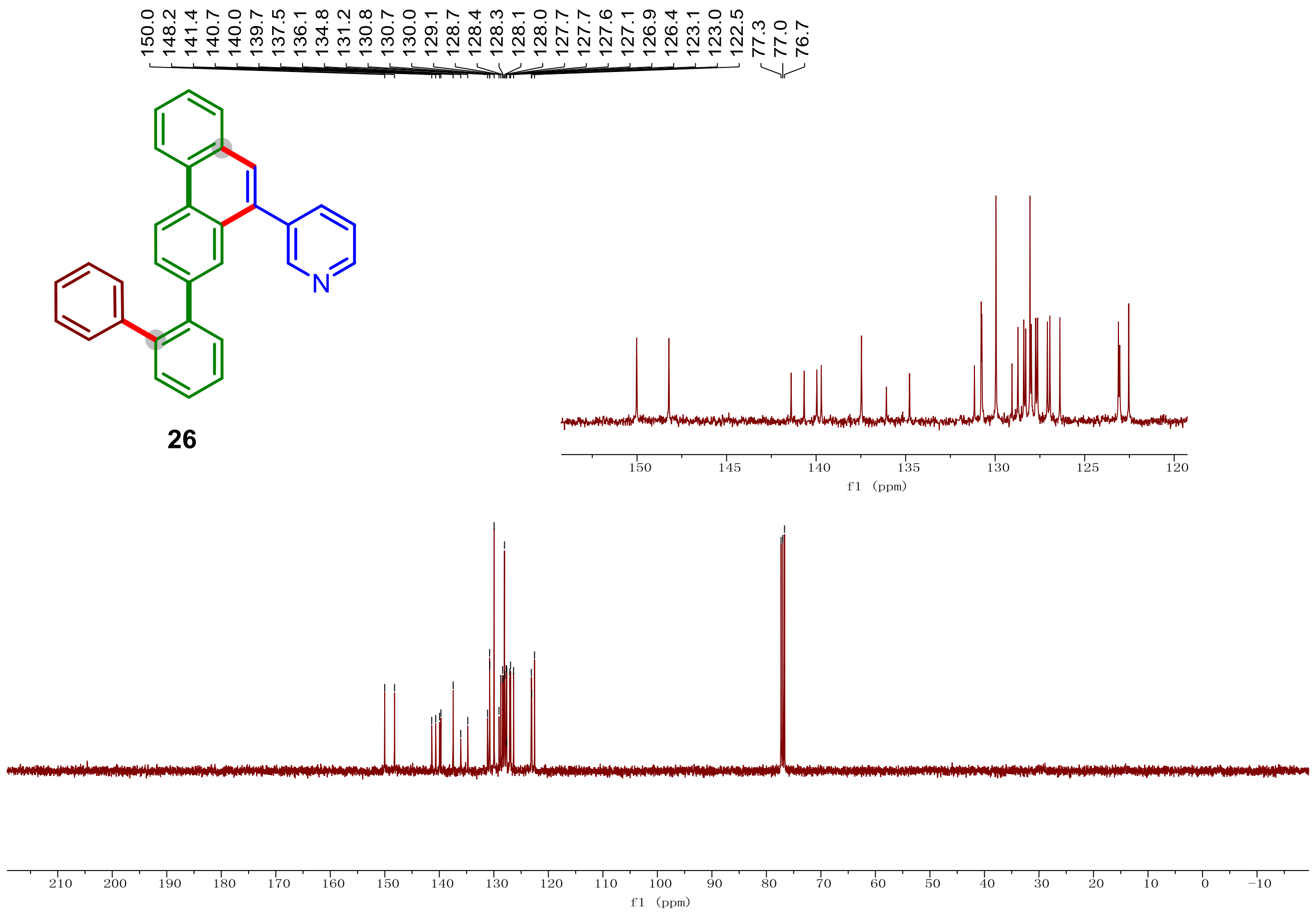

Supplementary Fig. 183. ^{1}H NMR of 27 (400 MHz, $CDCl_3$)

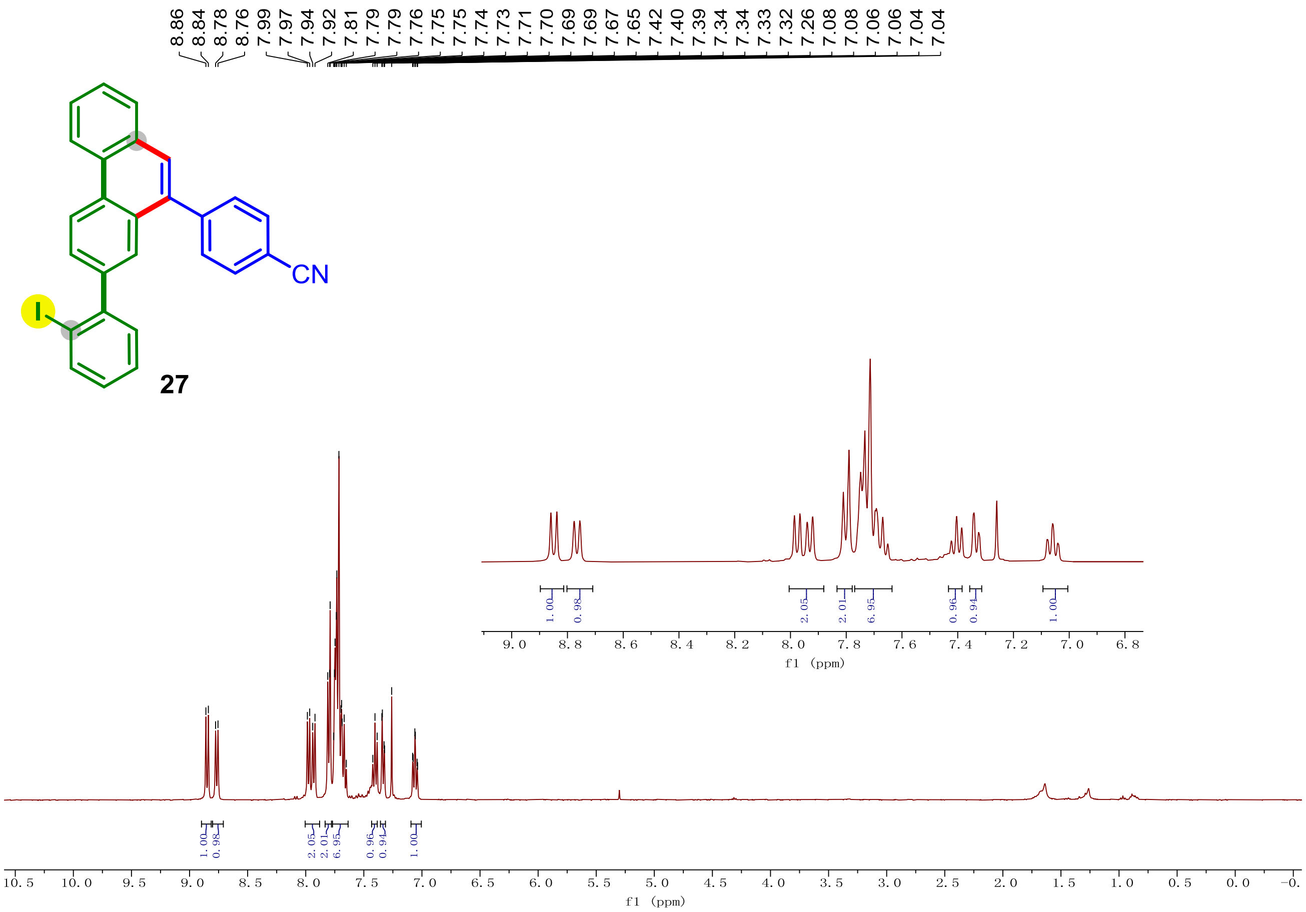

Supplementary Fig. 184. ^{13}C NMR of 27 (101 MHz, $CDCl_3$)

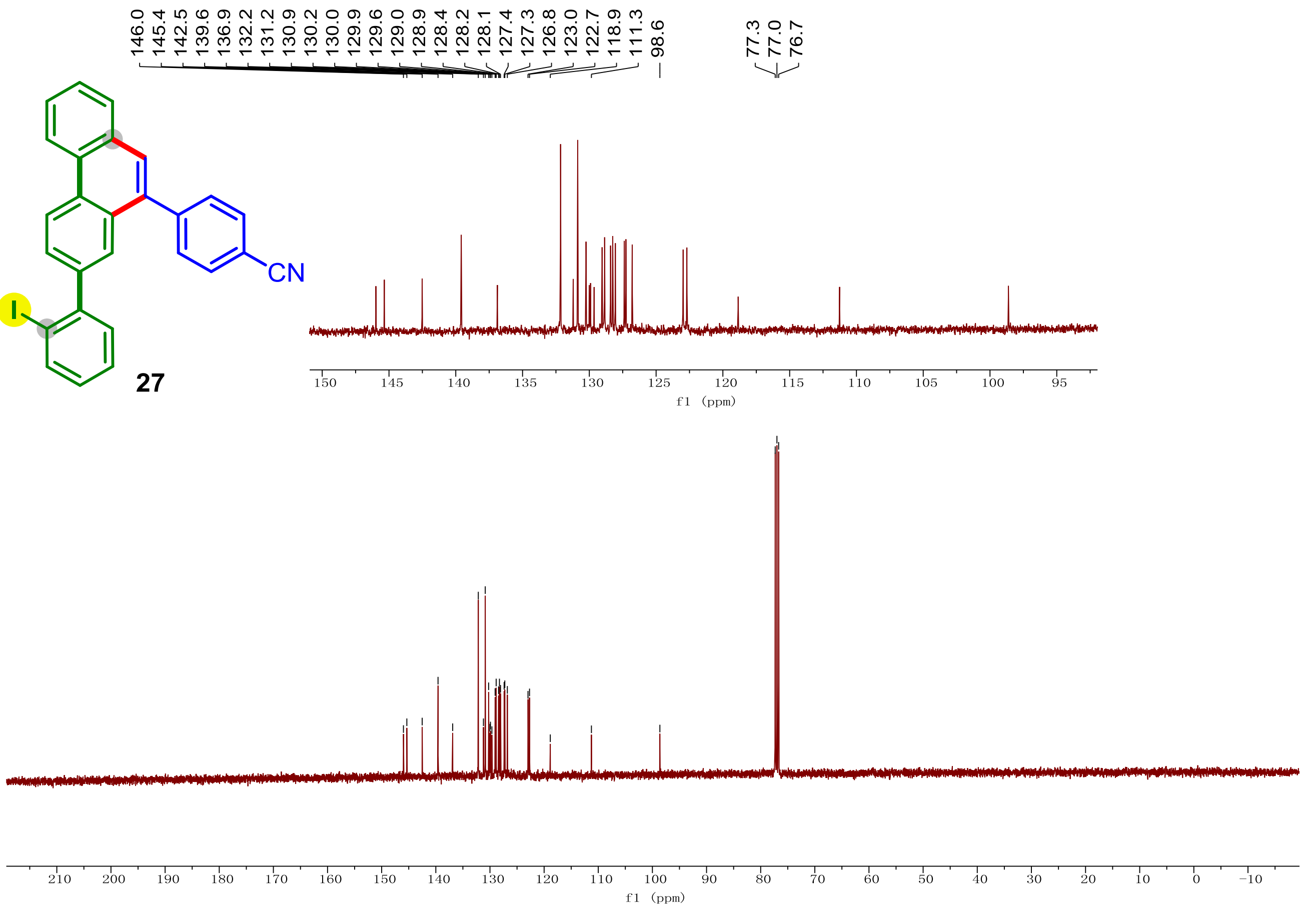

Supplementary Fig. 185. ^{1}H NMR of 28 (400 MHz, $CDCl_3$)

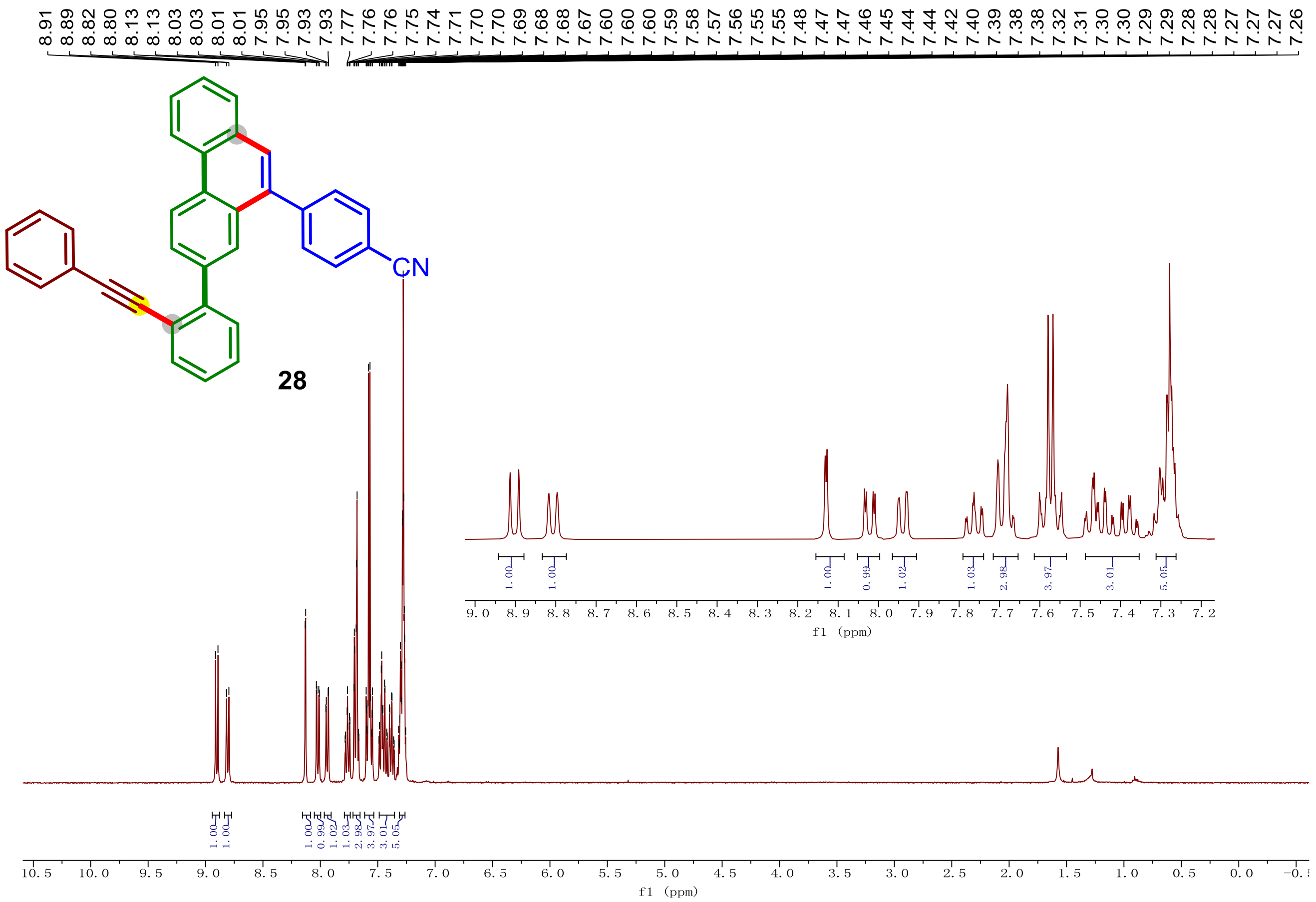

Supplementary Fig. 186. ^{13}C NMR of 28 (101 MHz, $CDCl_3$)

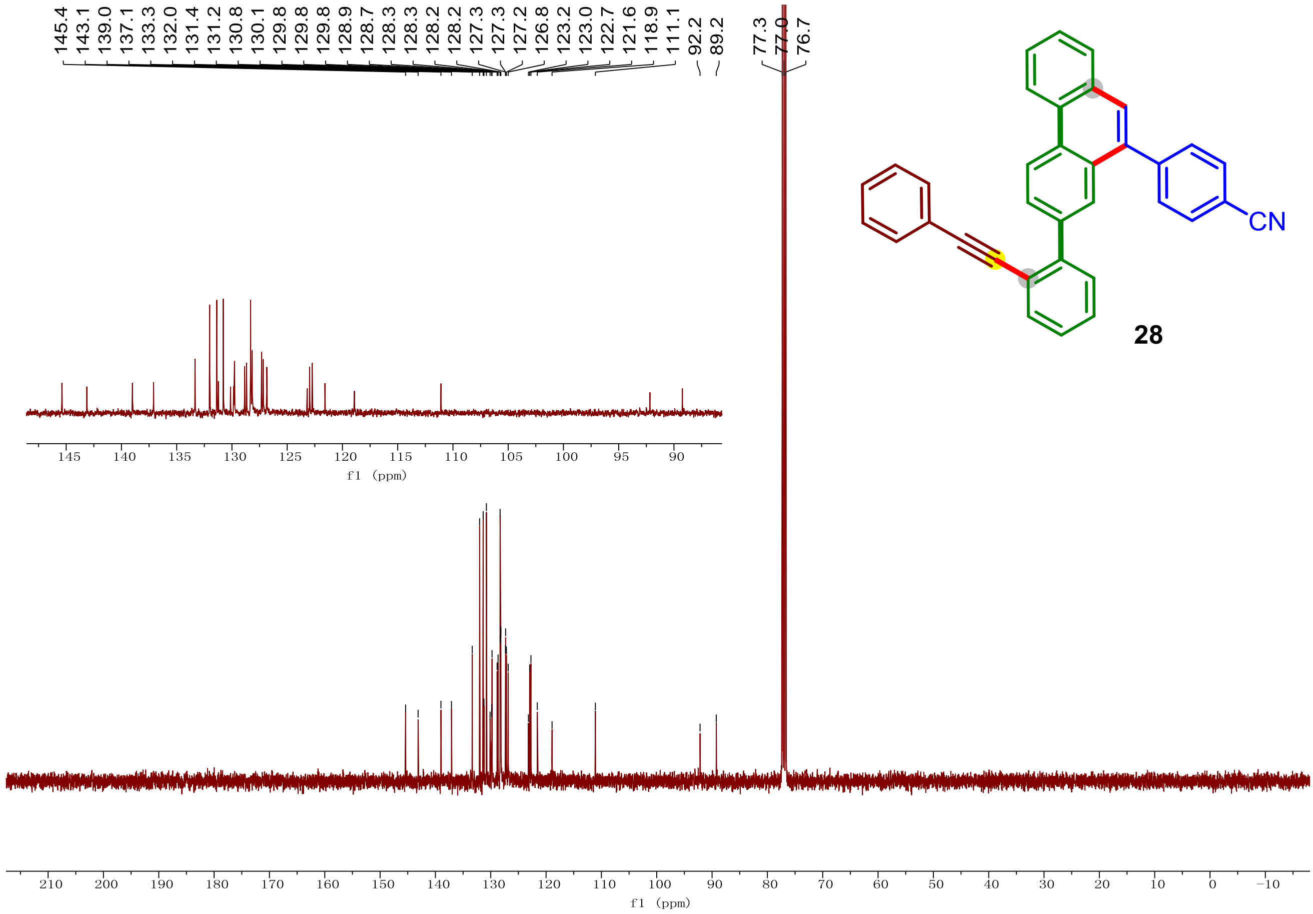

Supplementary Fig. 187. ¹H NMR of 35 (600 MHz, $CDCl_3$)

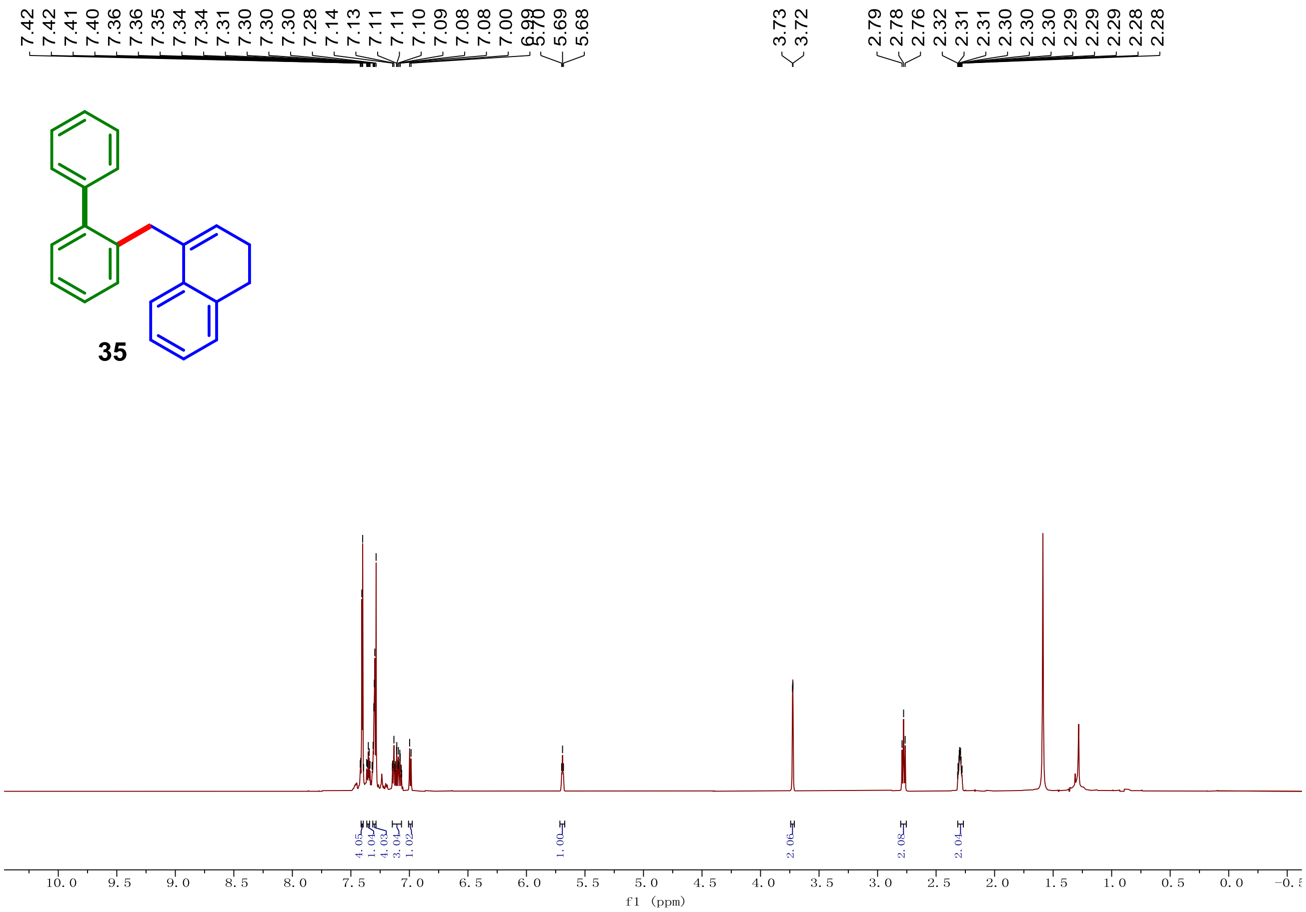

Supplementary Fig. 188. ¹³C NMR of 35 (151 MHz, $CDCl_3$)

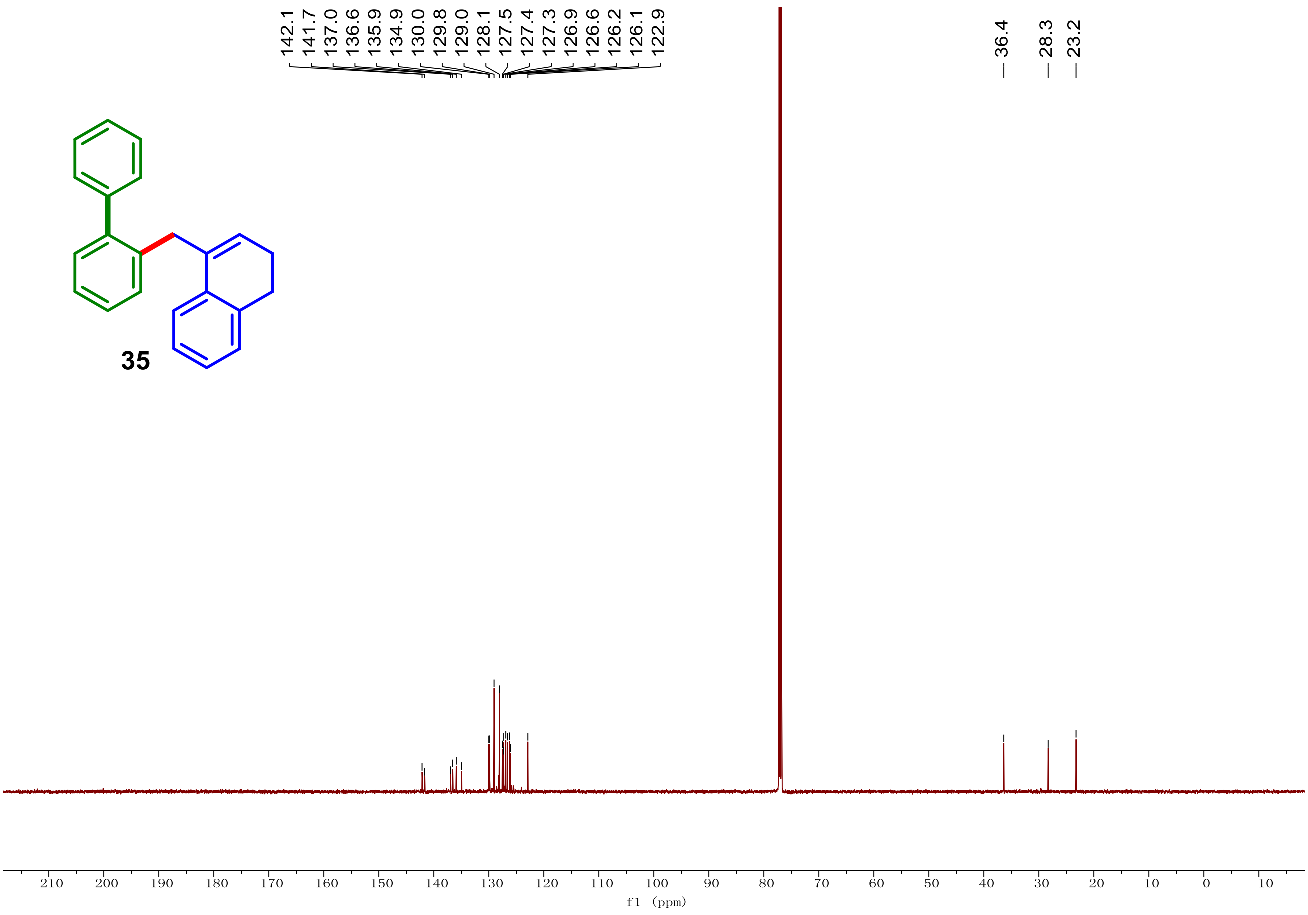

Supplementary Fig. 189. ^{1}H NMR of 36 (400 MHz, $CDCl_3$)

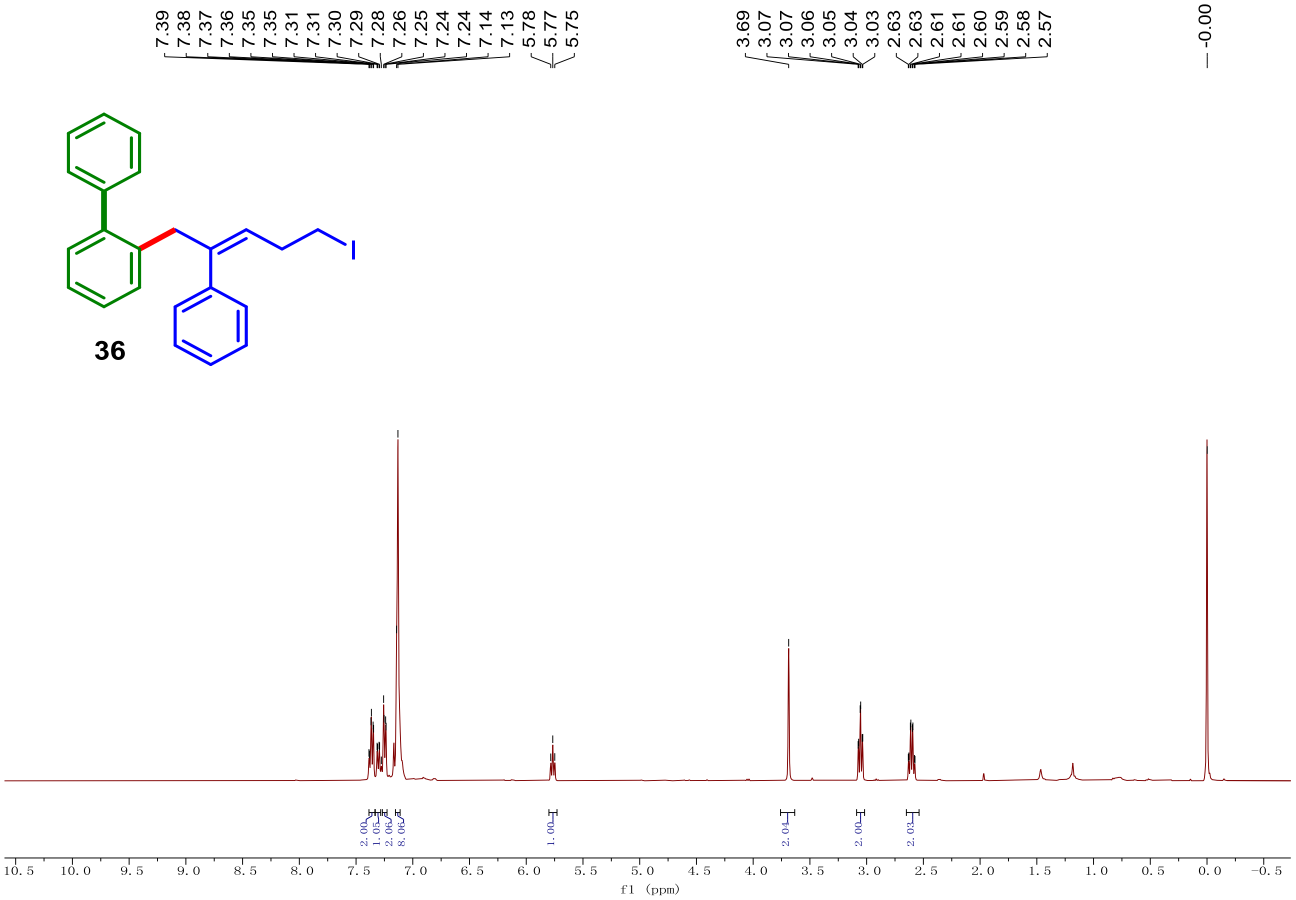

Supplementary Fig. 190. ^{13}C NMR of 36 (101 MHz, $CDCl_3$)

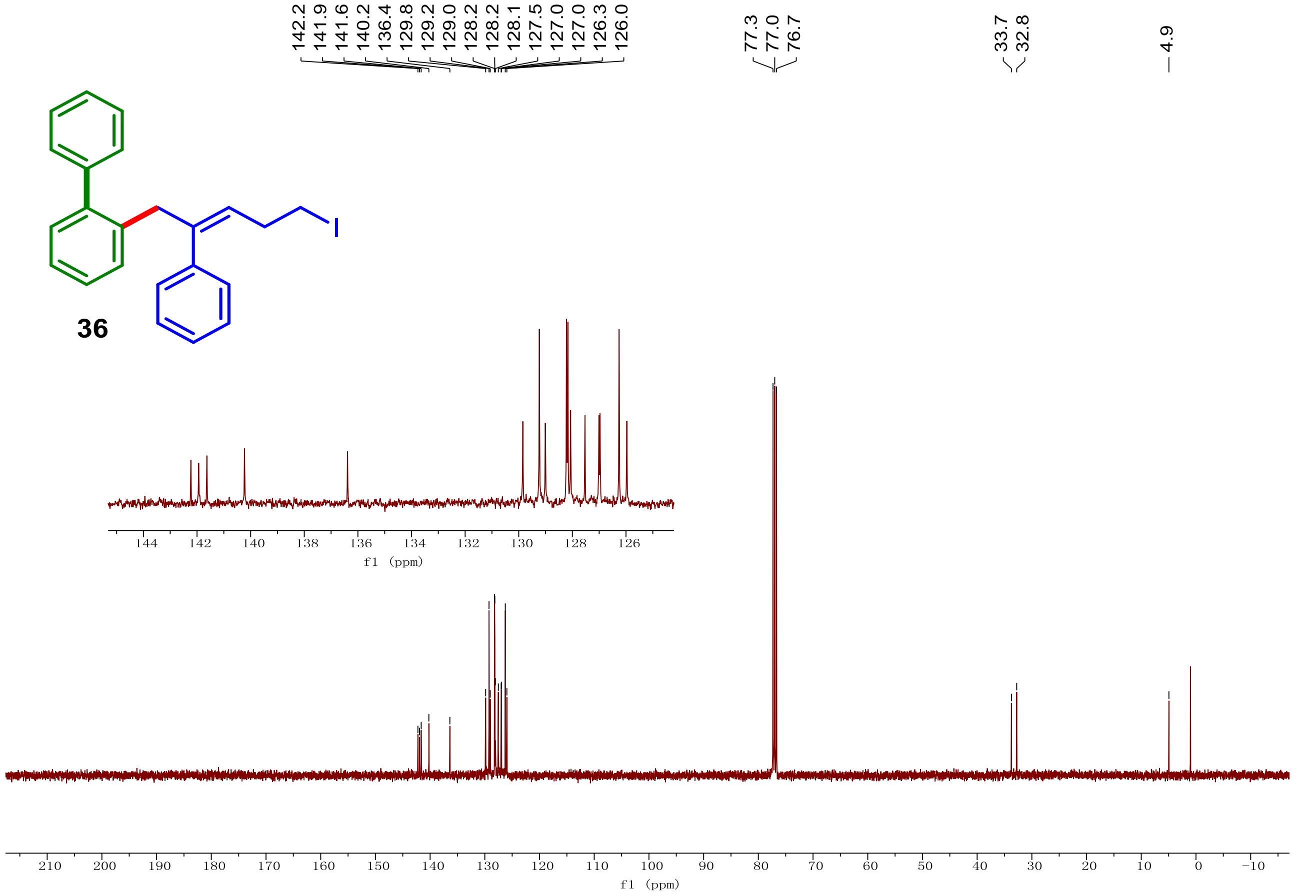

Supplementary Fig. 191. ^{1}H NMR of 3a-D^4 (400 MHz, $CDCl_3$)

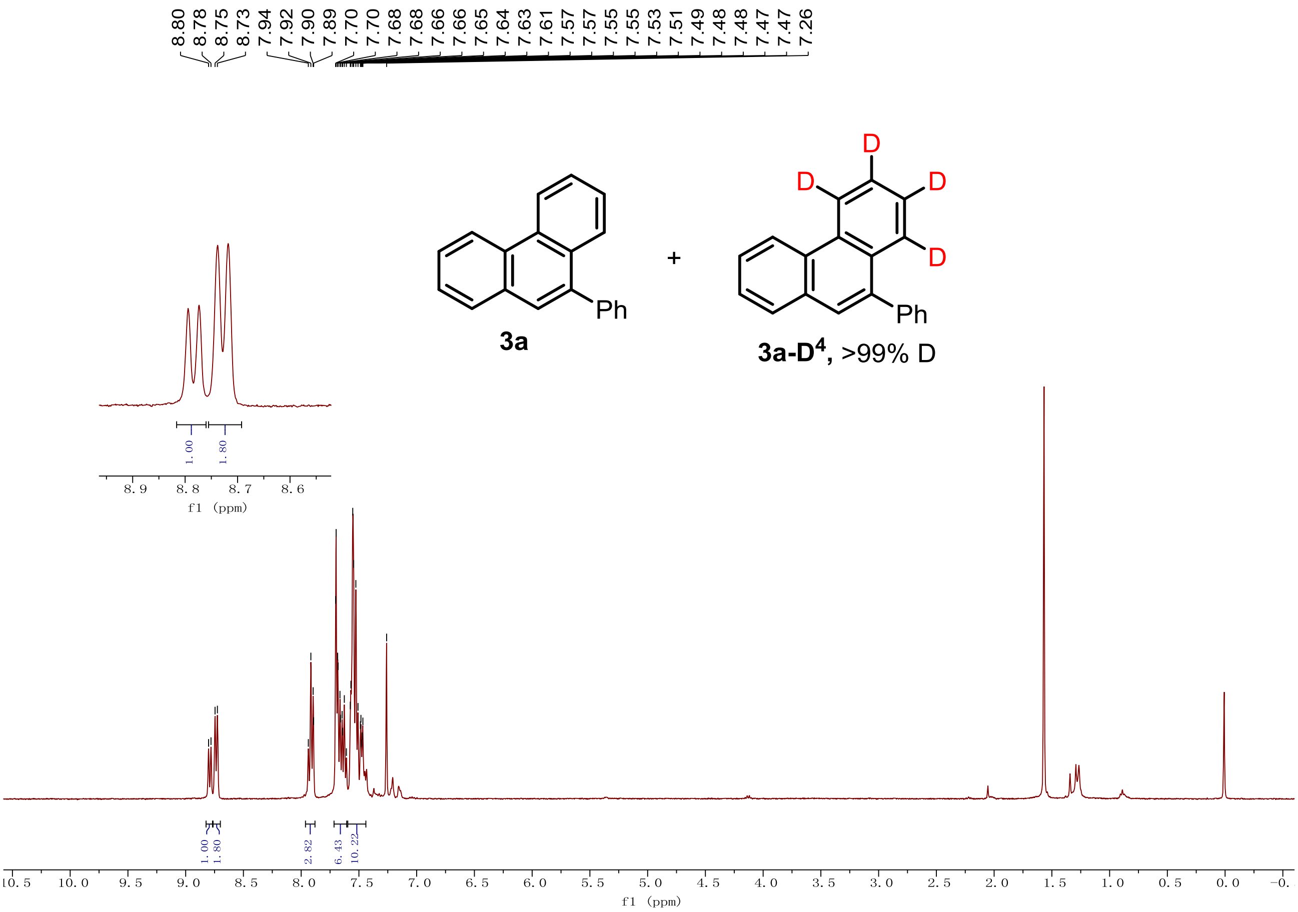

Supplementary Fig. 192. ^{13}C NMR of 3a-D^4 (101 MHz, $CDCl_3$)

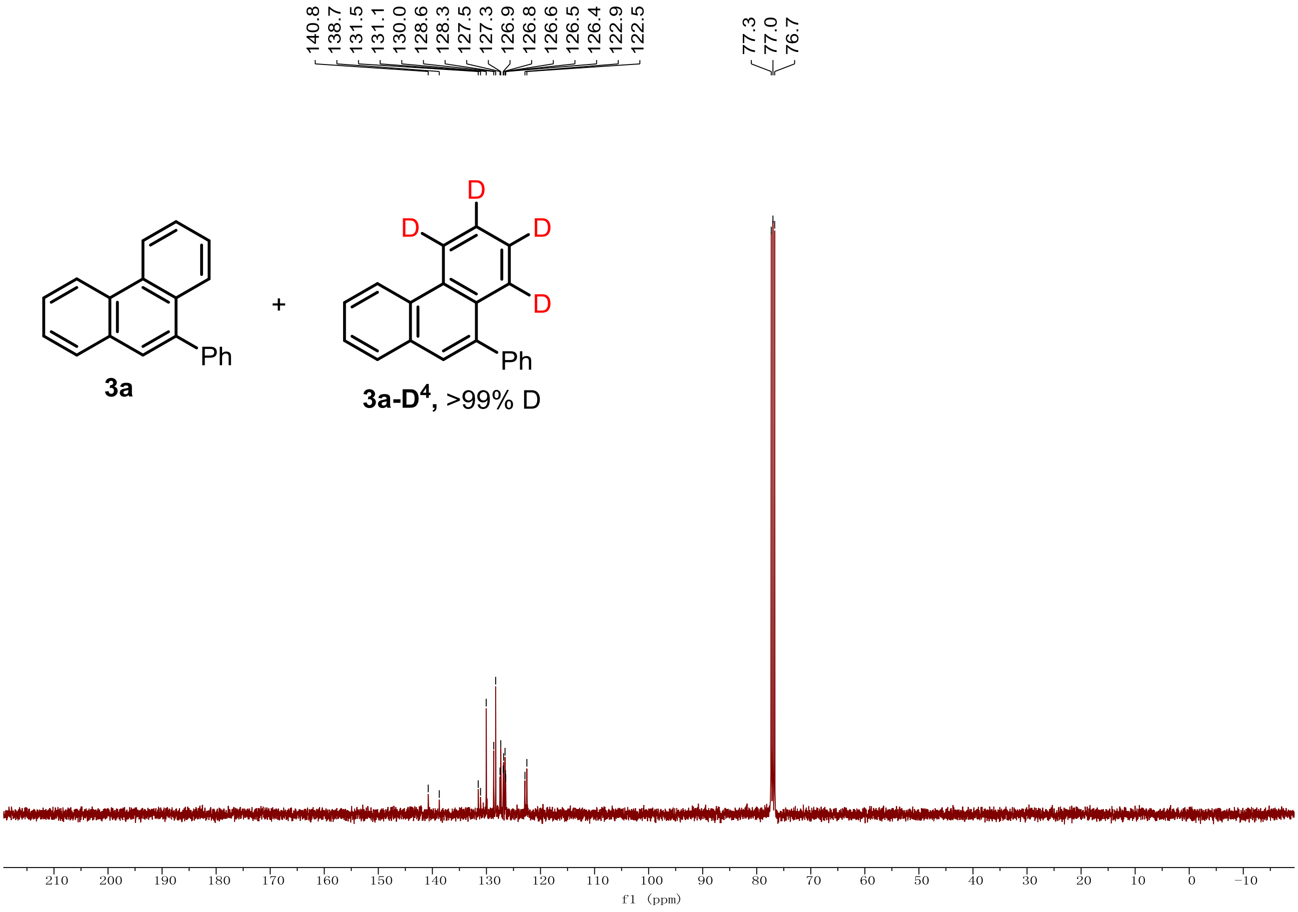

Supplementary Fig. 193. ^{1}H NMR of 3a-D^{4} (400 MHz, $CDCl_3$)

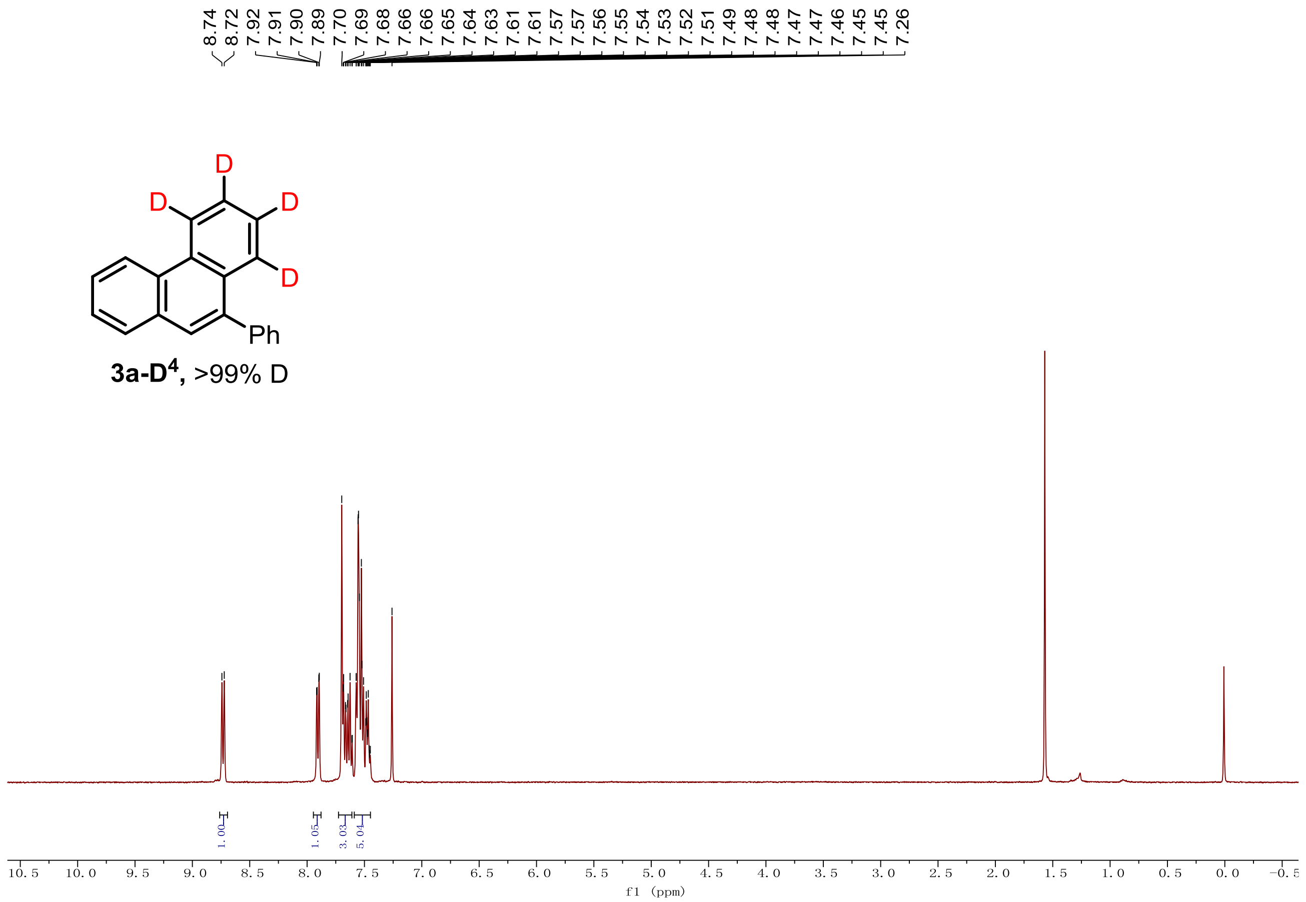

Supplementary Fig. 194. ^{13}C NMR of 3a-D^{4} (101 MHz, $CDCl_3$)

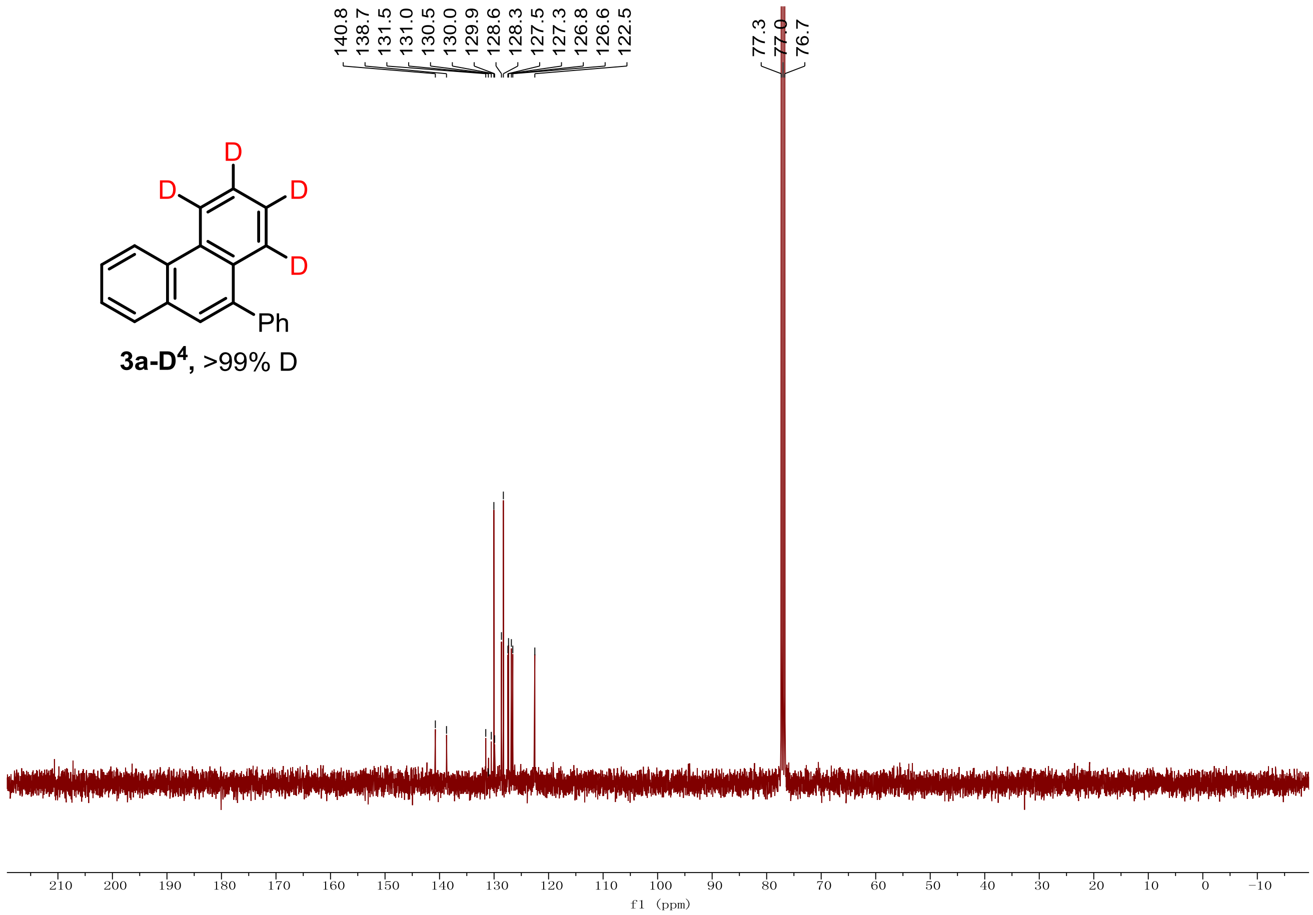

Supplementary Fig. 195. ^{1}H **NMR of 1a-D (400 MHz, CDCl**$_3$**)**

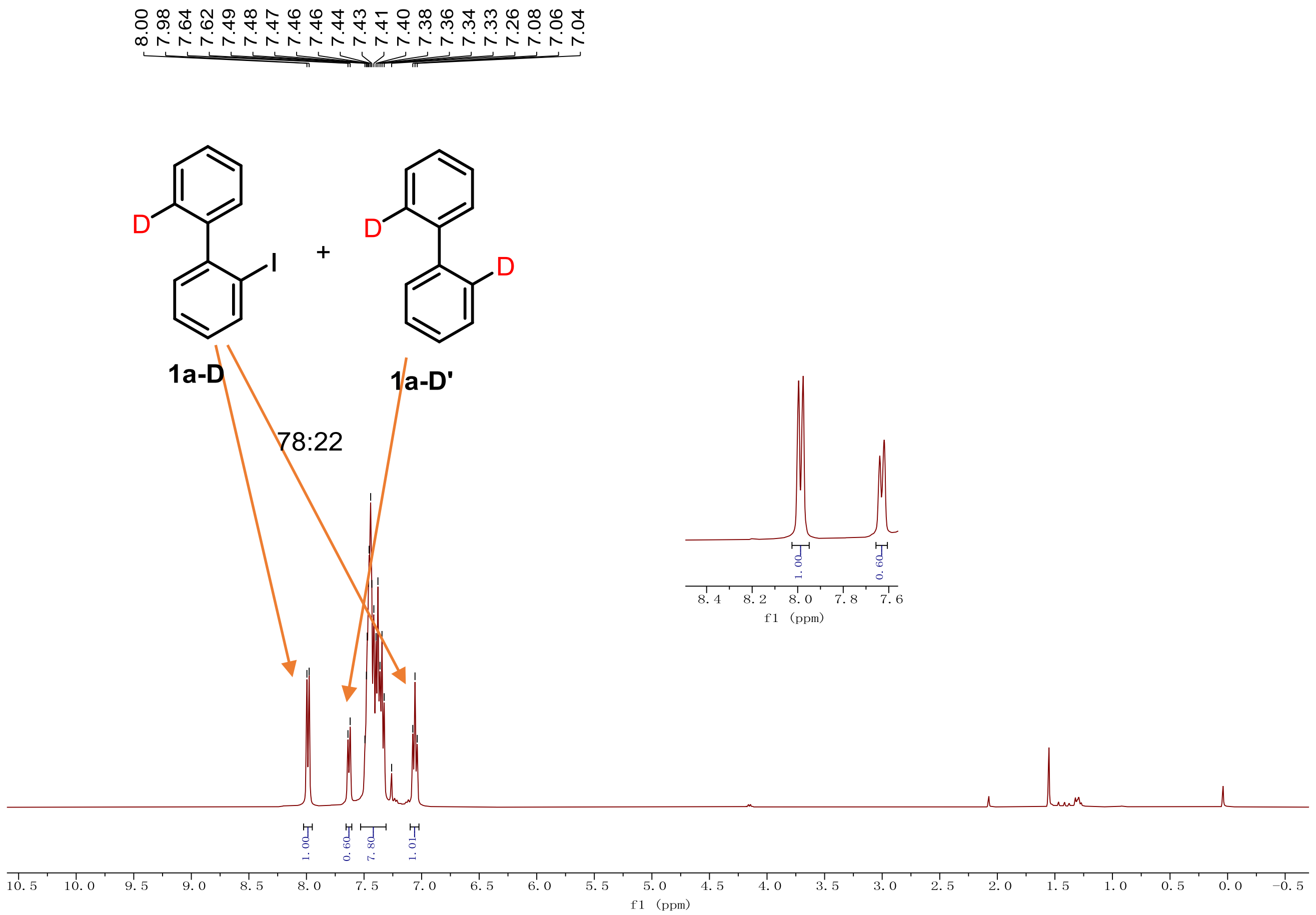

Supplementary Fig. 196. ^{13}C **NMR of 1a-D (101 MHz, CDCl**$_3$**)**

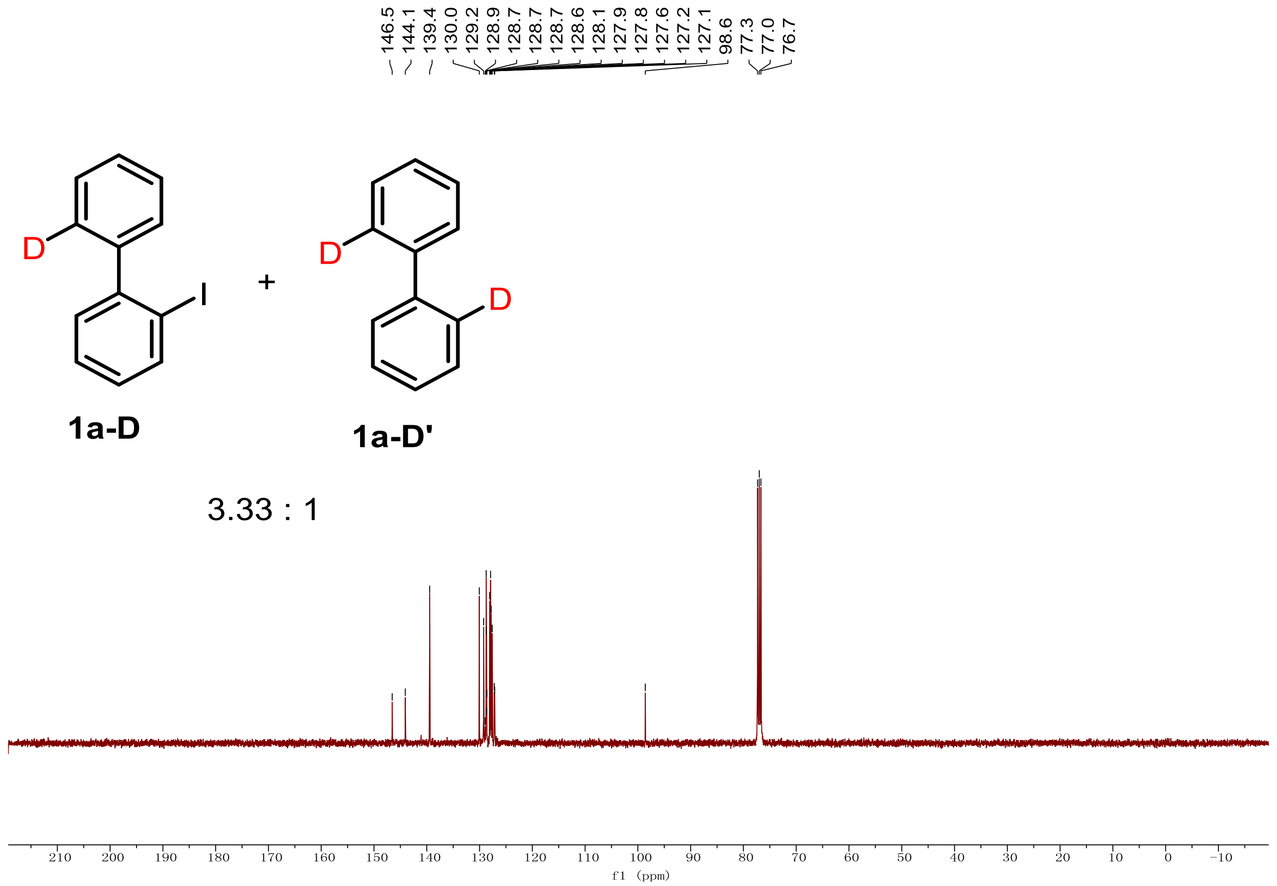

Supplementary Fig. 197. ^{1}H NMR (400 MHz, $CDCl_3$)

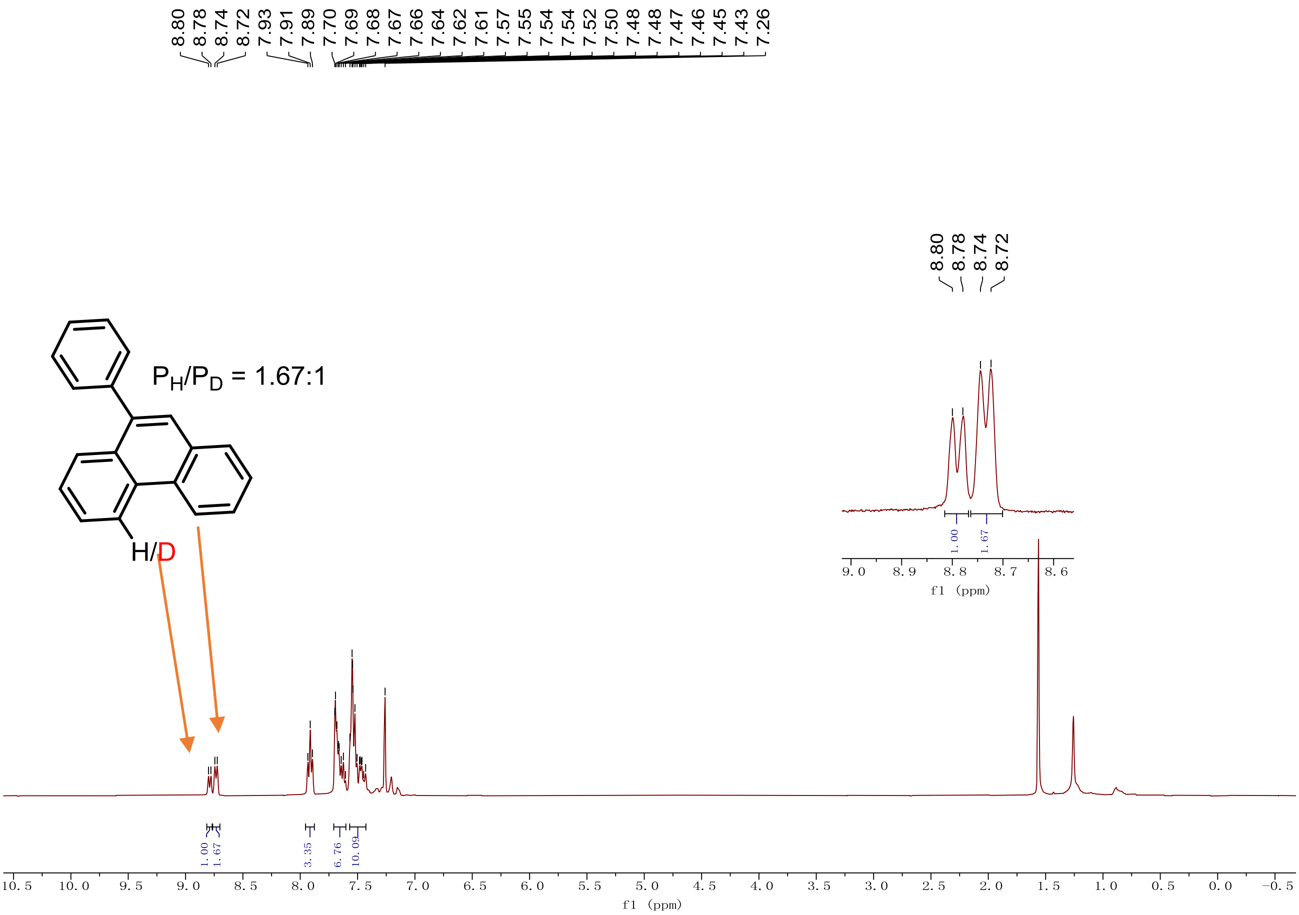

Supplementary Fig. 198. ^{13}C NMR (101 MHz, $CDCl_3$)

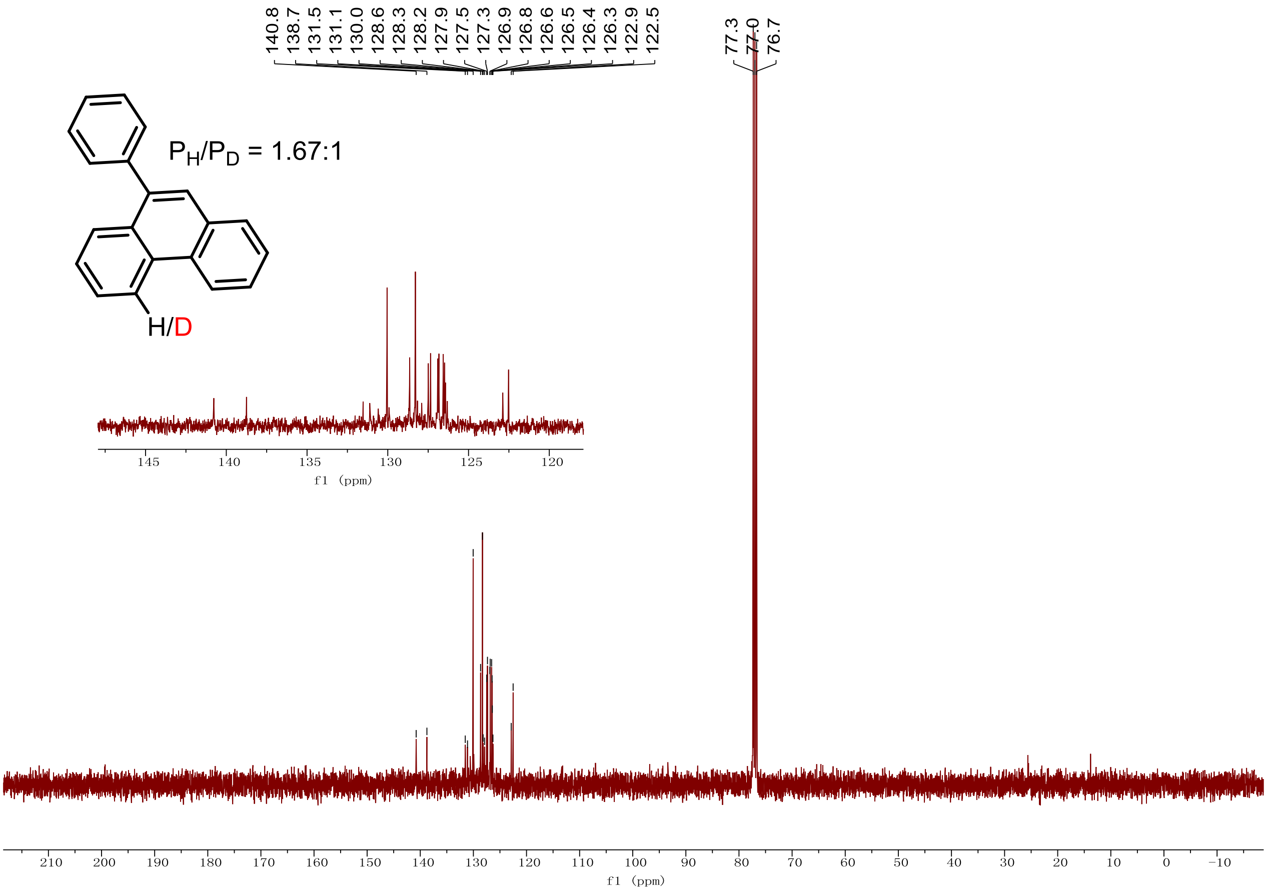

Supplementary Reference

1. Elsherbini, M. & Moran, W. J. Scalable electrochemical synthesis of diaryliodonium salts. *Org. Biomol. Chem.* **19**, 4706–4711 (2021).
2. Okuda, Y. *et al.* Chemodivergent synthesis of polycyclic aromatic diarylamines and carbazoles by thermal/photochemical process-controlled dephosphinylative functionalizations of amino(phosphinyl)arenes. *J. Org. Chem.* **89**, 7747–7757 (2024).
3. Li, W. *et al.* Synthesis of fluoren-9-ones via Pd-catalyzed annulation of 2-iodobiphenyls with vinylene carbonate. *Chem. Asian J.* **19**, e202301040 (2024).
4. Pan, S. *et al.* Synthesis of triphenylenes starting from 2-iodobiphenyls and iodobenzenes via palladium-catalyzed dual C–H activation and double C–C bond formation. *Org. Lett.* **18**, 5192–5195 (2016).
5. Campo, M. A. *et al.* Aryl to aryl palladium migration in the Heck and Suzuki coupling of o-halobiaryls. *J. Am. Chem. Soc.* **129**, 6298–6307 (2007).
6. Choi, H. *et al.* Mechanistic approach toward the C4-selective amination of pyridines via nucleophilic substitution of hydrogen. *Angew. Chem. Int. Ed.* **63**, e202401388 (2024).
7. Liu, H., El-Salfiti, M. & Lautens, M. Expeditious synthesis of tetrasubstituted helical alkenes by a cascade of palladium-catalyzed C–H activations. *Angew. Chem. Int. Ed.* **51**, 9846–9850 (2012).
8. Li, P. *et al.* Intramolecular remote C–H activation via sequential 1,4-palladium migration to access fused polycycles. *Org. Lett.* **21**, 6765–6769 (2019).
9. Jiang, H. *et al.* An approach to tetraphenylenes via Pd-catalyzed C–H functionalization. *Org. Lett.* **18**, 2032–2035 (2016).
10. Golling, F. E. *et al.* Concise synthesis of 3D π-extended polyphenylene cylinders. *Angew. Chem. Int. Ed.* **53**, 1525–1528 (2014).
11. Liu, M. *et al.* meta-Selective O-arylation of cyclic diaryliodonium salts with phenols via aryne intermediates. *Org. Lett.* **25**, 2777–2781 (2023).
12. Zhang, X., Sarkar, S. & Larock, R. C. Synthesis of naphthalenes and 2-naphthols by the electrophilic cyclization of alkynes. *J. Org. Chem.* **71**, 236–243 (2006).
13. Yao, T., Campo, M. A. & Larock, R. C. Synthesis of polycyclic aromatics and heteroaromatics via electrophilic cyclization. *J. Org. Chem.* **70**, 3511–3517 (2005).
14. Lv, W. *et al.* Palladium-catalyzed Catellani-type bis-silylation and bis-germanylation of aryl iodides and norbornenes. *J. Org. Chem.* **83**, 12683–12693 (2018).
15. Roy, V. J., Chakraborty, J. & Raha Roy, S. Catalytic π–π interactions triggered photoinduced synthesis of biaryls. *Org. Lett.* **26**, 183–187 (2024).
16. Dutta, U., Lupton, D. W. & Maiti, D. Aryl nitriles from alkynes using tert-butyl nitrite: metal-free approach to C–C bond cleavage. *Org. Lett.* **18**, 860–863 (2016).

17. Wang, S. *et al.* Palladium-catalyzed carbonylative synthesis of polycyclic 3,4-dihydroquinolin-2(1H)-one scaffolds containing perfluoroalkyl and carbonyl units. *Org. Lett.* **25**, 5314–5318 (2023).
18. Bu, M. J., Lu, G. P. & Cai, C. Ascorbic acid promoted [4+2] benzannulation: a mild, operationally simple approach to the synthesis of phenanthrenes. *Org. Chem. Front.* **3**, 630–634 (2016).
19. Appa, R. M. *et al.* Structure-controlled Au@Pd NPs/rGO as robust heterogeneous catalyst for Suzuki coupling in biowaste-derived water extract of pomegranate ash. *Appl. Organomet. Chem.* **35**, e6188 (2021).
20. Ho, C. Y., Chan, C. W. & He, L. Catalytic asymmetric hydroalkenylation of vinylarenes: electronic effects of substrates and chiral N-heterocyclic carbene ligands. *Angew. Chem. Int. Ed.* **54**, 4512–4516 (2015).
21. Liu, X. *et al.* Application of sulfoxonium ylides or iodonium ylides in rhodium-catalyzed synthesis of phenanthrenes. *Adv. Synth. Catal.* **366**, 1744–1750 (2024).
22. Gao, P. *et al.* Mechanochemical activation of aryl diazonium salts: synthesis of polycyclic (hetero)aromatics. *J. Org. Chem.* **89**, 12197–12203 (2024).
23. Chatterjee, T., Lee, D. S. & Cho, E. J. Extended study of visible-light-induced photocatalytic [4+2] benzannulation: synthesis of polycyclic (hetero)aromatics. *J. Org. Chem.* **82**, 4369–4378 (2017).
24. Ma, W. T. *et al.* Ru(II)-catalyzed P(III)-assisted C8-alkylation of naphthphosphines. *Chem. Commun.* **58**, 7152–7155 (2022).
25. Ding, Y. X. *et al.* Rhodium-catalyzed asymmetric hydrogenation of all-carbon aromatic rings. *Angew. Chem. Int. Ed.* **61**, e202205623 (2022).
26. Mann, J. A. & Dichtel, W. R. Improving the binding characteristics of tripodal compounds on single-layer graphene. *ACS Nano* **7**, 7193–7199 (2013).
27. Takahashi, I. *et al.* Brønsted acid-catalyzed hydroarylation of unactivated alkynes in a fluoroalcohol-hydrocarbon biphasic system: construction of phenanthrene frameworks. *Chem. Commun.* **55**, 9267–9270 (2019).
28. Chai, D. I. & Lautens, M. Tandem Pd-catalyzed double C–C bond formation: effect of water. *J. Org. Chem.* **74**, 3054–3061 (2009).
29. Jin, R. *et al.* Merging photoredox catalysis with Lewis acid catalysis: activation of carbon–carbon triple bonds. *Chem. Commun.* **52**, 9909–9912 (2016).
30. Lin, J. *et al.* Direct synthesis of naphthalenes by nickel-catalyzed cascade cyclization of o-vinyl chlorobenzenes with internal alkynes. *Eur. J. Org. Chem.* **2021**, 6764–6767 (2021).
31. Zhang, J. *et al.* Metal-free cycloisomerizations of o-alkynylbiaryls. *Chem. Commun.* **54**, 12455–12458 (2018).
32. Hossain, M. M. *et al.* π-Extended dibenzo[*g, p*]chrysenes. *Org. Chem. Front.* **8**, 2393–2401 (2021).
33. Arsenyan, P., Lapcinska, S., Ivanova, A. & Vasiljeva, J. Peptide functionalization through the

generation of selenocysteine electrophile. *Eur. J. Org. Chem.* **2019**, 4951–4961 (2019).

34. Yu, J., Yan, H. & Zhu, C. Synthesis of multiply substituted polycyclic aromatic hydrocarbons by iridium-catalyzed annulation of ring-fused benzocyclobutenol with alkyne through C–C bond cleavage. *Angew. Chem. Int. Ed.* **55**, 1143–1146 (2016).
35. Qian, C. G. & Xiao, B. 4,5-Disubstituted pyrenes from phenangermoles. *Org. Chem. Front.* **10**, 640–644 (2023).
36. Akhmetov, V., Feofanov, M., Sharapa, D. I. & Amsharov, K. Alumina-mediated π-activation of alkynes. *J. Am. Chem. Soc.* **143**, 15420–15426 (2021).
37. Murai, M. *et al.* Bismuth-catalyzed synthesis of polycyclic aromatic hydrocarbons with a phenanthrene backbone via cyclization and aromatization of 2-(2-arylphenyl) vinyl ethers. *Org. Lett.* **16**, 4134–4137 (2014).
38. Suzuki, N. *et al.* Aluminium-mediated aromatic C–F bond activation: regioswitchable construction of benzene-fused triphenylene frameworks. *Chem. Commun.* **52**, 12948–12951 (2016).
39. Ogawa, N. *et al.* Synthesis of polycyclic spirocarbocycles via acid-promoted ring-contraction/dearomative ring-closure cascade of oxapropellanes. *Org. Lett.* **21**, 7563–7567 (2019).
40. McAtee, C. C., Riehl, P. S. & Schindler, C. S. Polycyclic aromatic hydrocarbons via iron(III)-catalyzed carbonyl–olefin metathesis. *J. Am. Chem. Soc.* **139**, 2960–2963 (2017).
41. Shi, G. *et al.* Synthesis of fluorenes starting from 2-iodobiphenyls and CH2Br2 through palladium-catalyzed dual C–C bond formation. *Org. Lett.* **18**, 2958–2961 (2016).
